# Selective autophagy of RIPosomes maintains innate immune homeostasis during bacterial infection

**DOI:** 10.15252/embj.2022111289

**Published:** 2022-10-11

**Authors:** Subhash Mehto, Kautilya Kumar Jena, Rina Yadav, Swatismita Priyadarsini, Pallavi Samal, Sivaram Krishna, Kollori Dhar, Ashish Jain, Nishant Ranjan Chauhan, Krushna C Murmu, Ramyasingh Bal, Rinku Sahu, Pundrik Jaiswal, Bhabani Sankar Sahoo, Srinivas Patnaik, Thomas A Kufer, Tor Erik Rusten, Swati Chauhan, Punit Prasad, Santosh Chauhan

**Affiliations:** ^1^ Cell Biology and Infectious Diseases Unit, Department of Infectious Disease Biology Institute of Life Sciences Bhubaneswar India; ^2^ Regional Centre for Biotechnology, NCR Biotech Science Cluster Faridabad India; ^3^ Institute of Life Sciences Bhubaneswar India; ^4^ Centre for Cancer Cell Reprogramming, Institute of Clinical Medicine, Faculty of Medicine University of Oslo Oslo Norway; ^5^ Department of Molecular Cell Biology, Institute for Cancer Research Oslo University Hospital Oslo Norway; ^6^ Epigenetic and Chromatin Biology Unit Institute of Life Sciences Bhubaneswar India; ^7^ School of Biotechnology KIIT University Bhubaneswar India; ^8^ Department of Immunology, Institute of Nutritional Medicine University of Hohenheim Stuttgart Germany; ^9^ CSIR–Centre For Cellular And Molecular Biology (CCMB) Hyderabad India; ^10^ Present address: Division of Immunology, Boston Children's Hospital Harvard Medical School Boston MA USA

**Keywords:** autophagy, inflammation, Irgm1, NOD1/2‐RIPK2‐NF‐κB, RIPosomes, Autophagy & Cell Death, Microbiology, Virology & Host Pathogen Interaction

## Abstract

The NOD1/2‐RIPK2 is a key cytosolic signaling complex that activates NF‐κB pro‐inflammatory response against invading pathogens. However, uncontrolled NF‐κB signaling can cause tissue damage leading to chronic diseases. The mechanisms by which the NODs‐RIPK2‐NF‐κB innate immune axis is activated and resolved remain poorly understood. Here, we demonstrate that bacterial infection induces the formation of endogenous RIPK2 oligomers (RIPosomes) that are self‐assembling entities that coat the bacteria to induce NF‐κB response. Next, we show that autophagy proteins IRGM and p62/SQSTM1 physically interact with NOD1/2, RIPK2 and RIPosomes to promote their selective autophagy and limit NF‐κB activation. IRGM suppresses RIPK2‐dependent pro‐inflammatory programs induced by *Shigella* and *Salmonella*. Consistently, the therapeutic inhibition of RIPK2 ameliorates *Shigella* infection‐ and DSS‐induced gut inflammation in Irgm1 KO mice. This study identifies a unique mechanism where the innate immune proteins and autophagy machinery are recruited together to the bacteria for defense as well as for maintaining immune homeostasis.

## Graphical abstract

Bacterial infection induces the formation of RIPK2 oligomers that coat the bacteria to enhance NF‐κB response, a process that is counteracted by selective autophagy of RIPK2 oligomers.

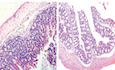

## Introduction

NOD1 and NOD2 (NODs) are cytosolic pattern recognition receptors (PRRs) that detect pathogen‐associated molecular patterns (PAMPs), iE‐DAP (D‐glutamyl‐meso‐diaminopimelic acid), and MDP (muramyl dipeptide; Tanabe *et al*, 2004; Laroui *et al*, 2011; Caruso *et al*, 2014). Stimulated NODs interact and activate adaptor protein RIPK2 (RICK, RIP2) for a cascade of events resulting in NF‐κB activation and pro‐inflammatory cytokine release (Girardin *et al*, 2001; Caruso *et al*, 2014). NODs‐RIPK2 is one of the major innate immune axis that senses intracellular bacterial pathogens and mounts a powerful NF‐κB‐dependent pro‐inflammatory cytokine response to eliminate bacteria.

An important step for triggering pro‐inflammatory innate immune pathways is the oligomerization of PRRs and/or adaptor proteins (Hou *et al*, 2011; Xie *et al*, 2013; Xu *et al*, 2014; Wu & Fuxreiter, 2016). For example, activation of RIG‐I signaling induces polymerization of MAVS to form detergent‐resistant, protease‐resistant, and self‐perpetuating prion‐like aggregates (filamentous structure) to activate and transmit antiviral signaling (Hou *et al*, 2011; Cai *et al*, 2014; Wu *et al*, 2014; Xu *et al*, 2014). The inflammasome is another classical example, where several self‐polymerized inflammatory proteins interact with each other to form multiprotein signalosomes that execute pro‐inflammatory responses (Cai *et al*, 2014; Lu *et al*, 2014, 2016). Recently, structural studies demonstrated that purified RIPK2 polymerizes to form filamentous structures that are important for NODs‐dependent NF‐κB signaling (Gong *et al*, 2018; Pellegrini *et al*, 2018). Also, ectopically expressed RIPK2 was shown to form detergent‐insoluble higher‐order oligomeric structures upon *Shigella* infection (Ellwanger *et al*, 2019). These structures were termed “RIPosomes.” To date, it is not clear whether endogenous RIPK2 forms RIPosomes and if yes, how they regulate NF‐κB signaling upon bacterial infection.

The NODs‐RIPK2‐NF‐κB pro‐inflammatory axis is critical for clearing pathogens; however, its aberrant activation can cause chronic inflammation, oncogenesis, and autoimmune disease (Miceli‐Richard *et al*, 2001; Kanazawa *et al*, 2005; Henckaerts & Vermeire, 2007; Caso *et al*, 2015; Taniguchi & Karin, 2018). To avoid these conditions, a balanced immune state needs to be maintained by negative feedback mechanisms. Inflammophagy, the autophagic degradation of inflammatory aggregates and proteins, is an emerging mechanism to limit inflammation to maintain innate immune homeostasis (Chauhan *et al*, 2021; Deretic, 2021). Several of the cytosolic PRRs and adaptor proteins including RIG‐I, NLRP3, AIM2, cGAS, MAVS, and STING are targeted by p62‐ or NDP52‐dependent selective autophagy to dampen the excess inflammation (Shi *et al*, 2012; Liu *et al*, 2016; Du *et al*, 2018; Prabakaran *et al*, 2018; He *et al*, 2019; Mehto *et al*, 2019b; Jena *et al*, 2020; Chauhan *et al*, 2021; Deretic, 2021). To date, it is unclear how selective autophagy contributes to fine‐tuning of the NODs‐RIPK2‐NF‐κB pathways.

Genetic mutations in the Immunity‐related GTPase M (IRGM) gene or promoter are suggested to increase susceptibility to several inflammatory and infectious diseases including Crohn's disease, tuberculosis, sepsis, and ankylosing spondylitis (Massey & Parkes, 2007; Parkes *et al*, 2007; Intemann *et al*, 2009; Lu *et al*, 2013; Kimura *et al*, 2014; Lin *et al*, 2016; Xia *et al*, 2017; Yao *et al*, 2018). IRGM is also a critical factor for antimicrobial autophagy (Singh *et al*, 2006; Chauhan *et al*, 2015). IRGM interacts with autophagy and lysosome regulatory proteins to stimulate autophagosome/lysosome biogenesis for efficient degradation of cargo including microbes (Singh *et al*, 2010; Chauhan *et al*, 2015; Kumar *et al*, 2018, 2020). IRGM plays a prominent role in the inflammophagy of several innate immune sensing proteins including NLRP3, RIG‐I, and cGAS to limit inflammasome and interferon responses (Mehto *et al*, 2019a, 2019b; Jena *et al*, 2020; Chauhan *et al*, 2021; Nath *et al*, 2021). IRGM interacts with NOD2 to scaffold the signaling events for xenophagy (Chauhan *et al*, 2015); however, it is unclear whether IRGM also could regulate the activity of NODs and RIPK2 (RIPosomes) to control NF‐κB pro‐inflammatory cytokine response.

Here, we demonstrate that pathogenic bacteria induce the formation of endogenous RIPosomes in the proximity of the bacteria to activate the NF‐κB cytokine response. Further, we found that NODs, RIPK2, and RIPosomes are targets of selective autophagy. The autophagy scaffolding proteins, IRGM and p62 physically interact with NODs, RIPK2, and RIPosomes, and using the canonical autophagy machinery coordinate their selective degradation to limit cytokine responses. In agreement, the global transcriptomic analysis revealed that during *Salmonella* and *Shigella* infection, IRGM suppresses multiple RIPK2‐dependent pro‐inflammatory pathways including NF‐κB and interferon (IFN) response. Consistently, in animal studies, inhibition of RIPK2 using GSK583 ameliorated shigellosis‐ and dextran sodium sulfate (DSS)‐induced gut inflammation, and pathology in *Irgm1* ^*KO*^ mice. Together, this study delineates new cell‐autonomous mechanisms of NODs‐RIPK2‐dependent pro‐inflammatory response and its resolution by selective autophagy. Further, our study also suggests that inhibition of RIPK2 could be a good therapeutic strategy for suppression of gut inflammation associated with IRGM depletion, a risk factor in the progression of Crohn's disease.

## Results

### RIPosomes recruit over the bacteria

**Figure 1:**
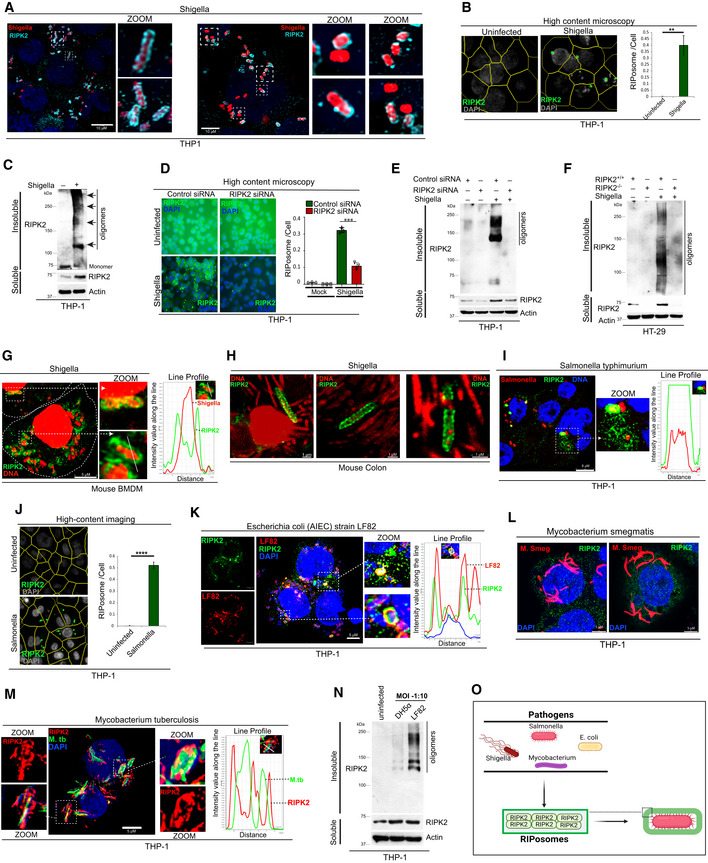
Pathogenic bacteria trigger the formation of RIPosomes that recruit over the bacteria ARepresentative confocal images of RIPosomes in THP‐1 cells infected with a red fluorescent protein (RFP) expressing *Shigella flexneri* (MOI 1:25, 8 h). Scale bar, 10 μm. Zoom panels are digital magnifications.BRepresentative high‐content microscopy images (see Appendix Fig S1D for full images; yellow masks represent software algorithms‐defined cell boundaries) of RIPosomes in THP‐1 cells infected with *S. flexneri* (MOI 1:25, 8 h). About 50,000 cells were plated per well and RIPosomes were screened in 35 fields per well in three technical replicates. Right panel, the graph depicts an average number of RIPosomes/cell, which is calculated from three biological replicates, Mean ± SD. ***P < 0.005, Student's unpaired *t*‐test.CWestern blot analysis of soluble and insoluble fractions of *S. flexneri*‐infected THP‐1 cells (MOI 1:25, 8 h). Arrow indicates oligomers of RIPK2.DLeft panel, representative high‐content microscopy image of control and RIPK2 knockdown THP‐1 cells infected with *S. flexneri* (MOI 1:25, 8 h). Right panel, the graph depicts an average number of RIPosomes/cell, which is calculated from three biological replicates, Mean ± SD. ***P < 0.0005, Student's unpaired *t*‐test.E, FWestern blot analysis of soluble and insoluble fractions of *S. flexneri*‐infected (E) control siRNA and RIPK2 siRNA transfected THP‐1 cells (F) Control CRISPR (RIPK2^+/+^) cells and RIPK2 CRISPR knockout (RIPK2^−/−^) HT‐29 cells.GRepresentative confocal images of RIPosomes in mouse BMDMs infected with *S. flexneri* (MOI 1:25, 8 h). Line profile: co‐localization analysis using line intensity profile. Scale bar, 5 μm. Zoom panels are digital magnifications. DNA is stained with DAPI (pseudo‐colored red for better contrast).HRepresentative confocal images of mouse colon tissues showing recruitment of RIPK2 over the *S. flexneri*. Scale bar, 1 μm. Zoom panels are super‐resolution confocal images. DNA is stained with DAPI (pseudo‐colored red for better contrast).IRepresentative confocal images of RIPosomes in THP‐1 cells infected with RFP expressing *Salmonella typhimurium* (MOI 1:5, 4 h). Line profile: co‐localization analysis using line intensity profile. Scale bar, 8 μm. Zoom panels are digital magnifications. DNA is stained with DAPI.JLeft panel, representative high‐content microscopy images (yellow masks represent software algorithms‐defined cell boundaries) of RIPosomes in THP‐1 cells infected with *S. typhimurium* (MOI 1:5, 4 h). The same cell numbers and conditions are used as indicated in (B). Right panel, the graph depicts an average number of RIPosomes/cell. Mean ± SD, *n* = 4 (biological replicates), ****P < 0.00005, Student's unpaired *t*‐test. DNA is stained with DAPI.K–MRepresentative confocal images of RIPosomes in THP‐1 cells infected with (K) *E. coli* LF82 strain (MOI 1: 10, 8 h), (LPS antibody is used to stain LF82) (L) mCherry expressing *M. smegmatis* (MOI 1:10, 4 h) (M) mCherry (pseudocolored to green) expressing *M. tuberculosis* (MOI 1:10, 8 h). Line profile: co‐localization analysis using line intensity profiles. Scale bar, 5 μm. Zoom panels are digital magnifications. DNA is stained with DAPI.NWestern blot analysis of soluble and insoluble fractions from THP‐1 cells, uninfected or infected with *E. coli* DH5α (MOI 1:10, 8 h) or *E. coli* LF82 strains (MOI 1:10, 8 h).OPictorial representation of data obtained in this figure shows that bacterial infection triggers oligomerization of RIPK2 or RIPosome formation that coats bacteria. Source data are available online for this figure.

The existence of endogenous RIPK2 oligomeric structures (RIPosomes) is not reported. We found that infection of macrophage‐like differentiated THP‐1 cells with *Shigella flexneri* induces RIPosome formation. RIPosomes were detected only in infected cells but not in control cells (Appendix Fig S1A). Several of the RIPosomes were recruited over the bacteria (Fig 1A and Appendix Fig S1A–C and Movie EV1). However, not all the intracellular bacteria were covered with oligomeric RIPK2 puncta (Fig 1A). High‐content microscopy was performed to quantitate the RIPosomes present in the cell (Fig 1B and Appendix Fig S1D). The presence of endogenous RIPK2 oligomeric structures was further confirmed by Western blotting of detergent‐insoluble fractions from *Shigella*‐infected cells (Fig 1C). To confirm specificity, next we depleted RIPK2 and tested the formation of RIPosomes by high‐content microscopy and Western blotting. *Shigella*‐induced RIPosomes were dramatically reduced upon RIPK2 siRNA knockdown in THP‐1 macrophages (Fig 1D and E) or RIPK2 CRISPR knockout HT‐29 cells (henceforth, RIPK2^−/−^; Fig 1F).

Next, we infected mouse bone marrow‐derived macrophages (BMDMs), HT‐29 colon epithelial cells, and mouse embryonic fibroblast (MEF) cells with *Shigella*. RIPosomes were recruited over and adjacent to the bacteria in the infected cells (Fig 1G and Appendix Fig S1E–G), whereas the uninfected cells were devoid of them (Appendix Fig S1F). Thus, *Shigella* infection can induce RIPosome formation in different cell types. Next, C57BL/6 mice were infected by the intraperitoneal injection of *Shigella* (Yang *et al*, 2014), and immunohistochemistry with the colon tissues was performed. Several of the bacteria were covered with RIPosomes in colon tissues (Fig 1H).

To test whether RIPosome formation is specific to *Shigella* infection or is a generalized event during bacterial infection, we infected THP‐1 cells with *Salmonella typhimurium*. Like *Shigella*, *Salmonella* enhanced the oligomerization of RIPK2 (Appendix Fig S1H) and triggered RIPosome formation (Fig 1I and J). Furthermore, infection with the Crohn's disease‐associated adherent invasive *Escherichia coli* (AIEC) strain LF82 (Glasser *et al*, 2001) also prompted the formation of RIPosomes that were coating the bacteria (Fig 1K). Next, we compared RIPosome inducing capacity of pathogenic *Mycobacterium tuberculosis* (*M.tb*, H37Rv) and nonpathogenic *Mycobacterium smegmatis* (*M. smeg*, MC^2^155). Surprisingly, only *M.tb* triggered the massive formation of RIPosomes over the bacteria (Fig 1L and M and Appendix Fig S1I and Movie EV2), indicating that only pathogenic bacteria could activate RIPosome formation. To further corroborate this notion, we compared the RIPK2 oligomerization capacity of nonpathogenic *E. coli* DH5α and pathogenic AIEC LF82. Indeed, LF82 was more efficient in triggering the oligomerization of RIPK2 (Fig 1N).

No RIPosomes were formed upon infection with GFP‐tagged vesicular stomatitis virus (Appendix Fig S1J). Similarly, the NOD1 and NOD2 ligands, iE‐DAP or MDP were not able to trigger visible RIPosome formation (Appendix Fig S1K). However, iE‐DAP or MDP increased oligomerization of RIPK2 in the insoluble fraction of cells (Appendix Fig S1L and M), suggesting that they have a reduced capacity to induce RIPK2 oligomerization.

Altogether, these findings demonstrate that bacterial infection triggers RIPosome formation that is recruited at cytoinvasive bacteria both *in vitro* and *in vivo* conditions (Fig 1O).

### RIPosomes are self‐assembling structures that promote NODs oligomerization

**Figure 2:**
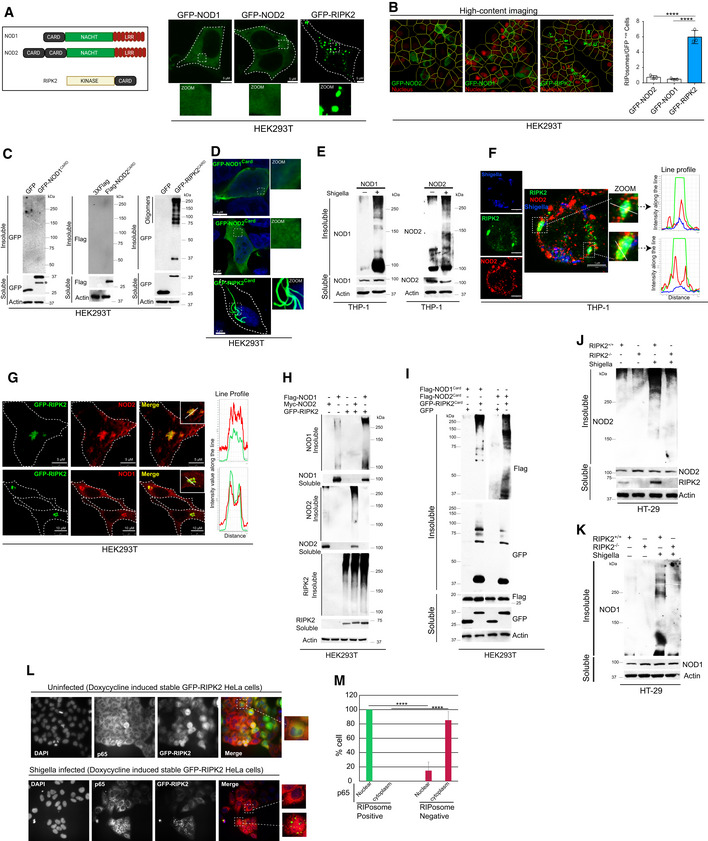
RIPK2 oligomers provide a platform for NODs oligomerization ALeft panel, the domain organization map of NOD1, NOD2, and RIPK2. Right panel, representative confocal images of immunofluorescence assays performed in HEK293T cells transfected with GFP‐NOD1, GFP‐NOD2, and GFP‐RIPK2 plasmids for 9 h. Scale bar 5 μm.BLeft panel, representative high‐content microscopy images of HEK293T cells transfected with GFP‐NOD1, GFP‐NOD2, and GFP‐RIPK2 plasmids for 9 h. Right panel, the graph depicts the average number of RIPosomes per GFP‐positive cell. About 15,000 cells were plated per well and RIPosomes were screened in 35 fields per well. Mean ± SD, *n* = 3 (biological replicates), ****P < 0.00005, ordinary one‐way ANOVA (Tukey's multiple comparisons test).CWestern blot analysis of soluble and insoluble fractions of HEK293T cells transfected with the CARD domain‐containing region of NOD1, NOD2, and RIPK2.DRepresentative confocal images of HEK293T cells transfected with GFP‐NOD1^CARD^, GFP‐NOD2^CARD^, and GFP‐RIPK2^CARD^. Scale bar 5 μm.EThe soluble and insoluble fractions of *S. flexneri*‐infected THP‐1 (MOI 1:25, 8 h) cell lysates were subjected to Western blot analysis with indicated antibodies.FRepresentative confocal images of immunofluorescence assays conducted with THP‐1 cells infected with RFP expressing *S. flexneri* (pseudo‐colored, blue) (MOI 1:25, 8 h). Line profile: co‐localization analysis using line intensity profiles. Scale bar, 5 μm. Zoom panels are digital magnifications.GRepresentative confocal images of immunofluorescence assays performed with HEK293T cells transfected with GFP‐RIPK2 and Flag‐NOD2 (upper panel) or Flag‐NOD1 (lower panel). Line profile: co‐localization analysis using line intensity profiles. Scale bar, 5 or 10 μm as indicated in figures. Inset zoom panels are digital magnifications.H, IHEK293T cells were transfected with (H) full length and (I) CARD domains of NOD1, NOD2, and RIPK2 followed by Western blot analysis with soluble and insoluble fractions using indicated antibodies.J, KWestern blot analysis of soluble and insoluble fractions of RIPK2^+/+^ and RIPK2^−/−^ HT‐29 cells infected with *S. flexneri* (MOI 1:25, 8 h) with indicated antibodies.L, MRepresentative immunofluorescence images of doxycycline‐inducible GFP‐RIPK2 expressing HeLa cells. (L) Upper panel, uninfected. Lower panel, *S. flexneri*‐infected MOI 1:25, 4 h). Immunostaining was performed with the p65 antibody (red) and DNA stained with DAPI (Blue). (M) The graph indicates % of cells that are RIPosomes positive or negative with nuclear/cytoplasmic p65 (5 fields (each group), Mean ± SD, *n* = 3). ****P < 0.00005, Student's unpaired *t*‐test. Source data are available online for this figure.

RIPK2 is a common adaptor protein for NOD1 or NOD2‐dependent NF‐κB signaling. Several inflammatory proteins utilize their CARD domain to self‐oligomerize and/or to hetero‐oligomerize with downstream cognate signaling adaptor proteins for activation and signal transduction (Park, 2019). All three proteins, NOD1, NOD2, and RIPK2 possess CARD domain/s (Fig 2A); however, only RIPK2 self‐oligomerized and formed RIPosomes (Fig 2A and B and Appendix Fig S2A) and fractionated in the insoluble (pellet) portion of cells (Appendix Fig S2B and C). Further, the CARD domain of RIPK2 (RIPK2^CARD^) formed high‐order oligomers (Fig 2C) and large filamentous structures (Fig 2D and Movie EV3), whereas the CARD domain of NOD1^CARD^ and NOD2^CARDs^ domain failed to do so (Fig 2C and D). These results suggest that only RIPK2 (but not NODs) can self‐oligomerize using its CARD domain.

Interestingly, upon *Shigella* infection, endogenous NOD2 formed puncta (Appendix Fig S2D) and the oligomeric aggregates of both NOD1 and NOD2 were increased in the insoluble cell fractions (Fig 2E). NOD2 puncta were found to be juxtaposed to the bacteria (Appendix Fig S2D) where they co‐localized/juxtaposed with RIPosomes (Fig 2F and Appendix Fig S2E). This apparent discrepancy between overexpressed and endogenous results could be due to the presence of endogenous RIPK2 in THP‐1 cells (compared with HEK293T) whose self‐assembly may have prompted co‐oligomerization of NOD1/2 leading to puncta formation. Indeed, both the NOD1 and NOD2 formed insoluble oligomeric structures when co‐expressed with RIPK2, as apparent from immunofluorescence (Fig 2G and Appendix Fig S2F), quantitative high‐content microscopy (Appendix Fig S2G), and soluble/insoluble fractionation assays (Fig 2H). NODs were perfectly co‐localized with RIPK2 in these structures (Fig 2G and Appendix Fig S2F and G). We termed these structures NODo‐RIPosomes. These data were further supported by experiments where we found NOD1^CARD^ or NOD2^CARDs^ domain/s start oligomerizing once they are co‐expressed with the RIPK2^CARD^ (Fig 2I). Also, the NOD1^CARD^ or NOD2^CARD^ formed the punctate structures when co‐expressed with RIPK2^CARD^ (Appendix Fig S2H and I). Taken together, the data suggest that RIPK2 facilitates the oligomerization of NOD proteins via CARD domain/s. This notion was further tested in endogenous conditions by assessing the oligomerization of NOD proteins in the absence of RIPK2. *Shigella*‐induced oligomerization of endogenous NODs was reduced in RIPK2^−/−^ HT‐29 cells compared with WT (Fig 2J and K). Thus, we conclude that RIPK2 self‐assembling property is critical for inducing oligomerization of NOD1 and NOD2. Conversely, we noticed that the NOD proteins, in turn, enhanced the self‐oligomerization capacity of RIPK2 (Appendix Fig S2J and K).

The formation of RIPosomes is important for the activation of NF‐κB signaling (Gong *et al*, 2018; Pellegrini *et al*, 2018). We used a HeLa cell line expressing stable doxycycline‐inducible human GFP‐RIPK2 (Ellwanger *et al*, 2019) to evaluate whether *Shigella* infection‐induced RIPosomes prompt nuclear translocation of NF‐κB‐p65, a signature of NF‐κB activation. The p65 was not translocated into the nucleus in uninfected GFP‐RIPK2 expressing cells (Fig 2L, upper panel), whereas a distinct nuclear translocation was observed in the *Shigella*‐infected cells in which the RIPosomes are formed (Fig 2L, lower panel and M). However, within the *Shigella*‐infected cells, the cells that are negative for RIPosomes, a majority had cytoplasmic p65 (Fig 2L, lower panel and M). Also, the nuclear translocation of phospho‐p65 (Ser536) is significantly induced upon *Shigella* infection in GFP‐RIPK2 overexpressing HeLa cells (Appendix Fig S2L). The data suggest that the biogenesis of RIPosome is important for the activation of NF‐κB response.

To evaluate whether the NODo‐RIPosomes could also induce NF‐kB activation, we electroporated purified RIPosomes and NODo‐RIPosomes into HEK293T cells. Both RIPosomes and NODo‐RIPosomes induced NF‐κB activation measured by luciferase reporter assays, where NODo‐RIPosomes were consistently more efficient than RIPosomes in triggering NF‐κB activation (Appendix Fig S2M).

### NODs, RIPK2, and RIPosomes are the target of selective autophagy

**Figure 3:**
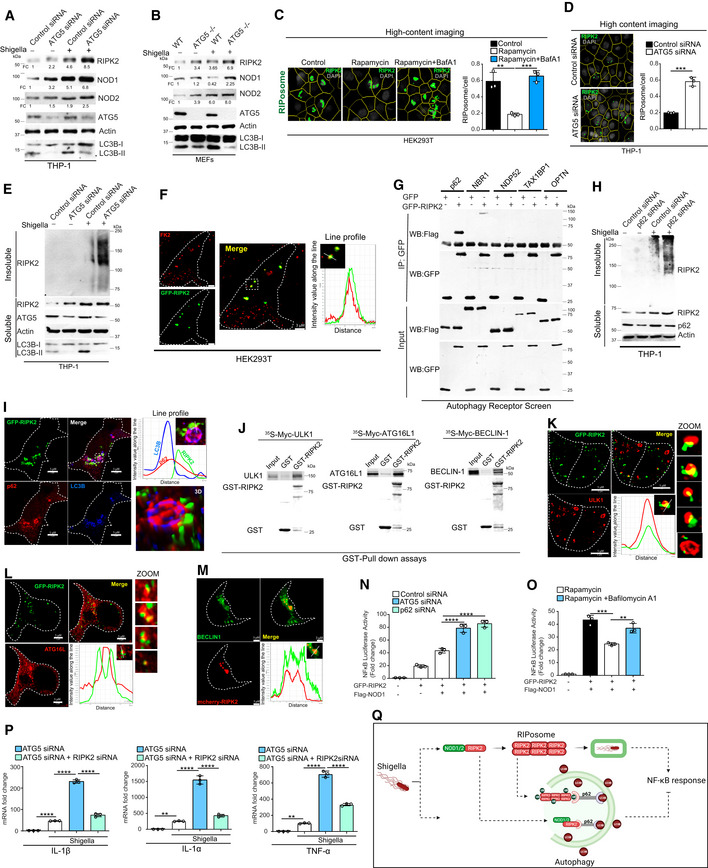
NODs, RIPK2, and RIPosomes are degraded by p62‐dependent selective autophagy A, BWestern blot analysis with cell lysate of uninfected and *S. flexneri*‐infected (MOI 1:25, 8 h) (A) control and ATG5 siRNA knockdown THP‐1 cells, (B) wild‐type (WT) and ATG5 knockout (ATG5^−/−^) MEF cells with indicated antibodies. Densitometric analysis was performed using Image J software. FC, fold change.CLeft panel, representative high‐content microscopy images of RIPosomes in HEK293T cells that are control cells or cells treated with rapamycin (500 nM, 4 h) or cells treated with rapamycin (500 nM, 4 h) and bafilomycin A1 (BafA1, 300 nM). Right panel, the graph depicts the average number of RIPosomes/cell. About 15,000 cells were plated per well and RIPosomes were screened in 35 fields per well. Mean ± SD, *n* = 3 (biological replicates), **P < 0.005 and, ***P < 0.0005, ordinary one‐way ANOVA (Tukey's multiple comparisons test).DLeft panel, representative high‐content microscopy images of RIPosomes in control and ATG5 siRNA transfected THP‐1 cells infected with *S. flexneri* (8 h). Right panel, the graph depicts average number of RIPosomes/cell. About 50,000 cells were plated per well and RIPosomes were screened in 35 fields per well. Mean ± SD, *n* = 3 (biological replicates), ***P < 0.0005, Student's unpaired *t*‐test.EThe soluble and insoluble fractions of *S. flexneri*‐infected (MOI 1:25, 8 h) control and ATG5 siRNA knockdown THP‐1 cells were subjected to Western blot analysis with indicated antibodies.FRepresentative confocal images of HEK293T cells transfected with GFP‐RIPK2 and immunostained with anti‐FK2 antibodies. Line profile: co‐localization analysis using line intensity profiles. Scale bar, 3 μm.GAutophagy receptor screen using co‐immunoprecipitation (Co‐IP) assay to analyze the interaction between GFP‐RIPK2 and Flag‐p62 or Flag‐NBRI or Flag‐NDP52 or Flag‐TAX1BP1 or Flag‐OPTINEURIN in HEK293T cell lysate.HWestern blot analysis of soluble and insoluble fractions of control and p62 siRNA transfected and S*. flexneri*‐infected THP‐1 cells (MOI 1:25, 8 h).IRepresentative confocal images of HEK293T cells expressing GFP‐RIPK2 (6 h) immunostained with p62 and LC3B antibodies. Zoom panels are digital magnifications.JGST pull‐down assay using purified GST or GST‐RIPK2 proteins with in vitro translated ^35^S radiolabeled myc‐ULK1 or myc‐ATG16L or myc‐BECLIN1.K–MRepresentative confocal images of HEK293T cells transfected with GFP‐RIPK2 or mcherry‐RIPK2 (9 h) and immunofluorescence assay performed with antibodies specific to (K) ULK1, (L) ATG16L (M) BECLIN1. Line profile: co‐localization analysis using line intensity profiles. Scale bar, 5 or 3 μm as indicated. Zoom panels are digital magnifications.NLuciferase assays were performed with ATG5 or p62 knockdown HEK293T cells transfected with NF‐κB luciferase reporter vector pGL4.32 NFκB‐RE, GFP‐RIPK2, and Flag‐NOD1 plasmids. Mean ± SD, *n* = 3 (biological replicates), ****P < 0.00005, ordinary one‐way ANOVA (Tukey's multiple comparisons test).OLuciferase assays performed with HEK293T cells transfected with NF‐κB luciferase reporter vector pGL4.32NFκB‐RE, GFP‐RIPK2, and Flag‐NOD1 plasmids followed by treatment with rapamycin (500 nM, 4 h) alone or in combination with bafilomycin A1 (300 nM, 5 h). Mean ± SD, *n* = 3 (biological replicates), **P < 0.005 and ***P < 0.0005, ordinary one‐way ANOVA (Tukey's multiple comparisons test). The control conditions and readings for Fig 3N and P are the same.PThe qRT–PCR analysis with total RNA isolated from the uninfected and *S. flexneri*‐infected (MOI 1:25, 6 h) control or ATG5 knockdown or ATG5 and RIPK2 double knockdown THP‐1 cells. Mean ± SD, *n* = 3 (biological replicates), **P < 0.005, and ****P < 0.00005, ordinary one‐way ANOVA (Tukey's multiple comparisons test).QPictorial representation of results obtained in this section where we found that p62‐dependent selective autophagy degrades NODs, RIPK2, and RIPosomes to suppress NF‐κB cytokine response. Source data are available online for this figure.

**Figure EV1:**
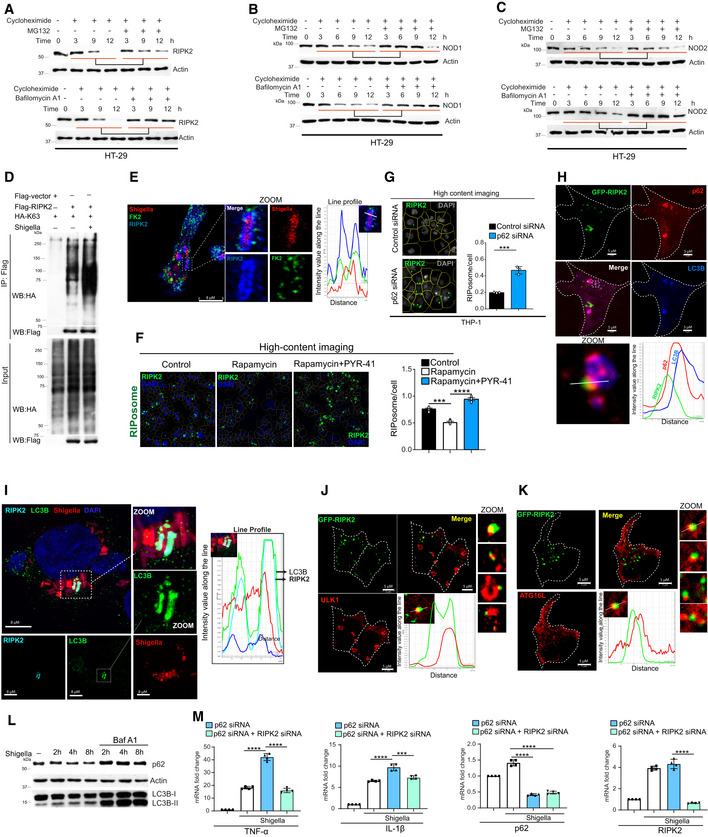
NODs, RIPK2, and RIPosomes are the target of selective autophagy A–CWestern blot analysis with the cell lysates of HT‐29 cells treated with cycloheximide (100 μg/ml) alone or in combination with Bafilomycin A1 (300 nM) or MG132 (20 μM) for different time points as indicated.DWestern blot analysis of IP experiments performed with lysates of HEK293T cells transiently transfected with Flag‐RIPK2 and HA‐K63‐Ubiquitin [variants of ubiquitin that can only be ubiquitinated at lysine 63 (K63)] and infected with *S. flexneri*, (MOI: 1:25, 6 h). IP was performed with Flag antibody and Western blotting was performed with indicated antibodies.ERepresentative confocal images of THP‐1 cells infected with RFP expressing *S. flexneri*, (MOI: 1:25, 8 h) and immunostained with anti‐RIPK2 and anti‐FK2 antibodies. Line profile: co‐localization analysis using line intensity profiles. Scale bar, 5 μm.FLeft panel, representative high‐content microscopy images (digitally zoomed) of RIPosomes in HEK293T cells. The HEK293T cells were transfected with GFP RIPK2 (100 ng/well of 96‐well plates) for 4 h followed by treatment with rapamycin (500 nM, 4 h), or rapamycin (500 nM, 4 h) and PYR‐41 (5 μM) for 5 h. Right panel, the graph depicts an average number of RIPosomes/cell. About 15,000 cells were plated per well and RIPosomes were screened in 35 fields per well. Mean ± SD, *n* = 3 (biological replicates), ***P < 0.0005, ****P < 0.00005, ordinary one‐way ANOVA (Tukey's multiple comparisons test).GLeft panels, representative high‐content microscopy images (digitally zoomed) of control or p62 knockdown THP‐1 cells infected with *S. flexneri* (6 hpi). About 50,000 cells were plated per well in a 96‐well plate and RIPosomes were screened in 35 fields per well. Right panel, the graph depicts the average number of RIPosomes/cell, which is calculated from three biological replicates. Mean ± SD. ***P < 0.0005, Student's unpaired *t*‐test.HRepresentative confocal images of HEK293T cells transfected with GFP‐RIPK2 (6 h) and immunostained with p62 and LC3B antibody. Zoom panel is a digital magnification. Line profile: co‐localization analysis using line intensity profile. Scale bar, 5 μm.IRepresentative confocal images of THP‐1 cells infected with RFP expressing *S. flexneri*, (MOI 1:25) and immunostained with RIPK2 and LC3B antibodies. DNA stained with DAPI. Scale bar, 8 μm. Line profile: co‐localization analysis using line intensity profile. Zoom panels are digital magnifications.J, KRepresentative confocal images of HEK293T cells transfected with (I) GFP‐RIPK2 and Myc‐ULK1 (6 h) and (J) GFP‐RIPK2 and Flag‐ATG16L (6 h). Zoom panels are digital magnification. Line profile: co‐localization analysis using line intensity profile. Scale bar, 5 μm.LWestern blot analysis with the cell lysate of PMA‐treated THP‐1 cells infected with *S. flexneri*, (MOI: 1:25) with or with Bafilomycin A1 (300 nM) for different time points as indicated.MThe qRT–PCR analysis with total RNA isolated from the uninfected and *S. flexneri* (MOI 1:2.5, 4 h) infected control or p62 knockdown or p62 and RIPK2 double knockdown THP‐1 cells. Mean ± SD, *n* = 4 (biological replicates), ***P < 0.0005 and ****P < 0.00005, ordinary one‐way ANOVA (Tukey's multiple comparisons test). Source data are available online for this figure.

We performed cycloheximide chase assays to determine the role of proteasome and/or autophagy processes in the turnover of endogenous NODs and RIPK2. The inhibition of autophagy flux using Bafilomycin A1 (Baf A1) completely protected RIPK2 and NOD1 from degradation, whereas proteasome inhibition using MG132 was partially protective (Fig EV1A and B). In the case of NOD2, only inhibition of autophagy protected it from degradation (Fig EV1C). These data indicate that autophagy plays a major role in the degradation of NODs and RIPK2. To confirm the role of autophagy in NODs and RIPK2 degradation, we monitored the levels of these proteins in ATG5 knockdown THP‐1 or ATG5 knockout MEFs cells in uninfected and *Shigella*‐infected cells. Enhanced amounts of RIPK2, NOD1, and NOD2 were detected in the ATG5‐depleted cells both in control or *Shigella*‐infected cells confirming that autophagy is critical in suppressing the levels of NODs and RIPK2 (Fig 3A and B).

Next, we tested whether RIPosomes are the target of autophagy. Treatment of cells with rapamycin dramatically reduced the number of RIPosomes (Fig 3C). This effect was completely rescued when the cells were additionally treated with Baf A1 (Fig 3C). Further, the numbers of RIPosome were significantly increased in the absence of ATG5 in *Shigella*‐infected THP‐1 cells (Fig 3D). Additionally, although RIPK2 soluble levels were induced in ATG5‐depleted cells in basal conditions, RIPosomes (insoluble oligomeric aggregates) were formed only as a result of *Shigella* infection and were further induced in ATG5 KD cells (Fig 3E). Collectively, these results demonstrate that NODs and RIPK2 proteins as well as RIPosomes are the targets of autophagy.

### SQSTM1/p62 mediates selective autophagy of ubiquitinated RIPosomes

Ubiquitin marks cargoes before the autophagy machinery recognizes them and degrades them (Shaid *et al*, 2013). The infection of *Shigella* induced the K63‐linked ubiquitination of RIPK2 *(*Fig EV1D). An evident co‐localization or juxtaposition of ubiquitin with RIPosomes was observed in HEK293T cells (Fig 3F) and *Shigella*‐infected THP‐1 cells (Fig EV1E). To understand whether ubiquitination of RIPosomes is important for their autophagic degradation, we inhibited ubiquitination in cells using PYR‐41, which is a selective and cell‐permeable inhibitor of ubiquitin‐activating enzyme E1. In high‐content microscopy, we found that rapamycin‐induced autophagic degradation of RIPosomes is blocked by PYR‐41 (Fig EV1F), indicating that ubiquitinated RIPosomes are the target of autophagy.

Autophagy receptor proteins are critical in bridging the ubiquitinated cargoes to the autophagosomes (Shaid *et al*, 2013). To identify the receptor that recognizes RIPK2, we screened the interaction between RIPK2 and key autophagy receptors, including p62, NBR1, NDP52, TAX1BP1, and Optineurin (Fig 3G). A strong physical interaction was observed between RIPK2 and p62 (Fig 3G). NBR1 was faintly bound and other receptors completely failed to interact with RIPK2 (Fig 3G). Thus, we tested whether p62 mediates autophagic degradation of RIPK2 and RIPosomes. Levels of both soluble and insoluble forms of RIPK2 were increased in *Shigella*‐infected THP‐1 cells upon p62 knockdown (Fig 3H). Also, we found an increased number of RIPosomes in p62 knockdown cells compared with control cells (Fig EV1G), suggesting that p62 plays a critical role in the autophagic degradation of RIPosomes. In agreement, p62 and LC3B (autophagosomes) were either co‐localized or juxtaposed to RIPosomes (Figs 3I and EV1H). The high‐resolution microscopy and 3D rendering of images indicate that p62 tethered the RIPosome to LC3B decorated autophagosomes (Fig 3I). Also, LC3B was found to be co‐localized with RIPosome recruited over the *Shigella* (Fig EV1I).

Next, we asked whether other key autophagosome initiations (ULK1) and elongation proteins (ATG16L1 and BECLIN1) interact and co‐localize with RIPK2/RIPosomes. We found that *in vitro* translated ULK1, BECLIN1, and ATG16L1 directly interacted with purified GST‐RIPK2 in GST pull‐down assays (Fig 3J). Further, all the three important autophagy proteins were either completely co‐localized or juxtaposed to the RIPosomes (Figs 3K–M and EV1J and K) indicating a *de novo* biogenesis of autophagosomes occurring adjacent to the RIPosomes for their degradation. When cargo is degraded via autophagy, typically autophagy receptors specifically p62 also subjected to degradation. We observed that p62 is degraded upon *Shigella* infection and this degradation is rescued upon Bafilomycin A1 treatment, suggesting that p62 is targeted by autophagy along with cargo upon *Shigella* infection (Fig EV1L).

The depletion of ATG5 and p62 significantly increased NOD1/RIPK2‐dependent NF‐κB activity in luciferase reporter assays (Fig 3N). Conversely, autophagy activation by rapamycin reduced the NOD1/RIPK2‐dependent NF‐κB activity (Fig 3O) that was rescued by Baf A1 treatment (Fig 3O). Finally, the depletion of ATG5 *or p62* significantly enhanced *Shigella*‐induced NF‐κB‐mediated pro‐inflammatory cytokine response (TNFα, IL‐1β, and IL‐1α) in THP‐1 cells (Figs 3P and EV1M). This enhanced cytokine response was rescued by RIPK2 silencing (Figs 3P and EV1M), suggesting that autophagy suppresses RIPK2‐dependent NF‐κB pro‐inflammatory cytokine response.

Altogether, we found that p62‐dependent selective autophagy mediates the degradation of NODs, RIPK2, and RIPosomes to suppress NF‐κB activation and pro‐inflammatory cytokine response (Fig 3Q).

### Autophagy protein, IRGM is recruited over the bacteria and interacts with RIPosomes

**Figure 4:**
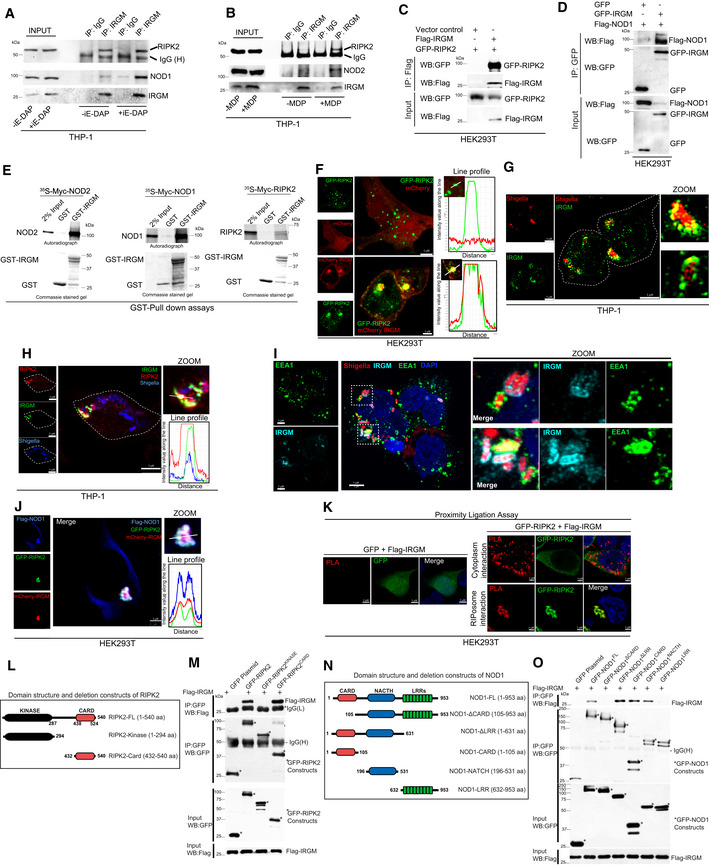
Autophagy protein IRGM interacts and co‐localizes with NODs, RIPK2, and RIPosomes A, BThe THP‐1 cell lysates were subjected to immunoprecipitation analysis (A) untreated and treated with iE‐DAP (40 μg/ml, 6 h), (B) untreated and treated with MDP (40 μg/ml, 6 h). IP was performed with isotype control IgG or IRGM antibody and Western blotting was performed with indicated antibodies. IgG (H), IgG heavy chain.C, DCo‐IP analysis of the interactions between (C) GFP‐RIPK2 and Flag‐IRGM or (D) Flag‐NOD1 and GFP‐IRGM in HEK293T cell lysates.EGST pull‐down assay using purified GST and GST‐IRGM and *in vitro* translated ^35^S radiolabeled myc‐NOD2, myc‐NOD1, and myc‐RIPK2.FRepresentative confocal images of HEK293T cells expressing GFP‐RIPK2 and mCherry or mCherry‐IRGM. Line profile: co‐localization analysis using line intensity profiles. Scale bar, 5 μm.G, HRepresentative confocal images of THP‐1 cells infected with RFP expressing *S. flexneri*, (MOI 1:25, 8 h) and immunostained with, (G) IRGM antibody (H) IRGM and RIPK2 antibody. Line profile: co‐localization analysis using line intensity profiles. Scale bar, 5 μm. Zoom panels are digital magnifications. In image (H) for better contrast, RFP expressing *Shigella* is pseudo‐colored to blue.IRepresentative confocal images of THP‐1 cells infected with RFP expressing *S. flexneri*, (MOI 1:25, 20 min) and immunostained with IRGM and EEA1 antibodies. DNA stained with DAPI. Scale bar, 5 μm. Zoom panels are digital magnifications.JRepresentative confocal images of HEK293T cells expressing GFP‐RIPK2, Flag‐NOD1, and mCherry‐IRGM. Line profile: co‐localization analysis using line intensity profiles. Scale bar, 3 μm. Zoom panels are digital magnifications.KRepresentative confocal images of proximity ligation assay (PLA) in HEK293T transfected with GFP or GFP‐RIPK2 and Flag‐IRGM plasmid. Scale bar 3 or 5 μm as indicated.LThe domain organization map of RIPK2 and deletion construct cloned as GFP‐tagged proteins.MA co‐IP analysis is performed with HEK293T cell lysates expressing various domains of RIPK2 and IRGM to map the domain/s of RIPK2 interacting with IRGM. Asterisk indicates the main band of overexpressed protein.NThe domain organization map of NOD1 and deletion construct cloned as GFP‐tagged proteins.OA co‐IP analysis is performed with HEK293T cell lysates expressing various domains of NOD1 and IRGM to map the domain/s of NOD1 interacting with IRGM. Asterisk indicates the main band of overexpressed protein. Source data are available online for this figure.

IRGM is an autophagy protein that plays a critical role in the selective autophagic degradation of pro‐inflammatory proteins (Mehto *et al*, 2019a, 2019b; Jena *et al*, 2020; Chauhan *et al*, 2021). We set out to examine the role of IRGM in the autophagic degradation of NODs, RIPK2, and RIPosomes. For that, first, we tested whether IRGM interacts with NODs and RIPK2. In the immunoprecipitation (IP) assays, endogenous IRGM interacted with RIPK2 and NODs (Fig 4A and B). The interaction of IRGM with NOD1/RIPK2 and NOD2/RIPK2 complex is further increased when the cells were treated with iE‐DAP (NOD1 agonist) or MDP (NOD2 agonist), respectively (Fig 4A and B). In IP assays with the HT‐29 cell line stably expressing Flag‐IRGM, IRGM immunoprecipitated endogenous NOD1, NOD2, and RIPK2 (Appendix Fig S3A). A strong interaction between overexpressed IRGM with NOD1, NOD2, and RIPK2 was also observed in HEK293T cells (Fig 4C and D, and Appendix Fig S3B). Furthermore, a direct interaction of purified GST‐IRGM with NODs and RIPK2 was observed in GST pull‐down assays (Fig 4E).

IRGM does not form oligomeric structures alone in the cells (Appendix Fig S3C). However, when expressed together with RIPK2, IRGM formed structures that were fully co‐localized or juxtaposed to RIPosomes (Fig 4F and Appendix Fig S3D). Analysis of data from quantitative high‐content microscopy displayed a high level of co‐localization between IRGM and RIPK2 (Appendix Fig S3E). Interestingly, in *Shigella*‐infected cells, IRGM was recruited to the intracellular bacteria (Fig 4G and Movie EV4) together with RIPosomes (Fig 4H). In some cells, we observed the formation of cage‐like structures of RIPK2 and IRGM surrounding the bacteria (Appendix Fig S3F). *Shigella* is a predominantly cytosolic bacterium that ruptures its bacteria‐containing vesicle very rapidly (within 10–15 min) after the invasion (Ray *et al*, 2010; Lopez‐Montero & Enninga, 2018). Also, *Shigella* tends to avoid the recruitment of Rab GTPases and other maturation proteins (including LAMP proteins) using several mechanisms (Ray *et al*, 2010; Lopez‐Montero & Enninga, 2018). We infected THP‐1 cells with *Shigella* for 20 min and performed immunofluorescence with EEA1 (early phagosome/endosome marker) or LAMP2A (late phagosome maturation marker) and IRGM to understand whether IRGM is recruited to phagosomal or cytosolic *Shigella*. We found that very few bacteria were positive for EEA1 protein (Fig 4I) and did not observe LAMP2A recruitment over the *Shigella* (Appendix Fig S3G) confirming the previous observations. EEA1‐marked phagosomes were rarely localized with IRGM, whereas IRGM was recruited to several of the bacteria. The data suggest that IRGM is recruited to *Shigella* once they escape phagosomes and are in the cytosolic compartment. Also, IRGM was found to be recruited on NODo‐RIPosome complexes (Fig 4J and Appendix Fig S3H). IRGM and RIPK2 interaction was confirmed by proximity ligation assay (PLA), which reports direct protein–protein interactions (Fig 4K). Direct interaction between IRGM and RIPK2 was observed in the cytosol as well as over the RIPosomes (Fig 4K).

NOD2 interacts with IRGM primarily via CARD domain (Chauhan *et al*, 2015). We mapped the domain by which RIPK2 or NOD1 interact with IRGM. RIPK2 has one kinase and one CARD domain (Fig 4L), but only the CARD domain interacted with IRGM (Fig 4M). NOD1 consists of a CARD, a NACHT, and several LRR domains (Fig 4N). The CARD and NACTH domains are utilized by NOD1 to interact with IRGM (Fig 4O, lanes 5 and 6). No interaction was detected with the LRR domain (Fig 4O, lane 7). Consistently, deleting LRR domains did not affect the NOD1‐IRGM interaction (or rather increased interaction; Fig 4O, lane 4); however, removing the CARD domain abolished NOD1‐IRGM interaction (Fig 4O, lane 3), suggesting that the CARD domain may provide a primary interface for the interaction (Fig 4O, lane 3). Thus, the findings suggest that the CARD domain of NODs and RIPK2 provides a primary interface for interaction with IRGM.

In summary, IRGM strongly and specifically interacts with NODs and RIPK2. We show that IRGM is recruited to the cytosolic *Shigella* bacteria where it co‐localizes with RIPosomes. Along with IRGM, other autophagy proteins including ULK1 and p62 were recruited to the *Shigella* bacteria (Appendix Fig S3I and J). ULK1 which is a known interaction partner of IRGM (Chauhan *et al*, 2015) was found to be completely covering the bacteria along with IRGM (Appendix Fig S3J).

### IRGM mediates NODs, RIPK2, and RIPosome degradation to suppress NF‐κB response

**Figure 5:**
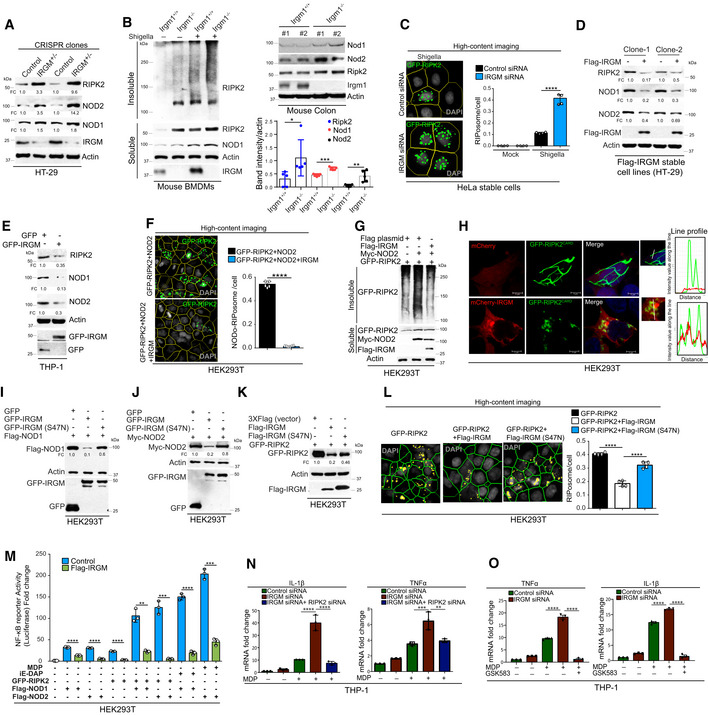
IRGM mediates the degradation of NODs, RIPK2, and RIPosomes to suppress NF‐κB‐dependent cytokine response AWestern blot analysis with the cell lysates of control CRISPR cells and CRISPR‐Cas9 mediated IRGM partial knockout (IRGM^+/−^) HT‐29 cells (2 clones were tested). Densitometric analysis was performed using Image J software. FC, fold change.BLeft panel, soluble and insoluble fractions of *S. flexneri* (MOI 1:25, 8 h) infected mouse BMDMs from *Irgm1* ^+/+^ and *Irgm1* ^−/−^ mice were subjected to immunoblot analysis with antibodies as indicated. Right panel, Western blot analysis with the colon lysates from *Irgm1* ^+/+^ and *Irgm1* ^−/−^ mice with indicated antibodies. The graph indicate ratio of band intensity (measured using ImageJ) and actin (*n* = 5, Mean ± SD, *P < 0.05, **P < 0.005, and ****P < 0.00005, Student's unpaired *t*‐test).CDoxycycline‐inducible stable GFP‐RIPK2 HeLa cells were transfected with control siRNA or IRGM siRNA followed by infection with *S. flexneri* (MOI 1:25, 4 h). The cells were fixed and subjected to high‐content microscopy to quantitate the number of RIPosomes formed. The graph depicts an average number of RIPosome/cell. About 10,000 cells were plated per well and RIPosomes were screened in 35 fields per well. Mean ± SD, *n* = 4 (biological replicates), ****P < 0.00005, Student's unpaired *t*‐test.D, EWestern blot analysis with the cell lysate of (D) HT‐29 clones stably expressing Flag‐vector control or Flag‐IRGM (E) THP‐1 cells transiently transfected with GFP or GFP‐IRGM for 6 h. Densitometric analysis was performed using Image J software. FC, fold change.FLeft panel, representative high‐content microscopy images of NODo‐RIPosomes in HEK293T cells transfected with GFP‐RIPK2 and myc‐NOD2 (upper panel) or GFP‐RIPK2, myc‐NOD2, and Flag‐IRGM (lower panel). Right panel, the graph depicts average number of NODo‐RIPosome/cell. About 15,000 cells plated per well and RIPosomes were screened in 35 fields per well. Mean ± SD, *n* = 5 (biological replicates), ****P < 0.00005, Student's unpaired *t*‐test.GThe soluble and insoluble fractions of HEK293T cells transfected with indicated plasmids were subjected to immunoblot analysis with indicated antibodies.HRepresentative confocal images of HEK293T cells transfected with mCherry and GFP‐RIPK2^CARD^ or mCherry‐IRGM and GFP‐RIPK2^CARD^ for 9 h. Line profile: co‐localization analysis using line intensity profiles. Scale bar, 5 μm.I–KWestern blot analysis with the cell lysates of HEK293T transfected with indicated plasmids and probe with Actin, Flag, Myc, and, GFP antibodies as indicated. Densitometric analysis was performed using Image J software. FC, fold change.LLeft panels, representative high‐content microscopy images of HEK293T cells transfected with GFP‐RIPK2 or GFP‐RIPK2 and Flag‐IRGM or GFP‐RIPK2 and Flag‐IRGM (S47N). The graph depicts the average number of RIPosome/cell. The details are mentioned in the legends of Fig 1F. Mean ± SD, *n* = 4 (biological replicates), ****P < 0.00005, ordinary one‐way ANOVA (Tukey's multiple comparisons test).MLuciferase assays performed with HEK293T cells transfected with NF‐κB luciferase reporter vector pGL4.32NFκB‐RE, along with plasmids as indicated, followed by treatment with MDP (10 μg/ml, 4 h) or iE‐DAP (10 μg/ml, 4 h) as indicated. Mean ± SD, *n* = 3 (biological replicates), **P < 0.005, ***P < 0.0005 and ****P < 0.00005, Student's unpaired *t*‐test.N, OThe qRT–PCR analysis with total RNA isolated from THP1 cells transfected with indicated siRNA and treated with (N) L‐18 MDP (1 μg/ml, 6 h) for indicated genes (O) L‐18 MDP (1 μg/ml, 6 h) and GSK583 (1 μM). Mean ± SD, *n* = 3 (biological replicates), *P < 0.05, **P < 0.005, ***P < 0.0005 and, ****P < 0.00005, ordinary one‐way ANOVA (Tukey's multiple comparisons test). Source data are available online for this figure.

**Figure EV2:**
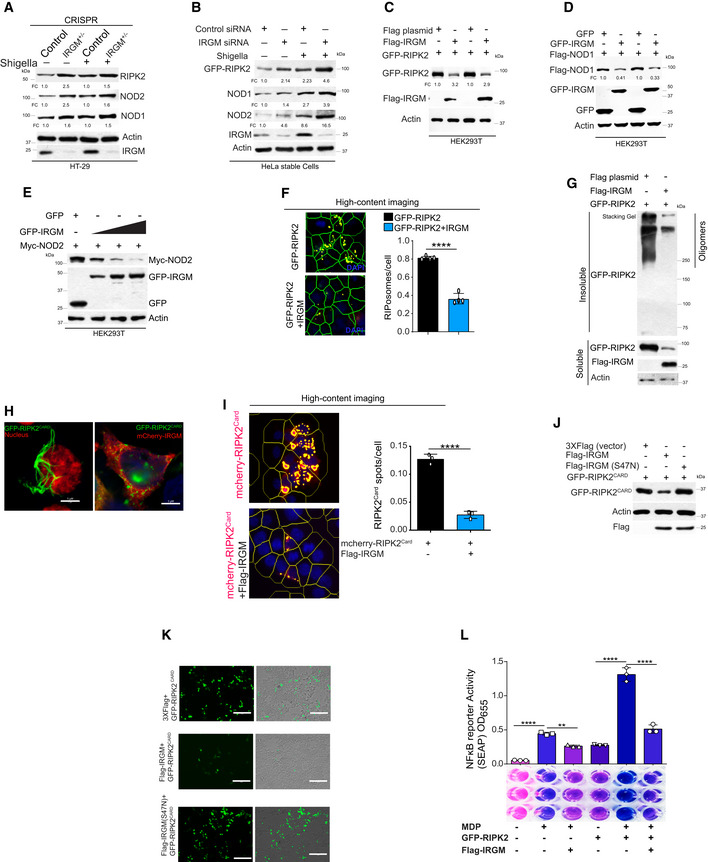
IRGM mediates the degradation of NODs, RIPK2, and RIPosomes AThe cell lysates of *S. flexneri*‐infected IRGM^+/+^ and IRGM^+/−^ HT‐29 cells were subjected to Western blot analysis with indicated antibodies. Densitometric analysis was performed using Image J software. FC, fold change.BWestern blot analysis with the cell lysates of control and IRGM siRNA transfected doxycycline (1 μg/ml) induced HeLa GFP‐RIPK2 stable cells infected with S. *flexneri* (MOI 1:25, 8 h). Densitometric analysis was performed using Image J software. FC, fold change.C, DWestern blot analysis with the cell lysates of HEK293T transfected with indicated plasmids. Densitometric analysis was performed using Image J software. FC, fold change.EWestern blot analysis with the cell lysate of HEK293T cells transfected Myc‐NOD2 and GFP or increasing concentration of GFP‐IRGM (2, 4, and 6 μg) plasmids for 12 h.FLeft panels, representative high‐content microscopy images (Green masks, software algorithms‐defined cell boundaries, digitally zoomed images) of HEK293T cells transfected with GFP‐RIPK2 or GFP‐RIPK2 and Flag‐IRGM. About 17,000 cells were plated per well in a 96‐well plate and RIPosomes were screened in 35 fields per well. Right panel, the graph depicts the average number of RIPosomes/cell, which is calculated from four biological replicates, Mean ± SD. ****P < 0.00005, Student's unpaired *t*‐test.GWestern blot analysis of soluble and insoluble fractions of HEK293T cells transfected with indicated plasmids for 12 h.HRepresentative confocal image of HEK293T cells transfected with GFP‐RIPK2^CARD^ or GFP‐RIPK2^CARD^ and mCherry‐IRGM. Scale bar, 5 μm.IRight panels, representative high‐content microscopy images (Yellow masks, software algorithms‐defined cell boundaries, digitally zoomed images) of HEK293T cells transfected with mCherry‐RIPK2^CARD.^ Alone or with Flag‐IRGM. About 17,000 cells were plated per well in a 96‐well plate and RIPK2^CARD^ spots were screened in 35 fields per well. Left panel, the graph depicts an average number of RIPK2^CARD^ spots/cell, which is calculated from three biological replicates, Mean ± SD. ****P < 0.00005, Student's unpaired *t*‐test.JWestern blot analysis with the cell lysates of HEK293T transfected with indicated plasmids for 12 h.KRepresentative fluorescence microscopy images of HEK293T cells transfected with GFP‐RIPK2 ^CARD^ alone or with Flag‐IRGM or with catalytic mutant Flag‐IRGM (S47N) for 12 h. Scale bar, 150 μm.LAssessment of NF‐κB‐induced SEAP (secreted embryonic alkaline phosphatase) activity in the cell culture supernatant of HEK‐Blue™ hNOD2 cells (InvivoGen) transfected with plasmids as indicated and treated with L18‐MDP (100 ng/ml, 24 h) as indicated. Three technical replicates, Mean ± SD. **P < 0.005, ****P < 0.00005, Student's unpaired *t*‐test. Source data are available online for this figure.

Next, we investigated how IRGM interaction with NODs, RIPK2, and RIPosomes modulates their functions. An increased protein level of RIPK2 and NODs was observed in IRGM CRISPR (partial) knockout HT‐29 (IRGM^+/−^) cells compared with the control cells (Fig 5A). The IRGM^+/−^ HT29 cells are described previously (Jena *et al*, 2020). Also, *Shigella*‐induced expression of NODs and RIPK2 was further enhanced in IRGM^+/−^ HT‐29 cells (Fig EV2A). Increased protein levels of NODs and RIPK2 were detected in colons and BMDMs of *Irgm1* knockout (*Irgm1* ^−/−^) mice (Fig 5B). An increased amount of insoluble oligomeric RIPK2 was observed in Irgm1^−/−^ BMDMs lysates as compared to the control cells (Fig 5B). Next, we used a HeLa cell line expressing stable doxycycline‐inducible human GFP‐RIPK2 (Ellwanger *et al*, 2019). Silencing IRGM in the *Shigella*‐infected cells enhanced the levels of NODs and RIPK2 (Fig EV2B), and a significantly increased number of RIPosomes was observed (Fig 5C). Conversely, transient and stable overexpression of IRGM in HT‐29, THP‐1, and HEK293T cells resulted in degradation of endogenous and overexpressed NODs and RIPK2 (Figs 5D and E, and EV2C–E). Overexpression of IRGM significantly reduced the RIPosomes and NODo‐RIPosomes formed in the cells (Figs 5F and EV2F). Also, the expression of IRGM reduced the insoluble oligomeric forms of RIPK2 and NOD2/RIPK2 (Figs 5G and EV2G). IRGM was found to be co‐localized with RIPK2^CARD^ and was able to degrade the RIPK2^CARD^ filamentous structure into small punctate assemblies (Figs 5H, and EV2H and I). Taken together, the data show that IRGM mediates the degradation of NODs, RIPK2, and RIPosomes.

A point mutation in the GTPase domain (Serine to Glycine at 47^th^ position, S47N) of IRGM renders it inactive to perform autophagy functions (Chauhan *et al*, 2015; Kumar *et al*, 2018; Mehto *et al*, 2019a; Jena *et al*, 2020). As compared to wild‐type IRGM, the catalytic mutant (S47N) of IRGM was unable to mediate the degradation of NOD1 (Fig 5I), NOD2 (Fig 5J), RIPK2 (Fig 5K), RIPK2^CARD^ (Fig EV2J), and their oligomers (Figs 5L and EV2K). These results demonstrate the specificity of IRGM‐mediated effects and also indicate the role of GTPase‐dependent autophagy activity of IRGM in the degradation of NODs, RIPK2, and their oligomers.

Next, we examined the effect of IRGM on NODs/RIPK2‐dependent NF‐κB activation. First, we chased the phosphorylation (Ser536) status of p65 (p‐p65) in the presence and absence of IRGM. The p‐p65 is induced upon *Shigella* infection that is further increased upon IRGM knockdown (Appendix Fig S4A). The data suggest that *Shigella*‐induced IRGM expression suppresses the NF‐κB activation. In luciferase NF‐κB reporter assays, IRGM strongly suppressed basal and NODs agonists (MDP and iE‐DAP) induced NF‐κB promoter activity (Fig 5M). We also employed an MDP‐inducible NOD2 expressing secreted alkaline phosphatase (SEAP)‐based NF‐κB reporter cell line (Invivogen) to assess the effect of IRGM on NF‐κB response. The overexpression of IRGM diminished MDP‐induced NOD2 and NOD2‐RIPK2 dependent NF‐κB reporter activity (Fig EV2L). By contrast, silencing IRGM in MDP‐treated THP‐1 cells resulted in increased mRNA expression of IL‐1β and TNFα (Fig 5N), which was restored in the RIPK2‐depleted cells (Fig 5N). Similarly, GSK583, a specific and potent inhibitor of RIPK2 (Haile *et al*, 2016), suppressed the cytokine response increased upon IRGM knockdown (Fig 5O). Taken together, our results show that IRGM facilitates the degradation of NODs, RIPK2, and their oligomers to suppress NF‐κB activity and cytokine response.

Endogenous IRGM levels in cells were increased upon exposure to *Shigella*, (Figs 5B, and EV2A and B; compare lanes 1 and 3), MDP (Appendix Fig S4B and D), and iE‐DAP (Appendix Fig S4C and E). Thus, microbes and NODs agonists induce expression of IRGM that by a negative feedback loop mediates degradation of NODs‐RIPK2 signaling proteins to suppress NF‐κB response to maintain cell‐autonomous innate immune homeostasis.

### IRGM and p62 cooperate to conduct selective autophagy of NODs and RIPK2/RIPosomes

**Figure EV3:**
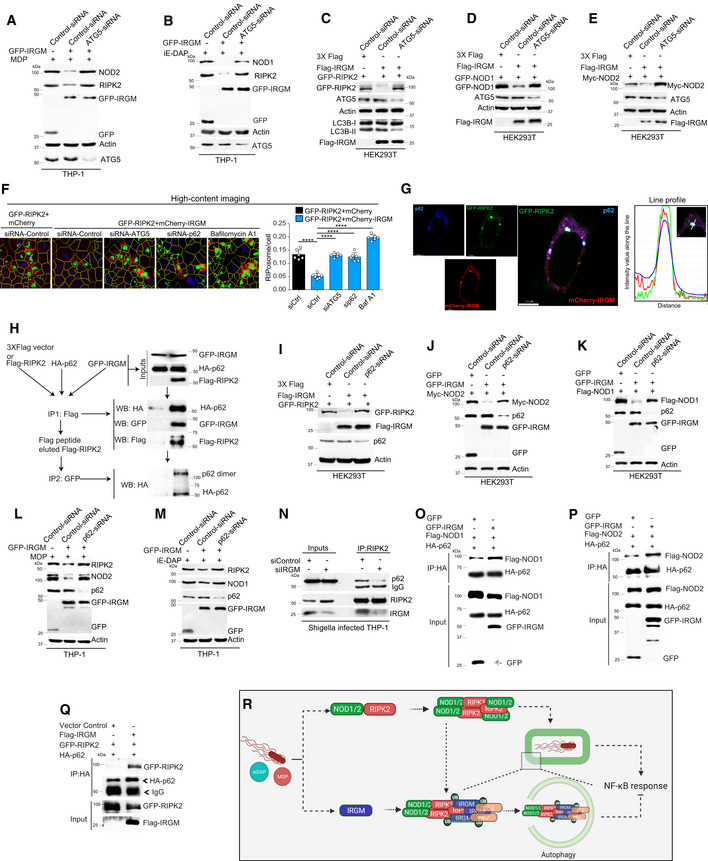
IRGM and p62 cooperatively execute selective autophagy of NODs, RIPK2, and RIPosomes A, BWestern blot analysis with cell lysates of control cells and ATG5 knockdown THP‐1 cells transiently transfected with GFP‐IRGM plasmids and treated with (A) MDP (40 μg/ml, 4 h) or (B) iE‐DAP (40 μg/ml, 4 h).C–EThe control and ATG5 knockdown HEK293T cells were transfected with indicated plasmids and cell lysates were subjected to immunoblot analysis.FLeft panels, representative high‐content microscopy images (digitally zoomed) of cells that were knockdown for indicated genes and transfected with plasmids as indicated. In the last panel, GFP‐RIPK2 and mcherry‐IRGM transfected cells were treated with Bafilomycin A1 (300 nM, 5 h). Right panel, the graph depicts an average number of RIPosomes/cell. About 50,000 cells were plated per well and RIPosomes were screened in 35 fields per well. Mean ± SD, *n* = 6 (biological replicates), ****P < 0.00005, ordinary one‐way ANOVA (Tukey's multiple comparisons test).GRepresentative confocal images of cells transfected with GFP‐RIPK2, and mCherry‐IRGM and immunostained with p62. Line profile: co‐localization analysis using line intensity profiles. Scale bar, 5.5 μm.HSequential immunoprecipitation assay from the lysate where the HEK293T transiently transfected with Flag‐RIPK2 (or Flag‐vector control), HA‐p62, and, GFP‐IRGM for 12 h. The first immunoprecipitation was performed with Flag antibody followed by elution with flag peptide. The flag peptide eluted samples were further subjected to a second IP with anti‐GFP (for IRGM) and probed with indicated antibodies.I–KThe control and p62 knockdown HEK293T cells were transfected with indicated plasmids and cell lysates were subjected to Western blot analysis with indicated antibodies.L, MWestern blot analysis with cell lysates of control cells and p62 knockdown THP‐1 cells transiently transfected with GFP‐IRGM plasmids treated with (L) MDP (40 μg/ml, 4 h) or (M) iE‐DAP (40 μg/ml, 4 h).NImmunoprecipitation analysis of the interaction between endogenous RIPK2 and endogenous p62 in the lysate of *S. flexneri* (MOI 1:25, 6 h) infected control and IRGM knockdown THP‐1 cells. IgG, IgG heavy chain.O–QCo‐IP analysis with the lysate of HEK293T cells transiently transfected with (O) Flag‐NOD1 and HA‐p62, (P) Flag‐NOD2 and HA‐p62, and, (Q) GFP‐RIPK2 and HA‐p62 in the presence and absence of IRGM (GFP or Flag) or vector controls.RPictorial representation of data. We found that IRGM and p62 coordinate selective autophagy of NODs, RIPK2, and RIPosomes. Source data are available online for this figure.

We scrutinized whether IRGM‐dependent degradation of NODs and RIPK2 is mediated through proteasome or autophagy. Inhibition of autophagy flux (using Baf A1) but not proteasome (using MG132) restored the IRGM‐dependent degradation of NODs and RIPK2 (Appendix Fig S5A–C). Further, the depletion of ATG5 rescued the IRGM‐mediated degradation of endogenous (THP‐1 cells; Fig EV3A and B) or overexpressed (HEK293T cells) NODs and RIPK2 (Fig EV3C–E). Also, the IRGM‐dependent RIPosome degradation is restored when the cells were either depleted of ATG5 or were treated with Baf A1 (Fig EV3F). This and the above‐discussed catalytic mutant (IRGM^S47N^) data demonstrate that IRGM utilizes autophagy to degrade NODs, RIPK2, and RIPosomes. Further, PYR‐41 (inhibitor of ubiquitin‐activating enzyme E1) rescued the IRGM‐mediated degradation of RIPK2, indicating that ubiquitinated RIPK2 is targeted by IRGM‐dependent autophagy (Appendix Fig S5D).

Like RIPK2, IRGM interacts only with p62 among the autophagy receptor proteins (Jena *et al*, 2020). Therefore, we tested whether IRGM cooperates with p62 for the autophagic degradation of RIPK2 and RIPosomes. First, we investigated whether IRGM, p62, and RIPK2 are present in the same molecular complex. IRGM and p62 were co‐localized or juxtaposed to the RIPosomes (also NODo‐RIPosomes; Fig EV3G and Appendix Fig S5E and F). Also, co‐localization/juxtaposition of RIPosomes, IRGM, and p62 with LC3B was observed (Appendix Fig S5G). Using sequential IP assay, we found that RIPK2, p62, and IRGM were present in the same complex (Fig EV3H). Silencing of p62 rescued the IRGM‐dependent autophagic degradation of RIPosomes (Fig EV3I), and also IRGM‐mediated degradation of overexpressed as well as endogenous RIPK2, NOD1, and NOD2 (Fig EV3J–M). Thus, IRGM utilizes p62 adaptor protein to mediate autophagic degradation of NODs, RIPK2, and RIPosomes. Next, we tested whether IRGM is required for the interaction between p62 and RIPK2. Indeed, the depletion of IRGM reduced the interaction between p62 and RIPK2 (Fig EV3N). Conversely, the presence of IRGM increased the interaction of p62 with NODs and RIPK2 (Fig EV3O–Q).

Taken together, the results show that IRGM and p62 cooperate to conduct inflammophagy of NODs, RIPK2, and RIPosome complexes. In addition, we observed that autophagy initiation and elongation protein ULK1 and ATG16L1 were co‐localized with IRGM over the RIPosomes (Appendix Fig S5H and I), indicating that IRGM engages canonical autophagy machinery for degradation.

Combined results from this and the previous sections suggest microbes induce RIPosome formation and IRGM expression. Both IRGMs and RIPosomes are recruited over bacteria. Where RIPosome formation induces NF‐κB response, IRGM‐dependent autophagic degradation of NODs‐RIPK2 complex suppresses the NF‐κB response to balance the inflammatory outputs (Fig EV3R).

### IRGM negatively regulates bacteria‐induced RIPK2‐dependent pro‐inflammatory signaling pathways

**Figure EV4:**
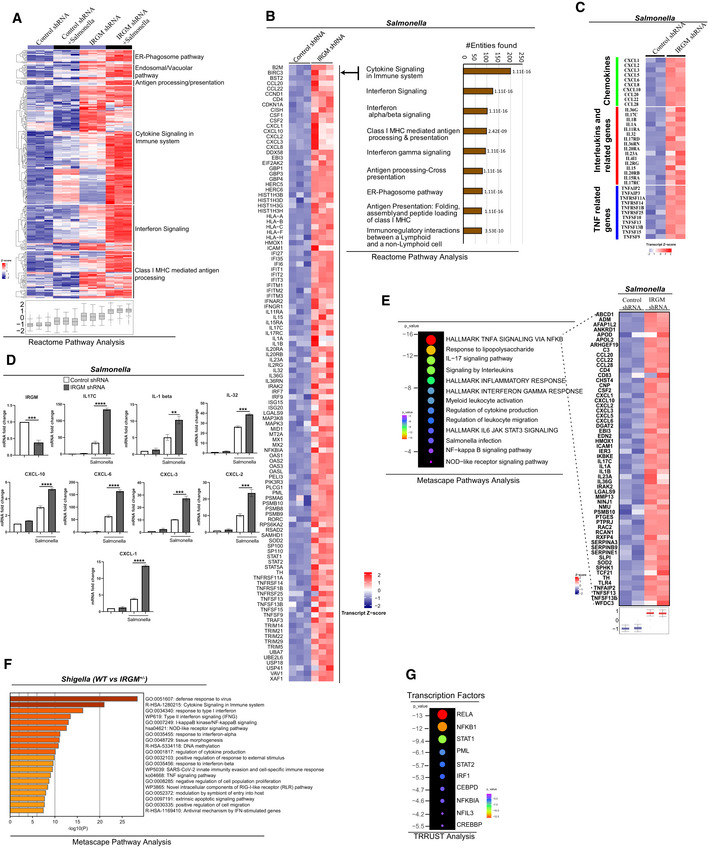
IRGM negatively regulates bacterial infection‐induced programs of pro‐inflammatory response Heatmap of the gene clusters related to top biological pathways overrepresented in the Reactome pathway analysis. Reactome pathway analysis was performed with the set of differentially regulated genes (1.5‐fold, *P* < 0.05, Wald Chi‐Squared test, *n* = 3, biological replicates) in uninfected and *Salmonella*‐infected control and IRGM shRNA knockdown HT‐29 cells. The graph below the heat map shows box plot with median obtained from data scaled to Z‐score.Left panel, heatmap depicts the gene set belonging to the “Cytokine signaling in immune system” pathway that is one of the top upregulated pathways in Reactome pathway analysis. Reactome pathway analysis was performed with the set of genes significantly upregulated (1.5‐fold, *P* < 0.05, Wald test, *n* = 3) in *Salmonella typhimurium* infected IRGM shRNA knockdown HT‐29 cells compared with control shRNA cells. Right panel, the bar graph represents the top 10 biological pathways upregulated in GO‐based Reactome pathways analysis using a set of genes that are significantly (1.5‐fold, *P* < 0.05, Wald Chi‐Squared test, *n* = 3 in each group) induced in *Salmonella*‐infected IRGM shRNA HT‐29 as compared to *Salmonella*‐infected control cells.Heatmap depicts the significantly upregulated (1.5‐fold, *P* < 0.05, Wald Chi‐Squared test, *n* = 3) NF‐κB regulated cytokine and chemokine genes in *Salmonella*‐infected IRGM shRNA HT‐29 cells compared with *Salmonella*‐infected control shRNA cells.A qRT–PCR validation of significantly upregulated cytokines and chemokines in RNA‐Seq data with total RNA isolated from uninfected and *Salmonella*‐infected control and IRGM shRNA HT‐29 cells. Mean ± SD, *n* = 3 (Biological replicates). **P < 0.05, ***P < 0.005, ****P < 0.0005, Student's unpaired *t*‐test.Left panel, Metascape pathway analysis with the set of genes that are significantly upregulated in IRGM^+/−^ cells (compared with control; 1.5‐fold, *P* < 0.05, Wald Chi‐Squared test, *n* = 3) and at the same time were significantly rescued by RIPK2 depletion in IRGM^+/−^ HT‐29 cells (*P* < 0.05, Wald Chi‐Squared test, 0.8, *n* = 3). Right panel, heatmap depicts the gene set belonging to “Hallmark TNFα signaling via NF‐κB” pathway that is the top upregulated pathway in Metascape analysis. The graph below the heat map shows box plot with median obtained from data scaled to Z‐score.Bar graph depicts top biological pathway upregulated in GO‐based metascape pathway analysis with genes significantly induced (1.5‐fold, *P* 0.05, *n* = 3) in *Shigella*‐infected IRGM^+/−^ HT‐29 cells.TRRUST analysis (database for the study of the transcriptional regulation involved in human diseases) with the set of genes that are significantly upregulated in IRGM^+/−^ cells (compared with control; 1.5‐fold, *P* < 0.05, Wald Chi‐Squared test, *n* = 3, biological replicates) and at the same time were significantly rescued by RIPK2 depletion in IRGM^+/−^ HT‐29 cells (*P* < 0.05, Wald Chi‐Squared test, 0.8, *n* = 3). Source data are available online for this figure.

To understand the role of IRGM in regulating host response to bacterial infection, we performed RNA‐sequencing (RNA‐seq) experiment with *Salmonella typhimurium* infected control and stable IRGM shRNA knockdown HT‐29 colon cells. Hierarchical clustering based on gene ontology (GO) terms was performed using genes differentially regulated (*P* < 0.05, 1.5‐fold) in basal and *Salmonella*‐infected IRGM‐depleted cells compared with controls. Several inflammatory (e.g., Interferon signaling and cytokine signaling) and infection‐related processes (e.g. ER‐phagosome and antigen processing/presentation) were among the top‐enriched pathways (Reactome pathway analysis) induced in IRGM‐depleted cells, which were further increased upon *Salmonella* infection (Datasets EV1 and EV2, and Fig EV4A). In basal conditions, IRGM suppresses a large number of IFN‐stimulated genes (ISGs; Jena *et al*, 2020). Here, we found that during *Salmonella* infection, in addition to ISGs, IRGM suppressed a large number of chemokines (*CXCL1*, *2*, *3*, *5*, *6*, *8*, *10* and *CCL20*, *22*, *28*, etc.) interleukins (*IL1A*, *IL1B*, *IL1E/36G*, *IL17C*, *IL32*, *IL15*, etc.) and TNF superfamily genes (*TNFSF9*, *TNFSF10*, *TNFSF13*, *TNFSF15*, etc.; Dataset EV3, and Fig EV4B and C). Several other pathways such as endoplasmic reticulum‐phagosome response, endosomal/vacuolar pathways, and antigen processing and presentation were upregulated in *Salmonella*‐infected IRGM‐depleted cells (Fig EV4A and B). The RNA‐seq results were validated by performing qRT–PCR with several chemokines and interleukins (Fig EV4D). Next, to define the IRGM‐dependent transcriptome that is exclusively upregulated upon *Salmonella* infection, we filtered out all the basal level deferentially upregulated genes and performed Reactome pathway analysis (Fabregat *et al*, 2018) with the rest of the transcriptome (Dataset EV4). In this analysis, the top‐enriched pathways were TNFα signaling, IL‐17 signaling, NF‐κB signaling, interleukin‐dependent signaling, interferon‐gamma response, and NOD‐like receptor signaling pathway (Fig EV4E). These data indicate that during bacterial infection IRGM limits an extensive and comprehensive program of pro‐inflammatory response including NF‐κB, TNFα, NODs, and IFN signaling pathways.

**Figure 6:**
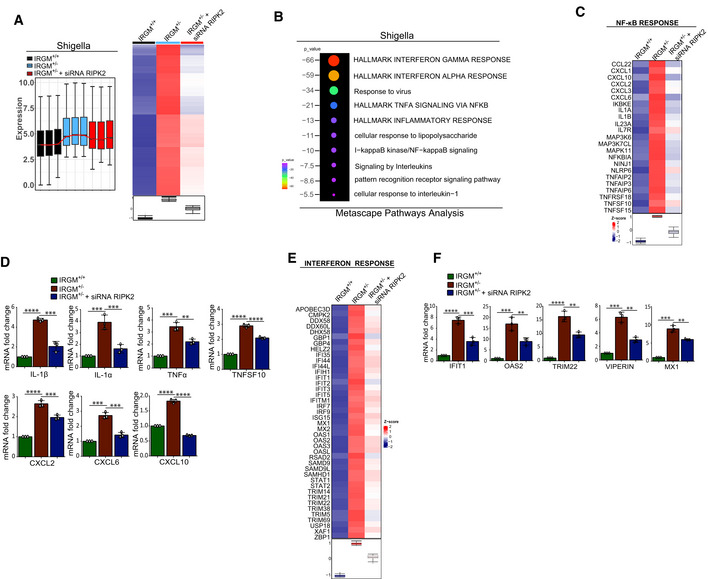
IRGM negatively regulates *Shigella*‐induced RIPK2‐dependent pro‐inflammatory signaling pathways Box plot distribution with normalized log expression (Left panel) and heatmap (Right panel) of the genes that were significantly upregulated in IRGM^+/−^ HT‐29 cells (compared with control; 1.5‐fold, *P* < 0.05, Wald Chi‐Squared test, *n* = 3, biological replicates) and at the same time were significantly rescued by RIPK2 depletion in IRGM^+/−^ HT‐29 cells (*P* < 0.05, 0.8, *n* = 3) infected with *S. flexneri* for 6 h (MOI 1:25). Left panel, the box and whisker plot shows upper and lower quartile of the datasets. Right panel, the graph below the heat map shows box plot with median obtained from data scaled to Z‐score.Bubble plot graph depicting the overrepresented pathways (in order of *P*‐value) obtained using Metascape pathway analysis software from the genes significantly upregulated in IRGM^+/−^ cells (compared with control; 1.5‐fold, *P* < 0.05, Wald Chi‐Squared test, *n* = 3) and at the same time were significantly rescued by RIPK2 depletion in IRGM^+/−^ HT‐29 cells (*P* < 0.05, 0.8, *n* = 3) infected with *S. flexneri* for 6 h (MOI 1:25).Heatmap depicting the representative NF‐κB responsive genes that were significantly upregulated in IRGM^+/−^ cells (compared with control; 1.5‐fold, *P* < 0.05, Wald Chi‐Squared test, *n* = 3 biological replicates) and at the same time were significantly rescued by RIPK2 depletion in IRGM^+/−^ HT‐29 cells (*P* < 0.05, Wald Chi‐Squared test, 0.8, *n* = 3 biological replicates). The graph below the heat map shows box plot with median obtained from data scaled to Z‐score.The qRT–PCR analysis of NF‐κB responsive cytokines and chemokines with total RNA isolated from the *S. flexneri*‐infected IRGM^+/+^, IRGM^+/−^, and RIPK2‐depleted IRGM^+/−^ HT‐29 cells. Mean ± SD, *n* = 3 (biological replicates), **P < 0.005, ***P < 0.0005 and ****P < 0.00005, ordinary one‐way ANOVA (Tukey's multiple comparisons test).Heatmap depicting the representative IFN response genes significantly upregulated in IRGM^+/−^ cells (compared with control; 1.5‐fold, *P* < 0.05, Wald Chi‐Squared test, *n* = 3 biological replicates) and at the same time were significantly rescued by RIPK2 depletion in IRGM^+/−^ HT‐29 cells (*P* < 0.05, Wald Chi‐Squared test, 0.8, *n* = 3, biological replicates). The graph below the heat map shows box plot with median obtained from data scaled to Z‐score.The qRT–PCR analysis of NF‐κB responsive cytokines and chemokines with total RNA isolated from the *S. flexneri*‐infected IRGM^+/+^, IRGM^+/−^, and RIPK2‐depleted IRGM^+/−^ HT‐29 cells. Mean ± SD, *n* = 3 (biological replicates), **P < 0.005, ***P < 0.0005 and ****P < 0.00005, ordinary one‐way ANOVA (Tukey's multiple comparisons test). Source data are available online for this figure.

Our next step was to evaluate the extent to which IRGM‐mediated suppression of the inflammatory response was dependent upon NOD1/2‐RIPK2 signaling. For that, we performed RNA‐seq analysis with *Shigella flexineri* infected WT, IRGM^+/−^, and RIPK2‐depleted IRGM^+/−^ HT‐29 cells. To better understand whether the suppression of the pro‐inflammatory response by IRGM is specific to *Salmonella* or applies globally, we performed transcriptome analysis on *Shigella*‐infected cells. A comparison of the analysis of the transcriptome induced in *Shigella* versus *Salmonella*‐infected IRGM‐depleted cells suggests that almost similar genes and pathways were upregulated in both the conditions (Fig EV4B vs. F, Dataset EV5), suggesting that IRGM has identical anti‐inflammatory functions during different bacterial infections. Next, we performed the analysis with a set of genes that are significantly upregulated in IRGM^+/−^ HT‐29 cells (compared with control, *P* < 0.05, 1.5‐fold, *n* = 3) and at the same time were significantly rescued by RIPK2 depletion (*P* < 0.05, 0.8, *n* = 3; Dataset EV6). A large number of genes (~250 genes) that were induced upon IRGM depletion were rescued by RIPK2 knockdown, suggesting that IRGM regulates pro‐inflammatory responses through modulation of RIPK2 protein levels (Fig 6A and Dataset EV6). Metascape pathway analysis (Tripathi *et al*, 2015) with this gene set revealed that upon *Shigella* infection, IRGM suppresses several inflammatory responses including IFN response, NF‐κB signaling, and interleukin signaling in a RIPK2‐dependent manner (Fig 6B). Several of the NF‐κB‐regulated genes such us *NINJ1*, *MAPK11*, and cytokines such as CXCLs (*CXCL1*, *3*, *6*, *10*, *and 11*), Interleukins (*IL1α and IL1β*), tumor necrosis factors, and receptors (*TNF‐α*, *TNSFAIP's*, *and TNSF's*), that were induced upon IRGM^+/−^ were rescued by additional siRNA knockdown of RIPK2 (Fig 6C and D). Interestingly, during bacterial infection IRGM limited the interferon response in a RIPK2‐dependent manner (Fig 6E and F). This was evident by the partial rescue of several sentinels' interferon‐responsive genes including *IFITM's*, *GBP's*, *OAS1‐3*, *MX1/2*, *ISG15*, and *RSAD2* when RIPK2 is knockdown in IRGM^+/−^ HT‐29 cells (Fig 6E and F). In agreement with these results, TRRUST (database of literature‐curated human TF‐target interactions; Han *et al*, 2018) analysis predicted RELA, NF‐κB, and STAT1 as the major transcription factors for this response (Fig EV4G).

Taken together, these results demonstrate that IRGM suppresses multiple inflammatory responses during bacterial infection and limits the array of RIPK2‐dependent pro‐inflammatory responses.

### RIPK2 inhibition ameliorates shigellosis and DSS‐induced gut inflammation in *Irgm1* ^*KO*^ mice

Genetic variations in *IRGM* are associated with increased susceptibility to sepsis, bacterial infections, and gut inflammatory diseases (Massey & Parkes, 2007; Parkes *et al*, 2007; Intemann *et al*, 2009; Kimura *et al*, 2014). Consistently, *Irgm1* ^−/−^ mice were found to be more susceptible to DSS‐induced colitis (Liu *et al*, 2013). Since we found that IRGM negatively regulates RIPK2‐dependent pro‐inflammatory responses, we hypothesized that therapeutic inhibition of RIPK2 could reduce gut inflammation associated with *Irgm1* depletion in shigellosis‐ and DSS‐induced colitis models.

**Figure 7:**
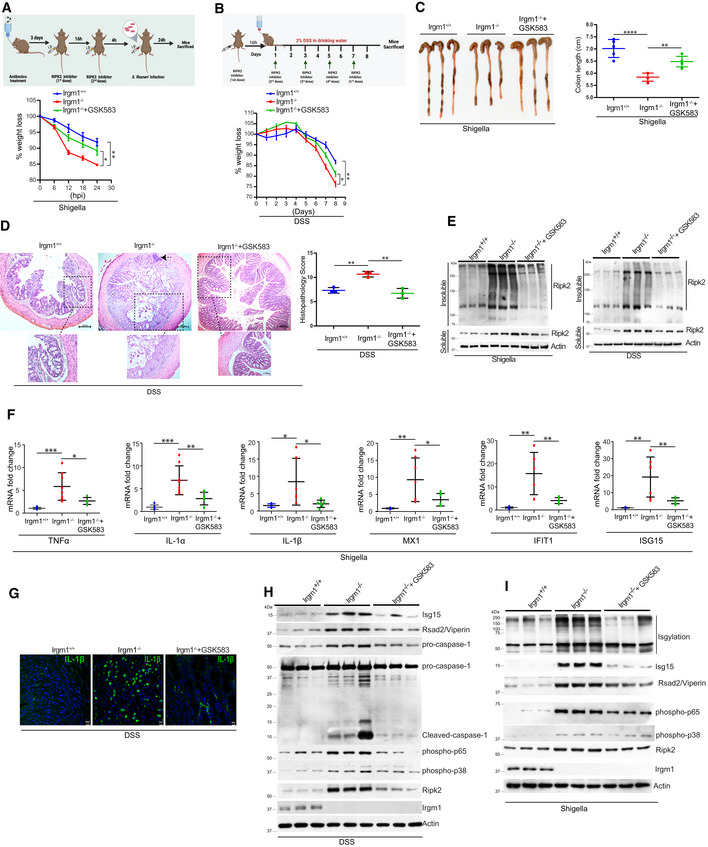
RIPK2 inhibition ameliorates shigellosis‐ and DSS‐induced gut inflammation in *Irgm1* knockout mice A, BUpper panels, schematic representation of shigellosis‐ and DSS‐induced colitis models used. In lower panels, the graph depicts percentage loss in body weight in *Irgm1* ^+/+^ and GSK583 untreated or treated *Irgm* ^−/−^ mice when (A) infected with *S. flexneri* (*n* = 6 mice) or (B) administrated with DSS (*n* = 3 mice). Mean ± SD, *P < 0.05, **P < 0.005, Student's unpaired *t*‐test.CLeft panel, representative pictures of colons of *Irgm1* ^+/+^ and *Irgm* ^−/−^ mice untreated or treated with GSK583 infected with *S. flexneri*. Right panel, the graph depicts the average colon lengths of the mice groups. Mean ± SD, *n* = 6, **P < 0.005, ***P < 0.0005, Student's unpaired *t*‐test.DRepresentative microscopic images of H&E staining of colon tissues of *Irgm1* ^+/+^ and *Irgm* ^−/−^ mice untreated or treated with GSK583 administrated with DSS. The graph depicts the combined histological scores. Mean ± SD, *n* = 3 (DSS), **P < 0.005, Student's unpaired *t*‐test. Scale bar, 200 μm.EThe soluble and insoluble fractionations of lysates from colon tissues of *Shigella*‐infected or DSS‐treated *Irgm1* ^+/+^ and *Irgm* ^−/−^ mice treated with GSK583 as indicated, were subjected to immunoblot analysis with indicated antibodies.FThe qRT–PCR analysis with total RNA isolated from the colon tissues of *S. flexneri*‐infected Irgm1^+/+^ or Irgm1^−/−^ or GSK583‐treated Irgm1^−/−^ mice. Mean ± SD, *n* = 6–8, *P < 0.05, **P < 0.005, ***P < 0.0005, ordinary one‐way ANOVA (Tukey's multiple comparisons test).GRepresentative confocal images of IL‐1β immunostained colon tissues of DSS‐treated *Irgm1* ^+/+^ or *Irgm1* ^−/−^ or GSK583‐treated *Irgm1* ^−/−^ mice. Scale bar, 10 μm.H, IWestern blot analysis with the colon tissue lysates of DSS‐treated or *S. flexneri*‐infected mice groups as indicated. Source data are available online for this figure.

**Figure EV5:**
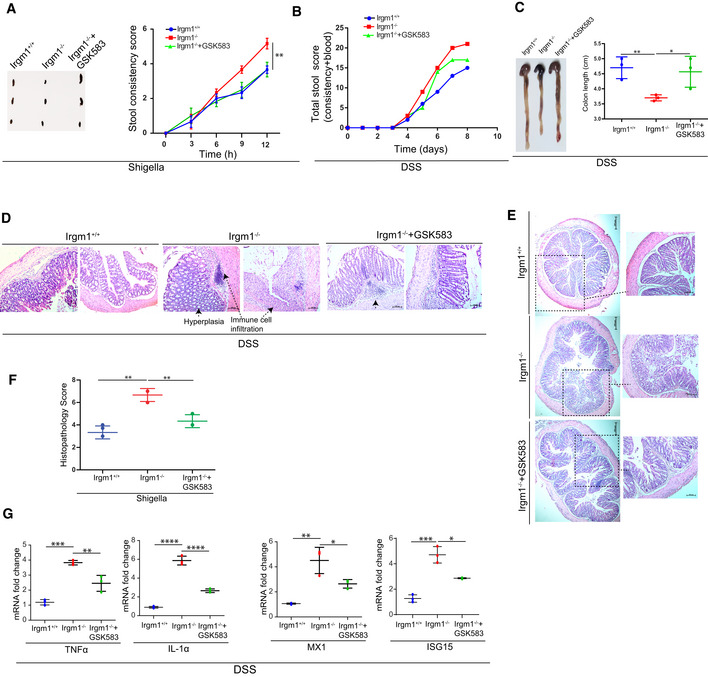
RIPK2 inhibition ameliorates shigellosis and DSS‐induced gut inflammation in *Irgm1* knockout mice Right panel, representative picture of fecal samples collected from *S. flexneri*‐infected *Irgm1* ^+/+^ or *Irgm1* ^−/−^ or GSK583‐treated *Irgm1* ^−/−^ mice. Left panel, the graph depicts fecal pathology scores based on stool consistency and color *of S. flexneri*‐infected and GSK583‐treated C57BL/6 mice. (*n* = 6 mice in each group, Mean ± SD. **P < 0.005, Student's unpaired *t*‐test).The graph depicts the total stool scores (stool consistency + blood) of DSS‐treated *Irgm1* ^+/+^ or *Irgm1* ^−/−^ or GSK583‐treated *Irgm1* ^−/−^ mice.Right panel, representative picture of colons of DSS‐treated *Irgm1* ^+/+^ or *Irgm1* ^−/−^ or GSK583‐treated *Irgm1* ^−/−^ mice; Left panel, the graph depicts the average colon length of same. (*n* = 3 mice each group, Mean ± SD. *P < 0.05, **P < 0.005, Student's unpaired *t*‐test).Representative microscopic images of hematoxylin–eosin (H&E) staining of colon tissue of DSS‐treated *Irgm1* ^+/+^ or *Irgm1* ^−/−^ or GSK583‐treated *Irgm1* ^−/−^ mice.Representative microscopic images of hematoxylin–eosin (H&E) staining of colon tissue of *S. flexneri*‐infected *Irgm1* ^+/+^ or *Irgm1* ^−/−^ or GSK583‐treated *Irgm1* ^−/−^ mice.The graph depicts the histopathology score (average pathological scores from HE staining based on hyperplasia, inflammatory cells infiltration, epithelial cell death, and loss of goblet cells) of *S. flexneri*‐infected *Irgm1* ^+/+^ or *Irgm1* ^−/−^ or GSK583‐treated *Irgm1* ^−/−^ mice. (*n* = 3 mice in each group, Mean ± SD, **P < 0.005, Student's unpaired *t*‐test).The qRT–PCR analysis for indicated genes with the total RNA isolated from the colons of DSS‐treated *Irgm1* ^+/+^ or *Irgm1* ^−/−^ or GSK583‐treated *Irgm1* ^−/−^ mice. (*n* = 3 mice in each group, Mean ± SD. *P < 0.05. **P < 0.005, ***P < 0.0005, ****P < 0.00005, ordinary one‐way ANOVA (Tukey's multiple comparisons test). Source data are available online for this figure.

For *Shigella* infection in mice, we used the intraperitoneal model of shigellosis (Yang *et al*, 2014). In both colitis models, *Irgm1*‐deficiency significantly accelerated body weight loss (Fig 7A and B) and increased the scores of stool consistency and rectal bleeding (Fig EV5A and B). Treatment of *Irgm1* ^−/−^ mice with the RIPK2 inhibitor GSK583 (Haile *et al*, 2016; Goncharov *et al*, 2018) significantly ameliorated the acute colitis symptoms (Figs 7A and B, and EV5A and B). Consistently, GSK583 was able to suppress the colon shortening in *Irgm1* ^−/−^ mice in both shigellosis‐ and DSS‐induced colitis models (Figs 7C and EV5C). Next, we examined the severity of colon damage and inflammation by histopathological analysis using hematoxylin and eosin (H&E) staining. In *Irgm1* ^−/−^ mice, a significant increase in DSS‐induced epithelial injury, loss of goblet cells, hyperplasia, and immune cell (neutrophils) infiltration was observed in the colon (Figs 7D and EV5D). Treatment of *Irgm1* ^−/−^ mice with GSK583 significantly attenuated histopathology and immune cell invasion (Figs 7D and EV5D). Similarly, *Shigella*‐induced colon histopathology in *Irgm1* ^−/−^ mice was significantly improved by treatment with GSK583 (Fig EV5E and F).

An enhanced oligomerization of RIPK2 was observed in *Irgm1* ^−/−^ mice colon lysates that were dampened by the administration of GSK583 in these mice (Fig 7E). The increased levels of NF‐κB (IL1α, IL1β, and TNF‐α) and IFN (MX1, IFIT1, and ISG15) dependent cytokine response in *Irgm1* ^−/−^ mice colon were suppressed by treatment of GSK583 in both shigellosis‐ and DSS‐induced colitis models (Figs 7F and EV5G). Immunohistochemistry analysis showed enhanced staining of IL1β in *Irgm1* ^−/−^ mice colon that was significantly reduced in GSK583‐treated *Irgm1* ^−/−^ mice (Fig 7G). The enhanced expression of ISG15, RSAD2 (Viperin), and protein ISGylation (IFN response markers) in *Irgm1* ^−/−^ mice colon was considerably reduced upon treatment with GSK583 (Fig 7H). Similarly, GSK583 administration considerably diminished the increased protein levels of pro‐caspase‐1, cleaved caspase‐1, phospho‐p65, and phospho‐p38MAPK in *Irgm1* ^−/−^ mice colon (Fig 7H and I).

Taken together, the data show that RIPK2 inhibition can ameliorate the gut inflammation and pathology associated with *Irgm1* deprivation in mouse colitis models.

## Discussion

In this study, we made two major advances in understanding the regulation of NODs‐RIPK2‐NF‐κB signaling. First, we revealed that when pathogenic bacteria infect cells, RIPK2 forms RIPosomes, which recruit over the bacteria and induce NF‐κB response. Second, we show that autophagy suppresses NF‐κB pro‐inflammatory signaling by selectively degrading NODs, RIPK2, and RIPosomes. Consequently, this study demonstrates how the two cells' autonomous systems that are loaded over the bacteria work in concert for innate immune defense against the pathogens and cutting down excess inflammation as a safeguard.

The cryogenic electron microscopic (Cryo‐EM) structure of RIPosomes was illustrated by two recent studies (Gong *et al*, 2018; Pellegrini *et al*, 2018). They found that RIPK2 forms a filamentous structure in cells using its CARD domain. Interestingly, the helical symmetry of RIPosomes and ASC filaments (Lu *et al*, 2014) was found to be strikingly similar suggesting that their assembly is governed by a similar mechanism. Further, they suggest that the CARDS of NOD1/2 may transiently interact with the CARD of RIPK2 to induce their oligomerization, similar to the phenomenon observed in RIG‐I–MAVS signaling complexes (Wu & Hur, 2015). We found that RIPK2 can self‐polymerize; however, both NOD1 and NOD2 failed to do so unless co‐expressed with RIPK2. This is consistent with previous studies that suggest that NOD1/2 has a low propensity to self‐associate (Fridh & Rittinger, 2012; Gong *et al*, 2018). Taken together, it appears that NODs monomers trigger RIPK2 oligomerization, which in turn increases the propensity of NODs to oligomerize and form NODo‐RIPosomes.

Ellwanger *et al*, 2019 demonstrated that XIAP controls RIPK2 signaling by preventing its depositions in speck‐like structures. Given the role of XIAP in autophagy, it will be interesting to determine whether XIAP has a role in the autophagic degradation of RIPosomes.

We found that upon bacterial infection endogenous RIPK2 undergoes oligomerization to form RIPosomes in macrophages. Also, we found that pathogenic bacteria have a greater ability to induce RIPosome formation than nonpathogenic bacteria. At present, we do not understand the basis of this difference as both pathogenic and nonpathogenic bacteria produce ligands for NODs activation. It could be a possibility that, in addition to these ligands, some other bacterial factors are required to induce endogenous oligomerization of RIPK2. Consistently, the NODs agonist, MDP, or iE‐DAP were inefficient in inducing RIPosome formation. Further studies are needed to identify the bacterial factors that enhance RIPosomes biogenesis. RIPosomes were either juxtaposed or recruited over the pathogenic bacteria. While NODs puncta co‐localize with RIPosomes over bacteria, they are less consistent than RIPosome depositions suggesting that like RIG‐I‐MAVS interaction (Wu & Hur, 2015), NODs might transiently interact with RIPK2 to activate it.

The NODs‐RIPK2‐dependent NF‐κB pro‐inflammatory response is important to eliminate the invading pathogens. However, extensive inflammation could be deleterious and lead to immuno‐pathologies. To prevent damage, cells employ several cell‐autonomous mechanisms to tone down the inflammation. Genetic mutations in components of the NODs‐RIPK2‐NF‐κB pathway predispose toward chronic autoimmune and inflammatory diseases (Miceli‐Richard *et al*, 2001; Kanazawa *et al*, 2005; Henckaerts & Vermeire, 2007; Caso *et al*, 2015; Taniguchi & Karin, 2018). Therefore, it is critically important to understand the mechanisms that negatively control this pathway. Autophagy plays a significant role in suppressing inflammation and maintaining innate immune balance in the cell by degrading multiple inflammatory proteins/complexes (Chauhan *et al*, 2021; Deretic, 2021). Our previous study showed that IRGM act as a scaffold protein to bring NOD2‐dependent bacterial sensing and autophagy machinery together to conduct antimicrobial defense (Chauhan *et al*, 2015). Here, we found that IRGM utilizes autophagy machinery to dampen the NOD2‐RIPK2‐dependent inflammation induced by pathogenic bacteria. Together, it emerges that IRGM has a dual function during bacterial infection. On the one hand, it leads to the clearance of intracellular bacteria by inducing xenophagy, and on the other hand, it reduces inflammation by inducing autophagic degradation of NODs and RIPK2.

IRGM is a genetic risk factor for several inflammatory and infectious diseases including inflammatory bowel disease, and tuberculosis (Massey & Parkes, 2007; Parkes *et al*, 2007; Lu *et al*, 2013; Kimura *et al*, 2014; Lin *et al*, 2016; Xia *et al*, 2017; Yao *et al*, 2018). The depletion of *Irgm1* in mice triggers inflammasome and IFN responses leading to autoimmune‐like conditions. Depletion of IRGM led to upregulation of several inflammatory responses including NF‐κB and IFN responses in a RIPK2‐dependent manner that is blocked by chemical inhibition of RIPK2. These data suggest that RIPK2 inhibitors could be used as therapeutic options in patients with inflammatory diseases caused by the loss of function of IRGM.

## Materials and Methods

### Cell culture

All the common cell lines including THP‐1 (ATCC TIB‐202), HT‐29 (ATCC HTB‐38), and HEK293T (ATCC CRL‐3216) cells were obtained from the American Type Cell Culture (ATCC), US. HEK‐Blue hNOD2 cells were purchased from InvivoGen and maintained as per the instructions. HT‐29, HEK293T, and HeLa cells were maintained in DMEM (GIBCO) supplemented with 10% heat‐inactivated fetal bovine serum (FBS) and penicillin/streptomycin. THP‐1 cells were maintained in RPMI‐1640 (GIBCO) supplemented with 10% heat‐inactivated FBS, Glucose (5%), HEPES buffer (10 mM), L‐glutamine (5 mM), sodium pyruvate (1 mM), and penicillin/streptomycin. All the cell lines were tested for mycoplasma contamination routinely (every 2–3 months). All the experiments were performed with cells below the 20^th^ passage.

### Generation of CRISPR/Cas9 KO cell lines

The RIPK2 knockout HT‐29 cell line is generated using the CRISPR‐Cas9 method. Briefly, HT‐29 cells seeded in 6‐well plates and transfected with RIPK2/RICK Double Nickase Plasmid (Santacruz; sc‐400,731‐NIC) using ViaFect transfection reagent (Promega # E4981). After 48 h, GFP‐positive cells were sorted into 96‐well plates using MoFlo Asterio cell sorter (Beckman Coulter Life Sciences). The cells were grown in a growth medium containing 1 μg/ml puromycin for 1 week. The individual clones were selected and screened for knockout using Western blot analysis. The IRGM knockout HT‐29 cell line was generated as described previously (Jena *et al*, 2020).

### Inhibitors and reagents

N‐Acetylmuramyl‐L‐alanyl‐D‐isoglutamine, MDP (InvivoGen # tlrl‐mdp); γ‐D‐glutamyl‐meso‐diaminopimelic acid, iE‐DAP (InvivoGen # tlrl‐dap); 6‐[(1,1‐dimethylethyl) sulfonyl]‐N‐(5‐fluoro‐1H‐indazol‐3‐yl)‐4‐quinolinamine, GSK583 (Cayman # 19739); Phorbol 12‐myristate 13‐acetate, PMA (Sigma # P8139); Bafilomycin A1 (CST# 54645S); Z‐leu‐leu‐leu‐al, MG132 (Sigma # C2211‐5MG); Dextran sulfate sodium salt, DSS (MP Biomedicals # 160110), Ubiquitin E1 Inhibitor, PYR‐41 (Sigma # 662105), ProLong Gold antifade reagent with DAPI (Invitrogen # P36931), ProLong Gold antifade reagent (Invitrogen # P36930).

### Soluble‐insoluble fractionation

For soluble‐insoluble protein fractionation, cells were lysed in 1% Triton X‐100 lysis buffer containing 150 mM NaCl, 50 mM Tris (pH 7.5), 10% glycerol, 1 mM EDTA, 1 mM PMSF, and protease inhibitor cocktail at 4°C for 20 min followed by centrifugation for 10 min at 17,000 *g* at 4°C. After centrifugation, the supernatant was used as a soluble fraction and the pellet was resuspended in lysis buffer with 1% SDS and used as an insoluble fraction for Western blot analysis.

### Nuclear and cytoplasmic fractionation

Nuclear and cytoplasmic fractionation assay was performed using the NE‐PER Nuclear and Cytoplasmic Extraction Reagents (ThermoFisher Scientific # 78833) according to the manufacturer's protocol. Briefly, 2 × 10^6^ HeLa cells were infected with *S. flexneri* (MOI 1:25) for 4 h. The cells were harvested with trypsin–EDTA and centrifuged at 500 × *g* for 5 min. The cell pellet was washed with 1× PBS and resuspended in 200 μl CER I, vortexed vigorously for 15 s, and incubated on ice for 10 min. After the incubation, 11 μl of ice‐cold CER II were added to each sample, vortexed followed by incubation on ice for 1 min. The samples were centrifuged at 16,000 × *g* for 5 min to extract cytoplasmic fraction (supernatant) and the pellet (insoluble fraction) was resuspended in 100 μl NER, vortexed, incubated on ice with intermittent vortexing for 40 min. The samples were centrifuged at 16,000 × *g* for 10 min at 4°C to isolate nuclear fraction and subjected to western blot analysis.

### Cycloheximide chase assay

Approximately, 2 × 10^6^ HT‐29 cells were plated in a 6‐well plate and grown overnight. The next day, cells were treated with cycloheximide (100 μg/ml) with or without Bafilomycin A1 (300 nM) or MG132 (20 μM) for various time points. The cells were lysed in NP‐40 lysis buffer containing 1× protease inhibitors cocktail and 1 mM PMSF and subjected to Western blotting with indicated antibodies.

### Western blotting

The cells were lysed using NP‐40 lysis buffer (Invitrogen # FNN0021) containing 1 mM PMSF (Sigma # P7626), phosSTOP (Roche # 49068455001), and protease inhibitors cocktail (Roche # 1183617000). Mice tissue were homogenized in radio‐immunoprecipitation assay (RIPA) buffer (20 mM Tris, pH 8.0; 0.5 EGTA; 1 mM EDTA; 150 mM NaCl; 1% IGEPAL (Sigma); 10% glycerol; 0.1% Sodium deoxycholate). The protein concentration in the lysate was determined using the BCA method (Pierce™ BCA Protein Assay Kit # 23225). Lysates were separated using SDS–PAGE, transferred onto a nitrocellulose membrane (Bio‐Rad), and blocked for 1 h in 5% skimmed milk followed by incubation in primary antibody overnight at 4°C. The membrane was washed with 1× PBS/PBST three times and then incubated with HRP conjugated secondary antibody for 1 h at room temperature. After washing with 1× PBS/PBST, the blots were developed using enhanced chemiluminescence reagents.

### Immunofluorescence assay

Approximately, HEK293T/HeLa 10^5^ cells were seeded on a coverslip and allowed to adhere overnight before transfection with plasmids. For THP‐1 cells, approximately, 5 × 10^5^ cells were treated with 50 ng/ml of PMA (Sigma # P8139) and seeded on the coverslip for differentiation into macrophages for 16 h. After a resting period of 24 h, cells were treated with stimulants or infected as required. The cells were fixed in 4% paraformaldehyde for 15 min, permeabilized with 0.1% Triton X‐100 (or 0.1% saponin) for 15 min, followed by blocking with 1% bovine serum albumin (BSA) for 30 min at room temperature (RT). The permeabilized cells were then incubated with primary antibody as indicated for 1 h at RT or overnight at 4°C, washed thrice with 1× PBS, followed by incubation with Alexa fluor‐conjugated secondary antibodies for 1 h at RT. The cells were washed thrice with 1× PBS, mounted with ProLong™ Gold antifade mountant with or without DAPI, air‐dried, and visualized using a super‐resolution Leica SP8 lightning confocal microscope.

### Proximity ligation assay (PLA)

Proximity ligation assay (PLA) was performed using a Duolink *in situ* detection kit as per the manufacturer's protocol (Sigma #DUO92008). Briefly, 2 × 10^5^ HEK293T cells were seeded on the coverslip and allowed to adhere overnight before transfection with the desired plasmids. After 12 h of transfection, cells were fixed with a 4% paraformaldehyde solution. Next, antigen retrieval was performed in sodium citrate buffer (10 mM, pH 6.0) and the cells were permeabilized with 0.1% Triton X‐100 followed by blocking with 1× blocking solution (provided with the kit) in a preheated humidity chamber for 1 h at 37°C. The cells were incubated overnight with primary antibody diluted with diluent at 4°C. Next, the coverslips were washed twice with 1× wash buffer‐A followed by incubation with Duolink *in situ* PLA probes in a preheated humidity chamber for 1 h at 37°C. After PLA probe incubation, coverslips were washed twice with 1× wash buffer‐A followed by ligation with Duolink *in situ* ligase for 30 min in a preheated humidity chamber at 37°C. Next, coverslips were washed twice with 1× wash buffer‐A followed by amplification through Duolink *in situ* polymerase for 90 min in a preheated humidity chamber at 37°C. Final washing was done twice with 1× and 0.01× wash buffer‐B. The coverslips were mounted over glass slides using Duolink *in situ* mounting media with DAPI and the edges of the coverslip were sealed, mounted, and visualized using a Leica SP8 confocal microscope.

### High‐content microscopy imaging

For high‐content microscopy imaging, approximately 17,000 HEK293T cells, 8,000 HeLa cells, and 50,000 PMA‐treated THP‐1 cells were seeded in a black flat bottom 96‐well plate (Thermo Scientific, Nunc) and transfected with plasmids or treated or infected as indicated. The cells were fixed with 4% PFA and permeabilized with 0.1% Triton X‐100 for 15 min, followed by blocking with 1% bovine serum albumin (BSA) for 30 min and incubation with primary antibody for 1 h. The cells were washed thrice with 1× PBS and incubated with secondary antibody for 1 h and then counterstained with 0.5 μg/ml of DAPI for 5 min. Imaging was performed by using Cell insight CX7 LZR high‐content screening platform (Thermo Scientific). Automated image scanning and analysis were carried out using HCS studio and iDEV software, respectively. Automated images were captured at 10× (or 20×) by taking 1,500–20,000 cells and 35 fields per well or as indicated in figure legends. Images were quantified using scanning parameters and object mask definitions. A threshold value was set to find out the primary objects (cells). DAPI staining was used for autofocus and identification of the primary objects. The cells and regions of interest (ROI) or desired targets were further validated by total area, shape/segmentation, maximum/minimum average intensity, and total intensity. By using the cell mask and intensity of the puncta, the number of puncta per cell was counted. All data processed and analyzed were computer‐based.

### Hematoxylin and eosin (H&E) staining and immunohistochemistry analysis for mice colon tissue

The colon sections were deparaffinized and hydrated through an alcohol gradient followed by 1× PBS wash. For hematoxylin and eosin staining, the sections were stained with hematoxylin for 5 min followed by washing in water to remove excess stain. The sections were then incubated in Scott's tap water followed by staining with Eosin dye. The slides were washed, dehydrated in absolute ethanol, cleared in xylene, and mounted with DPX. Finally, slides were observed under Zeiss Apotome 2.0 microscope.

For immunohistochemistry, colon sections were deparaffinized and hydrated through ethanol gradient followed by 1× PBS wash. Antigen retrieval was performed in sodium citrate buffer (pH 6.0) for 10 min followed by permeabilization with 1× PBS (pH 7.4) containing 0.25% Triton X‐100, blocked with goat serum. The slides were washed for 2 min thrice with PBS (pH 7.4) containing 0.05% Tween 20 followed by incubation of sections with an unconjugated affinity purified F(ab) fragment anti‐mouse IgG (H + L) (Abcam #ab6668) for 1 h at room temperature. The sections were incubated overnight at 4°C with antibodies as indicated. The next day, the sections were washed twice with 1× PBS (pH 7.4) containing 0.05% Tween 20, followed by incubation with goat anti‐rabbit/mouse IgG (H + L) Alexa Fluor 488/568 conjugated secondary antibody for 1 h. Sections were again washed and counterstained with DAPI for 1 min followed by incubation with an auto‐fluorescence quencher (Vector Labs #SP‐8400). Sections were finally mounted with Vectashield Vibrance Antifade mounting media. The slides were visualized under Leica TCS SP8 STED confocal microscope.

### Co‐immunoprecipitation assay

The cells were lysed in NP‐40 lysis buffer containing 1× protease inhibitors cocktail and 1 mM PMSF for 20 min at 4°C and centrifuged at 12,000 *g* for 30 min at 4°C. The supernatant was incubated with the antibody at 4°C for 2 h on a rotational cell mixer followed by incubation with protein G Dynabeads (ThermoFisher#10003D) for 2 h at 4°C on a rotational cell mixer. The beads were washed with ice‐cold 1× PBS four times, and the proteins were eluted from the beads in 2× SDS–PAGE Laemmli buffer by boiling for 5 min for Western blot analysis.

### Glutathione S‐Transferase (GST)‐pull‐down assay

The glutathione S‐transferase (GST) pull‐down assay was performed according to the previously described methods (Mehto *et al*, 2019a, 2019b). Briefly, GST or GST‐RIPK2 or GST‐IRGM recombinant proteins were expressed in *E. coli* SoluBL21 (Amsbio), and the proteins were purified on Glutathione Sepharose 4 Fast‐Flow beads (GE Healthcare). The [^35^S] labeled‐ Myc‐NOD1, Myc‐NOD2, Myc‐RIPK2, Myc‐ULK1, Myc‐ATG16L1 or Myc‐Beclin‐1 proteins were *in vitro* translated using TnT T7–coupled reticulocyte lysate system (Promega). The GST proteins were incubated with [^35^S]‐labeled proteins in 200 μl of NETN‐E buffer (50 mmol/l Tris, pH 8.0, 100 mm NaCl, 6 mm EDTA, 0.5% NP‐40, and 1 mm dithiothreitol (DTT) supplemented with complete mini EDTA‐free protease inhibitor cocktail; Roche) for 2 h at 4°C. After incubation, the beads were washed five times with NETN‐E buffer, boiled with loading buffer, and subjected to SDS–PAGE. The gels were stained with coomassie blue and vacuum‐dried. The GST was detected by staining with coomassie blue stain, whereas the [^35^S]‐ labeled Myc‐tagged proteins were detected in PharosFX imager (Bio‐Rad Laboratories).

### Antibodies and dilutions

#### Western blotting:

IRGM (Abcam # 69494; 1:500), NOD1 (CST# 3545S; 1:1000), NOD2 (Proteintech # 20980‐1‐AP; 1:1000), RIPK2 (CST#4142S; 1:1000), Anti‐beta Actin (Abcam # ab6276; 1:5000), GFP (Abcam # ab290; 1:5000), Flag M2 (Sigma # F3165; 1:1000), p62 (BD Biosciences # BD‐610832; 1:1000), ATG5 (CST # 2630S; 1:1000), c‐Myc (Santa Cruz # sc‐40; 1:1000), HA‐Tag (CST # 3724S; 1:1000), RICK (Santa Cruz # sc‐22,763; 1:1000), Anti‐LC‐3B antibody (Sigma # L7543; 1:1000), Anti‐RICK A‐10 (Santa Cruz # sc‐166,765; 1:1000), IL‐1β (CST#12242), Anti‐pro Caspase1 + p10 + p12 Antibody (Abcam # ab179515; 1:1000), ISG15 (Santa Cruz# sc‐166755; 1:500), Phospho‐p38 MAPK(Thr180/Tyr182; 1:1000; CST#9211; 1:1000), Phospho‐NF‐κB p65 (ser536) (93H1) (CST# 3033; 1:1000). HRP conjugate secondary antibodies were purchased from Novus (1:2000) or Promega (1:5000).

#### Immunofluorescence

IRGM (Santa Cruz #68202; 1:50), RICK H‐300 (Santa Cruz # sc‐22,763; 1:100), NOD2 (Millipore # 04‐145; 1:50), p62 (BD Biosciences # BD‐610832; 1:250), LC3b (Sigma # L7543; 1:250), LC3b (MBL # PM036; 1:100), LAMP2A (Abcam # ab18528; 1:50), EEA1 (CST # 3288; 1:50), FK2 (MBL # D058‐3;1:250), LC3b (CST # 83506, 1:50), IL‐1β (CST # 12242), ULK1 (Santa Cruz # 33182; 1:100), c‐Myc (Santa Cruz # sc‐40; 1:500), Flag M2 (Sigma # F3165; 1:250), Alexa fluor Secondary antibodies (1:2000) were purchased from Thermo Fisher Scientific.

### Plasmids and deletion constructs

The mcherry‐RIPK2, GFP‐RIPK2 and its deletion construct, GFP‐NOD1 and its deletion construct and GFP‐NOD2 were generated using gateway cloning strategy as per standard protocols (Invitrogen). pGL4.32[luc2P NF‐kB‐RE Hygro] purchased from Promega (Promega # E8491).

### Transient transfection with siRNA

The THP‐1 cells were electroporated using the Neon transfection system (Invitrogen # MPK5000; setting: 1400 V, 10 ms, 3 pulses, 100 μl tip) with 30 nM siRNA or as indicated: nontargeting siRNA, p62 siRNA (SASI_Hs01_00118616), IRGM siRNA (SASI_HS02_00518571), ATG5 (SASI_Hs01_00173156), human RIPK2 siRNA (SASI_Hs01_00199696), mice RIPK2 siRNA (SASI_Mm01_00188069) from Sigma‐Aldrich. After 24‐h transfection, one more time transfection was performed in a similar condition and incubated for an additional 48 h before the start of each experiment. The HT‐29, HeLa, and HEK293T cells were transfected with 30 nM siRNA using Lipofectamine RNAiMAX transfection reagents (Invitrogen# 13778075) as per the manufacturer's instructions.

### Transient transfection with plasmid

For transient transfection in THP‐1, cells were electroporated using the Neon transfection system (Invitrogen # MPK5000; setting: 1400 V, 10 ms, 3 pulses, 100 μl tip). For overexpression experiments in HEK293T cells, required plasmids were transfected using the calcium phosphate method as per the manufacturer's instruction (Clontech, Promega).

### Flag‐RIPK2 protein electroporation

Approximately, 2 × 10^6^ HEK293T cells transiently expressing pGL4.32NFκB‐RE reporter plasmid electroporated with 10 μg Purified Flag‐RIPK2 in 100 μl neon resuspension buffer (setting: 1300 V, 10 ms, 1 pulse, 100 μl tip). The cells were harvested 6‐h postelectroporation, and a luciferase assay was performed as described below.

### NF‐κB reporter assay

The NF‐kb reporter assay was performed using a luciferase assay kit (Promega) according to the manufacturer's instructions and as described previously (Jena *et al*, 2020). Briefly, HEK293T cells were seeded into the 24‐well plate. The next day, cells were transfected with pGL4.32 NF‐κB‐RE (Addgene100 ng) together with required plasmids using the calcium phosphate method. After 9 h, the growth medium was removed and cells were washed thrice with 1× PBS and lysed using 100 μl (1X) passive lysis buffer (Promega). The cell lysates were cleared by centrifugation at 12,000 *g* for 30 s at 4°C, and protein concentration was estimated using the BCA method. In a 96‐well plate, 15 μg/20 μl lysate mixed with 100 μl LARII reagent and luminescence measurement was performed using a PerkinElmer VICTOR Nivo Multimode plate reader.

### Secreted embryonic alkaline phosphatase (SEAP) reporter assay

Approximately, 3 × 10^5^ HEK293‐hNOD2 Blue cells were plated in a 6‐well culture plate and incubated at 37°C in a 5% CO_2_ incubator overnight. The next day, cells were transfected with the plasmids as indicated using the calcium phosphate method as per the manufacturer's instruction (Clontech, Promega). Six‐hours post‐transfection, the cell culture medium was replaced with fresh medium with or without L‐18 MDP (100 ng/ml) and incubated at 37°C in a 5% CO_2_ incubator for 24 h. SEAP activity was determined using QUANTI‐Blue reagent as per the manufacturer's instruction. Briefly, in a flat bottom 96‐well plate, 20 μl of sample supernatant was mixed with 180 μl of QUANTI‐Blue solution and incubated for 30 min at 37°C. Optical density (OD) was measured at 620–655 nm using a microplate reader (Bio‐Rad).

### RNA isolation and quantitative real‐time PCR

The total RNA was extracted using TRIzol™ isolation reagent (Invitrogen # 15596026) according to the manufacturer's protocol. One to two microgram of total RNA was used to synthesize cDNA using the high‐capacity cDNA synthesis kit (Applied Biosystem #4368813), and qRT–PCR was performed using Power SYBR green PCR master mix (Applied Biosystem #4367659) or TaqMan master mix (Applied Biosystem # 4369016) according to the manufacturer's protocol. The assay was normalized using the housekeeping gene (GAPDH or β‐Actin). The fold change in gene expression was calculated by the 2^−▵▵Ct^ method. The graphs were generated using Graph Pad software. The sequence for human and mouse qRT–PCR primers is shown in Appendix Table S1.

### RNA‐sequencing sample preparation

The total RNA was extracted from the cells using an RNeasy mini kit (QIAGEN #74104). The quality and quantity of RNA were checked using agarose gel and Qubit 3.0. After assessing the quality of RNA, ~850 ng of total RNA was taken for library preparation using NEBNext®Ultra™ II Directional RNA Library kit for Illumina® (# E7760L) and NEBNext® Poly (A) mRNA Magnetic Isolation Module (# E7490L) as per manufacturer's protocol. The prepared library was quantified using a Qubit dsDNA assay kit (Invitrogen, Q32851) followed by a quality check (QC) and fragment size distribution using a High Sensitivity Tape station Kit (Agilent 2200, 5067–5585, and 5067–5584). The library was sequenced using the HiSeq 4000 Illumina platform.

### RNA‐sequencing data processing and gene expression analysis

The paired‐end (PE) reads quality checks for each sample were performed using FastQC v.0.11.5 (http://www.bioinformatics.babraham.ac.uk/projects/fastqc/). The adapter sequence was trimmed using the BBDuk version 37.58, and the alignment was performed using STAR v.2.5.3a with default parameters (Dobin *et al*, 2013) with human hg38 genome build, gencode v21 gtf 9GRCh38 from the gencode. Duplicates were removed using Picard‐2.9.4 (https://broadinstitute.github.io/picard/) from the aligned files, and read counts were generated using featureCount v.1.5.3 from subread‐1.5.3 package (https://bioweb.pasteur.fr/packages/pack@subread@1.5.3) with Q = 10 for mapping quality. The count files were used as input for downstream differential gene expression analysis with DESeq2 version 1.14.1 9. (Love *et al*, 2014). The genes with read counts of ≤ 10 in any comparison were removed followed by count transformation and statistical analysis using DESeq “R.” The “*P*” values were adjusted using the Benjamini and Hochberg multiple testing correction (Haynes, 2013), and the differentially expressed genes were identified (fold change of ≥ 1.5, *P*‐value < 0.05). A unified nonredundant gene list was made for different comparisons and subjected to gene ontology (GO) analysis using reactome database (https://reactome.org/). The top pathways (*P* < 0.05) were used for generating heat maps using Complexheatmap (Version 2.0.0) through unsupervised hierarchical clustering. The expression clusters were annotated based on enriched GO terms. Normalized gene expression was used to generate the boxplots with median depicting the trends in the expression across the different conditions using ggplot2 [version 3.3.5]. The pathways analysis was performed using Metascape database (https://metascape.org/gp/index.html#/main/step1). The top pathways (*P* < 0.05) were taken for constructing bubble plots using ggplot2 [version 3.3.5].

The basal conditions groups (for the *Salmonella* infection group) for the dataset E‐MTAB‐12074 are the same as described previously (Jena *et al*, 2020) and deposited under the accession number E‐MTAB‐9142 (http://www.ebi.ac.uk/arrayexpress/experiments/E‐MTAB‐9142). The RNA‐sequencing experiment for the basal conditions (dataset E‐MTAB‐9142) and Salmonella‐infected conditions (dataset E‐MTAB‐12074) was performed together. However, we have used basal conditions groups' dataset E‐MTAB‐9142 in our previous publication (Jena *et al*, 2020) and reanalyzed it here along with Salmonella‐infected dataset E‐MTAB‐12074.

### Bacterial strains and infection of cells in culture

*Escherichia coli* DH5α, *Salmonella typhimurium* strain *S*T 1433, and *Shigella flexneri* (MTCC:1457) (gifted by Dr. Suraj Kumar Tripathy, KIIT, India) were cultured in Luria‐Bertani (LB) overnight at 37°C with shaking. Bacteria were subcultured (1:100 dilution) in fresh LB broth and grown until OD_600_ reached 0.4–0.6. *Mycobacterium smegmatis* MC^2^ 155 *and Mycobacterium tuberculosis* H37Rv (gifted by Dr. Sunil Raghav, ILS, India) were grown in Middlebrook 7H9 liquid medium supplemented with 5 g/l albumin, 2 g/l dextrose, and 0.003 g/l catalase along with 0.05% tween 80.

For fluorescence microscopy, THP‐1 cells were treated with PMA (50 ng/ml) and seeded onto glass coverslips in 12‐well plates and kept for differentiation. After 16 h, the medium was replaced with fresh medium and incubated for another 24 h. Cells were infected with *Shigella flexneri* 1457 (MOI, 1:25 or 1:50), *Salmonella typhimurium* strain *S*T 1433 (MOI, 1:10), *E. coli* LF‐82 (MOI, 1:10), *Mycobacterium smegmatis* MC^2^ 155 (MOI, 1:10), *and Mycobacterium tuberculosis* H37Rv (MOI, 1:10). Infection was facilitated by centrifugation at 700 × *g* for 10 min at room temperature and proceeded for 2 h at 37°C on 5% CO_2_ incubator. Infected cells were washed three times with 1× PBS and fixed with 4% paraformaldehyde for 30 min at room temperature.

For the RNA‐sequencing experiment, approximately 3 × 10^6^ HT‐29 cells were seeded in a 60 mm dish and allowed to adhere overnight. The cells were infected with *Shigella flexneri* 1457 (1:25 MOI, 6 h) or *Salmonella typhimurium* strain *S*T 1433 (MOI, 1:10, 8 h).

### Intraperitoneal *Shigella* infection in mice

The mice experiments were performed with the procedure approved by the institutional animal ethical committee at the Institute of Life Sciences (ILS), Bhubaneswar, India. The mice were housed at the animal house facility of ILS. C57BL/6 wild‐type and *Irgm1* knockout mice were described previously (Liu *et al*, 2013). About 6–8‐week‐old male *Irgm1* ^+/+^ (wild‐type, *n* = 9) and *Irgm1* ^−/−^ (*n* = 18, includes GSK583 group) mice were used for the infection. Intraperitoneal infection of *S. flexneri* was performed as reported previously (Yang *et al*, 2014). Briefly, *Shigella* was cultured to the O.D. of 0.4–0.5 and 10^8^ colony‐forming units (CFU) were injected intraperitoneal into the mice. GSK583 (30 mg/kg of body weight) is administered intraperitoneally 6 h before the infection. Mice were monitored for body weight, stool consistency, and other clinical parameters. All mice were sacrificed 24‐h postinfection. The fecal pathology scores were assigned as follows: stool consistency (0, normal; 1, loose; 2, soft; 3, hard) and color (0, brown; 1, yellow; 2, light green; 3, green).

Pathological scores assigned in colon tissue sections for *Shigella* infection in mice based on the following parameter (Erben *et al*, 2014): hyperplasia (1, less than 25%; 2, mild 26–35%; 3, moderate 36–50%; 4, marked 51% above), loss of goblet cells(1, minimal less than 25%; 2, mild 26–35%; 3, moderate 36–50%; 4, marked 51% above), Leucocytes infiltrate (1, minimal less than 10%; 2, mild 26–35%; 3, moderate 36–50%; 4, marked 51% above).

### Dextran sulfate sodium (DSS)‐induced colitis model in mice

The 6–7‐week‐old female C57BL/6 *Irgm1* ^+/+^ (*n* = 3) and *Irgm1* ^−/−^ (*n* = 6, including GSK583 group) mice were used for the DSS‐induced colitis experiment. All the mice were given 2% (wt/vol) DSS dissolved in drinking water for 8 days. The DSS solution was replaced on alternate days. To inhibit the RIPK2, GSK583 (25 mg/kg) was injected intraperitoneally (i.p) 16 h before the start of DSS treatment and also injected intraperitoneally on the 1^st^, 3^rd^, 5^th^, and 7^th^ day. The control group mice were injected intraperitoneally with vehicle control. Each day, mice were monitored for body weight, stool consistency, and the presence of blood in feces. The stool scores were assigned as follows: 0, well‐formed pellets; 1, semisolid; 2, semisolid and not adhere to the anus; 3, liquid and adhere to the anus; and 4, diarrhea. Bleeding scores were assigned as follows; 0, no blood in stool; 1, light faint; 2, clear visible; and 3 gross rectal bleeding. The mice were sacrificed on the 9^th^ day. The entire colon was extracted and measured using the vernier scale. The colon tissue was homogenized in RIPA buffer or Triton X‐100 buffer containing PMSF, phosSTOP, and protease inhibitors cocktails and subjected to Western blotting. The tissue was homogenized in trizol for total RNA extraction.

Pathological scores assigned in colon tissue sections for DSS‐induced colitis mice based on the following parameter (Erben *et al*, 2014): hyperplasia (1, less than 25%; 2, mild 26–35%; 3, moderate 36–50%; 4, marked 51% above), loss of goblet cells (1, minimal less than 25%; 2, mild 26–35%; 3, moderate 36–50%; 4, marked 51% above), Leucocytes infiltrate (1, minimal less than 10%; 2, mild 26–35%; 3, moderate 36–50%; 4, marked 51% above).

### Software and statistical significance

GraphPad Prism 6 was used to analyze and present most of the data. High‐content microscopy imaging data were scanned and analyzed using HCS studio and iDEV software, respectively. The number of biological/technical replicates mentioned in each figure legend. The unpaired Student's *t*‐test or ordinary one‐way ANOVO (Tukey's multiple comparison test) statistical methods are used as appropriate. The *P*‐values are mentioned in graphs as appropriate. The densitometry analysis was performed using Image J software.

All the graphical representations are created using Biorender online tool (https://biorender.com/).

## Author contributions

**Santosh Chauhan:** Conceptualization; data curation; formal analysis; supervision; funding acquisition; investigation; visualization; methodology; writing – original draft; project administration; writing – review and editing. **Subhash Mehto:** Data curation; supervision; funding acquisition; validation; investigation; methodology; writing – review and editing. **Kautilya Kumar Jena:** Data curation; formal analysis; investigation; methodology; writing – review and editing. **Rina Yadav:** Data curation; investigation; methodology. **Swatismita Priyadarsini:** Validation; investigation; methodology. **Pallavi Samal:** Investigation; methodology. **Sivaram Krishna:** Investigation; methodology. **Kollori Dhar:** Investigation; methodology. **Ashish Jain:** Investigation; methodology. **Nishant Ranjan Chauhan:** Investigation; methodology. **Krushna Chandra Murmu:** Investigation; methodology. **Ramyasingh Bal:** Investigation; methodology. **Rinku Sahu:** Investigation; methodology. **Pundrik Jaiswal:** Investigation; methodology. **Bhabani Sankar Sahoo:** Investigation; visualization. **Srinivas Patnaik:** Supervision; methodology. **Thomas A Kufer:** Resources; writing – review and editing. **Tor Erik Rusten:** Resources; supervision. **Swati Chauhan:** Data curation; investigation; methodology. **Punit Prasad:** Resources; supervision; visualization.

## Disclosure and competing interests statement

The authors declare that they have no conflict of interest.

## Supporting information



Appendix

Expanded View Figures PDF

Dataset EV1

Dataset EV2

Dataset EV3

Dataset EV4

Dataset EV5

Dataset EV6

Movie EV1

Movie EV2

Movie EV3

Movie EV4

Source Data for Expanded View and Appendix

Source Data for Figure 1

Source Data for Figure 2

Source Data for Figure 3

Source Data for Figure 4

Source Data for Figure 5

Source Data for Figure 6

Source Data for Figure 7

PDF+

## Data Availability

The RNA‐seq datasets produced in this study have been deposited in the ArrayEXpress database at EMBL‐EBI (www.ebi.ac.uk/arrayexpress) under accession numbers E‐MTAB‐12067 and E‐MTAB‐12074.
