# Supplementary material for: Selective autophagy of RIPosomes maintains innate immune homeostasis during bacterial infection
Source: EMBO J. 2022 Oct 11;41(23):e111289. doi: 10.15252/embj.2022111289 (PMC9713718; doi:10.15252/embj.2022111289)
Supplement: Supplementary file 1 — Appendix [file EMBJ-41-e111289-s005.pdf]

“Selective Autophagy of RIPosomes Maintains Innate Immune Homeostasis during Bacterial Infection”

Mehto et al

Table of content

Appendix Figure S1 to S5

Appendix Figure legends S1 to S5

Appendix Table S1

Appendix Figure S1. Bacterial infection induces RIPosome formation

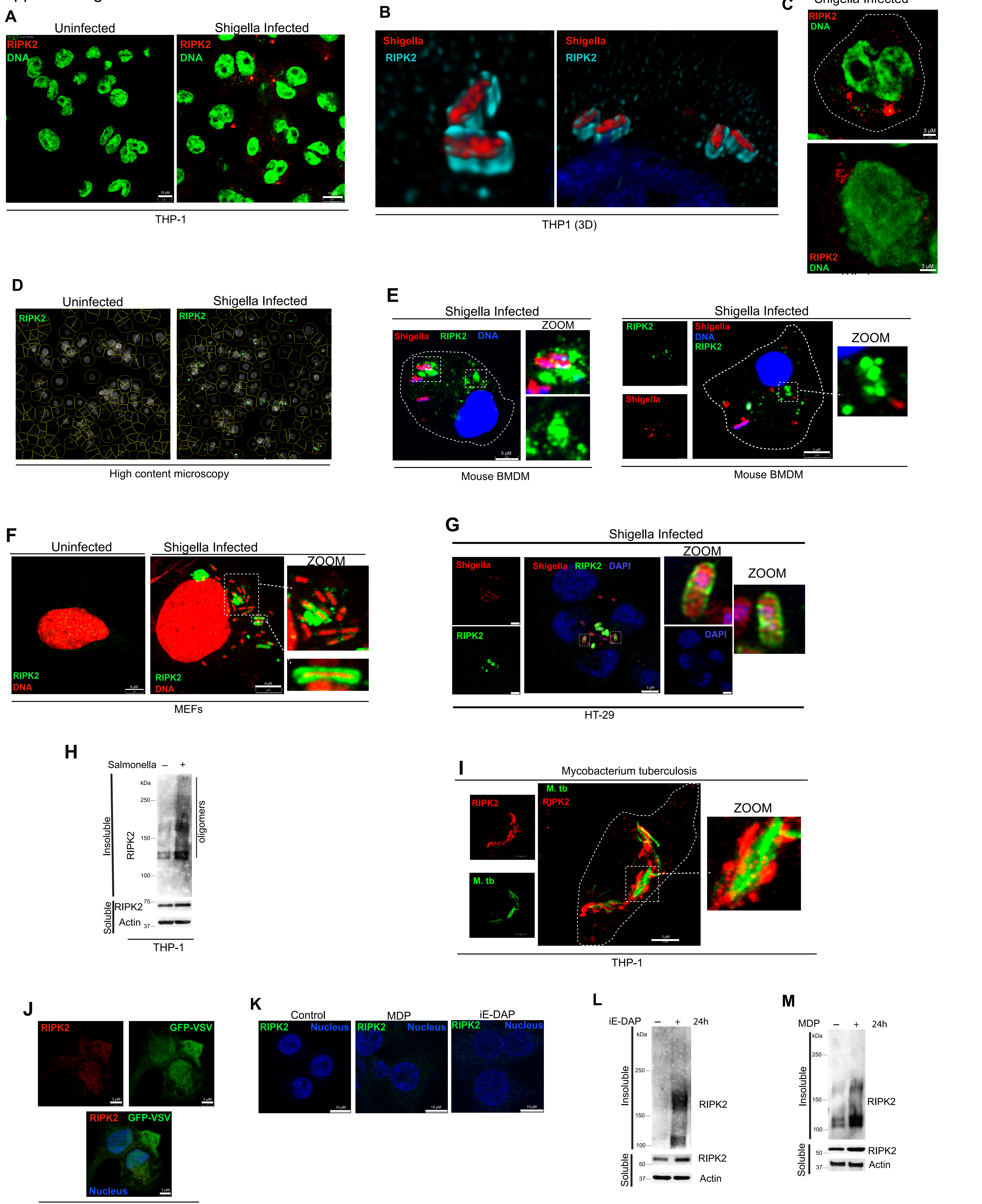

Appendix Fig S1. Bacterial infection induces RIPosomes formation

(A) Representative confocal images of uninfected and S. *flexneri* infected THP-1 cells (MOI 1:25, 8 hpi), Scale bar 10 µm.

(B) The digitally magnified three dimensional (3D) confocal images of RIPosomes recruited over RFP labelled S. *flexneri* in THP-1 cells (8hpi). DNA is stained with DAPI.

(C) Representative confocal images showing RIPosomes in S. *flexneri* infected THP-1 cells (MOI 1:25, 8 hpi).

(D) Representative high-content microscopy images of one full field (Yellow masks, software algorithms-defined cell boundaries) of RIPosomes in THP-1 cells infected with *S. flexneri* (MOI 1:25, 8 hpi).

(E-G) Representative confocal images of S. *flexneri* infected **(E)** mouse BMDM cells (Scale bar, 5 µm) or **(F)** MEFs cells (Scale bar, 3 µm) or **(G)** HT-29 cells (Scale bar, 8 µm). Zoom panels are digital magnification. DNA is stained with DAPI. DAPI is pseudocolored red in panel F for better contrast. In panel E and G, RFP-labelled S. *flexneri* was used for infection.

(H) Western blot analysis of soluble and insoluble fractions of THP-1 cell lysates, uninfected or infected with *Salmonella typhimurium* (4hpi).

(I) Representative confocal images of THP-1 cells infected with *M. tuberculosis* H37Rv (4 hpi). Zoom panels are digital magnification. Scale bar, 5 µm.

(J) Representative confocal images of RIPosomes in THP-1 cells infected with GFP VSV (MOI, 1:5, 8 hpi). DNA is stained with DAPI. Scale bar, 5 µm.

(K) Representative confocal images of RIPosomes in THP-1 cells treated with MDP (40 µg/ml) MDP or iE-DAP (40 µg/ml) for 24 h. DNA is stained with DAPI. Scale bar, 10 µm.

(L-M) Western blot analysis of soluble and insoluble fractions of THP-1 cells stimulated with **(L)** iE-DAP (40 µg/ml, 24 h) or**(M)** MDP (40 µg/ml, 24 h).

Appendix FigS2. NODs oligomerization is dependent on presence of RIPK2 oligomers

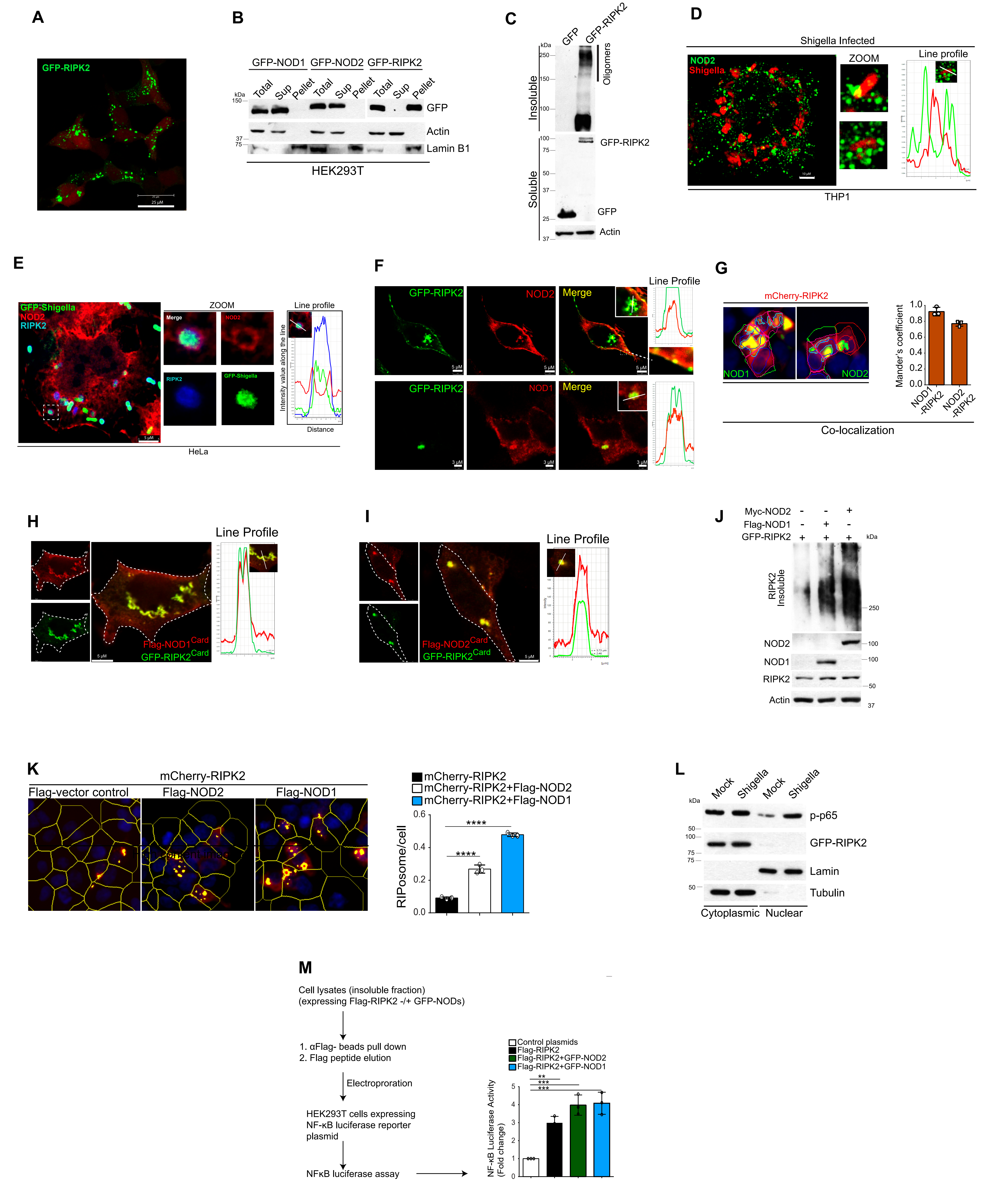

Appendix Fig S2. RIPK2 oligomers provide a platform for NODo-RIPosome formation.

(A) Representative confocal image of HEK293T transfected with GFP-RIPK2 plasmid (9 h). Scale bar, 25 μm.

(**B)** HEK293T cells transfected with GFP-NOD1 or GFP-NOD2 or GFP-RIPK2 were subjected to western blot analysis with total cellular extract (Total), soluble (Sup), and insoluble (pellet) fractions.

(C) Western blot analysis of soluble and insoluble fractions of HEK293T cells transfected with GFP-RIPK2 (9 h).

(D) Representative confocal images of THP-1 cells infected with RFP- labelled *S. flexneri* (8 hpi) and immuno-stained with NOD2 antibody. Zoom panels are digital magnification. Line profile: co-localization analysis using line intensity profile. Scale bar, 10 μm.

(E) Representative confocal images of HeLa cells transfected with myc-NOD2 for 4 h followed by infection with GFP-*S. flexneri* (MOI 1:25, 4 hpi). The cells were immuno-stained with c-Myc and RIPK2 antibodies. Zoom panels are digital magnification. Line profile: co-localization analysis using line intensity profile. Scale bar, 5 μm.

(F) Representative confocal images of HEK293T cells transfected with **(Upper panel)** GFP-RIPK2 and Flag-NOD2, Scale bar, 5 μm or **(Lower panel**) GFP-RIPK2 and Flag-NOD1. Line profile: co-localization analysis using line intensity profile. Scale bar, 3 μm.

(G) Right panel, representative high-content microscopy images (digitally zoomed; green masks for Flag-NOD1 or Flag-NOD2 and red masks for mCherry-RIPK2, blue mask for DAPI, cyan mask for co-localization, software algorithms-defined boundaries) of HEK293T cells transfected with mcherry-RIPK2 and GFP-NOD1 or GFP-NOD2 plasmids. About 17000 cells plated per well in a 96-well plate and NODo-RIPosomes were screened in 35 fields per well in 3 technical replicates. The graph depicts Mander's overlap (co-localization) coefficient. (n=3 biological replicates, Mean ± SD).

(H-I) Representative confocal images of HEK293T cells transfected with (**H)** GFP-RIPK2^{CARD} and Flag-NOD1^{CARD} or **(I)** GFP-RIPK2^{CARD} and Flag-NOD2^{CARD}. Line profile: co-localization analysis using line intensity profile. Scale bar, 5 μm.

(J) HEK293T cells transfected with GFP-RIPK2 and Flag-NOD1 or myc-NOD2 for 9 h. The triton X-100 soluble and insoluble fraction subjected to western blot analysis with indicated antibodies.

(K) Representative high-content microscopy image (yellow masks, software algorithms-defined cell boundaries) of RIPosomes in HEK293T cells transfected with mcherry-RIPK2 and Flag-NOD1 or Flag-NOD2 plasmid. About 17000 cells plated per well and were screened in 35 fields per well in 3 technical replicates. The graph depicts the average number of RIPosomes/cell. Mean ± SD. ****$p < 0.00005$, ordinary one-way ANOVA (Tukey's multiple comparisons test)**.**

(L) Cytoplasmic and nuclear fraction of *S. flexneri* infected (MOI 1:25, 4h) GFP-RIPK2 expressing HeLa cells subjected to western blot analysis with indicated antibodies

(M) Luciferase assay performed with the cell lysate of HEK293T cells transfected with NF-κB luciferase reporter vector and electroporated with purified RIPosomes (from insoluble fractions) with or without NOD1 or NOD2 and. Mean ± SD, n=3. **$p < 0.005$ and ***$p < 0.0005$, ordinary one-way ANOVA (Tukey's multiple comparisons test).

Appendix Fig S3. IRGM interacts and co-localizes with NODs, RIPK2, and RIPosomes.

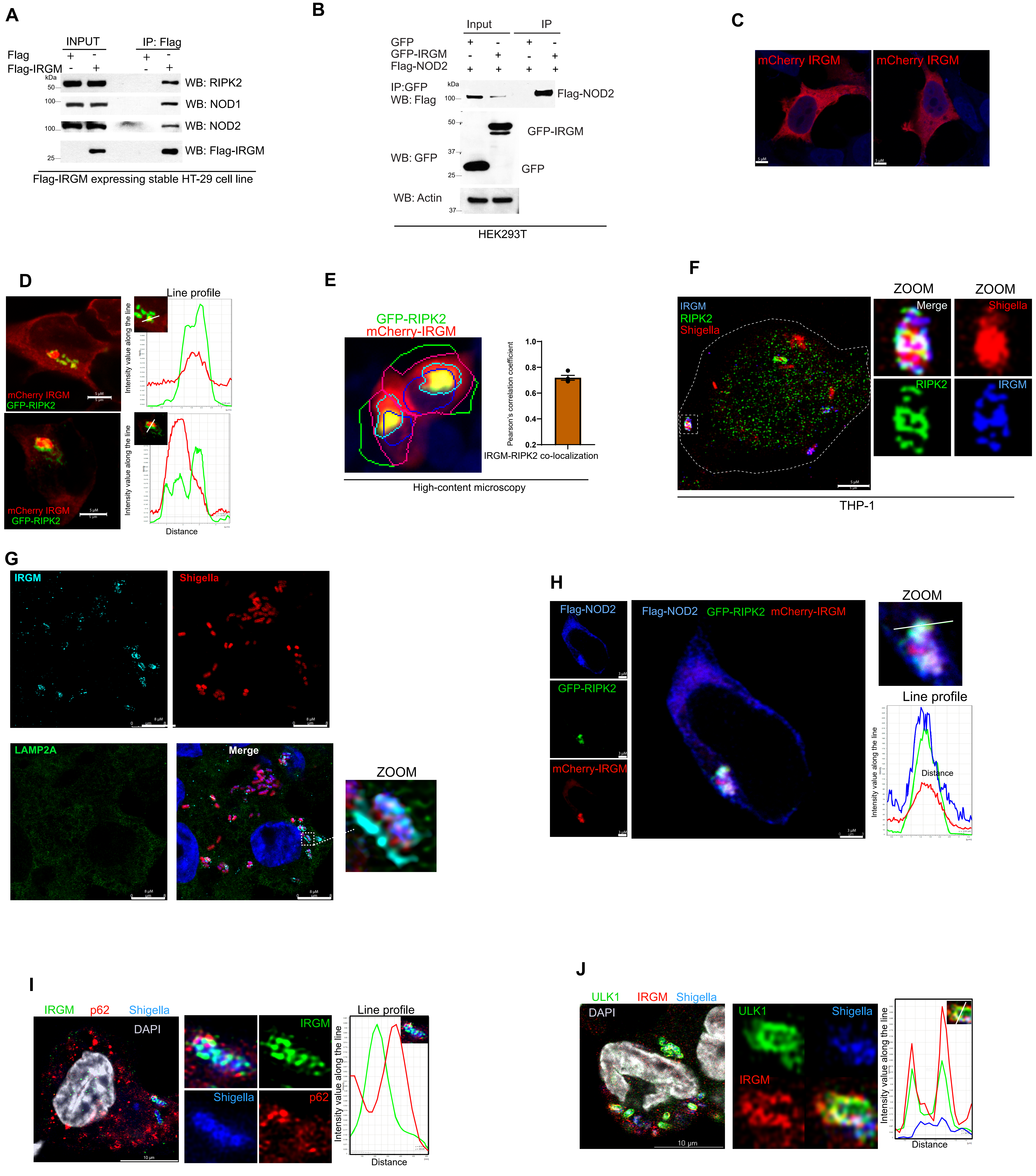

Appendix Fig S3. IRGM interacts and co-localizes with NODs, RIPK2, and RIPosomes.

(A) The lysates of HT-29 cells stably expressing control vector or Flag-IRGM were subjected to immunoprecipitation assay with Flag antibody and western blots are performed with indicated antibodies.

(B) Co-immunoprecipitation analysis using GFP antibody with lysates of HEK293T cell expressing Flag-NOD2 and GFP-IRGM.

(C-D) Representative confocal images of HEK293T cells transiently expressing **(C)** mCherry-IRGM or (**D)** mCherry-IRGM and GFP-RIPK2. Zoom panel is a digital magnification. Line profile: co-localization analysis using line intensity profile. Scale bar, 5 µm.

(E) Left panel, representative high-content microscopy image of HEK293T cells (green masks for GFP-RIPK2, red mask for mCherry-IRGM, and cyan mask for co-localization, software algorithms-defined cell boundaries) transfected with GFP-RIPK2 and mCherry-IRGM. About 17000 cells were plated per well in a 96-well plate and 35 fields per well were screened (n=4, biological replicates). The graph depicts Pearson's correlation coefficient.

(F) Representative confocal images of THP-1 cells infected with RFP expressing *S. flexneri* (MOI 1:25, 8 hpi) and immunostained with RIPK2 and IRGM antibodies. Zoom panels are digital magnification. Scale bar, 5 µm

(G) Representative confocal images of THP-1 cells infected with *S. flexneri* (MOI 1:25, 20 min) and immunostained with LAMP2A and IRGM antibodies. Zoom panels are digital magnification. Scale bar, 8µm

(H) Representative confocal images of HEK293T cells transfected with Flag-NOD2, GFP-RIPK2, and mCherry-IRGM. Zoom panels are digital magnification. Line profile: co-localization analysis using line intensity profile. Scale bar, 3 µm.

(I-J) Representative confocal images of THP-1 cells infected with *S. flexneri* (8 hpi) and immunostained with (**I)** p62 and IRGM antibodies or (**J)** ULK1 and IRGM antibodies**.** Zoom panels are digital magnification. Line profile: co-localization analysis using line intensity profile. Scale bar, 10 µm. (RFP-*Shigella* is pseudo colored to blue and p62 (panel **I**) or IRGM (panel J) are pseudo colored to red for better visualization of co-localization/juxtaposition, DAPI is pseudo colored to grey)

Appendix Fig S4. IRGM mediates degradation of NODs, RIPK2 and RIPosomes

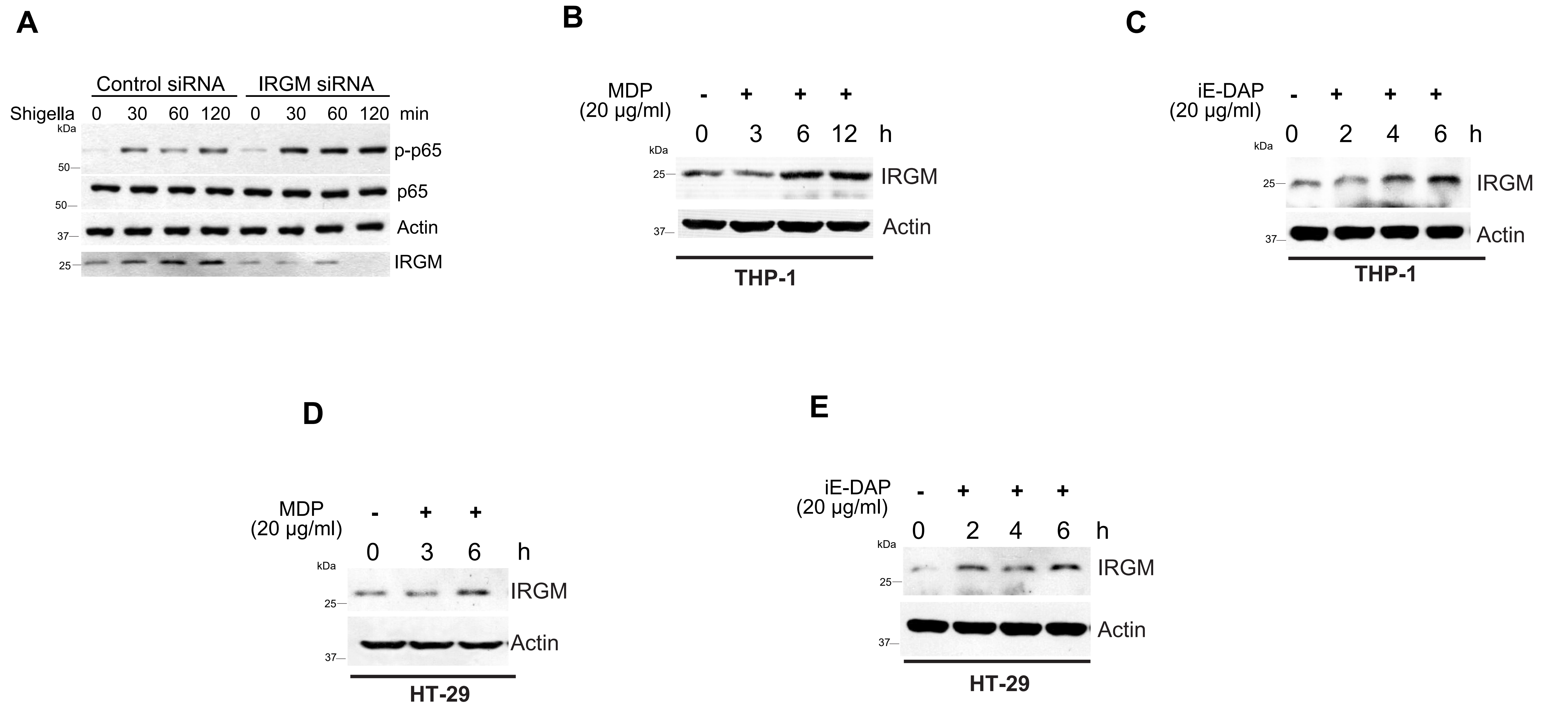

Appendix Fig S4. **IRGM mediates degradation of NODs, RIPK2 and RIPosomes.**

(**A**) Western blot analysis with the cell lysate of control and IRGM knockdown THP-1 cells infected with S. flexneri (MOI 1:25) for indicated time points and probe with indicated antibodies.
(**B-C**) Western blot analysis with the cell lysate of THP-1 cells treated with (B) MDP (20 µg/ml) or (C) iE-DAP (20 µg/ml) for indicated time points and probe with indicated antibodies.
(**D-E)** Western blot analysis with the cell lysate of HT-29 cells treated with (D) MDP (20 µg/ml) or (E) iE-DAP (20 µg/ml) for indicated time points and probe with indicated antibodies.

Appendix Figure S5. IRGM and p62 cooperate to mediate autophagic degradation of NODs, RIPK2, and RIPosomes

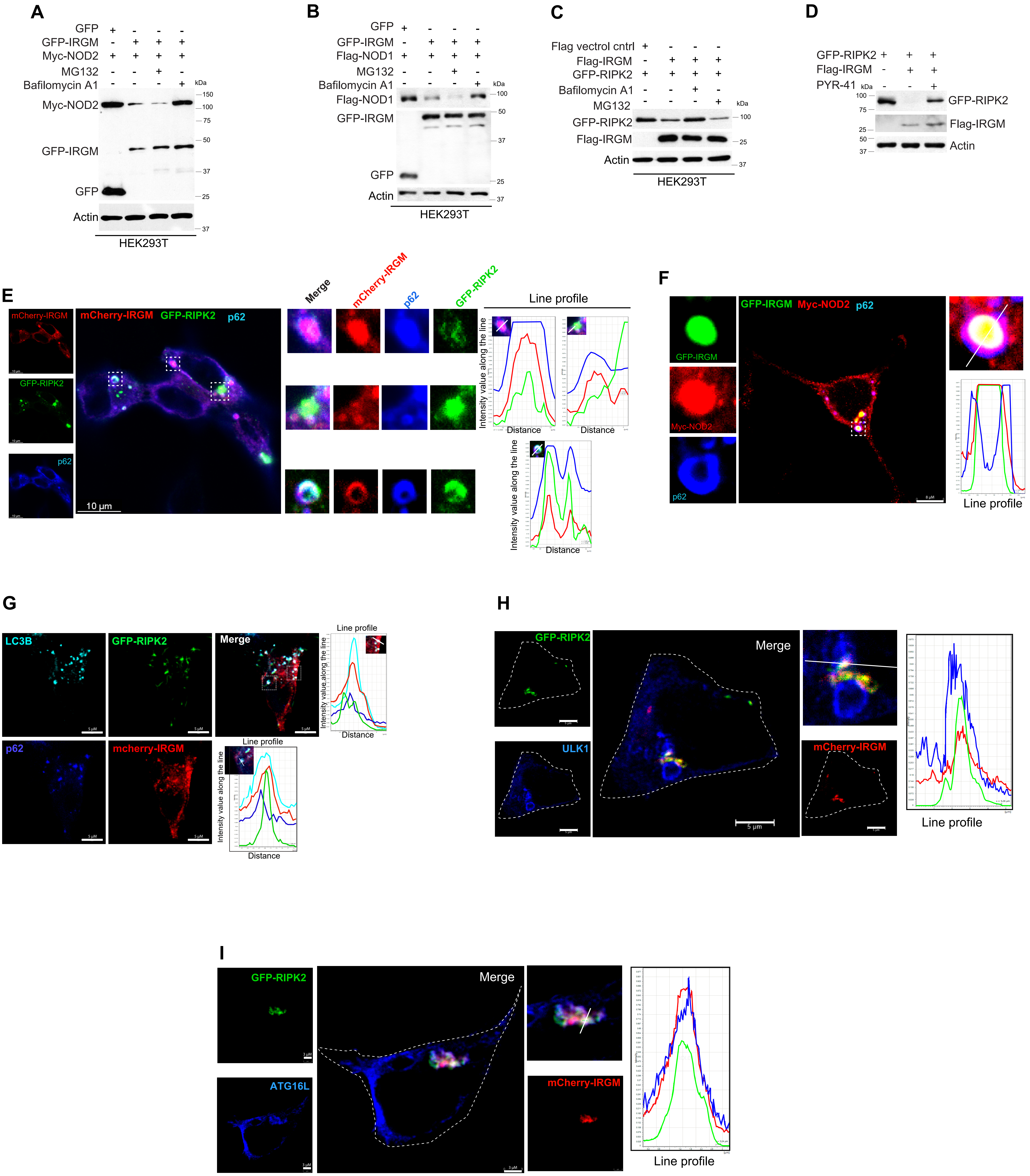

Appendix Fig S5. IRGM and p62 cooperates to mediate autophagic degradation of NODs, RIPK2 and RIPosomes.

(A-C) The cell lysate of HEK293T cells transfected with indicated plasmids and treated with MG132 (20 μM, 5 h) or Bafilomycin A1 (300 nM, 5 h) were subjected to immunoblot analysis.

(D) Western blot analysis with the lysate of HEK93T cells transiently transfected with GFP-RIPK2 in the presence and absence of Flag-IRGM for 4 h followed by treatment with PYR-41 (10 μM) for 5 h.

(E-F) Representative confocal images of HEK293T cells transfected with (**E)** GFP-RIPK2 and mCherry-IRGM, or (**F)** GFP-IRGM and myc-NOD2 immuno-stained with p62 antibody. Line profile: co-localization analysis using line intensity profile. Zoom panels are digital magnifications.

(G) Representative confocal images of HEK293T cells transfected with GFP-RIPK2 and mCherry-IRGM for 9h and immunostained with LC3b and p62.

(H-I) Representative confocal images of HEK293T cells transiently transfected with (**H**) GFP-RIPK2 and myc-ULK1 or (**I)** GFP-RIPK2 and Flag-ATG16L, Scale bar 5. Line profile: co-localization analysis using line intensity profile. Zoom panels are digital magnifications.

Appendix Table S1

Primers	Sequences (5'- 3')
Human IL-1β-Forward	AAGCTTGGTGATGTCTGGTC
Human IL-1β-Reverse	ACAAAGGACATGGAGAACACC
Human TNF-α Forward	ACTTTGGAGTGATCGGCC
Human TNF-α Reverse	AACATGGGCTACAGGCTTG
Human IL-1A Forward	TGTATGTGACTGCCCAAGATG
Human IL-1A Reverse	TTAGTGCCGTGAGTTTCCC
Human IL17C Forward	GAGGTGTTGGAGGCAGAC
Human IL17C Reverse	CAGCTTCTGTGGATAGCGG
Human TNFSF10 Forward	GTCTCTCTGTGTGGCTGTAAC
Human TNFSF10 Reverse	GGGTCCCAATAACTGTCATCTTC
Human CXCL-2 Forward	AACCGAAGTCATAGCCACAC
Human CXCL-2 Reverse	CTTCTGGTCAGTTGGATTTGC
Human CXCL-6 Forward	GTTTGTCTGGACCCGGAAG
Human CXCL-6 Reverse	GTCCAGGGATCTCCAGAAAAC
Human CXCL-10 Forward	CTCCAGTCTCAGCACCATG
Human CXCL-10 Reverse	CAGGTACAGCGTACAGTTCTAG
Human IFIT1 Forward	TGAAGCCCTGGAGTACTATGAG
Human IFIT1 Reverse	AAAGTGGCTGATATCTGGGTG
Human MX1 Forward	CCAGTAATGTGGACATCGCC
Human MX1 Reverse	CTTGTCTTCAGTTCCTTTGTCC
Human OAS2 Forward	GAATACCTGAAGCCCTACGAAG
Human OAS2 Reverse	GACTGTTTTCCGTCCATAGGAG
Human TRIM22 Forward	GCACGCTCATCTCAGATCTC
Human TRIM22 Reverse	TCAATGTCCAGCTTTCACTCC
Human RSAD2/VIPERIN Forward	GAAGAGGACATGACGGAACAG
Human RSAD2/VIPERIN Reverse	CAATTAAGAGGCACTGGAACAC
Human β- Actin Forward	GAGCACAGAGCCTCGCCTTT
Human β- Actin Reverse	ACATGCCGGAGCCGTTGTC
Mouse TNF-α Forward	ACGGACCCCAAAAGATGAAG
Mouse TNF-α Reverse	TTCTCCACAGCCACAATGAG
Mouse IL-1β Forward	GGAACTGGCAGAAGAGGCACTC
Mouse IL-1β Reverse	GCAGGAATGAGAAGAGGCTGAGAC
Mouse IL-1A Forward	CCACTTGGTTAAATGACCTGC
Mouse IL-1A Reverse	GCTCACGAACAGTTGTGAATC
Mouse IFIT1 Forward	AGAGTCAAGGCAGGTTTCTG
Mouse IFIT1 Reverse	AAGCAGATTCTCCATGACCTG
Mouse MX1 Forward	AGGCAGTGGTATTGTCACCA
Mouse MX1 Reverse	AGACTTTGCCTCTCCACTCC
Mouse ISG15 Forward	GAGAGCAAGCAGCCAGAAG
Mouse ISG15 Reverse	CCCAGGCCATTGCTGCAGGC
