# Supplementary material for: Selective autophagy of RIPosomes maintains innate immune homeostasis during bacterial infection
Source: EMBO J. 2022 Oct 11;41(23):e111289. doi: 10.15252/embj.2022111289 (PMC9713718; doi:10.15252/embj.2022111289)
Supplement: Supplementary file 2 — Expanded View Figures PDF [file EMBJ-41-e111289-s016.pdf]

Expanded View Figures

Figure EV1. NODs, RIPK2, and RIPosomes are the target of selective autophagy.

A–C Western blot analysis with the cell lysates of HT-29 cells treated with cycloheximide (100 μg/ml) alone or in combination with Bafilomycin A1 (300 nM) or MG132 (20 μM) for different time points as indicated.

D Western blot analysis of IP experiments performed with lysates of HEK293T cells transiently transfected with Flag-RIPK2 and HA-K63-Ubiquitin [variants of ubiquitin that can only be ubiquitinated at lysine 63 (K63)] and infected with *S. flexneri*, (MOI: 1:25, 6 h). IP was performed with Flag antibody and Western blotting was performed with indicated antibodies.

E Representative confocal images of THP-1 cells infected with RFP expressing *S. flexneri*, (MOI: 1:25, 8 h) and immunostained with anti-RIPK2 and anti-FK2 antibodies. Line profile: co-localization analysis using line intensity profiles. Scale bar, 5 μm.

F Left panel, representative high-content microscopy images (digitally zoomed) of RIPosomes in HEK293T cells. The HEK293T cells were transfected with GFP RIPK2 (100 ng/well of 96-well plates) for 4 h followed by treatment with rapamycin (500 nM, 4 h), or rapamycin (500 nM, 4 h) and PYR-41 (5 μM) for 5 h. Right panel, the graph depicts an average number of RIPosomes/cell. About 15,000 cells were plated per well and RIPosomes were screened in 35 fields per well. Mean ± SD, $n = 3$ (biological replicates), ***$P < 0.0005$, ****$P < 0.00005$, ordinary one-way ANOVA (Tukey's multiple comparisons test).

G Left panels, representative high-content microscopy images (digitally zoomed) of control or p62 knockdown THP-1 cells infected with *S. flexneri* (6 hpi). About 50,000 cells were plated per well in a 96-well plate and RIPosomes were screened in 35 fields per well. Right panel, the graph depicts the average number of RIPosomes/cell, which is calculated from three biological replicates. Mean ± SD. ***$P < 0.0005$, Student's unpaired *t*-test.

H Representative confocal images of HEK293T cells transfected with GFP-RIPK2 (6 h) and immunostained with p62 and LC3B antibody. Zoom panel is a digital magnification. Line profile: co-localization analysis using line intensity profile. Scale bar, 5 μm.

I Representative confocal images of THP-1 cells infected with RFP expressing *S. flexneri*, (MOI 1:25) and immunostained with RIPK2 and LC3B antibodies. DNA stained with DAPI. Scale bar, 8 μm. Line profile: co-localization analysis using line intensity profile. Zoom panels are digital magnifications.

J, K Representative confocal images of HEK293T cells transfected with (I) GFP-RIPK2 and Myc-ULK1 (6 h) and (J) GFP-RIPK2 and Flag-ATG16L (6 h). Zoom panels are digital magnification. Line profile: co-localization analysis using line intensity profile. Scale bar, 5 μm.

L Western blot analysis with the cell lysate of PMA-treated THP-1 cells infected with *S. flexneri*, (MOI: 1:25) with or with Bafilomycin A1 (300 nM) for different time points as indicated.

M The qRT–PCR analysis with total RNA isolated from the uninfected and *S. flexneri* (MOI 1:2.5, 4 h) infected control or p62 knockdown or p62 and RIPK2 double knockdown THP-1 cells. Mean ± SD, $n = 4$ (biological replicates), ***$P < 0.0005$ and ****$P < 0.00005$, ordinary one-way ANOVA (Tukey's multiple comparisons test).

Source data are available online for this figure.

© 2022 The Authors

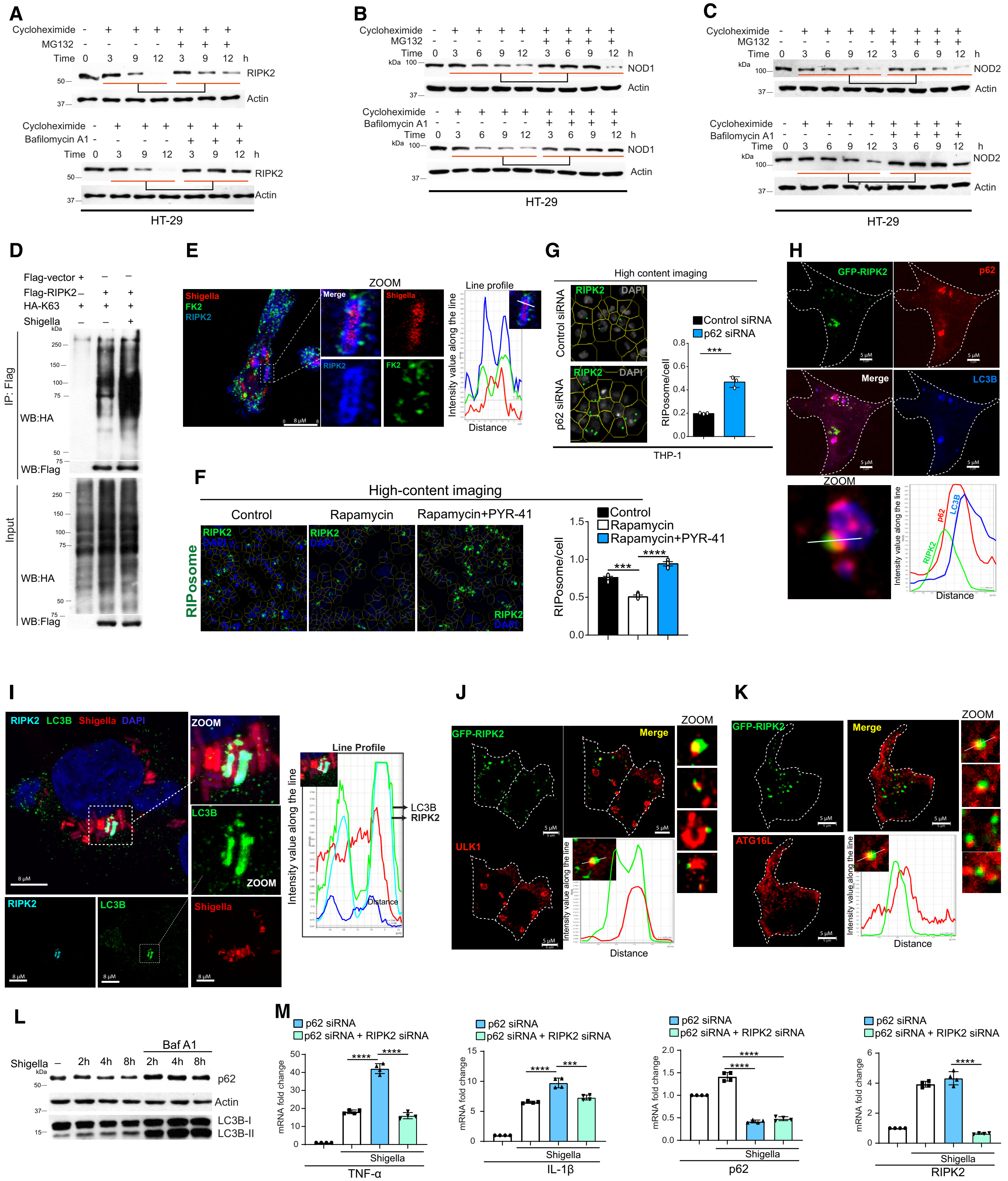

A
B
C
D
E
F
G
H
I
J
K
L
M
Cycloheximide
MG132
Bafilomycin A1
Time
RIPK2
NOD1
NOD2
Actin
HT-29
Flag-vector
Flag-RIPK2
HA-K63
Shigella
IP: Flag
WB:HA
WB:Flag
Input
ZOOM
Merge
FK2
Line profile
Intensity value along the line
Distance
High content imaging
Control siRNA
p62 siRNA
RIPK2
DAPI
RIPosome/cell
THP-1
High-content imaging
Control
Rapamycin
Rapamycin+PYR-41
RIPosome
GFP-RIPK2
p62
LC3B
Line Profile
ULK1
ATG16L
Baf A1
LC3B-I
LC3B-II
p62 siRNA + RIPK2 siRNA
mRNA fold change
TNF-α
IL-1β

Figure EV1.

Figure EV2. IRGM mediates the degradation of NODs, RIPK2, and RIPosomes.

A The cell lysates of *S. flexneri*-infected IRGM$^{+/+}$ and IRGM$^{+/-}$ HT-29 cells were subjected to Western blot analysis with indicated antibodies. Densitometric analysis was performed using Image J software. FC, fold change.

B Western blot analysis with the cell lysates of control and IRGM siRNA transfected doxycycline (1 μg/ml) induced HeLa GFP-RIPK2 stable cells infected with S. *flexneri* (MOI 1:25, 8 h). Densitometric analysis was performed using Image J software. FC, fold change.

C, D Western blot analysis with the cell lysates of HEK293T transfected with indicated plasmids. Densitometric analysis was performed using Image J software. FC, fold change.

E Western blot analysis with the cell lysate of HEK293T cells transfected Myc-NOD2 and GFP or increasing concentration of GFP-IRGM (2, 4, and 6 μg) plasmids for 12 h.

F Left panels, representative high-content microscopy images (Green masks, software algorithms-defined cell boundaries, digitally zoomed images) of HEK293T cells transfected with GFP-RIPK2 or GFP-RIPK2 and Flag-IRGM. About 17,000 cells were plated per well in a 96-well plate and RIPosomes were screened in 35 fields per well. Right panel, the graph depicts the average number of RIPosomes/cell, which is calculated from four biological replicates, Mean ± SD. ****$P < 0.00005$, Student's unpaired *t*-test.

G Western blot analysis of soluble and insoluble fractions of HEK293T cells transfected with indicated plasmids for 12 h.

H Representative confocal image of HEK293T cells transfected with GFP-RIPK2^{CARD} or GFP-RIPK2^{CARD} and mCherry-IRGM. Scale bar, 5 μm.

I Right panels, representative high-content microscopy images (Yellow masks, software algorithms-defined cell boundaries, digitally zoomed images) of HEK293T cells transfected with mCherry-RIPK2$^{CARD.}$ Alone or with Flag-IRGM. About 17,000 cells were plated per well in a 96-well plate and RIPK2^{CARD} spots were screened in 35 fields per well. Left panel, the graph depicts an average number of RIPK2^{CARD} spots/cell, which is calculated from three biological replicates, Mean ± SD. ****$P < 0.00005$, Student's unpaired *t*-test.

J Western blot analysis with the cell lysates of HEK293T transfected with indicated plasmids for 12 h.

K Representative fluorescence microscopy images of HEK293T cells transfected with GFP-RIPK2 ^{CARD} alone or with Flag-IRGM or with catalytic mutant Flag-IRGM (S47N) for 12 h. Scale bar, 150 μm.

L Assessment of NF-κB-induced SEAP (secreted embryonic alkaline phosphatase) activity in the cell culture supernatant of HEK-Blue™ hNOD2 cells (InvivoGen) transfected with plasmids as indicated and treated with L18-MDP (100 ng/ml, 24 h) as indicated. Three technical replicates, Mean ± SD. **$P < 0.005$, ****$P < 0.00005$, Student's unpaired *t*-test.

Source data are available online for this figure.

© 2022 The Authors

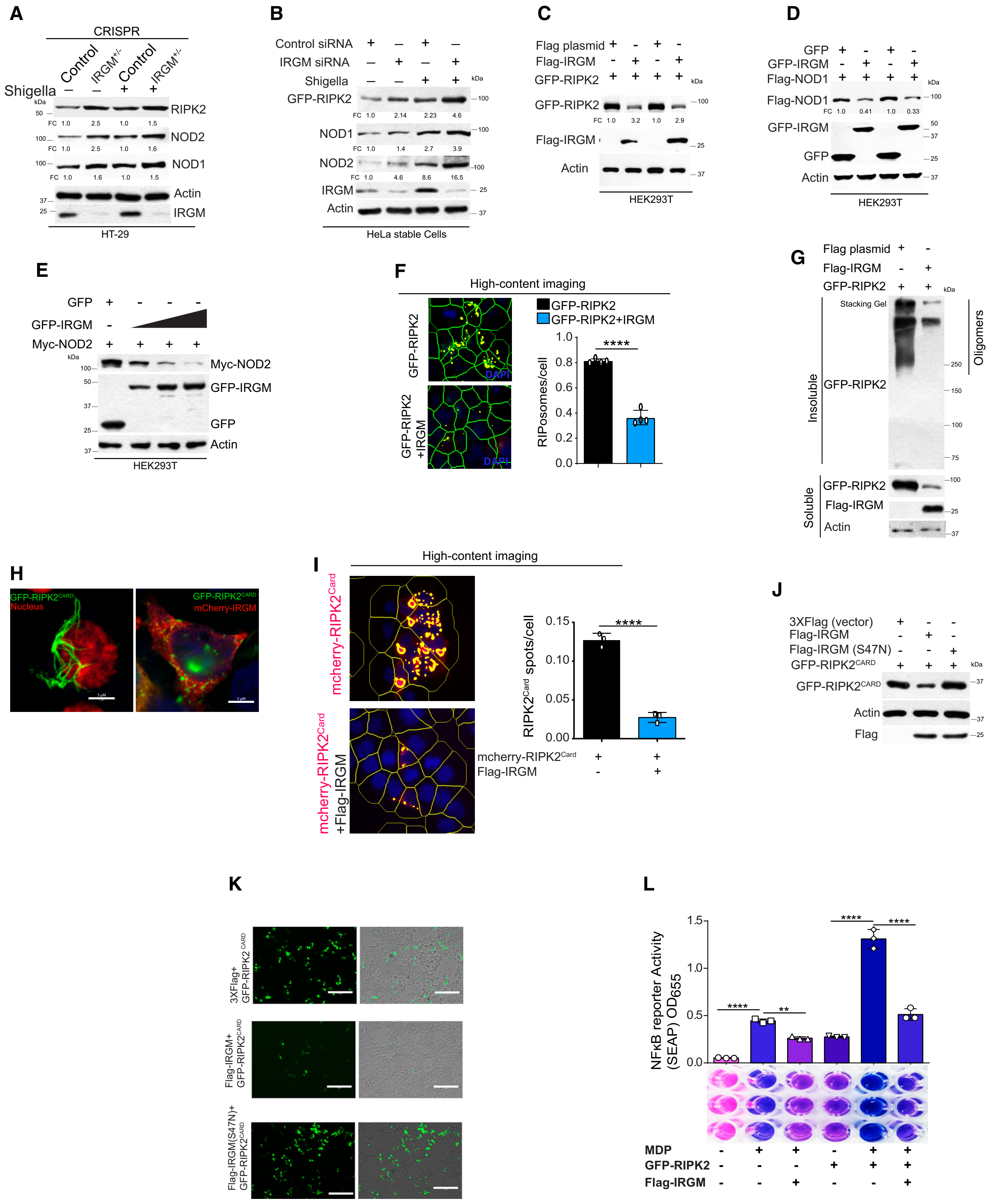
A
CRISPR
Control
IRGM+/-
Control
IRGM+/-
Shigella
RIPK2
NOD2
NOD1
Actin
IRGM
HT-29
B
Control siRNA
IRGM siRNA
Shigella
GFP-RIPK2
NOD1
NOD2
IRGM
Actin
HeLa stable Cells
C
Flag plasmid
Flag-IRGM
GFP-RIPK2
GFP-RIPK2
Flag-IRGM
Actin
HEK293T
D
GFP
GFP-IRGM
Flag-NOD1
Flag-NOD1
GFP-IRGM
GFP
Actin
HEK293T
E
GFP
GFP-IRGM
Myc-NOD2
Myc-NOD2
GFP-IRGM
GFP
Actin
HEK293T
F
High-content imaging
GFP-RIPK2
GFP-RIPK2+IRGM
RIPosomes/cell
G
Flag plasmid
Flag-IRGM
GFP-RIPK2
Stacking Gel
Oligomers
Insoluble
Soluble
GFP-RIPK2
Flag-IRGM
Actin
H
GFP-RIPK2CARD
Nucleus
mCherry-IRGM
I
High-content imaging
mcherry-RIPK2Card
mcherry-RIPK2Card +Flag-IRGM
RIPK2Card spots/cell
Flag-IRGM
J
3XFlag (vector)
Flag-IRGM
Flag-IRGM (S47N)
GFP-RIPK2CARD
Actin
Flag
K
3XFlag+ GFP-RIPK2CARD
Flag-IRGM+ GFP-RIPK2CARD
Flag-IRGM(S47N)+ GFP-RIPK2CARD
L
NFκB reporter Activity (SEAP) OD655
MDP
GFP-RIPK2
Flag-IRGM

Figure EV2.

Figure EV3. IRGM and p62 cooperatively execute selective autophagy of NODs, RIPK2, and RIPosomes.

A, B Western blot analysis with cell lysates of control cells and ATG5 knockdown THP-1 cells transiently transfected with GFP-IRGM plasmids and treated with (A) MDP (40 μg/ml, 4 h) or (B) iE-DAP (40 μg/ml, 4 h).

C–E The control and ATG5 knockdown HEK293T cells were transfected with indicated plasmids and cell lysates were subjected to immunoblot analysis.

F Left panels, representative high-content microscopy images (digitally zoomed) of cells that were knockdown for indicated genes and transfected with plasmids as indicated. In the last panel, GFP-RIPK2 and mcherry-IRGM transfected cells were treated with Bafilomycin A1 (300 nM, 5 h). Right panel, the graph depicts an average number of RIPosomes/cell. About 50,000 cells were plated per well and RIPosomes were screened in 35 fields per well. Mean ± SD, $n = 6$ (biological replicates), $^{****}P < 0.00005$, ordinary one-way ANOVA (Tukey's multiple comparisons test).

G Representative confocal images of cells transfected with GFP-RIPK2, and mCherry-IRGM and immunostained with p62. Line profile: co-localization analysis using line intensity profiles. Scale bar, 5.5 μm.

H Sequential immunoprecipitation assay from the lysate where the HEK293T transiently transfected with Flag-RIPK2 (or Flag-vector control), HA-p62, and, GFP-IRGM for 12 h. The first immunoprecipitation was performed with Flag antibody followed by elution with flag peptide. The flag peptide eluted samples were further subjected to a second IP with anti-GFP (for IRGM) and probed with indicated antibodies.

I–K The control and p62 knockdown HEK293T cells were transfected with indicated plasmids and cell lysates were subjected to Western blot analysis with indicated antibodies.

L, M Western blot analysis with cell lysates of control cells and p62 knockdown THP-1 cells transiently transfected with GFP-IRGM plasmids treated with (L) MDP (40 μg/ml, 4 h) or (M) iE-DAP (40 μg/ml, 4 h).

N Immunoprecipitation analysis of the interaction between endogenous RIPK2 and endogenous p62 in the lysate of *S. flexneri* (MOI 1:25, 6 h) infected control and IRGM knockdown THP-1 cells. IgG, IgG heavy chain.

O–Q Co-IP analysis with the lysate of HEK293T cells transiently transfected with (O) Flag-NOD1 and HA-p62, (P) Flag-NOD2 and HA-p62, and, (Q) GFP-RIPK2 and HA-p62 in the presence and absence of IRGM (GFP or Flag) or vector controls.

R Pictorial representation of data. We found that IRGM and p62 coordinate selective autophagy of NODs, RIPK2, and RIPosomes.

Source data are available online for this figure.

© 2022 The Authors

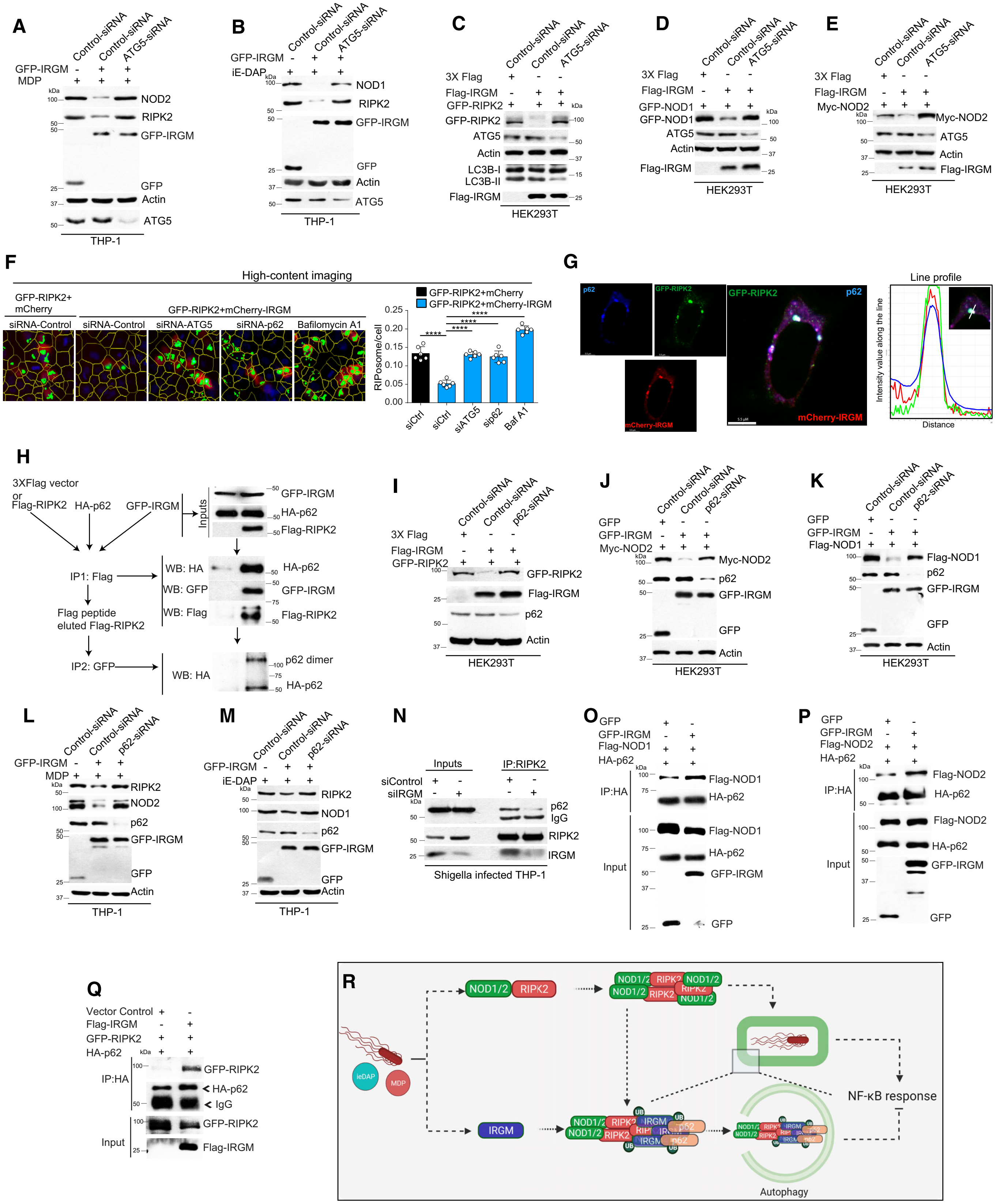
A
Control-siRNA
Control-siRNA
ATG5-siRNA
GFP-IRGM - + +
MDP + + +
kDa
100
NOD2
RIPK2
50
50
GFP-IRGM
25
GFP
37
Actin
50
ATG5
THP-1
B
GFP-IRGM - + +
iE-DAP + + +
NOD1
RIPK2
GFP-IRGM
GFP
Actin
ATG5
THP-1
C
3X Flag + - -
Flag-IRGM - + +
GFP-RIPK2 + + +
GFP-RIPK2
ATG5
Actin
LC3B-I
LC3B-II
Flag-IRGM
HEK293T
D
3X Flag + - -
Flag-IRGM - + +
GFP-NOD1 + + +
GFP-NOD1
ATG5
Actin
Flag-IRGM
HEK293T
E
3X Flag + - -
Flag-IRGM - + +
Myc-NOD2 + + +
Myc-NOD2
ATG5
Actin
Flag-IRGM
HEK293T
F
High-content imaging
GFP-RIPK2+ mCherry
GFP-RIPK2+mCherry-IRGM
siRNA-Control
siRNA-Control
siRNA-ATG5
siRNA-p62
Bafilomycin A1
GFP-RIPK2+mCherry
GFP-RIPK2+mCherry-IRGM
RIPosome/cell
siCtrl
siCtrl
siATG5
sip62
Baf A1
G
p62
GFP-RIPK2
mCherry-IRGM
Line profile
Intensity value along the line
Distance
H
3XFlag vector or Flag-RIPK2
HA-p62
GFP-IRGM
Inputs
GFP-IRGM
HA-p62
Flag-RIPK2
IP1: Flag
WB: HA
WB: GFP
WB: Flag
Flag peptide eluted Flag-RIPK2
IP2: GFP
p62 dimer
HA-p62
I
Control-siRNA
Control-siRNA
p62-siRNA
3X Flag + - -
Flag-IRGM - + +
GFP-RIPK2 + + +
GFP-RIPK2
Flag-IRGM
p62
Actin
HEK293T
J
GFP + - -
GFP-IRGM - + +
Myc-NOD2 + + +
Myc-NOD2
p62
GFP-IRGM
GFP
Actin
HEK293T
K
GFP + - -
GFP-IRGM - + +
Flag-NOD1 + + +
Flag-NOD1
p62
GFP-IRGM
GFP
Actin
HEK293T
L
GFP-IRGM - + +
MDP + + +
RIPK2
NOD2
p62
GFP-IRGM
GFP
Actin
THP-1
M
GFP-IRGM - + +
iE-DAP + + +
RIPK2
NOD1
p62
GFP-IRGM
GFP
Actin
THP-1
N
Inputs
IP:RIPK2
siControl + - + -
siIRGM - + - +
p62
IgG
RIPK2
IRGM
Shigella infected THP-1
O
GFP + -
GFP-IRGM - +
Flag-NOD1 + +
HA-p62 + +
IP:HA
Flag-NOD1
HA-p62
Input
Flag-NOD1
HA-p62
GFP-IRGM
GFP
P
GFP + -
GFP-IRGM - +
Flag-NOD2 + +
HA-p62 + +
IP:HA
Flag-NOD2
HA-p62
Input
Flag-NOD2
HA-p62
GFP-IRGM
GFP
Q
Vector Control + -
Flag-IRGM - +
GFP-RIPK2 + +
HA-p62 + +
IP:HA
GFP-RIPK2
HA-p62
IgG
Input
GFP-RIPK2
Flag-IRGM
R
NOD1/2
RIPK2
ieDAP
MDP
IRGM
p62
NF-κB response
Autophagy

Figure EV3.

Figure EV4. IRGM negatively regulates bacterial infection-induced programs of pro-inflammatory response.

A Heatmap of the gene clusters related to top biological pathways overrepresented in the Reactome pathway analysis. Reactome pathway analysis was performed with the set of differentially regulated genes (1.5-fold, $P < 0.05$, Wald Chi-Squared test, $n = 3$, biological replicates) in uninfected and *Salmonella*-infected control and IRGM shRNA knockdown HT-29 cells. The graph below the heat map shows box plot with median obtained from data scaled to Z-score.

B Left panel, heatmap depicts the gene set belonging to the "Cytokine signaling in immune system" pathway that is one of the top upregulated pathways in Reactome pathway analysis. Reactome pathway analysis was performed with the set of genes significantly upregulated (1.5-fold, $P < 0.05$, Wald test, $n = 3$) in *Salmonella typhimurium* infected IRGM shRNA knockdown HT-29 cells compared with control shRNA cells. Right panel, the bar graph represents the top 10 biological pathways upregulated in GO-based Reactome pathways analysis using a set of genes that are significantly (1.5-fold, $P < 0.05$, Wald Chi-Squared test, $n = 3$ in each group) induced in *Salmonella*-infected IRGM shRNA HT-29 as compared to *Salmonella*-infected control cells.

C Heatmap depicts the significantly upregulated (1.5-fold, $P < 0.05$, Wald Chi-Squared test, $n = 3$) NF-κB regulated cytokine and chemokine genes in *Salmonella*-infected IRGM shRNA HT-29 cells compared with *Salmonella*-infected control shRNA cells.

D A qRT–PCR validation of significantly upregulated cytokines and chemokines in RNA-Seq data with total RNA isolated from uninfected and *Salmonella*-infected control and IRGM shRNA HT-29 cells. Mean ± SD, $n = 3$ (Biological replicates). **$P < 0.05$, ***$P < 0.005$, ****$P < 0.0005$, Student's unpaired *t*-test.

E Left panel, Metascape pathway analysis with the set of genes that are significantly upregulated in IRGM$^{+/-}$ cells (compared with control; 1.5-fold, $P < 0.05$, Wald Chi-Squared test, $n = 3$) and at the same time were significantly rescued by RIPK2 depletion in IRGM$^{+/-}$ HT-29 cells ($P < 0.05$, Wald Chi-Squared test, 0.8, $n = 3$). Right panel, heatmap depicts the gene set belonging to "Hallmark TNFα signaling via NF-κB" pathway that is the top upregulated pathway in Metascape analysis. The graph below the heat map shows box plot with median obtained from data scaled to Z-score.

F Bar graph depicts top biological pathway upregulated in GO-based metascape pathway analysis with genes significantly induced (1.5-fold, *P* 0.05, $n = 3$) in *Shigella*-infected IRGM$^{+/-}$ HT-29 cells.

G TRRUST analysis (database for the study of the transcriptional regulation involved in human diseases) with the set of genes that are significantly upregulated in IRGM$^{+/-}$ cells (compared with control; 1.5-fold, $P < 0.05$, Wald Chi-Squared test, $n = 3$, biological replicates) and at the same time were significantly rescued by RIPK2 depletion in IRGM$^{+/-}$ HT-29 cells ($P < 0.05$, Wald Chi-Squared test, 0.8, $n = 3$).

Source data are available online for this figure.

 © 2022 The Authors

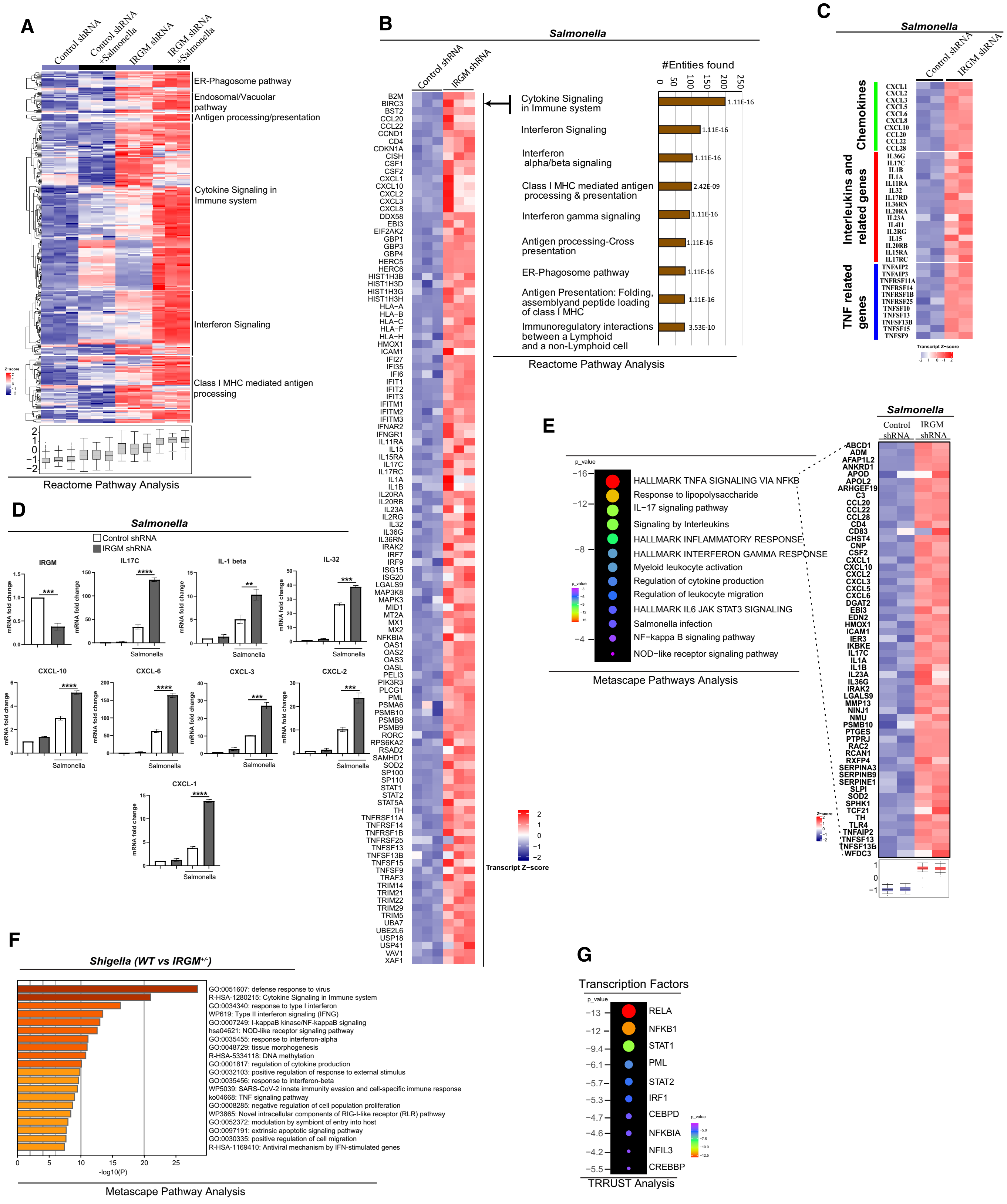

Figure EV4.

© 2022 The Authors

Figure EV5. RIPK2 inhibition ameliorates shigellosis and DSS-induced gut inflammation in *Irgm1* knockout mice.

A Right panel, representative picture of fecal samples collected from *S. flexneri*-infected *Irgm1*$^{+/+}$ or *Irgm1*$^{-/-}$ or GSK583-treated *Irgm1*$^{-/-}$ mice. Left panel, the graph depicts fecal pathology scores based on stool consistency and color *of S. flexneri*-infected and GSK583-treated C57BL/6 mice. (n = 6 mice in each group, Mean ± SD. $^{**}P < 0.005$, Student's unpaired t-test).

B The graph depicts the total stool scores (stool consistency + blood) of DSS-treated *Irgm1*$^{+/+}$ or *Irgm1*$^{-/-}$ or GSK583-treated *Irgm1*$^{-/-}$ mice.

C Right panel, representative picture of colons of DSS-treated *Irgm1*$^{+/+}$ or *Irgm1*$^{-/-}$ or GSK583-treated *Irgm1*$^{-/-}$ mice; Left panel, the graph depicts the average colon length of same. (n = 3 mice each group, Mean ± SD. $^{*}P < 0.05$, $^{**}P < 0.005$, Student's unpaired t-test).

D Representative microscopic images of hematoxylin–eosin (H&E) staining of colon tissue of DSS-treated *Irgm1*$^{+/+}$ or *Irgm1*$^{-/-}$ or GSK583-treated *Irgm1*$^{-/-}$ mice.

E Representative microscopic images of hematoxylin–eosin (H&E) staining of colon tissue of *S. flexneri*-infected *Irgm1*$^{+/+}$ or *Irgm1*$^{-/-}$ or GSK583-treated *Irgm1*$^{-/-}$ mice.

F The graph depicts the histopathology score (average pathological scores from HE staining based on hyperplasia, inflammatory cells infiltration, epithelial cell death, and loss of goblet cells) of *S. flexneri*-infected *Irgm1*$^{+/+}$ or *Irgm1*$^{-/-}$ or GSK583-treated *Irgm1*$^{-/-}$ mice. (n = 3 mice in each group, Mean ± SD, $^{**}P < 0.005$, Student's unpaired t-test).

G The qRT–PCR analysis for indicated genes with the total RNA isolated from the colons of DSS-treated *Irgm1*$^{+/+}$ or *Irgm1*$^{-/-}$ or GSK583-treated *Irgm1*$^{-/-}$ mice. (n = 3 mice in each group, Mean ± SD. $^{*}P < 0.05$. $^{**}P < 0.005$, $^{***}P < 0.0005$, $^{****}P < 0.00005$, ordinary one-way ANOVA (Tukey's multiple comparisons test).

Source data are available online for this figure.

© 2022 The Authors

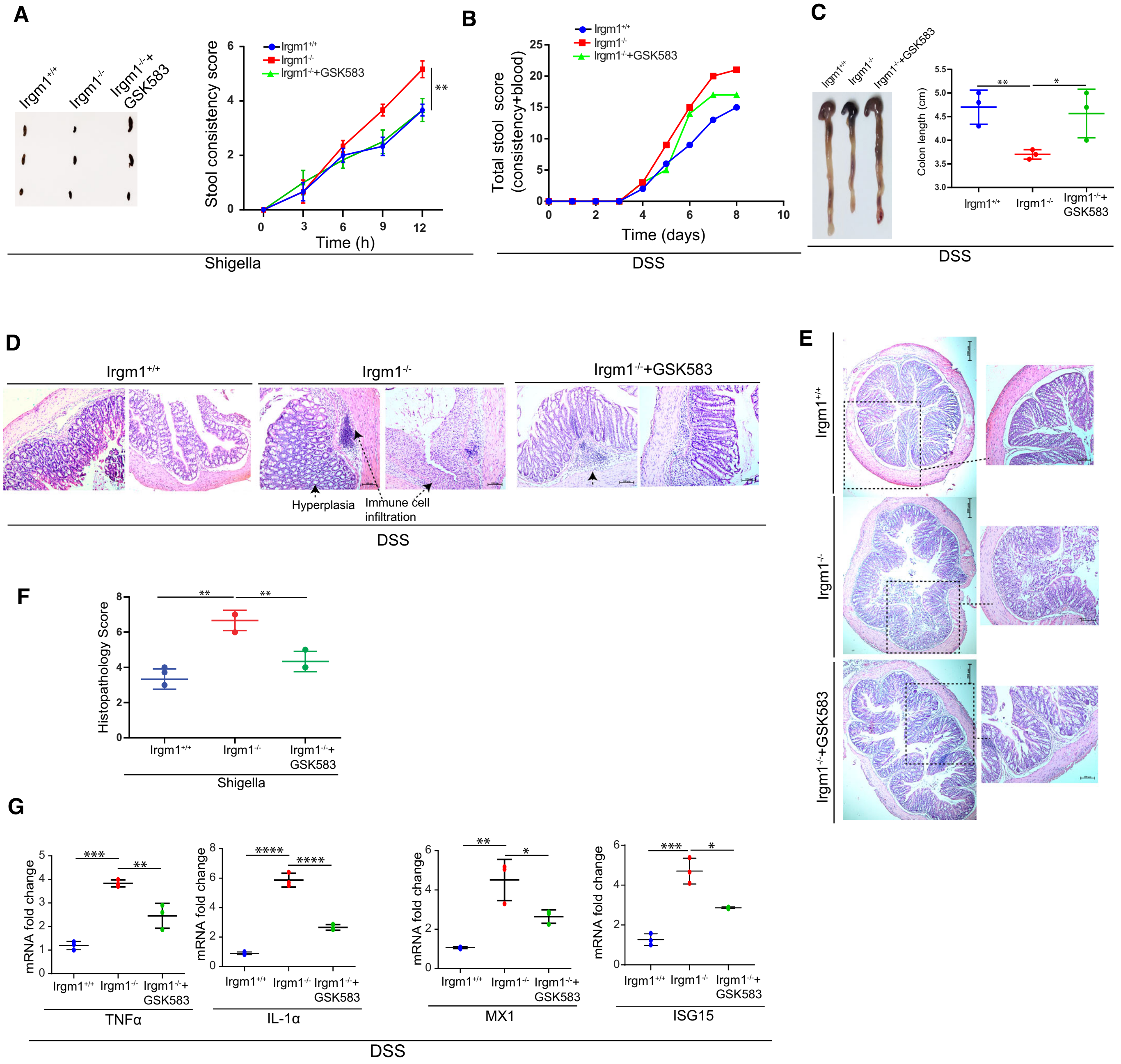
A
Irgm1+/+
Irgm1-/-
Irgm1-/-+ GSK583
Stool consistency score
Time (h)
Shigella
B
Total stool score (consistency+blood)
Time (days)
DSS
C
Colon length (cm)
DSS
D
Irgm1+/+
Irgm1-/-
Irgm1-/-+GSK583
Hyperplasia
Immune cell infiltration
DSS
E
F
Histopathology Score
Shigella
G
mRNA fold change
TNFα
IL-1α
MX1
ISG15
DSS

Figure EV5.
