# Supplementary figures and images for: Selective autophagy of RIPosomes maintains innate immune homeostasis during bacterial infection (part 1 of 2)
Source: EMBO J. 2022 Oct 11;41(23):e111289. doi: 10.15252/embj.2022111289 (PMC9713718; doi:10.15252/embj.2022111289)

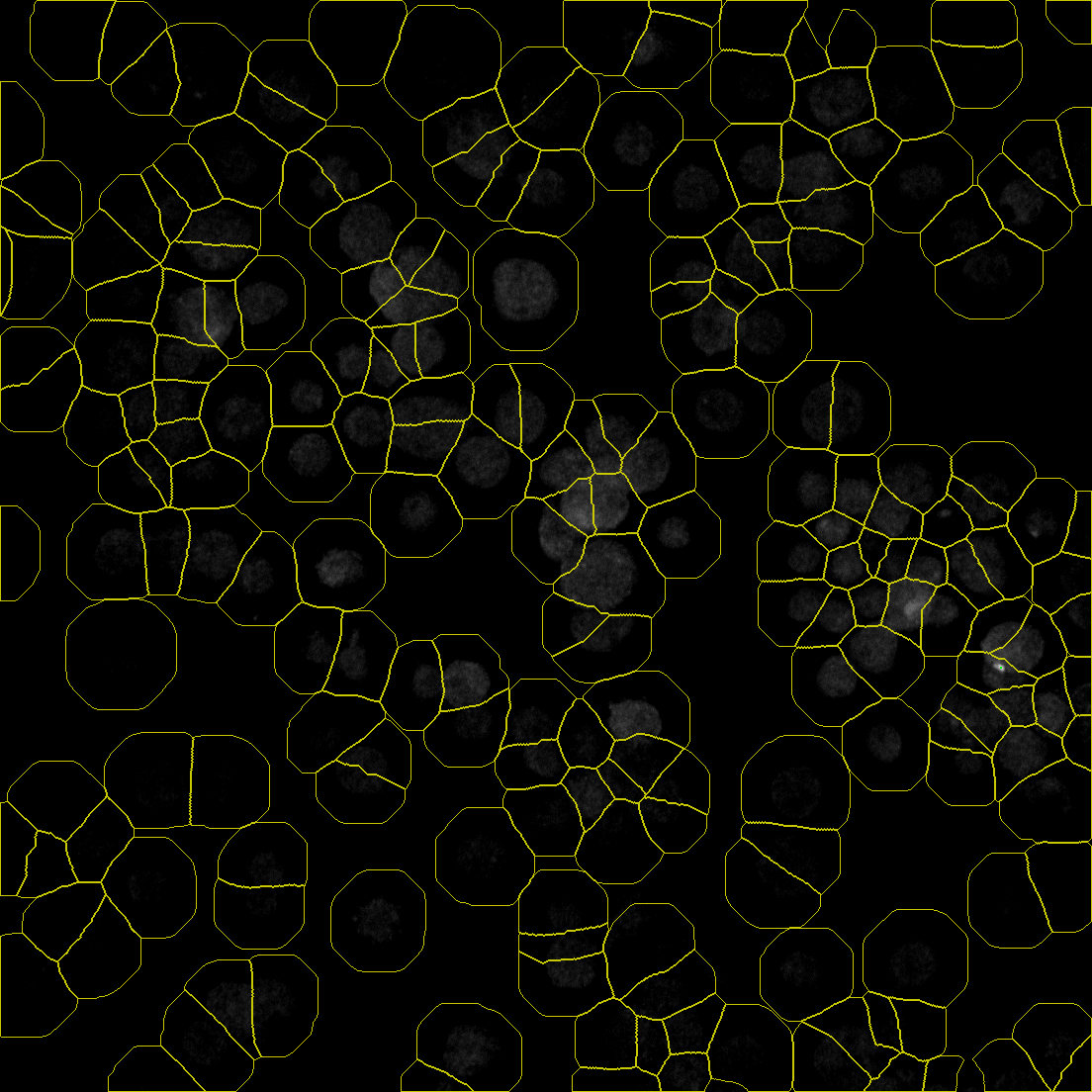

Supplement: Supplementary file 14 — Source Data for Figure 1 [file EMBJ-41-e111289-s020.zip › High Content Screening/1B/1B_CONTROL.bmp]

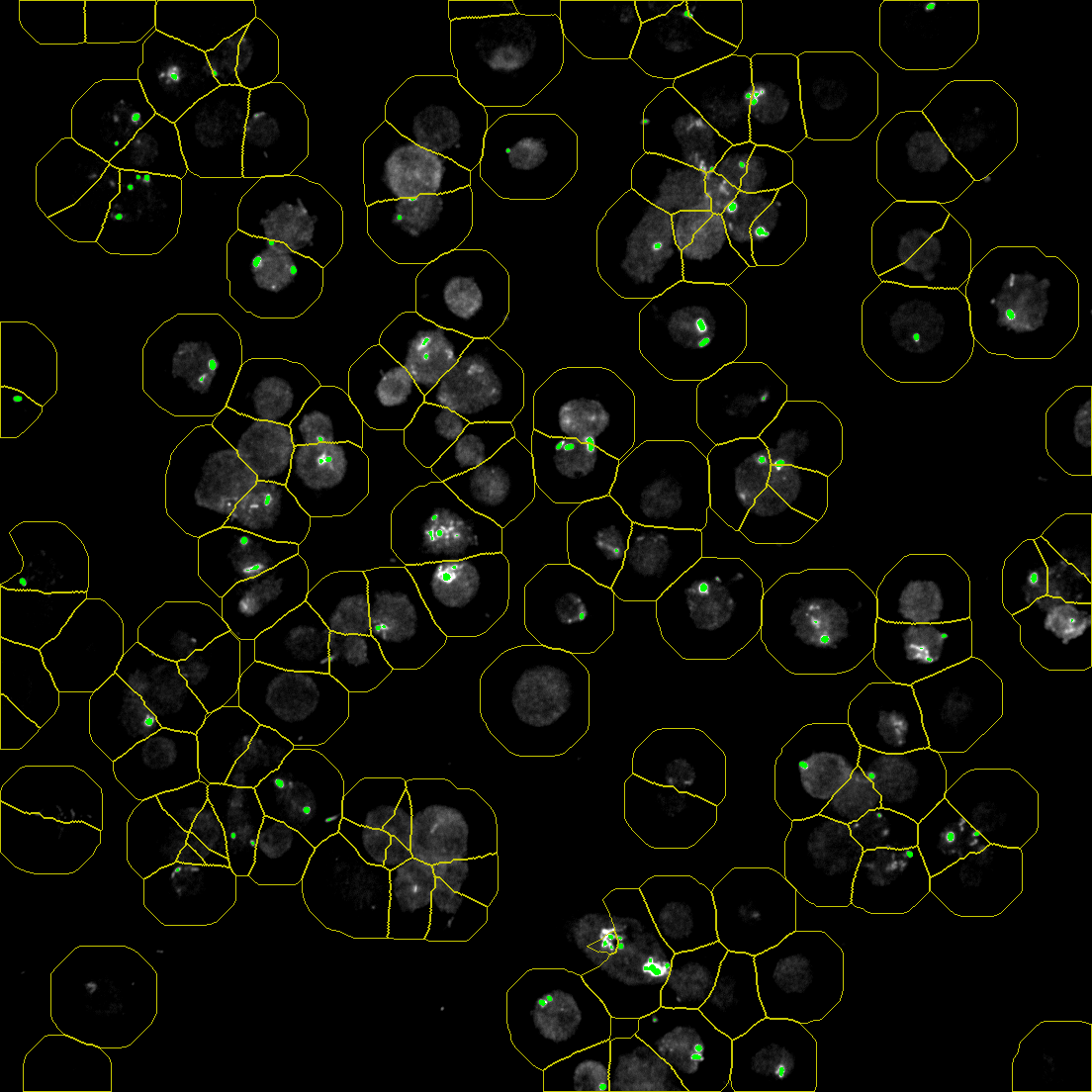

Supplement: Supplementary file 14 — Source Data for Figure 1 [file EMBJ-41-e111289-s020.zip › High Content Screening/1B/1B_SHIGELLA.bmp]

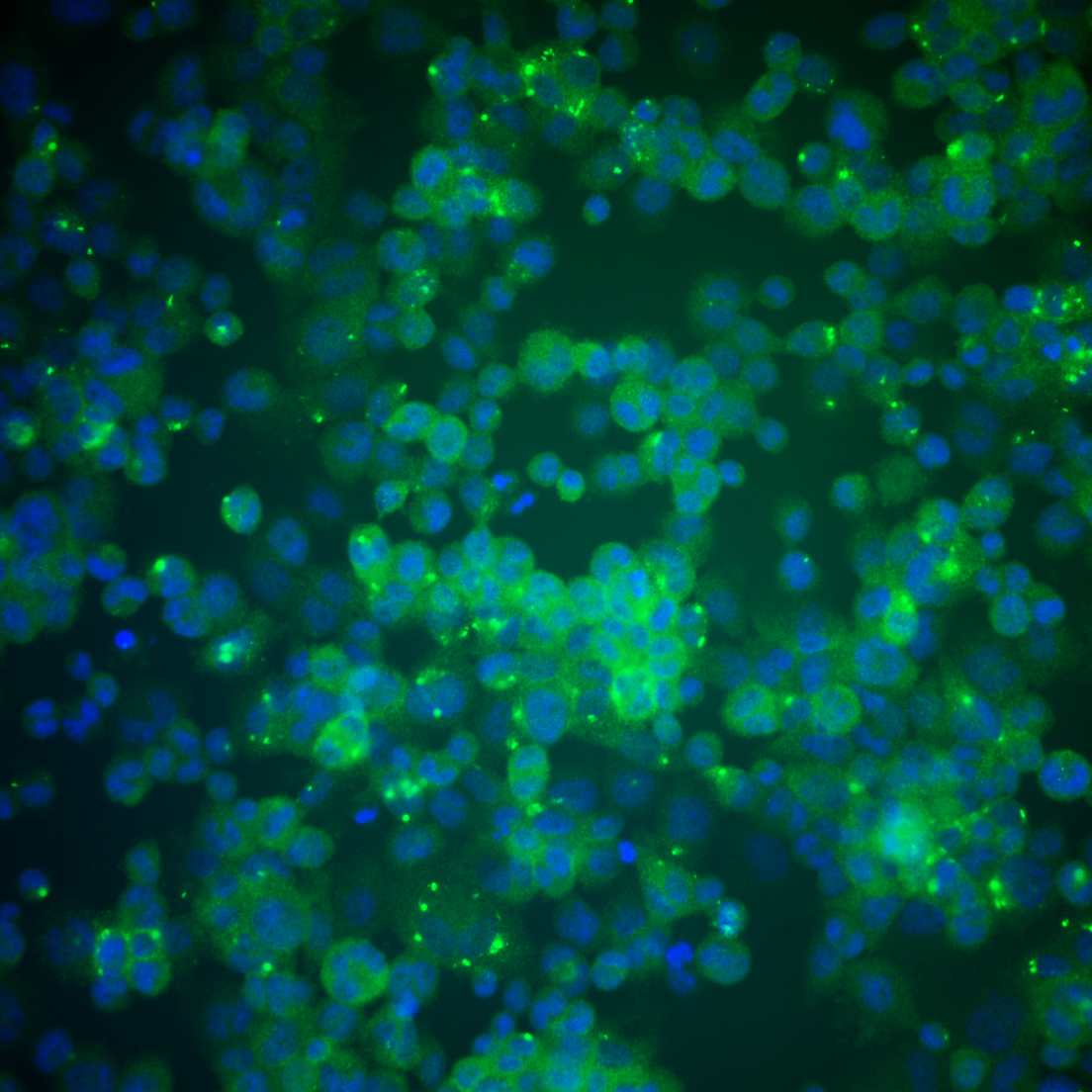

Supplement: Supplementary file 14 — Source Data for Figure 1 [file EMBJ-41-e111289-s020.zip › High Content Screening/1D/1D_Infected_Control.bmp]

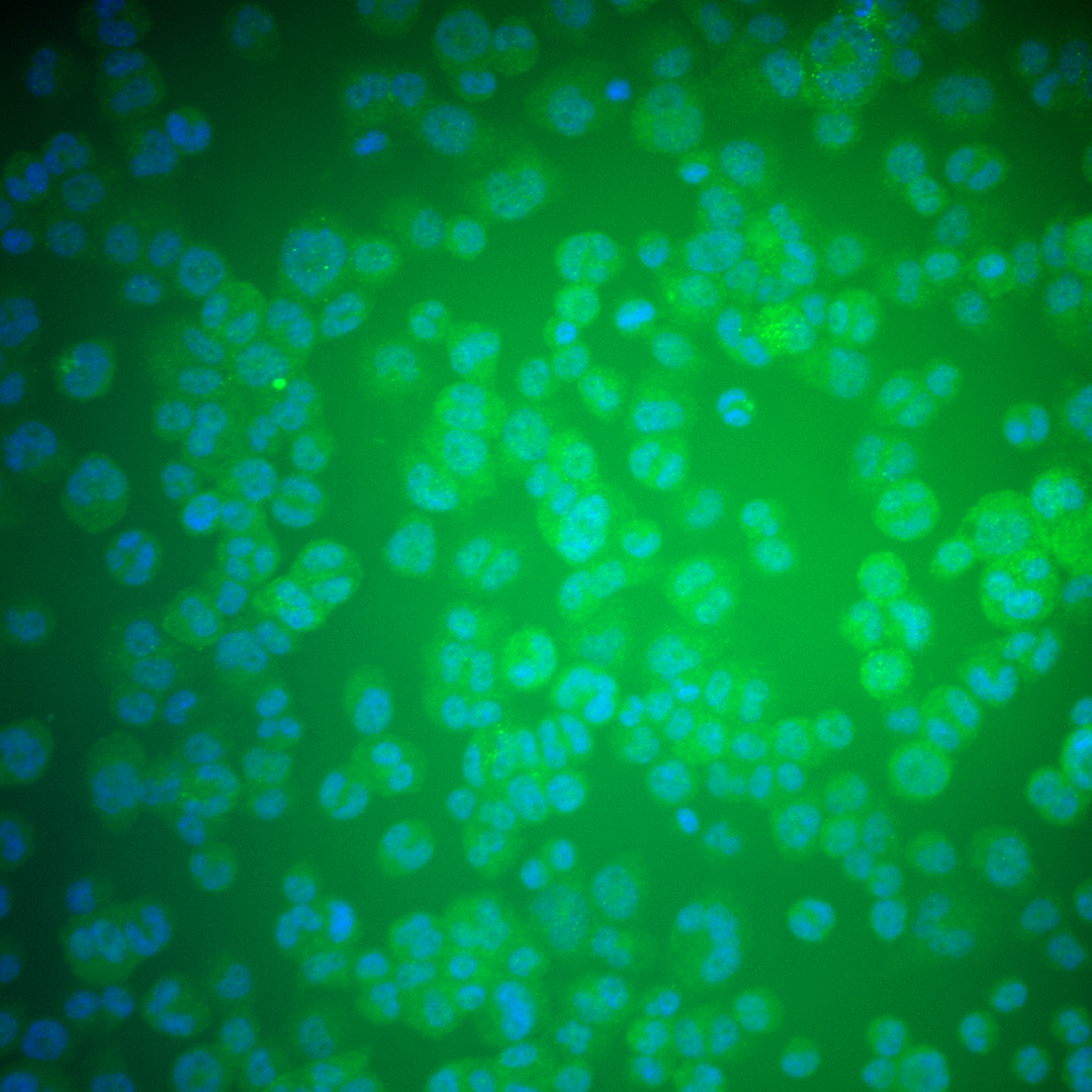

Supplement: Supplementary file 14 — Source Data for Figure 1 [file EMBJ-41-e111289-s020.zip › High Content Screening/1D/1D_ripk2 kd control.bmp]

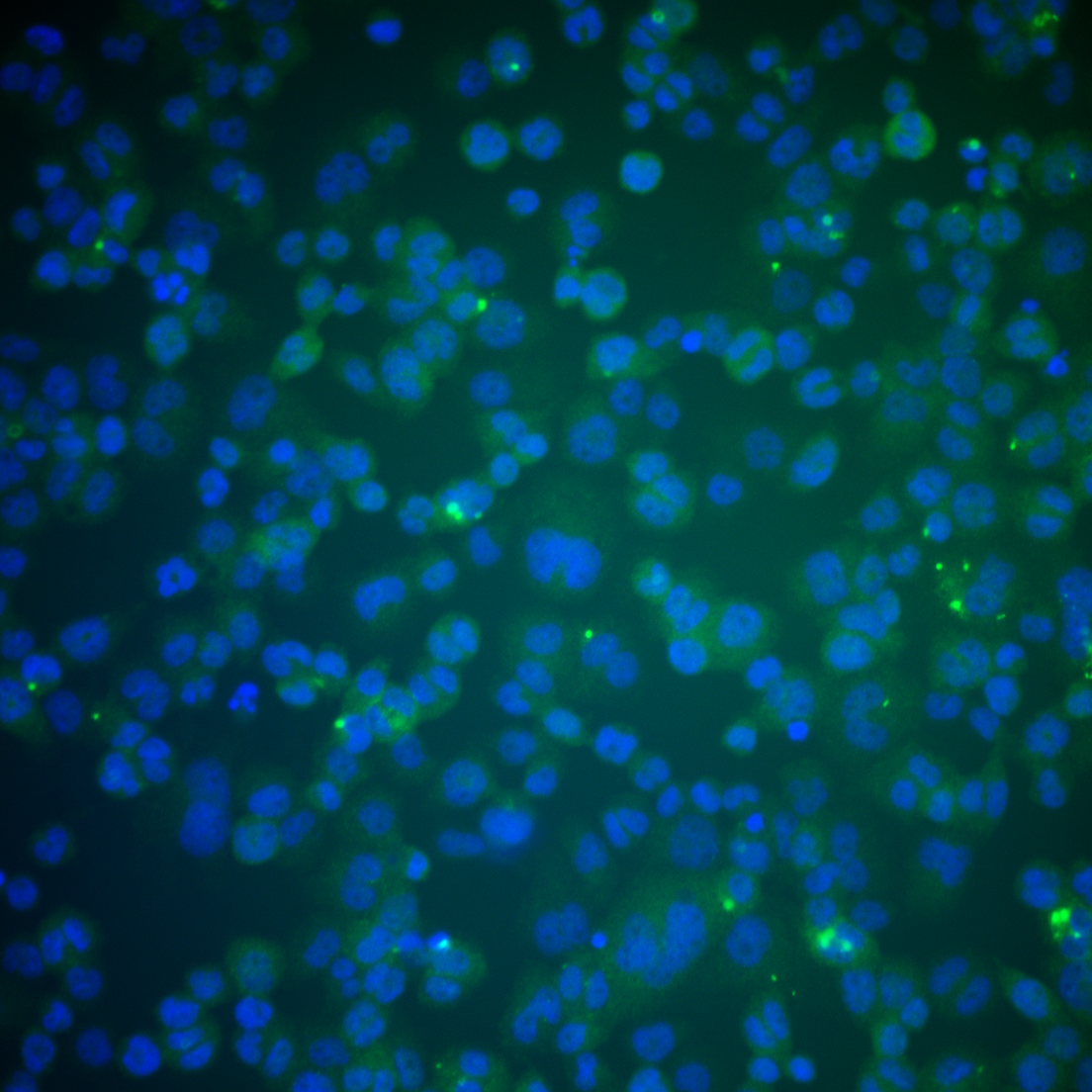

Supplement: Supplementary file 14 — Source Data for Figure 1 [file EMBJ-41-e111289-s020.zip › High Content Screening/1D/1D_ripk2 kd infected.bmp]

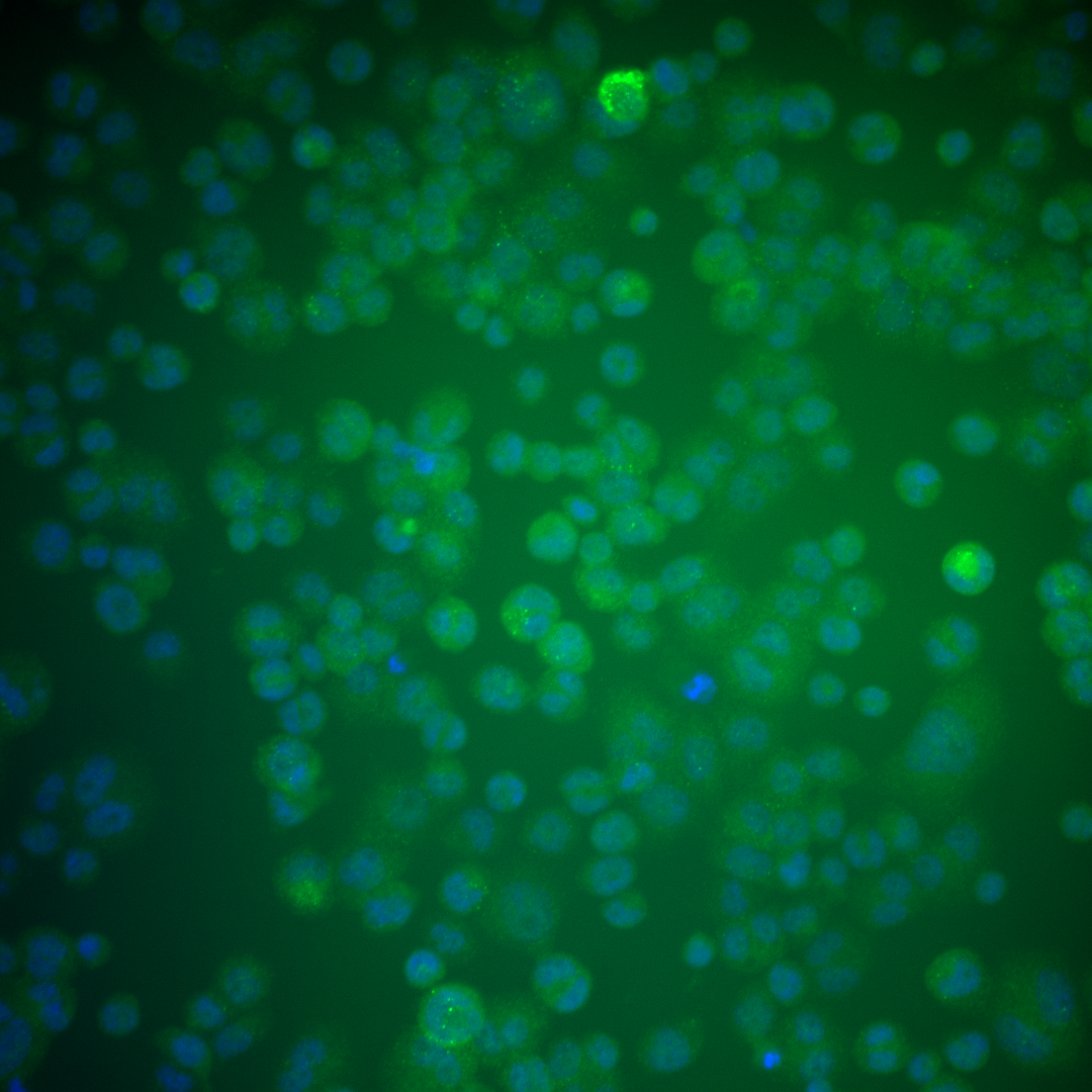

Supplement: Supplementary file 14 — Source Data for Figure 1 [file EMBJ-41-e111289-s020.zip › High Content Screening/1D/1D_Uninfected_Control.bmp]

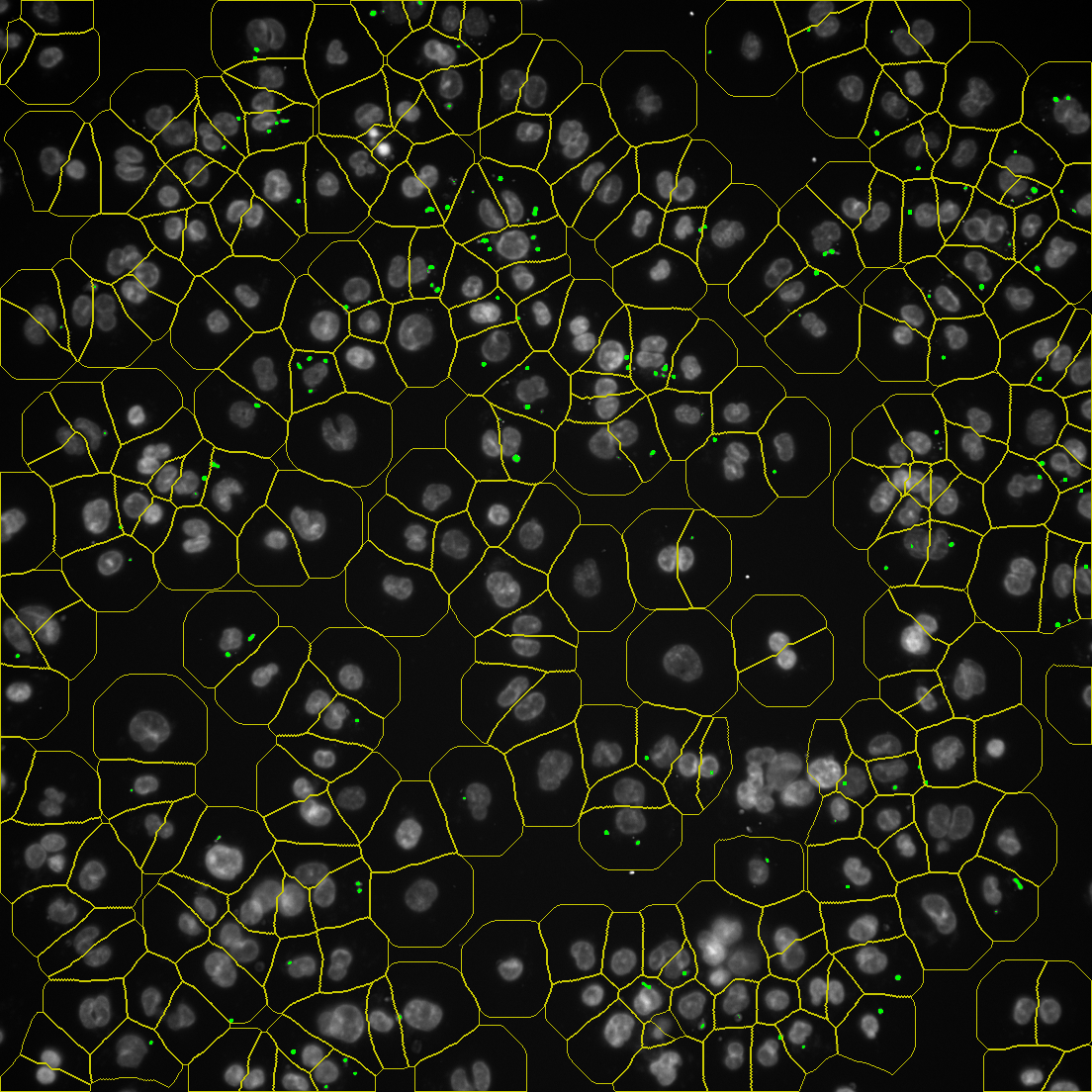

Supplement: Supplementary file 14 — Source Data for Figure 1 [file EMBJ-41-e111289-s020.zip › High Content Screening/1J/1J_infected.bmp]

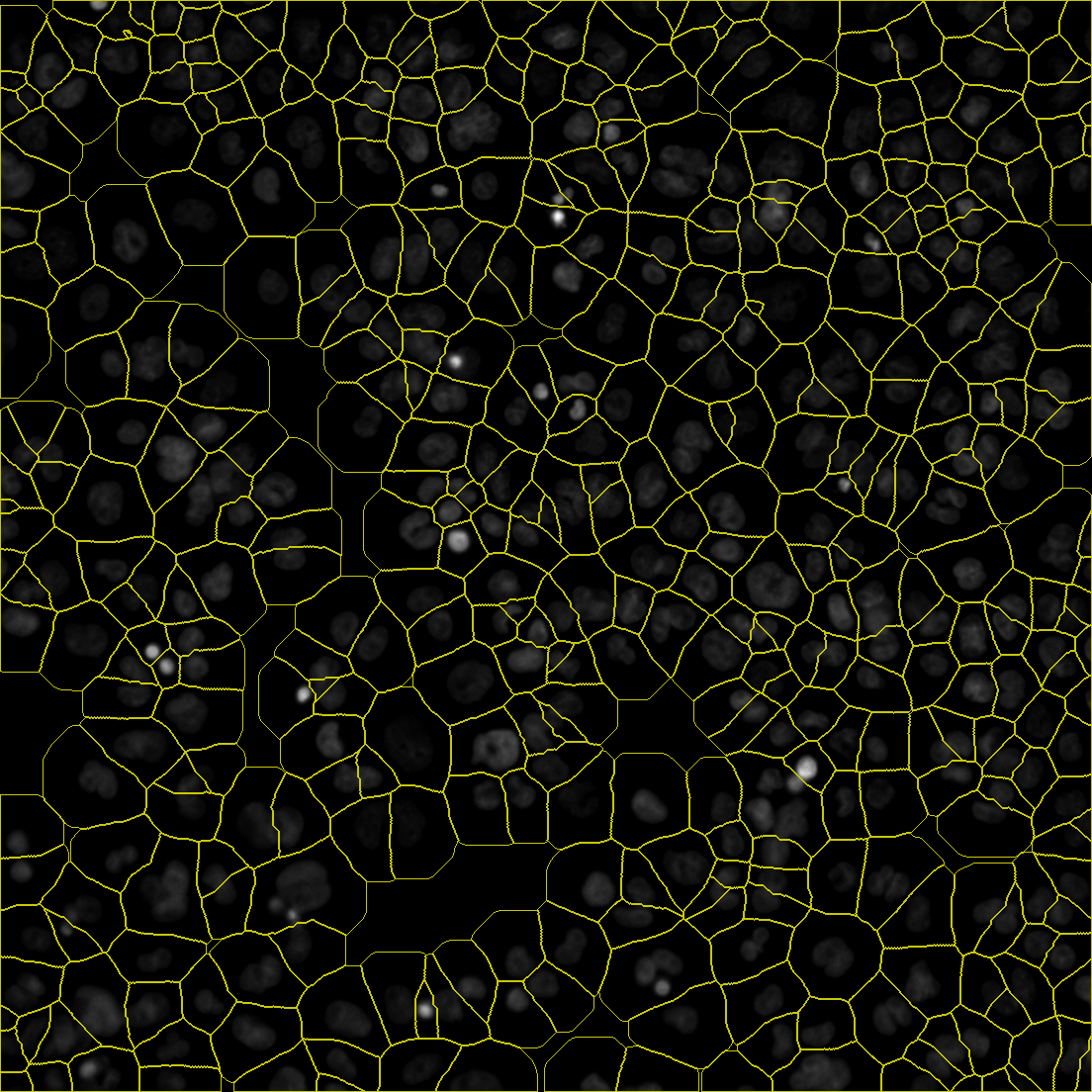

Supplement: Supplementary file 14 — Source Data for Figure 1 [file EMBJ-41-e111289-s020.zip › High Content Screening/1J/1J_uninfected.bmp]

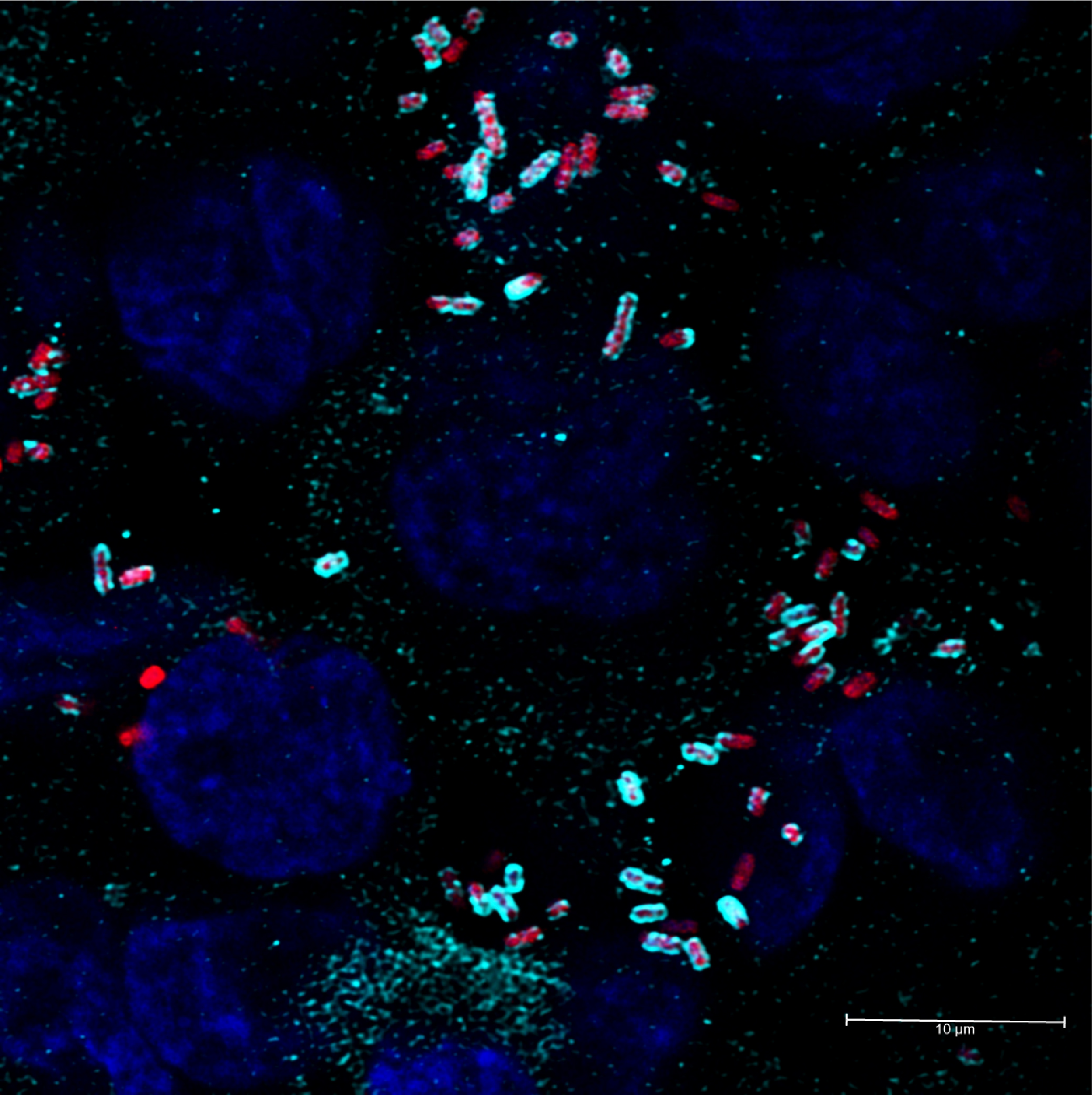

Supplement: Supplementary file 14 — Source Data for Figure 1 [file EMBJ-41-e111289-s020.zip › Microscopy_Confocal/1A/1A-A.jpg]

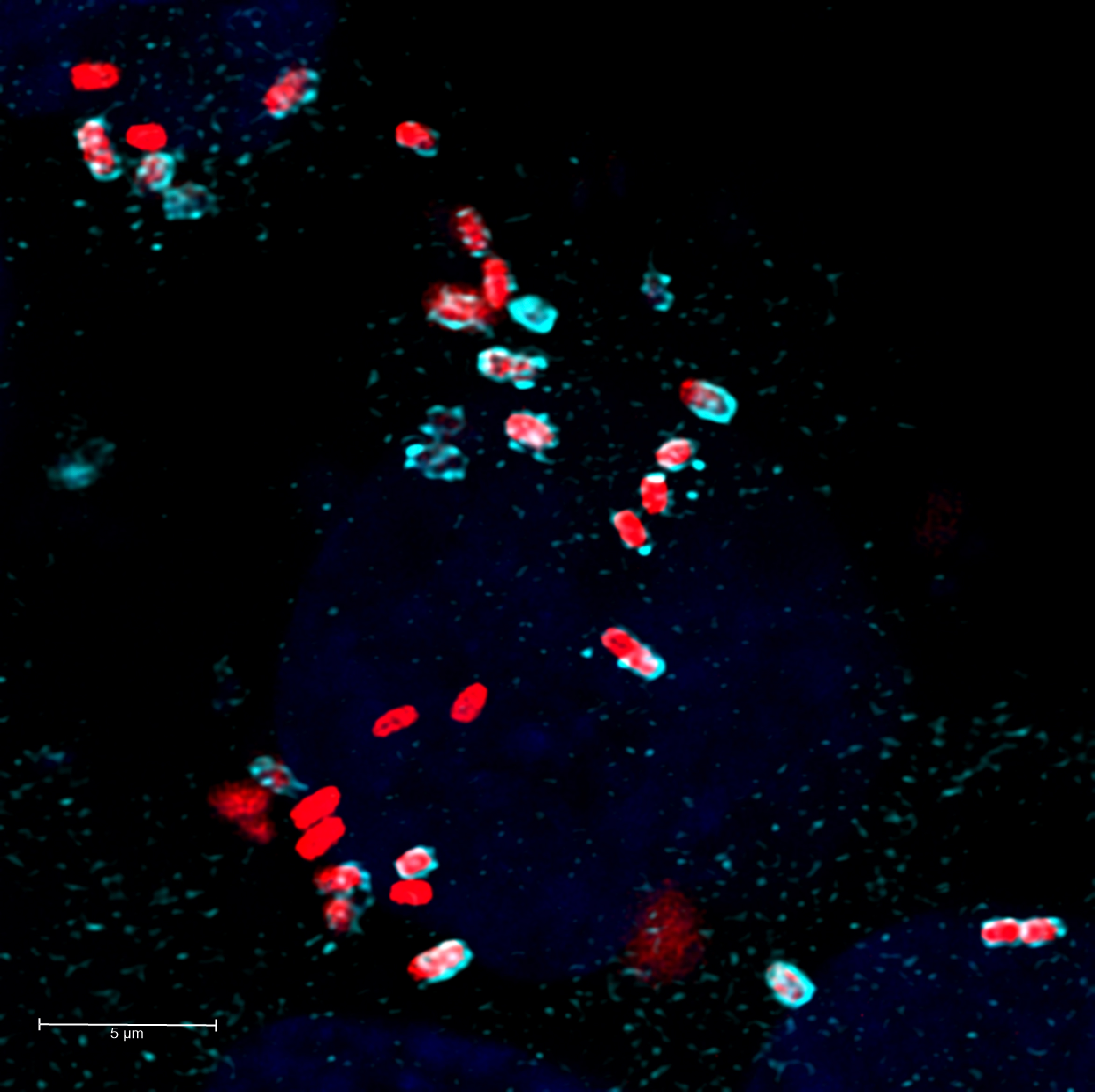

Supplement: Supplementary file 14 — Source Data for Figure 1 [file EMBJ-41-e111289-s020.zip › Microscopy_Confocal/1A/1A-B.jpg]

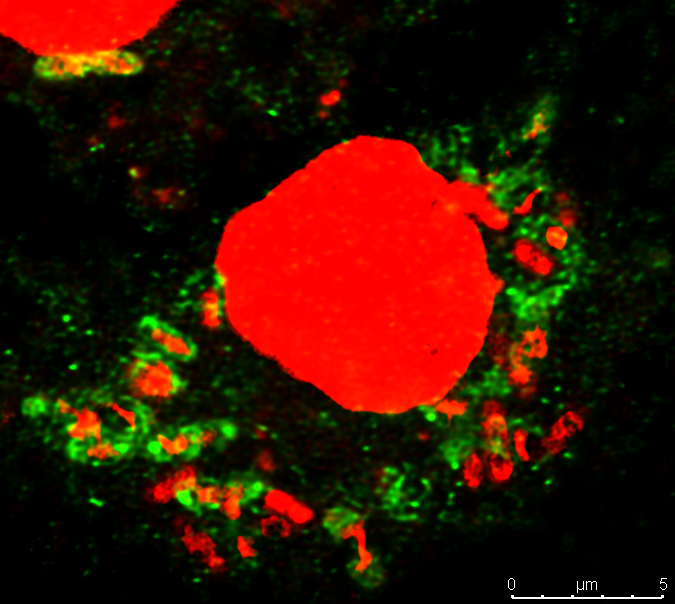

Supplement: Supplementary file 14 — Source Data for Figure 1 [file EMBJ-41-e111289-s020.zip › Microscopy_Confocal/1G/1G.tif]

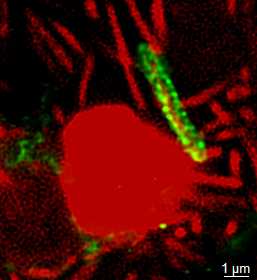

Supplement: Supplementary file 14 — Source Data for Figure 1 [file EMBJ-41-e111289-s020.zip › Microscopy_Confocal/1H/1H-A.tif]

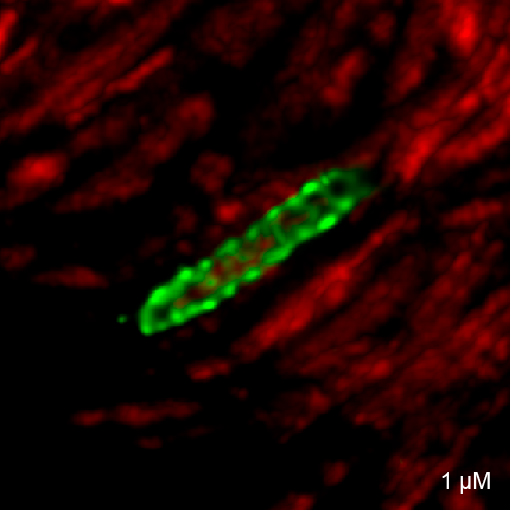

Supplement: Supplementary file 14 — Source Data for Figure 1 [file EMBJ-41-e111289-s020.zip › Microscopy_Confocal/1H/1H-B.jpg]

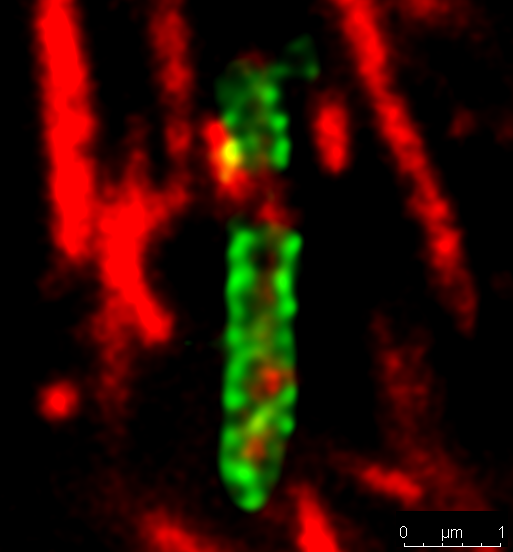

Supplement: Supplementary file 14 — Source Data for Figure 1 [file EMBJ-41-e111289-s020.zip › Microscopy_Confocal/1H/1H-C.tif]

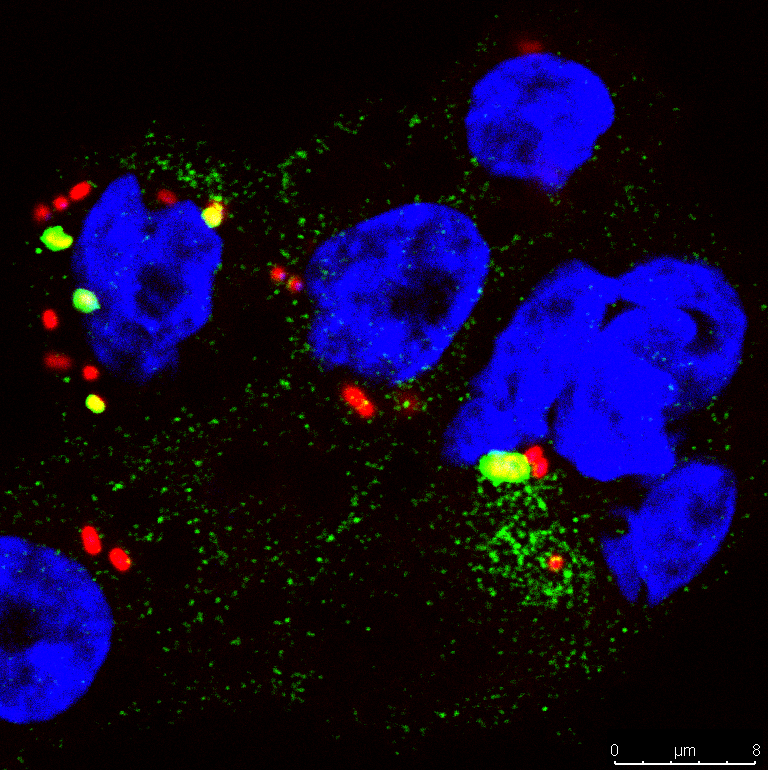

Supplement: Supplementary file 14 — Source Data for Figure 1 [file EMBJ-41-e111289-s020.zip › Microscopy_Confocal/1I/1I.tif]

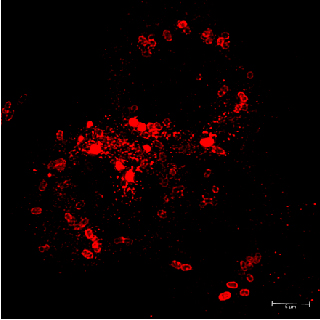

Supplement: Supplementary file 14 — Source Data for Figure 1 [file EMBJ-41-e111289-s020.zip › Microscopy_Confocal/1K/1K_LF82.jpg]

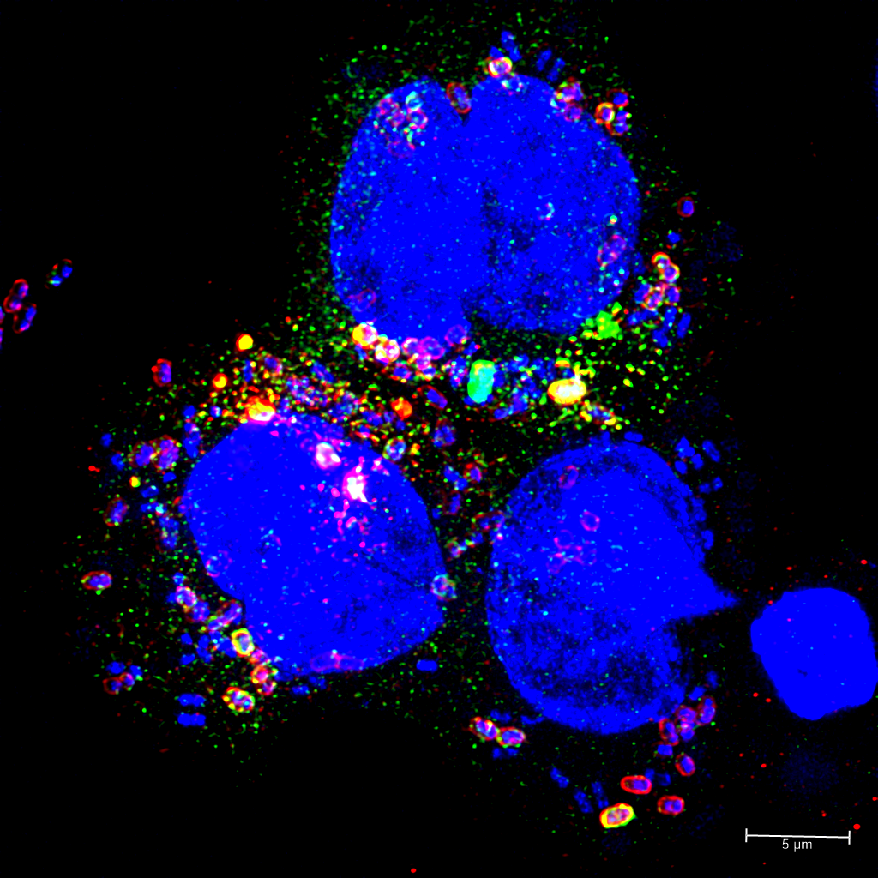

Supplement: Supplementary file 14 — Source Data for Figure 1 [file EMBJ-41-e111289-s020.zip › Microscopy_Confocal/1K/1K_Merge.tif]

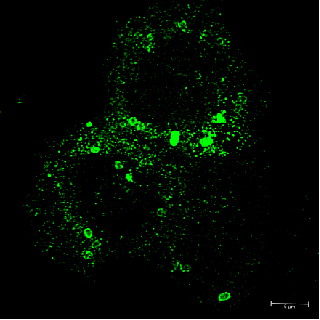

Supplement: Supplementary file 14 — Source Data for Figure 1 [file EMBJ-41-e111289-s020.zip › Microscopy_Confocal/1K/1K_RIPK2.jpg]

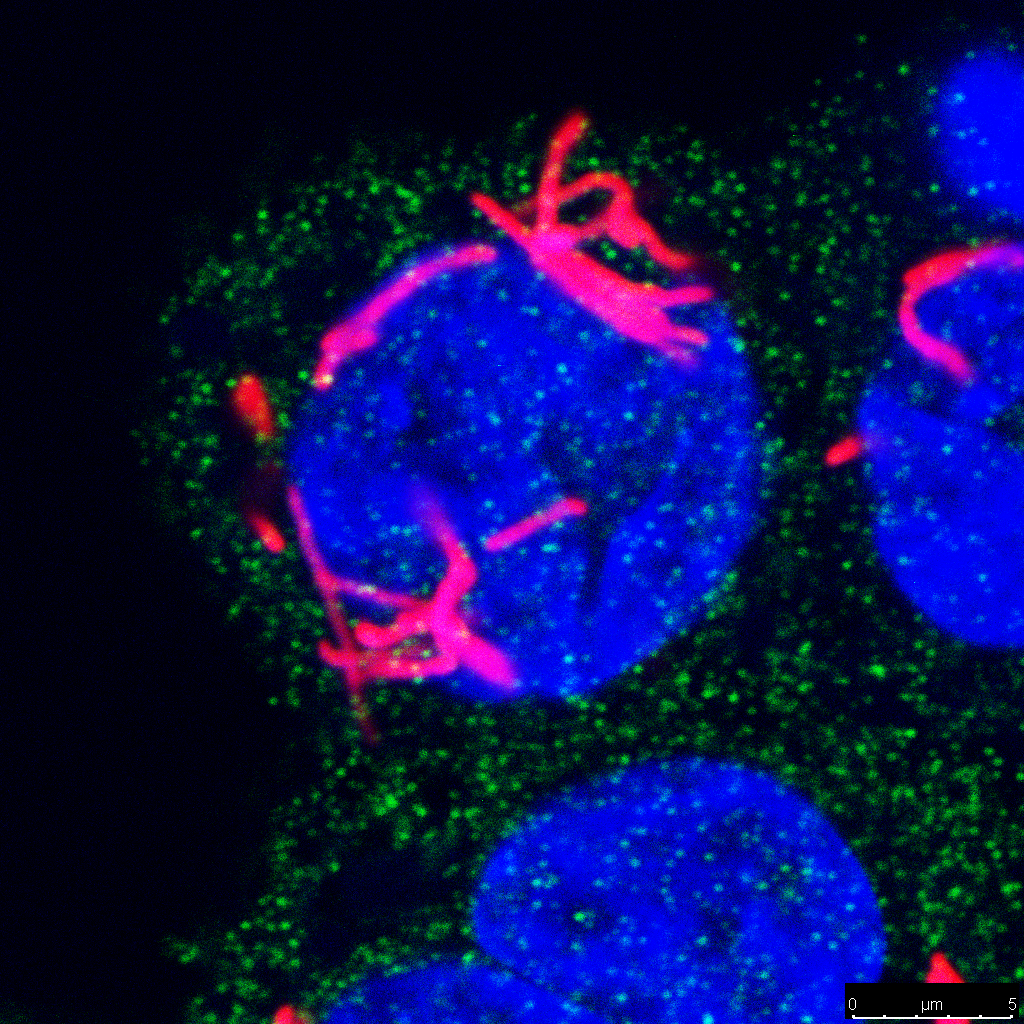

Supplement: Supplementary file 14 — Source Data for Figure 1 [file EMBJ-41-e111289-s020.zip › Microscopy_Confocal/1L/1L-A.tif]

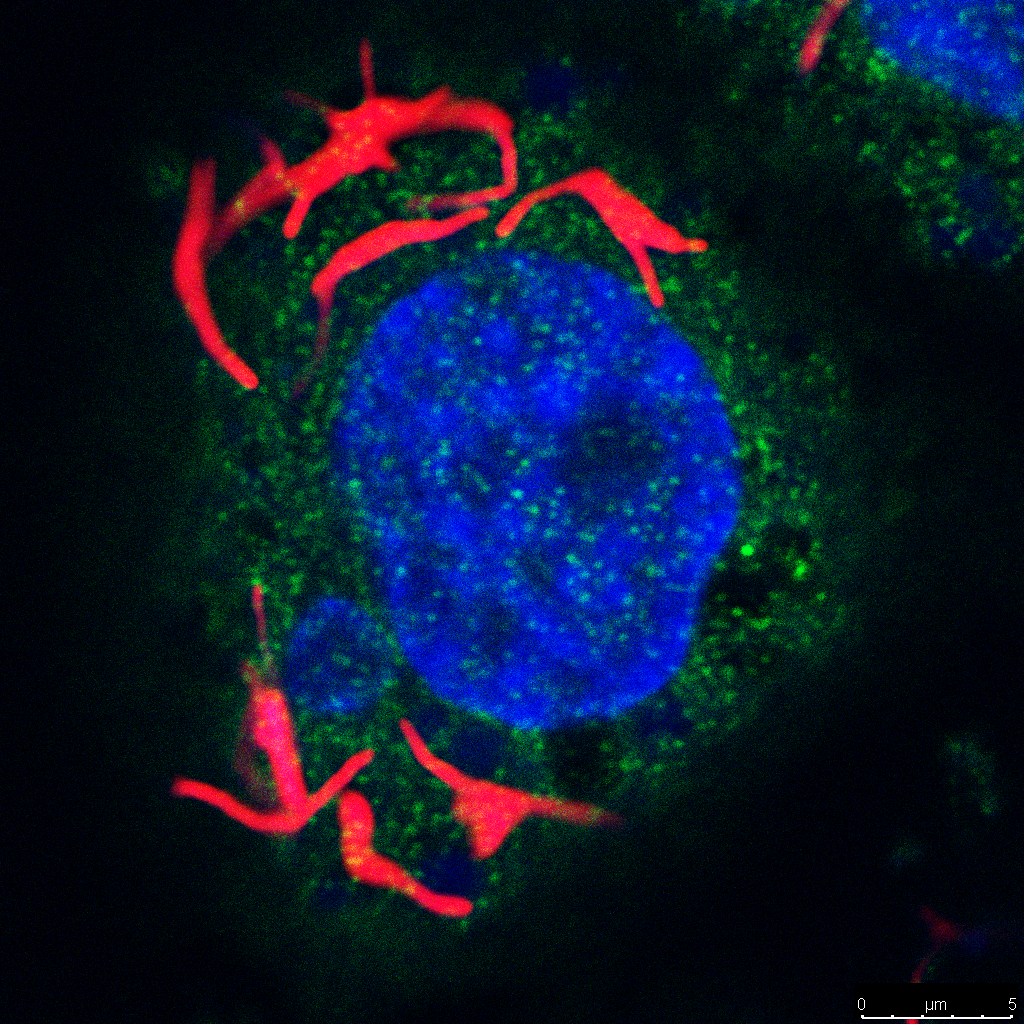

Supplement: Supplementary file 14 — Source Data for Figure 1 [file EMBJ-41-e111289-s020.zip › Microscopy_Confocal/1L/1L-B.tif]

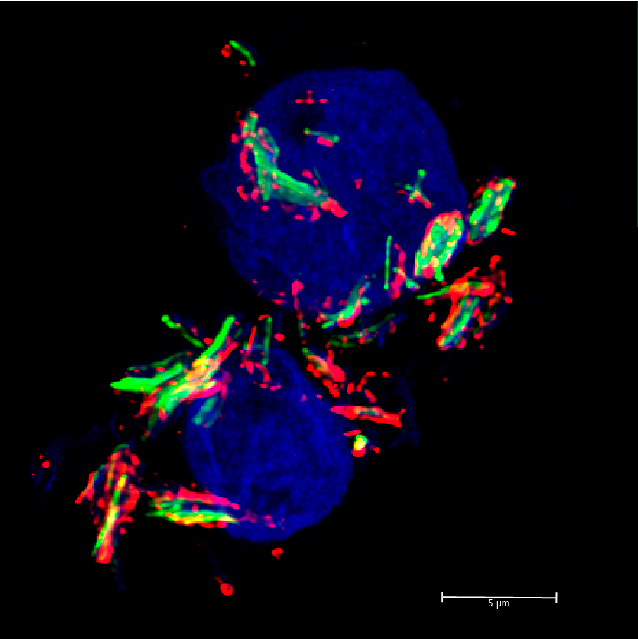

Supplement: Supplementary file 14 — Source Data for Figure 1 [file EMBJ-41-e111289-s020.zip › Microscopy_Confocal/1M/1M.jpg]

Figure-1

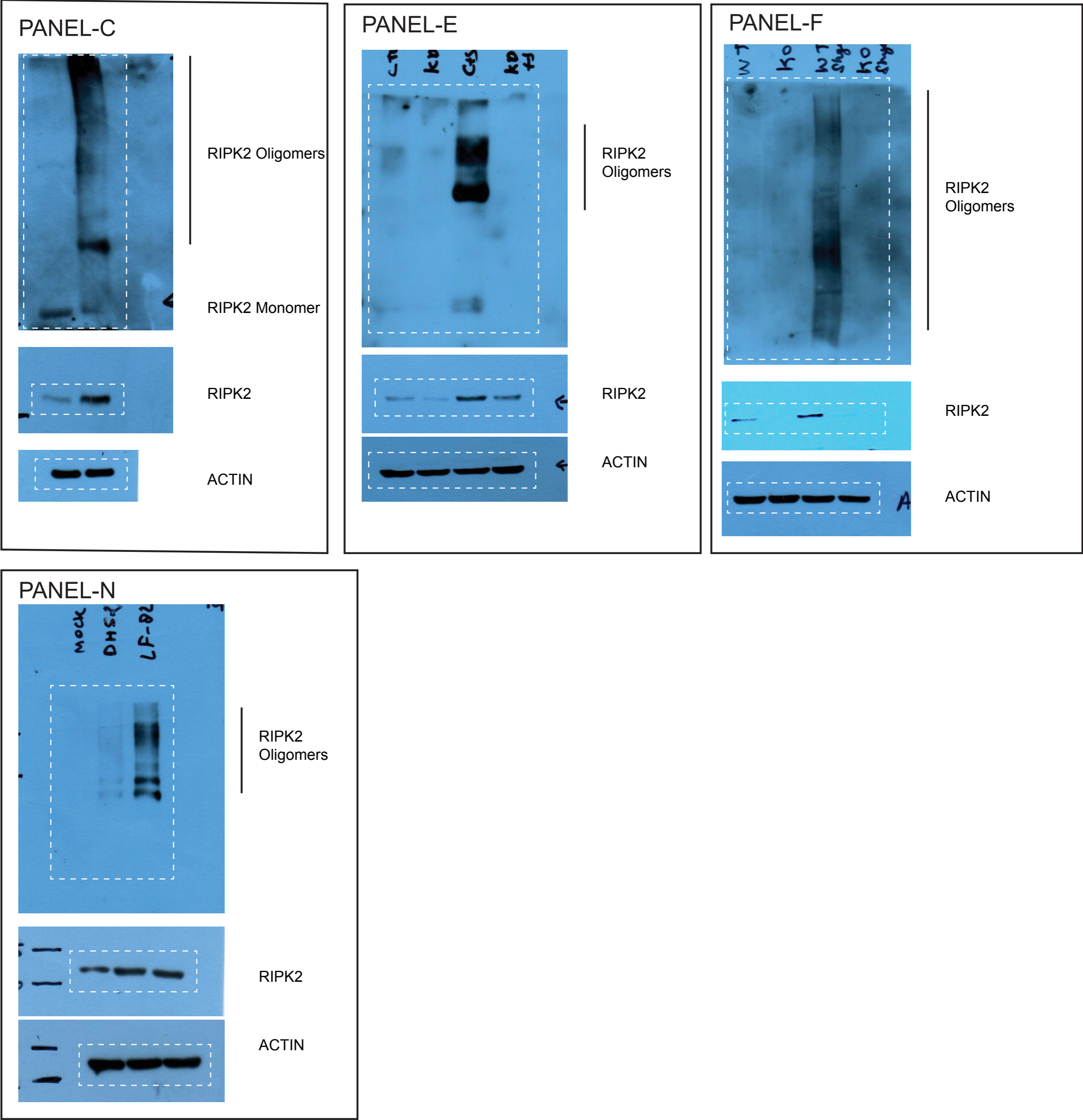

Supplement: Supplementary file 14 — Source Data for Figure 1 [file EMBJ-41-e111289-s020.zip › Western Blot/Western Blot Figure-1.pdf]

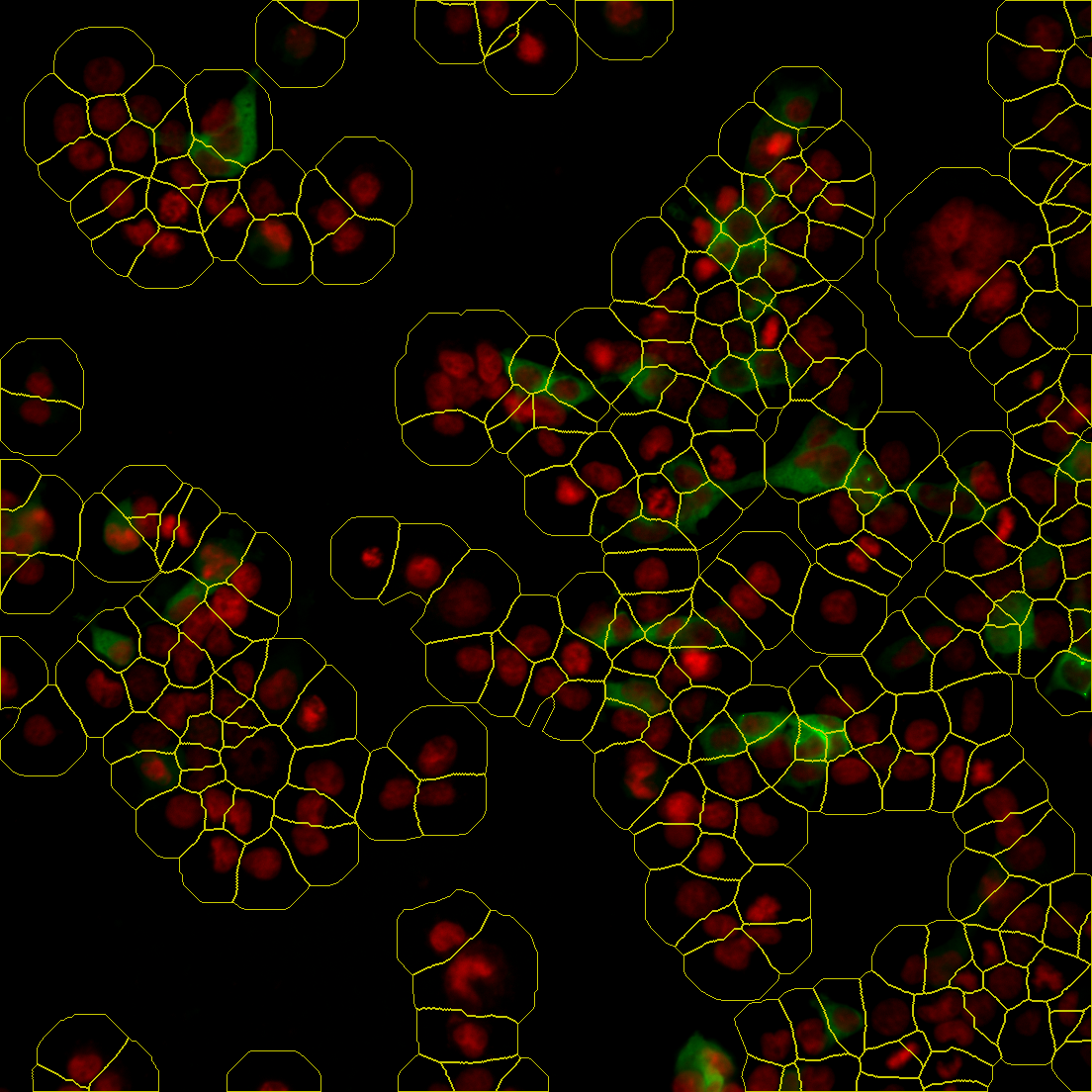

Supplement: Supplementary file 15 — Source Data for Figure 2 [file EMBJ-41-e111289-s008.zip › High Content Screening/2B/2B_GFP NOD1.bmp]

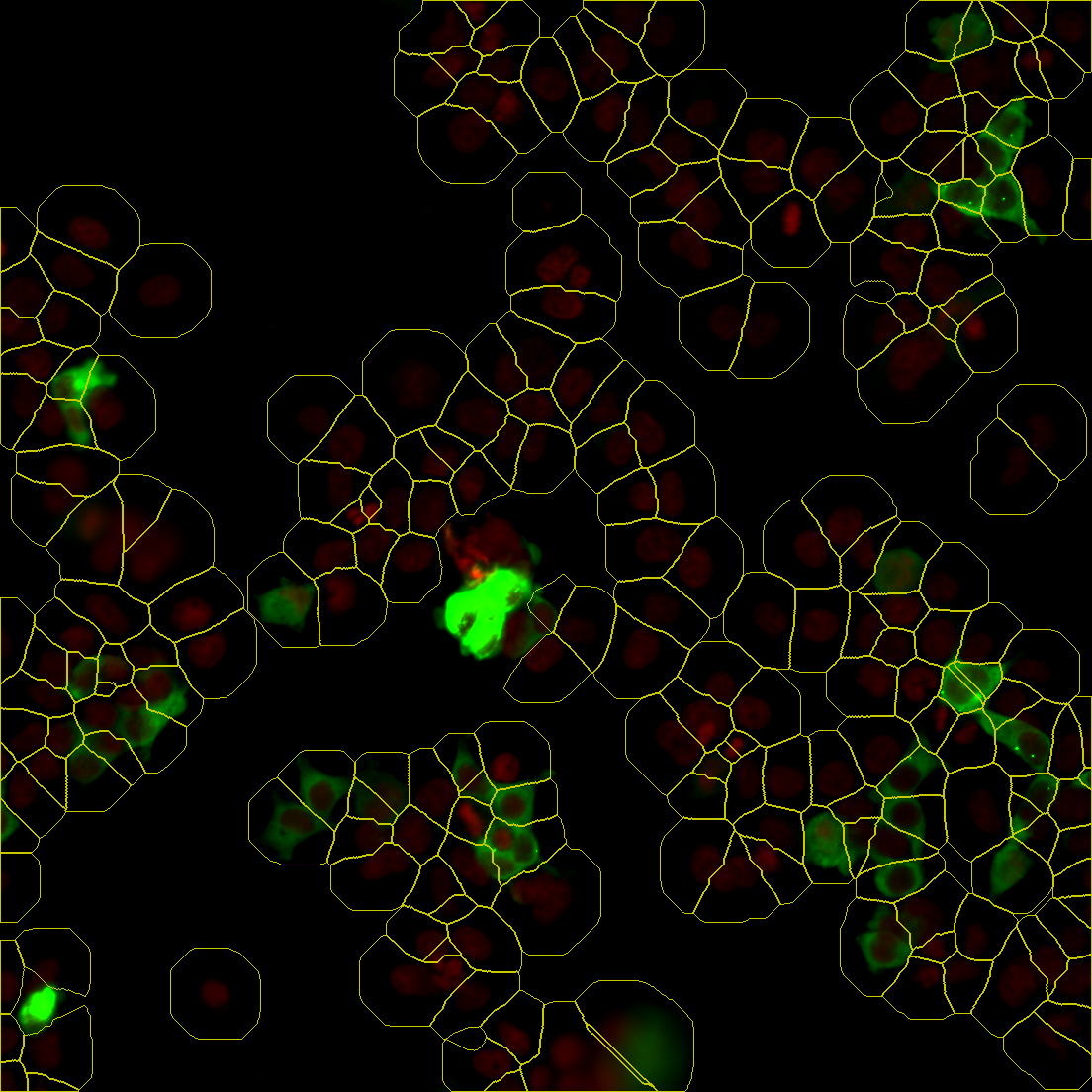

Supplement: Supplementary file 15 — Source Data for Figure 2 [file EMBJ-41-e111289-s008.zip › High Content Screening/2B/2B_GFP NOD2.bmp]

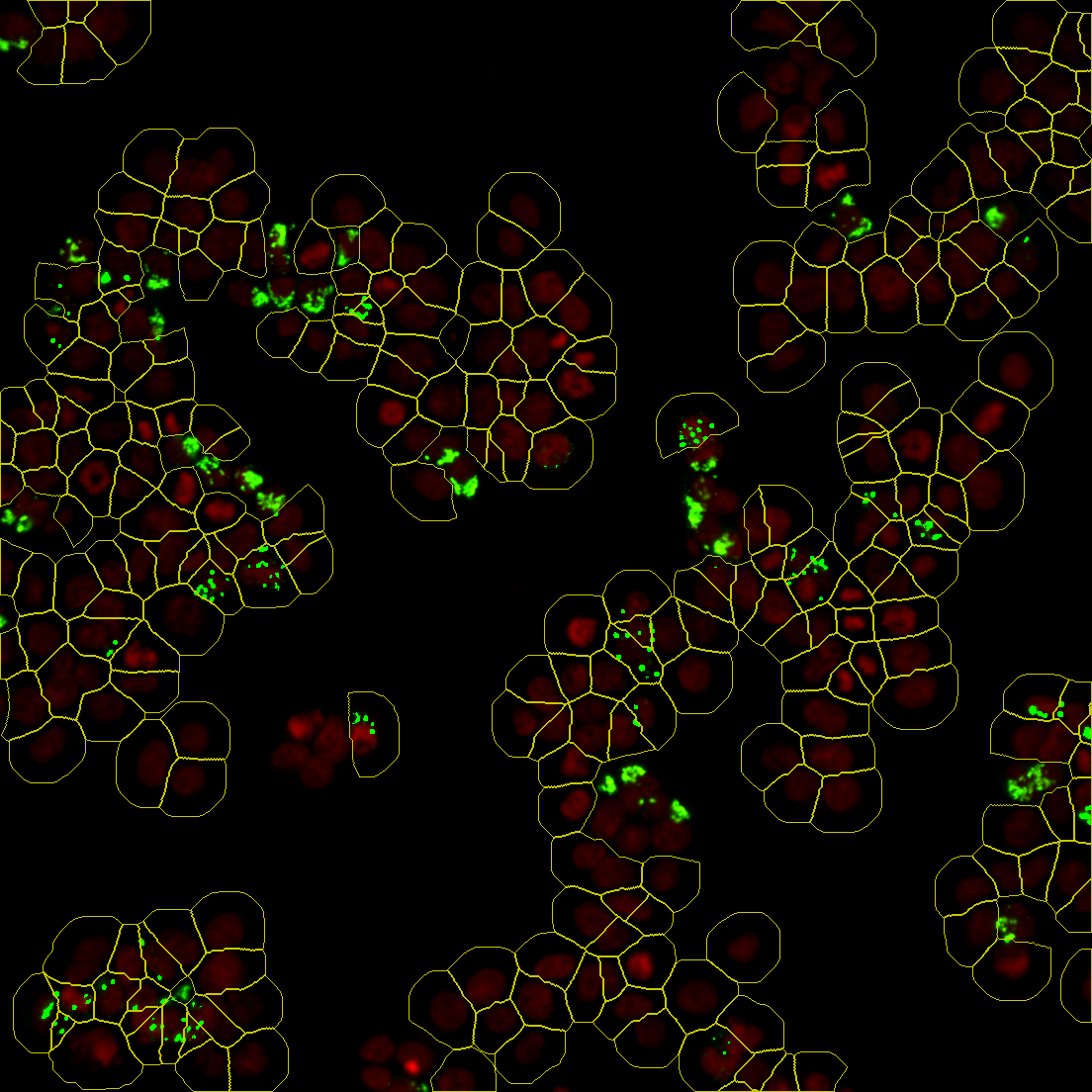

Supplement: Supplementary file 15 — Source Data for Figure 2 [file EMBJ-41-e111289-s008.zip › High Content Screening/2B/2B_GFP RIPK2.bmp]

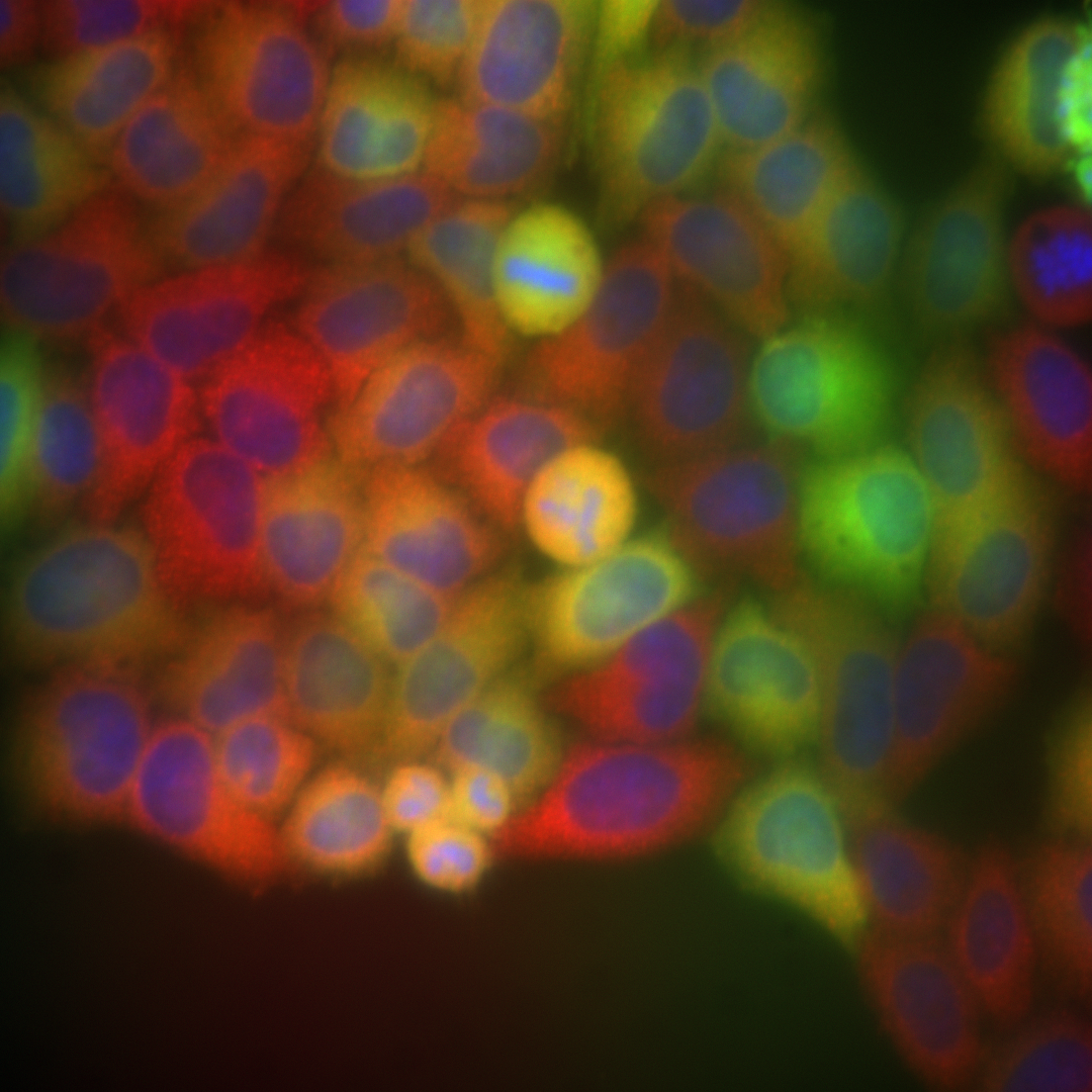

Supplement: Supplementary file 15 — Source Data for Figure 2 [file EMBJ-41-e111289-s008.zip › High Content Screening/2L/2L- un-4.BMP]

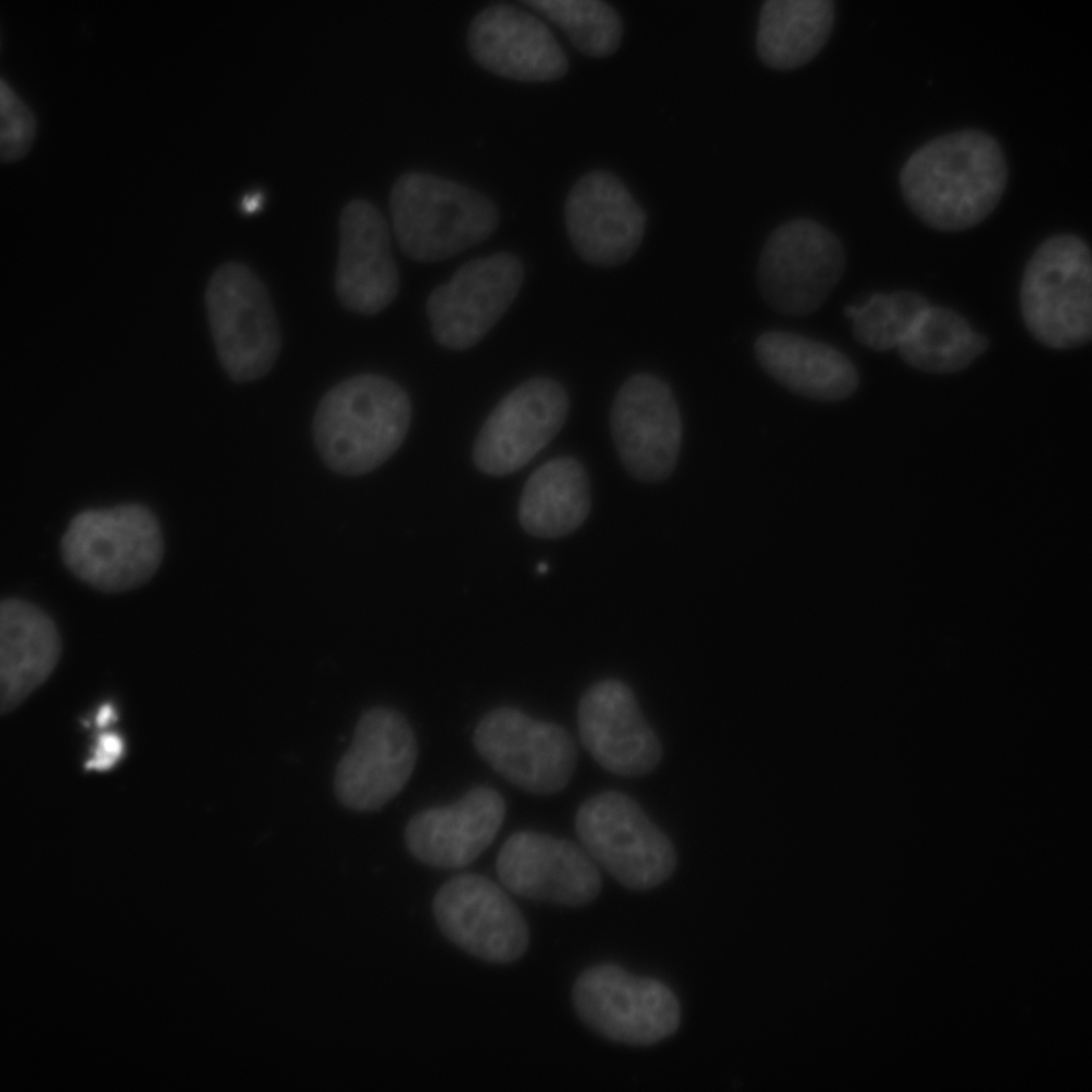

Supplement: Supplementary file 15 — Source Data for Figure 2 [file EMBJ-41-e111289-s008.zip › High Content Screening/2L/2L-inf-1.TIF]

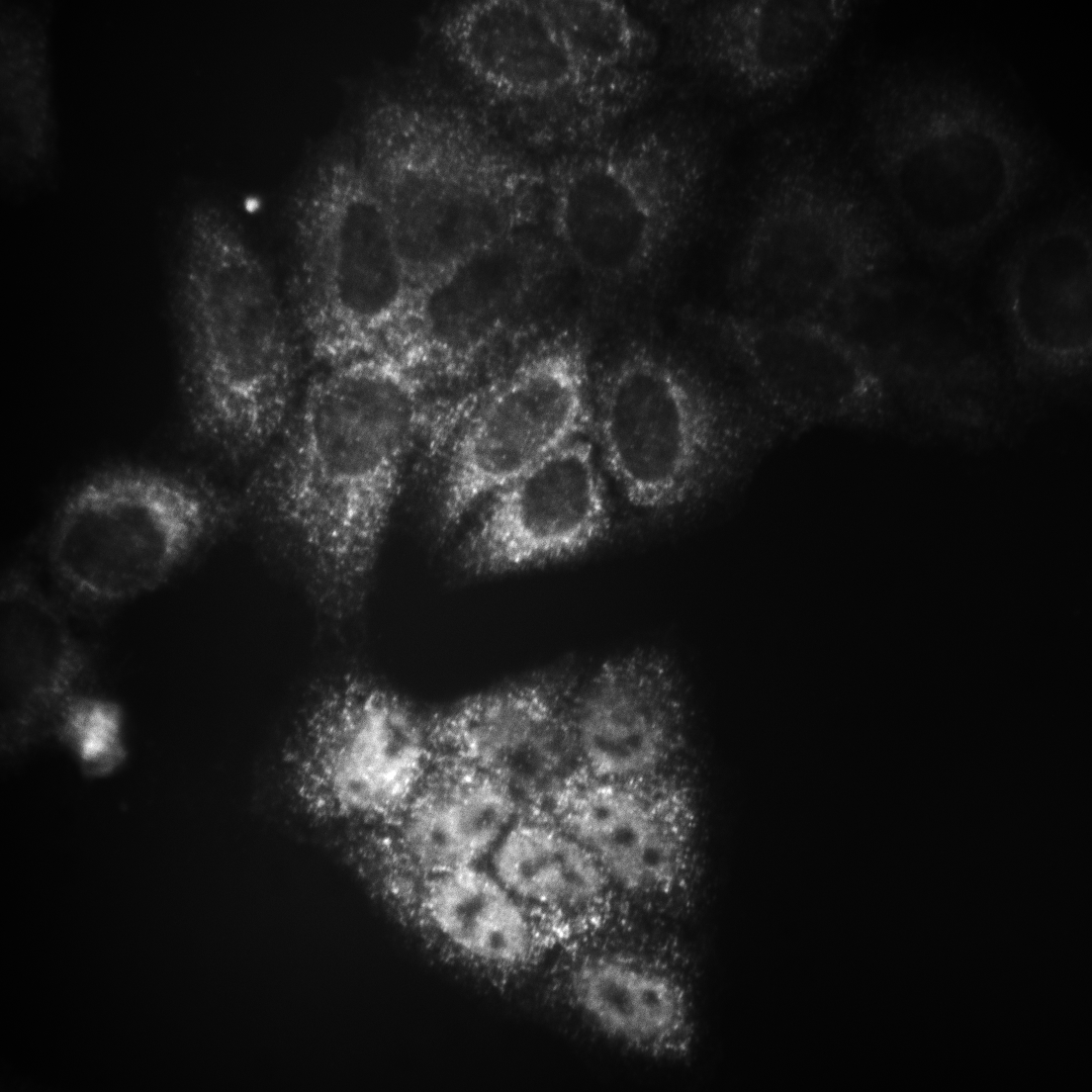

Supplement: Supplementary file 15 — Source Data for Figure 2 [file EMBJ-41-e111289-s008.zip › High Content Screening/2L/2L-inf-2.TIF]

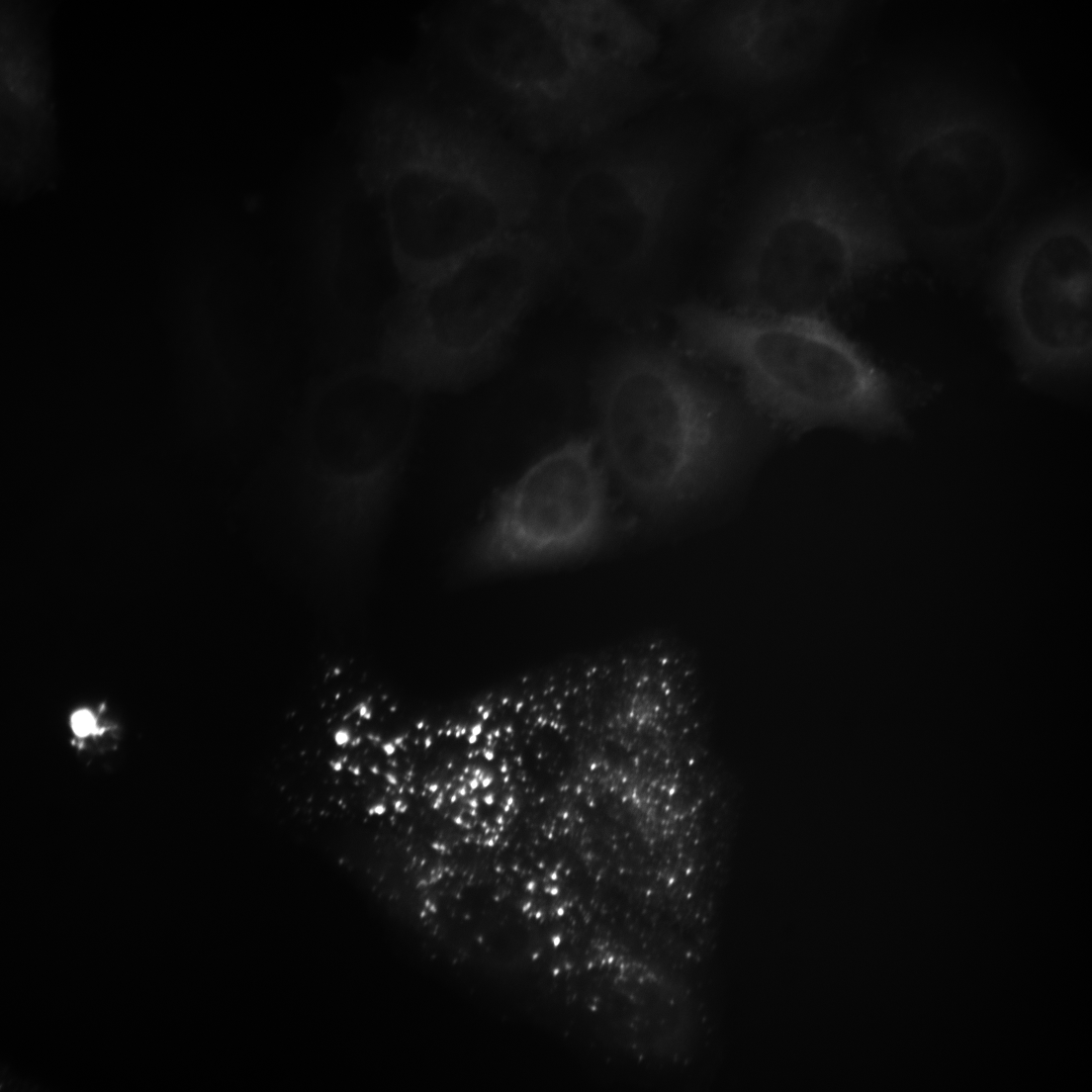

Supplement: Supplementary file 15 — Source Data for Figure 2 [file EMBJ-41-e111289-s008.zip › High Content Screening/2L/2L-inf-3.TIF]

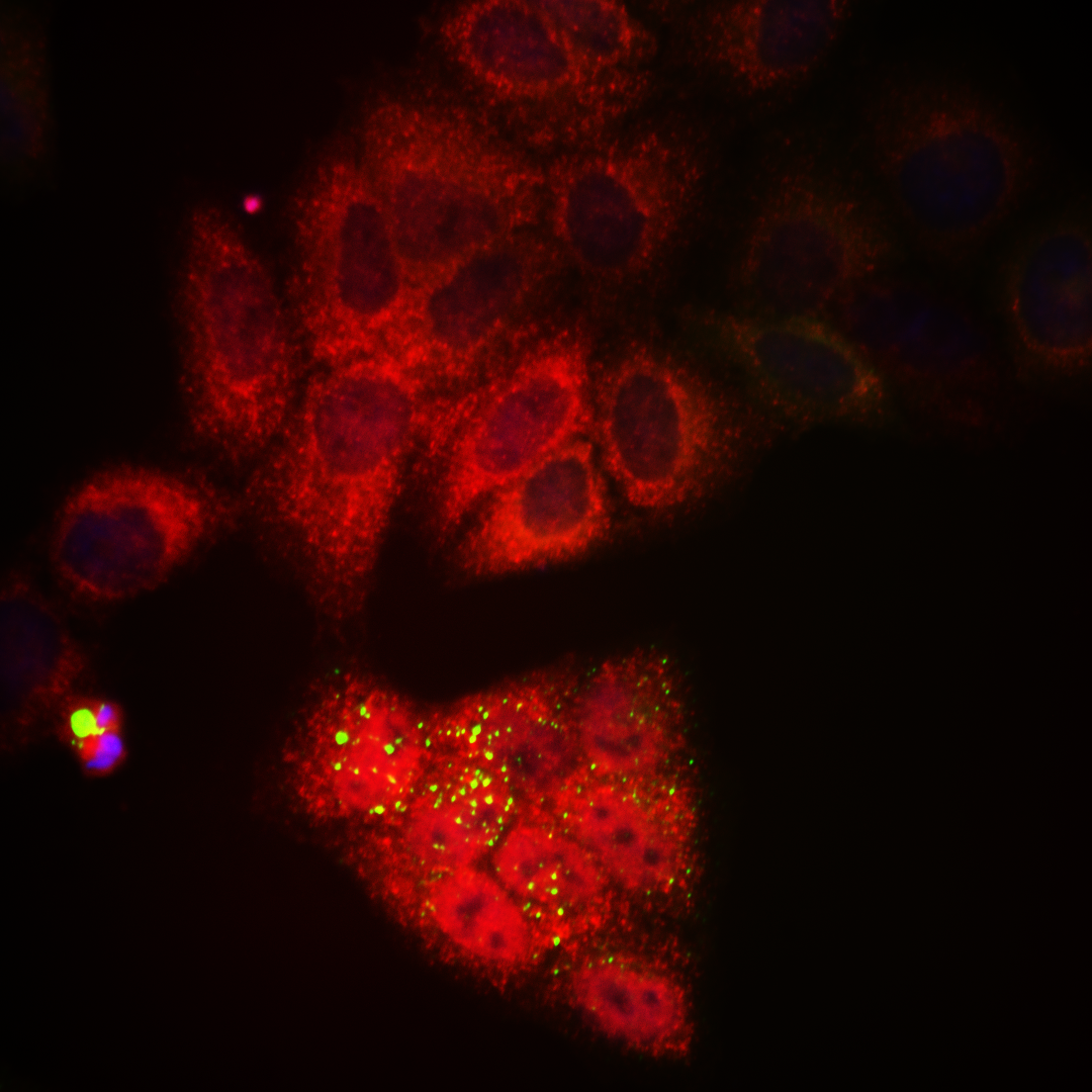

Supplement: Supplementary file 15 — Source Data for Figure 2 [file EMBJ-41-e111289-s008.zip › High Content Screening/2L/2L-inf-4.BMP]

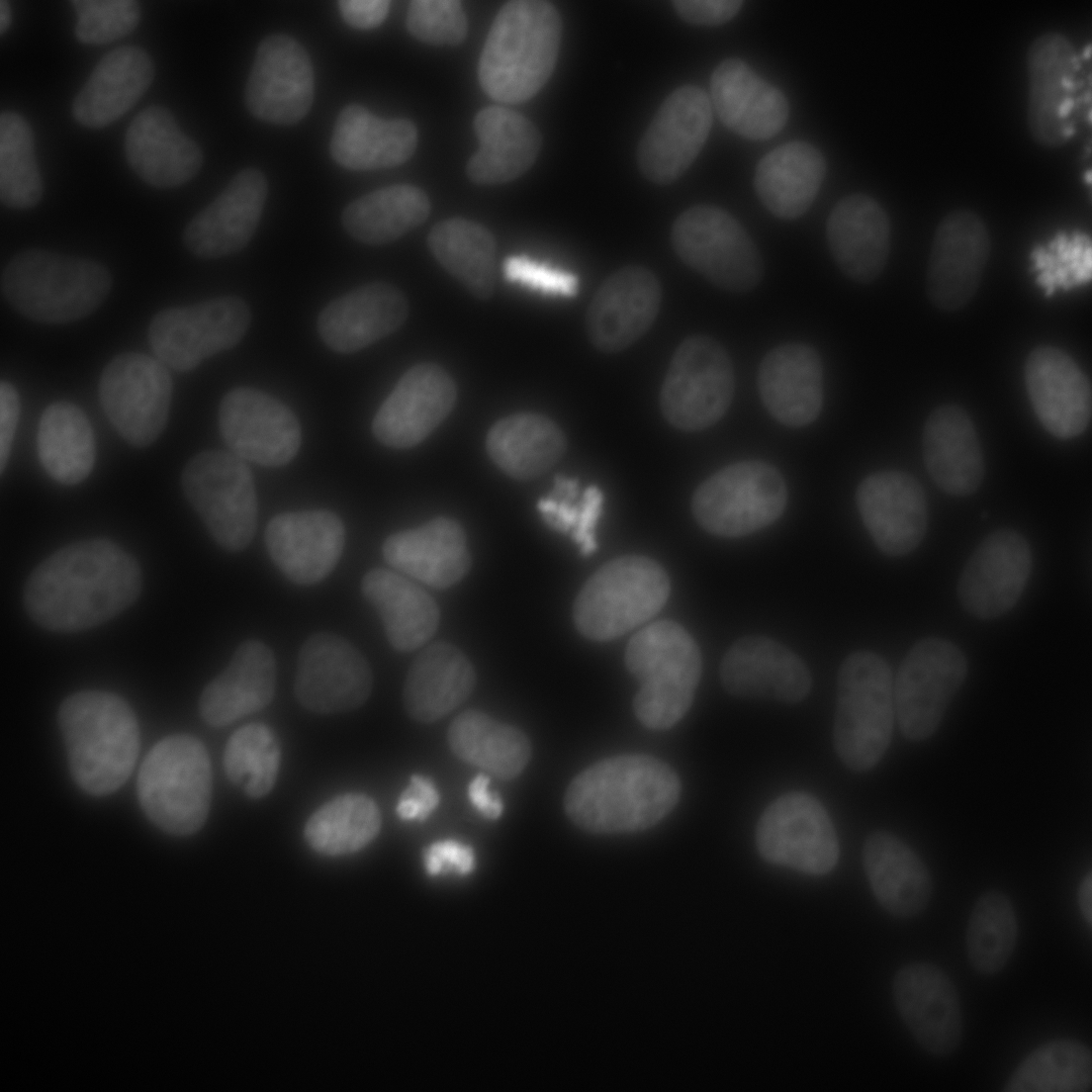

Supplement: Supplementary file 15 — Source Data for Figure 2 [file EMBJ-41-e111289-s008.zip › High Content Screening/2L/2L-un-1.TIF]

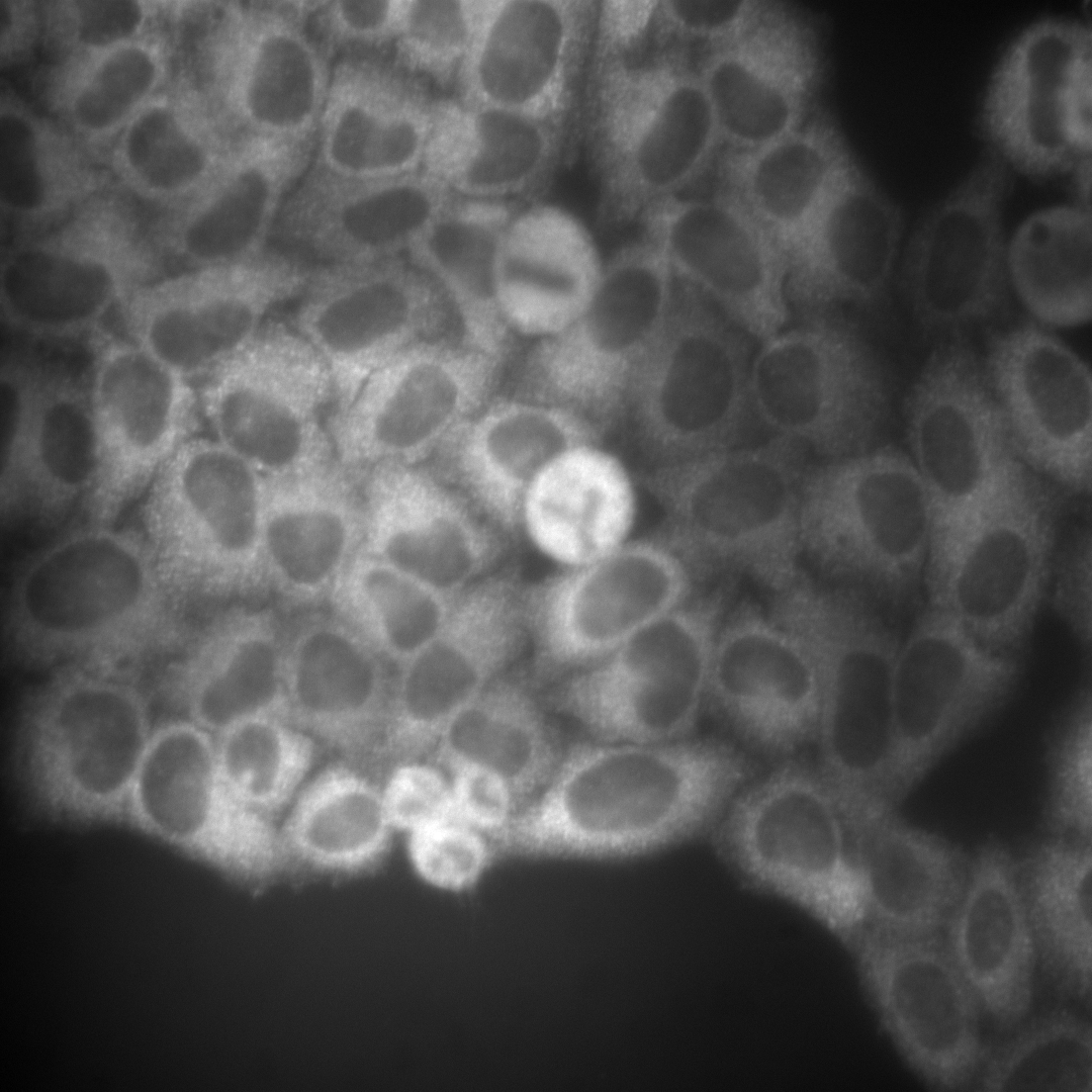

Supplement: Supplementary file 15 — Source Data for Figure 2 [file EMBJ-41-e111289-s008.zip › High Content Screening/2L/2L-un-2.TIF]

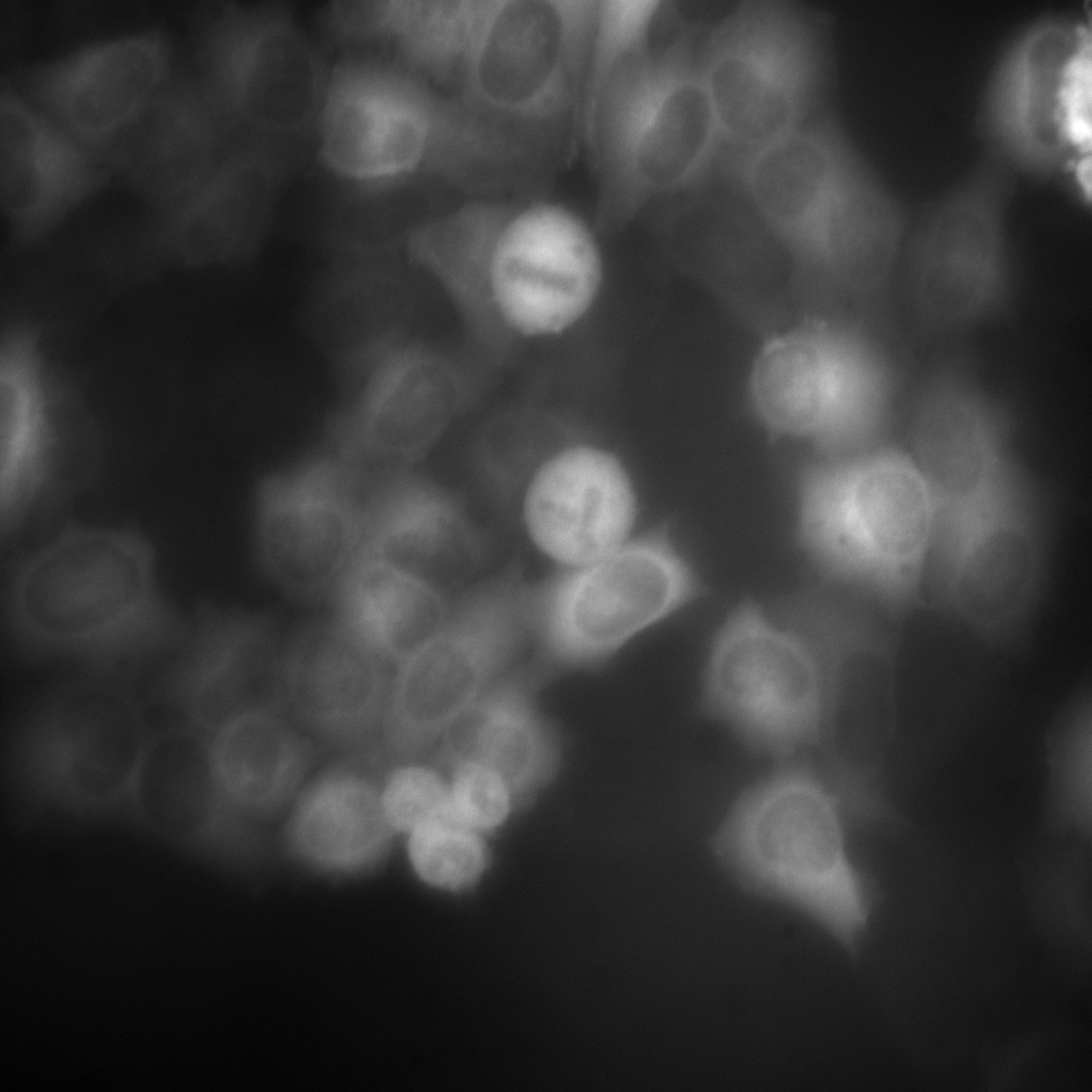

Supplement: Supplementary file 15 — Source Data for Figure 2 [file EMBJ-41-e111289-s008.zip › High Content Screening/2L/2L-un-3.TIF]

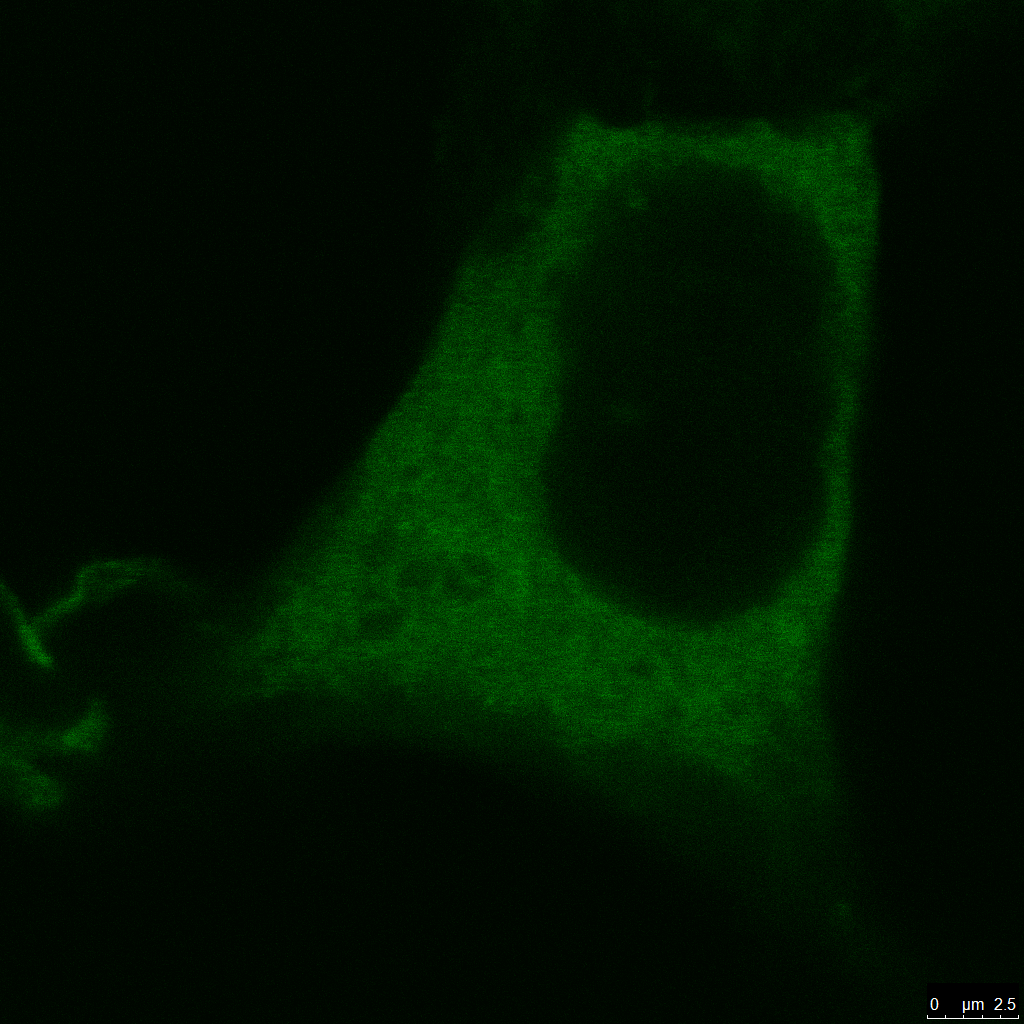

Supplement: Supplementary file 15 — Source Data for Figure 2 [file EMBJ-41-e111289-s008.zip › Microscopy_Confocal/2A/2A-A.tif]

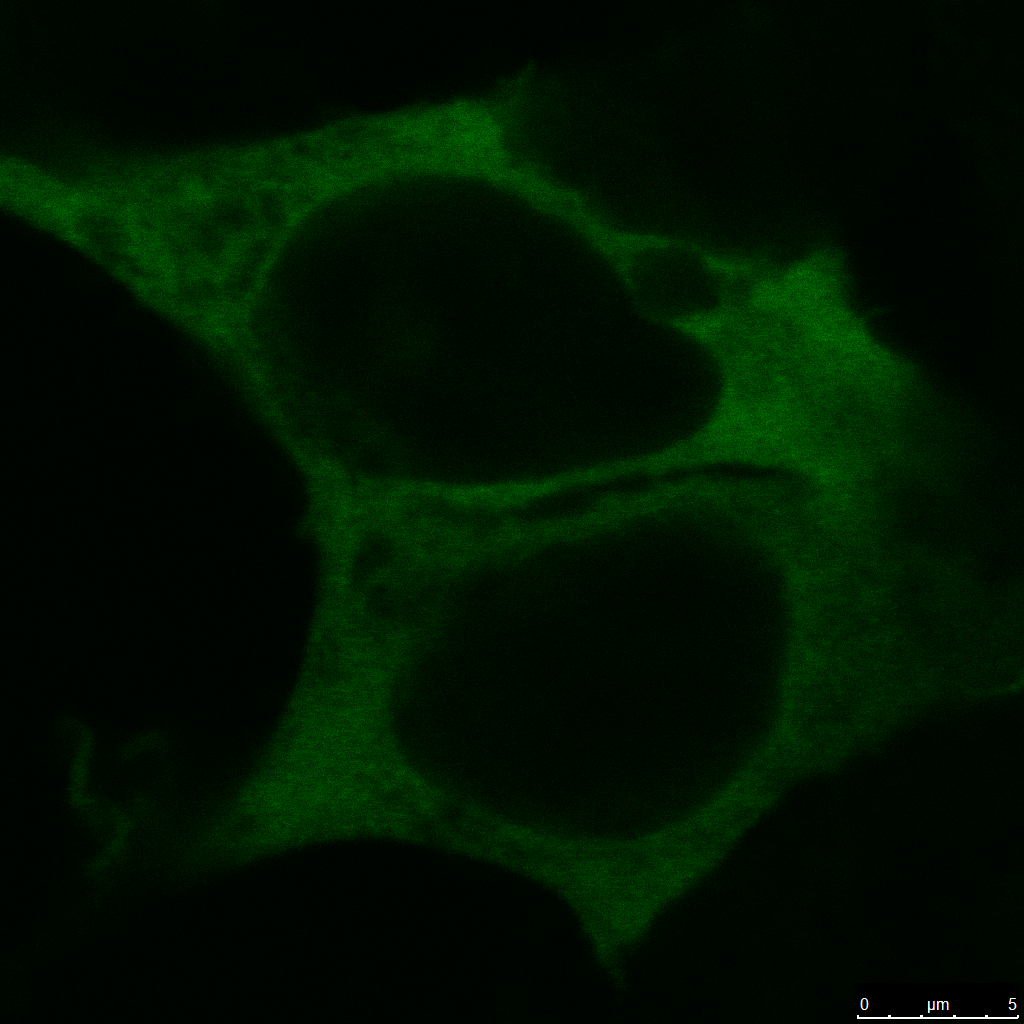

Supplement: Supplementary file 15 — Source Data for Figure 2 [file EMBJ-41-e111289-s008.zip › Microscopy_Confocal/2A/2A-B.tif]

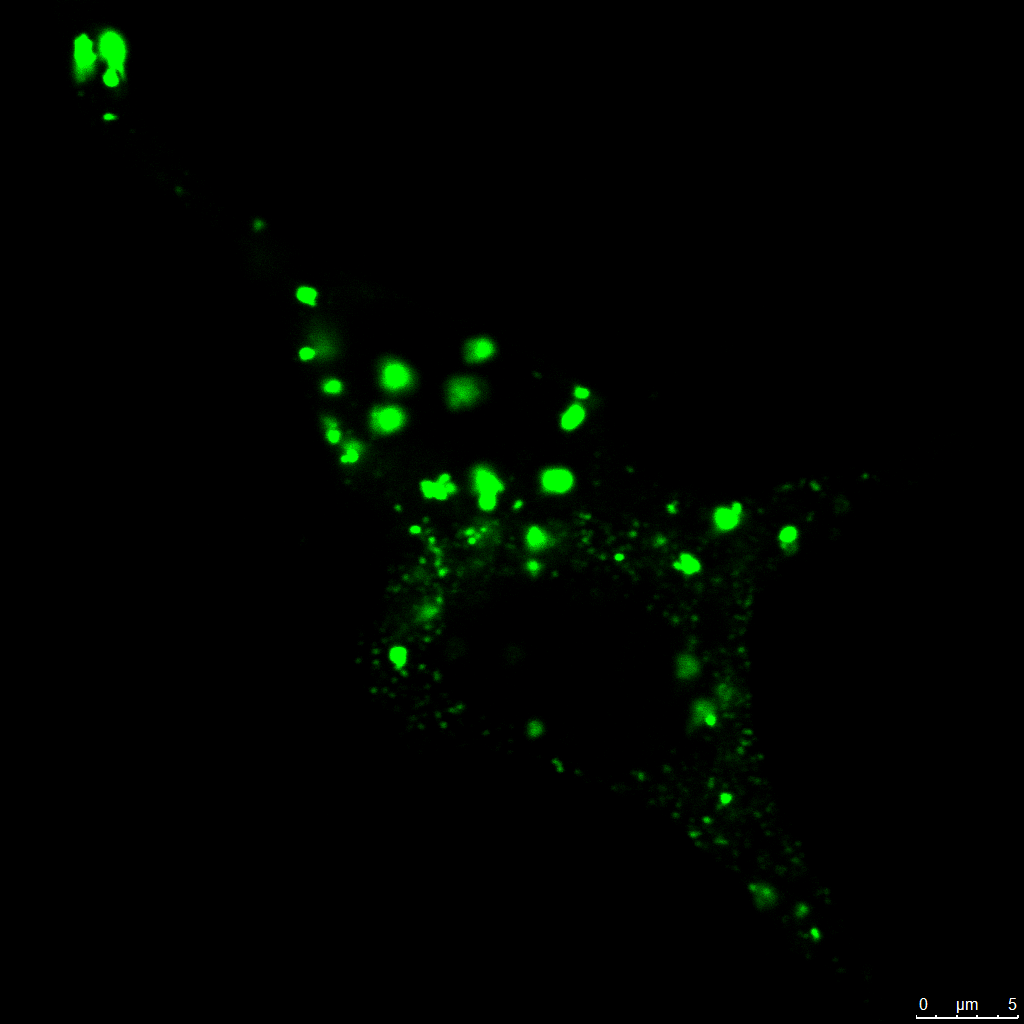

Supplement: Supplementary file 15 — Source Data for Figure 2 [file EMBJ-41-e111289-s008.zip › Microscopy_Confocal/2A/2A-C.tif]

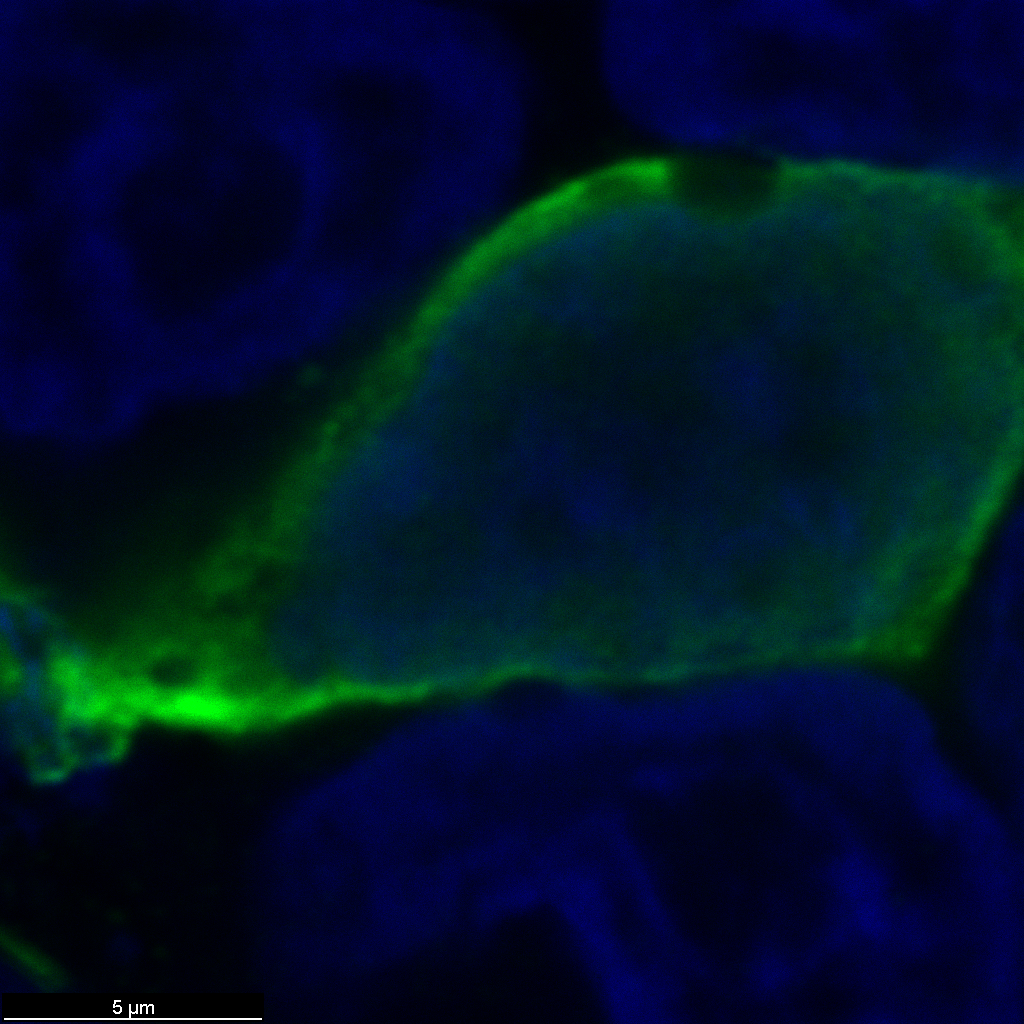

Supplement: Supplementary file 15 — Source Data for Figure 2 [file EMBJ-41-e111289-s008.zip › Microscopy_Confocal/2D/2D-A.tif]

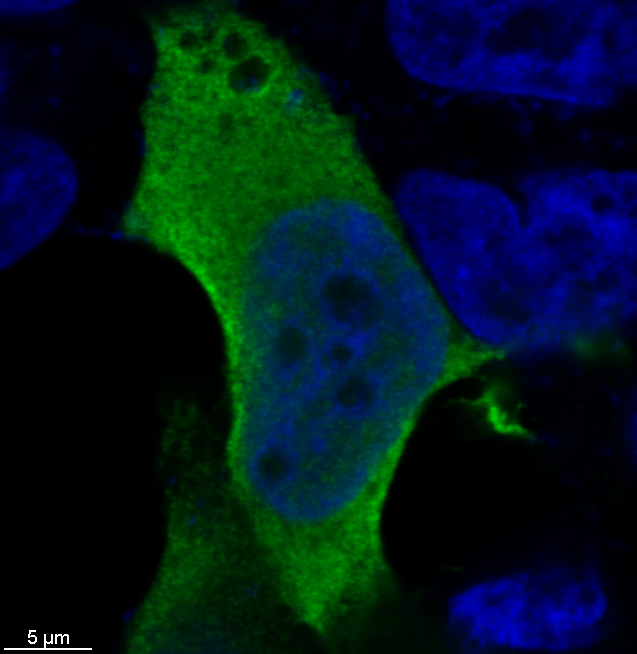

Supplement: Supplementary file 15 — Source Data for Figure 2 [file EMBJ-41-e111289-s008.zip › Microscopy_Confocal/2D/2D-B.tif]

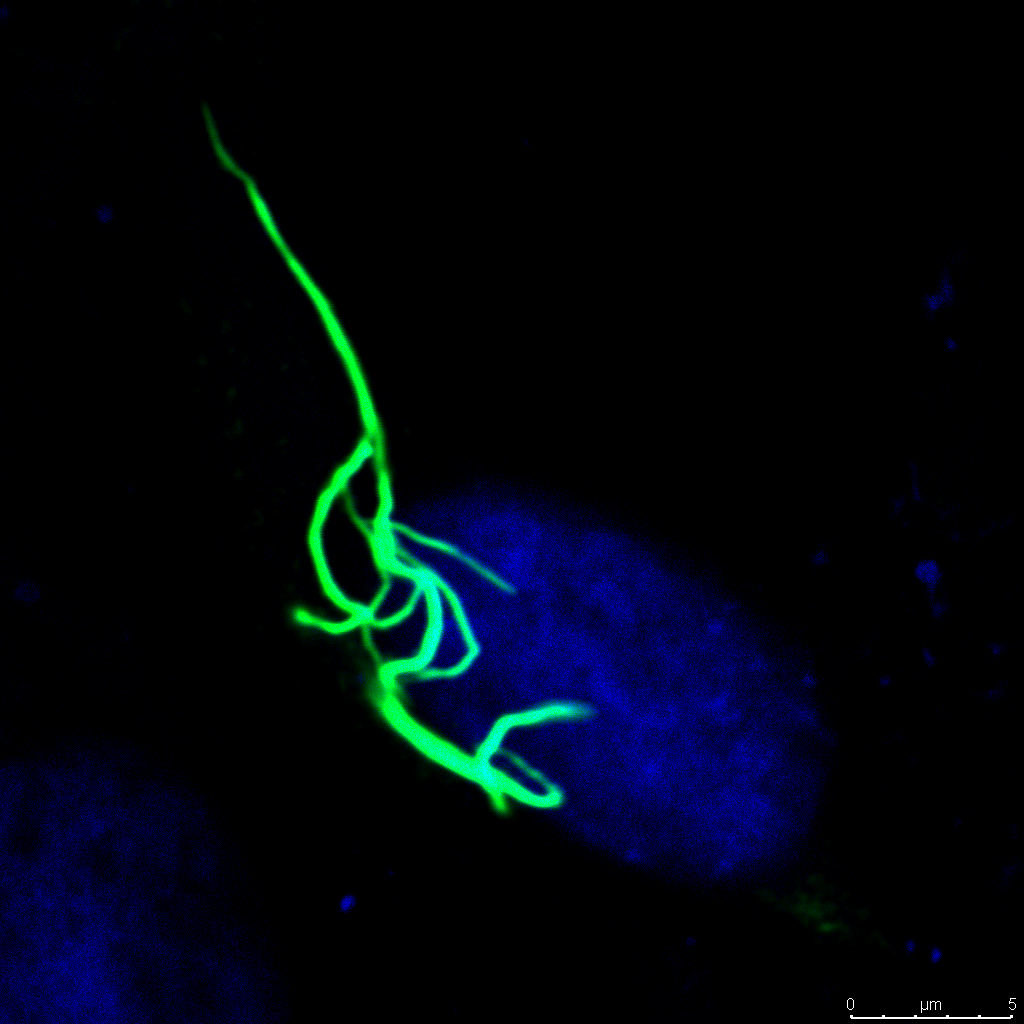

Supplement: Supplementary file 15 — Source Data for Figure 2 [file EMBJ-41-e111289-s008.zip › Microscopy_Confocal/2D/2D-C.tif]

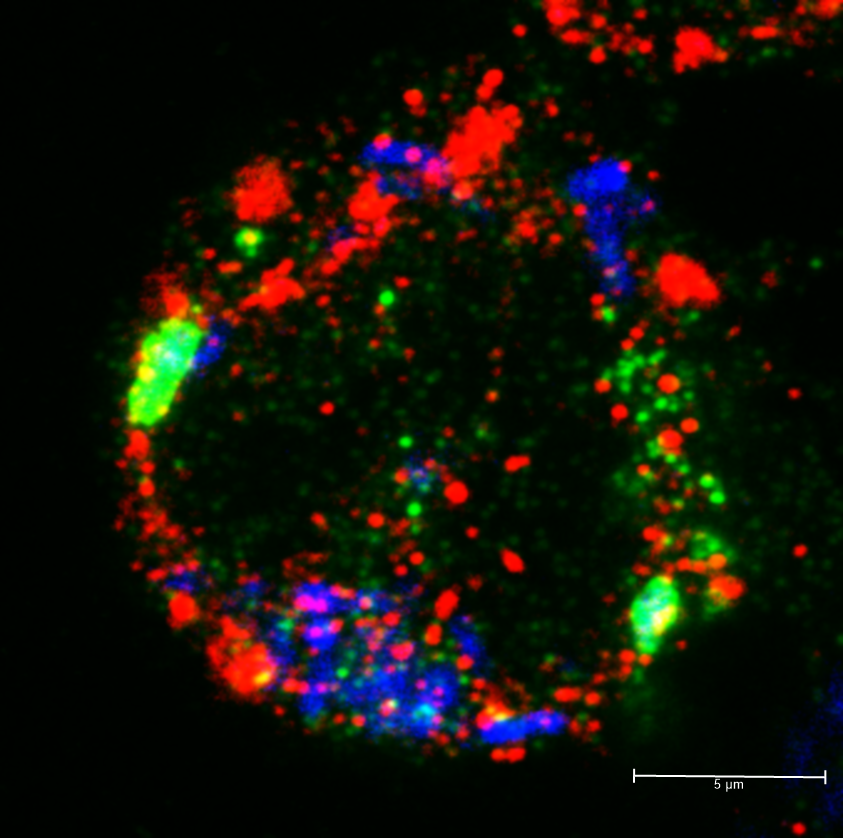

Supplement: Supplementary file 15 — Source Data for Figure 2 [file EMBJ-41-e111289-s008.zip › Microscopy_Confocal/2F/2F-A.tif]

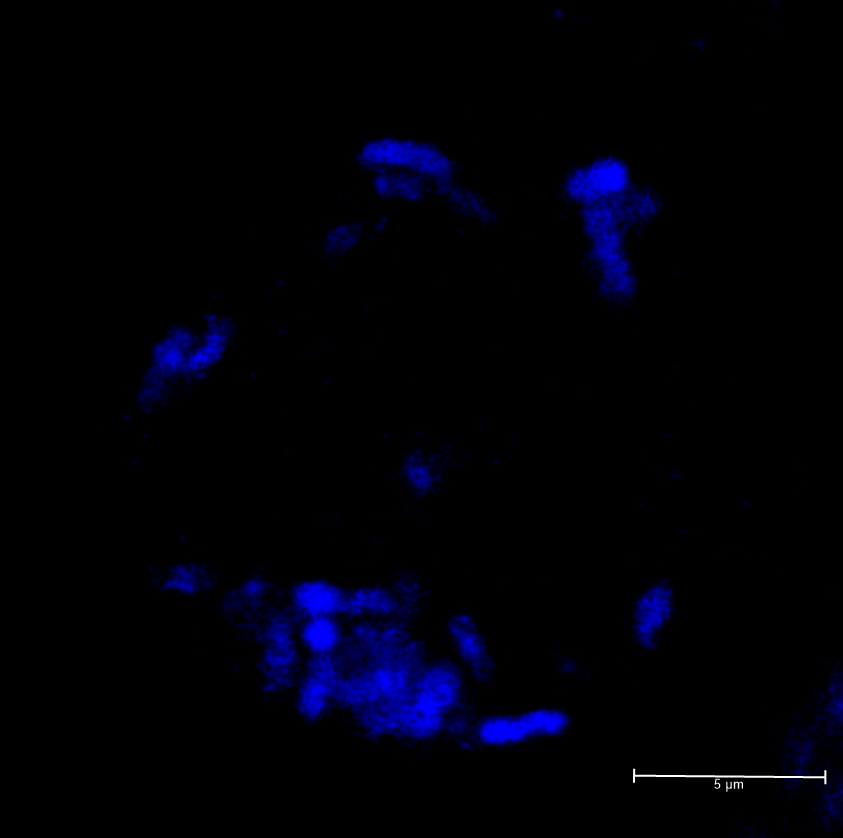

Supplement: Supplementary file 15 — Source Data for Figure 2 [file EMBJ-41-e111289-s008.zip › Microscopy_Confocal/2F/2F-B.tif]

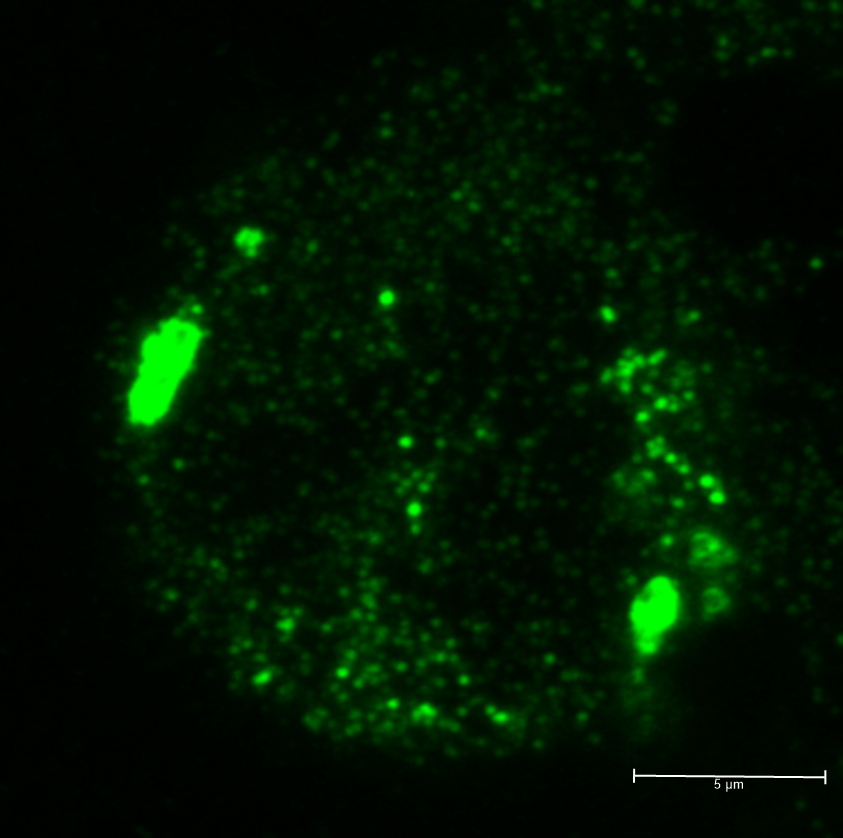

Supplement: Supplementary file 15 — Source Data for Figure 2 [file EMBJ-41-e111289-s008.zip › Microscopy_Confocal/2F/2F-C.tif]

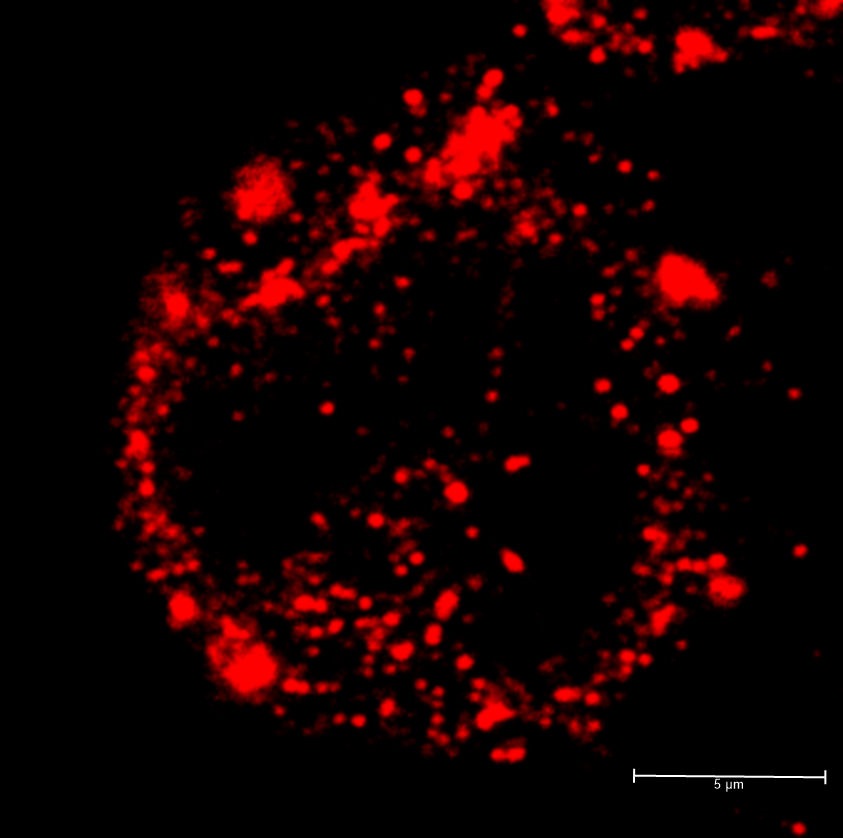

Supplement: Supplementary file 15 — Source Data for Figure 2 [file EMBJ-41-e111289-s008.zip › Microscopy_Confocal/2F/2F-D.tif]

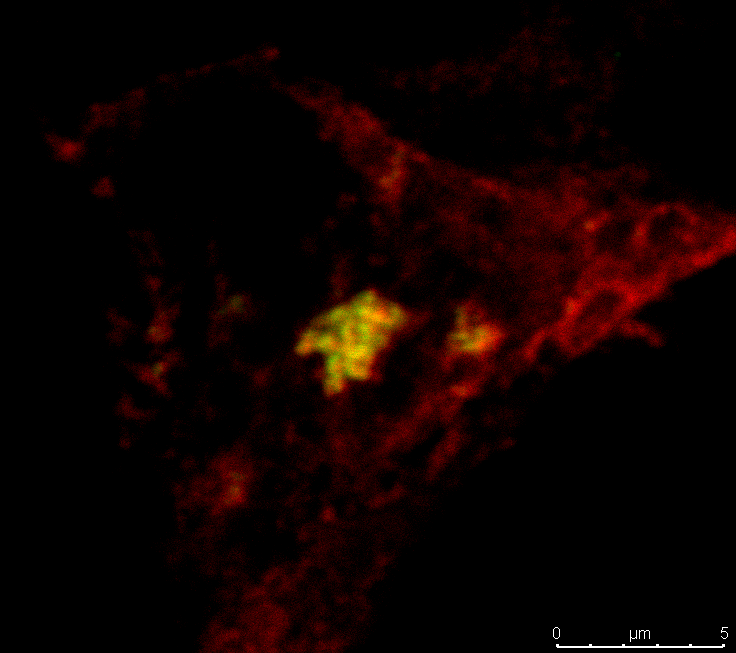

Supplement: Supplementary file 15 — Source Data for Figure 2 [file EMBJ-41-e111289-s008.zip › Microscopy_Confocal/2G/2G-A.tif]

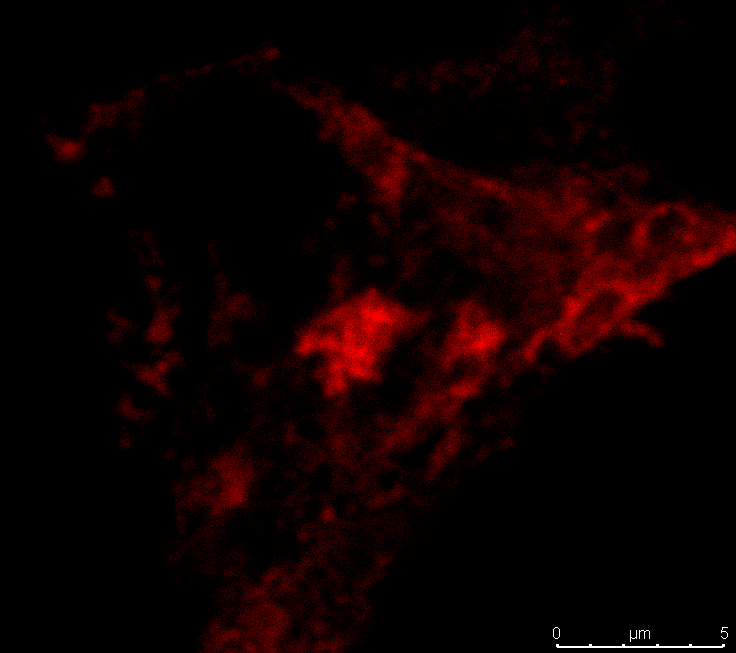

Supplement: Supplementary file 15 — Source Data for Figure 2 [file EMBJ-41-e111289-s008.zip › Microscopy_Confocal/2G/2G-B.tif]

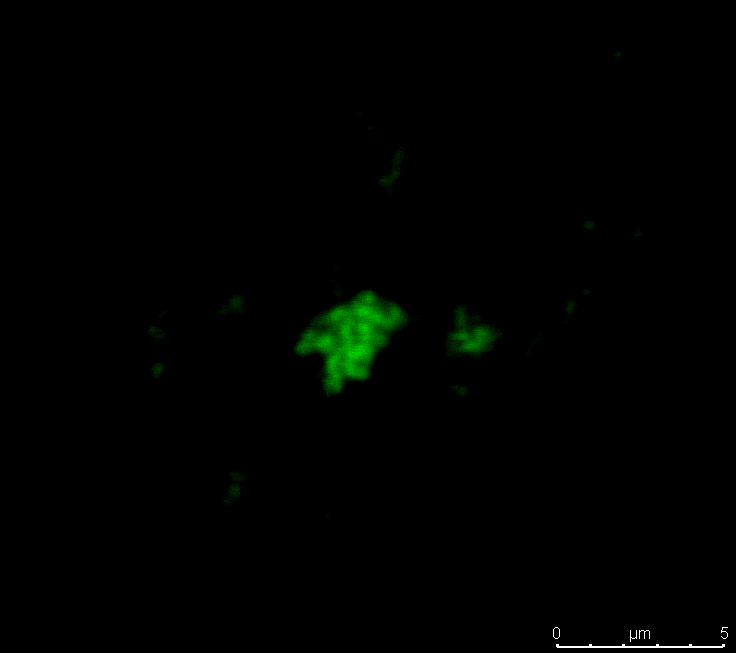

Supplement: Supplementary file 15 — Source Data for Figure 2 [file EMBJ-41-e111289-s008.zip › Microscopy_Confocal/2G/2G-C.tif]

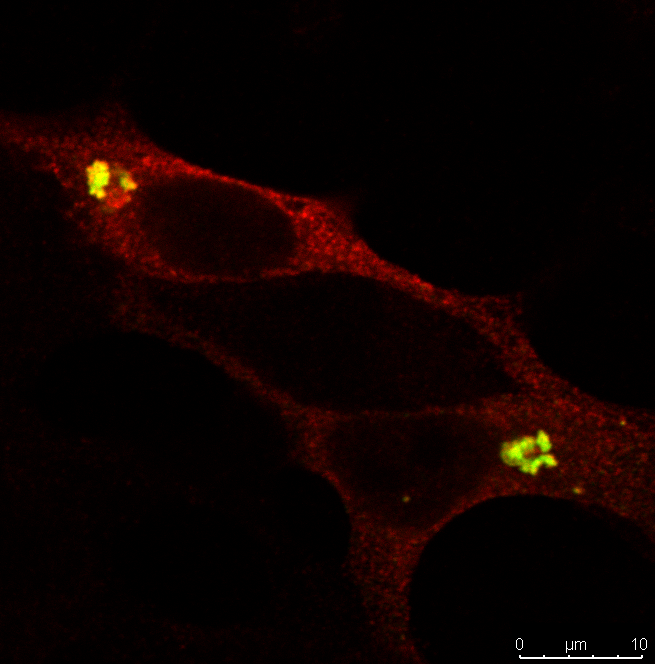

Supplement: Supplementary file 15 — Source Data for Figure 2 [file EMBJ-41-e111289-s008.zip › Microscopy_Confocal/2G/2G-D.tif]

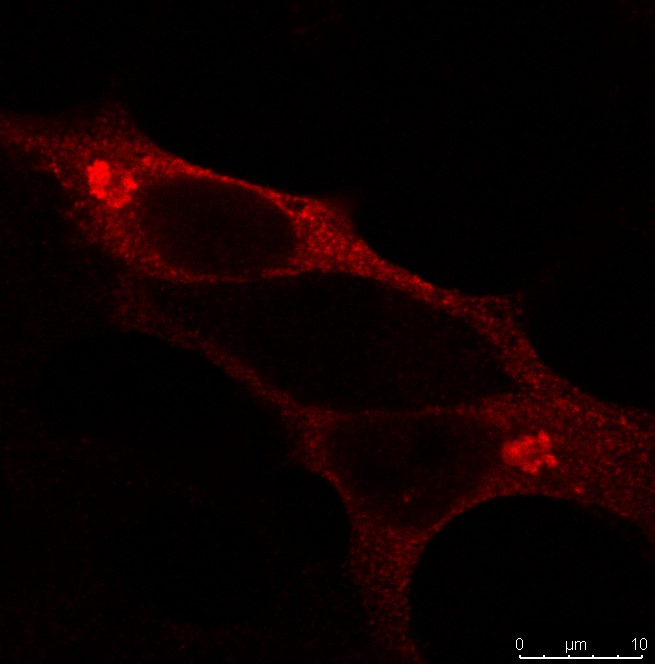

Supplement: Supplementary file 15 — Source Data for Figure 2 [file EMBJ-41-e111289-s008.zip › Microscopy_Confocal/2G/2G-E.tif]

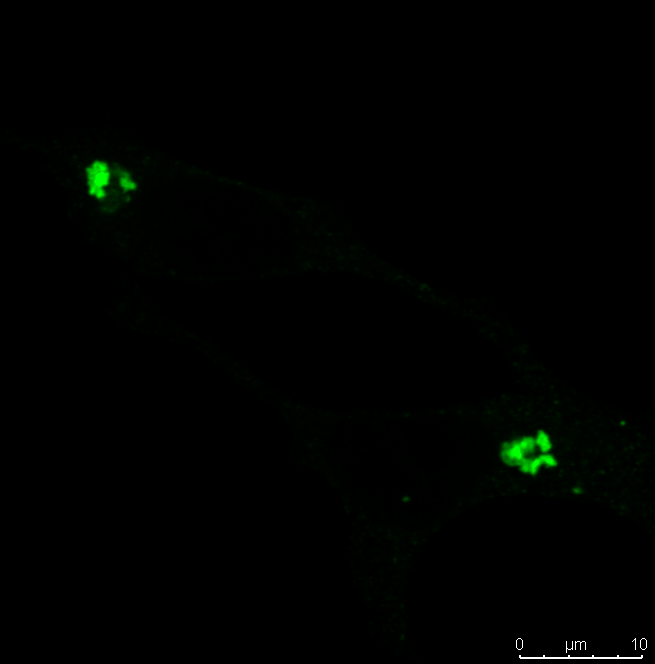

Supplement: Supplementary file 15 — Source Data for Figure 2 [file EMBJ-41-e111289-s008.zip › Microscopy_Confocal/2G/2G-F.tif]

Figure-2

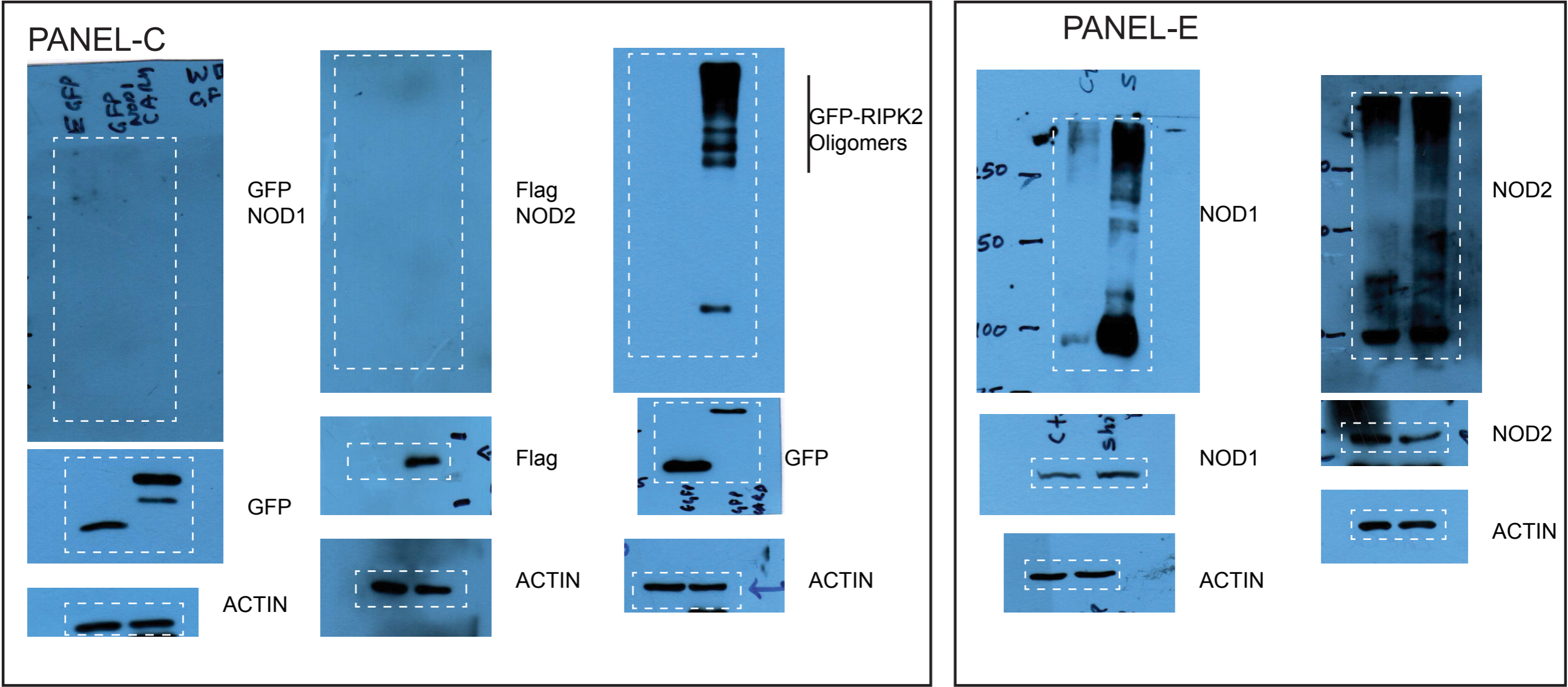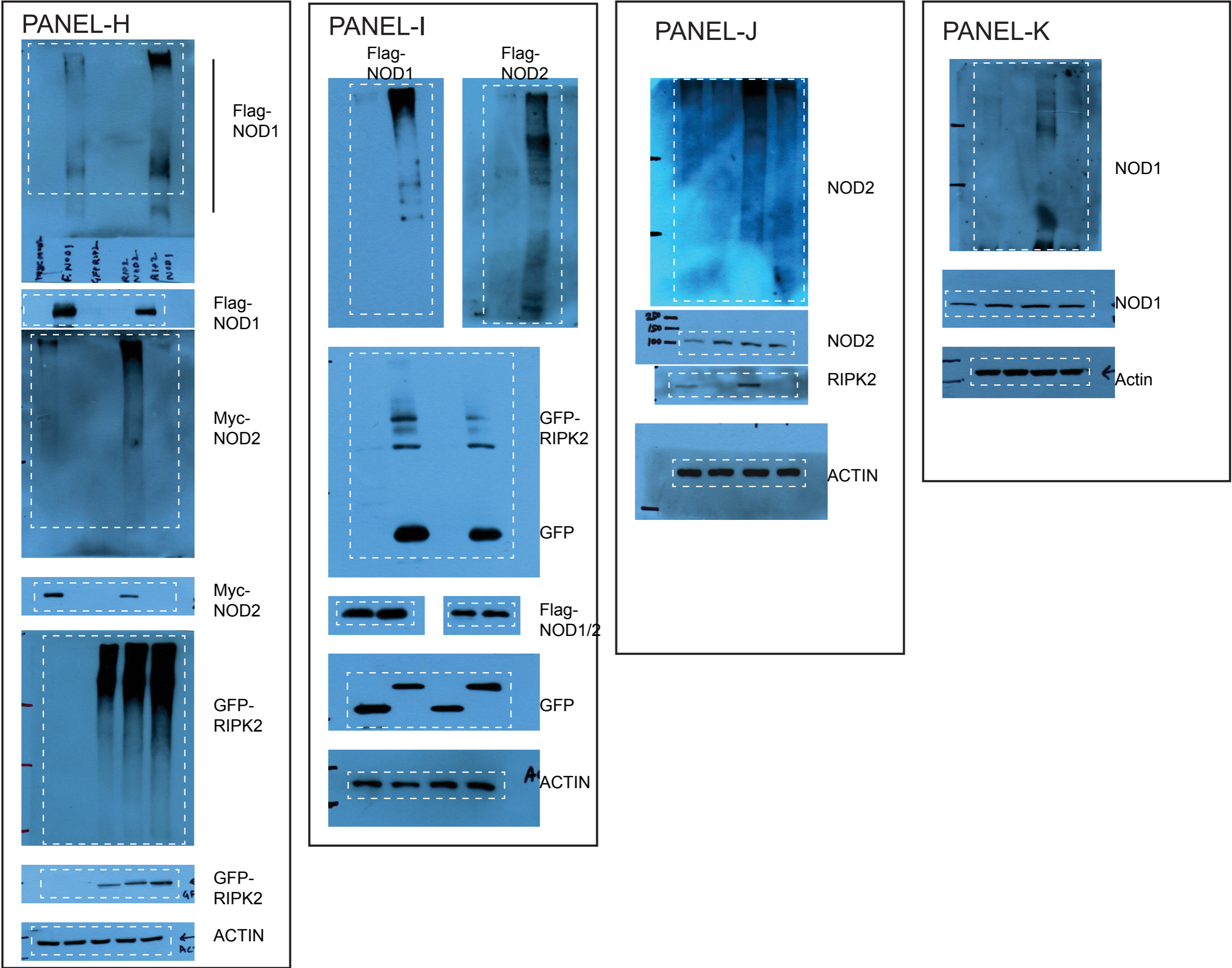

Supplement: Supplementary file 15 — Source Data for Figure 2 [file EMBJ-41-e111289-s008.zip › Western Blot/Western Blot Figure-2.pdf]

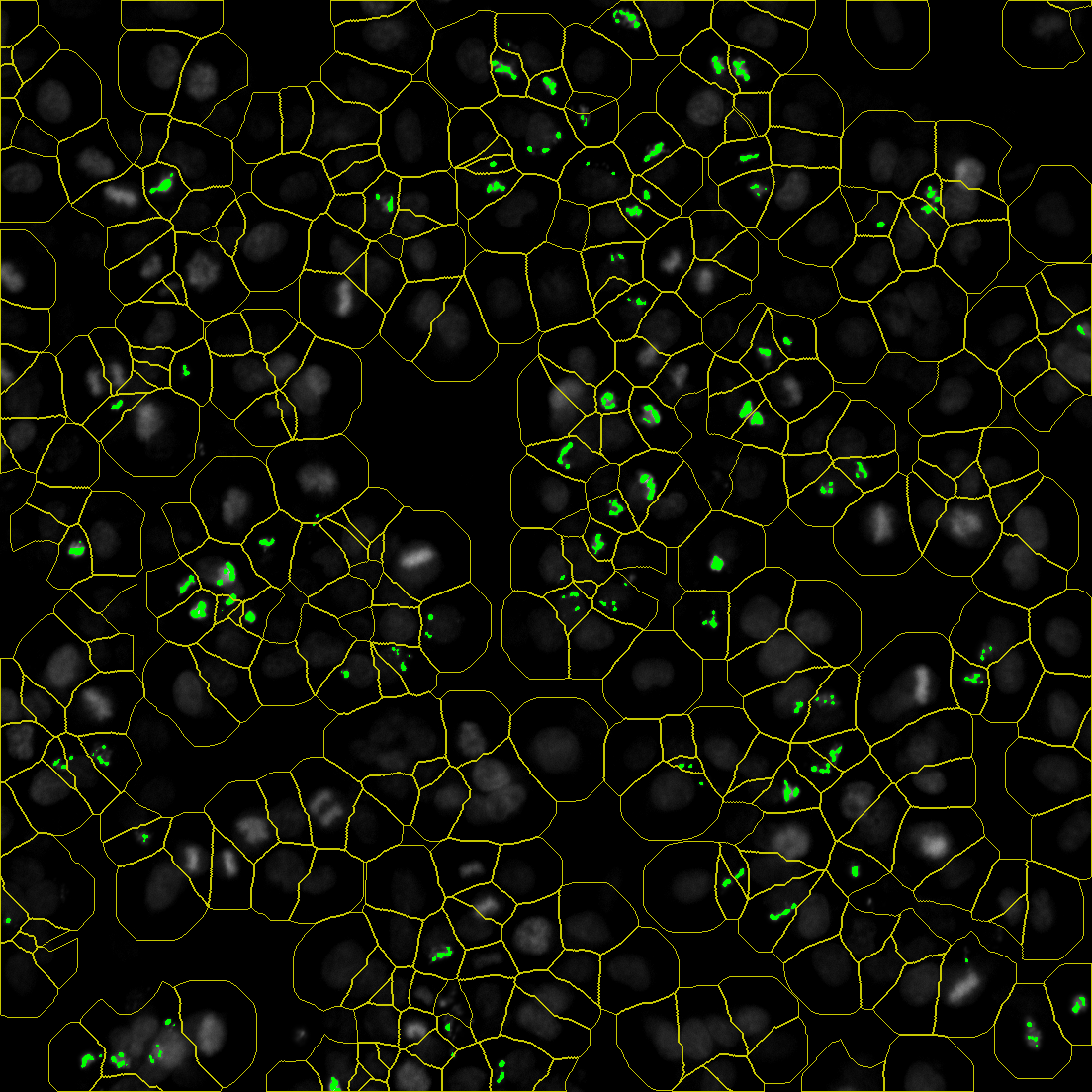

Supplement: Supplementary file 16 — Source Data for Figure 3 [file EMBJ-41-e111289-s017.zip › High Content Screening/3C/3C_Control.bmp]

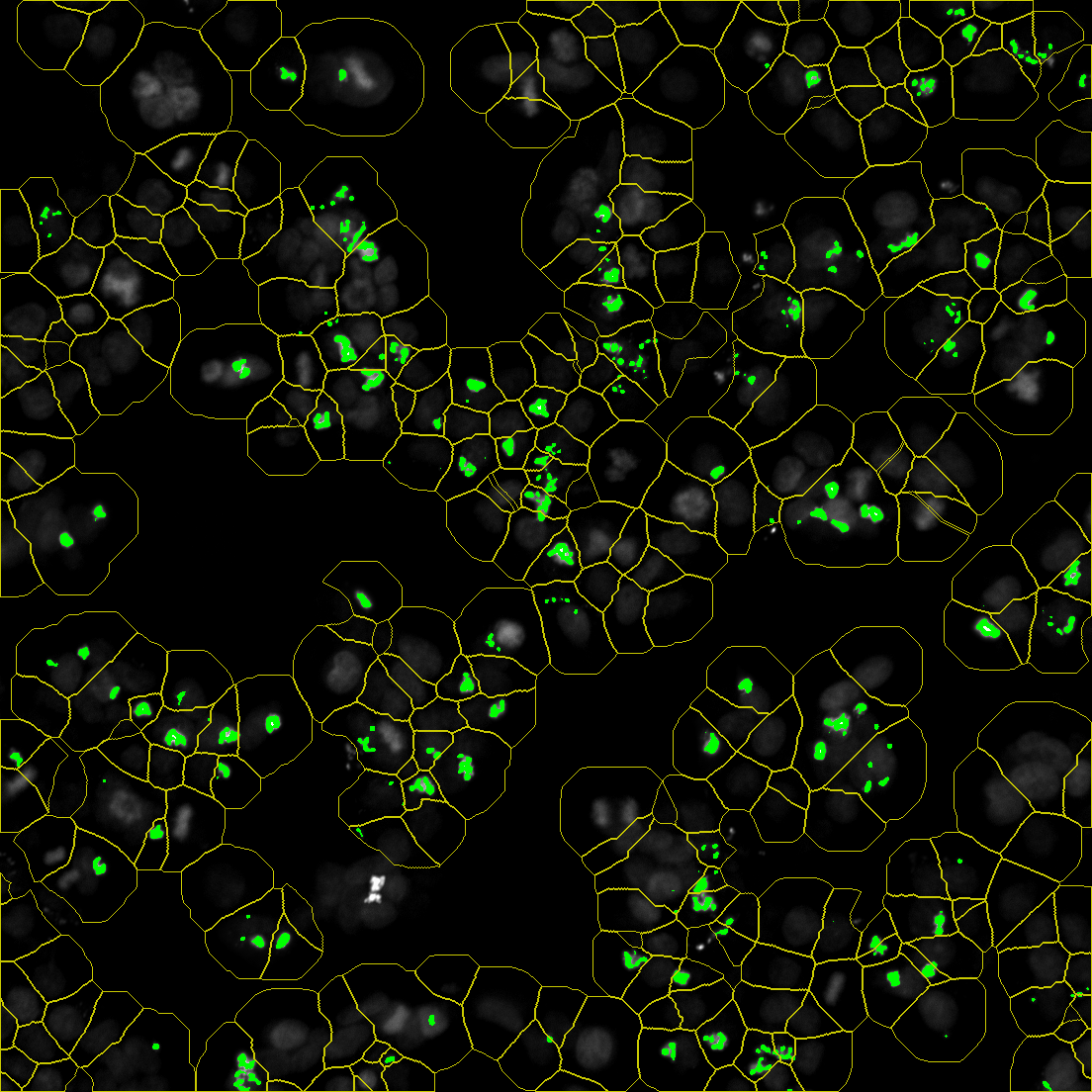

Supplement: Supplementary file 16 — Source Data for Figure 3 [file EMBJ-41-e111289-s017.zip › High Content Screening/3C/3C_Rapamycin+BafA1.bmp]

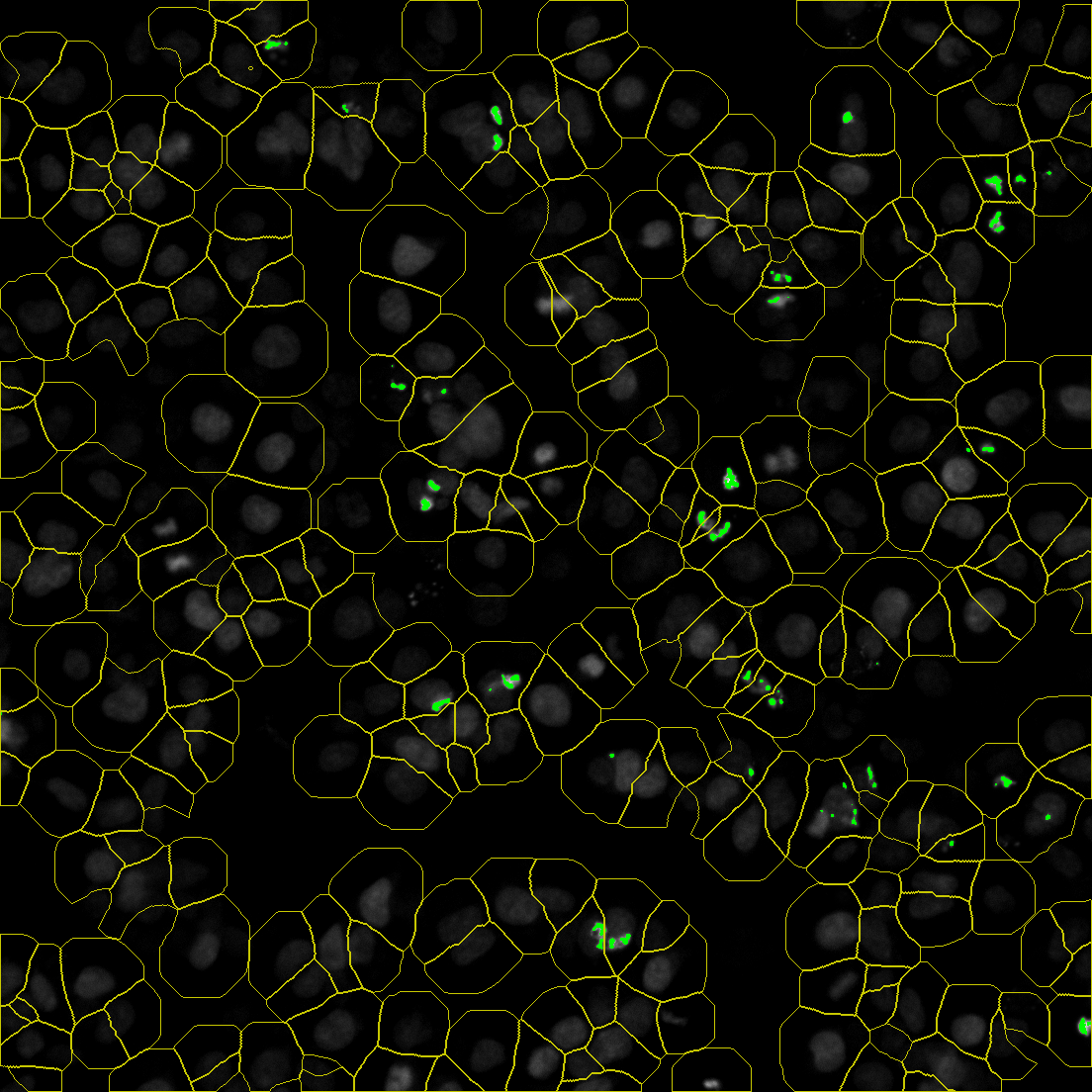

Supplement: Supplementary file 16 — Source Data for Figure 3 [file EMBJ-41-e111289-s017.zip › High Content Screening/3C/3C_Rapamycin.bmp]

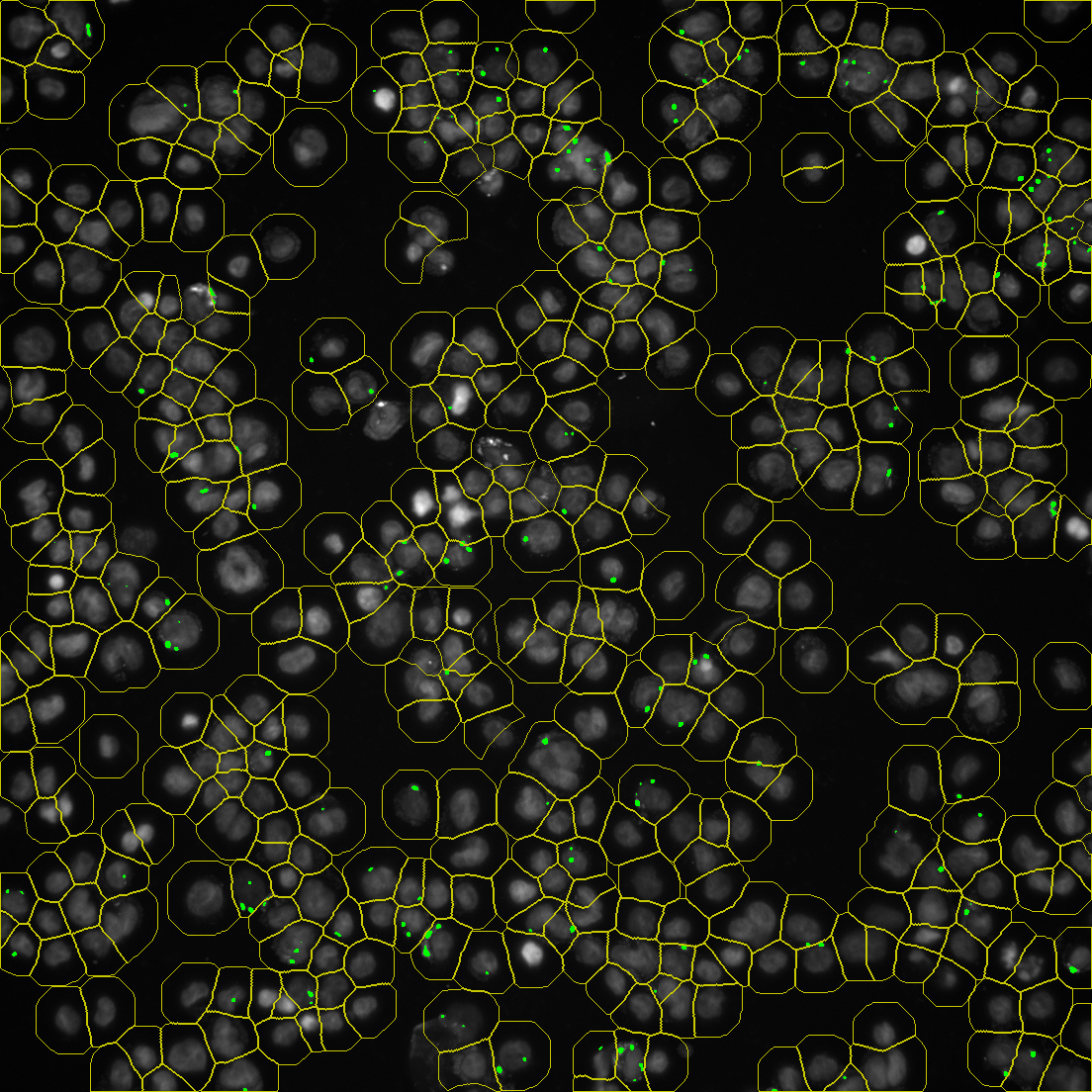

Supplement: Supplementary file 16 — Source Data for Figure 3 [file EMBJ-41-e111289-s017.zip › High Content Screening/3D/3D_siRNA-ATG5.bmp]

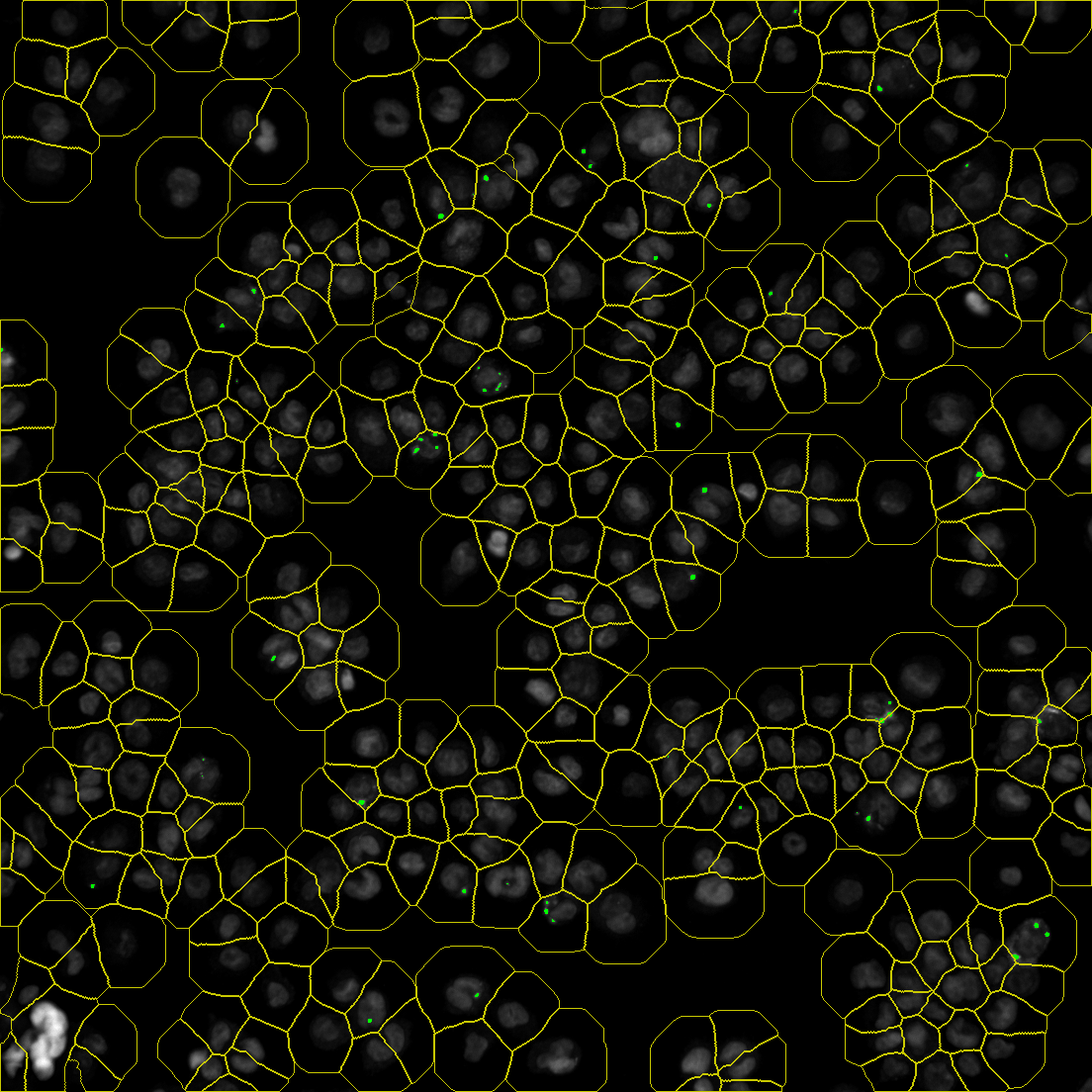

Supplement: Supplementary file 16 — Source Data for Figure 3 [file EMBJ-41-e111289-s017.zip › High Content Screening/3D/3D_siRNA-control.bmp]

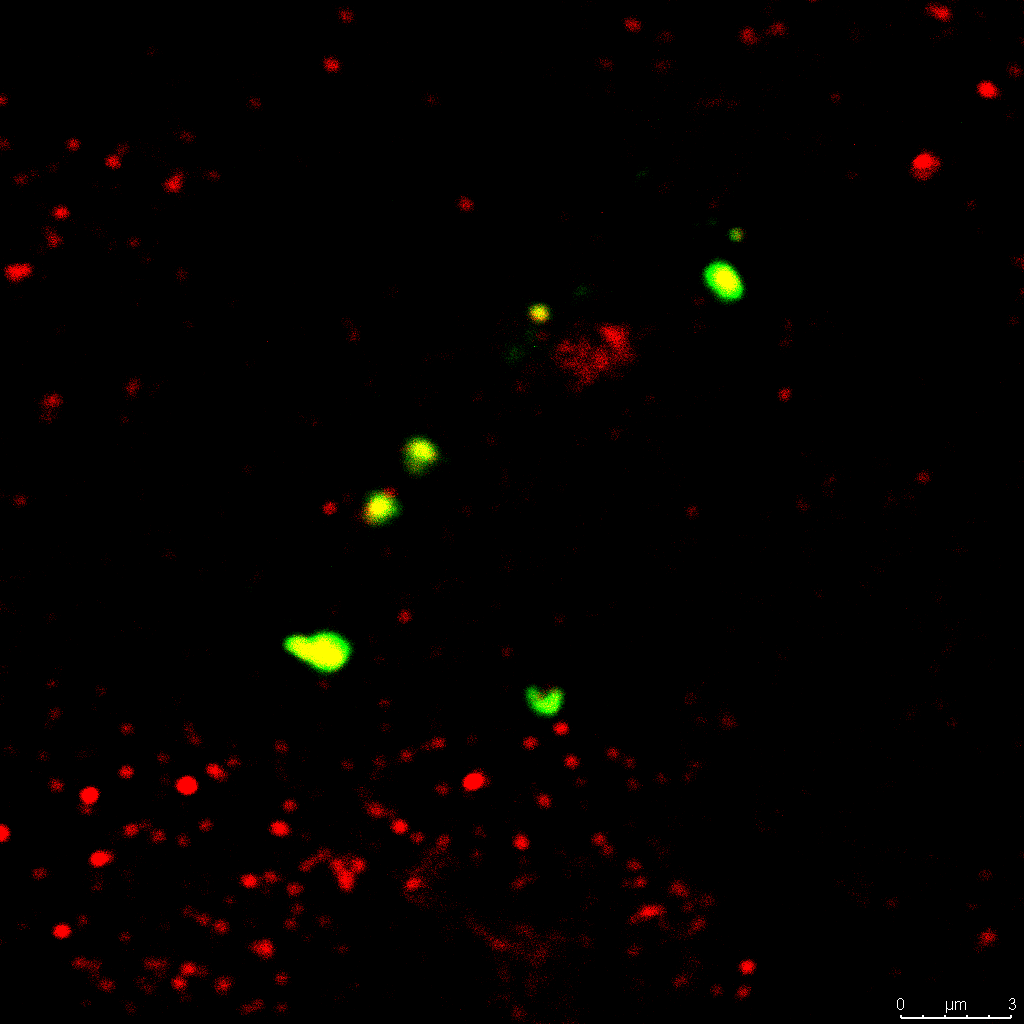

Supplement: Supplementary file 16 — Source Data for Figure 3 [file EMBJ-41-e111289-s017.zip › Microscopy_Confocal/3F/3F-A.tif]

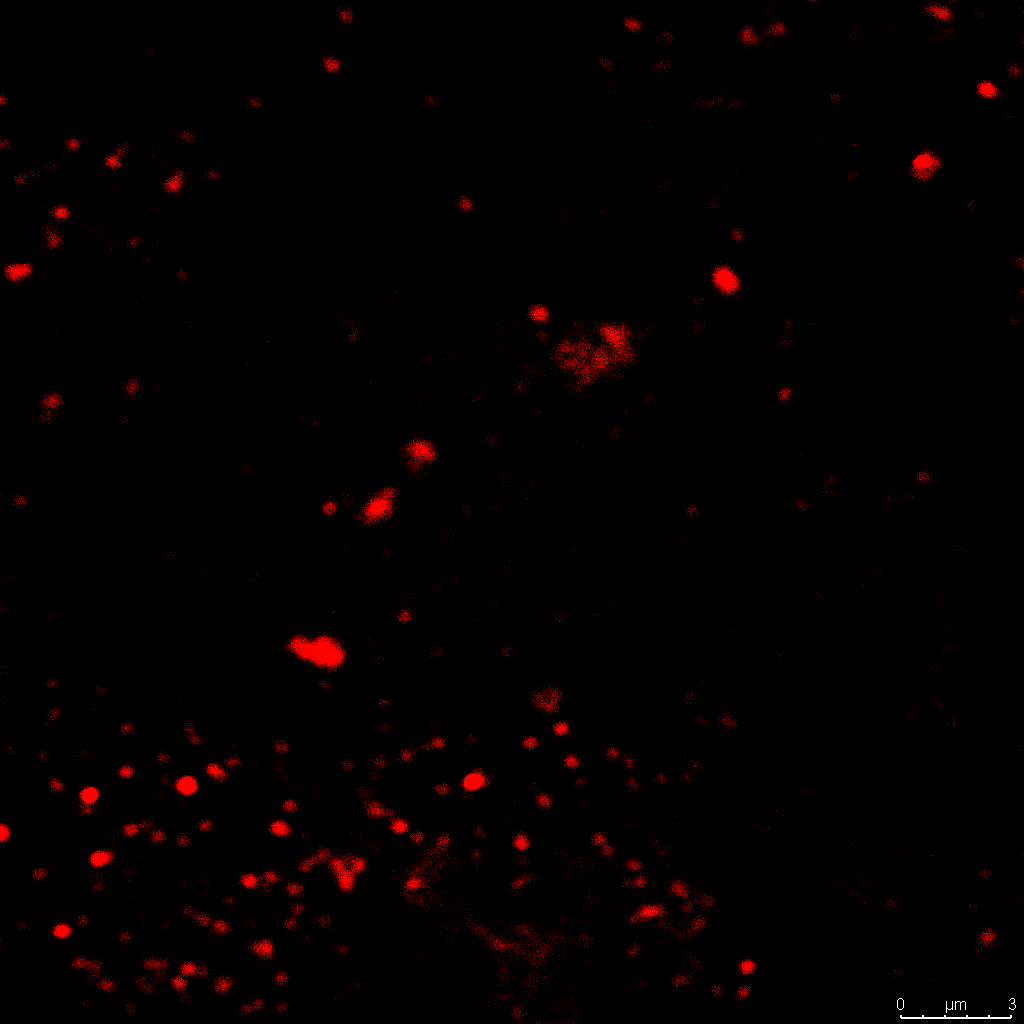

Supplement: Supplementary file 16 — Source Data for Figure 3 [file EMBJ-41-e111289-s017.zip › Microscopy_Confocal/3F/3F-B.tif]

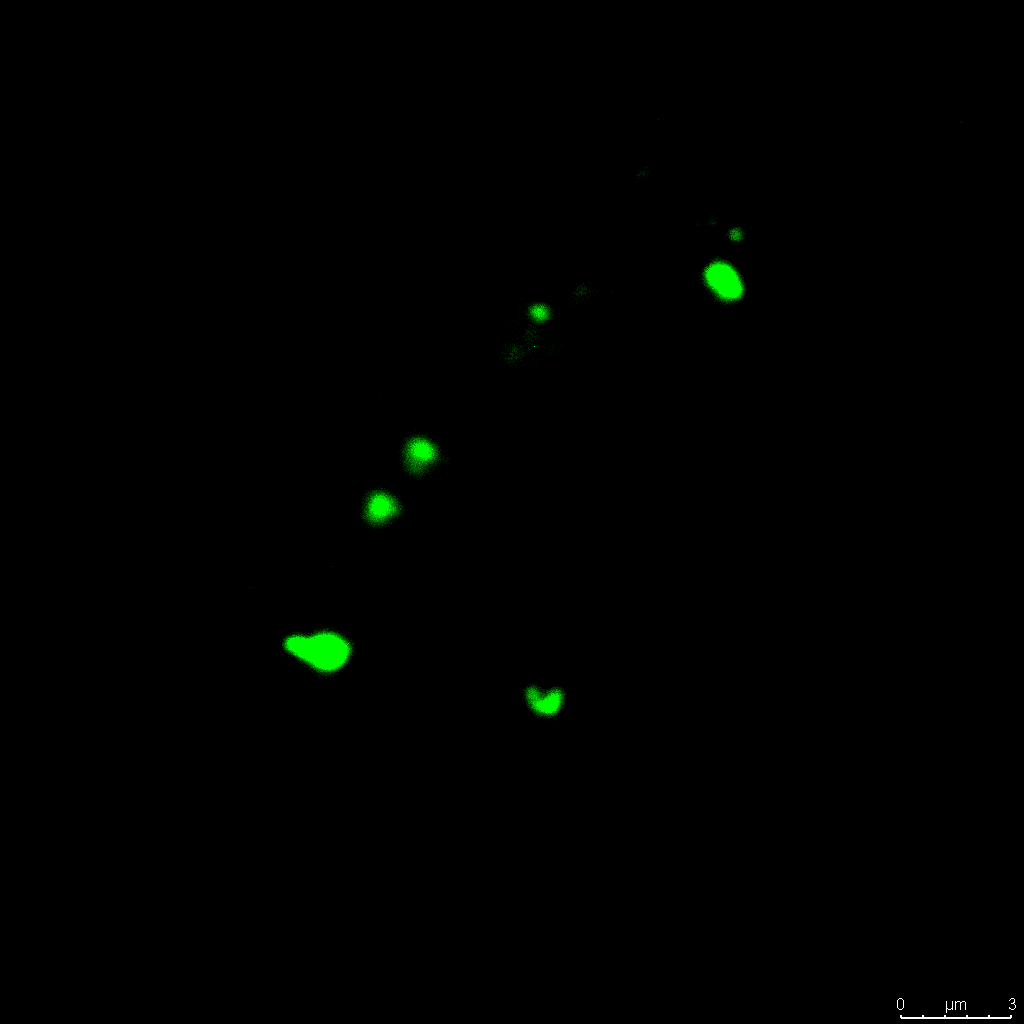

Supplement: Supplementary file 16 — Source Data for Figure 3 [file EMBJ-41-e111289-s017.zip › Microscopy_Confocal/3F/3F-C.tif]

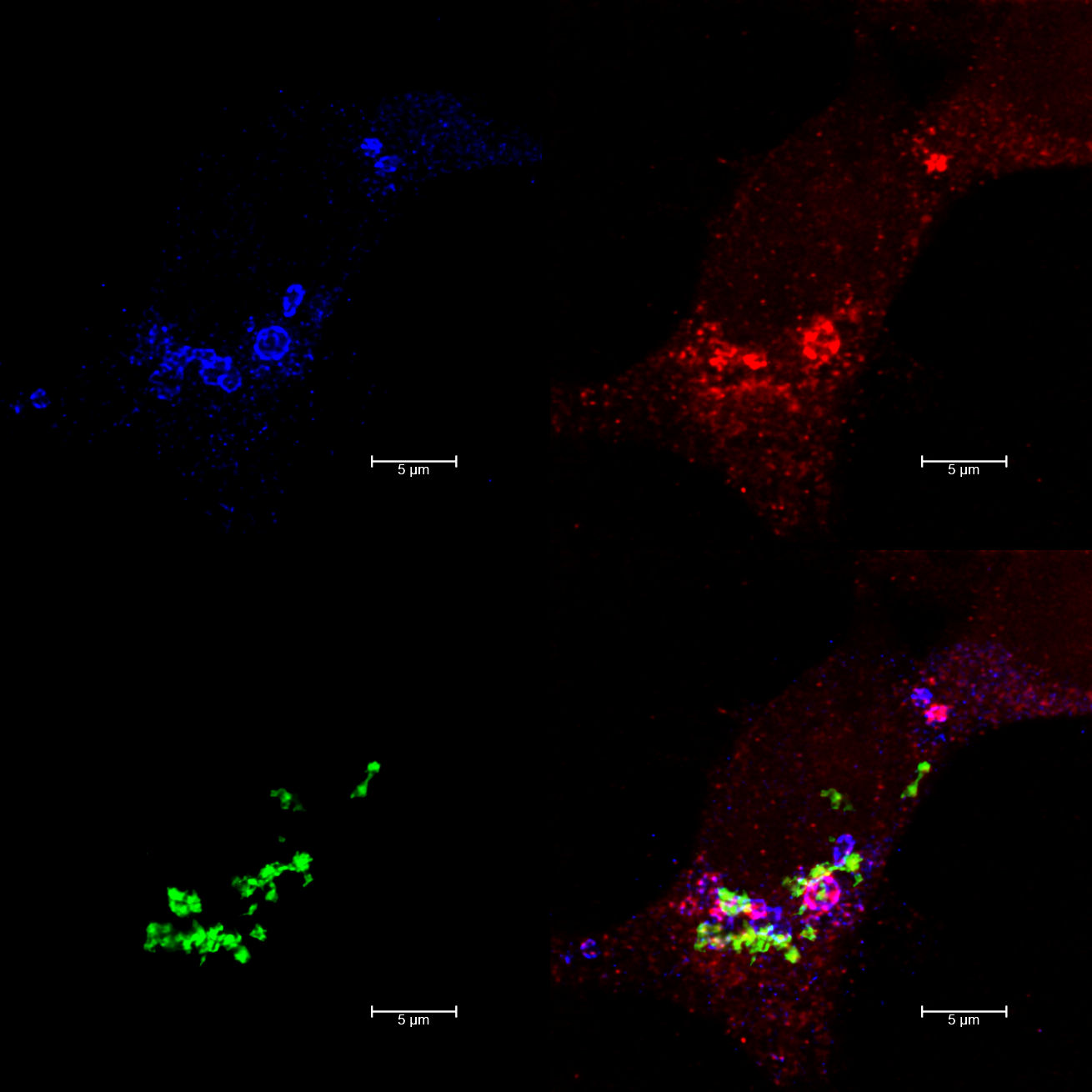

Supplement: Supplementary file 16 — Source Data for Figure 3 [file EMBJ-41-e111289-s017.zip › Microscopy_Confocal/3I/3I-A.tif]

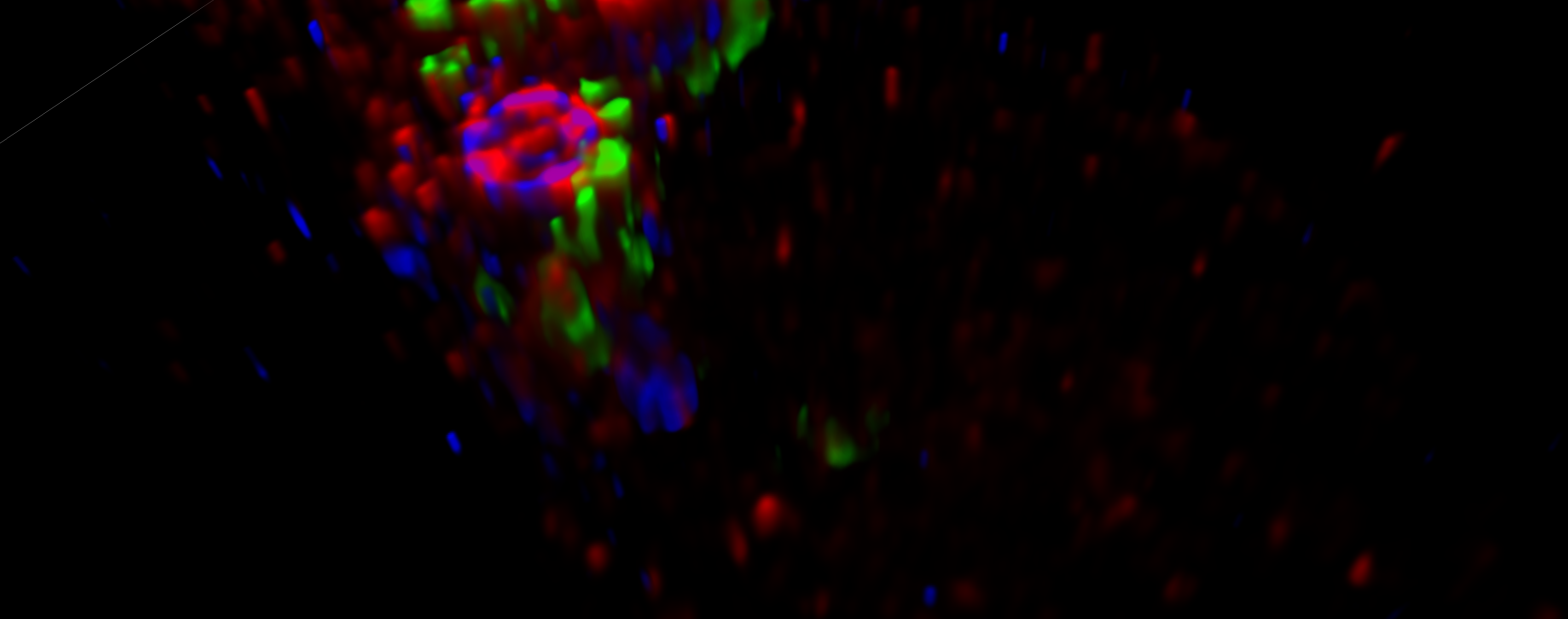

Supplement: Supplementary file 16 — Source Data for Figure 3 [file EMBJ-41-e111289-s017.zip › Microscopy_Confocal/3I/3I-B.tif]

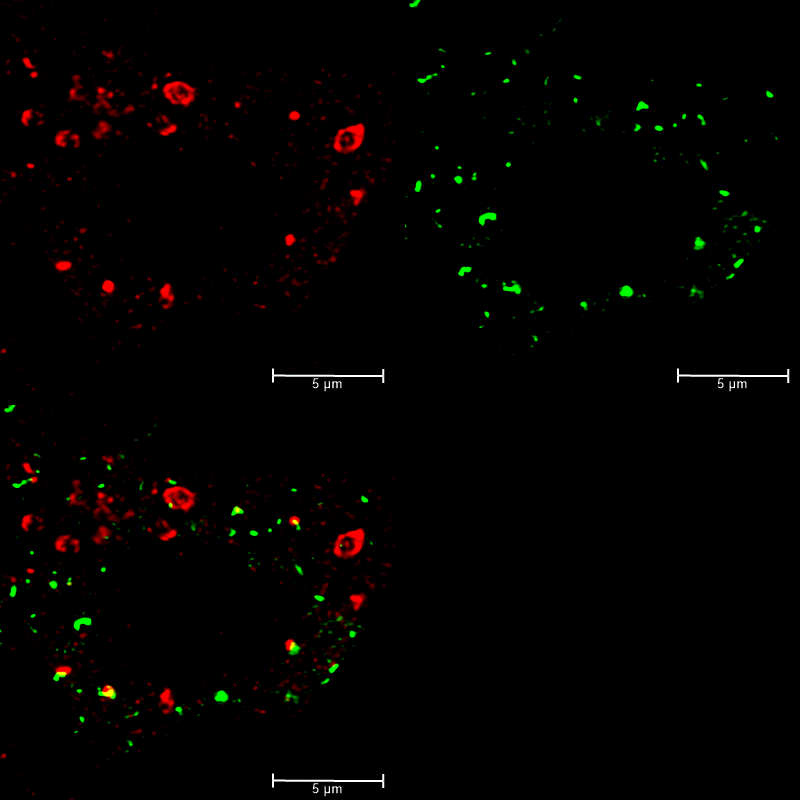

Supplement: Supplementary file 16 — Source Data for Figure 3 [file EMBJ-41-e111289-s017.zip › Microscopy_Confocal/3K/3K-A.tif]

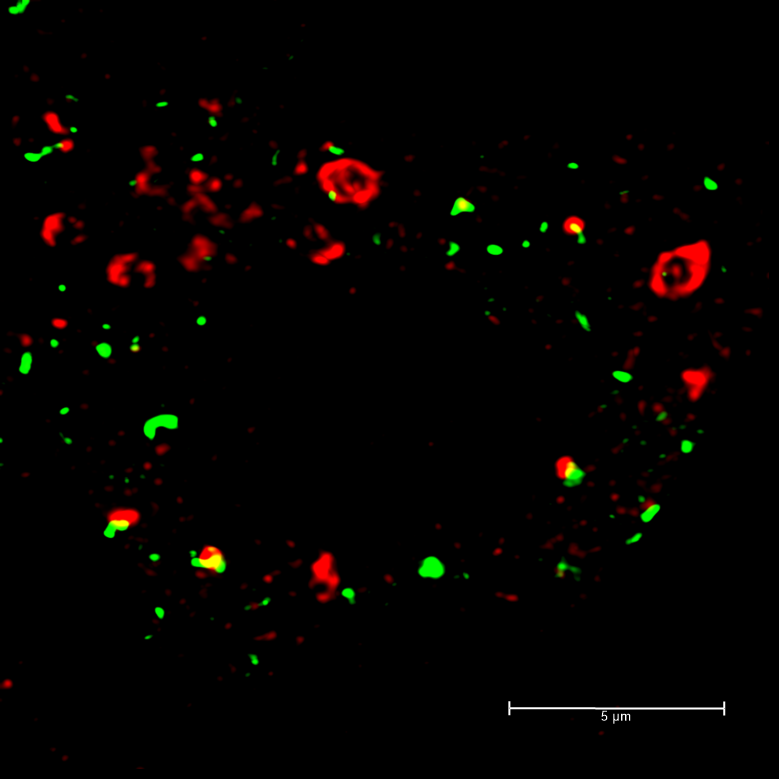

Supplement: Supplementary file 16 — Source Data for Figure 3 [file EMBJ-41-e111289-s017.zip › Microscopy_Confocal/3K/3K-B.tif]

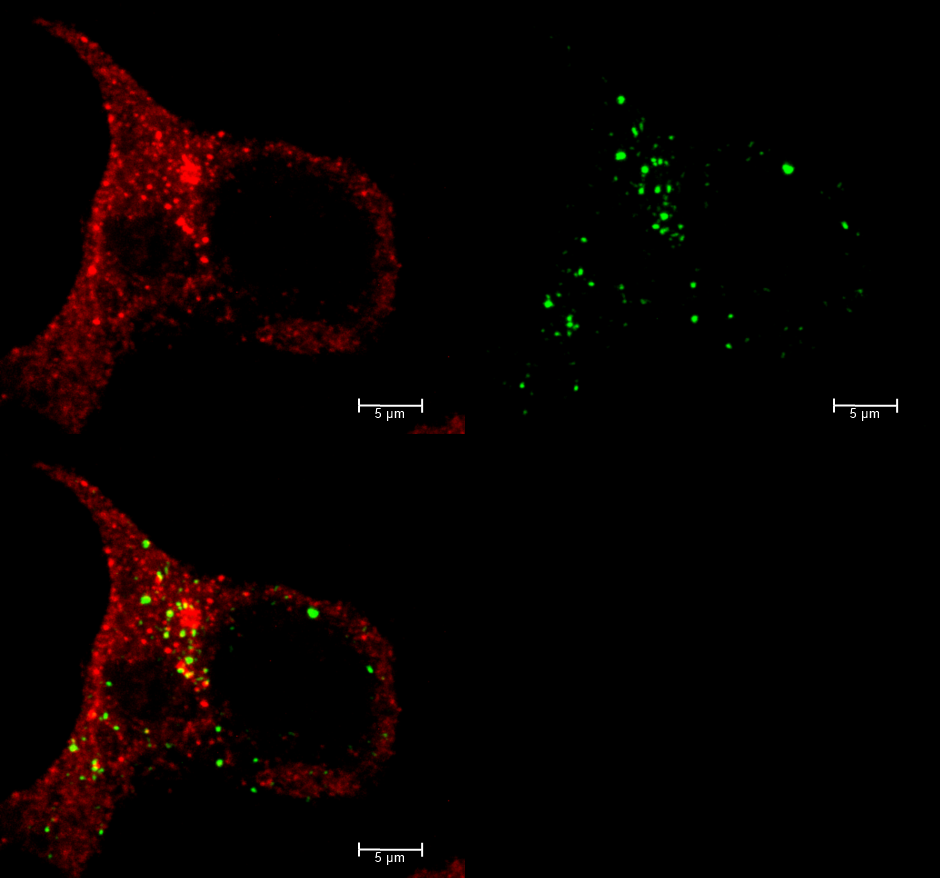

Supplement: Supplementary file 16 — Source Data for Figure 3 [file EMBJ-41-e111289-s017.zip › Microscopy_Confocal/3L/3L.tif]

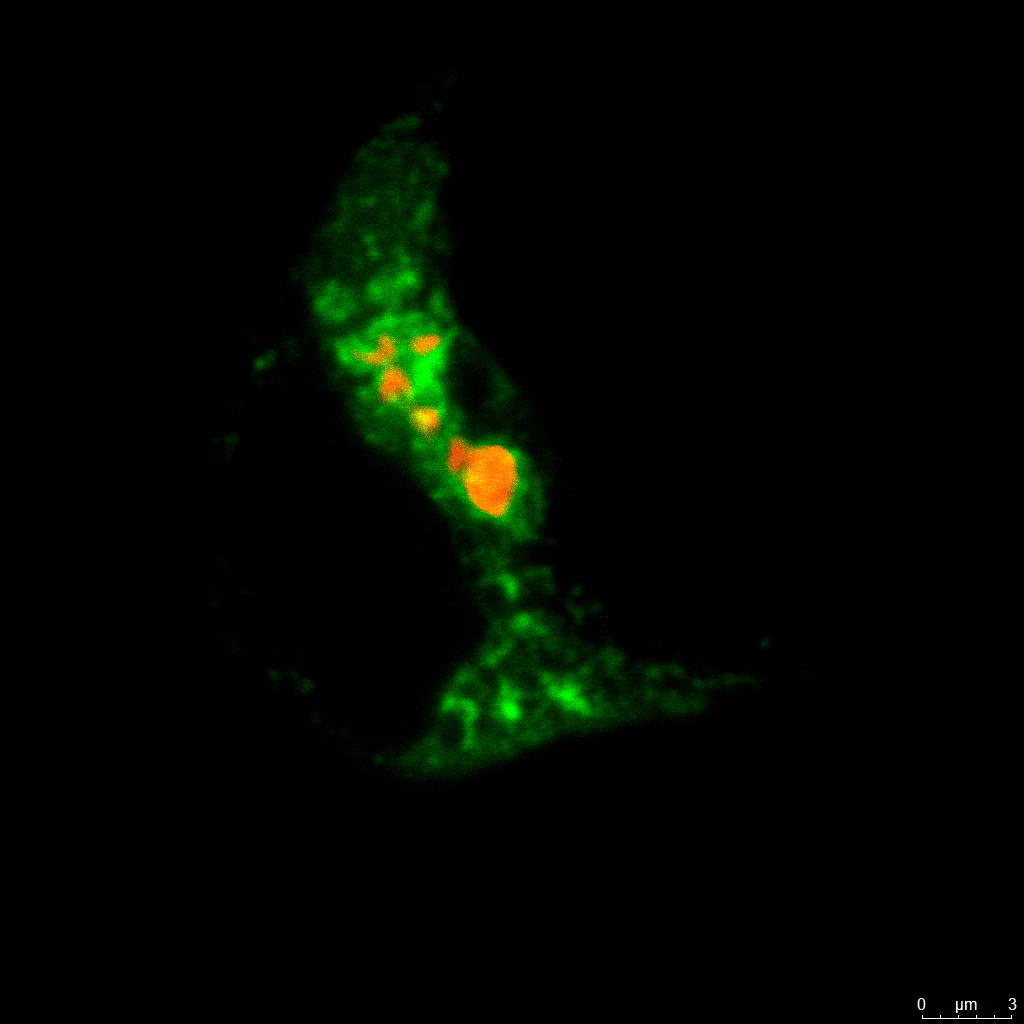

Supplement: Supplementary file 16 — Source Data for Figure 3 [file EMBJ-41-e111289-s017.zip › Microscopy_Confocal/3M/3M-A.tif]

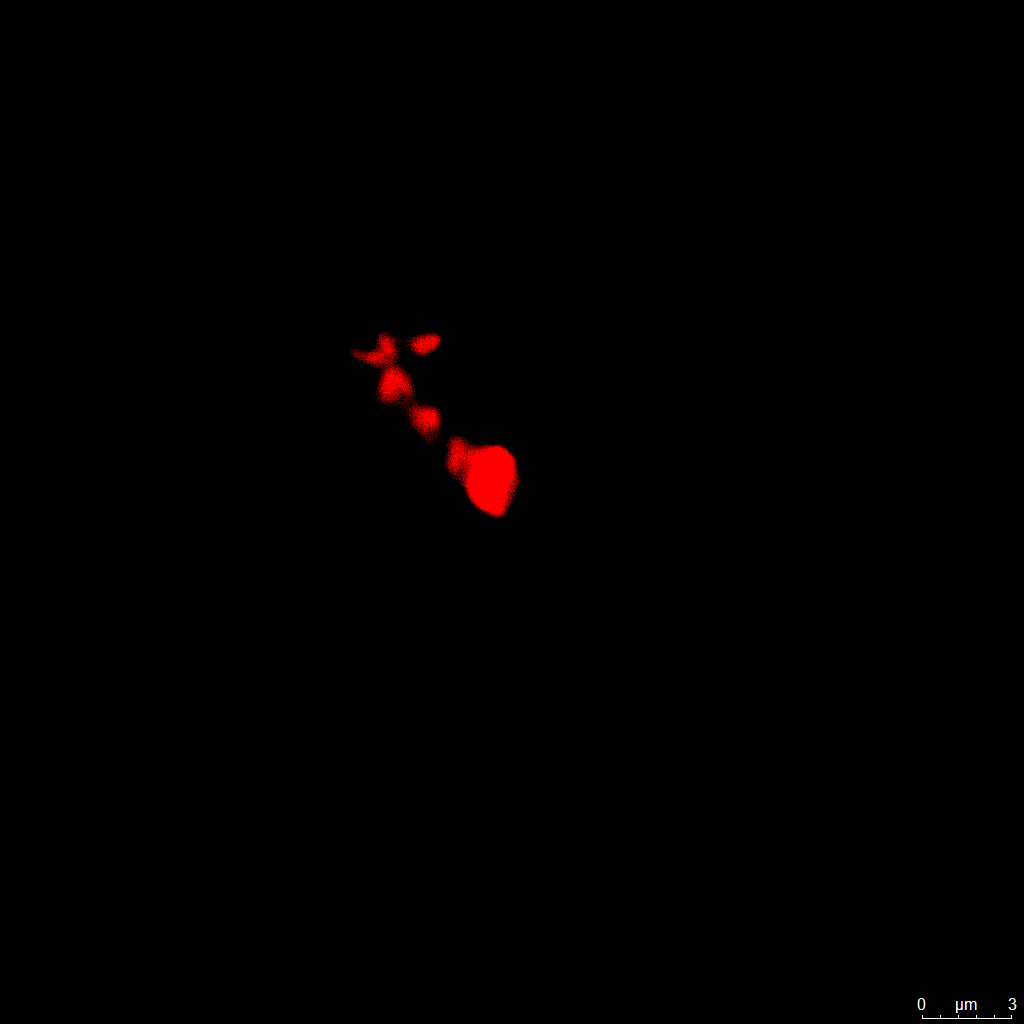

Supplement: Supplementary file 16 — Source Data for Figure 3 [file EMBJ-41-e111289-s017.zip › Microscopy_Confocal/3M/3M-B.tif]

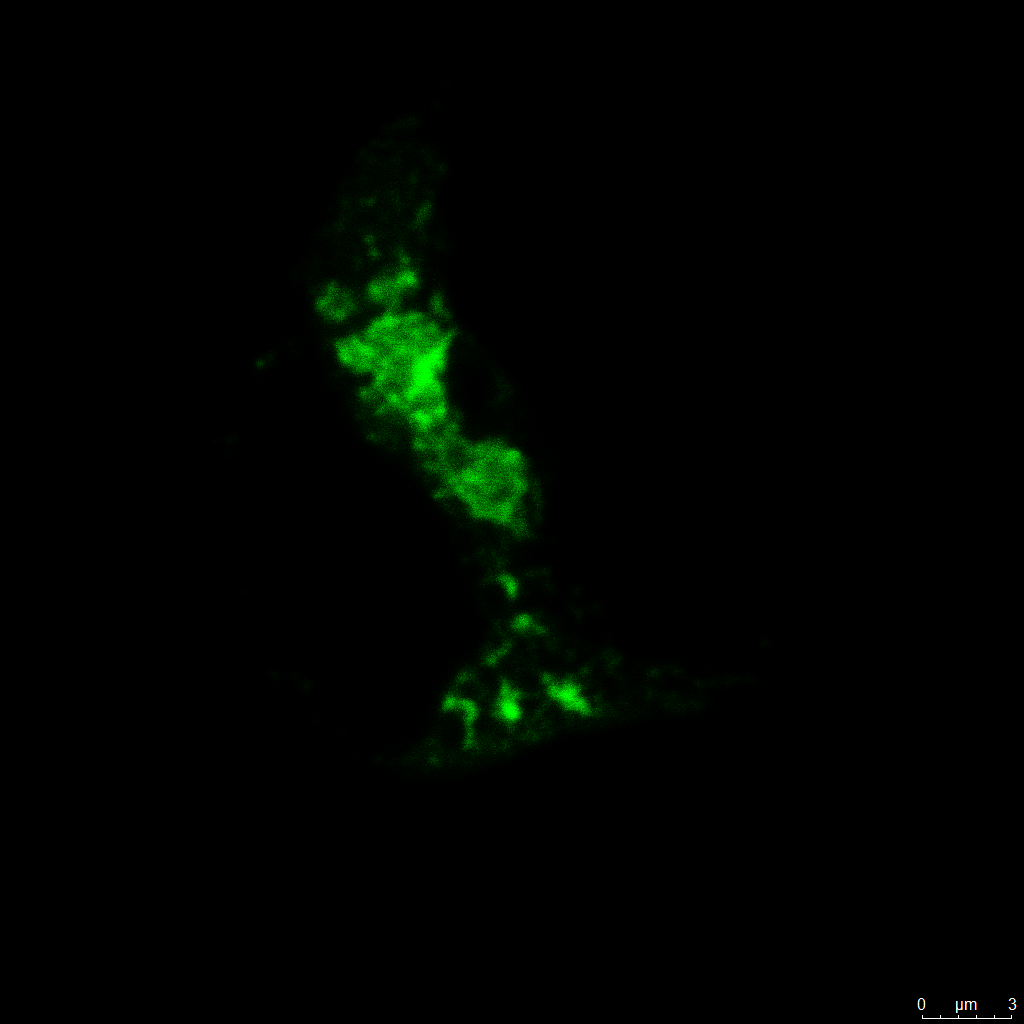

Supplement: Supplementary file 16 — Source Data for Figure 3 [file EMBJ-41-e111289-s017.zip › Microscopy_Confocal/3M/3M-C.tif]

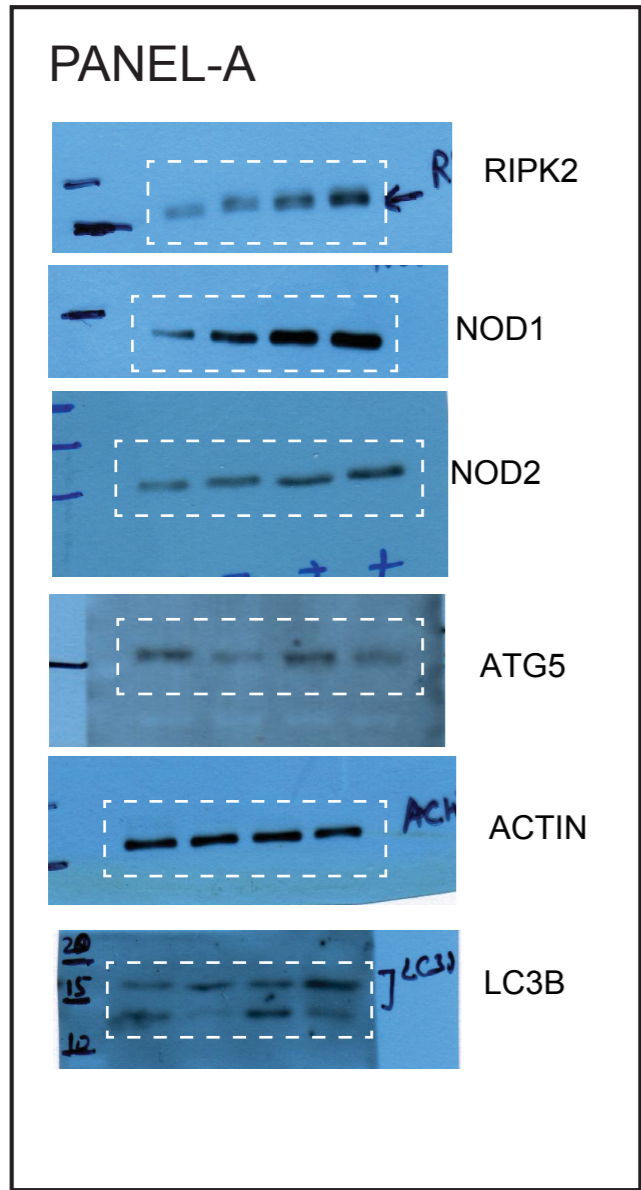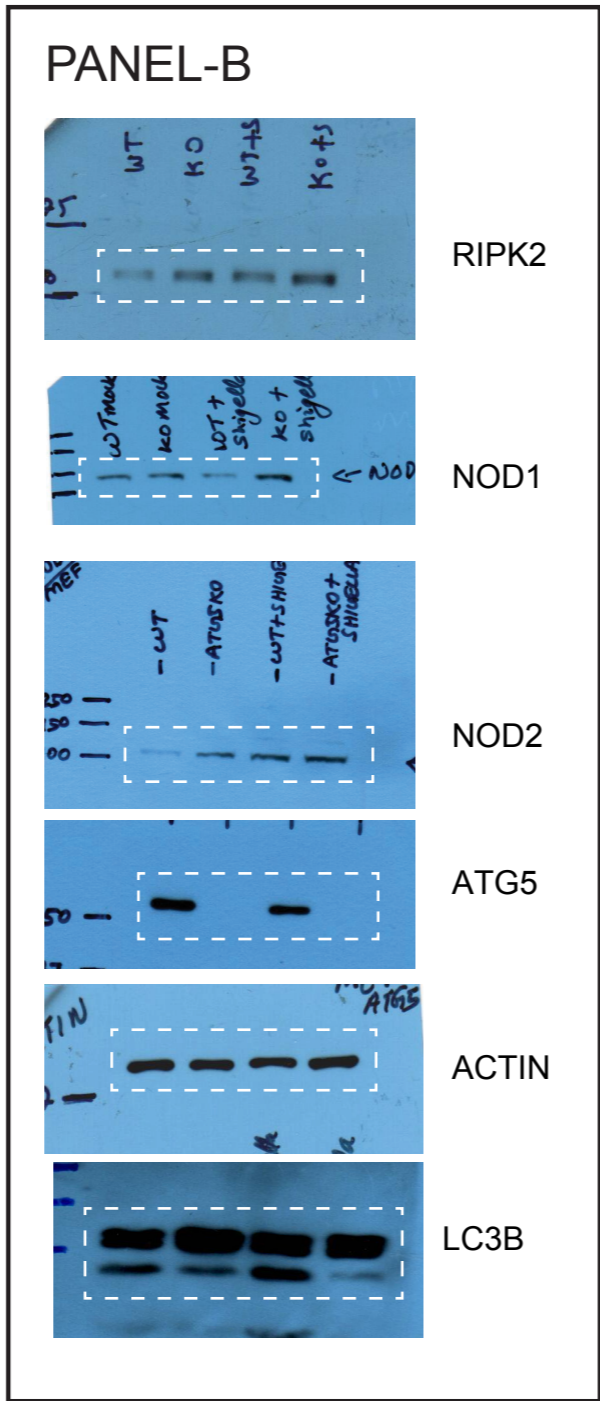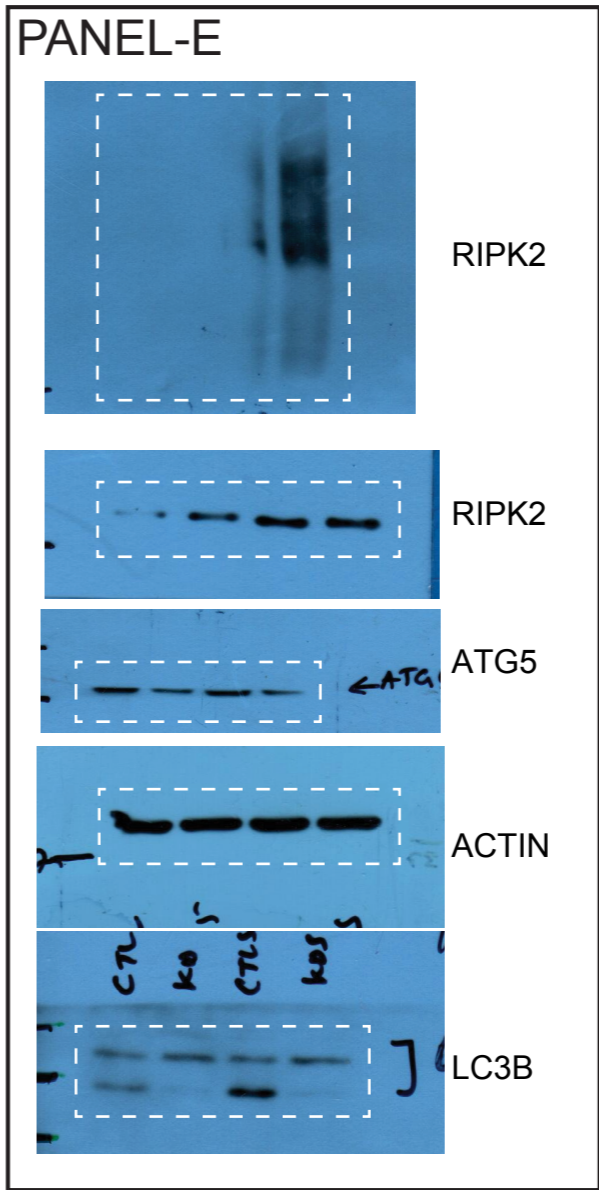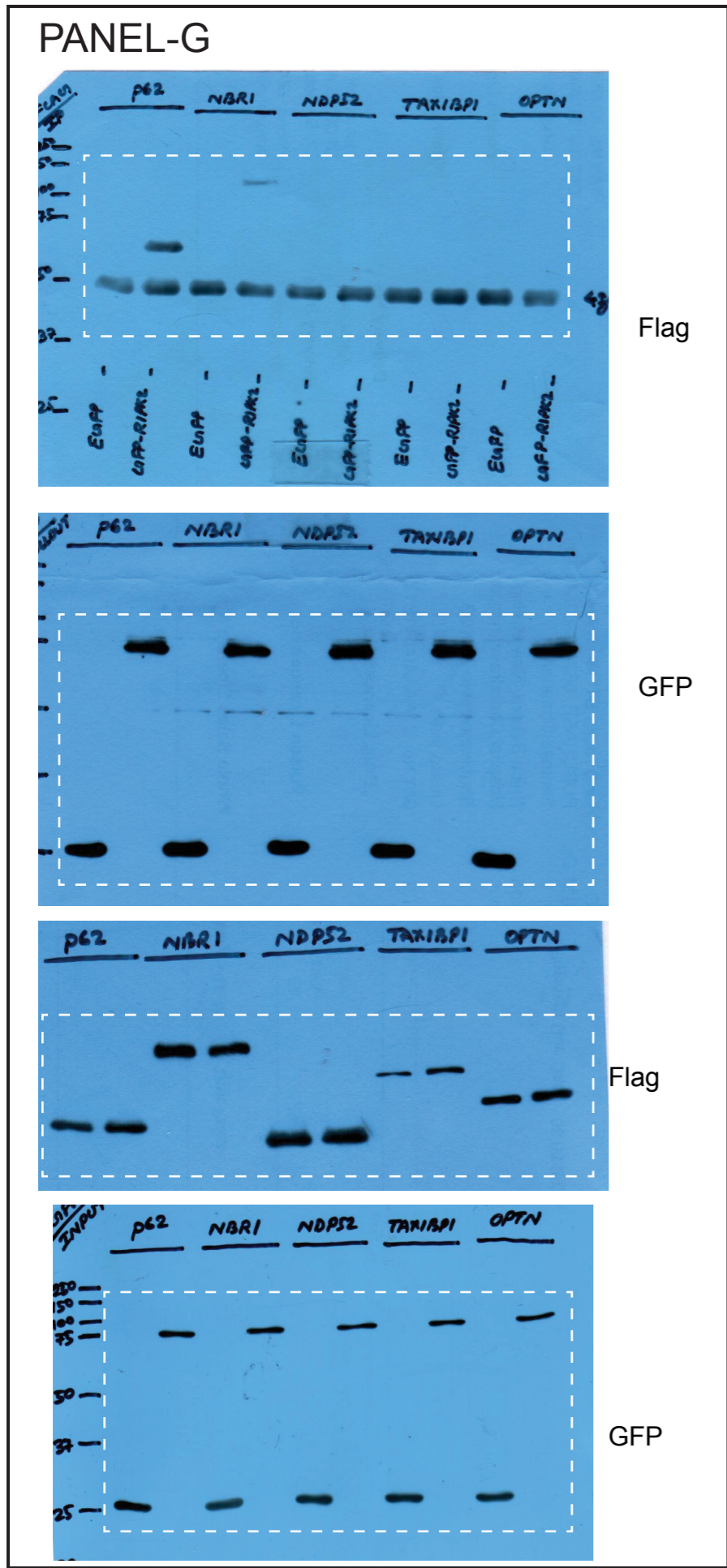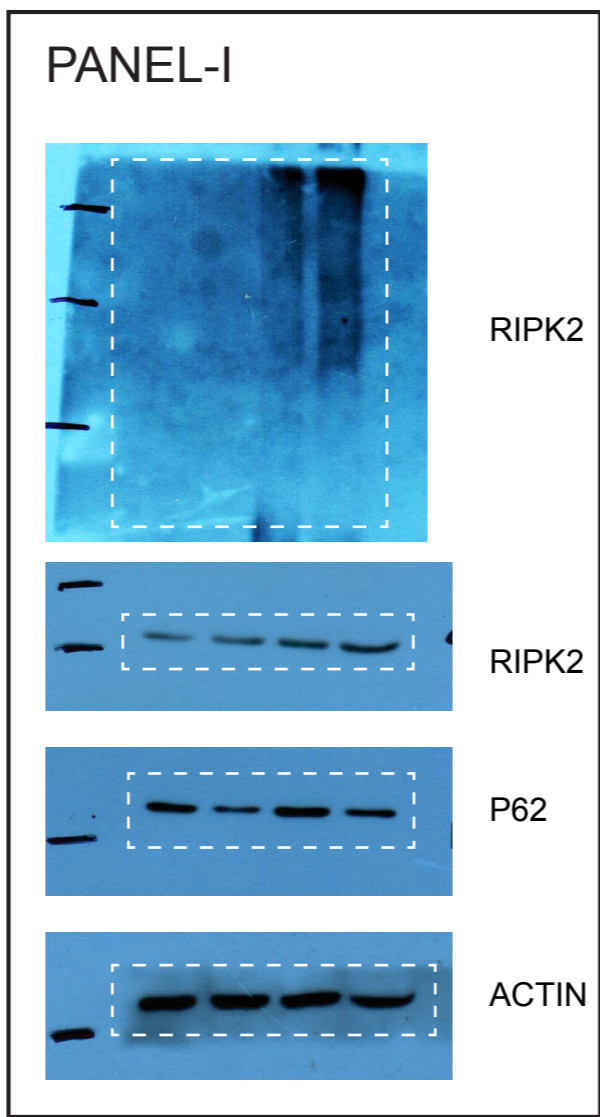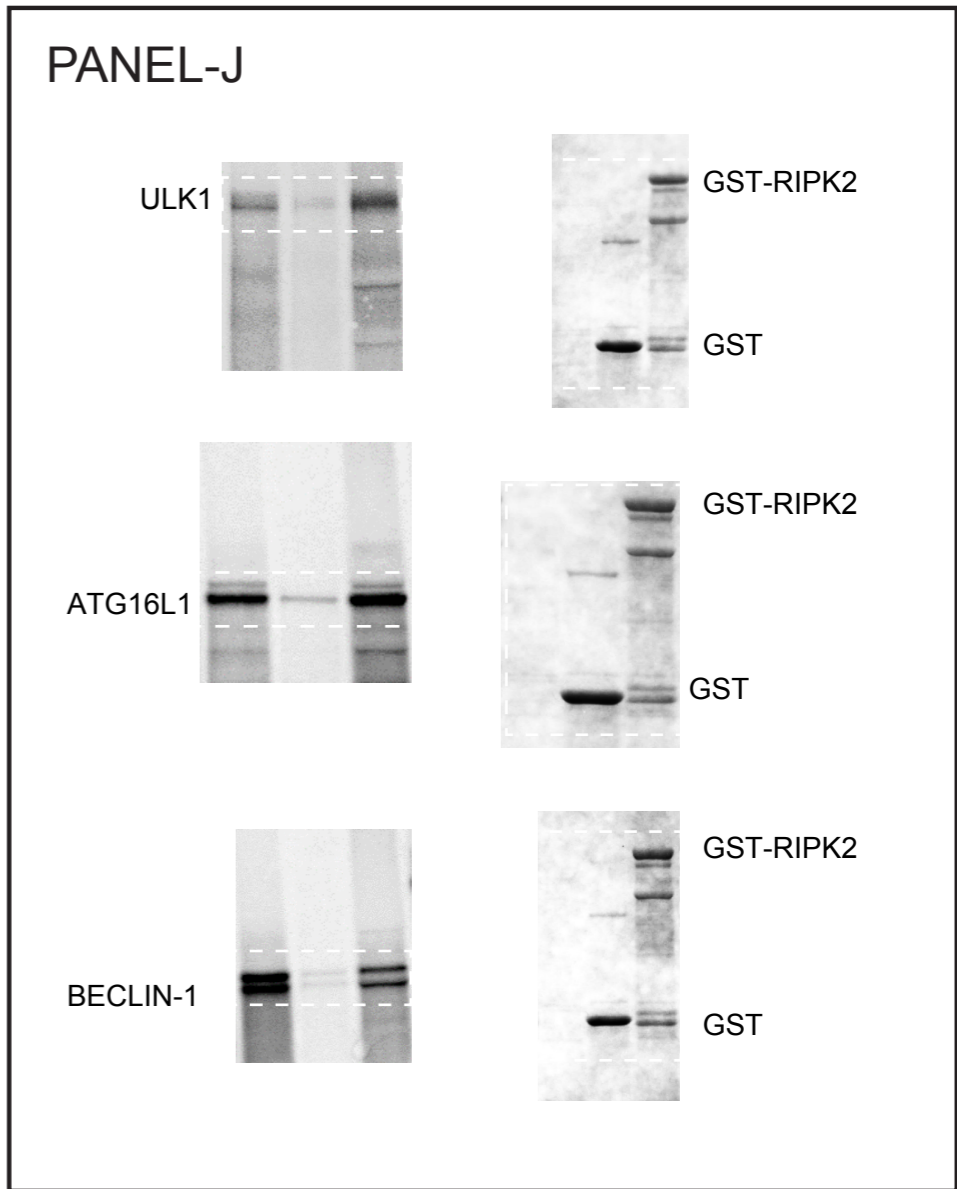

Supplement: Supplementary file 16 — Source Data for Figure 3 [file EMBJ-41-e111289-s017.zip › Western Blot/Western Blot Figure-3.pdf]

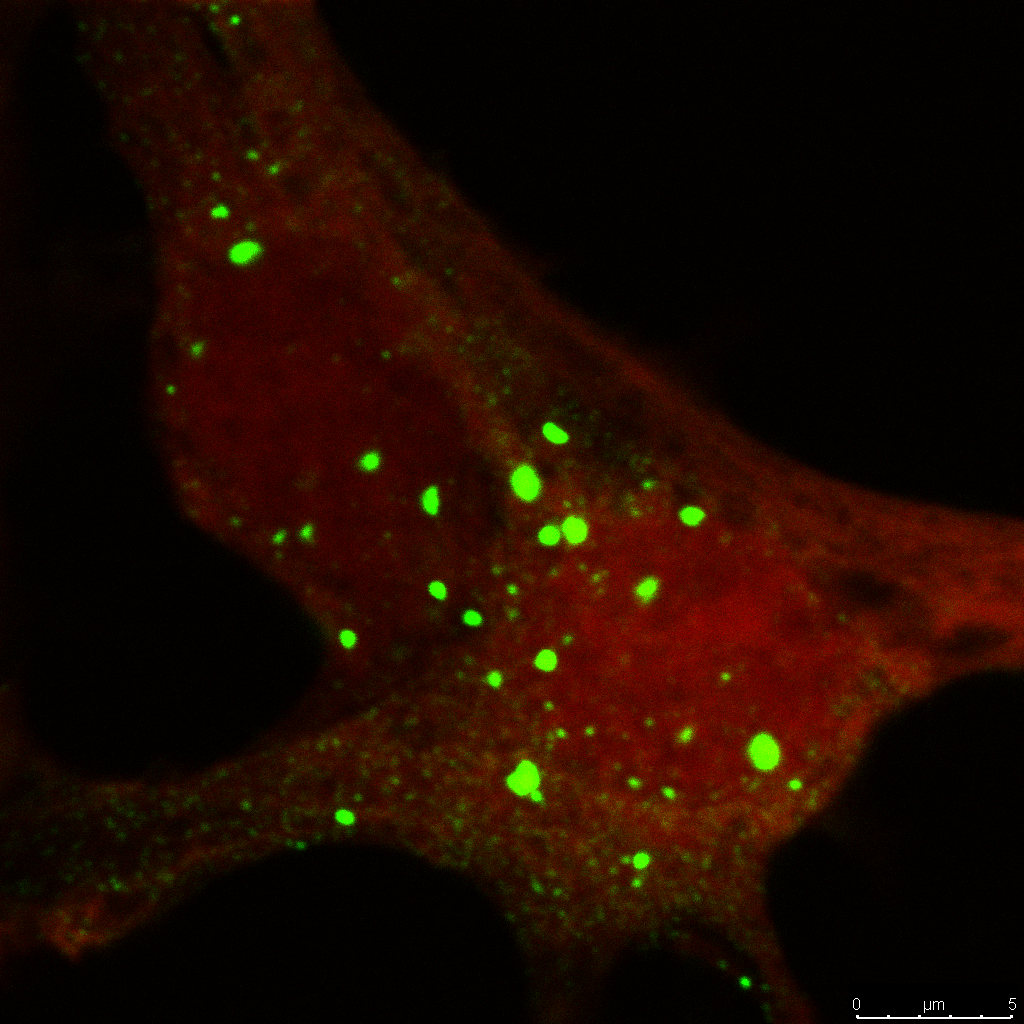

Supplement: Supplementary file 17 — Source Data for Figure 4 [file EMBJ-41-e111289-s003.zip › Microscopy_Confocal/4F/4F-A.tif]

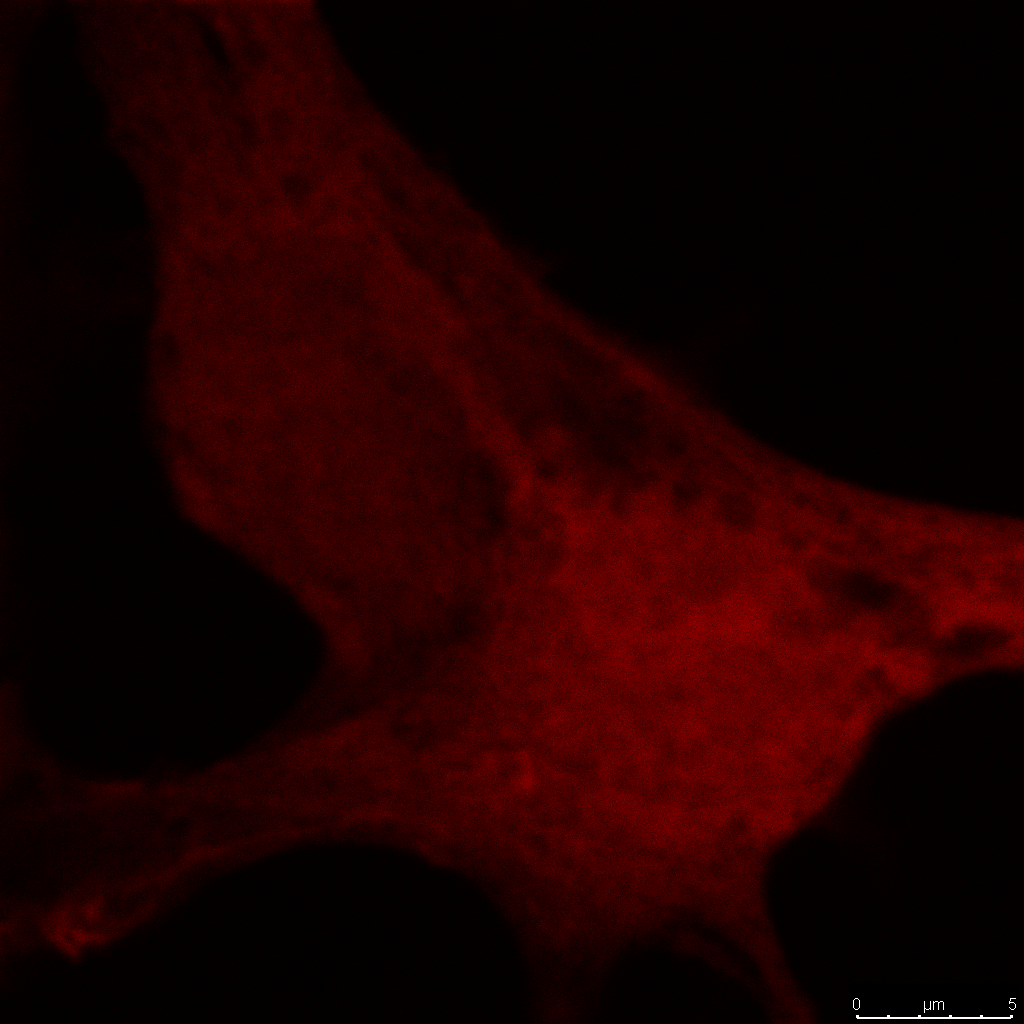

Supplement: Supplementary file 17 — Source Data for Figure 4 [file EMBJ-41-e111289-s003.zip › Microscopy_Confocal/4F/4F-B.tif]

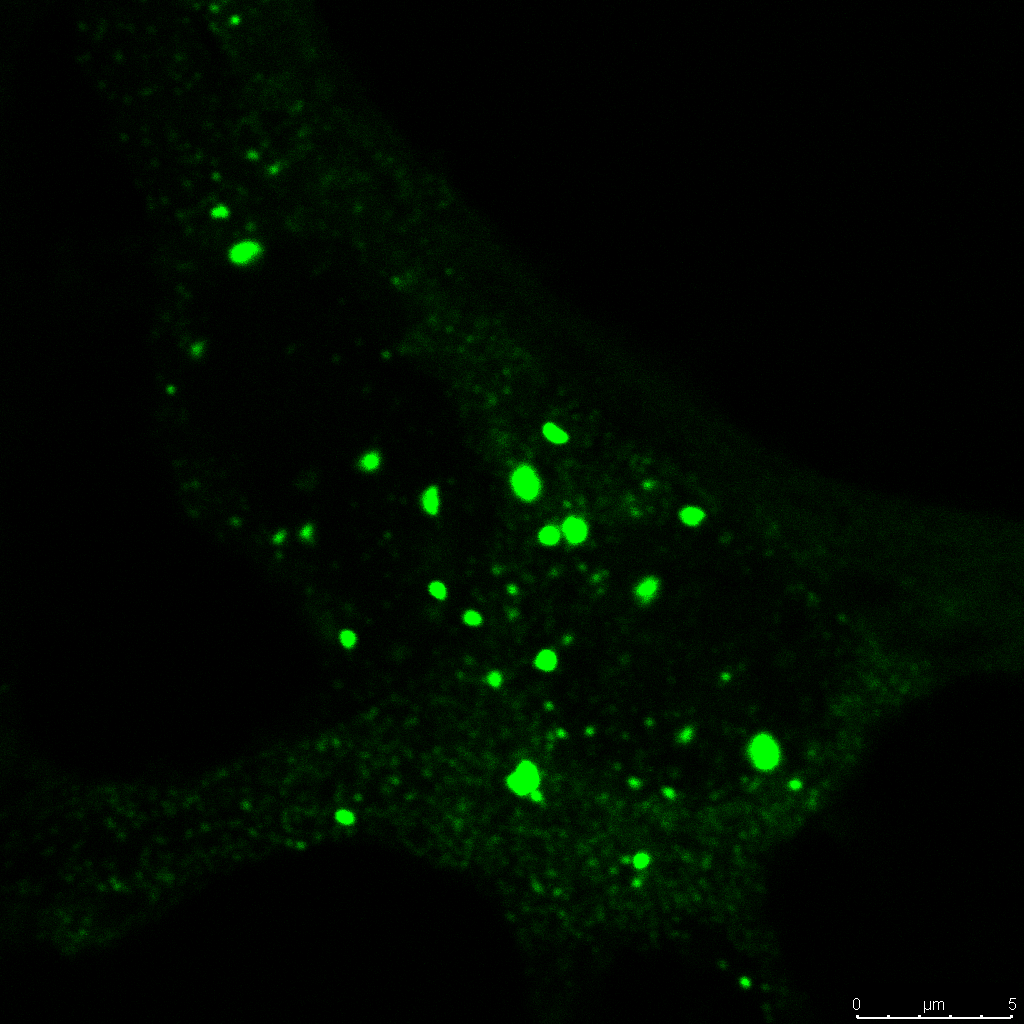

Supplement: Supplementary file 17 — Source Data for Figure 4 [file EMBJ-41-e111289-s003.zip › Microscopy_Confocal/4F/4F-C.tif]

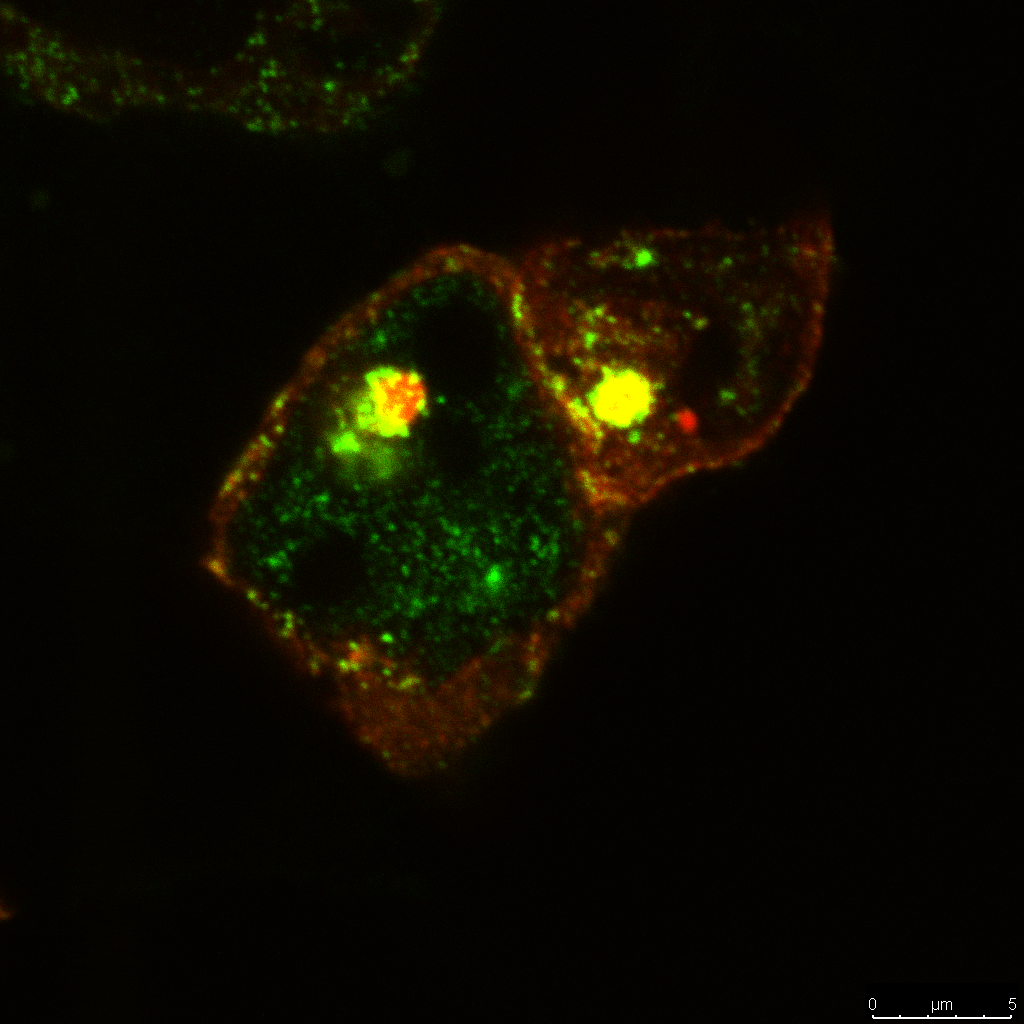

Supplement: Supplementary file 17 — Source Data for Figure 4 [file EMBJ-41-e111289-s003.zip › Microscopy_Confocal/4F/4F-D.tif]

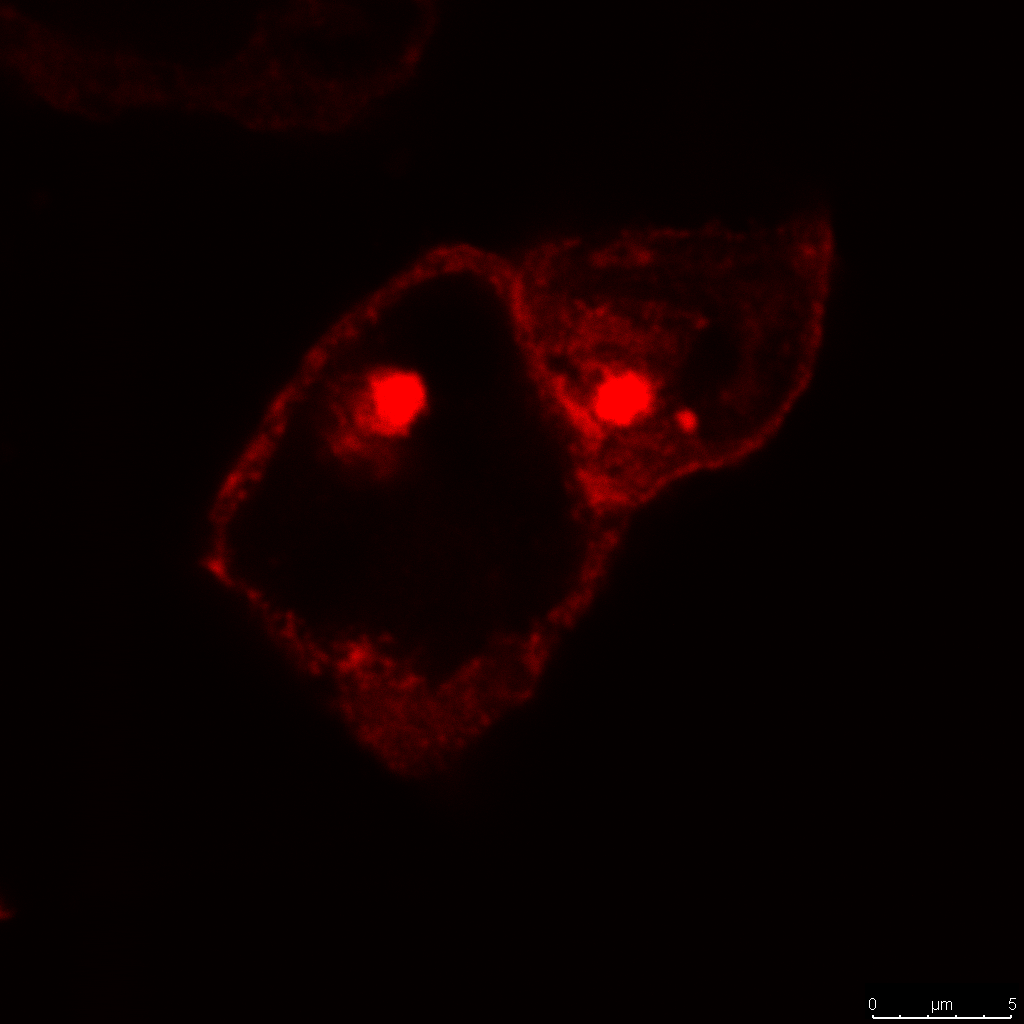

Supplement: Supplementary file 17 — Source Data for Figure 4 [file EMBJ-41-e111289-s003.zip › Microscopy_Confocal/4F/4F-E.tif]

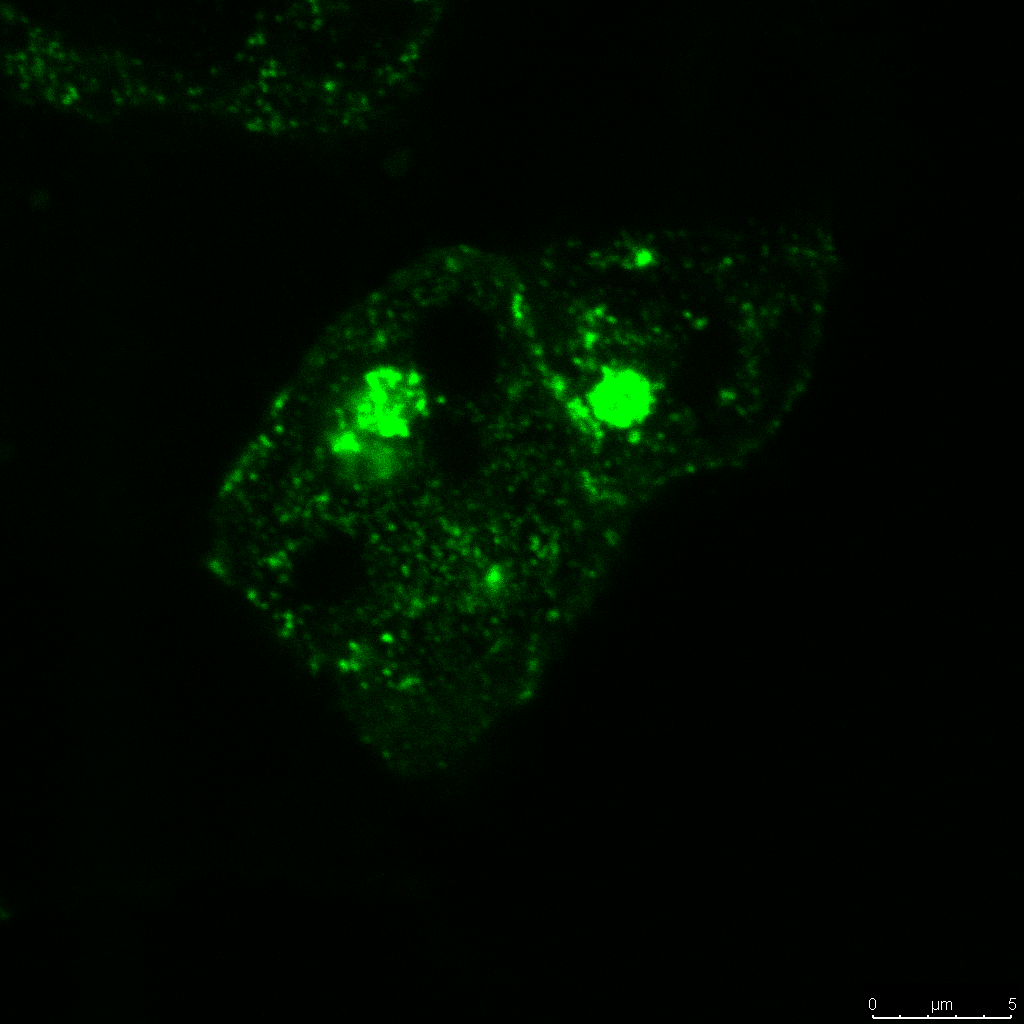

Supplement: Supplementary file 17 — Source Data for Figure 4 [file EMBJ-41-e111289-s003.zip › Microscopy_Confocal/4F/4F-F.tif]

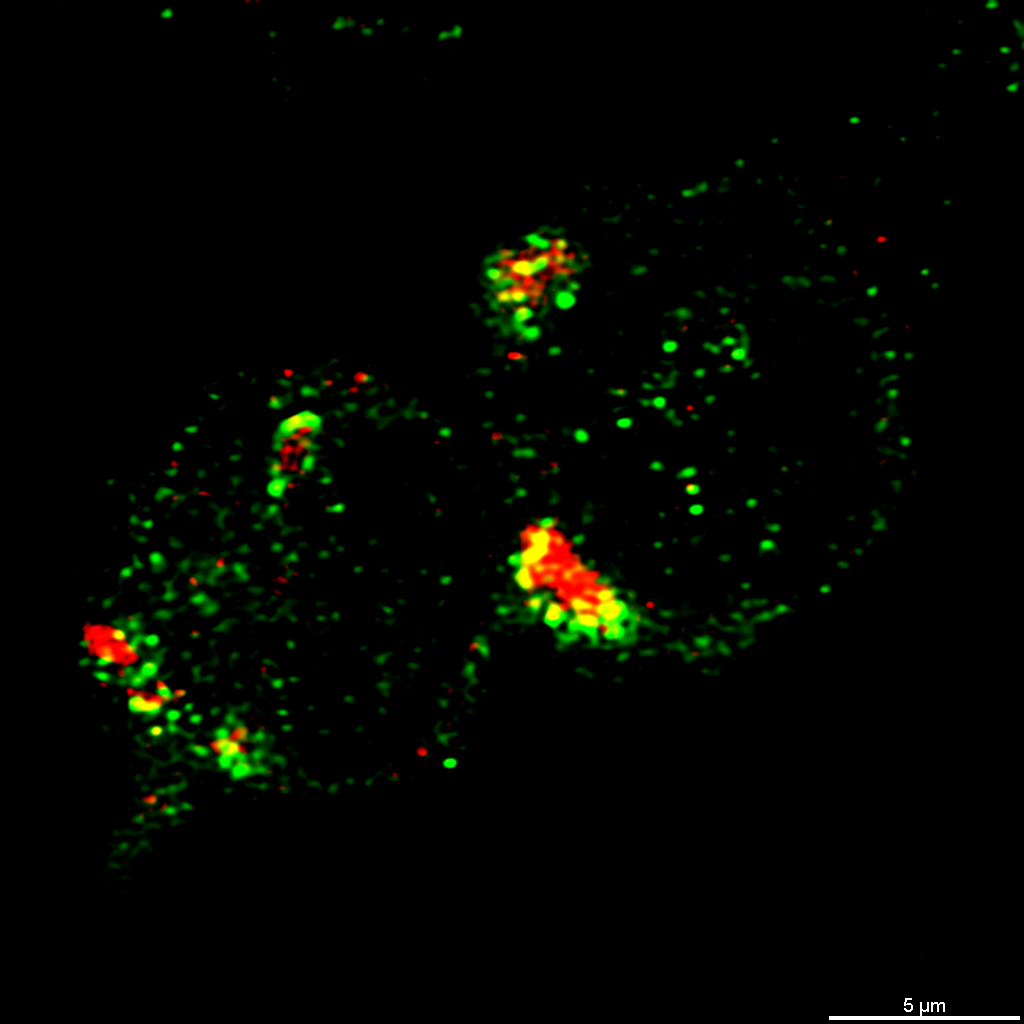

Supplement: Supplementary file 17 — Source Data for Figure 4 [file EMBJ-41-e111289-s003.zip › Microscopy_Confocal/4G/4G-A.tif]

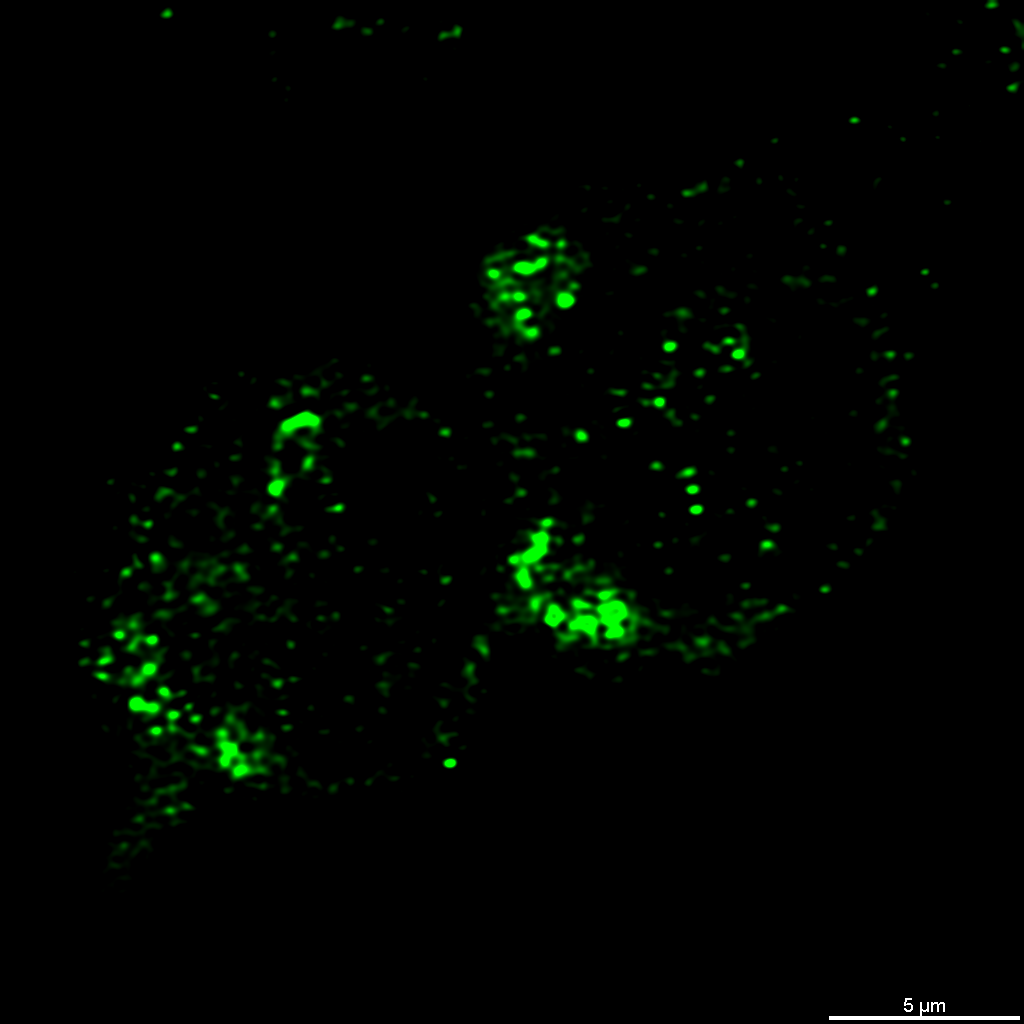

Supplement: Supplementary file 17 — Source Data for Figure 4 [file EMBJ-41-e111289-s003.zip › Microscopy_Confocal/4G/4G-B.tif]

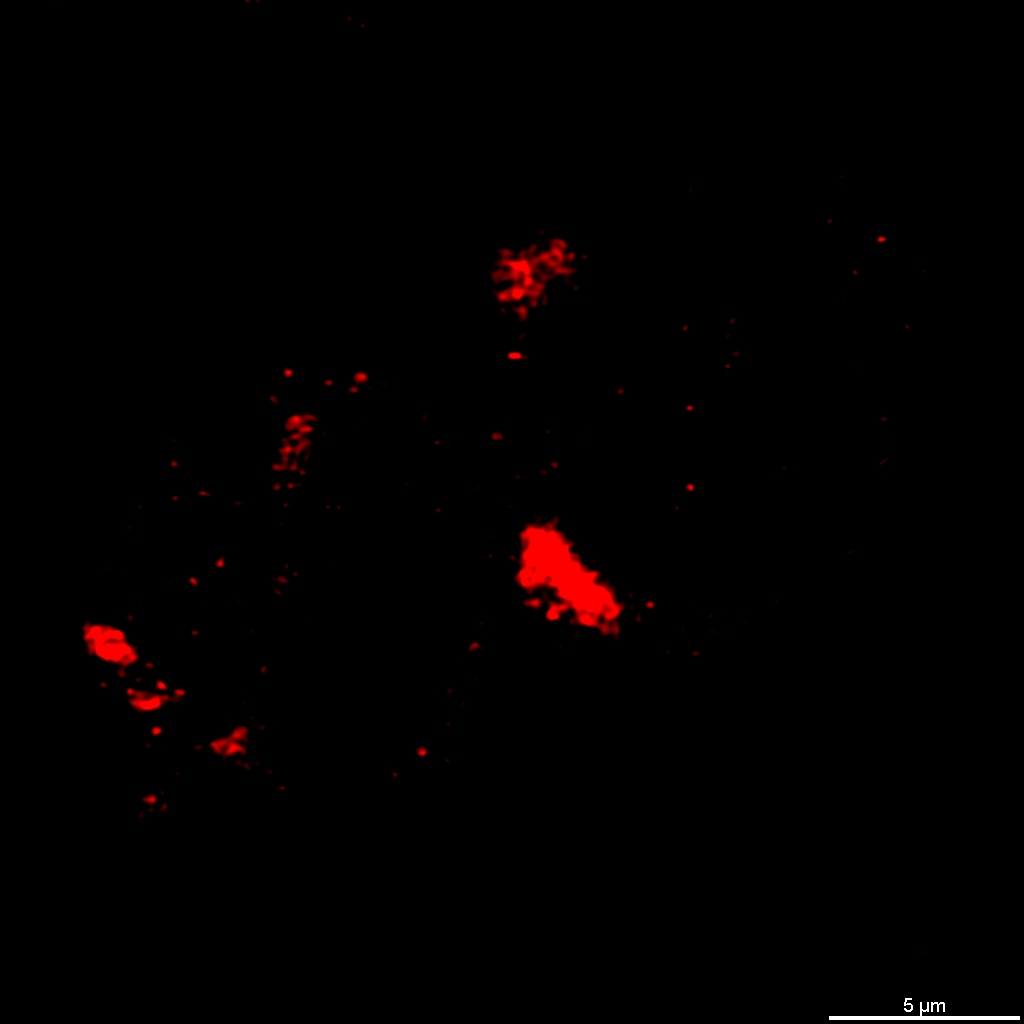

Supplement: Supplementary file 17 — Source Data for Figure 4 [file EMBJ-41-e111289-s003.zip › Microscopy_Confocal/4G/4G-C.tif]

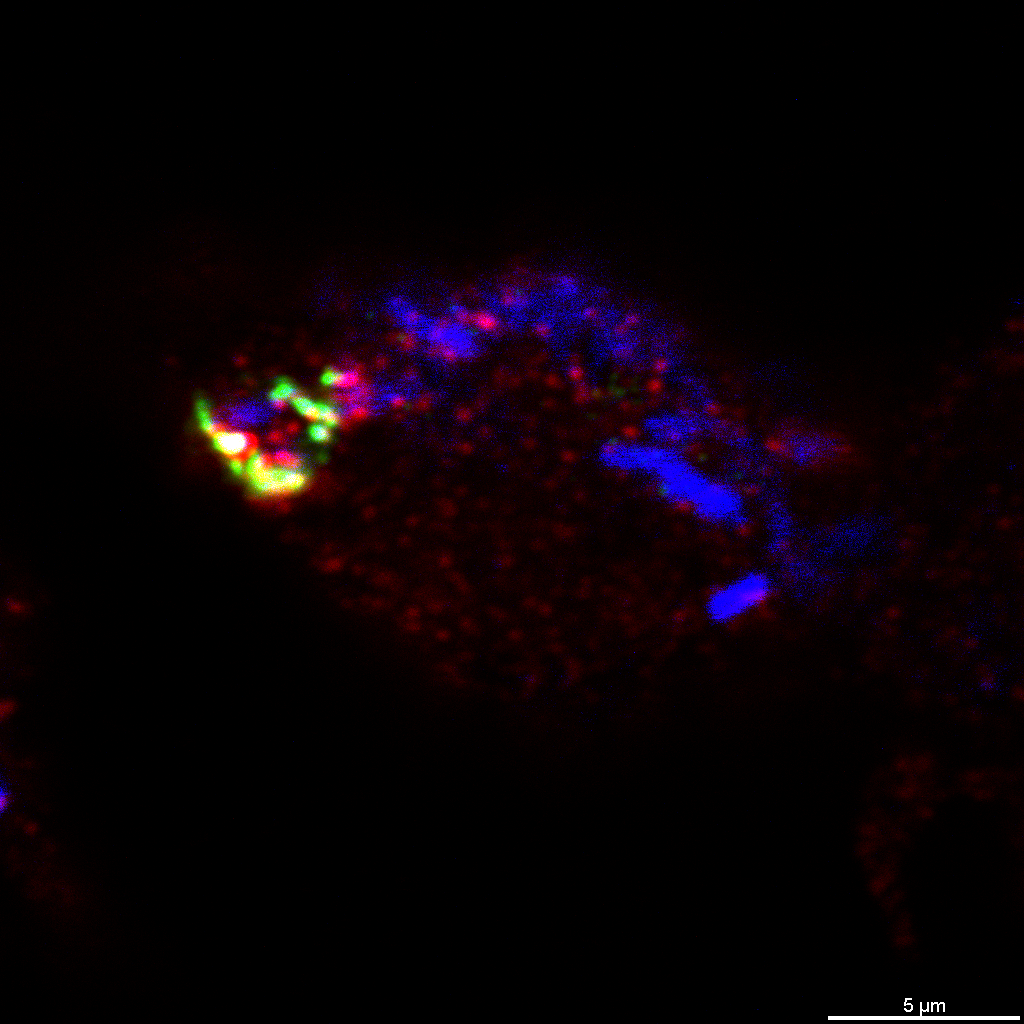

Supplement: Supplementary file 17 — Source Data for Figure 4 [file EMBJ-41-e111289-s003.zip › Microscopy_Confocal/4H/4H-A.tif]

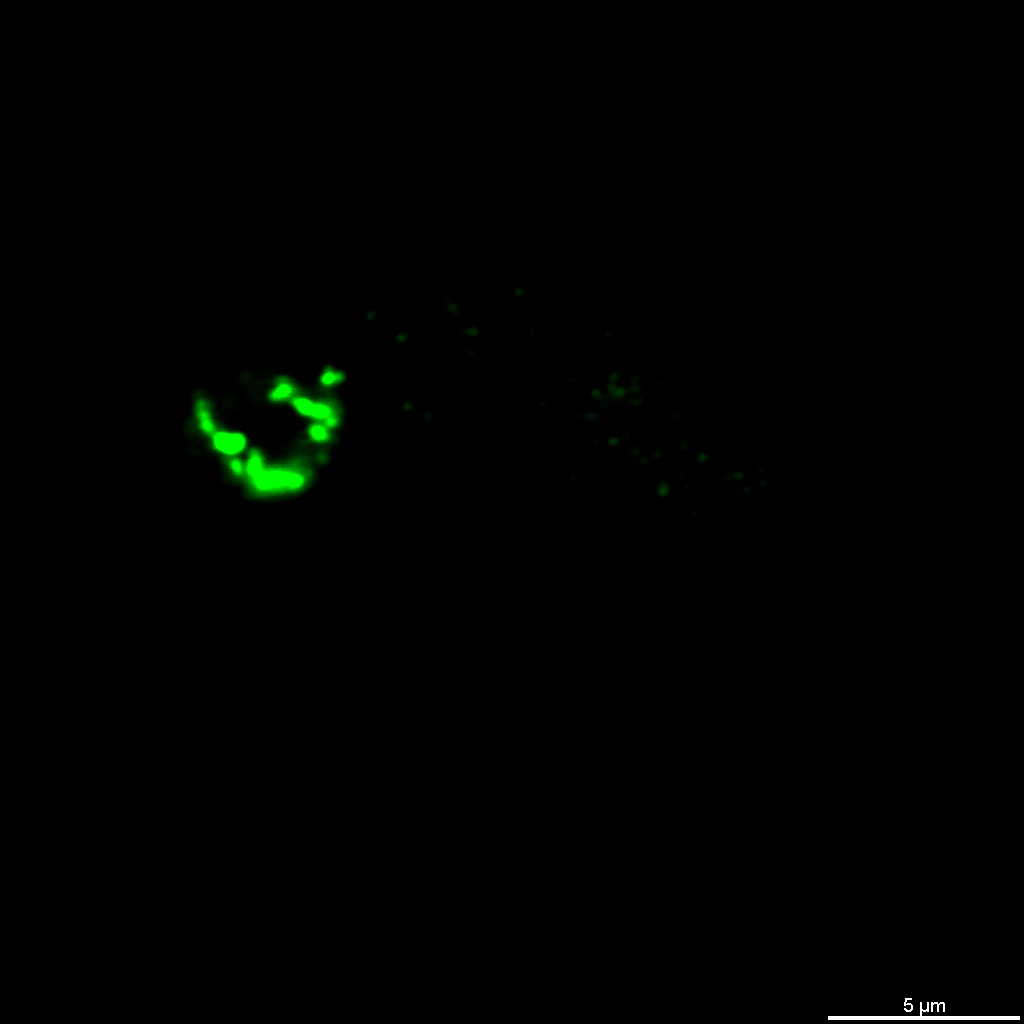

Supplement: Supplementary file 17 — Source Data for Figure 4 [file EMBJ-41-e111289-s003.zip › Microscopy_Confocal/4H/4H-B.tif]

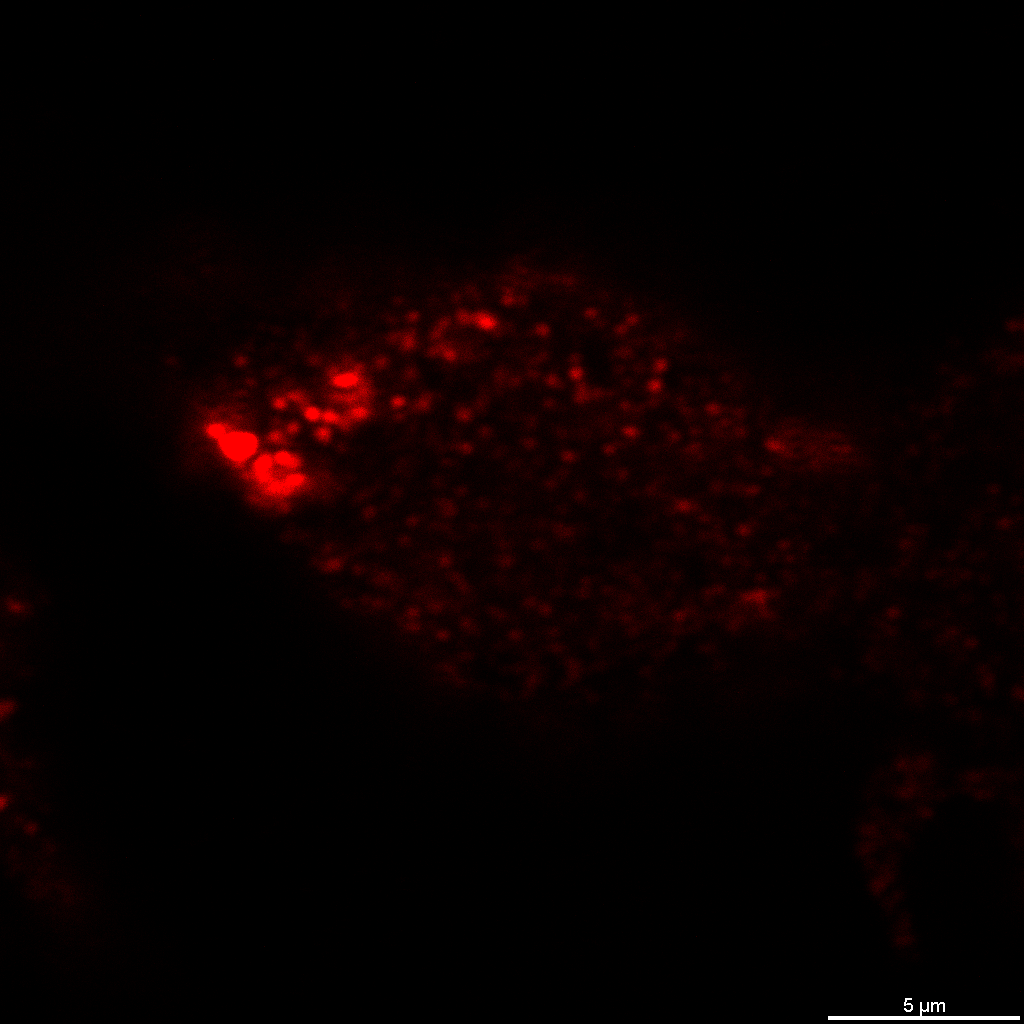

Supplement: Supplementary file 17 — Source Data for Figure 4 [file EMBJ-41-e111289-s003.zip › Microscopy_Confocal/4H/4H-C.tif]

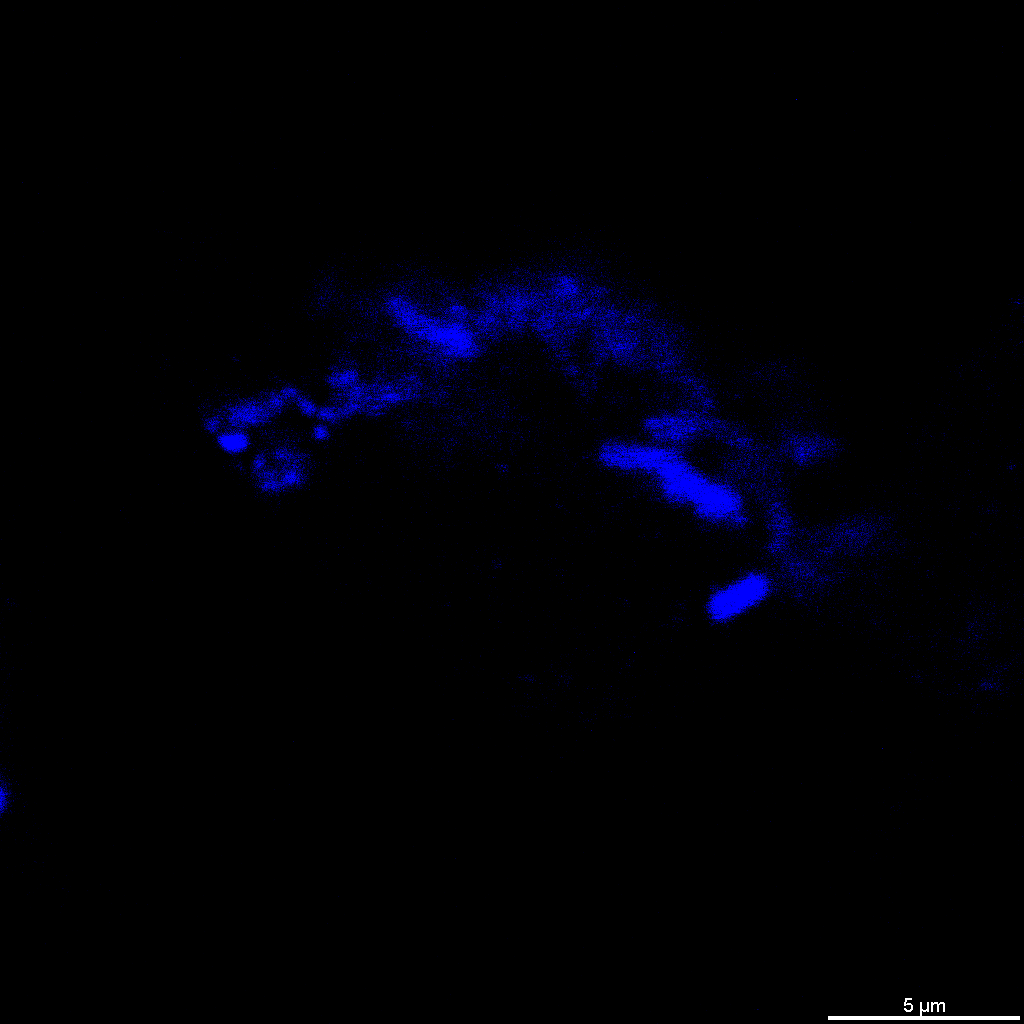

Supplement: Supplementary file 17 — Source Data for Figure 4 [file EMBJ-41-e111289-s003.zip › Microscopy_Confocal/4H/4H-D.tif]

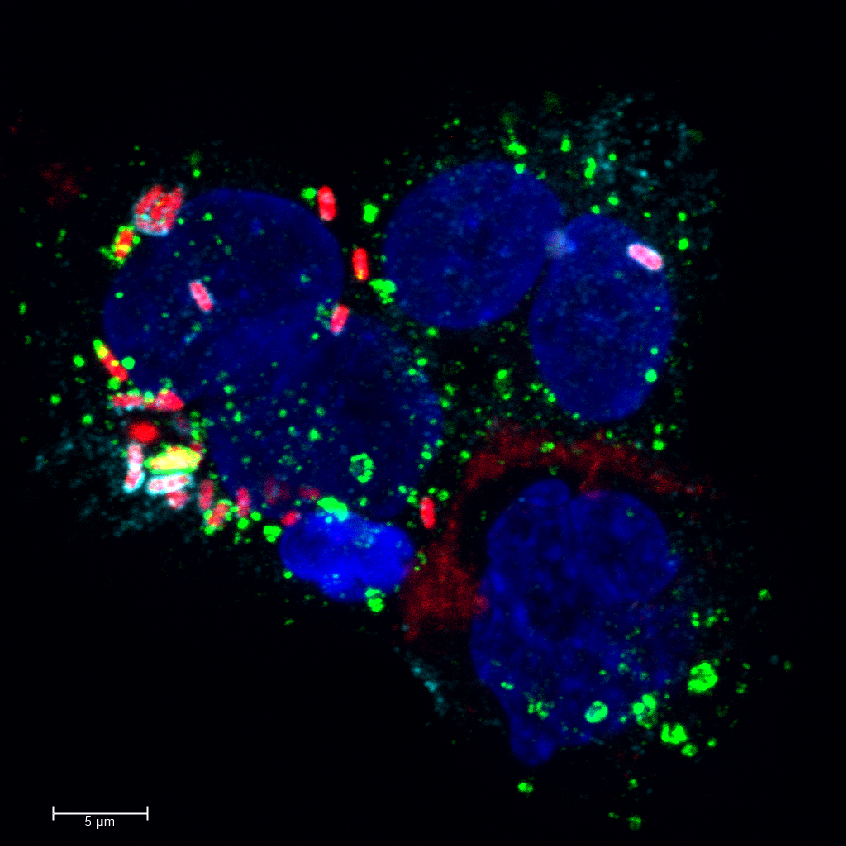

Supplement: Supplementary file 17 — Source Data for Figure 4 [file EMBJ-41-e111289-s003.zip › Microscopy_Confocal/4I/4I-A.tif]

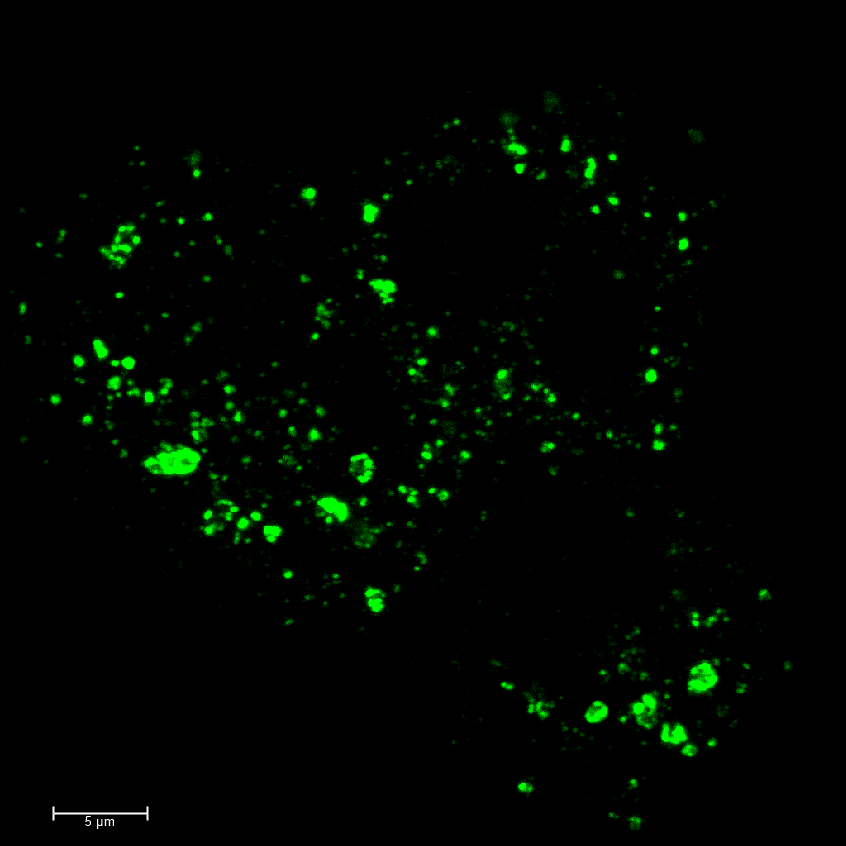

Supplement: Supplementary file 17 — Source Data for Figure 4 [file EMBJ-41-e111289-s003.zip › Microscopy_Confocal/4I/4I-B.tif]

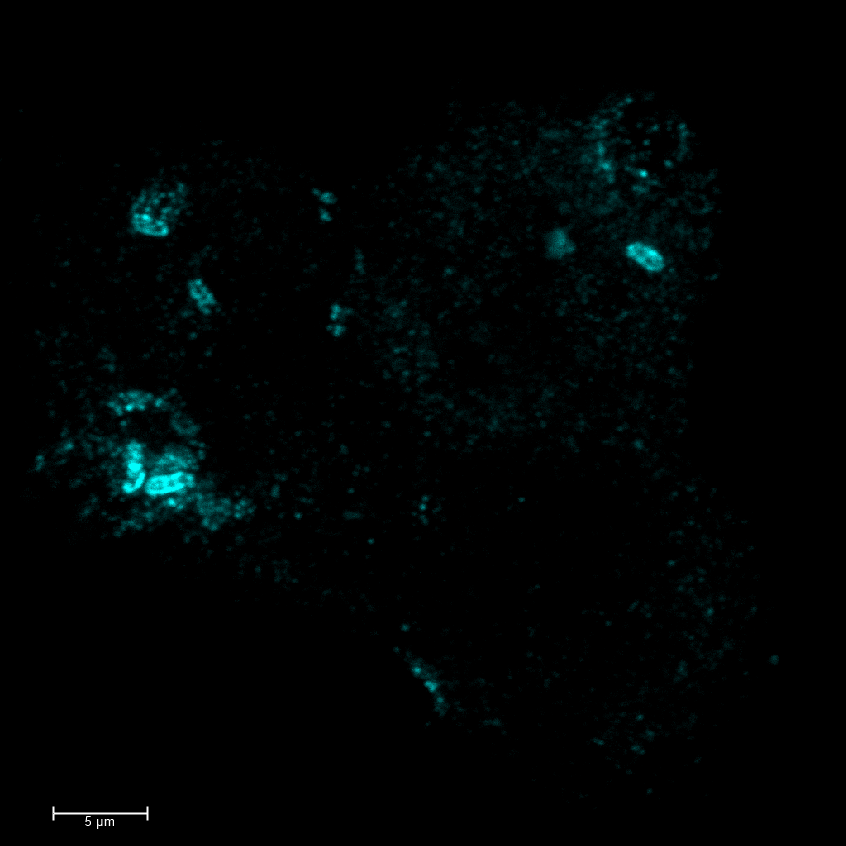

Supplement: Supplementary file 17 — Source Data for Figure 4 [file EMBJ-41-e111289-s003.zip › Microscopy_Confocal/4I/4I-C.tif]

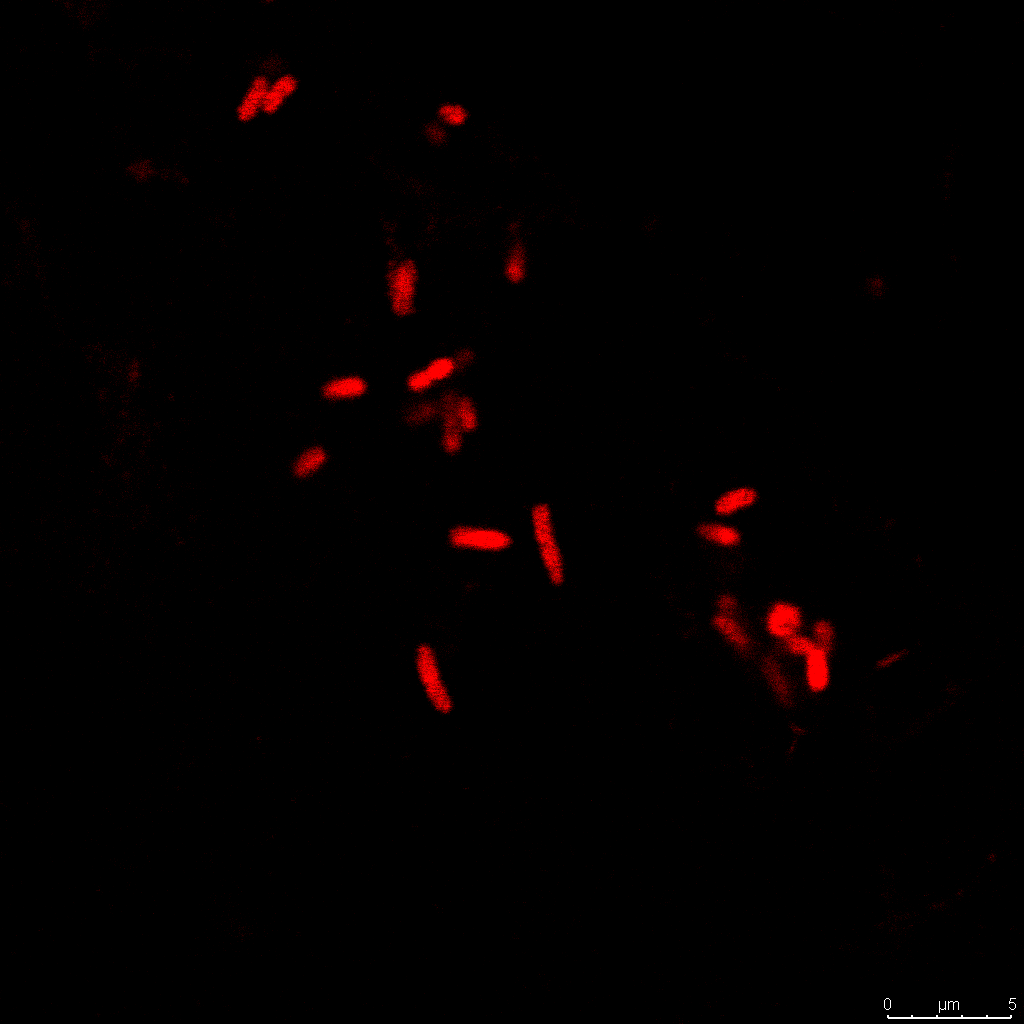

Supplement: Supplementary file 17 — Source Data for Figure 4 [file EMBJ-41-e111289-s003.zip › Microscopy_Confocal/4I/4I-D.tif]

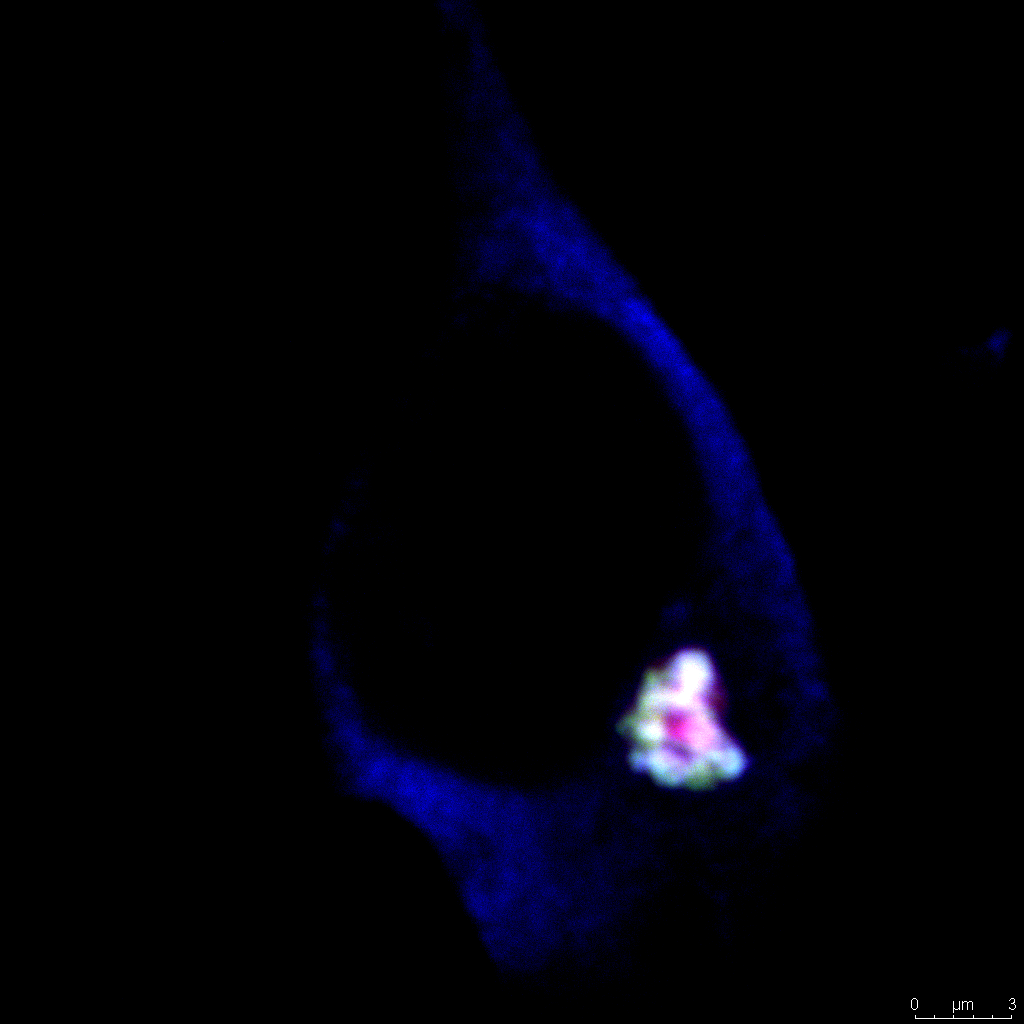

Supplement: Supplementary file 17 — Source Data for Figure 4 [file EMBJ-41-e111289-s003.zip › Microscopy_Confocal/4J/4J-A.tif]

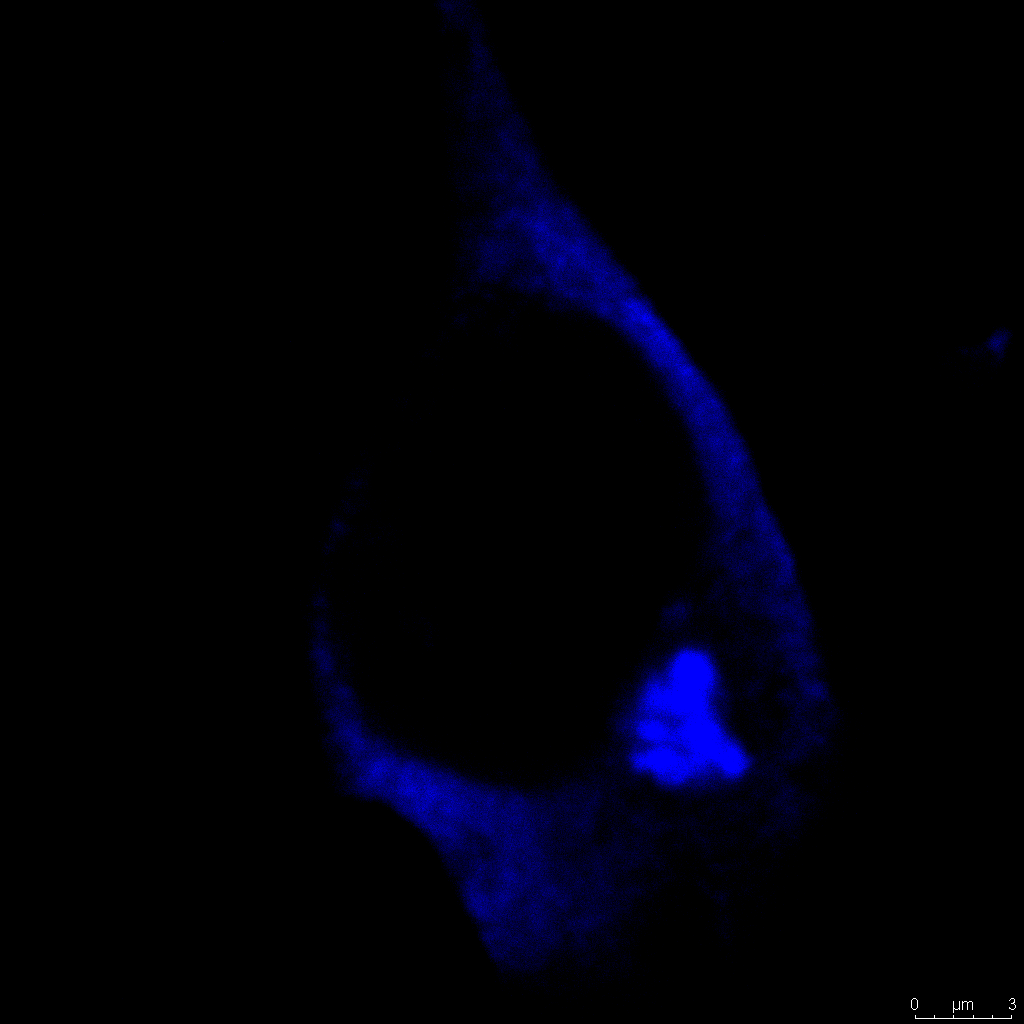

Supplement: Supplementary file 17 — Source Data for Figure 4 [file EMBJ-41-e111289-s003.zip › Microscopy_Confocal/4J/4J-B.tif]

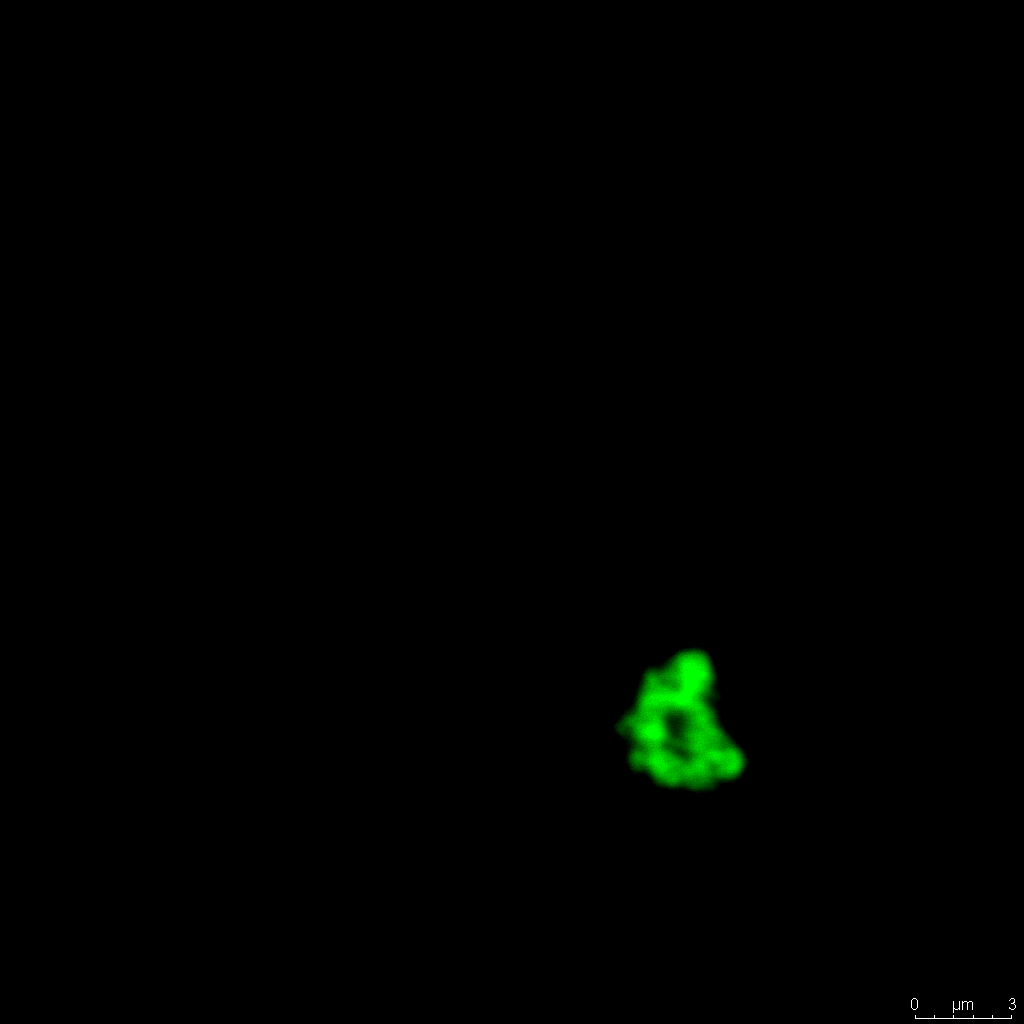

Supplement: Supplementary file 17 — Source Data for Figure 4 [file EMBJ-41-e111289-s003.zip › Microscopy_Confocal/4J/4J-C.tif]

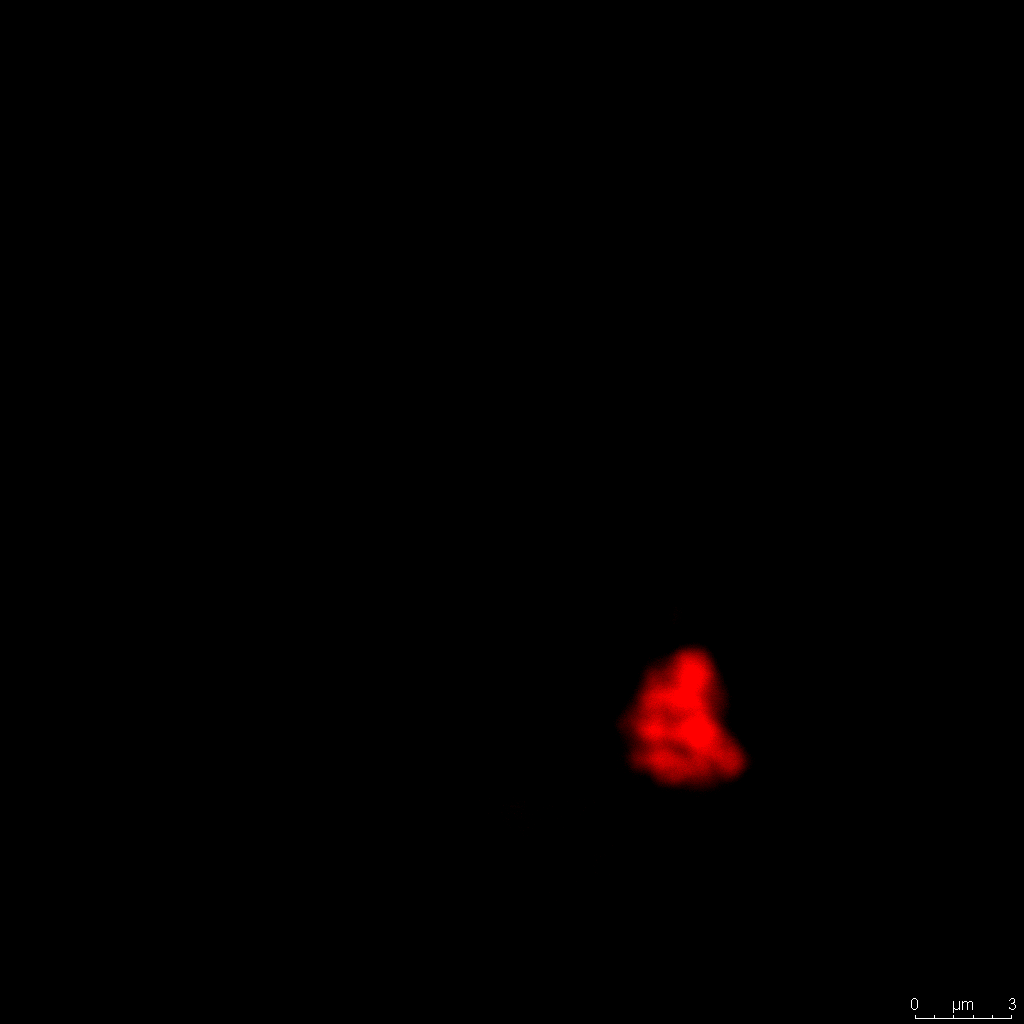

Supplement: Supplementary file 17 — Source Data for Figure 4 [file EMBJ-41-e111289-s003.zip › Microscopy_Confocal/4J/4J-D.tif]

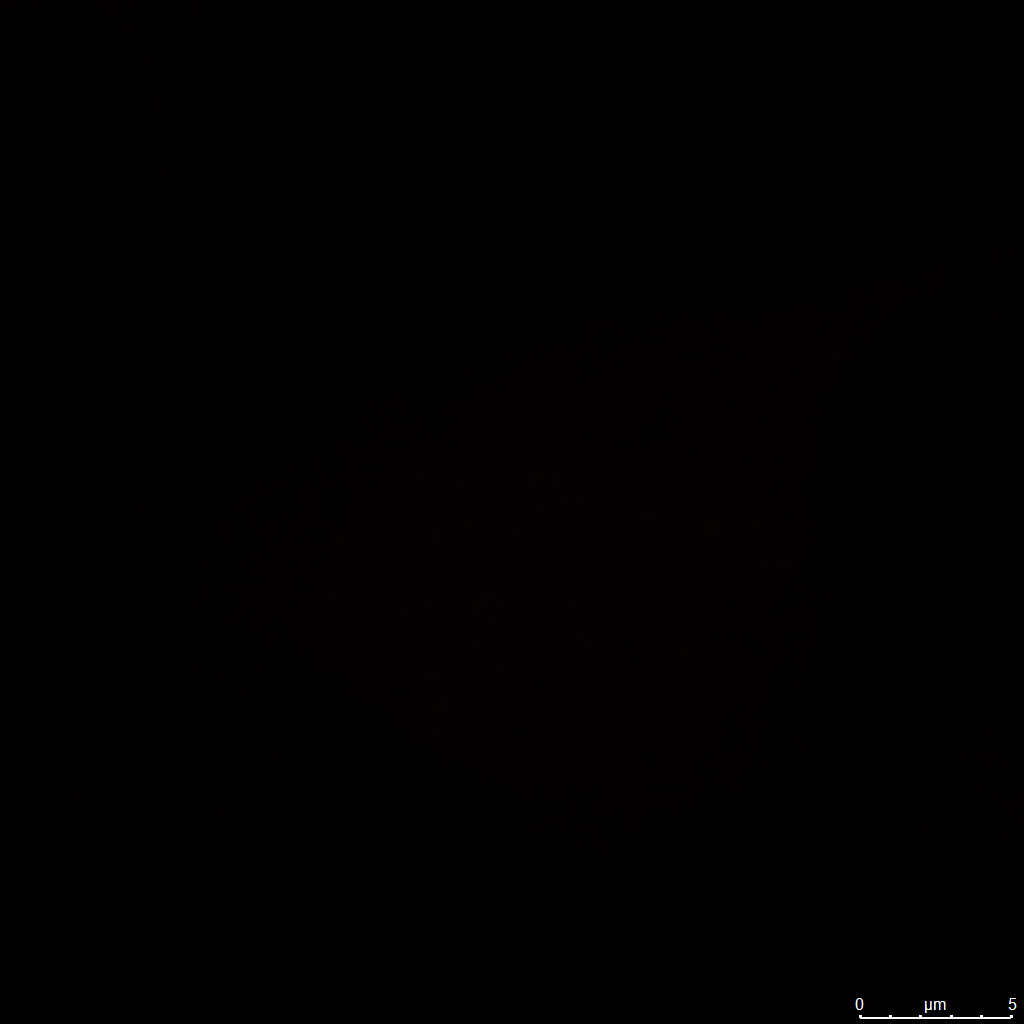

Supplement: Supplementary file 17 — Source Data for Figure 4 [file EMBJ-41-e111289-s003.zip › PLA/4K/4K-1.tif]

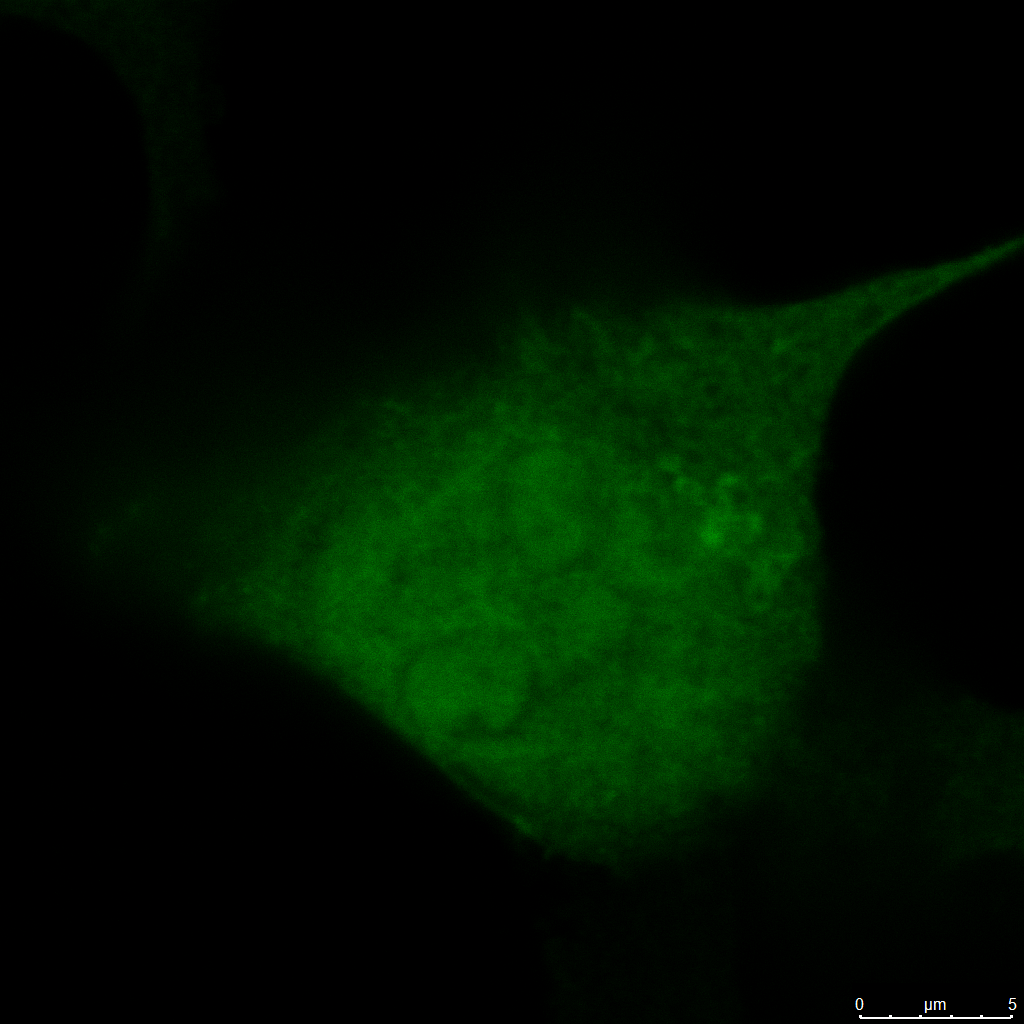

Supplement: Supplementary file 17 — Source Data for Figure 4 [file EMBJ-41-e111289-s003.zip › PLA/4K/4K-2.tif]

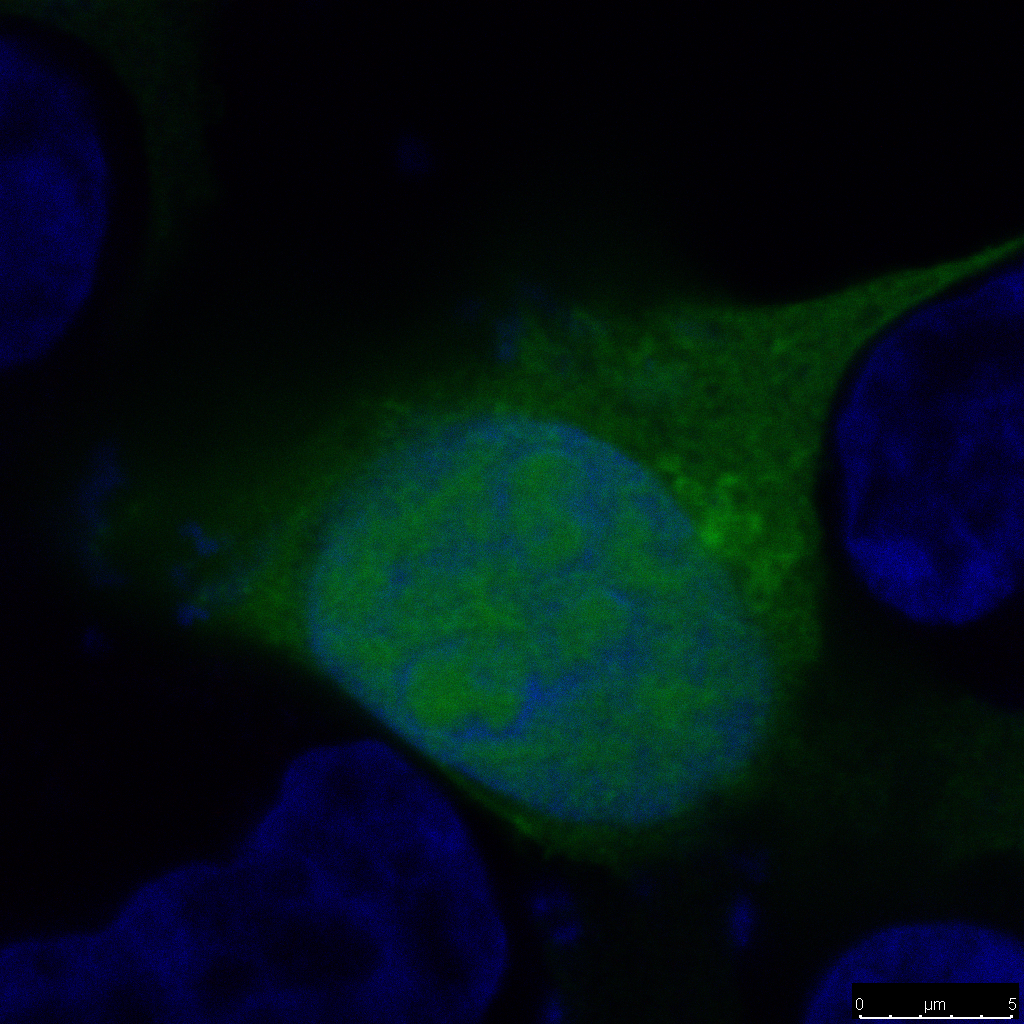

Supplement: Supplementary file 17 — Source Data for Figure 4 [file EMBJ-41-e111289-s003.zip › PLA/4K/4K-3.tif]

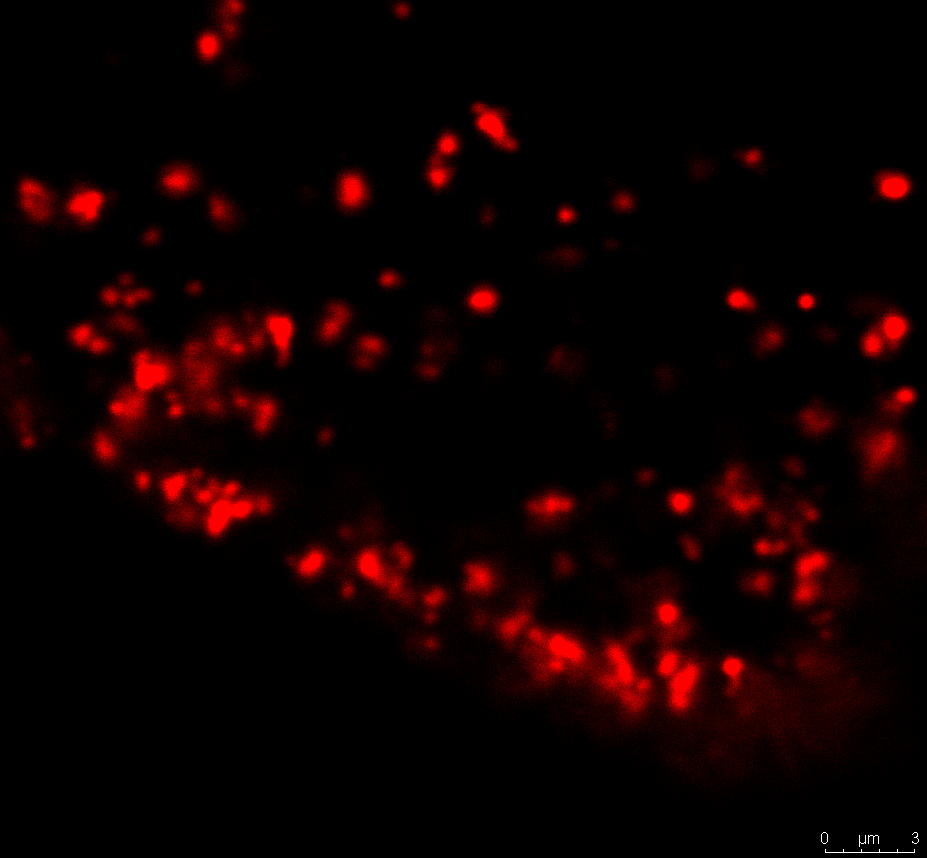

Supplement: Supplementary file 17 — Source Data for Figure 4 [file EMBJ-41-e111289-s003.zip › PLA/4K/4K-4.tif]

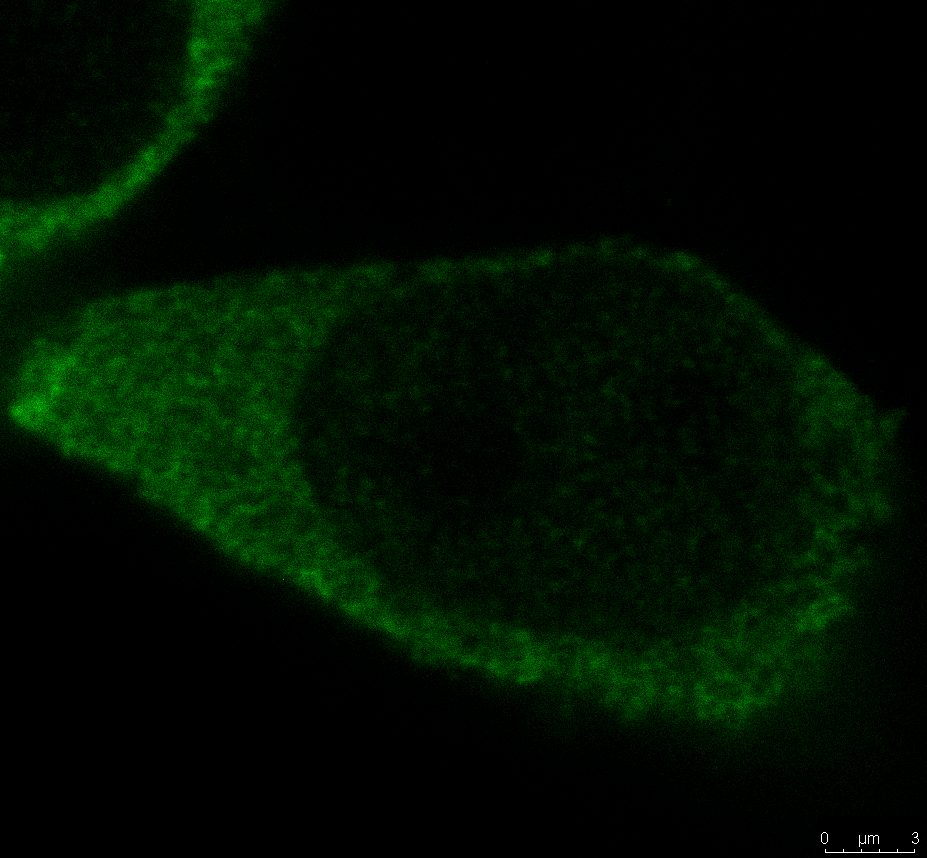

Supplement: Supplementary file 17 — Source Data for Figure 4 [file EMBJ-41-e111289-s003.zip › PLA/4K/4K-5.tif]

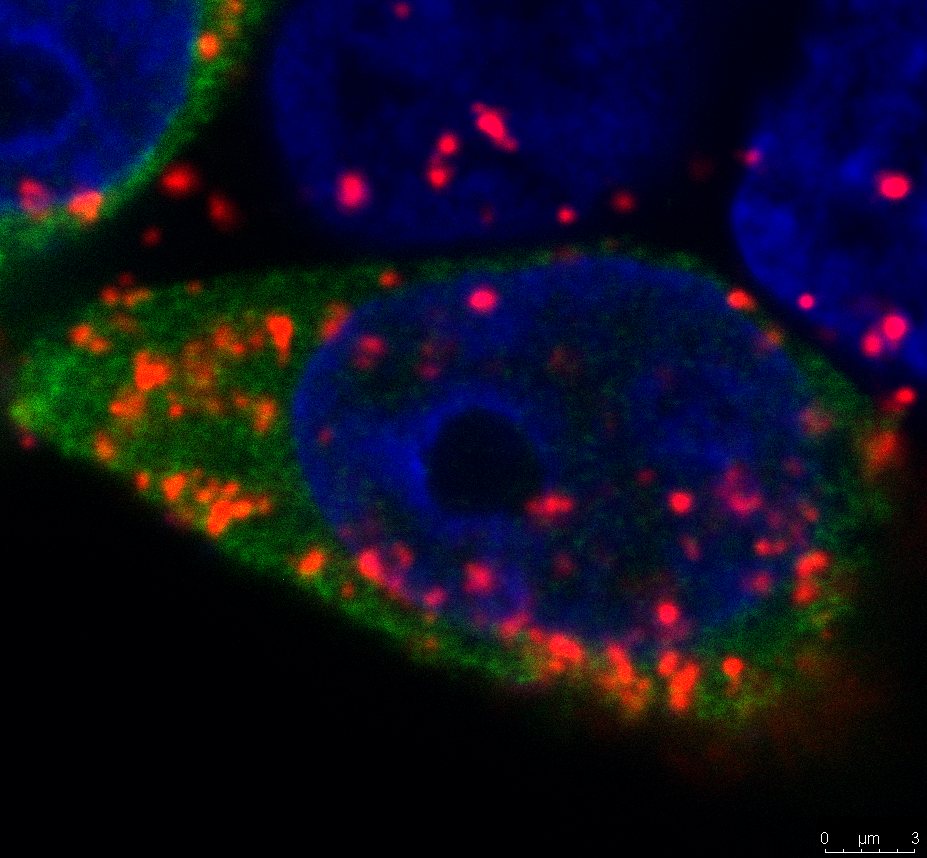

Supplement: Supplementary file 17 — Source Data for Figure 4 [file EMBJ-41-e111289-s003.zip › PLA/4K/4K-6.tif]

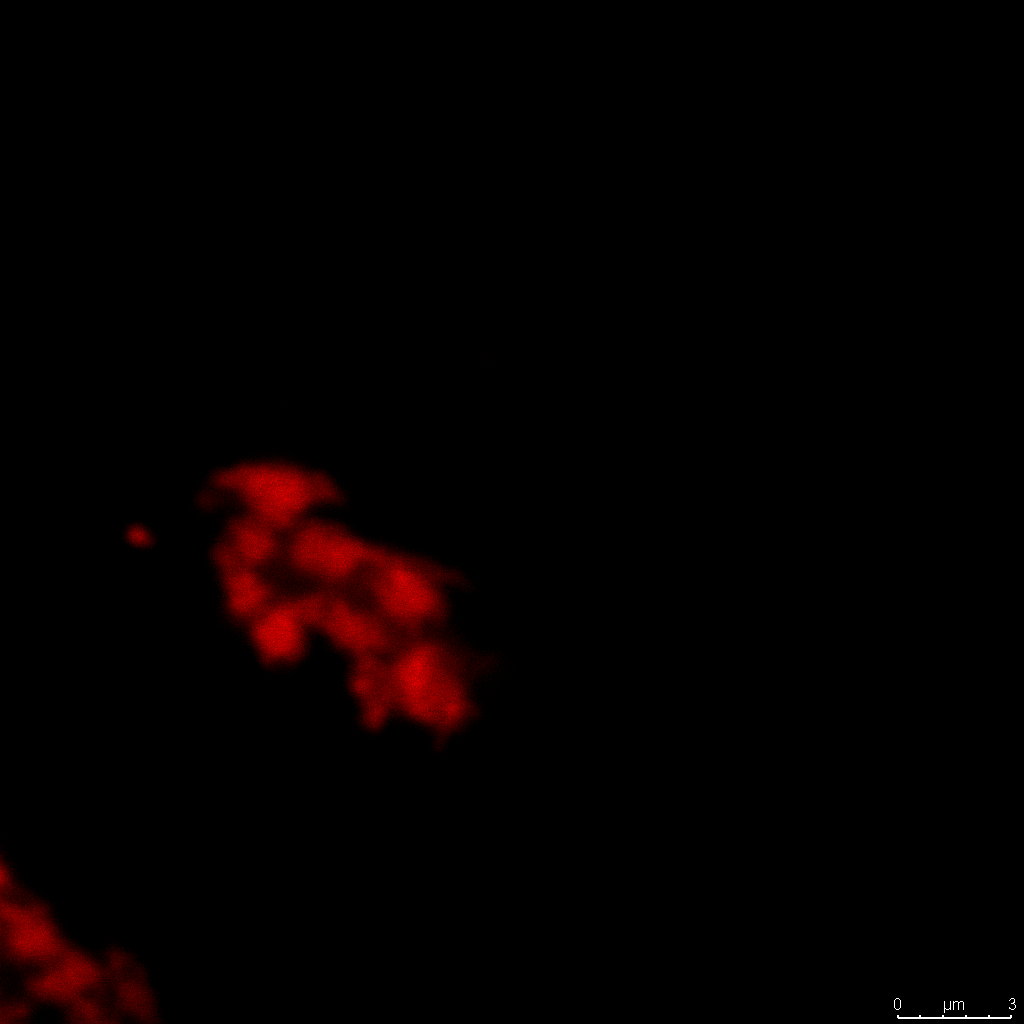

Supplement: Supplementary file 17 — Source Data for Figure 4 [file EMBJ-41-e111289-s003.zip › PLA/4K/4K-7.tif]

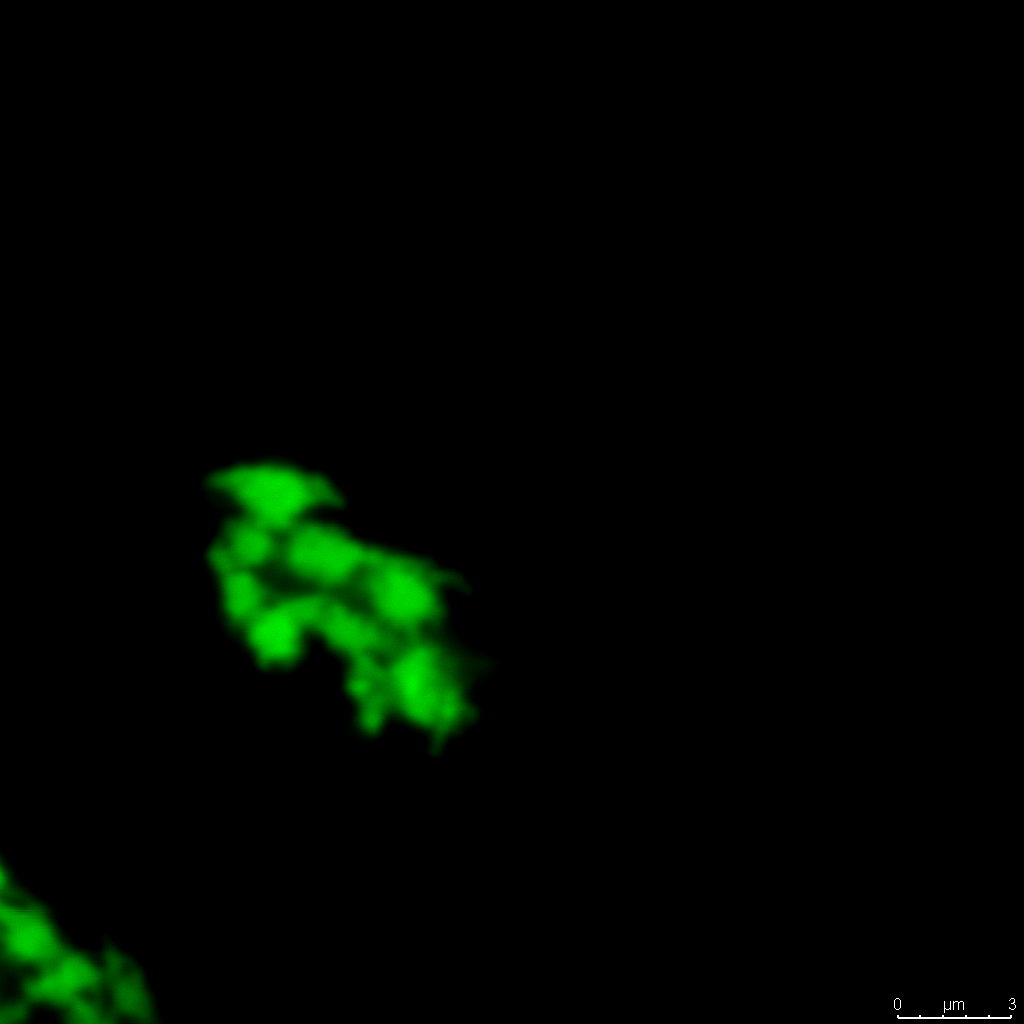

Supplement: Supplementary file 17 — Source Data for Figure 4 [file EMBJ-41-e111289-s003.zip › PLA/4K/4K-8.tif]

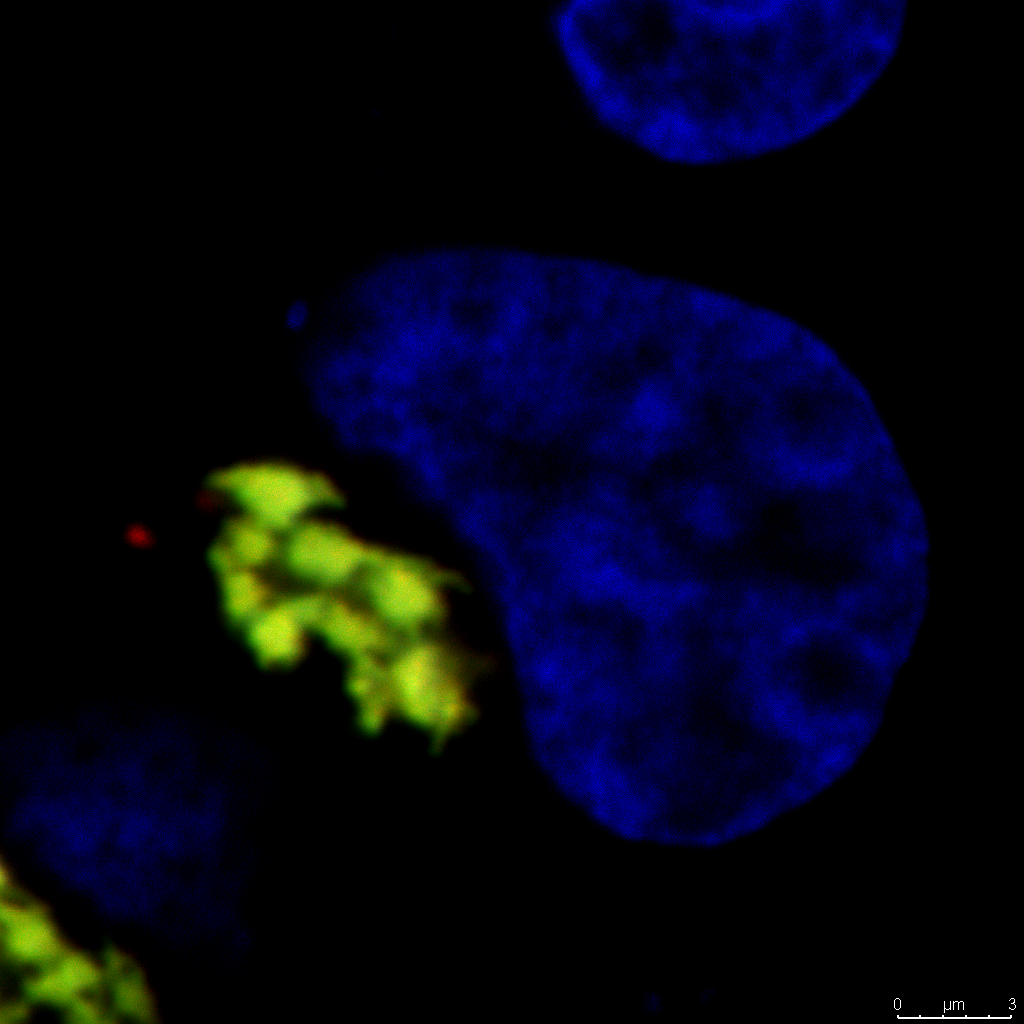

Supplement: Supplementary file 17 — Source Data for Figure 4 [file EMBJ-41-e111289-s003.zip › PLA/4K/4K-9.tif]

Figure-4

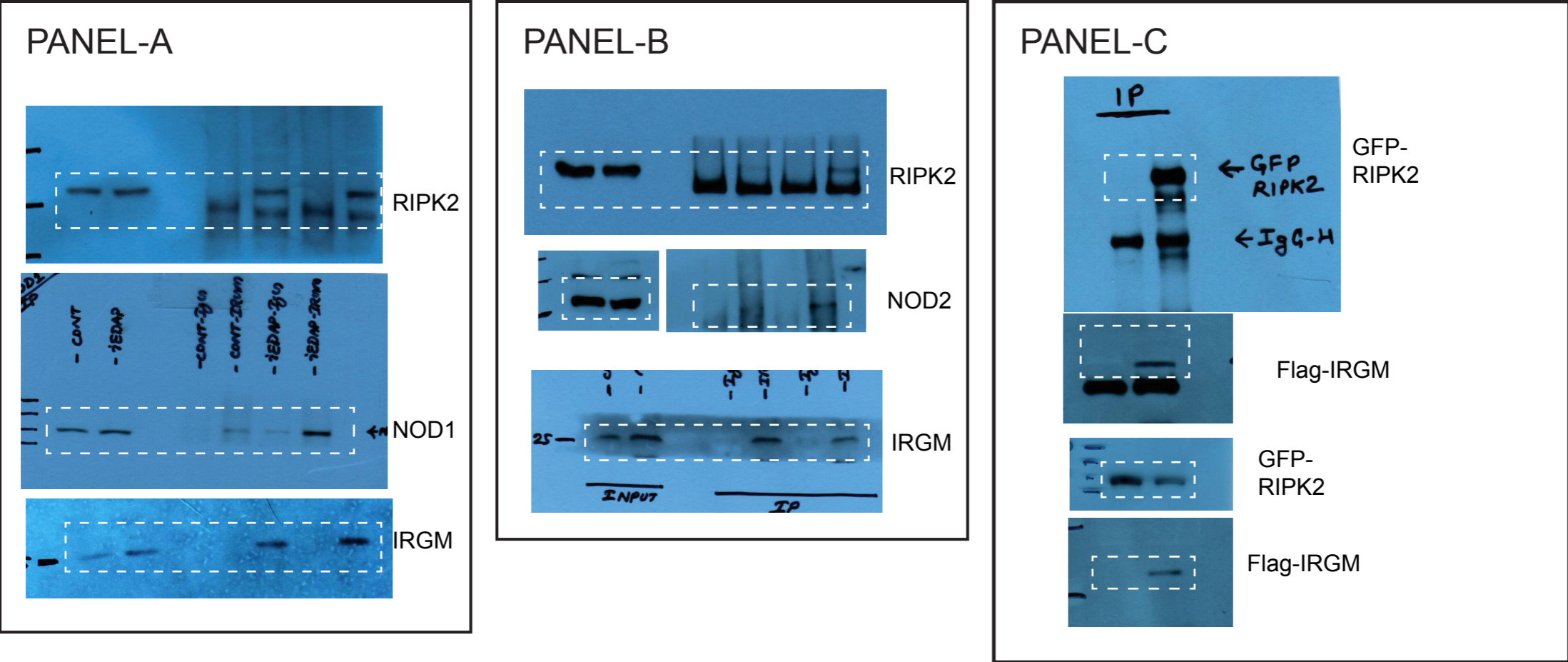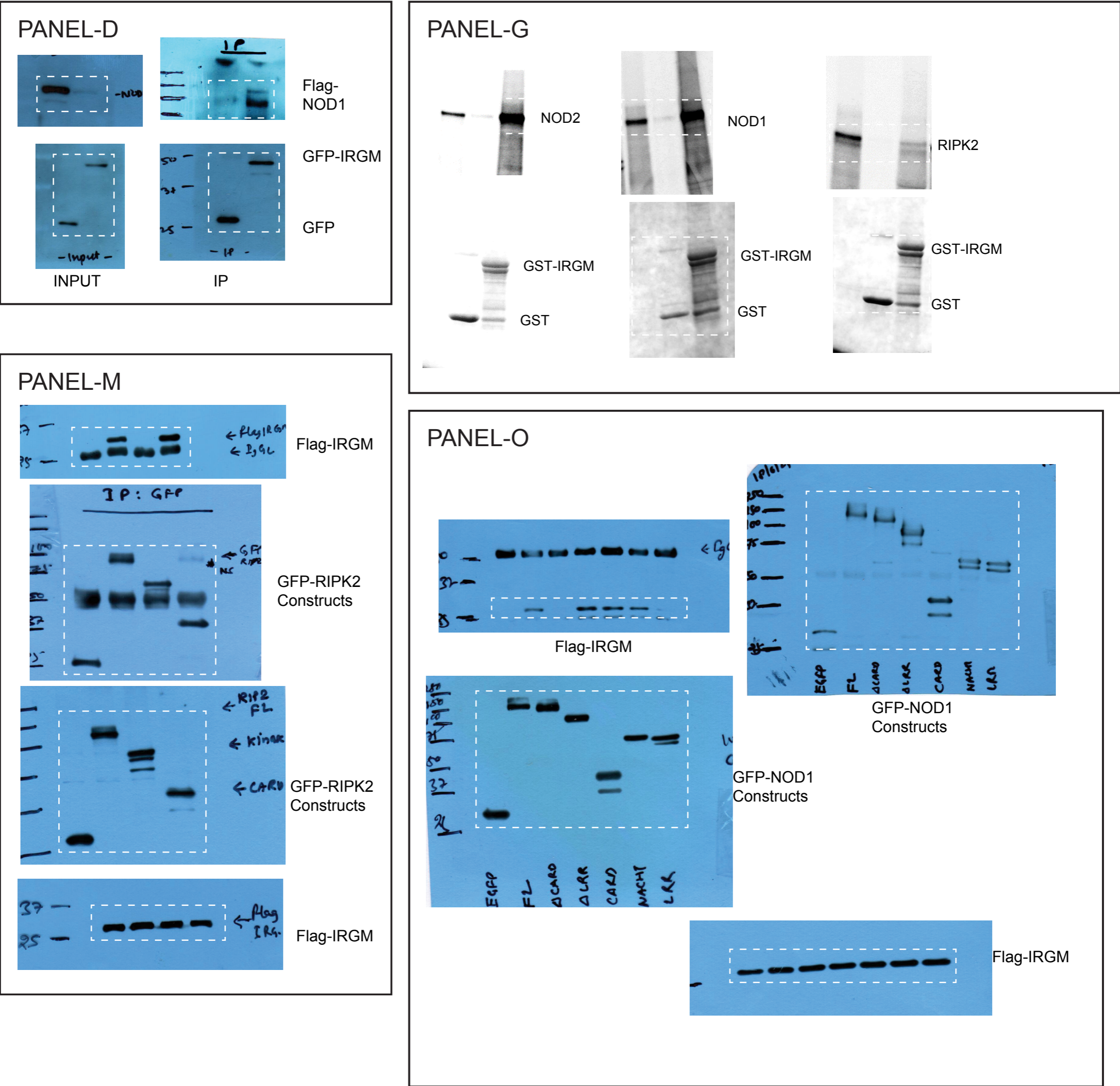

Supplement: Supplementary file 17 — Source Data for Figure 4 [file EMBJ-41-e111289-s003.zip › Western Blot/Western Blot Figure-4.pdf]

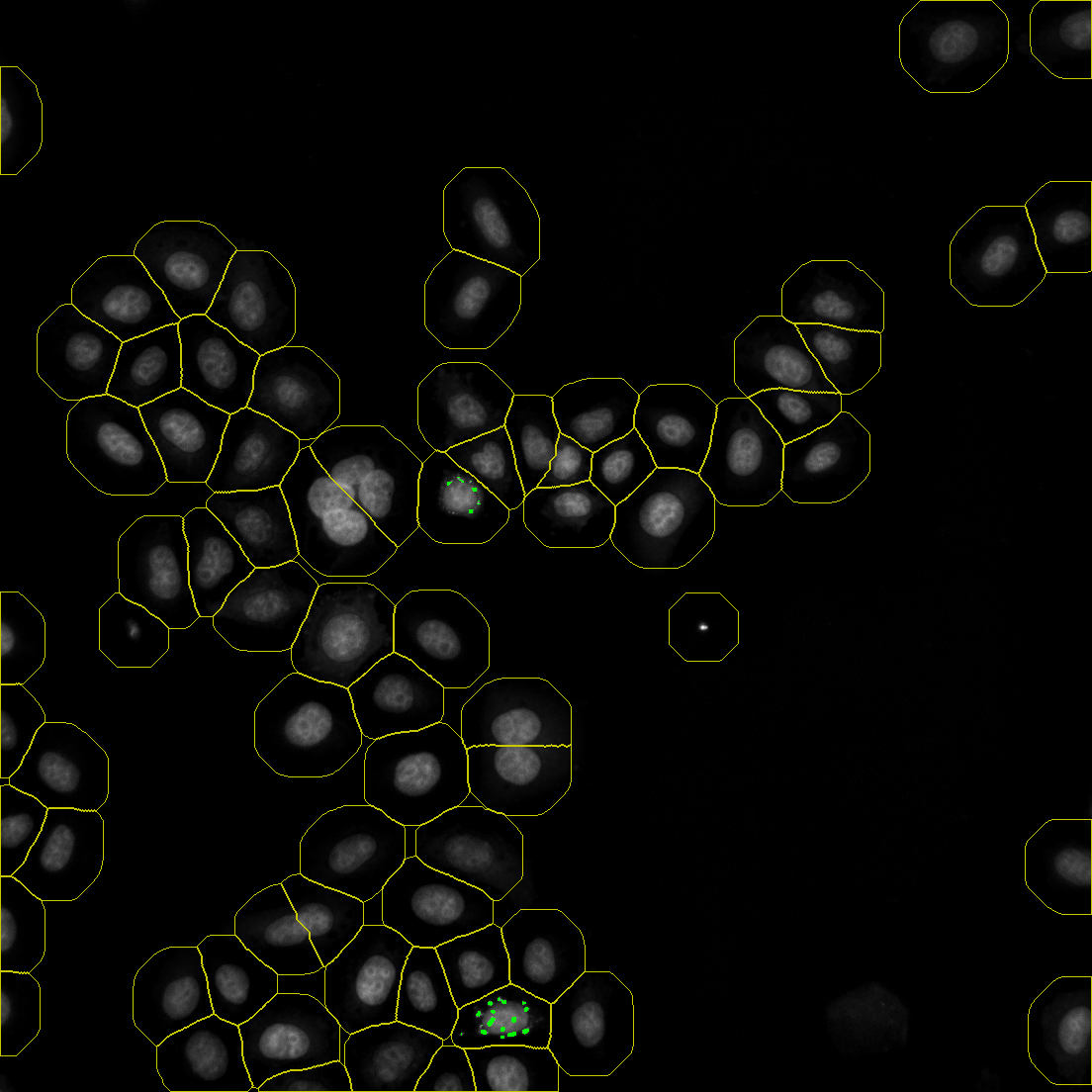

Supplement: Supplementary file 18 — Source Data for Figure 5 [file EMBJ-41-e111289-s014.zip › High Content Screening/5C/5C_siControl.bmp]

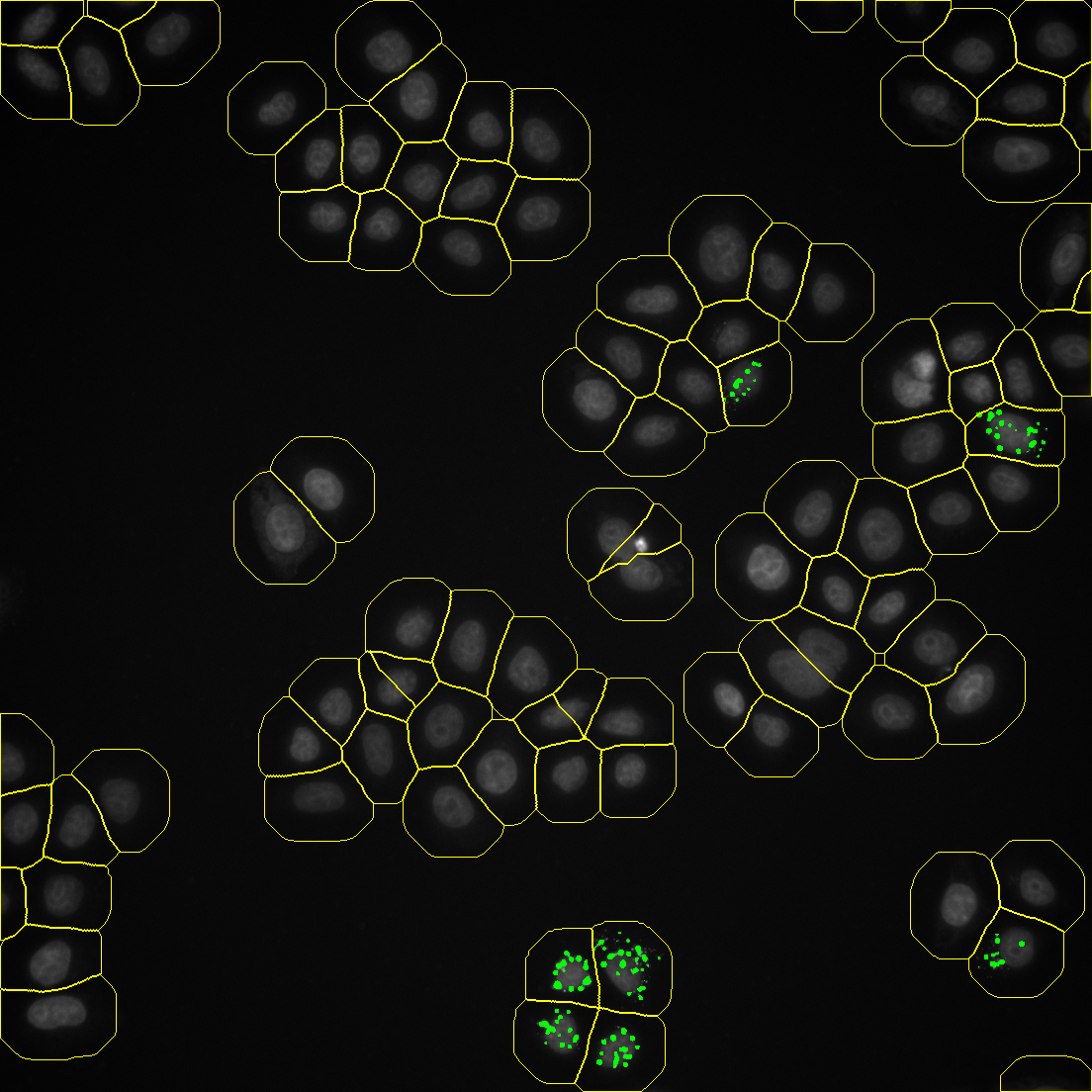

Supplement: Supplementary file 18 — Source Data for Figure 5 [file EMBJ-41-e111289-s014.zip › High Content Screening/5C/5C_siIRGM.bmp]
