# Supplementary figures and images for: Selective autophagy of RIPosomes maintains innate immune homeostasis during bacterial infection (part 2 of 2)
Source: EMBO J. 2022 Oct 11;41(23):e111289. doi: 10.15252/embj.2022111289 (PMC9713718; doi:10.15252/embj.2022111289)

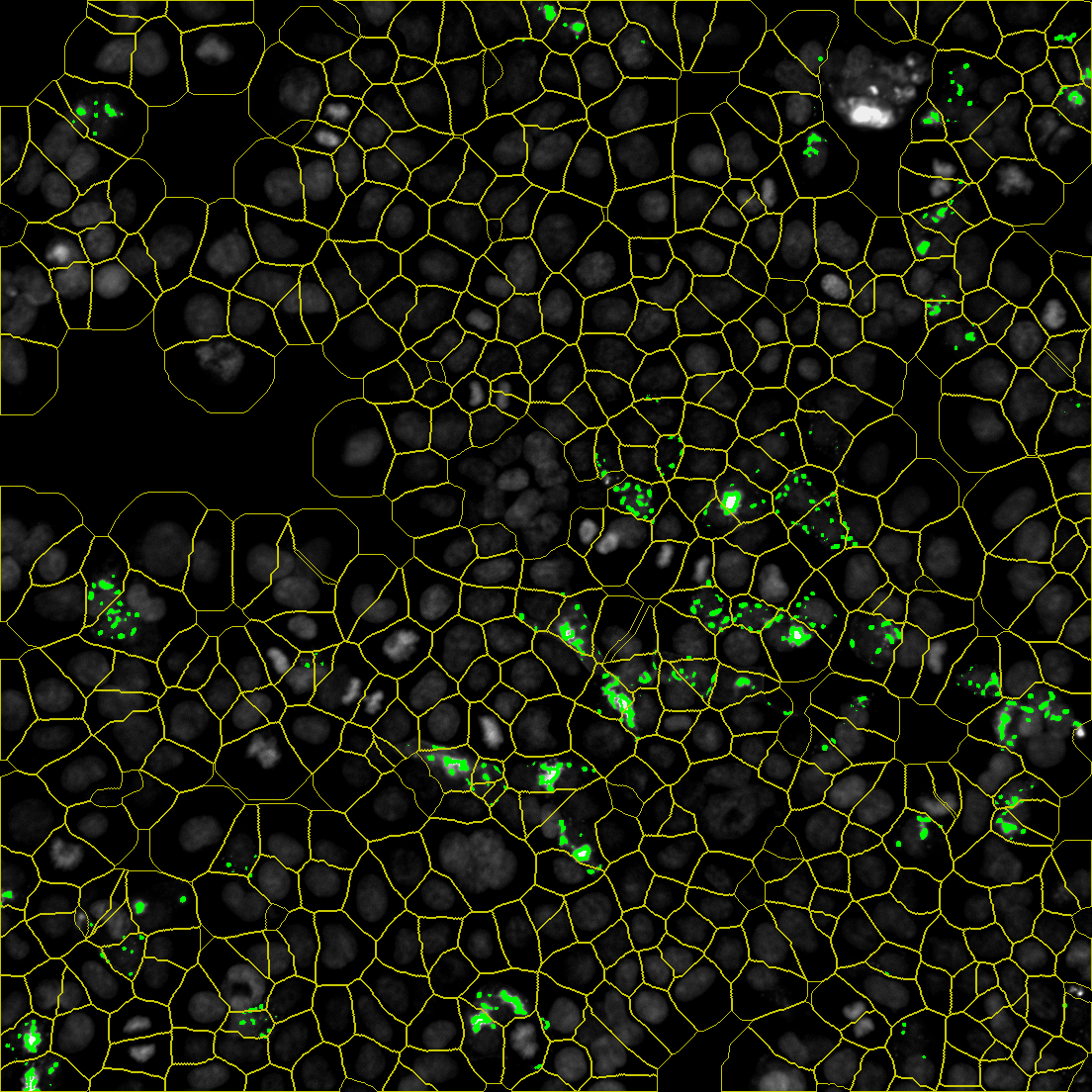

Supplement: Supplementary file 18 — Source Data for Figure 5 [file EMBJ-41-e111289-s014.zip › High Content Screening/5F/5F-1.bmp]

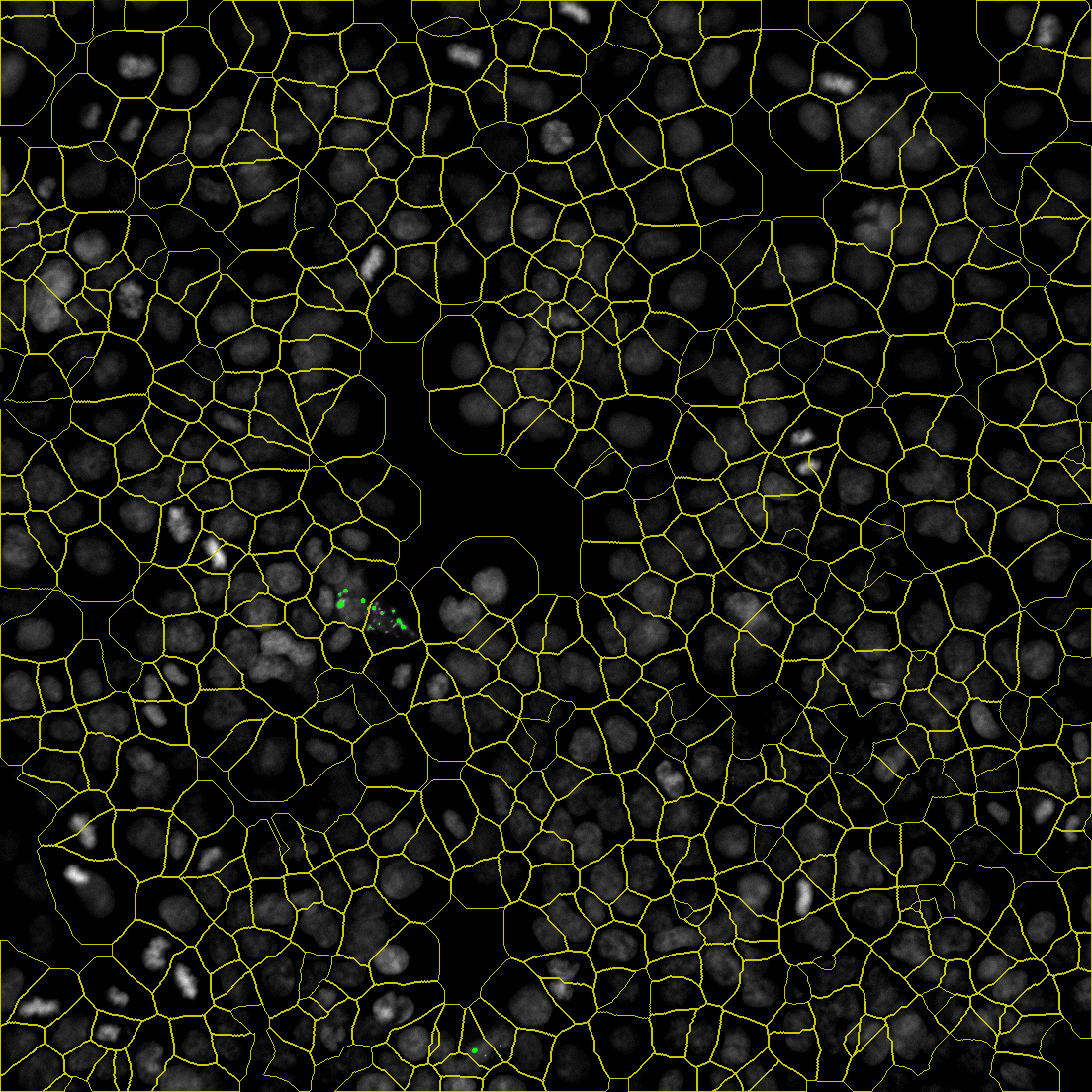

Supplement: Supplementary file 18 — Source Data for Figure 5 [file EMBJ-41-e111289-s014.zip › High Content Screening/5F/5F-2.bmp]

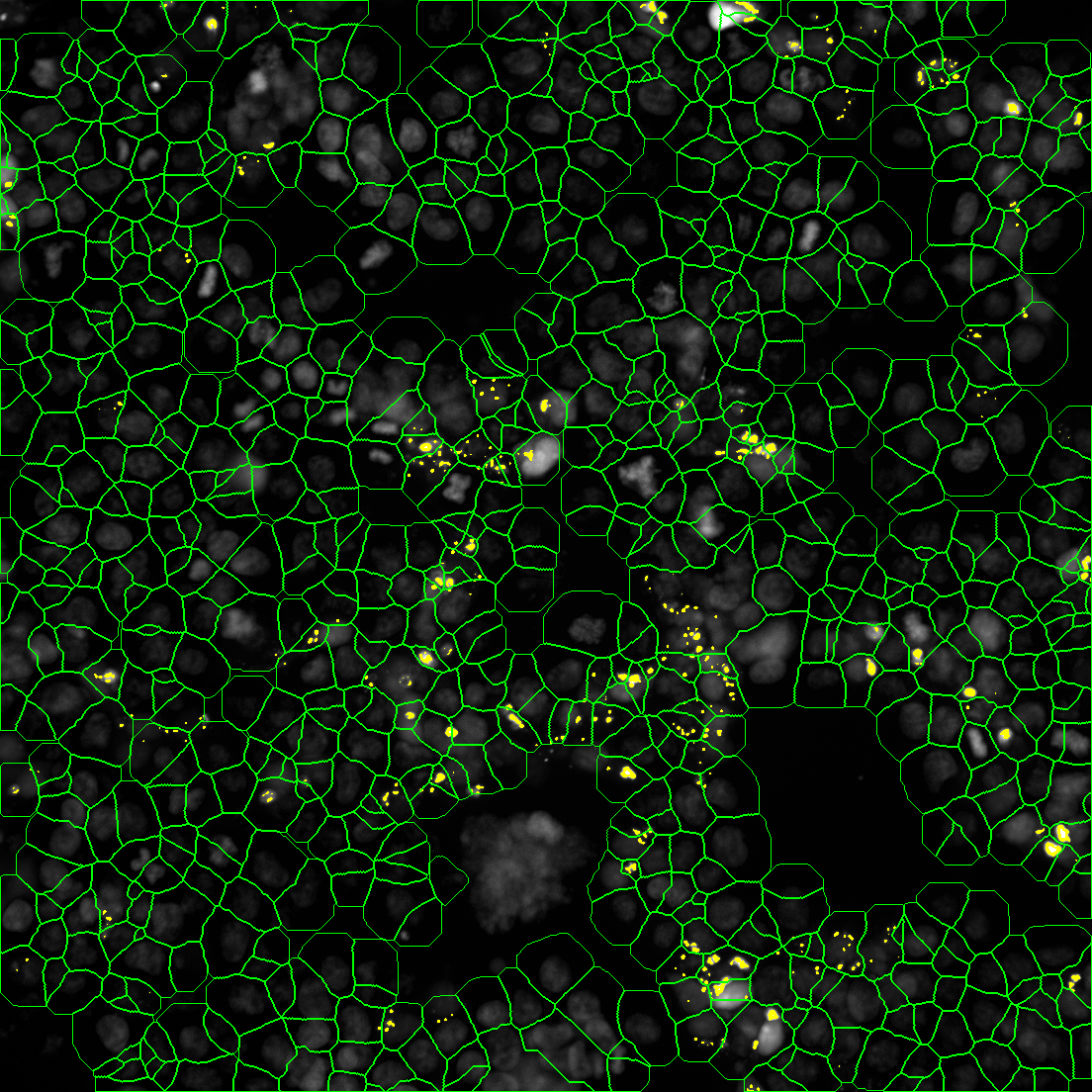

Supplement: Supplementary file 18 — Source Data for Figure 5 [file EMBJ-41-e111289-s014.zip › High Content Screening/5L/5L-1.bmp]

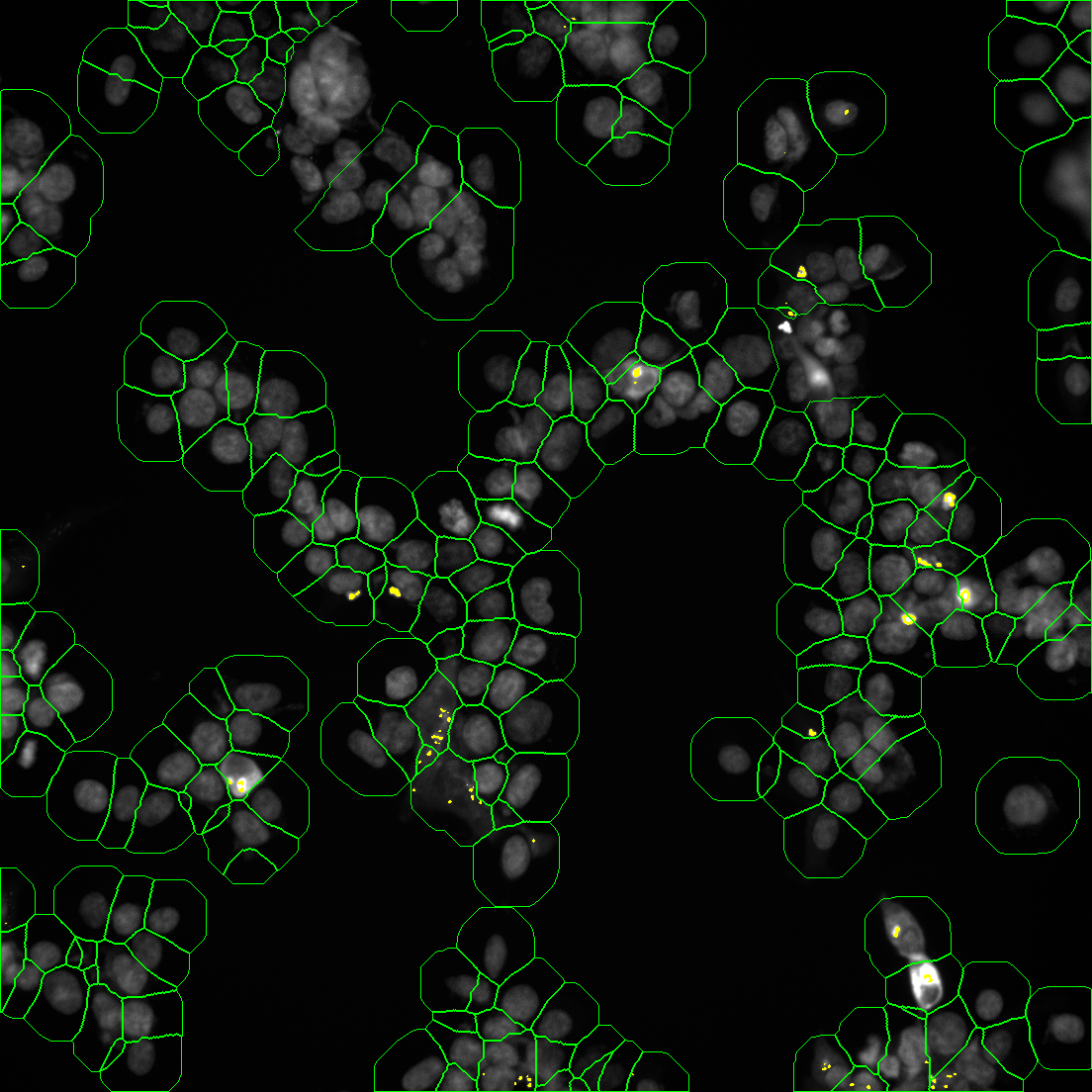

Supplement: Supplementary file 18 — Source Data for Figure 5 [file EMBJ-41-e111289-s014.zip › High Content Screening/5L/5L-2.bmp]

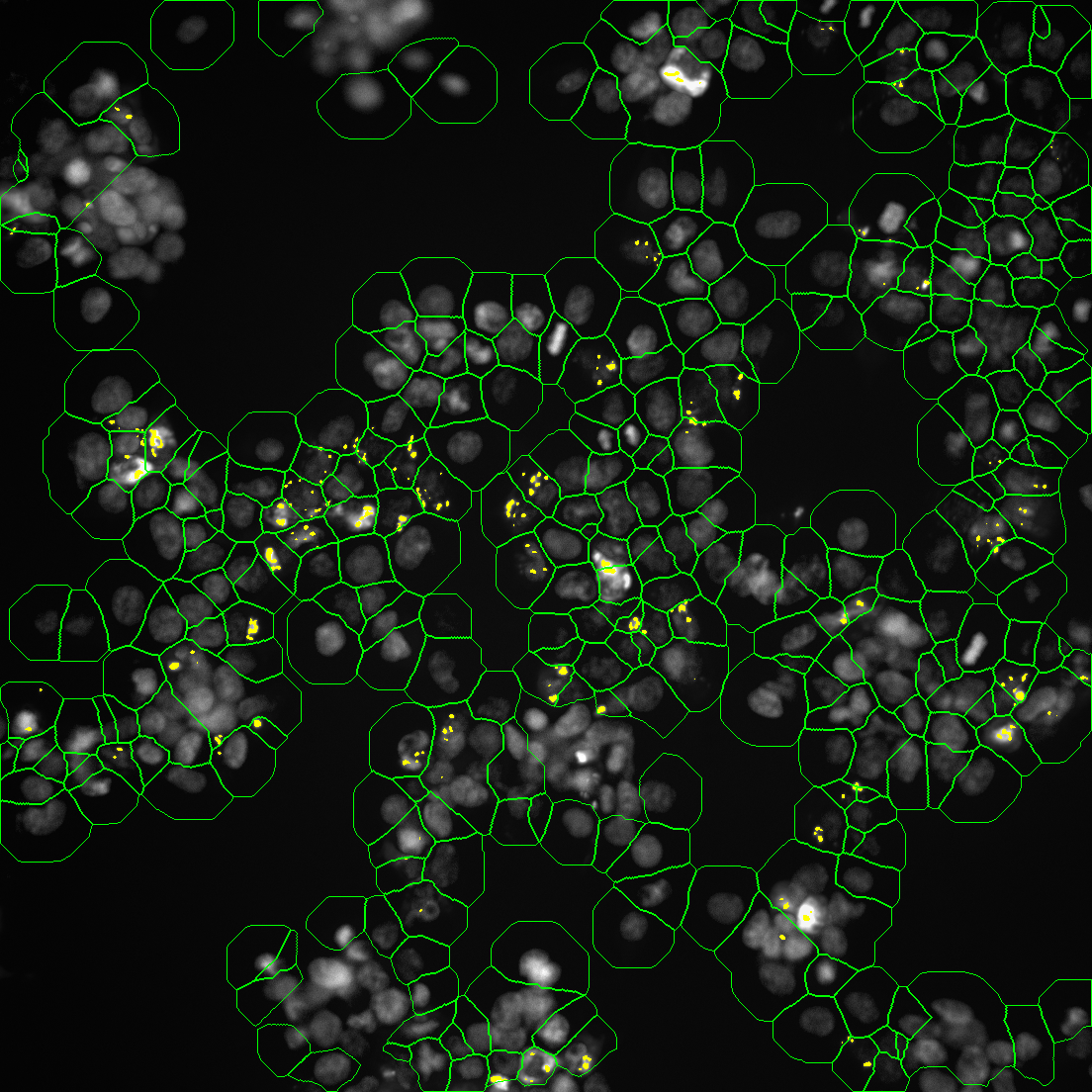

Supplement: Supplementary file 18 — Source Data for Figure 5 [file EMBJ-41-e111289-s014.zip › High Content Screening/5L/5L-3.bmp]

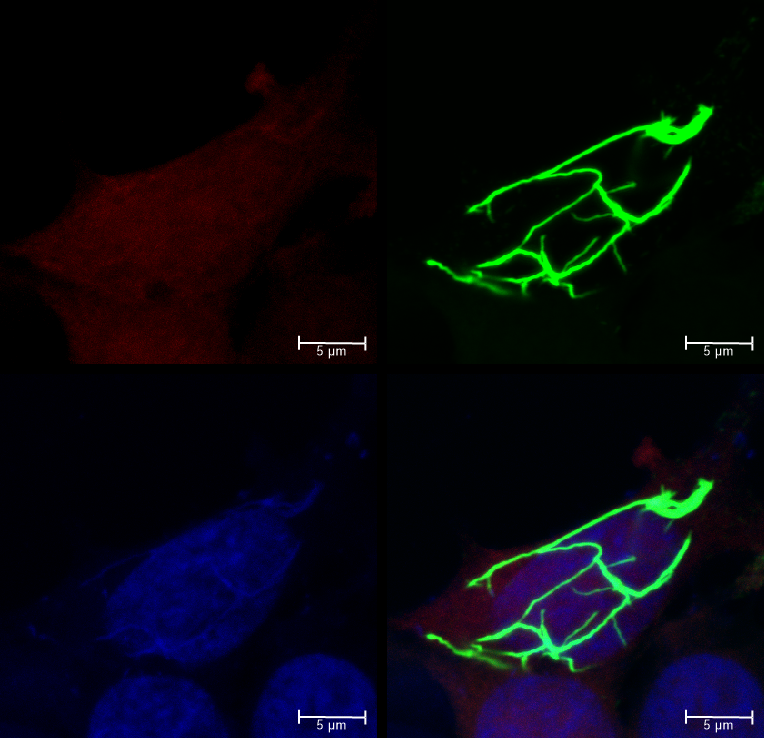

Supplement: Supplementary file 18 — Source Data for Figure 5 [file EMBJ-41-e111289-s014.zip › Microscopy_Confocal/5H/5H-A.tif]

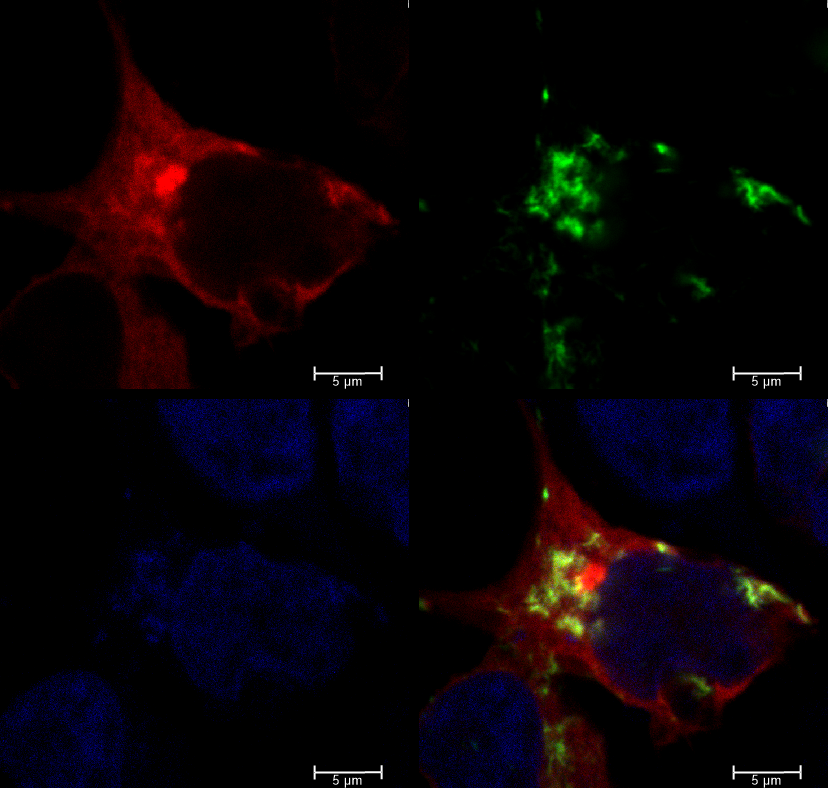

Supplement: Supplementary file 18 — Source Data for Figure 5 [file EMBJ-41-e111289-s014.zip › Microscopy_Confocal/5H/5H-B.tif]

Figure-5

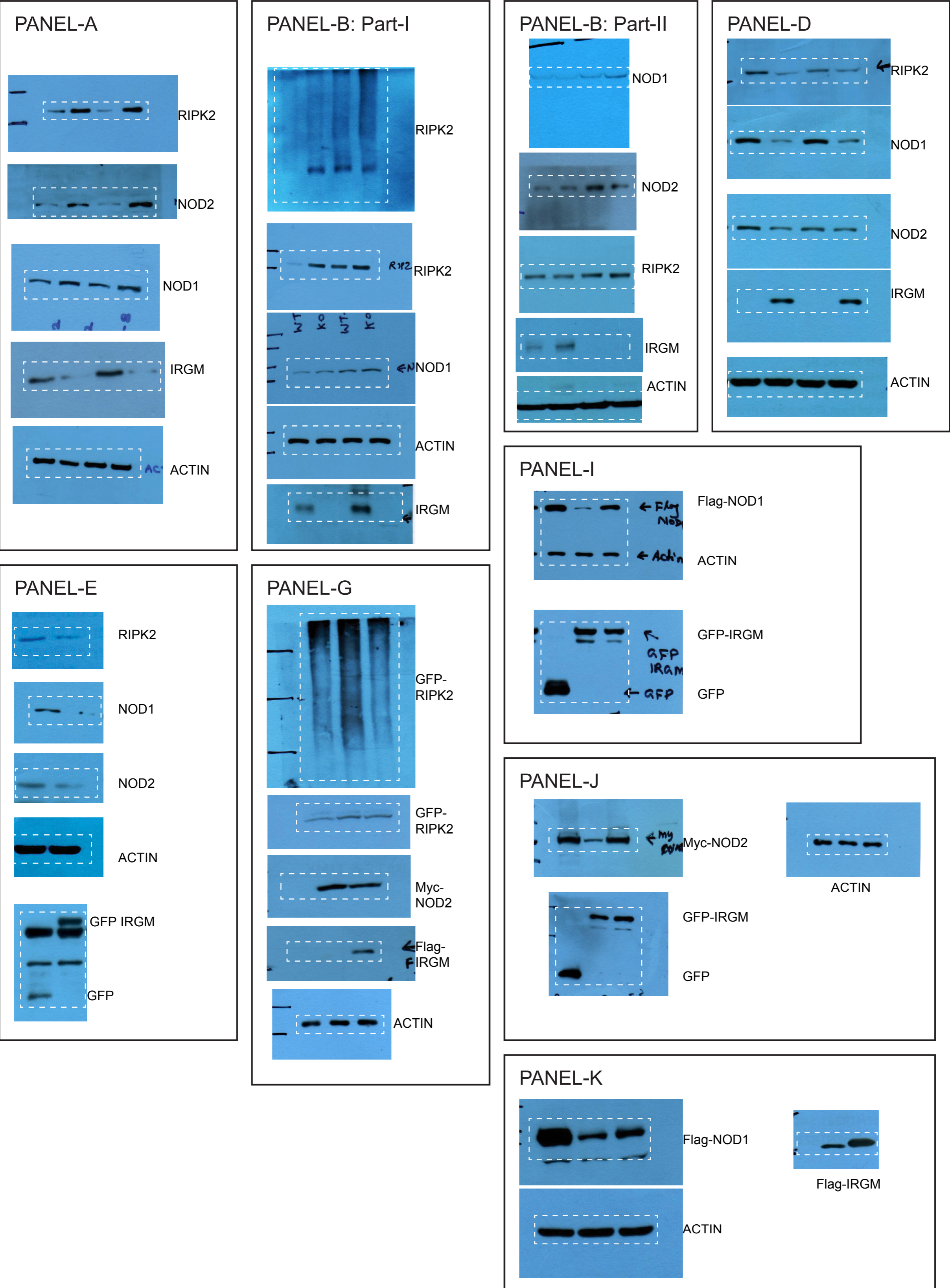

Supplement: Supplementary file 18 — Source Data for Figure 5 [file EMBJ-41-e111289-s014.zip › Western Blot/Western Blot Figure-5.pdf]

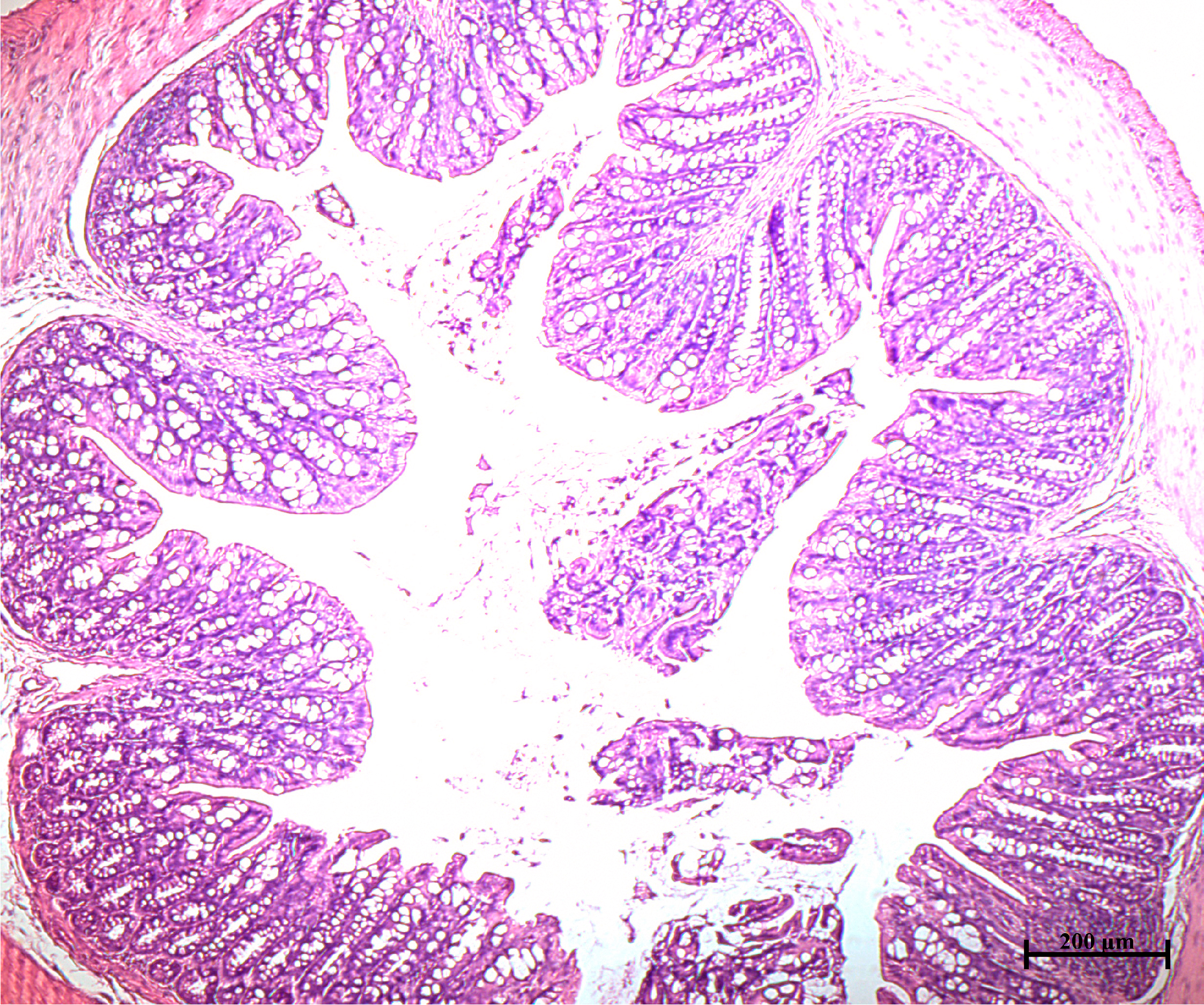

Supplement: Supplementary file 20 — Source Data for Figure 7 [file EMBJ-41-e111289-s006.zip › 7D/7D-3.jpg]

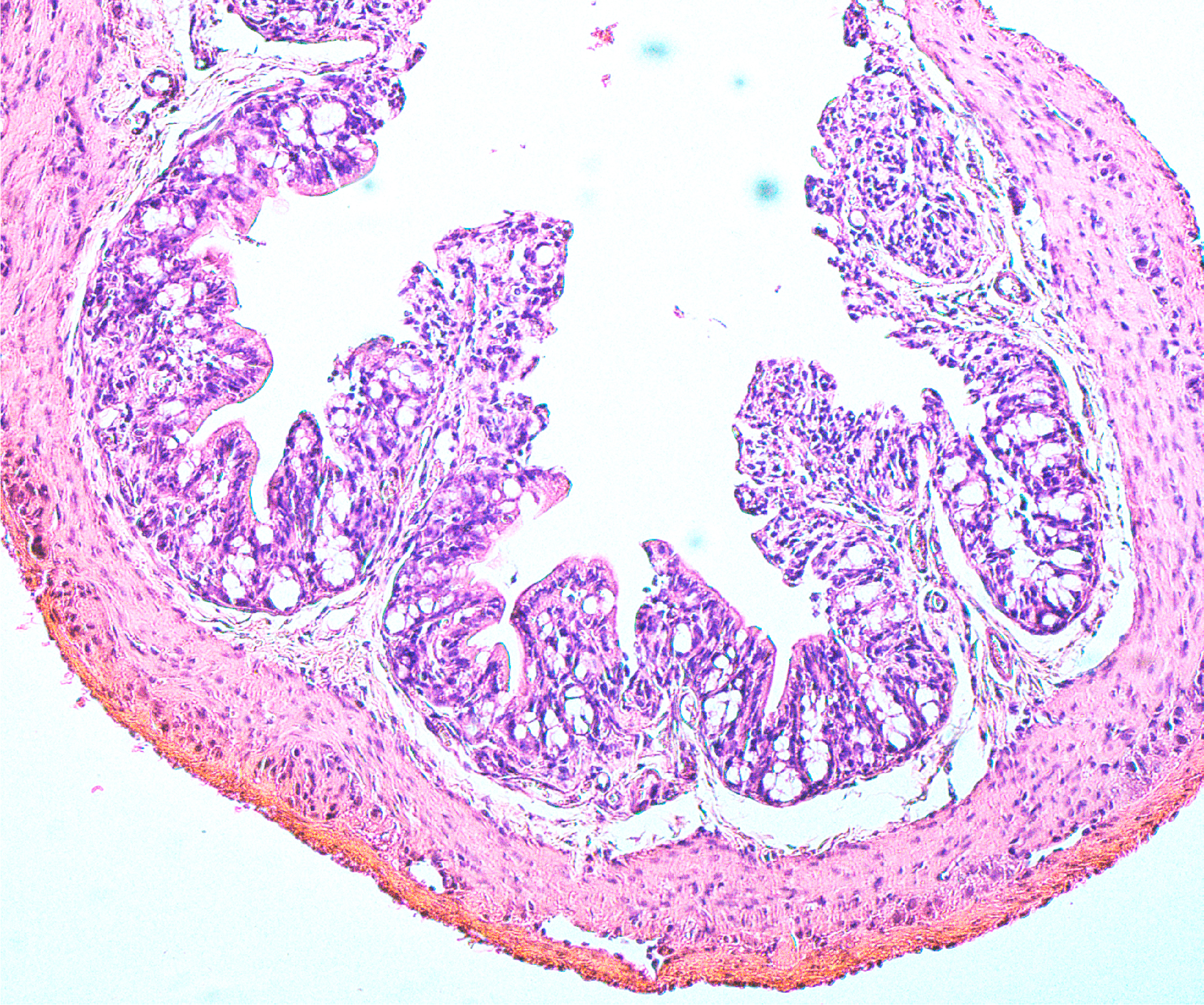

Supplement: Supplementary file 20 — Source Data for Figure 7 [file EMBJ-41-e111289-s006.zip › 7D/7D_1.jpg]

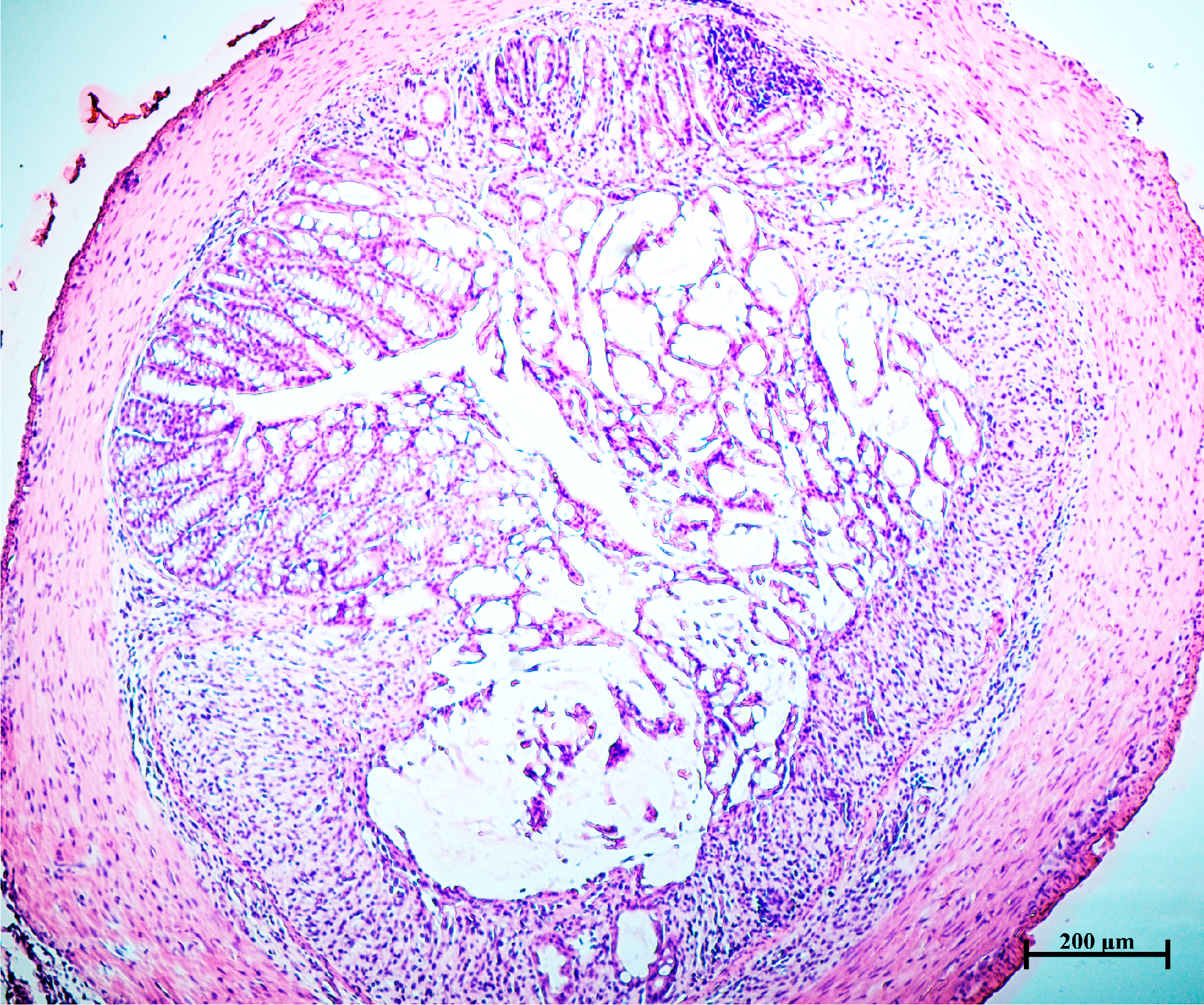

Supplement: Supplementary file 20 — Source Data for Figure 7 [file EMBJ-41-e111289-s006.zip › 7D/7D_2.jpg]

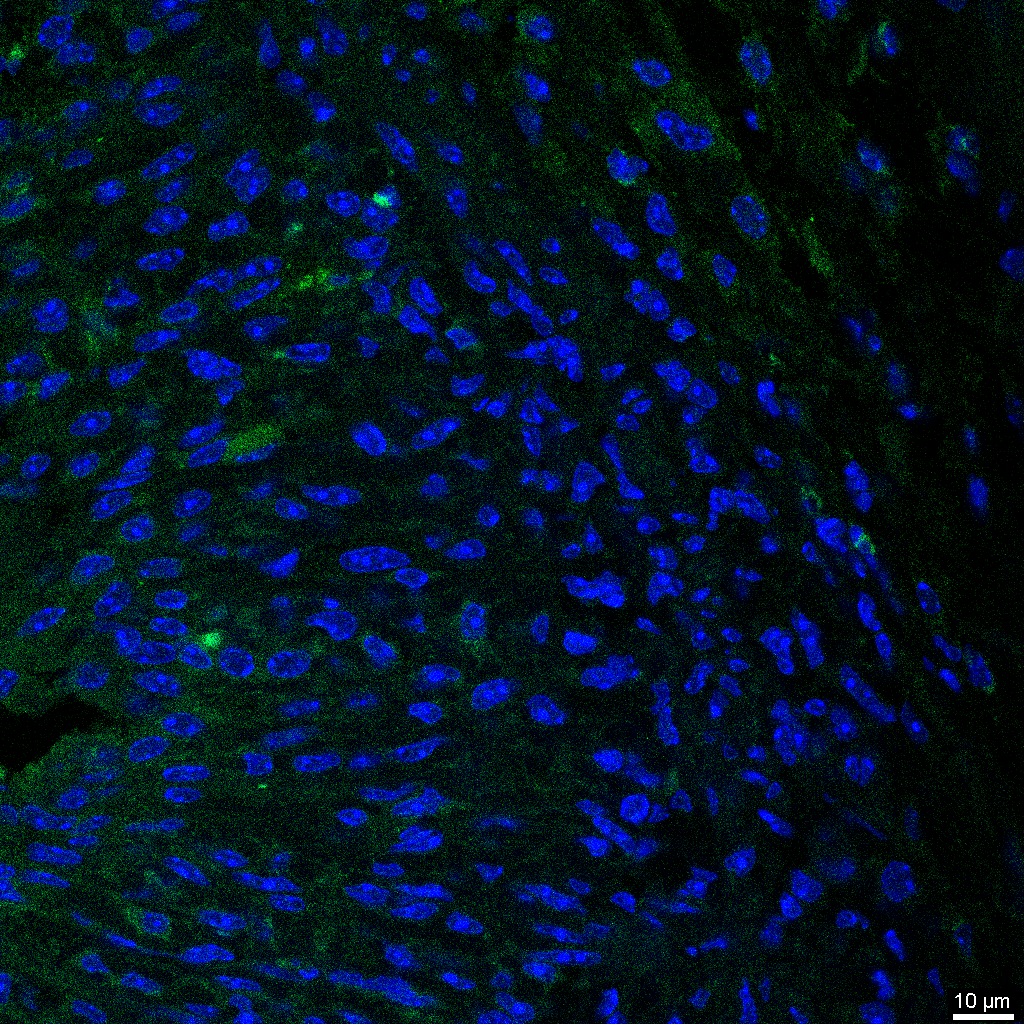

Supplement: Supplementary file 20 — Source Data for Figure 7 [file EMBJ-41-e111289-s006.zip › 7G/7G-1.tif]

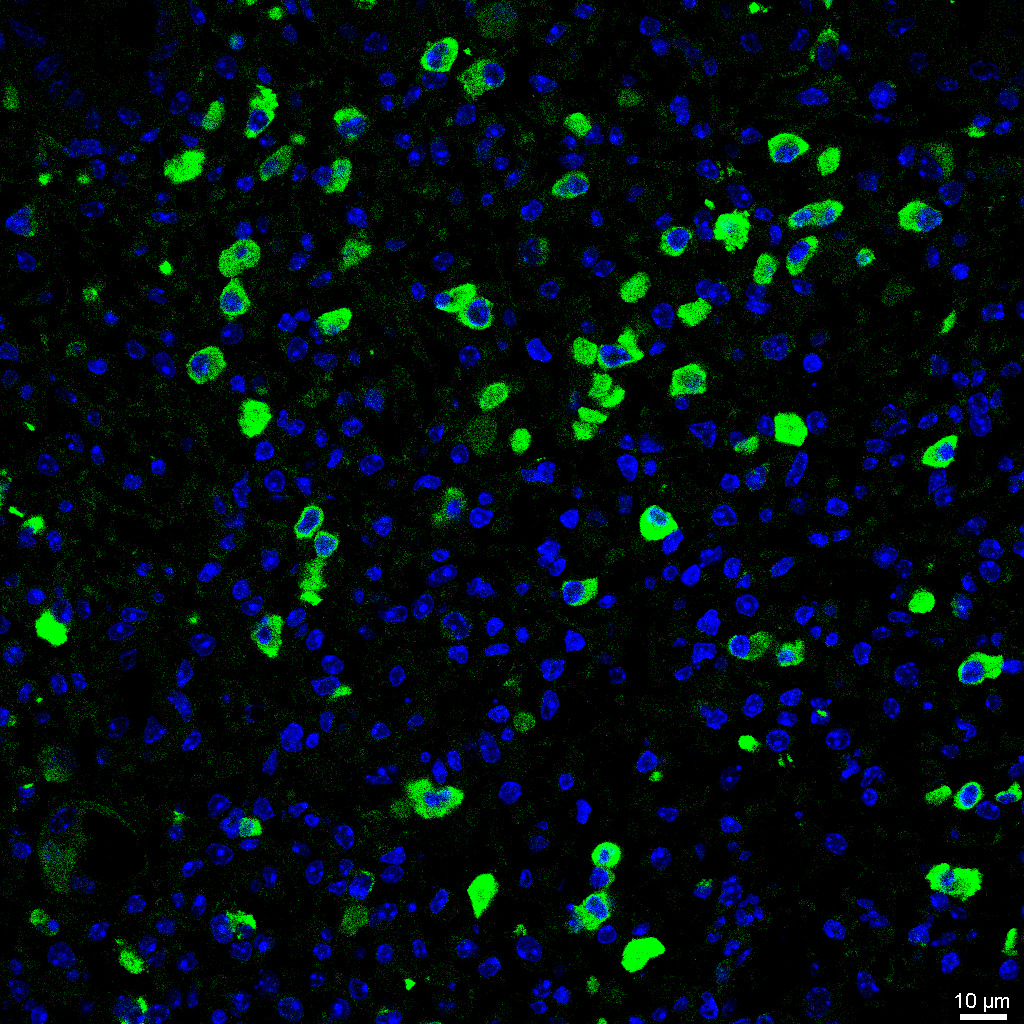

Supplement: Supplementary file 20 — Source Data for Figure 7 [file EMBJ-41-e111289-s006.zip › 7G/7G-2.tif]

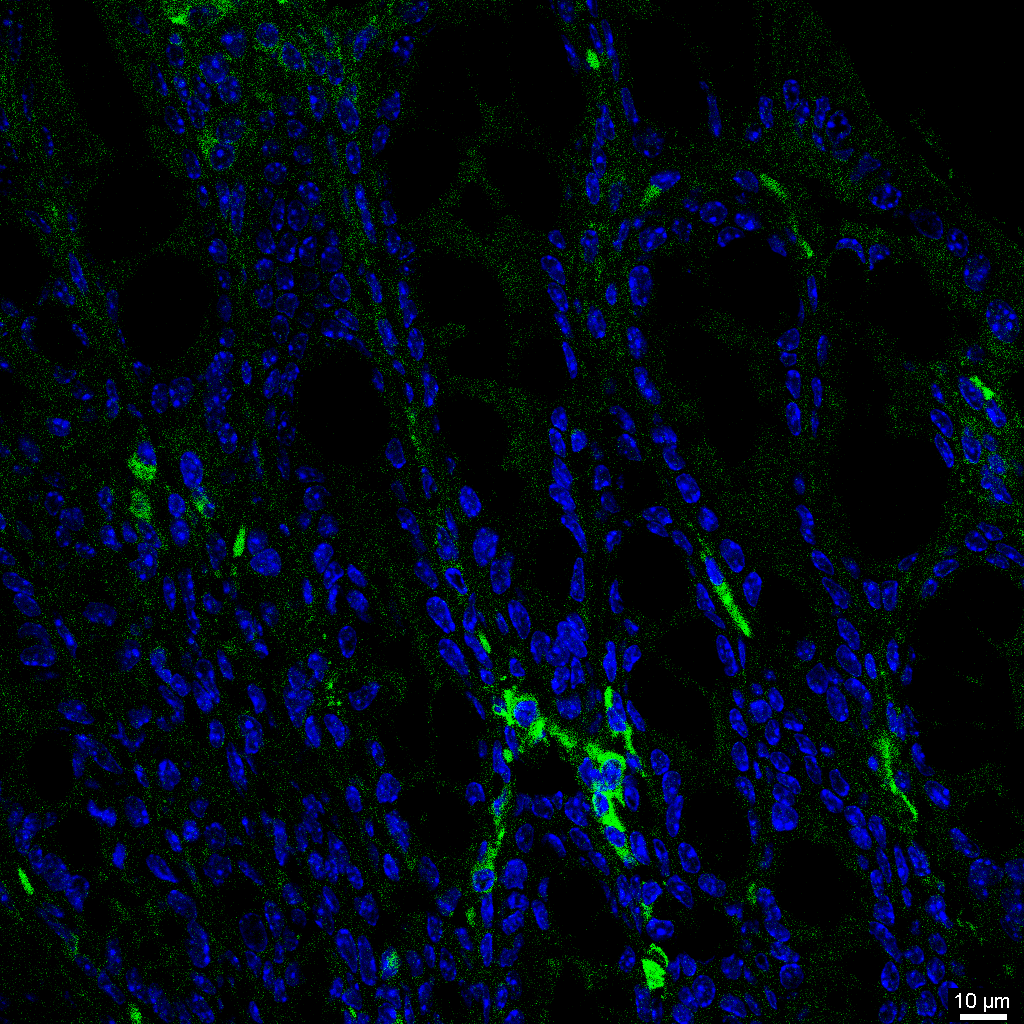

Supplement: Supplementary file 20 — Source Data for Figure 7 [file EMBJ-41-e111289-s006.zip › 7G/7G-3.tif]
