# Supplementary material for: Selective autophagy of RIPosomes maintains innate immune homeostasis during bacterial infection
Source: EMBO J. 2022 Oct 11;41(23):e111289. doi: 10.15252/embj.2022111289 (PMC9713718; doi:10.15252/embj.2022111289)
Supplement: Supplementary file 21 — PDF+ [file EMBJ-41-e111289-s018.pdf]

Article

SOURCE DATA | TRANSPARENT PROCESS | OPEN ACCESS

THE EMBO JOURNAL

Selective autophagy of RIPosomes maintains innate immune homeostasis during bacterial infection

Subhash Mehto[1,*,†], Kautilya Kumar Jena[1,†,‡], Rina Yadav[1,2], Swatismita Priyadarsini[3], Pallavi Samal[1], Sivaram Krishna[1,2], Kollori Dhar[1,2], Ashish Jain[4,5], Nishant Ranjan Chauhan[1], Krushna C Murmu[6], Ramyasingh Bal[1,7], Rinku Sahu[1,2], Pundrik Jaiswal[1], Bhabani Sankar Sahoo[3], Srinivas Patnaik[7], Thomas A Kufer[8], Tor Erik Rusten[4,5], Swati Chauhan[6], Punit Prasad[6] & Santosh Chauhan[1,9,**]

Abstract

The NOD1/2-RIPK2 is a key cytosolic signaling complex that activates NF-κB pro-inflammatory response against invading pathogens. However, uncontrolled NF-κB signaling can cause tissue damage leading to chronic diseases. The mechanisms by which the NODs-RIPK2-NF-κB innate immune axis is activated and resolved remain poorly understood. Here, we demonstrate that bacterial infection induces the formation of endogenous RIPK2 oligomers (RIPosomes) that are self-assembling entities that coat the bacteria to induce NF-κB response. Next, we show that autophagy proteins IRGM and p62/SQSTM1 physically interact with NOD1/2, RIPK2 and RIPosomes to promote their selective autophagy and limit NF-κB activation. IRGM suppresses RIPK2-dependent pro-inflammatory programs induced by *Shigella* and *Salmonella*. Consistently, the therapeutic inhibition of RIPK2 ameliorates *Shigella* infection- and DSS-induced gut inflammation in Irgm1 KO mice. This study identifies a unique mechanism where the innate immune proteins and autophagy machinery are recruited together to the bacteria for defense as well as for maintaining immune homeostasis.

Keywords autophagy; inflammation; Irgm1; NOD1/2-RIPK2-NF-κB; RIPosomes
Subject Categories Autophagy & Cell Death; Microbiology, Virology & Host Pathogen Interaction
DOI 10.15252/embj.2022111289 | Received 28 March 2022 | Revised 12 September 2022 | Accepted 15 September 2022 | Published online 11 October 2022
The EMBO Journal (2022) 41: e111289

Introduction

NOD1 and NOD2 (NODs) are cytosolic pattern recognition receptors (PRRs) that detect pathogen-associated molecular patterns (PAMPs), iE-DAP (D-glutamyl-meso-diaminopimelic acid), and MDP (muramyl dipeptide; Tanabe *et al*, 2004; Laroui *et al*, 2011; Caruso *et al*, 2014). Stimulated NODs interact and activate adaptor protein RIPK2 (RICK, RIP2) for a cascade of events resulting in NF-κB activation and pro-inflammatory cytokine release (Girardin *et al*, 2001; Caruso *et al*, 2014). NODs-RIPK2 is one of the major innate immune axis that senses intracellular bacterial pathogens and mounts a powerful NF-κB-dependent pro-inflammatory cytokine response to eliminate bacteria.

An important step for triggering pro-inflammatory innate immune pathways is the oligomerization of PRRs and/or adaptor proteins (Hou *et al*, 2011; Xie *et al*, 2013; Xu *et al*, 2014; Wu & Fuxreiter, 2016). For example, activation of RIG-I signaling induces polymerization of MAVS to form detergent-resistant, protease-resistant, and self-perpetuating prion-like aggregates (filamentous structure) to activate and transmit antiviral signaling (Hou *et al*, 2011; Cai *et al*, 2014; Wu *et al*, 2014; Xu *et al*, 2014). The inflammasome is another classical example, where several self-polymerized inflammatory proteins interact with each other to form multiprotein signalosomes that execute pro-inflammatory responses (Cai *et al*, 2014; Lu *et al*, 2014, 2016). Recently, structural studies demonstrated that purified RIPK2 polymerizes to form filamentous structures that are important for NODs-dependent NF-κB signaling (Gong *et al*, 2018; Pellegrini *et al*, 2018). Also, ectopically

1 Cell Biology and Infectious Diseases Unit, Department of Infectious Disease Biology, Institute of Life Sciences, Bhubaneswar, India
2 Regional Centre for Biotechnology, NCR Biotech Science Cluster, Faridabad, India
3 Institute of Life Sciences, Bhubaneswar, India
4 Centre for Cancer Cell Reprogramming, Institute of Clinical Medicine, Faculty of Medicine, University of Oslo, Oslo, Norway
5 Department of Molecular Cell Biology, Institute for Cancer Research, Oslo University Hospital, Oslo, Norway
6 Epigenetic and Chromatin Biology Unit, Institute of Life Sciences, Bhubaneswar, India
7 School of Biotechnology, KIIT University, Bhubaneswar, India
8 Department of Immunology, Institute of Nutritional Medicine, University of Hohenheim, Stuttgart, Germany
9 CSIR–Centre For Cellular And Molecular Biology (CCMB), Hyderabad, India
*Corresponding author. Tel: +91 7503157368; E-mail: subhash@ils.res.in
**Corresponding author. Tel: +91 7077702598; E-mail: schauhan@ils.res.in; schauhan@ccmb.res.in
†These authors contributed equally to this work
‡Present address: Division of Immunology, Boston Children's Hospital, Harvard Medical School, Boston, MA, USA

© 2022 The Authors. Published under the terms of the CC BY 4.0 license.

expressed RIPK2 was shown to form detergent-insoluble higher-order oligomeric structures upon *Shigella* infection (Ellwanger *et al*, 2019). These structures were termed “RIPosomes.” To date, it is not clear whether endogenous RIPK2 forms RIPosomes and if yes, how they regulate NF-κB signaling upon bacterial infection.

The NODs-RIPK2-NF-κB pro-inflammatory axis is critical for clearing pathogens; however, its aberrant activation can cause chronic inflammation, oncogenesis, and autoimmune disease (Miceli-Richard *et al*, 2001; Kanazawa *et al*, 2005; Henckaerts & Vermeire, 2007; Caso *et al*, 2015; Taniguchi & Karin, 2018). To avoid these conditions, a balanced immune state needs to be maintained by negative feedback mechanisms. Inflammophagy, the autophagic degradation of inflammatory aggregates and proteins, is an emerging mechanism to limit inflammation to maintain innate immune homeostasis (Chauhan *et al*, 2021; Deretic, 2021). Several of the cytosolic PRRs and adaptor proteins including RIG-I, NLRP3, AIM2, cGAS, MAVS, and STING are targeted by p62- or NDP52-dependent selective autophagy to dampen the excess inflammation (Shi *et al*, 2012; Liu *et al*, 2016; Du *et al*, 2018; Prabakaran *et al*, 2018; He *et al*, 2019; Mehto *et al*, 2019b; Jena *et al*, 2020; Chauhan *et al*, 2021; Deretic, 2021). To date, it is unclear how selective autophagy contributes to fine-tuning of the NODs-RIPK2-NF-κB pathways.

Genetic mutations in the Immunity-related GTPase M (IRGM) gene or promoter are suggested to increase susceptibility to several inflammatory and infectious diseases including Crohn’s disease, tuberculosis, sepsis, and ankylosing spondylitis (Massey & Parkes, 2007; Parkes *et al*, 2007; Intemann *et al*, 2009; Lu *et al*, 2013; Kimura *et al*, 2014; Lin *et al*, 2016; Xia *et al*, 2017; Yao *et al*, 2018). IRGM is also a critical factor for antimicrobial autophagy (Singh *et al*, 2006; Chauhan *et al*, 2015). IRGM interacts with autophagy and lysosome regulatory proteins to stimulate autophagosome/lysosome biogenesis for efficient degradation of cargo including microbes (Singh *et al*, 2010; Chauhan *et al*, 2015; Kumar *et al*, 2018, 2020). IRGM plays a prominent role in the inflammophagy of several innate immune sensing proteins including NLRP3, RIG-I, and cGAS to limit inflammasome and interferon responses (Mehto *et al*, 2019a, 2019b; Jena *et al*, 2020; Chauhan *et al*, 2021; Nath *et al*, 2021). IRGM interacts with NOD2 to scaffold the signaling events for xenophagy (Chauhan *et al*, 2015); however, it is unclear whether IRGM also could regulate the activity of NODs and RIPK2 (RIPosomes) to control NF-κB pro-inflammatory cytokine response.

Here, we demonstrate that pathogenic bacteria induce the formation of endogenous RIPosomes in the proximity of the bacteria to activate the NF-κB cytokine response. Further, we found that NODs, RIPK2, and RIPosomes are targets of selective autophagy. The autophagy scaffolding proteins, IRGM and p62 physically interact with NODs, RIPK2, and RIPosomes, and using the canonical autophagy machinery coordinate their selective degradation to limit cytokine responses. In agreement, the global transcriptomic analysis revealed that during *Salmonella* and *Shigella* infection, IRGM suppresses multiple RIPK2-dependent pro-inflammatory pathways including NF-κB and interferon (IFN) response. Consistently, in animal studies, inhibition of RIPK2 using GSK583 ameliorated shigellosis- and dextran sodium sulfate (DSS)-induced gut inflammation, and pathology in *Irgm1*^{KO} mice. Together, this study delineates new cell-autonomous mechanisms of NODs-RIPK2-dependent pro-inflammatory response and its resolution by selective autophagy. Further, our study also suggests that inhibition of RIPK2 could be a good therapeutic strategy for suppression of gut inflammation associated with IRGM depletion, a risk factor in the progression of Crohn’s disease.

Results

RIPosomes recruit over the bacteria

The existence of endogenous RIPK2 oligomeric structures (RIPosomes) is not reported. We found that infection of macrophage-like differentiated THP-1 cells with *Shigella flexneri* induces RIPosome formation. RIPosomes were detected only in infected cells but not in control cells (Appendix Fig S1A). Several of the RIPosomes were recruited over the bacteria (Fig 1A and Appendix Fig S1A–C and Movie EV1). However, not all the intracellular bacteria were covered with oligomeric RIPK2 puncta (Fig 1A). High-content microscopy was performed to quantitate the RIPosomes present in the cell (Fig 1B and Appendix Fig S1D). The presence of endogenous RIPK2 oligomeric structures was further confirmed by Western blotting of detergent-insoluble fractions from *Shigella*-infected cells (Fig 1C). To confirm specificity, next we depleted RIPK2 and tested the formation of RIPosomes by high-content microscopy and Western blotting. *Shigella*-induced RIPosomes were dramatically reduced upon RIPK2 siRNA knockdown in THP-1 macrophages (Fig 1D and E) or RIPK2 CRISPR knockout HT-29 cells (henceforth, RIPK2$^{-/-}$; Fig 1F).

Next, we infected mouse bone marrow-derived macrophages (BMDMs), HT-29 colon epithelial cells, and mouse embryonic fibroblast (MEF) cells with *Shigella*. RIPosomes were recruited over and adjacent to the bacteria in the infected cells (Fig 1G and Appendix Fig S1E–G), whereas the uninfected cells were devoid of them (Appendix Fig S1F). Thus, *Shigella* infection can induce RIPosome formation in different cell types. Next, C57BL/6 mice were infected by the intraperitoneal injection of *Shigella* (Yang *et al*, 2014), and immunohistochemistry with the colon tissues was performed. Several of the bacteria were covered with RIPosomes in colon tissues (Fig 1H).

To test whether RIPosome formation is specific to *Shigella* infection or is a generalized event during bacterial infection, we infected THP-1 cells with *Salmonella typhimurium*. Like *Shigella*, *Salmonella* enhanced the oligomerization of RIPK2 (Appendix Fig S1H) and triggered RIPosome formation (Fig 1I and J). Furthermore, infection with the Crohn’s disease-associated adherent invasive *Escherichia coli* (AIEC) strain LF82 (Glasser *et al*, 2001) also prompted the formation of RIPosomes that were coating the bacteria (Fig 1K). Next, we compared RIPosome inducing capacity of pathogenic *Mycobacterium tuberculosis* (*M.tb*, H37Rv) and nonpathogenic *Mycobacterium smegmatis* (*M. smeg*, MC^{2}155). Surprisingly, only *M.tb* triggered the massive formation of RIPosomes over the bacteria (Fig 1L and M and Appendix Fig S1I and Movie EV2), indicating that only pathogenic bacteria could activate RIPosome formation. To further corroborate this notion, we compared the RIPK2 oligomerization capacity of nonpathogenic *E. coli* DH5α and pathogenic AIEC LF82. Indeed, LF82 was more efficient in triggering the oligomerization of RIPK2 (Fig 1N).

 © 2022 The Authors

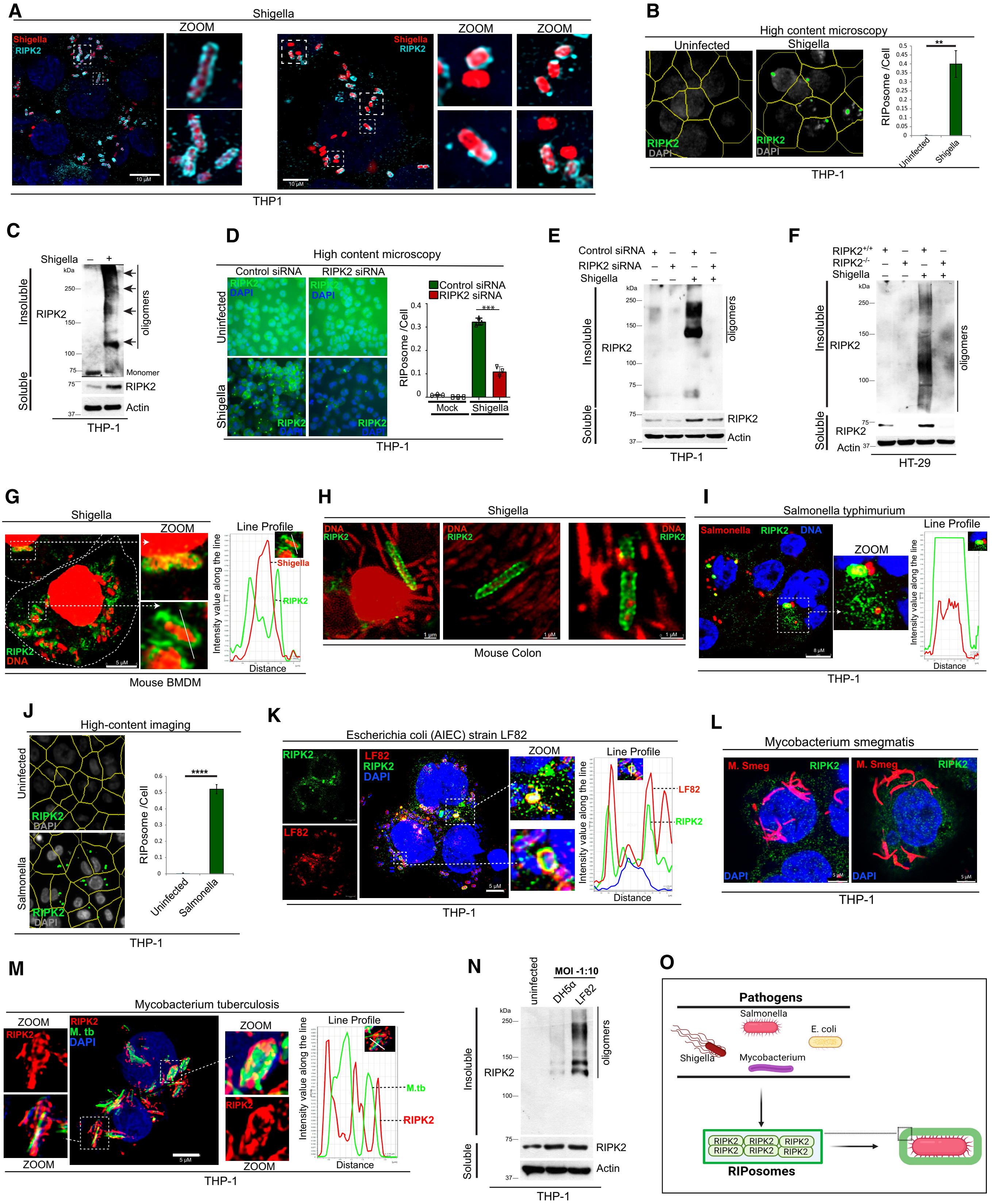
A
Shigella
ZOOM
ZOOM
ZOOM
Shigella
RIPK2
10 μM
THP1
B
High content microscopy
Uninfected
Shigella
RIPK2
DAPI
RIPosome /Cell
THP-1
C
Shigella
Insoluble
Soluble
RIPK2
oligomers
Monomer
Actin
THP-1
D
High content microscopy
Control siRNA
RIPK2 siRNA
Uninfected
Shigella
Mock
THP-1
E
Control siRNA
RIPK2 siRNA
Insoluble
Soluble
RIPK2
oligomers
Actin
THP-1
F
RIPK2+/+
RIPK2-/-
Shigella
HT-29
G
Shigella
ZOOM
Line Profile
Intensity value along the line
Distance
RIPK2
DNA
Mouse BMDM
H
Shigella
DNA
RIPK2
Mouse Colon
I
Salmonella typhimurium
Salmonella
DNA
THP-1
J
High-content imaging
Uninfected
Salmonella
K
Escherichia coli (AIEC) strain LF82
LF82
DAPI
L
Mycobacterium smegmatis
M. Smeg
M
Mycobacterium tuberculosis
M. tb
M.tb
N
uninfected
MOI -1:10
DH5α
O
Pathogens
E. coli
Mycobacterium
RIPosomes

Figure 1.

Figure 1. Pathogenic bacteria trigger the formation of RIPosomes that recruit over the bacteria.

A Representative confocal images of RIPosomes in THP-1 cells infected with a red fluorescent protein (RFP) expressing *Shigella flexneri* (MOI 1:25, 8 h). Scale bar, 10 μm. Zoom panels are digital magnifications.

B Representative high-content microscopy images (see Appendix Fig S1D for full images; yellow masks represent software algorithms-defined cell boundaries) of RIPosomes in THP-1 cells infected with *S. flexneri* (MOI 1:25, 8 h). About 50,000 cells were plated per well and RIPosomes were screened in 35 fields per well in three technical replicates. Right panel, the graph depicts an average number of RIPosomes/cell, which is calculated from three biological replicates, Mean ± SD. ***$P < 0.005$, Student's unpaired *t*-test.

C Western blot analysis of soluble and insoluble fractions of *S. flexneri*-infected THP-1 cells (MOI 1:25, 8 h). Arrow indicates oligomers of RIPK2.

D Left panel, representative high-content microscopy image of control and RIPK2 knockdown THP-1 cells infected with *S. flexneri* (MOI 1:25, 8 h). Right panel, the graph depicts an average number of RIPosomes/cell, which is calculated from three biological replicates, Mean ± SD. ***$P < 0.0005$, Student's unpaired *t*-test.

E, F Western blot analysis of soluble and insoluble fractions of *S. flexneri*-infected (E) control siRNA and RIPK2 siRNA transfected THP-1 cells (F) Control CRISPR ($RIPK2^{+/+}$) cells and RIPK2 CRISPR knockout ($RIPK2^{-/-}$) HT-29 cells.

G Representative confocal images of RIPosomes in mouse BMDMs infected with *S. flexneri* (MOI 1:25, 8 h). Line profile: co-localization analysis using line intensity profile. Scale bar, 5 μm. Zoom panels are digital magnifications. DNA is stained with DAPI (pseudo-colored red for better contrast).

H Representative confocal images of mouse colon tissues showing recruitment of RIPK2 over the *S. flexneri*. Scale bar, 1 μm. Zoom panels are super-resolution confocal images. DNA is stained with DAPI (pseudo-colored red for better contrast).

I Representative confocal images of RIPosomes in THP-1 cells infected with RFP expressing *Salmonella typhimurium* (MOI 1:5, 4 h). Line profile: co-localization analysis using line intensity profile. Scale bar, 8 μm. Zoom panels are digital magnifications. DNA is stained with DAPI.

J Left panel, representative high-content microscopy images (yellow masks represent software algorithms-defined cell boundaries) of RIPosomes in THP-1 cells infected with *S. typhimurium* (MOI 1:5, 4 h). The same cell numbers and conditions are used as indicated in (B). Right panel, the graph depicts an average number of RIPosomes/cell. Mean ± SD, $n = 4$ (biological replicates), ****$P < 0.00005$, Student's unpaired *t*-test. DNA is stained with DAPI.

K–M Representative confocal images of RIPosomes in THP-1 cells infected with (K) *E. coli* LF82 strain (MOI 1: 10, 8 h), (LPS antibody is used to stain LF82) (L) mCherry expressing *M. smegmatis* (MOI 1:10, 4 h) (M) mCherry (pseudocolored to green) expressing *M. tuberculosis* (MOI 1:10, 8 h). Line profile: co-localization analysis using line intensity profiles. Scale bar, 5 μm. Zoom panels are digital magnifications. DNA is stained with DAPI.

N Western blot analysis of soluble and insoluble fractions from THP-1 cells, uninfected or infected with *E. coli* DH5α (MOI 1:10, 8 h) or *E. coli* LF82 strains (MOI 1:10, 8 h).

O Pictorial representation of data obtained in this figure shows that bacterial infection triggers oligomerization of RIPK2 or RIPosome formation that coats bacteria.

Source data are available online for this figure.

No RIPosomes were formed upon infection with GFP-tagged vesicular stomatitis virus (Appendix Fig S1J). Similarly, the NOD1 and NOD2 ligands, iE-DAP or MDP were not able to trigger visible RIPosome formation (Appendix Fig S1K). However, iE-DAP or MDP increased oligomerization of RIPK2 in the insoluble fraction of cells (Appendix Fig S1L and M), suggesting that they have a reduced capacity to induce RIPK2 oligomerization.

Altogether, these findings demonstrate that bacterial infection triggers RIPosome formation that is recruited at cytoinvasive bacteria both *in vitro* and *in vivo* conditions (Fig 1O).

RIPosomes are self-assembling structures that promote NODs oligomerization

RIPK2 is a common adaptor protein for NOD1 or NOD2-dependent NF-κB signaling. Several inflammatory proteins utilize their CARD domain to self-oligomerize and/or to hetero-oligomerize with downstream cognate signaling adaptor proteins for activation and signal transduction (Park, 2019). All three proteins, NOD1, NOD2, and RIPK2 possess CARD domain/s (Fig 2A); however, only RIPK2 self-oligomerized and formed RIPosomes (Fig 2A and B and Appendix Fig S2A) and fractionated in the insoluble (pellet) portion of cells (Appendix Fig S2B and C). Further, the CARD domain of RIPK2 ($RIPK2^{CARD}$) formed high-order oligomers (Fig 2C) and large filamentous structures (Fig 2D and Movie EV3), whereas the CARD domain of $NOD1^{CARD}$ and $NOD2^{CARDs}$ domain failed to do so (Fig 2C and D). These results suggest that only RIPK2 (but not NODs) can self-oligomerize using its CARD domain.

Interestingly, upon *Shigella* infection, endogenous NOD2 formed puncta (Appendix Fig S2D) and the oligomeric aggregates of both NOD1 and NOD2 were increased in the insoluble cell fractions (Fig 2E). NOD2 puncta were found to be juxtaposed to the bacteria (Appendix Fig S2D) where they co-localized/juxtaposed with RIPosomes (Fig 2F and Appendix Fig S2E). This apparent discrepancy between overexpressed and endogenous results could be due to the presence of endogenous RIPK2 in THP-1 cells (compared with HEK293T) whose self-assembly may have prompted co-oligomerization of NOD1/2 leading to puncta formation. Indeed, both the NOD1 and NOD2 formed insoluble oligomeric structures when co-expressed with RIPK2, as apparent from immunofluorescence (Fig 2G and Appendix Fig S2F), quantitative high-content microscopy (Appendix Fig S2G), and soluble/insoluble fractionation assays (Fig 2H). NODs were perfectly co-localized with RIPK2 in these structures (Fig 2G and Appendix Fig S2F and G). We termed these structures NODo-RIPosomes. These data were further supported by experiments where we found $NOD1^{CARD}$ or $NOD2^{CARDs}$ domain/s start oligomerizing once they are co-expressed with the $RIPK2^{CARD}$ (Fig 2I). Also, the $NOD1^{CARD}$ or $NOD2^{CARD}$ formed the punctate structures when co-expressed with $RIPK2^{CARD}$ (Appendix Fig S2H and I). Taken together, the data suggest that RIPK2 facilitates the oligomerization of NOD proteins via CARD domain/s. This notion was further tested in endogenous conditions by assessing the oligomerization of NOD proteins in the absence of RIPK2. *Shigella*-induced oligomerization of endogenous NODs was reduced in $RIPK2^{-/-}$ HT-29 cells compared with WT (Fig 2J and K). Thus, we conclude that RIPK2 self-assembling property is critical for inducing oligomerization of NOD1 and NOD2. Conversely, we noticed that the NOD proteins, in turn, enhanced the self-oligomerization capacity of RIPK2 (Appendix Fig S2J and K).

The formation of RIPosomes is important for the activation of NF-κB signaling (Gong *et al*, 2018; Pellegrini *et al*, 2018). We used a HeLa cell line expressing stable doxycycline-inducible human GFP-RIPK2 (Ellwanger *et al*, 2019) to evaluate whether *Shigella* infection-induced RIPosomes prompt nuclear translocation of NF-

 © 2022 The Authors

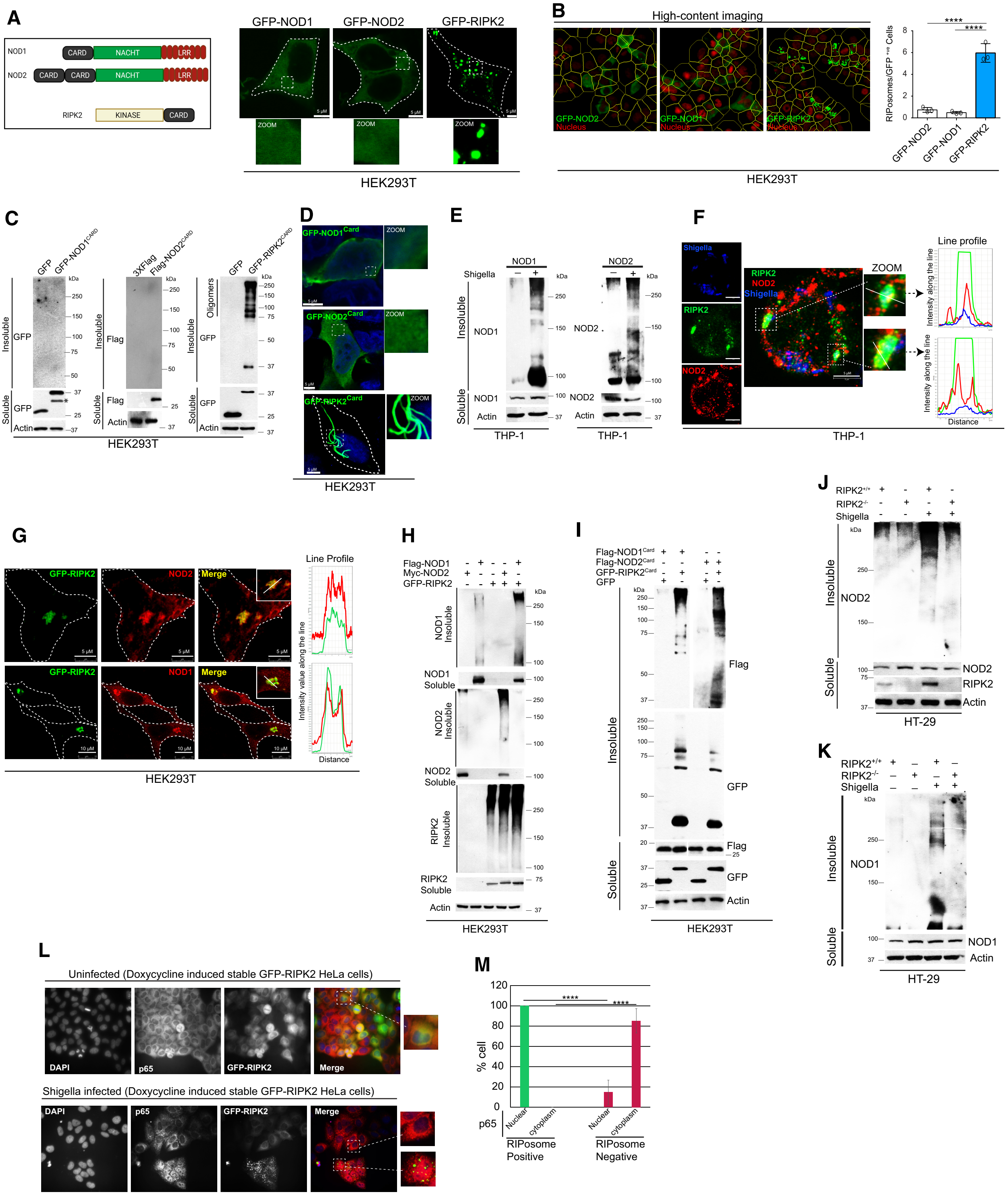
A
NOD1
CARD
NACHT
LRR
NOD2
CARD
CARD
NACHT
LRR
RIPK2
KINASE
CARD
GFP-NOD1
GFP-NOD2
GFP-RIPK2
ZOOM
HEK293T
B
High-content imaging
GFP-NOD2
Nucleus
GFP-NOD1
Nucleus
GFP-RIPK2
Nucleus
RIPosomes/GFP+ve Cells
****
****
GFP-NOD2
GFP-NOD1
GFP-RIPK2
HEK293T
C
GFP
GFP-NOD1CARD
3XFlag
Flag-NOD2CARD
GFP
GFP-RIPK2CARD
Insoluble
Soluble
Oligomers
Flag
Actin
kDa
HEK293T
D
GFP-NOD1Card
GFP-NOD2Card
GFP-RIPK2Card
ZOOM
HEK293T
E
Shigella
NOD1
NOD2
Insoluble
Soluble
Actin
THP-1
THP-1
F
Shigella
RIPK2
NOD2
ZOOM
Line profile
Intensity along the line
Distance
THP-1
G
GFP-RIPK2
NOD2
NOD1
Merge
Line Profile
Intensity value along the line
Distance
HEK293T
H
Flag-NOD1
Myc-NOD2
GFP-RIPK2
NOD1 Insoluble
NOD1 Soluble
NOD2 Insoluble
NOD2 Soluble
RIPK2 Insoluble
RIPK2 Soluble
Actin
HEK293T
I
Flag-NOD1Card
Flag-NOD2Card
GFP-RIPK2Card
GFP
Insoluble
Soluble
Flag
GFP
Actin
HEK293T
J
RIPK2+/+
RIPK2-/-
Shigella
Insoluble
Soluble
NOD2
RIPK2
Actin
HT-29
K
RIPK2+/+
RIPK2-/-
Shigella
Insoluble
Soluble
NOD1
Actin
HT-29
L
Uninfected (Doxycycline induced stable GFP-RIPK2 HeLa cells)
DAPI
p65
GFP-RIPK2
Merge
Shigella infected (Doxycycline induced stable GFP-RIPK2 HeLa cells)
M
% cell
p65
Nuclear
cytoplasm
RIPosome Positive
RIPosome Negative
****
****

Figure 2.

◀ **Figure 2. RIPK2 oligomers provide a platform for NODs oligomerization.**

A Left panel, the domain organization map of NOD1, NOD2, and RIPK2. Right panel, representative confocal images of immunofluorescence assays performed in HEK293T cells transfected with GFP-NOD1, GFP-NOD2, and GFP-RIPK2 plasmids for 9 h. Scale bar 5 μm.

B Left panel, representative high-content microscopy images of HEK293T cells transfected with GFP-NOD1, GFP-NOD2, and GFP-RIPK2 plasmids for 9 h. Right panel, the graph depicts the average number of RIPosomes per GFP-positive cell. About 15,000 cells were plated per well and RIPosomes were screened in 35 fields per well. Mean ± SD, $n = 3$ (biological replicates), ****$P < 0.00005$, ordinary one-way ANOVA (Tukey's multiple comparisons test).

C Western blot analysis of soluble and insoluble fractions of HEK293T cells transfected with the CARD domain-containing region of NOD1, NOD2, and RIPK2.

D Representative confocal images of HEK293T cells transfected with GFP-NOD1^{CARD}, GFP-NOD2^{CARD}, and GFP-RIPK2^{CARD}. Scale bar 5 μm.

E The soluble and insoluble fractions of *S. flexneri*-infected THP-1 (MOI 1:25, 8 h) cell lysates were subjected to Western blot analysis with indicated antibodies.

F Representative confocal images of immunofluorescence assays conducted with THP-1 cells infected with RFP expressing *S. flexneri* (pseudo-colored, blue) (MOI 1:25, 8 h). Line profile: co-localization analysis using line intensity profiles. Scale bar, 5 μm. Zoom panels are digital magnifications.

G Representative confocal images of immunofluorescence assays performed with HEK293T cells transfected with GFP-RIPK2 and Flag-NOD2 (upper panel) or Flag-NOD1 (lower panel). Line profile: co-localization analysis using line intensity profiles. Scale bar, 5 or 10 μm as indicated in figures. Inset zoom panels are digital magnifications.

H, I HEK293T cells were transfected with (H) full length and (I) CARD domains of NOD1, NOD2, and RIPK2 followed by Western blot analysis with soluble and insoluble fractions using indicated antibodies.

J, K Western blot analysis of soluble and insoluble fractions of RIPK2$^{+/+}$ and RIPK2$^{-/-}$ HT-29 cells infected with *S. flexneri* (MOI 1:25, 8 h) with indicated antibodies.

L, M Representative immunofluorescence images of doxycycline-inducible GFP-RIPK2 expressing HeLa cells. (L) Upper panel, uninfected. Lower panel, *S. flexneri*-infected MOI 1:25, 4 h). Immunostaining was performed with the p65 antibody (red) and DNA stained with DAPI (Blue). (M) The graph indicates % of cells that are RIPosomes positive or negative with nuclear/cytoplasmic p65 (5 fields (each group), Mean ± SD, $n = 3$). ****$P < 0.00005$, Student's unpaired t-test.

Source data are available online for this figure.

κB-p65, a signature of NF-κB activation. The p65 was not translocated into the nucleus in uninfected GFP-RIPK2 expressing cells (Fig 2L, upper panel), whereas a distinct nuclear translocation was observed in the *Shigella*-infected cells in which the RIPosomes are formed (Fig 2L, lower panel and M). However, within the *Shigella*-infected cells, the cells that are negative for RIPosomes, a majority had cytoplasmic p65 (Fig 2L, lower panel and M). Also, the nuclear translocation of phospho-p65 (Ser536) is significantly induced upon *Shigella* infection in GFP-RIPK2 overexpressing HeLa cells (Appendix Fig S2L). The data suggest that the biogenesis of RIPosome is important for the activation of NF-κB response.

To evaluate whether the NODo-RIPosomes could also induce NF-kB activation, we electroporated purified RIPosomes and NODo-RIPosomes into HEK293T cells. Both RIPosomes and NODo-RIPosomes induced NF-κB activation measured by luciferase reporter assays, where NODo-RIPosomes were consistently more efficient than RIPosomes in triggering NF-κB activation (Appendix Fig S2M).

NODs, RIPK2, and RIPosomes are the target of selective autophagy

We performed cycloheximide chase assays to determine the role of proteasome and/or autophagy processes in the turnover of endogenous NODs and RIPK2. The inhibition of autophagy flux using Bafilomycin A1 (Baf A1) completely protected RIPK2 and NOD1 from degradation, whereas proteasome inhibition using MG132 was partially protective (Fig EV1A and B). In the case of NOD2, only inhibition of autophagy protected it from degradation (Fig EV1C). These data indicate that autophagy plays a major role in the degradation of NODs and RIPK2. To confirm the role of autophagy in NODs and RIPK2 degradation, we monitored the levels of these proteins in ATG5 knockdown THP-1 or ATG5 knockout MEFs cells in uninfected and *Shigella*-infected cells. Enhanced amounts of RIPK2, NOD1, and NOD2 were detected in the ATG5-depleted cells both in control or *Shigella*-infected cells confirming that autophagy is critical in suppressing the levels of NODs and RIPK2 (Fig 3A and B).

Next, we tested whether RIPosomes are the target of autophagy. Treatment of cells with rapamycin dramatically reduced the number of RIPosomes (Fig 3C). This effect was completely rescued when the cells were additionally treated with Baf A1 (Fig 3C). Further, the numbers of RIPosome were significantly increased in the absence of ATG5 in *Shigella*-infected THP-1 cells (Fig 3D). Additionally, although RIPK2 soluble levels were induced in ATG5-depleted cells in basal conditions, RIPosomes (insoluble oligomeric aggregates) were formed only as a result of *Shigella* infection and were further induced in ATG5 KD cells (Fig 3E). Collectively, these results demonstrate that NODs and RIPK2 proteins as well as RIPosomes are the targets of autophagy.

SQSTM1/p62 mediates selective autophagy of ubiquitinated RIPosomes

Ubiquitin marks cargoes before the autophagy machinery recognizes them and degrades them (Shaid *et al*, 2013). The infection of *Shigella* induced the K63-linked ubiquitination of RIPK2 (Fig EV1D). An evident co-localization or juxtaposition of ubiquitin with RIPosomes was observed in HEK293T cells (Fig 3F) and *Shigella*-infected THP-1 cells (Fig EV1E). To understand whether ubiquitination of RIPosomes is important for their autophagic degradation, we inhibited ubiquitination in cells using PYR-41, which is a selective and cell-permeable inhibitor of ubiquitin-activating enzyme E1. In high-content microscopy, we found that rapamycin-induced autophagic degradation of RIPosomes is blocked by PYR-41 (Fig EV1F), indicating that ubiquitinated RIPosomes are the target of autophagy.

Autophagy receptor proteins are critical in bridging the ubiquitinated cargoes to the autophagosomes (Shaid *et al*, 2013). To identify the receptor that recognizes RIPK2, we screened the interaction between RIPK2 and key autophagy receptors, including p62, NBR1, NDP52, TAX1BP1, and Optineurin (Fig 3G). A strong physical interaction was observed between RIPK2 and p62 (Fig 3G). NBR1 was faintly bound and other receptors completely failed to interact with RIPK2 (Fig 3G). Thus, we tested whether p62 mediates autophagic degradation of RIPK2 and RIPosomes. Levels of both soluble and insoluble forms of RIPK2 were increased in *Shigella*-infected THP-1 cells upon p62 knockdown (Fig 3H). Also, we found an increased number of RIPosomes in p62 knockdown cells compared with

 © 2022 The Authors

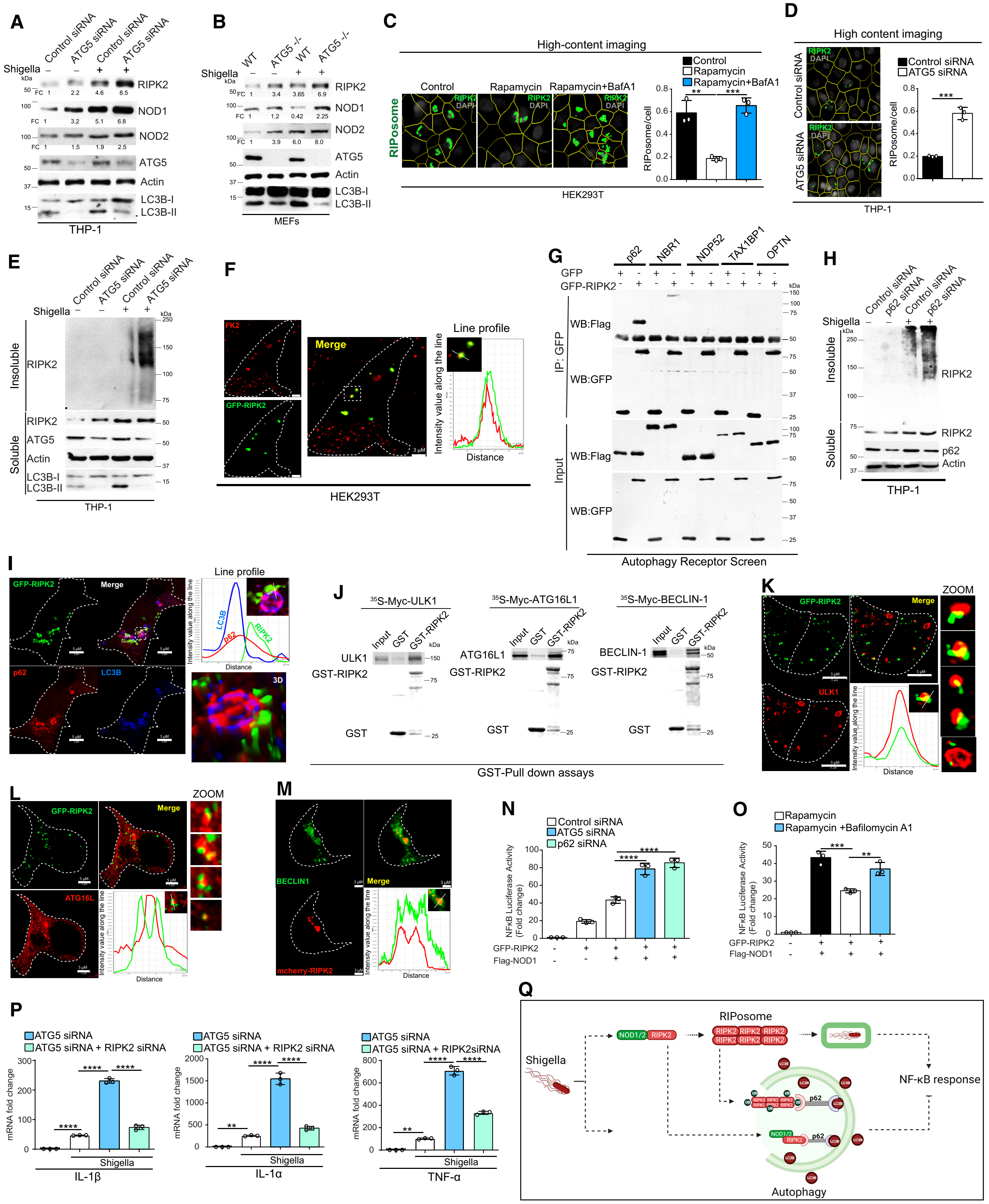

Figure 3.

Figure 3. NODs, RIPK2, and RIPosomes are degraded by p62-dependent selective autophagy.

A, B Western blot analysis with cell lysate of uninfected and *S. flexneri*-infected (MOI 1:25, 8 h) (A) control and ATG5 siRNA knockdown THP-1 cells, (B) wild-type (WT) and ATG5 knockout ($ATG5^{-/-}$) MEF cells with indicated antibodies. Densitometric analysis was performed using Image J software. FC, fold change.

C Left panel, representative high-content microscopy images of RIPosomes in HEK293T cells that are control cells or cells treated with rapamycin (500 nM, 4 h) or cells treated with rapamycin (500 nM, 4 h) and bafilomycin A1 (BafA1, 300 nM). Right panel, the graph depicts the average number of RIPosomes/cell. About 15,000 cells were plated per well and RIPosomes were screened in 35 fields per well. Mean ± SD, $n = 3$ (biological replicates), $^{**}P < 0.005$ and, $^{***}P < 0.0005$, ordinary one-way ANOVA (Tukey's multiple comparisons test).

D Left panel, representative high-content microscopy images of RIPosomes in control and ATG5 siRNA transfected THP-1 cells infected with *S. flexneri* (8 h). Right panel, the graph depicts average number of RIPosomes/cell. About 50,000 cells were plated per well and RIPosomes were screened in 35 fields per well. Mean ± SD, $n = 3$ (biological replicates), $^{***}P < 0.0005$, Student's unpaired *t*-test.

E The soluble and insoluble fractions of *S. flexneri*-infected (MOI 1:25, 8 h) control and ATG5 siRNA knockdown THP-1 cells were subjected to Western blot analysis with indicated antibodies.

F Representative confocal images of HEK293T cells transfected with GFP-RIPK2 and immunostained with anti-FK2 antibodies. Line profile: co-localization analysis using line intensity profiles. Scale bar, 3 μm.

G Autophagy receptor screen using co-immunoprecipitation (Co-IP) assay to analyze the interaction between GFP-RIPK2 and Flag-p62 or Flag-NBRI or Flag-NDP52 or Flag-TAX1BP1 or Flag-OPTINEURIN in HEK293T cell lysate.

H Western blot analysis of soluble and insoluble fractions of control and p62 siRNA transfected and *S. flexneri*-infected THP-1 cells (MOI 1:25, 8 h).

I Representative confocal images of HEK293T cells expressing GFP-RIPK2 (6 h) immunostained with p62 and LC3B antibodies. Zoom panels are digital magnifications.

J GST pull-down assay using purified GST or GST-RIPK2 proteins with in vitro translated ^{35}S radiolabeled myc-ULK1 or myc-ATG16L or myc-BECLIN1.

K–M Representative confocal images of HEK293T cells transfected with GFP-RIPK2 or mcherry-RIPK2 (9 h) and immunofluorescence assay performed with antibodies specific to (K) ULK1, (L) ATG16L (M) BECLIN1. Line profile: co-localization analysis using line intensity profiles. Scale bar, 5 or 3 μm as indicated. Zoom panels are digital magnifications.

N Luciferase assays were performed with ATG5 or p62 knockdown HEK293T cells transfected with NF-κB luciferase reporter vector pGL4.32 NFκB-RE, GFP-RIPK2, and Flag-NOD1 plasmids. Mean ± SD, $n = 3$ (biological replicates), $^{****}P < 0.00005$, ordinary one-way ANOVA (Tukey's multiple comparisons test).

O Luciferase assays performed with HEK293T cells transfected with NF-κB luciferase reporter vector pGL4.32NFκB-RE, GFP-RIPK2, and Flag-NOD1 plasmids followed by treatment with rapamycin (500 nM, 4 h) alone or in combination with bafilomycin A1 (300 nM, 5 h). Mean ± SD, $n = 3$ (biological replicates), $^{**}P < 0.005$ and $^{***}P < 0.0005$, ordinary one-way ANOVA (Tukey's multiple comparisons test). The control conditions and readings for Fig 3N and P are the same.

P The qRT–PCR analysis with total RNA isolated from the uninfected and *S. flexneri*-infected (MOI 1:25, 6 h) control or ATG5 knockdown or ATG5 and RIPK2 double knockdown THP-1 cells. Mean ± SD, $n = 3$ (biological replicates), $^{**}P < 0.005$, and $^{****}P < 0.00005$, ordinary one-way ANOVA (Tukey's multiple comparisons test).

Q Pictorial representation of results obtained in this section where we found that p62-dependent selective autophagy degrades NODs, RIPK2, and RIPosomes to suppress NF-κB cytokine response.

Source data are available online for this figure.

control cells (Fig EV1G), suggesting that p62 plays a critical role in the autophagic degradation of RIPosomes. In agreement, p62 and LC3B (autophagosomes) were either co-localized or juxtaposed to RIPosomes (Figs 3I and EV1H). The high-resolution microscopy and 3D rendering of images indicate that p62 tethered the RIPosome to LC3B decorated autophagosomes (Fig 3I). Also, LC3B was found to be co-localized with RIPosome recruited over the *Shigella* (Fig EV1I).

Next, we asked whether other key autophagosome initiations (ULK1) and elongation proteins (ATG16L1 and BECLIN1) interact and co-localize with RIPK2/RIPosomes. We found that *in vitro* translated ULK1, BECLIN1, and ATG16L1 directly interacted with purified GST-RIPK2 in GST pull-down assays (Fig 3J). Further, all the three important autophagy proteins were either completely co-localized or juxtaposed to the RIPosomes (Figs 3K–M and EV1J and K) indicating a *de novo* biogenesis of autophagosomes occurring adjacent to the RIPosomes for their degradation. When cargo is degraded via autophagy, typically autophagy receptors specifically p62 also subjected to degradation. We observed that p62 is degraded upon *Shigella* infection and this degradation is rescued upon Bafilomycin A1 treatment, suggesting that p62 is targeted by autophagy along with cargo upon *Shigella* infection (Fig EV1L).

The depletion of ATG5 and p62 significantly increased NOD1/RIPK2-dependent NF-κB activity in luciferase reporter assays (Fig 3N). Conversely, autophagy activation by rapamycin reduced the NOD1/RIPK2-dependent NF-κB activity (Fig 3O) that was rescued by Baf A1 treatment (Fig 3O). Finally, the depletion of ATG5 *or p62* significantly enhanced *Shigella*-induced NF-κB-mediated pro-inflammatory cytokine response (TNFα, IL-1β, and IL-1α) in THP-1 cells (Figs 3P and EV1M). This enhanced cytokine response was rescued by RIPK2 silencing (Figs 3P and EV1M), suggesting that autophagy suppresses RIPK2-dependent NF-κB pro-inflammatory cytokine response.

Altogether, we found that p62-dependent selective autophagy mediates the degradation of NODs, RIPK2, and RIPosomes to suppress NF-κB activation and pro-inflammatory cytokine response (Fig 3Q).

Autophagy protein, IRGM is recruited over the bacteria and interacts with RIPosomes

IRGM is an autophagy protein that plays a critical role in the selective autophagic degradation of pro-inflammatory proteins (Mehto *et al*, 2019a, 2019b; Jena *et al*, 2020; Chauhan *et al*, 2021). We set out to examine the role of IRGM in the autophagic degradation of NODs, RIPK2, and RIPosomes. For that, first, we tested whether IRGM interacts with NODs and RIPK2. In the immunoprecipitation (IP) assays, endogenous IRGM interacted with RIPK2 and NODs (Fig 4A and B). The interaction of IRGM with NOD1/RIPK2 and NOD2/RIPK2 complex is further increased when the cells were treated with iE-DAP (NOD1 agonist) or MDP (NOD2 agonist), respectively (Fig 4A and B). In IP assays with the HT-29 cell line stably expressing Flag-IRGM, IRGM immunoprecipitated endogenous NOD1, NOD2, and RIPK2 (Appendix Fig S3A). A strong interaction between overexpressed IRGM with NOD1, NOD2, and RIPK2 was also observed in HEK293T cells (Fig 4C and D, and Appendix Fig S3B). Furthermore, a direct interaction of purified

 © 2022 The Authors

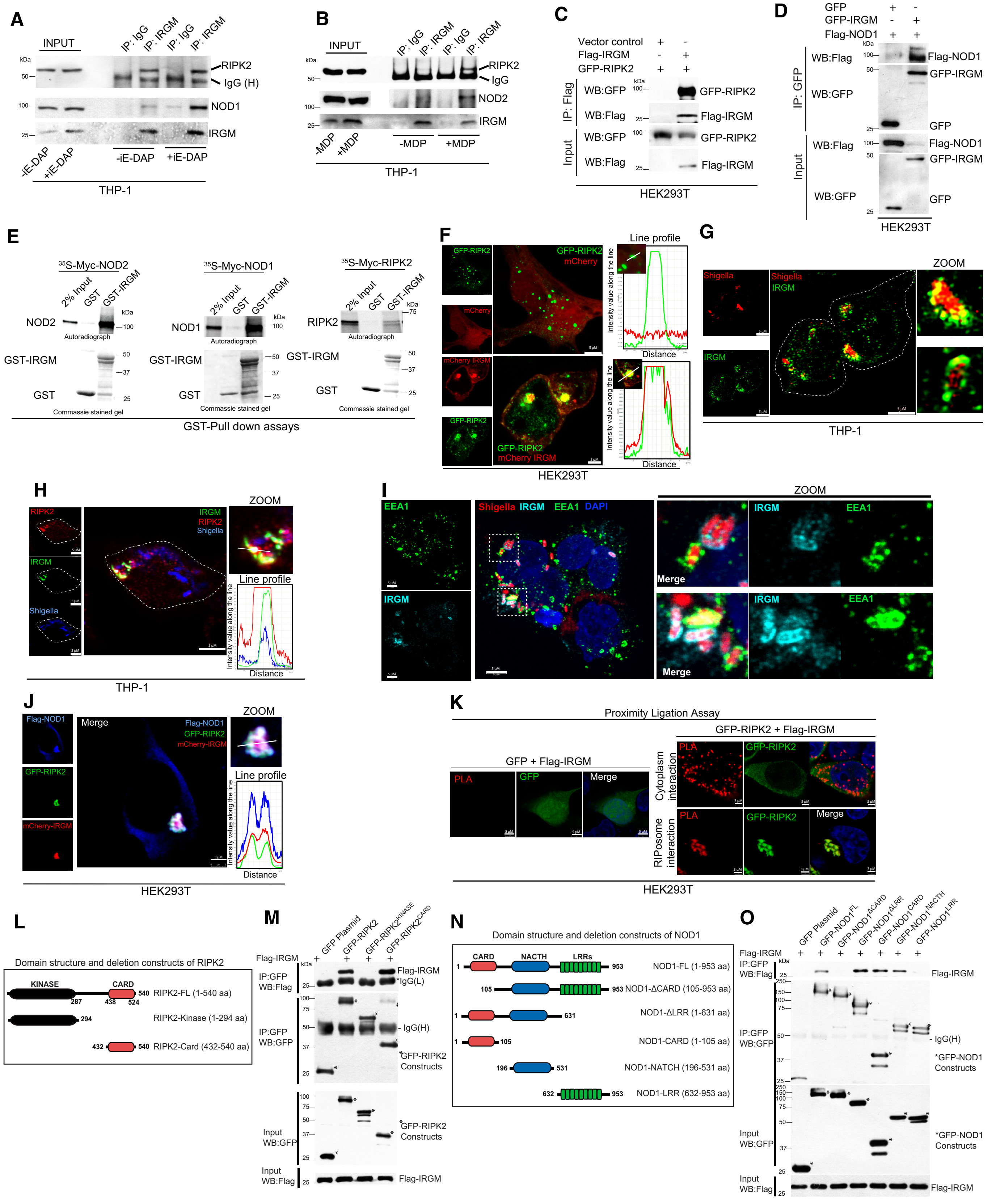

Figure 4.

Figure 4. Autophagy protein IRGM interacts and co-localizes with NODs, RIPK2, and RIPosomes.

A, B The THP-1 cell lysates were subjected to immunoprecipitation analysis (A) untreated and treated with iE-DAP (40 µg/ml, 6 h), (B) untreated and treated with MDP (40 µg/ml, 6 h). IP was performed with isotype control IgG or IRGM antibody and Western blotting was performed with indicated antibodies. IgG (H), IgG heavy chain.

C, D Co-IP analysis of the interactions between (C) GFP-RIPK2 and Flag-IRGM or (D) Flag-NOD1 and GFP-IRGM in HEK293T cell lysates.

E GST pull-down assay using purified GST and GST-IRGM and *in vitro* translated ^{35}S radiolabeled myc-NOD2, myc-NOD1, and myc-RIPK2.

F Representative confocal images of HEK293T cells expressing GFP-RIPK2 and mCherry or mCherry-IRGM. Line profile: co-localization analysis using line intensity profiles. Scale bar, 5 µm.

G, H Representative confocal images of THP-1 cells infected with RFP expressing *S. flexneri*, (MOI 1:25, 8 h) and immunostained with, (G) IRGM antibody (H) IRGM and RIPK2 antibody. Line profile: co-localization analysis using line intensity profiles. Scale bar, 5 µm. Zoom panels are digital magnifications. In image (H) for better contrast, RFP expressing *Shigella* is pseudo-colored to blue.

I Representative confocal images of THP-1 cells infected with RFP expressing *S. flexneri*, (MOI 1:25, 20 min) and immunostained with IRGM and EEA1 antibodies. DNA stained with DAPI. Scale bar, 5 µm. Zoom panels are digital magnifications.

J Representative confocal images of HEK293T cells expressing GFP-RIPK2, Flag-NOD1, and mCherry-IRGM. Line profile: co-localization analysis using line intensity profiles. Scale bar, 3 µm. Zoom panels are digital magnifications.

K Representative confocal images of proximity ligation assay (PLA) in HEK293T transfected with GFP or GFP-RIPK2 and Flag-IRGM plasmid. Scale bar 3 or 5 µm as indicated.

L The domain organization map of RIPK2 and deletion construct cloned as GFP-tagged proteins.

M A co-IP analysis is performed with HEK293T cell lysates expressing various domains of RIPK2 and IRGM to map the domain/s of RIPK2 interacting with IRGM. Asterisk indicates the main band of overexpressed protein.

N The domain organization map of NOD1 and deletion construct cloned as GFP-tagged proteins.

O A co-IP analysis is performed with HEK293T cell lysates expressing various domains of NOD1 and IRGM to map the domain/s of NOD1 interacting with IRGM. Asterisk indicates the main band of overexpressed protein.

Source data are available online for this figure.

GST-IRGM with NODs and RIPK2 was observed in GST pull-down assays (Fig 4E).

IRGM does not form oligomeric structures alone in the cells (Appendix Fig S3C). However, when expressed together with RIPK2, IRGM formed structures that were fully co-localized or juxtaposed to RIPosomes (Fig 4F and Appendix Fig S3D). Analysis of data from quantitative high-content microscopy displayed a high level of co-localization between IRGM and RIPK2 (Appendix Fig S3E). Interestingly, in *Shigella*-infected cells, IRGM was recruited to the intracellular bacteria (Fig 4G and Movie EV4) together with RIPosomes (Fig 4H). In some cells, we observed the formation of cage-like structures of RIPK2 and IRGM surrounding the bacteria (Appendix Fig S3F). *Shigella* is a predominantly cytosolic bacterium that ruptures its bacteria-containing vesicle very rapidly (within 10–15 min) after the invasion (Ray *et al*, 2010; Lopez-Montero & Enninga, 2018). Also, *Shigella* tends to avoid the recruitment of Rab GTPases and other maturation proteins (including LAMP proteins) using several mechanisms (Ray *et al*, 2010; Lopez-Montero & Enninga, 2018). We infected THP-1 cells with *Shigella* for 20 min and performed immunofluorescence with EEA1 (early phagosome/endosome marker) or LAMP2A (late phagosome maturation marker) and IRGM to understand whether IRGM is recruited to phagosomal or cytosolic *Shigella*. We found that very few bacteria were positive for EEA1 protein (Fig 4I) and did not observe LAMP2A recruitment over the *Shigella* (Appendix Fig S3G) confirming the previous observations. EEA1-marked phagosomes were rarely localized with IRGM, whereas IRGM was recruited to several of the bacteria. The data suggest that IRGM is recruited to *Shigella* once they escape phagosomes and are in the cytosolic compartment. Also, IRGM was found to be recruited on NODo-RIPosome complexes (Fig 4J and Appendix Fig S3H). IRGM and RIPK2 interaction was confirmed by proximity ligation assay (PLA), which reports direct protein–protein interactions (Fig 4K). Direct interaction between IRGM and RIPK2 was observed in the cytosol as well as over the RIPosomes (Fig 4K).

NOD2 interacts with IRGM primarily via CARD domain (Chauhan *et al*, 2015). We mapped the domain by which RIPK2 or NOD1 interact with IRGM. RIPK2 has one kinase and one CARD domain (Fig 4L), but only the CARD domain interacted with IRGM (Fig 4M). NOD1 consists of a CARD, a NACHT, and several LRR domains (Fig 4N). The CARD and NACTH domains are utilized by NOD1 to interact with IRGM (Fig 4O, lanes 5 and 6). No interaction was detected with the LRR domain (Fig 4O, lane 7). Consistently, deleting LRR domains did not affect the NOD1-IRGM interaction (or rather increased interaction; Fig 4O, lane 4); however, removing the CARD domain abolished NOD1-IRGM interaction (Fig 4O, lane 3), suggesting that the CARD domain may provide a primary interface for the interaction (Fig 4O, lane 3). Thus, the findings suggest that the CARD domain of NODs and RIPK2 provides a primary interface for interaction with IRGM.

In summary, IRGM strongly and specifically interacts with NODs and RIPK2. We show that IRGM is recruited to the cytosolic *Shigella* bacteria where it co-localizes with RIPosomes. Along with IRGM, other autophagy proteins including ULK1 and p62 were recruited to the *Shigella* bacteria (Appendix Fig S3I and J). ULK1 which is a known interaction partner of IRGM (Chauhan *et al*, 2015) was found to be completely covering the bacteria along with IRGM (Appendix Fig S3J).

IRGM mediates NODs, RIPK2, and RIPosome degradation to suppress NF-κB response

Next, we investigated how IRGM interaction with NODs, RIPK2, and RIPosomes modulates their functions. An increased protein level of RIPK2 and NODs was observed in IRGM CRISPR (partial) knockout HT-29 (IRGM$^{+/-}$) cells compared with the control cells (Fig 5A). The IRGM$^{+/-}$ HT29 cells are described previously (Jena *et al*, 2020). Also, *Shigella*-induced expression of NODs and RIPK2 was further enhanced in IRGM$^{+/-}$ HT-29 cells (Fig EV2A). Increased protein levels of NODs and RIPK2 were detected in colons and BMDMs of *Irgm1* knockout

 © 2022 The Authors

(*Irgm1*$^{-/-}$) mice (Fig 5B). An increased amount of insoluble oligomeric RIPK2 was observed in Irgm1$^{-/-}$ BMDMs lysates as compared to the control cells (Fig 5B). Next, we used a HeLa cell line expressing stable doxycycline-inducible human GFP-RIPK2 (Ellwanger *et al*, 2019). Silencing IRGM in the *Shigella*-infected cells enhanced the levels of NODs and RIPK2 (Fig EV2B), and a significantly increased number of RIPosomes was observed (Fig 5C). Conversely, transient and stable overexpression of IRGM in HT-29, THP-1, and HEK293T cells resulted in degradation of endogenous and overexpressed NODs and RIPK2 (Figs 5D and E, and EV2C–E). Overexpression of IRGM significantly reduced the RIPosomes and NODo-RIPosomes formed in the cells (Figs 5F and EV2F). Also, the expression of IRGM reduced the insoluble oligomeric forms of RIPK2 and NOD2/RIPK2 (Figs 5G and EV2G). IRGM was found to be co-localized with RIPK2^{CARD} and was able to degrade the RIPK2^{CARD} filamentous structure into small punctate assemblies (Figs 5H, and EV2H and I). Taken together, the data show that IRGM mediates the degradation of NODs, RIPK2, and RIPosomes.

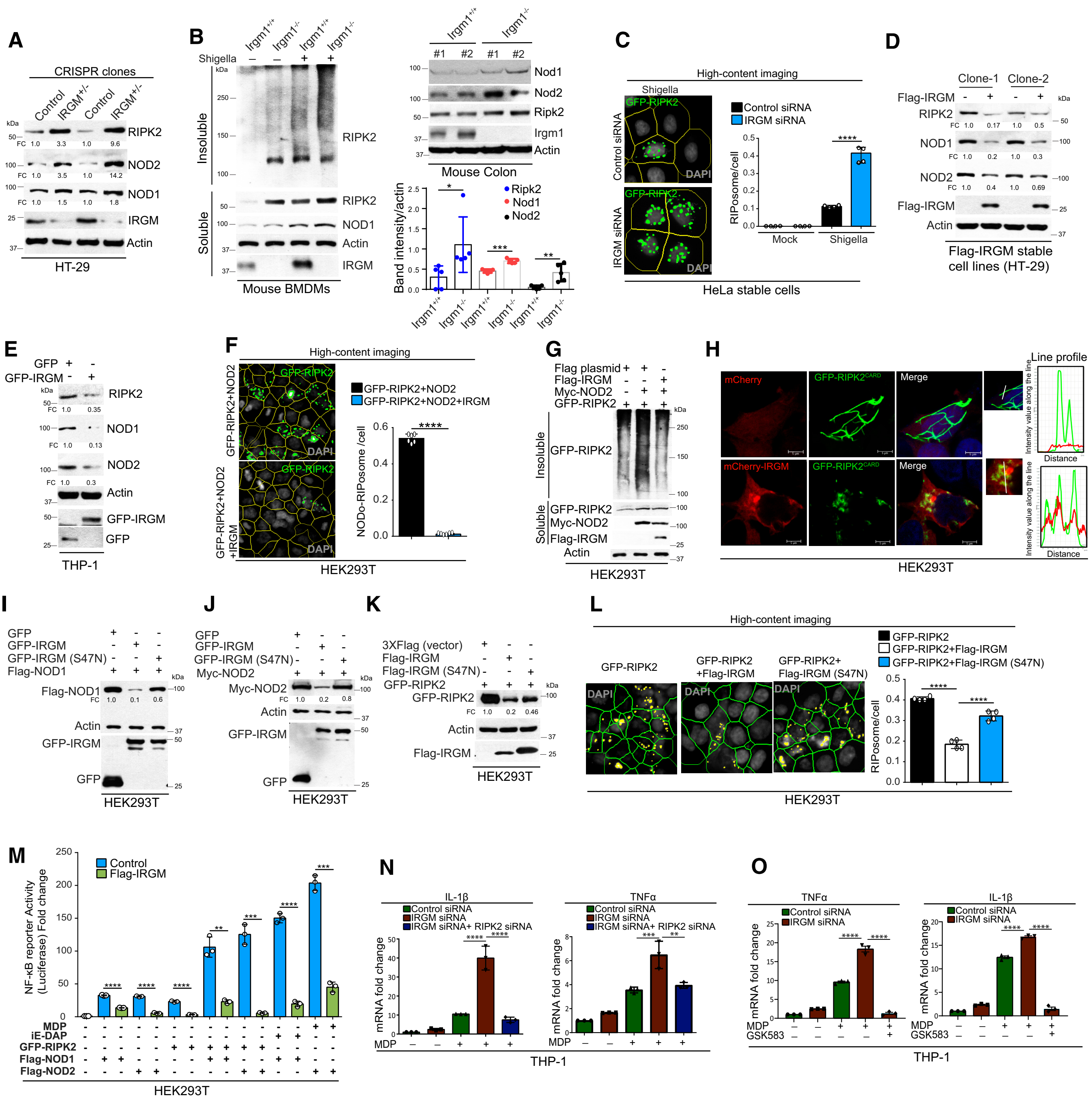

Figure 5.

Figure 5. IRGM mediates the degradation of NODs, RIPK2, and RIPosomes to suppress NF-κB-dependent cytokine response.

A Western blot analysis with the cell lysates of control CRISPR cells and CRISPR-Cas9 mediated IRGM partial knockout (IRGM$^{+/-}$) HT-29 cells (2 clones were tested). Densitometric analysis was performed using Image J software. FC, fold change.

B Left panel, soluble and insoluble fractions of *S. flexneri* (MOI 1:25, 8 h) infected mouse BMDMs from *Irgm1*$^{+/+}$ and *Irgm1*$^{-/-}$ mice were subjected to immunoblot analysis with antibodies as indicated. Right panel, Western blot analysis with the colon lysates from *Irgm1*$^{+/+}$ and *Irgm1*$^{-/-}$ mice with indicated antibodies. The graph indicate ratio of band intensity (measured using ImageJ) and actin ($n = 5$, Mean ± SD, $*P < 0.05$, $**P < 0.005$, and $****P < 0.00005$, Student's unpaired *t*-test).

C Doxycycline-inducible stable GFP-RIPK2 HeLa cells were transfected with control siRNA or IRGM siRNA followed by infection with *S. flexneri* (MOI 1:25, 4 h). The cells were fixed and subjected to high-content microscopy to quantitate the number of RIPosomes formed. The graph depicts an average number of RIPosome/cell. About 10,000 cells were plated per well and RIPosomes were screened in 35 fields per well. Mean ± SD, $n = 4$ (biological replicates), $****P < 0.00005$, Student's unpaired *t*-test.

D, E Western blot analysis with the cell lysate of (D) HT-29 clones stably expressing Flag-vector control or Flag-IRGM (E) THP-1 cells transiently transfected with GFP or GFP-IRGM for 6 h. Densitometric analysis was performed using Image J software. FC, fold change.

F Left panel, representative high-content microscopy images of NODo-RIPosomes in HEK293T cells transfected with GFP-RIPK2 and myc-NOD2 (upper panel) or GFP-RIPK2, myc-NOD2, and Flag-IRGM (lower panel). Right panel, the graph depicts average number of NODo-RIPosome/cell. About 15,000 cells plated per well and RIPosomes were screened in 35 fields per well. Mean ± SD, $n = 5$ (biological replicates), $****P < 0.00005$, Student's unpaired *t*-test.

G The soluble and insoluble fractions of HEK293T cells transfected with indicated plasmids were subjected to immunoblot analysis with indicated antibodies.

H Representative confocal images of HEK293T cells transfected with mCherry and GFP-RIPK2^{CARD} or mCherry-IRGM and GFP-RIPK2^{CARD} for 9 h. Line profile: co-localization analysis using line intensity profiles. Scale bar, 5 μm.

I–K Western blot analysis with the cell lysates of HEK293T transfected with indicated plasmids and probe with Actin, Flag, Myc, and, GFP antibodies as indicated. Densitometric analysis was performed using Image J software. FC, fold change.

L Left panels, representative high-content microscopy images of HEK293T cells transfected with GFP-RIPK2 or GFP-RIPK2 and Flag-IRGM or GFP-RIPK2 and Flag-IRGM (S47N). The graph depicts the average number of RIPosome/cell. The details are mentioned in the legends of Fig 1F. Mean ± SD, $n = 4$ (biological replicates), $****P < 0.00005$, ordinary one-way ANOVA (Tukey's multiple comparisons test).

M Luciferase assays performed with HEK293T cells transfected with NF-κB luciferase reporter vector pGL4.32NFκB-RE, along with plasmids as indicated, followed by treatment with MDP (10 μg/ml, 4 h) or iE-DAP (10 μg/ml, 4 h) as indicated. Mean ± SD, $n = 3$ (biological replicates), $**P < 0.005$, $***P < 0.0005$ and $****P < 0.00005$, Student's unpaired *t*-test.

N, O The qRT–PCR analysis with total RNA isolated from THP1 cells transfected with indicated siRNA and treated with (N) L-18 MDP (1 μg/ml, 6 h) for indicated genes (O) L-18 MDP (1 μg/ml, 6 h) and GSK583 (1 μM). Mean ± SD, $n = 3$ (biological replicates), $*P < 0.05$, $**P < 0.005$, $***P < 0.0005$ and, $****P < 0.00005$, ordinary one-way ANOVA (Tukey's multiple comparisons test).

Source data are available online for this figure.

A point mutation in the GTPase domain (Serine to Glycine at 47th position, S47N) of IRGM renders it inactive to perform autophagy functions (Chauhan *et al*, 2015; Kumar *et al*, 2018; Mehto *et al*, 2019a; Jena *et al*, 2020). As compared to wild-type IRGM, the catalytic mutant (S47N) of IRGM was unable to mediate the degradation of NOD1 (Fig 5I), NOD2 (Fig 5J), RIPK2 (Fig 5K), RIPK2^{CARD} (Fig EV2J), and their oligomers (Figs 5L and EV2K). These results demonstrate the specificity of IRGM-mediated effects and also indicate the role of GTPase-dependent autophagy activity of IRGM in the degradation of NODs, RIPK2, and their oligomers.

Next, we examined the effect of IRGM on NODs/RIPK2-dependent NF-κB activation. First, we chased the phosphorylation (Ser536) status of p65 (p-p65) in the presence and absence of IRGM. The p-p65 is induced upon *Shigella* infection that is further increased upon IRGM knockdown (Appendix Fig S4A). The data suggest that *Shigella*-induced IRGM expression suppresses the NF-κB activation. In luciferase NF-κB reporter assays, IRGM strongly suppressed basal and NODs agonists (MDP and iE-DAP) induced NF-κB promoter activity (Fig 5M). We also employed an MDP-inducible NOD2 expressing secreted alkaline phosphatase (SEAP)-based NF-κB reporter cell line (Invivogen) to assess the effect of IRGM on NF-κB response. The overexpression of IRGM diminished MDP-induced NOD2 and NOD2-RIPK2 dependent NF-κB reporter activity (Fig EV2L). By contrast, silencing IRGM in MDP-treated THP-1 cells resulted in increased mRNA expression of IL-1β and TNFα (Fig 5N), which was restored in the RIPK2-depleted cells (Fig 5N). Similarly, GSK583, a specific and potent inhibitor of RIPK2 (Haile *et al*, 2016), suppressed the cytokine response increased upon IRGM knockdown (Fig 5O). Taken together, our results show that IRGM facilitates the degradation of NODs, RIPK2, and their oligomers to suppress NF-κB activity and cytokine response.

Endogenous IRGM levels in cells were increased upon exposure to *Shigella*, (Figs 5B, and EV2A and B; compare lanes 1 and 3), MDP (Appendix Fig S4B and D), and iE-DAP (Appendix Fig S4C and E). Thus, microbes and NODs agonists induce expression of IRGM that by a negative feedback loop mediates degradation of NODs-RIPK2 signaling proteins to suppress NF-κB response to maintain cell-autonomous innate immune homeostasis.

IRGM and p62 cooperate to conduct selective autophagy of NODs and RIPK2/RIPosomes

We scrutinized whether IRGM-dependent degradation of NODs and RIPK2 is mediated through proteasome or autophagy. Inhibition of autophagy flux (using Baf A1) but not proteasome (using MG132) restored the IRGM-dependent degradation of NODs and RIPK2 (Appendix Fig S5A–C). Further, the depletion of ATG5 rescued the IRGM-mediated degradation of endogenous (THP-1 cells; Fig EV3A and B) or overexpressed (HEK293T cells) NODs and RIPK2 (Fig EV3C–E). Also, the IRGM-dependent RIPosome degradation is restored when the cells were either depleted of ATG5 or were treated with Baf A1 (Fig EV3F). This and the above-discussed catalytic mutant (IRGM^{S47N}) data demonstrate that IRGM utilizes autophagy to degrade NODs, RIPK2, and RIPosomes. Further, PYR-41 (inhibitor of ubiquitin-activating enzyme E1) rescued the IRGM-mediated degradation of RIPK2, indicating that ubiquitinated RIPK2 is targeted by IRGM-dependent autophagy (Appendix Fig S5D).

 © 2022 The Authors

Like RIPK2, IRGM interacts only with p62 among the autophagy receptor proteins (Jena *et al*, 2020). Therefore, we tested whether IRGM cooperates with p62 for the autophagic degradation of RIPK2 and RIPosomes. First, we investigated whether IRGM, p62, and RIPK2 are present in the same molecular complex. IRGM and p62 were co-localized or juxtaposed to the RIPosomes (also NODo-RIPosomes; Fig EV3G and Appendix Fig S5E and F). Also, co-localization/juxtaposition of RIPosomes, IRGM, and p62 with LC3B was observed (Appendix Fig S5G). Using sequential IP assay, we found that RIPK2, p62, and IRGM were present in the same complex (Fig EV3H). Silencing of p62 rescued the IRGM-dependent autophagic degradation of RIPosomes (Fig EV3I), and also IRGM-mediated degradation of overexpressed as well as endogenous RIPK2, NOD1, and NOD2 (Fig EV3J–M). Thus, IRGM utilizes p62 adaptor protein to mediate autophagic degradation of NODs, RIPK2, and RIPosomes. Next, we tested whether IRGM is required for the interaction between p62 and RIPK2. Indeed, the depletion of IRGM reduced the interaction between p62 and RIPK2 (Fig EV3N). Conversely, the presence of IRGM increased the interaction of p62 with NODs and RIPK2 (Fig EV3O–Q).

Taken together, the results show that IRGM and p62 cooperate to conduct inflammophagy of NODs, RIPK2, and RIPosome complexes. In addition, we observed that autophagy initiation and elongation protein ULK1 and ATG16L1 were co-localized with IRGM over the RIPosomes (Appendix Fig S5H and I), indicating that IRGM engages canonical autophagy machinery for degradation.

Combined results from this and the previous sections suggest microbes induce RIPosome formation and IRGM expression. Both IRGMs and RIPosomes are recruited over bacteria. Where RIPosome formation induces NF-κB response, IRGM-dependent autophagic degradation of NODs-RIPK2 complex suppresses the NF-κB response to balance the inflammatory outputs (Fig EV3R).

IRGM negatively regulates bacteria-induced RIPK2-dependent pro-inflammatory signaling pathways

To understand the role of IRGM in regulating host response to bacterial infection, we performed RNA-sequencing (RNA-seq) experiment with *Salmonella typhimurium* infected control and stable IRGM shRNA knockdown HT-29 colon cells. Hierarchical clustering based on gene ontology (GO) terms was performed using genes differentially regulated ($P < 0.05$, 1.5-fold) in basal and *Salmonella*-infected IRGM-depleted cells compared with controls. Several inflammatory (e.g., Interferon signaling and cytokine signaling) and infection-related processes (e.g. ER-phagosome and antigen processing/presentation) were among the top-enriched pathways (Reactome pathway analysis) induced in IRGM-depleted cells, which were further increased upon *Salmonella* infection (Datasets EV1 and EV2, and Fig EV4A). In basal conditions, IRGM suppresses a large number of IFN-stimulated genes (ISGs; Jena *et al*, 2020). Here, we found that during *Salmonella* infection, in addition to ISGs, IRGM suppressed a large number of chemokines (*CXCL1*, *2*, *3*, *5*, *6*, *8*, *10* and *CCL20*, *22*, *28*, etc.) interleukins (*IL1A*, *IL1B*, *IL1E/36G*, *IL17C*, *IL32*, *IL15*, etc.) and TNF superfamily genes (*TNFSF9*, *TNFSF10*, *TNFSF13*, *TNFSF15*, etc.; Dataset EV3, and Fig EV4B and C). Several other pathways such as endoplasmic reticulum-phagosome response, endosomal/vacuolar pathways, and antigen processing and presentation were upregulated in *Salmonella*-infected IRGM-depleted cells (Fig EV4A and B). The RNA-seq results were validated by performing qRT–PCR with several chemokines and interleukins (Fig EV4D). Next, to define the IRGM-dependent transcriptome that is exclusively upregulated upon *Salmonella* infection, we filtered out all the basal level deferentially upregulated genes and performed Reactome pathway analysis (Fabregat *et al*, 2018) with the rest of the transcriptome (Dataset EV4). In this analysis, the top-enriched pathways were TNFα signaling, IL-17 signaling, NF-κB signaling, interleukin-dependent signaling, interferon-gamma response, and NOD-like receptor signaling pathway (Fig EV4E). These data indicate that during bacterial infection IRGM limits an extensive and comprehensive program of pro-inflammatory response including NF-κB, TNFα, NODs, and IFN signaling pathways.

Our next step was to evaluate the extent to which IRGM-mediated suppression of the inflammatory response was dependent upon NOD1/2-RIPK2 signaling. For that, we performed RNA-seq analysis with *Shigella flexineri* infected WT, IRGM$^{+/-}$, and RIPK2-depleted IRGM$^{+/-}$ HT-29 cells. To better understand whether the suppression of the pro-inflammatory response by IRGM is specific to *Salmonella* or applies globally, we performed transcriptome analysis on *Shigella*-infected cells. A comparison of the analysis of the transcriptome induced in *Shigella* versus *Salmonella*-infected IRGM-depleted cells suggests that almost similar genes and pathways were upregulated in both the conditions (Fig EV4B vs. F, Dataset EV5), suggesting that IRGM has identical anti-inflammatory functions during different bacterial infections. Next, we performed the analysis with a set of genes that are significantly upregulated in IRGM$^{+/-}$ HT-29 cells (compared with control, $P < 0.05$, 1.5-fold, $n = 3$) and at the same time were significantly rescued by RIPK2 depletion ($P < 0.05$, 0.8, $n = 3$; Dataset EV6). A large number of genes (~250 genes) that were induced upon IRGM depletion were rescued by RIPK2 knockdown, suggesting that IRGM regulates pro-inflammatory responses through modulation of RIPK2 protein levels (Fig 6A and Dataset EV6). Metascape pathway analysis (Tripathi *et al*, 2015) with this gene set revealed that upon *Shigella* infection, IRGM suppresses several inflammatory responses including IFN response, NF-κB signaling, and interleukin signaling in a RIPK2-dependent manner (Fig 6B). Several of the NF-κB-regulated genes such us *NINJ1*, *MAPK11*, and cytokines such as CXCLs (*CXCL1*, *3*, *6*, *10*, *and 11*), Interleukins (*IL1α and IL1β*), tumor necrosis factors, and receptors (*TNF-α*, *TNSFAIP's*, *and TNSF's*), that were induced upon IRGM$^{+/-}$ were rescued by additional siRNA knockdown of RIPK2 (Fig 6C and D). Interestingly, during bacterial infection IRGM limited the interferon response in a RIPK2-dependent manner (Fig 6E and F). This was evident by the partial rescue of several sentinels' interferon-responsive genes including *IFITM's*, *GBP's*, *OAS1-3*, *MX1/2*, *ISG15*, and *RSAD2* when RIPK2 is knockdown in IRGM$^{+/-}$ HT-29 cells (Fig 6E and F). In agreement with these results, TRRUST (database of literature-curated human TF-target interactions; Han *et al*, 2018) analysis predicted RELA, NF-κB, and STAT1 as the major transcription factors for this response (Fig EV4G).

Taken together, these results demonstrate that IRGM suppresses multiple inflammatory responses during bacterial infection and limits the array of RIPK2-dependent pro-inflammatory responses.

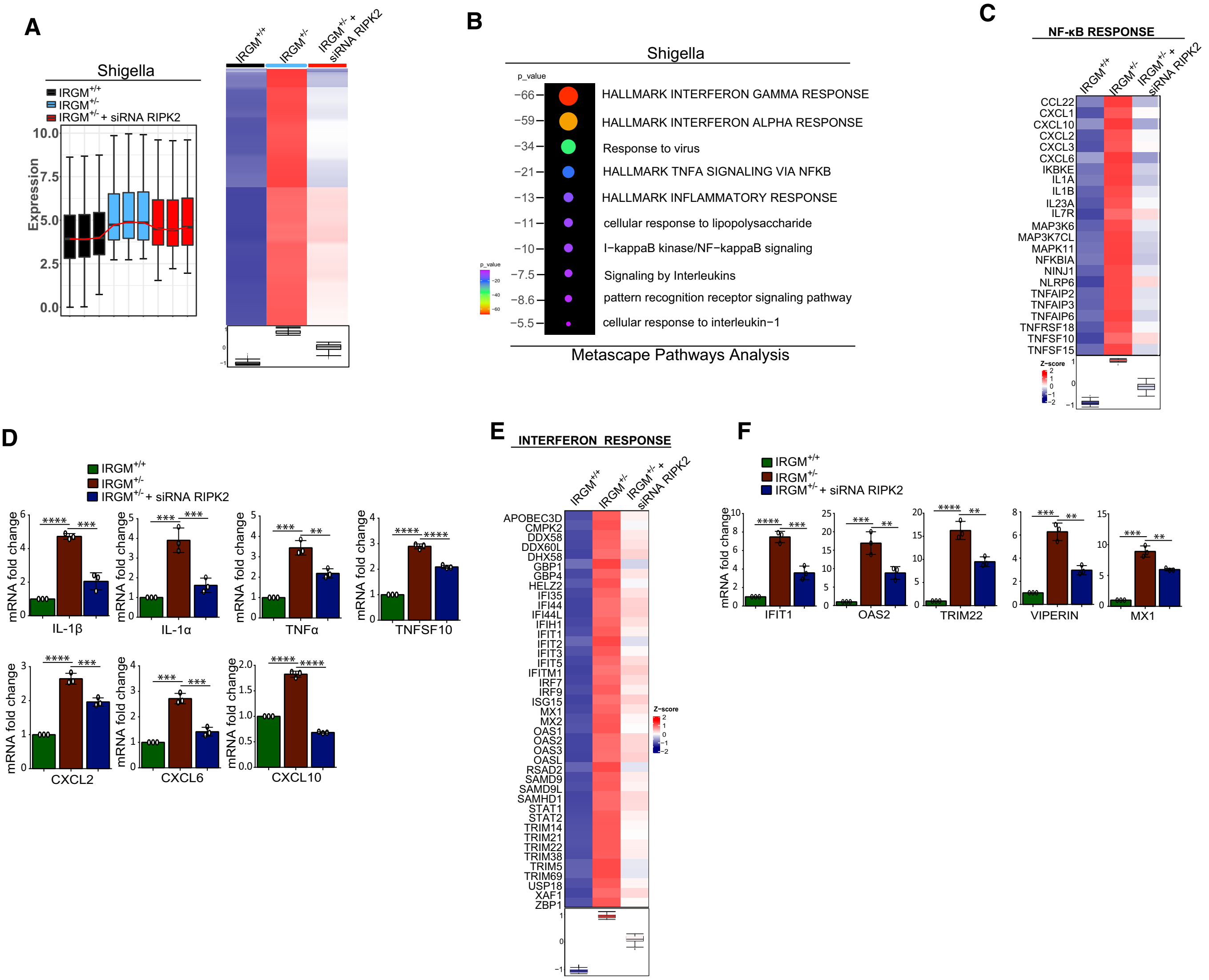

Figure 6. IRGM negatively regulates *Shigella*-induced RIPK2-dependent pro-inflammatory signaling pathways.

A Box plot distribution with normalized log expression (Left panel) and heatmap (Right panel) of the genes that were significantly upregulated in IRGM$^{+/-}$ HT-29 cells (compared with control; 1.5-fold, $P < 0.05$, Wald Chi-Squared test, $n = 3$, biological replicates) and at the same time were significantly rescued by RIPK2 depletion in IRGM$^{+/-}$ HT-29 cells ($P < 0.05$, 0.8, $n = 3$) infected with *S. flexneri* for 6 h (MOI 1:25). Left panel, the box and whisker plot shows upper and lower quartile of the datasets. Right panel, the graph below the heat map shows box plot with median obtained from data scaled to Z-score.

B Bubble plot graph depicting the overrepresented pathways (in order of *P*-value) obtained using Metascape pathway analysis software from the genes significantly upregulated in IRGM$^{+/-}$ cells (compared with control; 1.5-fold, $P < 0.05$, Wald Chi-Squared test, $n = 3$) and at the same time were significantly rescued by RIPK2 depletion in IRGM$^{+/-}$ HT-29 cells ($P < 0.05$, 0.8, $n = 3$) infected with *S. flexneri* for 6 h (MOI 1:25).

C Heatmap depicting the representative NF-κB responsive genes that were significantly upregulated in IRGM$^{+/-}$ cells (compared with control; 1.5-fold, $P < 0.05$, Wald Chi-Squared test, $n = 3$ biological replicates) and at the same time were significantly rescued by RIPK2 depletion in IRGM$^{+/-}$ HT-29 cells ($P < 0.05$, Wald Chi-Squared test, 0.8, $n = 3$ biological replicates). The graph below the heat map shows box plot with median obtained from data scaled to Z-score.

D The qRT–PCR analysis of NF-κB responsive cytokines and chemokines with total RNA isolated from the *S. flexneri*-infected IRGM$^{+/+}$, IRGM$^{+/-}$, and RIPK2-depleted IRGM$^{+/-}$ HT-29 cells. Mean ± SD, $n = 3$ (biological replicates), ***P* < 0.005, ****P* < 0.0005 and *****P* < 0.00005, ordinary one-way ANOVA (Tukey's multiple comparisons test).

E Heatmap depicting the representative IFN response genes significantly upregulated in IRGM$^{+/-}$ cells (compared with control; 1.5-fold, $P < 0.05$, Wald Chi-Squared test, $n = 3$ biological replicates) and at the same time were significantly rescued by RIPK2 depletion in IRGM$^{+/-}$ HT-29 cells ($P < 0.05$, Wald Chi-Squared test, 0.8, $n = 3$, biological replicates). The graph below the heat map shows box plot with median obtained from data scaled to Z-score.

F The qRT–PCR analysis of NF-κB responsive cytokines and chemokines with total RNA isolated from the *S. flexneri*-infected IRGM$^{+/+}$, IRGM$^{+/-}$, and RIPK2-depleted IRGM$^{+/-}$ HT-29 cells. Mean ± SD, $n = 3$ (biological replicates), ***P* < 0.005, ****P* < 0.0005 and *****P* < 0.00005, ordinary one-way ANOVA (Tukey's multiple comparisons test).

Source data are available online for this figure.

 © 2022 The Authors

RIPK2 inhibition ameliorates shigellosis and DSS-induced gut inflammation in *Irgm1*^{KO} mice

Genetic variations in *IRGM* are associated with increased susceptibility to sepsis, bacterial infections, and gut inflammatory diseases (Massey & Parkes, 2007; Parkes *et al*, 2007; Intemann *et al*, 2009; Kimura *et al*, 2014). Consistently, *Irgm1*$^{-/-}$ mice were found to be more susceptible to DSS-induced colitis (Liu *et al*, 2013). Since we found that IRGM negatively regulates RIPK2-dependent proinflammatory responses, we hypothesized that therapeutic inhibition of RIPK2 could reduce gut inflammation associated with *Irgm1* depletion in shigellosis- and DSS-induced colitis models.

For *Shigella* infection in mice, we used the intraperitoneal model of shigellosis (Yang *et al*, 2014). In both colitis models, *Irgm1*-deficiency significantly accelerated body weight loss (Fig 7A and B) and increased the scores of stool consistency and rectal bleeding (Fig EV5A and B). Treatment of *Irgm1*$^{-/-}$ mice with the RIPK2 inhibitor GSK583 (Haile *et al*, 2016; Goncharov *et al*, 2018) significantly ameliorated the acute colitis symptoms (Figs 7A and B, and EV5A and B). Consistently, GSK583 was able to suppress the colon shortening in *Irgm1*$^{-/-}$ mice in both shigellosis- and DSS-induced colitis models (Figs 7C and EV5C). Next, we examined the severity of colon damage and inflammation by histopathological analysis using hematoxylin and eosin (H&E) staining. In *Irgm1*$^{-/-}$ mice, a significant increase in DSS-induced epithelial injury, loss of goblet cells, hyperplasia, and immune cell (neutrophils) infiltration was observed in the colon (Figs 7D and EV5D). Treatment of *Irgm1*$^{-/-}$ mice with GSK583 significantly attenuated histopathology and immune cell invasion (Figs 7D and EV5D). Similarly, *Shigella*-induced colon histopathology in *Irgm1*$^{-/-}$ mice was significantly improved by treatment with GSK583 (Fig EV5E and F).

An enhanced oligomerization of RIPK2 was observed in *Irgm1*$^{-/-}$ mice colon lysates that were dampened by the administration of GSK583 in these mice (Fig 7E). The increased levels of NF-κB (IL1α, IL1β, and TNF-α) and IFN (MX1, IFIT1, and ISG15) dependent cytokine response in *Irgm1*$^{-/-}$ mice colon were suppressed by treatment of GSK583 in both shigellosis- and DSS-induced colitis models (Figs 7F and EV5G). Immunohistochemistry analysis showed enhanced staining of IL1β in *Irgm1*$^{-/-}$ mice colon that was significantly reduced in GSK583-treated *Irgm1*$^{-/-}$ mice (Fig 7G). The enhanced expression of ISG15, RSAD2 (Viperin), and protein ISGylation (IFN response markers) in *Irgm1*$^{-/-}$ mice colon was considerably reduced upon treatment with GSK583 (Fig 7H). Similarly, GSK583 administration considerably diminished the increased protein levels of pro-caspase-1, cleaved caspase-1, phospho-p65, and phospho-p38MAPK in *Irgm1*$^{-/-}$ mice colon (Fig 7H and I).

Taken together, the data show that RIPK2 inhibition can ameliorate the gut inflammation and pathology associated with *Irgm1* deprivation in mouse colitis models.

Discussion

In this study, we made two major advances in understanding the regulation of NODs-RIPK2-NF-κB signaling. First, we revealed that when pathogenic bacteria infect cells, RIPK2 forms RIPosomes, which recruit over the bacteria and induce NF-κB response. Second, we show that autophagy suppresses NF-κB pro-inflammatory signaling by selectively degrading NODs, RIPK2, and RIPosomes. Consequently, this study demonstrates how the two cells' autonomous systems that are loaded over the bacteria work in concert for innate immune defense against the pathogens and cutting down excess inflammation as a safeguard.

The cryogenic electron microscopic (Cryo-EM) structure of RIPosomes was illustrated by two recent studies (Gong *et al*, 2018; Pellegrini *et al*, 2018). They found that RIPK2 forms a filamentous structure in cells using its CARD domain. Interestingly, the helical symmetry of RIPosomes and ASC filaments (Lu *et al*, 2014) was found to be strikingly similar suggesting that their assembly is governed by a similar mechanism. Further, they suggest that the CARDS of NOD1/2 may transiently interact with the CARD of RIPK2 to induce their oligomerization, similar to the phenomenon observed in RIG-I–MAVS signaling complexes (Wu & Hur, 2015). We found that RIPK2 can self-polymerize; however, both NOD1 and NOD2 failed to do so unless co-expressed with RIPK2. This is consistent with previous studies that suggest that NOD1/2 has a low propensity to self-associate (Fridh & Rittinger, 2012; Gong *et al*, 2018). Taken together, it appears that NODs monomers trigger RIPK2 oligomerization, which in turn increases the propensity of NODs to oligomerize and form NODo-RIPosomes.

Ellwanger *et al*, 2019 demonstrated that XIAP controls RIPK2 signaling by preventing its depositions in speck-like structures. Given the role of XIAP in autophagy, it will be interesting to determine whether XIAP has a role in the autophagic degradation of RIPosomes.

Figure 7. RIPK2 inhibition ameliorates shigellosis- and DSS-induced gut inflammation in *Irgm1* knockout mice.

A, B Upper panels, schematic representation of shigellosis- and DSS-induced colitis models used. In lower panels, the graph depicts percentage loss in body weight in *Irgm1*$^{+/+}$ and GSK583 untreated or treated *Irgm*$^{-/-}$ mice when (A) infected with *S. flexneri* (n = 6 mice) or (B) administrated with DSS (n = 3 mice). Mean ± SD, **P* < 0.05, ***P* < 0.005, Student's unpaired *t*-test.

C Left panel, representative pictures of colons of *Irgm1*$^{+/+}$ and *Irgm*$^{-/-}$ mice untreated or treated with GSK583 infected with *S. flexneri*. Right panel, the graph depicts the average colon lengths of the mice groups. Mean ± SD, n = 6, ***P* < 0.005, ****P* < 0.0005, Student's unpaired *t*-test.

D Representative microscopic images of H&E staining of colon tissues of *Irgm1*$^{+/+}$ and *Irgm*$^{-/-}$ mice untreated or treated with GSK583 administrated with DSS. The graph depicts the combined histological scores. Mean ± SD, n = 3 (DSS), ***P* < 0.005, Student's unpaired *t*-test. Scale bar, 200 μm.

E The soluble and insoluble fractionations of lysates from colon tissues of *Shigella*-infected or DSS-treated *Irgm1*$^{+/+}$ and *Irgm*$^{-/-}$ mice treated with GSK583 as indicated, were subjected to immunoblot analysis with indicated antibodies.

F The qRT–PCR analysis with total RNA isolated from the colon tissues of *S. flexneri*-infected Irgm1$^{+/+}$ or Irgm1$^{-/-}$ or GSK583-treated Irgm1$^{-/-}$ mice. Mean ± SD, n = 6–8, **P* < 0.05, ***P* < 0.005, ****P* < 0.0005, ordinary one-way ANOVA (Tukey's multiple comparisons test).

G Representative confocal images of IL-1β immunostained colon tissues of DSS-treated *Irgm1*$^{+/+}$ or *Irgm1*$^{-/-}$ or GSK583-treated *Irgm1*$^{-/-}$ mice. Scale bar, 10 μm.

H, I Western blot analysis with the colon tissue lysates of DSS-treated or *S. flexneri*-infected mice groups as indicated.

Source data are available online for this figure.

© 2022 The Authors

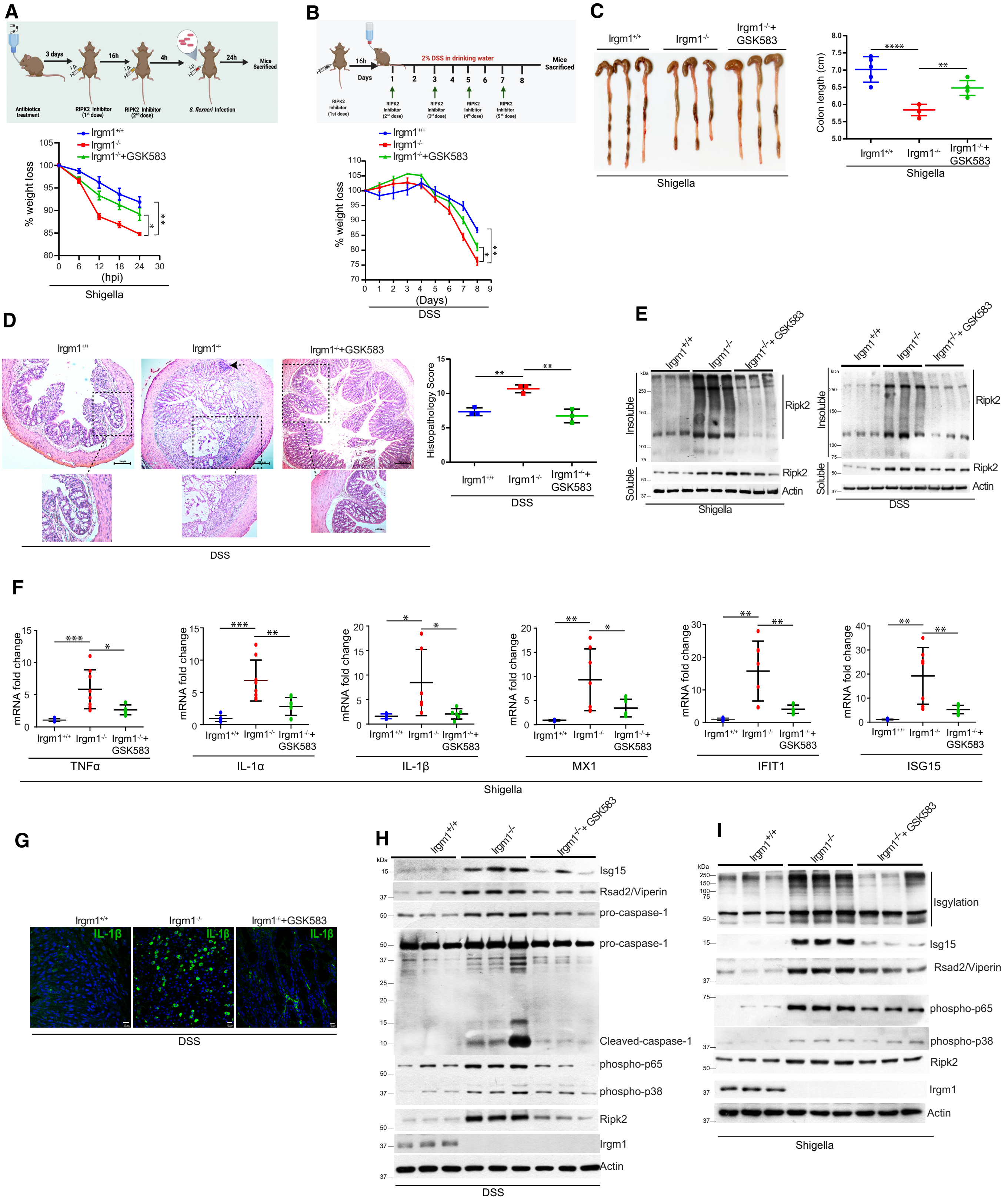

Figure 7.

 © 2022 The Authors

We found that upon bacterial infection endogenous RIPK2 undergoes oligomerization to form RIPosomes in macrophages. Also, we found that pathogenic bacteria have a greater ability to induce RIPosome formation than nonpathogenic bacteria. At present, we do not understand the basis of this difference as both pathogenic and nonpathogenic bacteria produce ligands for NODs activation. It could be a possibility that, in addition to these ligands, some other bacterial factors are required to induce endogenous oligomerization of RIPK2. Consistently, the NODs agonist, MDP, or iE-DAP were inefficient in inducing RIPosome formation. Further studies are needed to identify the bacterial factors that enhance RIPosomes biogenesis. RIPosomes were either juxtaposed or recruited over the pathogenic bacteria. While NODs puncta co-localize with RIPosomes over bacteria, they are less consistent than RIPosome depositions suggesting that like RIG-I-MAVS interaction (Wu & Hur, 2015), NODs might transiently interact with RIPK2 to activate it.

The NODs-RIPK2-dependent NF-κB pro-inflammatory response is important to eliminate the invading pathogens. However, extensive inflammation could be deleterious and lead to immuno-pathologies. To prevent damage, cells employ several cell-autonomous mechanisms to tone down the inflammation. Genetic mutations in components of the NODs-RIPK2-NF-κB pathway predispose toward chronic autoimmune and inflammatory diseases (Miceli-Richard *et al*, 2001; Kanazawa *et al*, 2005; Henckaerts & Vermeire, 2007; Caso *et al*, 2015; Taniguchi & Karin, 2018). Therefore, it is critically important to understand the mechanisms that negatively control this pathway. Autophagy plays a significant role in suppressing inflammation and maintaining innate immune balance in the cell by degrading multiple inflammatory proteins/complexes (Chauhan *et al*, 2021; Deretic, 2021). Our previous study showed that IRGM act as a scaffold protein to bring NOD2-dependent bacterial sensing and autophagy machinery together to conduct antimicrobial defense (Chauhan *et al*, 2015). Here, we found that IRGM utilizes autophagy machinery to dampen the NOD2-RIPK2-dependent inflammation induced by pathogenic bacteria. Together, it emerges that IRGM has a dual function during bacterial infection. On the one hand, it leads to the clearance of intracellular bacteria by inducing xenophagy, and on the other hand, it reduces inflammation by inducing autophagic degradation of NODs and RIPK2.

IRGM is a genetic risk factor for several inflammatory and infectious diseases including inflammatory bowel disease, and tuberculosis (Massey & Parkes, 2007; Parkes *et al*, 2007; Lu *et al*, 2013; Kimura *et al*, 2014; Lin *et al*, 2016; Xia *et al*, 2017; Yao *et al*, 2018). The depletion of *Irgm1* in mice triggers inflammasome and IFN responses leading to autoimmune-like conditions. Depletion of IRGM led to upregulation of several inflammatory responses including NF-κB and IFN responses in a RIPK2-dependent manner that is blocked by chemical inhibition of RIPK2. These data suggest that RIPK2 inhibitors could be used as therapeutic options in patients with inflammatory diseases caused by the loss of function of IRGM.

Materials and Methods

Cell culture

All the common cell lines including THP-1 (ATCC TIB-202), HT-29 (ATCC HTB-38), and HEK293T (ATCC CRL-3216) cells were obtained from the American Type Cell Culture (ATCC), US. HEK-Blue hNOD2 cells were purchased from InvivoGen and maintained as per the instructions. HT-29, HEK293T, and HeLa cells were maintained in DMEM (GIBCO) supplemented with 10% heat-inactivated fetal bovine serum (FBS) and penicillin/streptomycin. THP-1 cells were maintained in RPMI-1640 (GIBCO) supplemented with 10% heat-inactivated FBS, Glucose (5%), HEPES buffer (10 mM), L-glutamine (5 mM), sodium pyruvate (1 mM), and penicillin/streptomycin. All the cell lines were tested for mycoplasma contamination routinely (every 2–3 months). All the experiments were performed with cells below the 20th passage.

Generation of CRISPR/Cas9 KO cell lines

The RIPK2 knockout HT-29 cell line is generated using the CRISPR-Cas9 method. Briefly, HT-29 cells seeded in 6-well plates and transfected with RIPK2/RICK Double Nickase Plasmid (Santacruz; sc-400,731-NIC) using ViaFect transfection reagent (Promega # E4981). After 48 h, GFP-positive cells were sorted into 96-well plates using MoFlo Asterio cell sorter (Beckman Coulter Life Sciences). The cells were grown in a growth medium containing 1 μg/ml puromycin for 1 week. The individual clones were selected and screened for knockout using Western blot analysis. The IRGM knockout HT-29 cell line was generated as described previously (Jena *et al*, 2020).

Inhibitors and reagents

N-Acetylmuramyl-L-alanyl-D-isoglutamine, MDP (InvivoGen # tlrl-mdp); γ-D-glutamyl-meso-diaminopimelic acid, iE-DAP (InvivoGen # tlrl-dap); 6-[(1,1-dimethylethyl) sulfonyl]-N-(5-fluoro-1H-indazol-3-yl)-4-quinolinamine, GSK583 (Cayman # 19739); Phorbol 12-myristate 13-acetate, PMA (Sigma # P8139); Bafilomycin A1 (CST# 54645S); Z-leu-leu-leu-al, MG132 (Sigma # C2211-5MG); Dextran sulfate sodium salt, DSS (MP Biomedicals # 160110), Ubiquitin E1 Inhibitor, PYR-41 (Sigma # 662105), ProLong Gold antifade reagent with DAPI (Invitrogen # P36931), ProLong Gold antifade reagent (Invitrogen # P36930).

Soluble-insoluble fractionation

For soluble-insoluble protein fractionation, cells were lysed in 1% Triton X-100 lysis buffer containing 150 mM NaCl, 50 mM Tris (pH 7.5), 10% glycerol, 1 mM EDTA, 1 mM PMSF, and protease inhibitor cocktail at 4°C for 20 min followed by centrifugation for 10 min at 17,000 *g* at 4°C. After centrifugation, the supernatant was used as a soluble fraction and the pellet was resuspended in lysis buffer with 1% SDS and used as an insoluble fraction for Western blot analysis.

Nuclear and cytoplasmic fractionation

Nuclear and cytoplasmic fractionation assay was performed using the NE-PER Nuclear and Cytoplasmic Extraction Reagents (ThermoFisher Scientific # 78833) according to the manufacturer's protocol. Briefly, 2×10^6 HeLa cells were infected with *S. flexneri* (MOI 1:25) for 4 h. The cells were harvested with trypsin–EDTA and centrifuged at 500 × *g* for 5 min. The cell pellet was washed with 1× PBS and resuspended in 200 μl CER I, vortexed vigorously for 15 s, and incubated on ice for 10 min. After the incubation, 11 μl of ice-

cold CER II were added to each sample, vortexed followed by incubation on ice for 1 min. The samples were centrifuged at 16,000 × *g* for 5 min to extract cytoplasmic fraction (supernatant) and the pellet (insoluble fraction) was resuspended in 100 μl NER, vortexed, incubated on ice with intermittent vortexing for 40 min. The samples were centrifuged at 16,000 × *g* for 10 min at 4°C to isolate nuclear fraction and subjected to western blot analysis.

Cycloheximide chase assay

Approximately, 2×10^6 HT-29 cells were plated in a 6-well plate and grown overnight. The next day, cells were treated with cycloheximide (100 μg/ml) with or without Bafilomycin A1 (300 nM) or MG132 (20 μM) for various time points. The cells were lysed in NP-40 lysis buffer containing 1× protease inhibitors cocktail and 1 mM PMSF and subjected to Western blotting with indicated antibodies.

Western blotting

The cells were lysed using NP-40 lysis buffer (Invitrogen # FNN0021) containing 1 mM PMSF (Sigma # P7626), phosSTOP (Roche # 49068455001), and protease inhibitors cocktail (Roche # 1183617000). Mice tissue were homogenized in radio-immunoprecipitation assay (RIPA) buffer (20 mM Tris, pH 8.0; 0.5 EGTA; 1 mM EDTA; 150 mM NaCl; 1% IGEPAL (Sigma); 10% glycerol; 0.1% Sodium deoxycholate). The protein concentration in the lysate was determined using the BCA method (Pierce™ BCA Protein Assay Kit # 23225). Lysates were separated using SDS–PAGE, transferred onto a nitrocellulose membrane (Bio-Rad), and blocked for 1 h in 5% skimmed milk followed by incubation in primary antibody overnight at 4°C. The membrane was washed with 1× PBS/PBST three times and then incubated with HRP conjugated secondary antibody for 1 h at room temperature. After washing with 1× PBS/PBST, the blots were developed using enhanced chemiluminescence reagents.

Immunofluorescence assay

Approximately, HEK293T/HeLa 10^5 cells were seeded on a coverslip and allowed to adhere overnight before transfection with plasmids. For THP-1 cells, approximately, 5×10^5 cells were treated with 50 ng/ml of PMA (Sigma # P8139) and seeded on the coverslip for differentiation into macrophages for 16 h. After a resting period of 24 h, cells were treated with stimulants or infected as required. The cells were fixed in 4% paraformaldehyde for 15 min, permeabilized with 0.1% Triton X-100 (or 0.1% saponin) for 15 min, followed by blocking with 1% bovine serum albumin (BSA) for 30 min at room temperature (RT). The permeabilized cells were then incubated with primary antibody as indicated for 1 h at RT or overnight at 4°C, washed thrice with 1× PBS, followed by incubation with Alexa fluor-conjugated secondary antibodies for 1 h at RT. The cells were washed thrice with 1× PBS, mounted with ProLong™ Gold antifade mountant with or without DAPI, air-dried, and visualized using a super-resolution Leica SP8 lightning confocal microscope.

Proximity ligation assay (PLA)

Proximity ligation assay (PLA) was performed using a Duolink *in situ* detection kit as per the manufacturer's protocol (Sigma #DUO92008). Briefly, 2×10^5 HEK293T cells were seeded on the coverslip and allowed to adhere overnight before transfection with the desired plasmids. After 12 h of transfection, cells were fixed with a 4% paraformaldehyde solution. Next, antigen retrieval was performed in sodium citrate buffer (10 mM, pH 6.0) and the cells were permeabilized with 0.1% Triton X-100 followed by blocking with 1× blocking solution (provided with the kit) in a preheated humidity chamber for 1 h at 37°C. The cells were incubated overnight with primary antibody diluted with diluent at 4°C. Next, the coverslips were washed twice with 1× wash buffer-A followed by incubation with Duolink *in situ* PLA probes in a preheated humidity chamber for 1 h at 37°C. After PLA probe incubation, coverslips were washed twice with 1× wash buffer-A followed by ligation with Duolink *in situ* ligase for 30 min in a preheated humidity chamber at 37°C. Next, coverslips were washed twice with 1× wash buffer-A followed by amplification through Duolink *in situ* polymerase for 90 min in a preheated humidity chamber at 37°C. Final washing was done twice with 1× and 0.01× wash buffer-B. The coverslips were mounted over glass slides using Duolink *in situ* mounting media with DAPI and the edges of the coverslip were sealed, mounted, and visualized using a Leica SP8 confocal microscope.

High-content microscopy imaging

For high-content microscopy imaging, approximately 17,000 HEK293T cells, 8,000 HeLa cells, and 50,000 PMA-treated THP-1 cells were seeded in a black flat bottom 96-well plate (Thermo Scientific, Nunc) and transfected with plasmids or treated or infected as indicated. The cells were fixed with 4% PFA and permeabilized with 0.1% Triton X-100 for 15 min, followed by blocking with 1% bovine serum albumin (BSA) for 30 min and incubation with primary antibody for 1 h. The cells were washed thrice with 1× PBS and incubated with secondary antibody for 1 h and then counterstained with 0.5 μg/ml of DAPI for 5 min. Imaging was performed by using Cell insight CX7 LZR high-content screening platform (Thermo Scientific). Automated image scanning and analysis were carried out using HCS studio and iDEV software, respectively. Automated images were captured at 10× (or 20×) by taking 1,500–20,000 cells and 35 fields per well or as indicated in figure legends. Images were quantified using scanning parameters and object mask definitions. A threshold value was set to find out the primary objects (cells). DAPI staining was used for autofocus and identification of the primary objects. The cells and regions of interest (ROI) or desired targets were further validated by total area, shape/segmentation, maximum/minimum average intensity, and total intensity. By using the cell mask and intensity of the puncta, the number of puncta per cell was counted. All data processed and analyzed were computer-based.

Hematoxylin and eosin (H&E) staining and immunohistochemistry analysis for mice colon tissue

The colon sections were deparaffinized and hydrated through an alcohol gradient followed by 1× PBS wash. For hematoxylin and eosin staining, the sections were stained with hematoxylin for 5 min followed by washing in water to remove excess stain. The sections were then incubated in Scott's tap water followed by staining with Eosin dye. The slides were washed, dehydrated in absolute ethanol, cleared in xylene, and mounted with DPX. Finally, slides were observed under Zeiss Apotome 2.0 microscope.

© 2022 The Authors

For immunohistochemistry, colon sections were deparaffinized and hydrated through ethanol gradient followed by 1× PBS wash. Antigen retrieval was performed in sodium citrate buffer (pH 6.0) for 10 min followed by permeabilization with 1× PBS (pH 7.4) containing 0.25% Triton X-100, blocked with goat serum. The slides were washed for 2 min thrice with PBS (pH 7.4) containing 0.05% Tween 20 followed by incubation of sections with an unconjugated affinity purified F(ab) fragment anti-mouse IgG (H + L) (Abcam #ab6668) for 1 h at room temperature. The sections were incubated overnight at 4°C with antibodies as indicated. The next day, the sections were washed twice with 1× PBS (pH 7.4) containing 0.05% Tween 20, followed by incubation with goat anti-rabbit/mouse IgG (H + L) Alexa Fluor 488/568 conjugated secondary antibody for 1 h. Sections were again washed and counterstained with DAPI for 1 min followed by incubation with an auto-fluorescence quencher (Vector Labs #SP-8400). Sections were finally mounted with Vectashield Vibrance Antifade mounting media. The slides were visualized under Leica TCS SP8 STED confocal microscope.

Co-immunoprecipitation assay

The cells were lysed in NP-40 lysis buffer containing 1× protease inhibitors cocktail and 1 mM PMSF for 20 min at 4°C and centrifuged at 12,000 *g* for 30 min at 4°C. The supernatant was incubated with the antibody at 4°C for 2 h on a rotational cell mixer followed by incubation with protein G Dynabeads (ThermoFisher#10003D) for 2 h at 4°C on a rotational cell mixer. The beads were washed with ice-cold 1× PBS four times, and the proteins were eluted from the beads in 2× SDS–PAGE Laemmli buffer by boiling for 5 min for Western blot analysis.

Glutathione S-Transferase (GST)-pull-down assay

The glutathione S-transferase (GST) pull-down assay was performed according to the previously described methods (Mehto *et al*, 2019a, 2019b). Briefly, GST or GST-RIPK2 or GST-IRGM recombinant proteins were expressed in *E. coli* SoluBL21 (Amsbio), and the proteins were purified on Glutathione Sepharose 4 Fast-Flow beads (GE Healthcare). The [^{35}S] labeled- Myc-NOD1, Myc-NOD2, Myc-RIPK2, Myc-ULK1, Myc-ATG16L1 or Myc-Beclin-1 proteins were *in vitro* translated using TnT T7–coupled reticulocyte lysate system (Promega). The GST proteins were incubated with [^{35}S]-labeled proteins in 200 µl of NETN-E buffer (50 mmol/l Tris, pH 8.0, 100 mm NaCl, 6 mm EDTA, 0.5% NP-40, and 1 mm dithiothreitol (DTT) supplemented with complete mini EDTA-free protease inhibitor cocktail; Roche) for 2 h at 4°C. After incubation, the beads were washed five times with NETN-E buffer, boiled with loading buffer, and subjected to SDS–PAGE. The gels were stained with coomassie blue and vacuum-dried. The GST was detected by staining with coomassie blue stain, whereas the [^{35}S]- labeled Myc-tagged proteins were detected in PharosFX imager (Bio-Rad Laboratories).

Antibodies and dilutions

Western blotting:

IRGM (Abcam # 69494; 1:500), NOD1 (CST# 3545S; 1:1000), NOD2 (Proteintech # 20980-1-AP; 1:1000), RIPK2 (CST#4142S; 1:1000), Anti-beta Actin (Abcam # ab6276; 1:5000), GFP (Abcam # ab290; 1:5000), Flag M2 (Sigma # F3165; 1:1000), p62 (BD Biosciences # BD-610832; 1:1000), ATG5 (CST # 2630S; 1:1000), c-Myc (Santa Cruz # sc-40; 1:1000), HA-Tag (CST # 3724S; 1:1000), RICK (Santa Cruz # sc-22,763; 1:1000), Anti-LC-3B antibody (Sigma # L7543; 1:1000), Anti-RICK A-10 (Santa Cruz # sc-166,765; 1:1000), IL-1β (CST#12242), Anti-pro Caspase1 + p10 + p12 Antibody (Abcam # ab179515; 1:1000), ISG15 (Santa Cruz# sc-166755; 1:500), Phospho-p38 MAPK (Thr180/Tyr182; 1:1000; CST#9211; 1:1000), Phospho-NF-κB p65 (ser536) (93H1) (CST# 3033; 1:1000). HRP conjugate secondary antibodies were purchased from Novus (1:2000) or Promega (1:5000).

Immunofluorescence

IRGM (Santa Cruz #68202; 1:50), RICK H-300 (Santa Cruz # sc-22,763; 1:100), NOD2 (Millipore # 04-145; 1:50), p62 (BD Biosciences # BD-610832; 1:250), LC3b (Sigma # L7543; 1:250), LC3b (MBL # PM036; 1:100), LAMP2A (Abcam # ab18528; 1:50), EEA1 (CST # 3288; 1:50), FK2 (MBL # D058-3;1:250), LC3b (CST # 83506, 1:50), IL-1β (CST # 12242), ULK1 (Santa Cruz # 33182; 1:100), c-Myc (Santa Cruz # sc-40; 1:500), Flag M2 (Sigma # F3165; 1:250), Alexa fluor Secondary antibodies (1:2000) were purchased from Thermo Fisher Scientific.

Plasmids and deletion constructs

The mcherry-RIPK2, GFP-RIPK2 and its deletion construct, GFP-NOD1 and its deletion construct and GFP-NOD2 were generated using gateway cloning strategy as per standard protocols (Invitrogen). pGL4.32[luc2P NF-kB-RE Hygro] purchased from Promega (Promega # E8491).

Transient transfection with siRNA

The THP-1 cells were electroporated using the Neon transfection system (Invitrogen # MPK5000; setting: 1400 V, 10 ms, 3 pulses, 100 µl tip) with 30 nM siRNA or as indicated: nontargeting siRNA, p62 siRNA (SASI_Hs01_00118616), IRGM siRNA (SASI_HS02_00518571), ATG5 (SASI_Hs01_00173156), human RIPK2 siRNA (SASI_Hs01_00199696), mice RIPK2 siRNA (SASI_Mm01_00188069) from Sigma-Aldrich. After 24-h transfection, one more time transfection was performed in a similar condition and incubated for an additional 48 h before the start of each experiment. The HT-29, HeLa, and HEK293T cells were transfected with 30 nM siRNA using Lipofectamine RNAiMAX transfection reagents (Invitrogen# 13778075) as per the manufacturer's instructions.

Transient transfection with plasmid

For transient transfection in THP-1, cells were electroporated using the Neon transfection system (Invitrogen # MPK5000; setting: 1400 V, 10 ms, 3 pulses, 100 µl tip). For overexpression experiments in HEK293T cells, required plasmids were transfected using the calcium phosphate method as per the manufacturer's instruction (Clontech, Promega).

Flag-RIPK2 protein electroporation

Approximately, 2×10^6 HEK293T cells transiently expressing pGL4.32NFκB-RE reporter plasmid electroporated with 10 µg

© 2022 The Authors

Purified Flag-RIPK2 in 100 µl neon resuspension buffer (setting: 1300 V, 10 ms, 1 pulse, 100 µl tip). The cells were harvested 6-h postelectroporation, and a luciferase assay was performed as described below.

NF-κB reporter assay

The NF-kb reporter assay was performed using a luciferase assay kit (Promega) according to the manufacturer's instructions and as described previously (Jena *et al*, 2020). Briefly, HEK293T cells were seeded into the 24-well plate. The next day, cells were transfected with pGL4.32 NF-κB-RE (Addgene100 ng) together with required plasmids using the calcium phosphate method. After 9 h, the growth medium was removed and cells were washed thrice with 1× PBS and lysed using 100 µl (1X) passive lysis buffer (Promega). The cell lysates were cleared by centrifugation at 12,000 *g* for 30 s at 4°C, and protein concentration was estimated using the BCA method. In a 96-well plate, 15 µg/20 µl lysate mixed with 100 µl LARII reagent and luminescence measurement was performed using a PerkinElmer VICTOR Nivo Multimode plate reader.

Secreted embryonic alkaline phosphatase (SEAP) reporter assay

Approximately, 3×10^5 HEK293-hNOD2 Blue cells were plated in a 6-well culture plate and incubated at 37°C in a 5% CO_2 incubator overnight. The next day, cells were transfected with the plasmids as indicated using the calcium phosphate method as per the manufacturer's instruction (Clontech, Promega). Six-hours post-transfection, the cell culture medium was replaced with fresh medium with or without L-18 MDP (100 ng/ml) and incubated at 37°C in a 5% CO_2 incubator for 24 h. SEAP activity was determined using QUANTI-Blue reagent as per the manufacturer's instruction. Briefly, in a flat bottom 96-well plate, 20 µl of sample supernatant was mixed with 180 µl of QUANTI-Blue solution and incubated for 30 min at 37°C. Optical density (OD) was measured at 620–655 nm using a microplate reader (Bio-Rad).

RNA isolation and quantitative real-time PCR

The total RNA was extracted using TRIzol™ isolation reagent (Invitrogen # 15596026) according to the manufacturer's protocol. One to two microgram of total RNA was used to synthesize cDNA using the high-capacity cDNA synthesis kit (Applied Biosystem #4368813), and qRT–PCR was performed using Power SYBR green PCR master mix (Applied Biosystem #4367659) or TaqMan master mix (Applied Biosystem # 4369016) according to the manufacturer's protocol. The assay was normalized using the housekeeping gene (GAPDH or β-Actin). The fold change in gene expression was calculated by the $2^{-\Delta\Delta Ct}$ method. The graphs were generated using Graph Pad software. The sequence for human and mouse qRT–PCR primers is shown in Appendix Table S1.

RNA-sequencing sample preparation

The total RNA was extracted from the cells using an RNeasy mini kit (QIAGEN #74104). The quality and quantity of RNA were checked using agarose gel and Qubit 3.0. After assessing the quality of RNA, ~850 ng of total RNA was taken for library preparation using NEBNext®Ultra™ II Directional RNA Library kit for Illumina® (# E7760L) and NEBNext® Poly (A) mRNA Magnetic Isolation Module (# E7490L) as per manufacturer's protocol. The prepared library was quantified using a Qubit dsDNA assay kit (Invitrogen, Q32851) followed by a quality check (QC) and fragment size distribution using a High Sensitivity Tape station Kit (Agilent 2200, 5067–5585, and 5067–5584). The library was sequenced using the HiSeq 4000 Illumina platform.

RNA-sequencing data processing and gene expression analysis

The paired-end (PE) reads quality checks for each sample were performed using FastQC v.0.11.5 (http://www.bioinformatics.babraham.ac.uk/projects/fastqc/). The adapter sequence was trimmed using the BBDuk version 37.58, and the alignment was performed using STAR v.2.5.3a with default parameters (Dobin *et al*, 2013) with human hg38 genome build, gencode v21 gtf 9GRCh38 from the gencode. Duplicates were removed using Picard-2.9.4 (https://broadinstitute.github.io/picard/) from the aligned files, and read counts were generated using featureCount v.1.5.3 from subread-1.5.3 package (https://bioweb.pasteur.fr/packages/pack@subread@1.5.3) with Q = 10 for mapping quality. The count files were used as input for downstream differential gene expression analysis with DESeq2 version 1.14.1 9. (Love *et al*, 2014). The genes with read counts of ≤ 10 in any comparison were removed followed by count transformation and statistical analysis using DESeq "R." The "*P*" values were adjusted using the Benjamini and Hochberg multiple testing correction (Haynes, 2013), and the differentially expressed genes were identified (fold change of ≥ 1.5, *P*-value < 0.05). A unified nonredundant gene list was made for different comparisons and subjected to gene ontology (GO) analysis using reactome database (https://reactome.org/). The top pathways ($P < 0.05$) were used for generating heat maps using Complexheatmap (Version 2.0.0) through unsupervised hierarchical clustering. The expression clusters were annotated based on enriched GO terms. Normalized gene expression was used to generate the boxplots with median depicting the trends in the expression across the different conditions using ggplot2 [version 3.3.5]. The pathways analysis was performed using Metascape database (https://metascape.org/gp/index.html#/main/step1). The top pathways ($P < 0.05$) were taken for constructing bubble plots using ggplot2 [version 3.3.5].

The basal conditions groups (for the *Salmonella* infection group) for the dataset E-MTAB-12074 are the same as described previously (Jena *et al*, 2020) and deposited under the accession number E-MTAB-9142 (http://www.ebi.ac.uk/arrayexpress/experiments/E-MTAB-9142). The RNA-sequencing experiment for the basal conditions (dataset E-MTAB-9142) and Salmonella-infected conditions (dataset E-MTAB-12074) was performed together. However, we have used basal conditions groups' dataset E-MTAB-9142 in our previous publication (Jena *et al*, 2020) and reanalyzed it here along with Salmonella-infected dataset E-MTAB-12074.

Bacterial strains and infection of cells in culture

Escherichia coli DH5α, *Salmonella typhimurium* strain ST 1433, and *Shigella flexneri* (MTCC:1457) (gifted by Dr. Suraj Kumar Tripathy, KIIT, India) were cultured in Luria-Bertani (LB) overnight at 37°C with shaking. Bacteria were subcultured (1:100 dilution) in fresh LB

 © 2022 The Authors

broth and grown until OD_{600} reached 0.4–0.6. *Mycobacterium smegmatis* MC^2 155 *and Mycobacterium tuberculosis* H37Rv (gifted by Dr. Sunil Raghav, ILS, India) were grown in Middlebrook 7H9 liquid medium supplemented with 5 g/l albumin, 2 g/l dextrose, and 0.003 g/l catalase along with 0.05% tween 80.

For fluorescence microscopy, THP-1 cells were treated with PMA (50 ng/ml) and seeded onto glass coverslips in 12-well plates and kept for differentiation. After 16 h, the medium was replaced with fresh medium and incubated for another 24 h. Cells were infected with *Shigella flexneri* 1457 (MOI, 1:25 or 1:50), *Salmonella typhimurium* strain *S*T 1433 (MOI, 1:10), *E. coli* LF-82 (MOI, 1:10), *Mycobacterium smegmatis* MC^2 155 (MOI, 1:10), *and Mycobacterium tuberculosis* H37Rv (MOI, 1:10). Infection was facilitated by centrifugation at 700 × *g* for 10 min at room temperature and proceeded for 2 h at 37°C on 5% CO_2 incubator. Infected cells were washed three times with 1× PBS and fixed with 4% paraformaldehyde for 30 min at room temperature.

For the RNA-sequencing experiment, approximately 3×10^6 HT-29 cells were seeded in a 60 mm dish and allowed to adhere overnight. The cells were infected with *Shigella flexneri* 1457 (1:25 MOI, 6 h) or *Salmonella typhimurium* strain *S*T 1433 (MOI, 1:10, 8 h).

Intraperitoneal *Shigella* infection in mice

The mice experiments were performed with the procedure approved by the institutional animal ethical committee at the Institute of Life Sciences (ILS), Bhubaneswar, India. The mice were housed at the animal house facility of ILS. C57BL/6 wild-type and *Irgm1* knockout mice were described previously (Liu *et al*, 2013). About 6–8-week-old male $Irgm1^{+/+}$ (wild-type, $n = 9$) and $Irgm1^{-/-}$ ($n = 18$, includes GSK583 group) mice were used for the infection. Intraperitoneal infection of *S. flexneri* was performed as reported previously (Yang *et al*, 2014). Briefly, *Shigella* was cultured to the O.D. of 0.4–0.5 and 10^8 colony-forming units (CFU) were injected intraperitoneal into the mice. GSK583 (30 mg/kg of body weight) is administered intraperitoneally 6 h before the infection. Mice were monitored for body weight, stool consistency, and other clinical parameters. All mice were sacrificed 24-h postinfection. The fecal pathology scores were assigned as follows: stool consistency (0, normal; 1, loose; 2, soft; 3, hard) and color (0, brown; 1, yellow; 2, light green; 3, green).

Pathological scores assigned in colon tissue sections for *Shigella* infection in mice based on the following parameter (Erben *et al*, 2014): hyperplasia (1, less than 25%; 2, mild 26–35%; 3, moderate 36–50%; 4, marked 51% above), loss of goblet cells(1, minimal less than 25%; 2, mild 26–35%; 3, moderate 36–50%; 4, marked 51% above), Leucocytes infiltrate (1, minimal less than 10%; 2, mild 26–35%; 3, moderate 36–50%; 4, marked 51% above).

Dextran sulfate sodium (DSS)-induced colitis model in mice

The 6–7-week-old female C57BL/6 $Irgm1^{+/+}$ ($n = 3$) and $Irgm1^{-/-}$ ($n = 6$, including GSK583 group) mice were used for the DSS-induced colitis experiment. All the mice were given 2% (wt/vol) DSS dissolved in drinking water for 8 days. The DSS solution was replaced on alternate days. To inhibit the RIPK2, GSK583 (25 mg/kg) was injected intraperitoneally (i.p) 16 h before the start of DSS treatment and also injected intraperitoneally on the 1st, 3rd, 5th, and 7th day. The control group mice were injected intraperitoneally with vehicle control. Each day, mice were monitored for body weight, stool consistency, and the presence of blood in feces. The stool scores were assigned as follows: 0, well-formed pellets; 1, semisolid; 2, semisolid and not adhere to the anus; 3, liquid and adhere to the anus; and 4, diarrhea. Bleeding scores were assigned as follows; 0, no blood in stool; 1, light faint; 2, clear visible; and 3 gross rectal bleeding. The mice were sacrificed on the 9th day. The entire colon was extracted and measured using the vernier scale. The colon tissue was homogenized in RIPA buffer or Triton X-100 buffer containing PMSF, phosSTOP, and protease inhibitors cocktails and subjected to Western blotting. The tissue was homogenized in trizol for total RNA extraction.

Pathological scores assigned in colon tissue sections for DSS-induced colitis mice based on the following parameter (Erben *et al*, 2014): hyperplasia (1, less than 25%; 2, mild 26–35%; 3, moderate 36–50%; 4, marked 51% above), loss of goblet cells (1, minimal less than 25%; 2, mild 26–35%; 3, moderate 36–50%; 4, marked 51% above), Leucocytes infiltrate (1, minimal less than 10%; 2, mild 26–35%; 3, moderate 36–50%; 4, marked 51% above).

Software and statistical significance

GraphPad Prism 6 was used to analyze and present most of the data. High-content microscopy imaging data were scanned and analyzed using HCS studio and iDEV software, respectively. The number of biological/technical replicates mentioned in each figure legend. The unpaired Student's *t*-test or ordinary one-way ANOVO (Tukey's multiple comparison test) statistical methods are used as appropriate. The *P*-values are mentioned in graphs as appropriate. The densitometry analysis was performed using Image J software.

All the graphical representations are created using Biorender online tool (https://biorender.com/).

Data availability

The RNA-seq datasets produced in this study have been deposited in the ArrayEXpress database at EMBL-EBI (www.ebi.ac.uk/arrayexpress) under accession numbers E-MTAB-12067 and E-MTAB-12074.

Expanded View for this article is available online.

Acknowledgements
This work is funded by Wellcome Trust/Department of Biotechnology (DBT) India Alliance (IA/I/15/2/502071) fellowship to SC, extramural grant from the Department of Biotechnology (DBT, BT/PR45223/MED/29/1613/2022) to SC, and DST-Inspire faculty fellowship (DST/INSPIRE/04/2019/001857) to SM. Funding from EMBO Global investigator fellowship to SC is greatly acknowledged. We gratefully acknowledge the support of the Institute of Life Sciences central facilities (Animal house, microscopy, FACS, and sequencing) funded by the Department of Biotechnology (India).

Author contributions
Santosh Chauhan: Conceptualization; data curation; formal analysis; supervision; funding acquisition; investigation; visualization; methodology; writing – original draft; project administration; writing – review and editing.
Subhash Mehto: Data curation; supervision; funding acquisition;

© 2022 The Authors

validation; investigation; methodology; writing – review and editing. **Kautilya Kumar Jena:** Data curation; formal analysis; investigation; methodology; writing – review and editing. **Rina Yadav:** Data curation; investigation; methodology. **Swatismita Priyadarsini:** Validation; investigation; methodology. **Pallavi Samal:** Investigation; methodology. **Sivaram Krishna:** Investigation; methodology. **Kollori Dhar:** Investigation; methodology. **Ashish Jain:** Investigation; methodology. **Nishant Ranjan Chauhan:** Investigation; methodology. **Krushna Chandra Murmu:** Investigation; methodology. **Ramyasingh Bal:** Investigation; methodology. **Rinku Sahu:** Investigation; methodology. **Pundrik Jaiswal:** Investigation; methodology. **Bhabani Sankar Sahoo:** Investigation; visualization. **Srinivas Patnaik:** Supervision; methodology. **Thomas A Kufer:** Resources; writing – review and editing. **Tor Erik Rusten:** Resources; supervision. **Swati Chauhan:** Data curation; investigation; methodology. **Punit Prasad:** Resources; supervision; visualization.

Disclosure and competing interests statement

The authors declare that they have no conflict of interest.

References

Cai X, Chen J, Xu H, Liu S, Jiang QX, Halfmann R, Chen ZJ (2014) Prion-like polymerization underlies signal transduction in antiviral immune defense and inflammasome activation. *Cell* 156: 1207 – 1222

Caruso R, Warner N, Inohara N, Nunez G (2014) NOD1 and NOD2: signaling, host defense, and inflammatory disease. *Immunity* 41: 898 – 908

Caso F, Galozzi P, Costa L, Sfriso P, Cantarini L, Punzi L (2015) Autoinflammatory granulomatous diseases: from Blau syndrome and early-onset sarcoidosis to NOD2-mediated disease and Crohn's disease. *RMD Open* 1: e000097

Chauhan S, Jena KK, Mehto S, Chauhan NR, Sahu R, Dhar K, Yadav R, Krishna S, Jaiswal P, Chauhan S (2021) Innate immunity and inflammophagy: balancing the defence and immune homeostasis. *FEBS J* 289: 4112 – 4131

Chauhan S, Mandell MA, Deretic V (2015) IRGM governs the core autophagy machinery to conduct antimicrobial defense. *Mol Cell* 58: 507 – 521

Deretic V (2021) Autophagy in inflammation, infection, and immunometabolism. *Immunity* 54: 437 – 453

Dobin A, Davis CA, Schlesinger F, Drenkow J, Zaleski C, Jha S, Batut P, Chaisson M, Gingeras TR (2013) STAR: ultrafast universal RNA-seq aligner. *Bioinformatics* 29: 15 – 21

Du Y, Duan T, Feng Y, Liu Q, Lin M, Cui J, Wang RF (2018) LRRC25 inhibits type I IFN signaling by targeting ISG15-associated RIG-I for autophagic degradation. *EMBO J* 37: 351 – 366

Ellwanger K, Briese S, Arnold C, Kienes I, Heim V, Nachbur U, Kufer TA (2019) XIAP controls RIPK2 signaling by preventing its deposition in speck-like structures. *Life Sci Alliance* 2: e201900346

Erben U, Loddenkemper C, Doerfel K, Spieckermann S, Haller D, Heimesaat MM, Zeitz M, Siegmund B, Kuhl AA (2014) A guide to histomorphological evaluation of intestinal inflammation in mouse models. *Int J Clin Exp Pathol* 7: 4557 – 4576

Fabregat A, Jupe S, Matthews L, Sidiropoulos K, Gillespie M, Garapati P, Haw R, Jassal B, Korninger F, May B *et al* (2018) The Reactome pathway knowledgebase. *Nucleic Acids Res* 46: D649 – D655

Fridh V, Rittinger K (2012) The tandem CARDs of NOD2: intramolecular interactions and recognition of RIP2. *PLoS ONE* 7: e34375

Girardin SE, Tournebize R, Mavris M, Page AL, Li X, Stark GR, Bertin J, DiStefano PS, Yaniv M, Sansonetti PJ *et al* (2001) CARD4/Nod1 mediates NF-kappaB and JNK activation by invasive *shigella* flexneri. *EMBO Rep* 2: 736 – 742

Glasser AL, Boudeau J, Barnich N, Perruchot MH, Colombel JF, Darfeuille-Michaud A (2001) Adherent invasive *Escherichia coli* strains from patients with Crohn's disease survive and replicate within macrophages without inducing host cell death. *Infect Immun* 69: 5529 – 5537

Goncharov T, Hedayati S, Mulvihill MM, Izrael-Tomasevic A, Zobel K, Jeet S, Fedorova AV, Eidenschenk C, deVoss J, Yu K *et al* (2018) Disruption of XIAP-RIP2 association blocks NOD2-mediated inflammatory signaling. *Mol Cell* 69: 551 – 565

Gong Q, Long Z, Zhong FL, Teo DET, Jin Y, Yin Z, Boo ZZ, Zhang Y, Zhang J, Yang R *et al* (2018) Structural basis of RIP2 activation and signaling. *Nat Commun* 9: 4993

Haile PA, Votta BJ, Marquis RW, Bury MJ, Mehlmann JF, Singhaus R Jr, Charnley AK, Lakdawala AS, Convery MA, Lipshutz DB *et al* (2016) The identification and pharmacological characterization of 6-(tert-Butylsulfonyl)-N-(5-fluoro-1H-indazol-3-yl)quinolin-4-amine (GSK583), a highly potent and selective inhibitor of RIP2 kinase. *J Med Chem* 59: 4867 – 4880

Han H, Cho JW, Lee S, Yun A, Kim H, Bae D, Yang S, Kim CY, Lee M, Kim E *et al* (2018) TRRUST v2: an expanded reference database of human and mouse transcriptional regulatory interactions. *Nucleic Acids Res* 46: D380 – D386

Haynes W (2013) Benjamini–Hochberg method. In *Encyclopedia of Systems Biology*, Dubitzky W, Wolkenhauer O, Cho K-H, Yokota H (eds), pp 78 – 78. New York, NY: Springer New York

He X, Zhu Y, Zhang Y, Geng Y, Gong J, Geng J, Zhang P, Zhang X, Liu N, Peng Y *et al* (2019) RNF34 functions in immunity and selective mitophagy by targeting MAVS for autophagic degradation. *EMBO J* 38: e100978

Henckaerts L, Vermeire S (2007) NOD2/CARD15 disease associations other than Crohn's disease. *Inflamm Bowel Dis* 13: 235 – 241

Hou F, Sun L, Zheng H, Skaug B, Jiang QX, Chen ZJ (2011) MAVS forms functional prion-like aggregates to activate and propagate antiviral innate immune response. *Cell* 146: 448 – 461

Intemann CD, Thye T, Niemann S, Browne EN, Amanua Chinbuah M, Enimil A, Gyapong J, Osei I, Owusu-Dabo E, Helm S *et al* (2009) Autophagy gene variant IRGM -261T contributes to protection from tuberculosis caused by *Mycobacterium tuberculosis* but not by *M. africanum* strains. *PLoS Pathog* 5: e1000577

Jena KK, Mehto S, Nath P, Chauhan NR, Sahu R, Dhar K, Das SK, Kolapalli SP, Murmu KC, Jain A *et al* (2020) Autoimmunity gene IRGM suppresses cGAS-STING and RIG-I-MAVS signaling to control interferon response. *EMBO Rep* 21: e50051

Kanazawa N, Okafuji I, Kambe N, Nishikomori R, Nakata-Hizume M, Nagai S, Fuji A, Yuasa T, Manki A, Sakurai Y *et al* (2005) Early-onset sarcoidosis and CARD15 mutations with constitutive nuclear factor-kappaB activation: common genetic etiology with Blau syndrome. *Blood* 105: 1195 – 1197

Kimura T, Watanabe E, Sakamoto T, Takasu O, Ikeda T, Ikeda K, Kotani J, Kitamura N, Sadahiro T, Tateishi Y *et al* (2014) Autophagy-related IRGM polymorphism is associated with mortality of patients with severe sepsis. *PLoS ONE* 9: e91522

Kumar S, Jain A, Choi SW, da Silva GPD, Allers L, Mudd MH, Peters RS, Anonsen JH, Rusten TE, Lazarou M *et al* (2020) Mammalian Atg8 proteins and the autophagy factor IRGM control mTOR and TFEB at a regulatory node critical for responses to pathogens. *Nat Cell Biol* 22: 973 – 985

Kumar S, Jain A, Farzam F, Jia J, Gu Y, Choi SW, Mudd MH, Claude-Taupin A, Wester MJ, Lidke KA *et al* (2018) Mechanism of Stx17 recruitment to autophagosomes via IRGM and mammalian Atg8 proteins. *J Cell Biol* 217: 997 – 1013

Laroui H, Yan Y, Narui Y, Ingersoll SA, Ayyadurai S, Charania MA, Zhou F, Wang B, Salaita K, Sitaraman SV *et al* (2011) L-ala-gamma-D-Glu-meso-

 © 2022 The Authors

diaminopimelic acid (DAP) interacts directly with leucine-rich region domain of nucleotide-binding oligomerization domain 1, increasing phosphorylation activity of receptor-interacting serine/threonine-protein kinase 2 and its interaction with nucleotide-binding oligomerization domain 1. *J Biol Chem* 286: 31003 – 31013

Lin YC, Chang PF, Lin HF, Liu K, Chang MH, Ni YH (2016) Variants in the autophagy-related gene IRGM confer susceptibility to non-alcoholic fatty liver disease by modulating lipophagy. *J Hepatol* 65: 1209 – 1216

Liu B, Gulati AS, Cantillana V, Henry SC, Schmidt EA, Daniell X, Grossniklaus E, Schoenborn AA, Sartor RB, Taylor GA (2013) Irgm1-deficient mice exhibit Paneth cell abnormalities and increased susceptibility to acute intestinal inflammation. *Am J Physiol Gastrointest Liver Physiol* 305: G573 – G584

Liu T, Tang Q, Liu K, Xie W, Liu X, Wang H, Wang RF, Cui J (2016) TRIM11 suppresses AIM2 inflammasome by degrading AIM2 via p62-dependent selective autophagy. *Cell Rep* 16: 1988 – 2002

Lopez-Montero N, Enninga J (2018) Diverted recycling-*shigella* subversion of Rabs. *Small GTPases* 9: 365 – 374

Love MI, Huber W, Anders S (2014) Moderated estimation of fold change and dispersion for RNA-seq data with DESeq2. *Genome Biol* 15: 550

Lu A, Li Y, Schmidt FI, Yin Q, Chen S, Fu TM, Tong AB, Ploegh HL, Mao Y, Wu H (2016) Molecular basis of caspase-1 polymerization and its inhibition by a new capping mechanism. *Nat Struct Mol Biol* 23: 416 – 425

Lu A, Magupalli VG, Ruan J, Yin Q, Atianand MK, Vos MR, Schroder GF, Fitzgerald KA, Wu H, Egelman EH (2014) Unified polymerization mechanism for the assembly of ASC-dependent inflammasomes. *Cell* 156: 1193 – 1206

Lu XC, Tao Y, Wu C, Zhao PL, Li K, Zheng JY, Li LX (2013) Association between variants of the autophagy related gene—IRGM and susceptibility to Crohn's disease and ulcerative colitis: a meta-analysis. *PLoS ONE* 8: e80602

Massey DC, Parkes M (2007) Genome-wide association scanning highlights two autophagy genes, ATG16L1 and IRGM, as being significantly associated with Crohn's disease. *Autophagy* 3: 649 – 651

Mehto S, Chauhan S, Jena KK, Chauhan NR, Nath P, Sahu R, Dhar K, Das SK, Chauhan S (2019a) IRGM restrains NLRP3 inflammasome activation by mediating its SQSTM1/p62-dependent selective autophagy. *Autophagy* 15: 1645 – 1647

Mehto S, Jena KK, Nath P, Chauhan S, Kolapalli SP, Das SK, Sahoo PK, Jain A, Taylor GA, Chauhan S (2019b) The Crohn's disease risk factor IRGM limits NLRP3 inflammasome activation by impeding its assembly and by mediating its selective autophagy. *Mol Cell* 73: e427

Miceli-Richard C, Lesage S, Rybojad M, Prieur AM, Manouvrier-Hanu S, Hafner R, Chamaillard M, Zouali H, Thomas G, Hugot JP (2001) CARD15 mutations in Blau syndrome. *Nat Genet* 29: 19 – 20

Nath P, Jena KK, Mehto S, Chauhan NR, Sahu R, Dhar K, Srinivas K, Chauhan S, Chauhan S (2021) IRGM links autoimmunity to autophagy. *Autophagy* 17: 578 – 580

Park HH (2019) Caspase recruitment domains for protein interactions in cellular signaling (review). *Int J Mol Med* 43: 1119 – 1127

Parkes M, Barrett JC, Prescott NJ, Tremelling M, Anderson CA, Fisher SA, Roberts RG, Nimmo ER, Cummings FR, Soars D *et al* (2007) Sequence variants in the autophagy gene IRGM and multiple other replicating loci contribute to Crohn's disease susceptibility. *Nat Genet* 39: 830 – 832

Pellegrini E, Desfosses A, Wallmann A, Schulze WM, Rehbein K, Mas P, Signor L, Gaudon S, Zenkeviciute G, Hons M *et al* (2018) RIP2 filament formation is required for NOD2 dependent NF-kappaB signalling. *Nat Commun* 9: 4043

Prabakaran T, Bodda C, Krapp C, Zhang BC, Christensen MH, Sun C, Reinert L, Cai Y, Jensen SB, Skouboe MK *et al* (2018) Attenuation of cGAS-STING signaling is mediated by a p62/SQSTM1-dependent autophagy pathway activated by TBK1. *EMBO J* 37: e97858

Ray K, Bobard A, Danckaert A, Paz-Haftel I, Clair C, Ehsani S, Tang C, Sansonetti P, Tran GV, Enninga J (2010) Tracking the dynamic interplay between bacterial and host factors during pathogen-induced vacuole rupture in real time. *Cell Microbiol* 12: 545 – 556

Shaid S, Brandts CH, Serve H, Dikic I (2013) Ubiquitination and selective autophagy. *Cell Death Differ* 20: 21 – 30

Shi CS, Shenderov K, Huang NN, Kabat J, Abu-Asab M, Fitzgerald KA, Sher A, Kehrl JH (2012) Activation of autophagy by inflammatory signals limits IL-1beta production by targeting ubiquitinated inflammasomes for destruction. *Nat Immunol* 13: 255 – 263

Singh SB, Davis AS, Taylor GA, Deretic V (2006) Human IRGM induces autophagy to eliminate intracellular mycobacteria. *Science* 313: 1438 – 1441

Singh SB, Ornatowski W, Vergne I, Naylor J, Delgado M, Roberts E, Ponpuak M, Master S, Pilli M, White E *et al* (2010) Human IRGM regulates autophagy and cell-autonomous immunity functions through mitochondria. *Nat Cell Biol* 12: 1154 – 1165

Tanabe T, Chamaillard M, Ogura Y, Zhu L, Qiu S, Masumoto J, Ghosh P, Moran A, Predergast MM, Tromp G *et al* (2004) Regulatory regions and critical residues of NOD2 involved in muramyl dipeptide recognition. *EMBO J* 23: 1587 – 1597

Taniguchi K, Karin M (2018) NF-kappaB, inflammation, immunity and cancer: coming of age. *Nat Rev Immunol* 18: 309 – 324

Tripathi S, Pohl MO, Zhou Y, Rodriguez-Frandsen A, Wang G, Stein DA, Moulton HM, DeJesus P, Che J, Mulder LC *et al* (2015) Meta- and orthogonal integration of influenza "OMICs" data defines a role for UBR4 in virus budding. *Cell Host Microbe* 18: 723 – 735

Wu B, Hur S (2015) How RIG-I like receptors activate MAVS. *Curr Opin Virol* 12: 91 – 98

Wu B, Peisley A, Tetrault D, Li Z, Egelman EH, Magor KE, Walz T, Penczek PA, Hur S (2014) Molecular imprinting as a signal-activation mechanism of the viral RNA sensor RIG-I. *Mol Cell* 55: 511 – 523

Wu H, Fuxreiter M (2016) The structure and dynamics of higher-order assemblies: amyloids, signalosomes, and granules. *Cell* 165: 1055 – 1066

Xia Q, Wang M, Yang X, Li X, Zhang X, Xu S, Shuai Z, Xu J, Fan D, Ding C *et al* (2017) Autophagy-related IRGM genes confer susceptibility to ankylosing spondylitis in a Chinese female population: a case-control study. *Genes Immun* 18: 42 – 47

Xie T, Peng W, Yan C, Wu J, Gong X, Shi Y (2013) Structural insights into RIP3-mediated necroptotic signaling. *Cell Rep* 5: 70 – 78

Xu H, He X, Zheng H, Huang LJ, Hou F, Yu Z, de la Cruz MJ, Borkowski B, Zhang X, Chen ZJ *et al* (2014) Structural basis for the prion-like MAVS filaments in antiviral innate immunity. *Elife* 3: e01489

Yang JY, Lee SN, Chang SY, Ko HJ, Ryu S, Kweon MN (2014) A mouse model of shigellosis by intraperitoneal infection. *J Infect Dis* 209: 203 – 215

Yao QM, Zhu YF, Wang W, Song ZY, Shao XQ, Li L, Song RH, An XF, Qin Q, Li Q *et al* (2018) Polymorphisms in autophagy-related gene IRGM are associated with susceptibility to autoimmune thyroid diseases. *Biomed Res Int* 2018: 7959707

License: This is an open access article under the terms of the Creative Commons Attribution License, which permits use, distribution and reproduction in any medium, provided the original work is properly cited.

Expanded View Figures

Figure EV1. NODs, RIPK2, and RIPosomes are the target of selective autophagy.

A–C Western blot analysis with the cell lysates of HT-29 cells treated with cycloheximide (100 μg/ml) alone or in combination with Bafilomycin A1 (300 nM) or MG132 (20 μM) for different time points as indicated.

D Western blot analysis of IP experiments performed with lysates of HEK293T cells transiently transfected with Flag-RIPK2 and HA-K63-Ubiquitin [variants of ubiquitin that can only be ubiquitinated at lysine 63 (K63)] and infected with *S. flexneri*, (MOI: 1:25, 6 h). IP was performed with Flag antibody and Western blotting was performed with indicated antibodies.

E Representative confocal images of THP-1 cells infected with RFP expressing *S. flexneri*, (MOI: 1:25, 8 h) and immunostained with anti-RIPK2 and anti-FK2 antibodies. Line profile: co-localization analysis using line intensity profiles. Scale bar, 5 μm.

F Left panel, representative high-content microscopy images (digitally zoomed) of RIPosomes in HEK293T cells. The HEK293T cells were transfected with GFP RIPK2 (100 ng/well of 96-well plates) for 4 h followed by treatment with rapamycin (500 nM, 4 h), or rapamycin (500 nM, 4 h) and PYR-41 (5 μM) for 5 h. Right panel, the graph depicts an average number of RIPosomes/cell. About 15,000 cells were plated per well and RIPosomes were screened in 35 fields per well. Mean ± SD, $n = 3$ (biological replicates), $^{***}P < 0.0005$, $^{****}P < 0.00005$, ordinary one-way ANOVA (Tukey's multiple comparisons test).

G Left panels, representative high-content microscopy images (digitally zoomed) of control or p62 knockdown THP-1 cells infected with *S. flexneri* (6 hpi). About 50,000 cells were plated per well in a 96-well plate and RIPosomes were screened in 35 fields per well. Right panel, the graph depicts the average number of RIPosomes/cell, which is calculated from three biological replicates. Mean ± SD. $^{***}P < 0.0005$, Student's unpaired *t*-test.

H Representative confocal images of HEK293T cells transfected with GFP-RIPK2 (6 h) and immunostained with p62 and LC3B antibody. Zoom panel is a digital magnification. Line profile: co-localization analysis using line intensity profile. Scale bar, 5 μm.

I Representative confocal images of THP-1 cells infected with RFP expressing *S. flexneri*, (MOI 1:25) and immunostained with RIPK2 and LC3B antibodies. DNA stained with DAPI. Scale bar, 8 μm. Line profile: co-localization analysis using line intensity profile. Zoom panels are digital magnifications.

J, K Representative confocal images of HEK293T cells transfected with (I) GFP-RIPK2 and Myc-ULK1 (6 h) and (J) GFP-RIPK2 and Flag-ATG16L (6 h). Zoom panels are digital magnification. Line profile: co-localization analysis using line intensity profile. Scale bar, 5 μm.

L Western blot analysis with the cell lysate of PMA-treated THP-1 cells infected with *S. flexneri*, (MOI: 1:25) with or with Bafilomycin A1 (300 nM) for different time points as indicated.

M The qRT–PCR analysis with total RNA isolated from the uninfected and *S. flexneri* (MOI 1:2.5, 4 h) infected control or p62 knockdown or p62 and RIPK2 double knockdown THP-1 cells. Mean ± SD, $n = 4$ (biological replicates), $^{***}P < 0.0005$ and $^{****}P < 0.00005$, ordinary one-way ANOVA (Tukey's multiple comparisons test).

Source data are available online for this figure.

 © 2022 The Authors

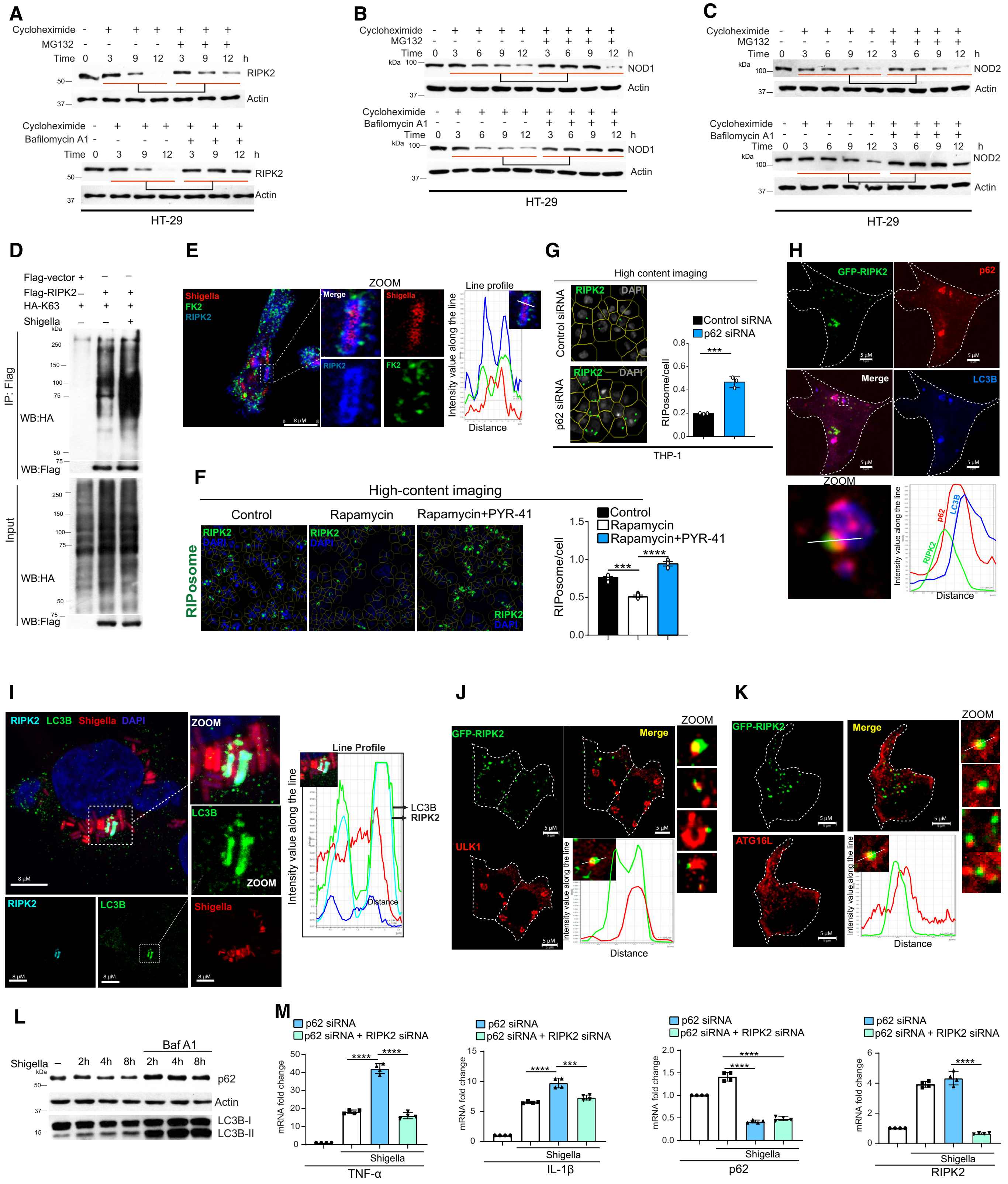
A
Cycloheximide
MG132
Time
RIPK2
Actin
Bafilomycin A1
HT-29
B
NOD1
C
NOD2
D
Flag-vector
Flag-RIPK2
HA-K63
Shigella
IP: Flag
WB:HA
WB:Flag
Input
E
Shigella
FK2
RIPK2
ZOOM
Merge
Line profile
Intensity value along the line
Distance
F
High-content imaging
Control
Rapamycin
Rapamycin+PYR-41
RIPosome
RIPK2
DAPI
RIPosome/cell
G
High content imaging
Control siRNA
p62 siRNA
THP-1
H
GFP-RIPK2
p62
Merge
LC3B
ZOOM
I
RIPK2 LC3B Shigella DAPI
Line Profile
J
GFP-RIPK2
ULK1
K
ATG16L
L
Baf A1
LC3B-I
LC3B-II
M
p62 siRNA
p62 siRNA + RIPK2 siRNA
mRNA fold change
TNF-α
IL-1β

Figure EV1.

Figure EV2. IRGM mediates the degradation of NODs, RIPK2, and RIPosomes.

A The cell lysates of *S. flexneri*-infected IRGM$^{+/+}$ and IRGM$^{+/-}$ HT-29 cells were subjected to Western blot analysis with indicated antibodies. Densitometric analysis was performed using Image J software. FC, fold change.

B Western blot analysis with the cell lysates of control and IRGM siRNA transfected doxycycline (1 μg/ml) induced HeLa GFP-RIPK2 stable cells infected with *S. flexneri* (MOI 1:25, 8 h). Densitometric analysis was performed using Image J software. FC, fold change.

C, D Western blot analysis with the cell lysates of HEK293T transfected with indicated plasmids. Densitometric analysis was performed using Image J software. FC, fold change.

E Western blot analysis with the cell lysate of HEK293T cells transfected Myc-NOD2 and GFP or increasing concentration of GFP-IRGM (2, 4, and 6 μg) plasmids for 12 h.

F Left panels, representative high-content microscopy images (Green masks, software algorithms-defined cell boundaries, digitally zoomed images) of HEK293T cells transfected with GFP-RIPK2 or GFP-RIPK2 and Flag-IRGM. About 17,000 cells were plated per well in a 96-well plate and RIPosomes were screened in 35 fields per well. Right panel, the graph depicts the average number of RIPosomes/cell, which is calculated from four biological replicates, Mean ± SD. ****$P < 0.00005$, Student's unpaired *t*-test.

G Western blot analysis of soluble and insoluble fractions of HEK293T cells transfected with indicated plasmids for 12 h.

H Representative confocal image of HEK293T cells transfected with GFP-RIPK2^{CARD} or GFP-RIPK2^{CARD} and mCherry-IRGM. Scale bar, 5 μm.

I Right panels, representative high-content microscopy images (Yellow masks, software algorithms-defined cell boundaries, digitally zoomed images) of HEK293T cells transfected with mCherry-RIPK2$^{CARD.}$ Alone or with Flag-IRGM. About 17,000 cells were plated per well in a 96-well plate and RIPK2^{CARD} spots were screened in 35 fields per well. Left panel, the graph depicts an average number of RIPK2^{CARD} spots/cell, which is calculated from three biological replicates, Mean ± SD. ****$P < 0.00005$, Student's unpaired *t*-test.

J Western blot analysis with the cell lysates of HEK293T transfected with indicated plasmids for 12 h.

K Representative fluorescence microscopy images of HEK293T cells transfected with GFP-RIPK2 ^{CARD} alone or with Flag-IRGM or with catalytic mutant Flag-IRGM (S47N) for 12 h. Scale bar, 150 μm.

L Assessment of NF-κB-induced SEAP (secreted embryonic alkaline phosphatase) activity in the cell culture supernatant of HEK-Blue™ hNOD2 cells (InvivoGen) transfected with plasmids as indicated and treated with L18-MDP (100 ng/ml, 24 h) as indicated. Three technical replicates, Mean ± SD. **$P < 0.005$, ****$P < 0.00005$, Student's unpaired *t*-test.

Source data are available online for this figure.

© 2022 The Authors

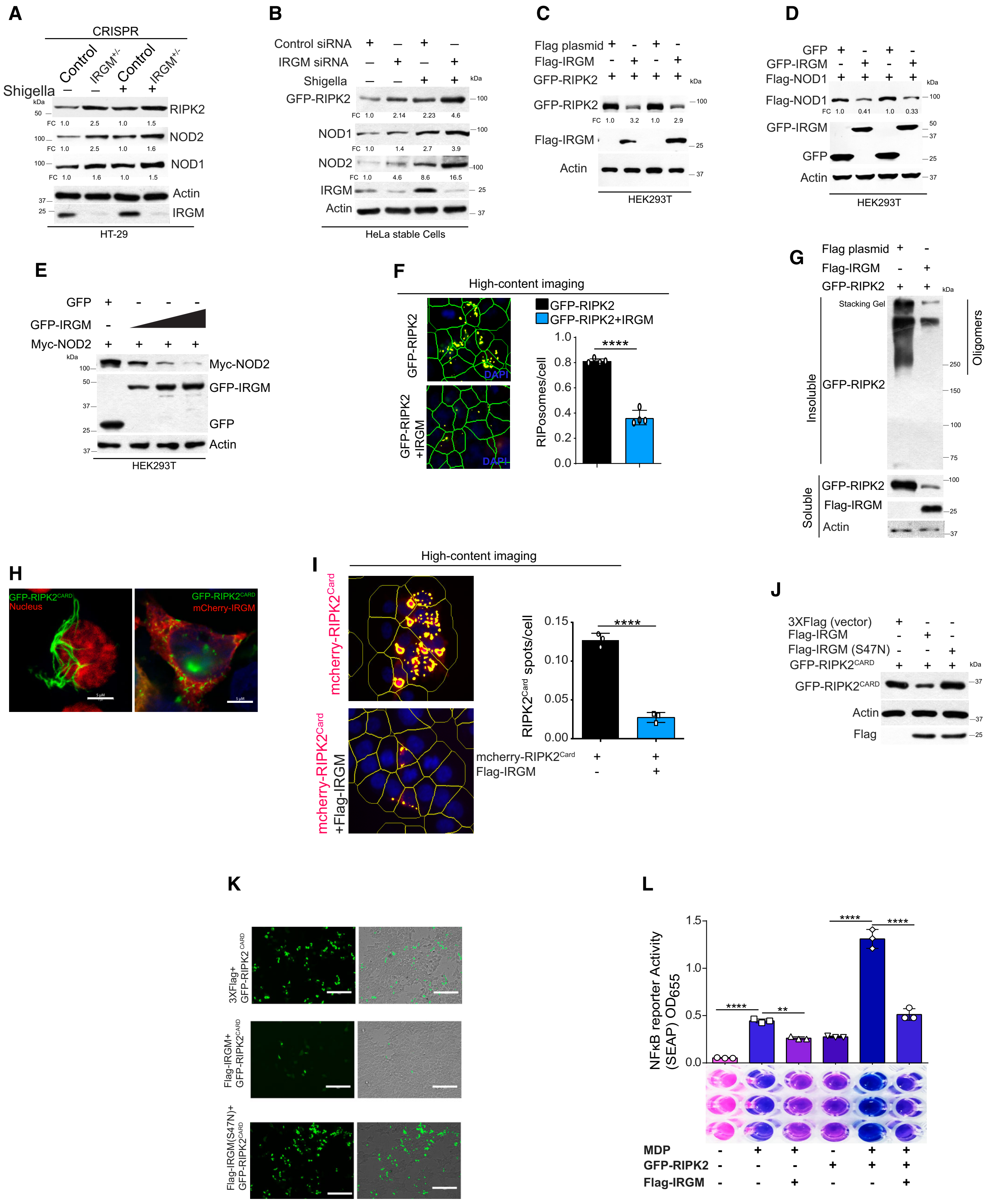
A
CRISPR
Control
IRGM+/-
Control
IRGM+/-
Shigella
RIPK2
NOD2
NOD1
Actin
IRGM
HT-29
B
Control siRNA
IRGM siRNA
Shigella
GFP-RIPK2
NOD1
NOD2
IRGM
Actin
HeLa stable Cells
C
Flag plasmid
Flag-IRGM
GFP-RIPK2
GFP-RIPK2
Flag-IRGM
Actin
HEK293T
D
GFP
GFP-IRGM
Flag-NOD1
Flag-NOD1
GFP-IRGM
GFP
Actin
HEK293T
E
GFP
GFP-IRGM
Myc-NOD2
Myc-NOD2
GFP-IRGM
GFP
Actin
HEK293T
F
High-content imaging
GFP-RIPK2
GFP-RIPK2 +IRGM
GFP-RIPK2
GFP-RIPK2+IRGM
RIPosomes/cell
G
Flag plasmid
Flag-IRGM
GFP-RIPK2
Stacking Gel
Oligomers
Insoluble
GFP-RIPK2
Soluble
GFP-RIPK2
Flag-IRGM
Actin
H
GFP-RIPK2CARD
Nucleus
GFP-RIPK2CARD
mCherry-IRGM
I
High-content imaging
mcherry-RIPK2Card
mcherry-RIPK2Card +Flag-IRGM
RIPK2Card spots/cell
mcherry-RIPK2Card
Flag-IRGM
J
3XFlag (vector)
Flag-IRGM
Flag-IRGM (S47N)
GFP-RIPK2CARD
GFP-RIPK2CARD
Actin
Flag
K
3XFlag+ GFP-RIPK2CARD
Flag-IRGM+ GFP-RIPK2CARD
Flag-IRGM(S47N)+ GFP-RIPK2CARD
L
NFκB reporter Activity (SEAP) OD655
MDP
GFP-RIPK2
Flag-IRGM

Figure EV2.

Figure EV3. IRGM and p62 cooperatively execute selective autophagy of NODs, RIPK2, and RIPosomes.

A, B Western blot analysis with cell lysates of control cells and ATG5 knockdown THP-1 cells transiently transfected with GFP-IRGM plasmids and treated with (A) MDP (40 μg/ml, 4 h) or (B) iE-DAP (40 μg/ml, 4 h).

C–E The control and ATG5 knockdown HEK293T cells were transfected with indicated plasmids and cell lysates were subjected to immunoblot analysis.

F Left panels, representative high-content microscopy images (digitally zoomed) of cells that were knockdown for indicated genes and transfected with plasmids as indicated. In the last panel, GFP-RIPK2 and mcherry-IRGM transfected cells were treated with Bafilomycin A1 (300 nM, 5 h). Right panel, the graph depicts an average number of RIPosomes/cell. About 50,000 cells were plated per well and RIPosomes were screened in 35 fields per well. Mean ± SD, $n = 6$ (biological replicates), $^{****}P < 0.00005$, ordinary one-way ANOVA (Tukey's multiple comparisons test).

G Representative confocal images of cells transfected with GFP-RIPK2, and mCherry-IRGM and immunostained with p62. Line profile: co-localization analysis using line intensity profiles. Scale bar, 5.5 μm.

H Sequential immunoprecipitation assay from the lysate where the HEK293T transiently transfected with Flag-RIPK2 (or Flag-vector control), HA-p62, and, GFP-IRGM for 12 h. The first immunoprecipitation was performed with Flag antibody followed by elution with flag peptide. The flag peptide eluted samples were further subjected to a second IP with anti-GFP (for IRGM) and probed with indicated antibodies.

I–K The control and p62 knockdown HEK293T cells were transfected with indicated plasmids and cell lysates were subjected to Western blot analysis with indicated antibodies.

L, M Western blot analysis with cell lysates of control cells and p62 knockdown THP-1 cells transiently transfected with GFP-IRGM plasmids treated with (L) MDP (40 μg/ml, 4 h) or (M) iE-DAP (40 μg/ml, 4 h).

N Immunoprecipitation analysis of the interaction between endogenous RIPK2 and endogenous p62 in the lysate of *S. flexneri* (MOI 1:25, 6 h) infected control and IRGM knockdown THP-1 cells. IgG, IgG heavy chain.

O–Q Co-IP analysis with the lysate of HEK293T cells transiently transfected with (O) Flag-NOD1 and HA-p62, (P) Flag-NOD2 and HA-p62, and, (Q) GFP-RIPK2 and HA-p62 in the presence and absence of IRGM (GFP or Flag) or vector controls.

R Pictorial representation of data. We found that IRGM and p62 coordinate selective autophagy of NODs, RIPK2, and RIPosomes.

Source data are available online for this figure.

 © 2022 The Authors

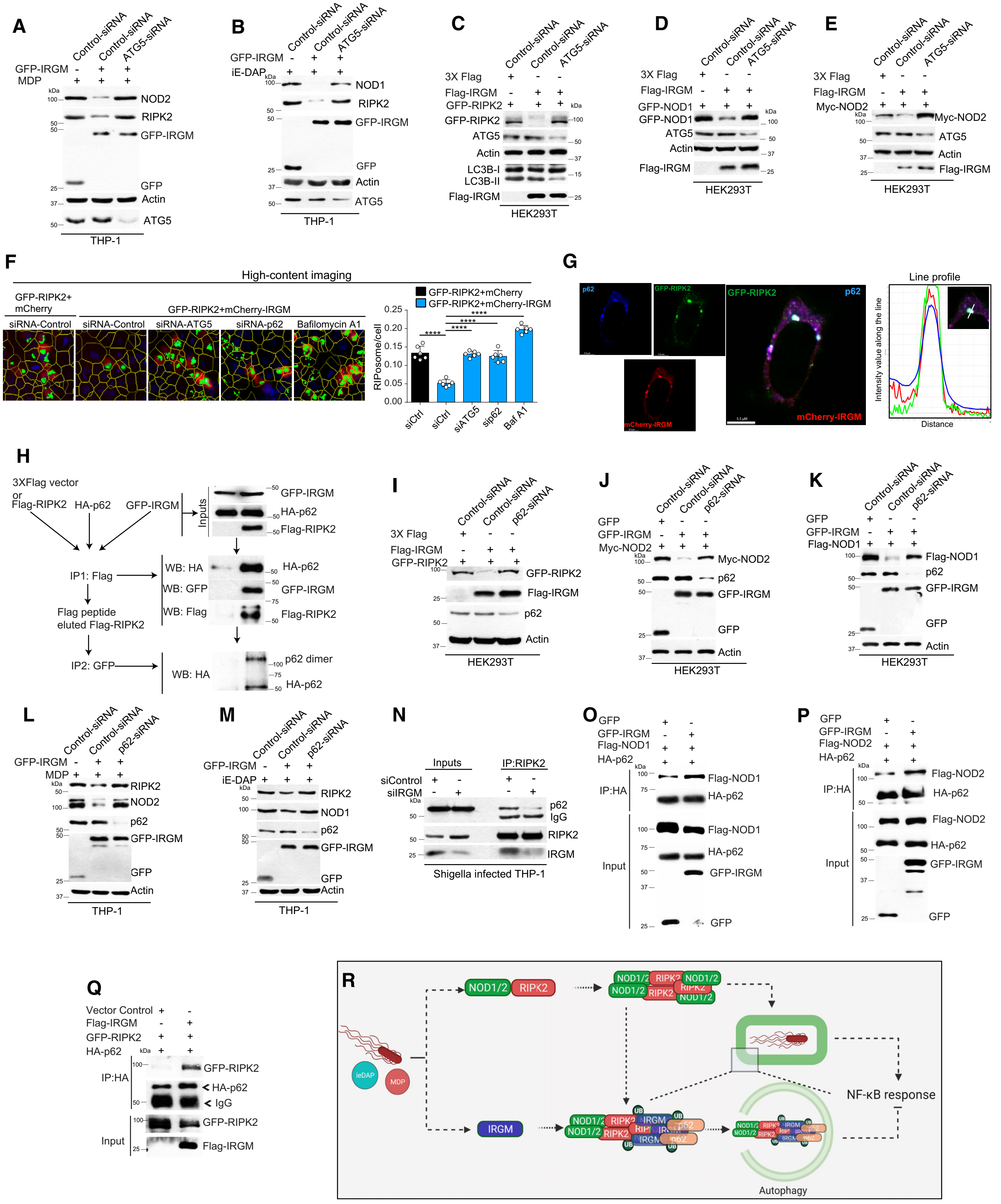

Figure EV3.

© 2022 The Authors

Figure EV4. IRGM negatively regulates bacterial infection-induced programs of pro-inflammatory response.

A Heatmap of the gene clusters related to top biological pathways overrepresented in the Reactome pathway analysis. Reactome pathway analysis was performed with the set of differentially regulated genes (1.5-fold, $P < 0.05$, Wald Chi-Squared test, $n = 3$, biological replicates) in uninfected and *Salmonella*-infected control and IRGM shRNA knockdown HT-29 cells. The graph below the heat map shows box plot with median obtained from data scaled to Z-score.

B Left panel, heatmap depicts the gene set belonging to the “Cytokine signaling in immune system” pathway that is one of the top upregulated pathways in Reactome pathway analysis. Reactome pathway analysis was performed with the set of genes significantly upregulated (1.5-fold, $P < 0.05$, Wald test, $n = 3$) in *Salmonella typhimurium* infected IRGM shRNA knockdown HT-29 cells compared with control shRNA cells. Right panel, the bar graph represents the top 10 biological pathways upregulated in GO-based Reactome pathways analysis using a set of genes that are significantly (1.5-fold, $P < 0.05$, Wald Chi-Squared test, $n = 3$ in each group) induced in *Salmonella*-infected IRGM shRNA HT-29 as compared to *Salmonella*-infected control cells.

C Heatmap depicts the significantly upregulated (1.5-fold, $P < 0.05$, Wald Chi-Squared test, $n = 3$) NF-κB regulated cytokine and chemokine genes in *Salmonella*-infected IRGM shRNA HT-29 cells compared with *Salmonella*-infected control shRNA cells.

D A qRT–PCR validation of significantly upregulated cytokines and chemokines in RNA-Seq data with total RNA isolated from uninfected and *Salmonella*-infected control and IRGM shRNA HT-29 cells. Mean ± SD, $n = 3$ (Biological replicates). $^{**}P < 0.05$, $^{***}P < 0.005$, $^{****}P < 0.0005$, Student’s unpaired *t*-test.

E Left panel, Metascape pathway analysis with the set of genes that are significantly upregulated in $IRGM^{+/-}$ cells (compared with control; 1.5-fold, $P < 0.05$, Wald Chi-Squared test, $n = 3$) and at the same time were significantly rescued by RIPK2 depletion in $IRGM^{+/-}$ HT-29 cells ($P < 0.05$, Wald Chi-Squared test, 0.8, $n = 3$). Right panel, heatmap depicts the gene set belonging to “Hallmark TNFα signaling via NF-κB” pathway that is the top upregulated pathway in Metascape analysis. The graph below the heat map shows box plot with median obtained from data scaled to Z-score.

F Bar graph depicts top biological pathway upregulated in GO-based metascape pathway analysis with genes significantly induced (1.5-fold, P 0.05, $n = 3$) in *Shigella*-infected $IRGM^{+/-}$ HT-29 cells.

G TRRUST analysis (database for the study of the transcriptional regulation involved in human diseases) with the set of genes that are significantly upregulated in $IRGM^{+/-}$ cells (compared with control; 1.5-fold, $P < 0.05$, Wald Chi-Squared test, $n = 3$, biological replicates) and at the same time were significantly rescued by RIPK2 depletion in $IRGM^{+/-}$ HT-29 cells ($P < 0.05$, Wald Chi-Squared test, 0.8, $n = 3$).

Source data are available online for this figure.

© 2022 The Authors

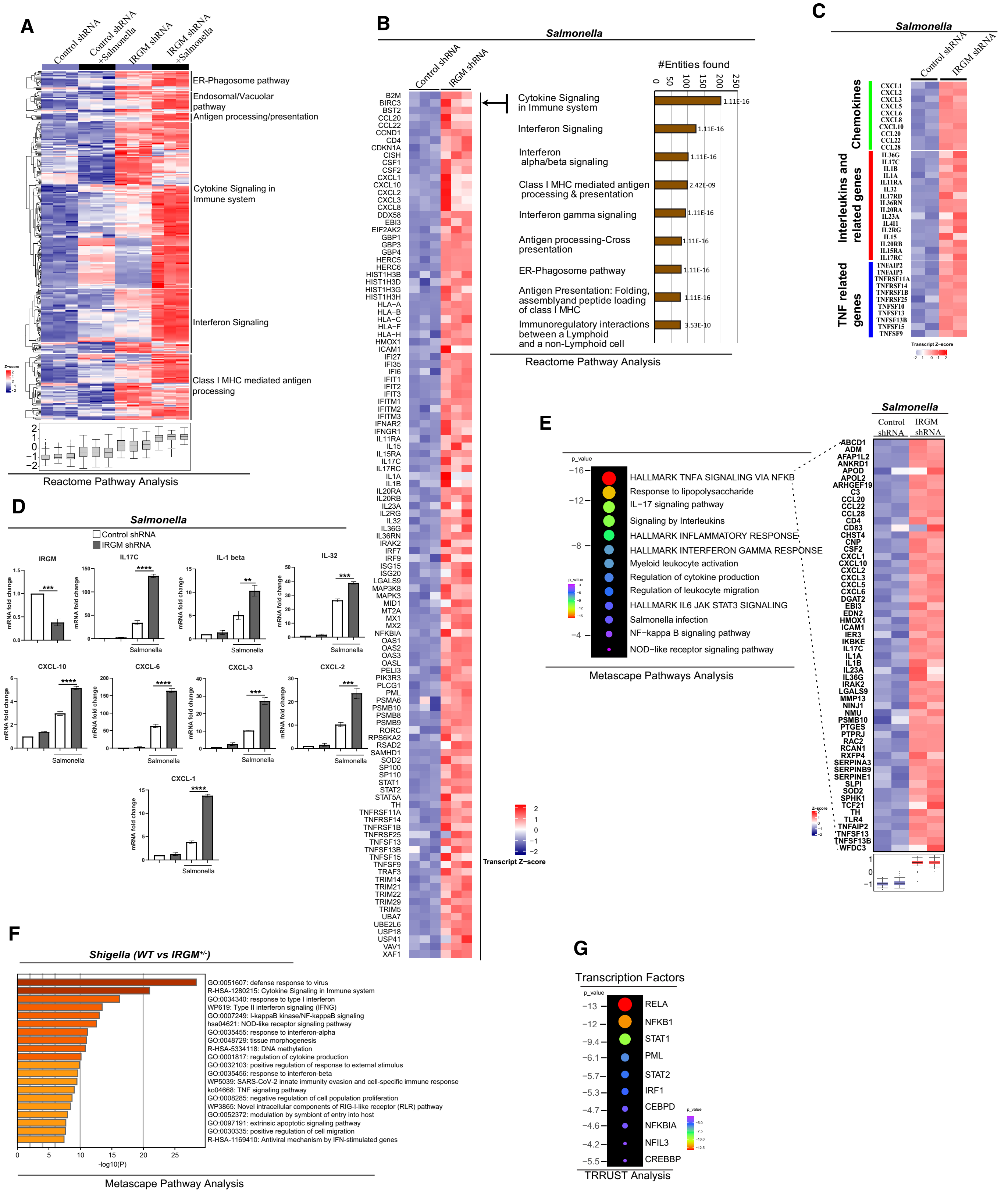
A
Control shRNA
Control shRNA +Salmonella
IRGM shRNA
IRGM shRNA +Salmonella
ER-Phagosome pathway
Endosomal/Vacuolar pathway
Antigen processing/presentation
Cytokine Signaling in Immune system
Interferon Signaling
Class I MHC mediated antigen processing
Reactome Pathway Analysis
B
Salmonella
Control shRNA
IRGM shRNA
#Entities found
Cytokine Signaling in Immune system
Interferon Signaling
Interferon alpha/beta signaling
Class I MHC mediated antigen processing & presentation
Interferon gamma signaling
Antigen processing-Cross presentation
ER-Phagosome pathway
Antigen Presentation: Folding, assemblyand peptide loading of class I MHC
Immunoregulatory interactions between a Lymphoid and a non-Lymphoid cell
Reactome Pathway Analysis
Transcript Z−score
C
Salmonella
Control shRNA
IRGM shRNA
Chemokines
Interleukins and related genes
TNF related genes
D
Salmonella
Control shRNA
IRGM shRNA
IRGM
IL17C
IL-1 beta
IL-32
CXCL-10
CXCL-6
CXCL-3
CXCL-2
CXCL-1
mRNA fold change
Salmonella
E
p_value
HALLMARK TNFA SIGNALING VIA NFKB
Response to lipopolysaccharide
IL−17 signaling pathway
Signaling by Interleukins
HALLMARK INFLAMMATORY RESPONSE
HALLMARK INTERFERON GAMMA RESPONSE
Myeloid leukocyte activation
Regulation of cytokine production
Regulation of leukocyte migration
HALLMARK IL6 JAK STAT3 SIGNALING
Salmonella infection
NF−kappa B signaling pathway
NOD−like receptor signaling pathway
Metascape Pathways Analysis
Salmonella
Control shRNA
IRGM shRNA
F
Shigella (WT vs IRGM+/-)
GO:0051607: defense response to virus
R-HSA-1280215: Cytokine Signaling in Immune system
GO:0034340: response to type I interferon
WP619: Type II interferon signaling (IFNG)
GO:0007249: I-kappaB kinase/NF-kappaB signaling
hsa04621: NOD-like receptor signaling pathway
GO:0035455: response to interferon-alpha
GO:0048729: tissue morphogenesis
R-HSA-5334118: DNA methylation
GO:0001817: regulation of cytokine production
GO:0032103: positive regulation of response to external stimulus
GO:0035456: response to interferon-beta
WP5039: SARS-CoV-2 innate immunity evasion and cell-specific immune response
ko04668: TNF signaling pathway
GO:0008285: negative regulation of cell population proliferation
WP3865: Novel intracellular components of RIG-I-like receptor (RLR) pathway
GO:0052372: modulation by symbiont of entry into host
GO:0097191: extrinsic apoptotic signaling pathway
GO:0030335: positive regulation of cell migration
R-HSA-1169410: Antiviral mechanism by IFN-stimulated genes
-log10(P)
Metascape Pathway Analysis
G
Transcription Factors
p_value
RELA
NFKB1
STAT1
PML
STAT2
IRF1
CEBPD
NFKBIA
NFIL3
CREBBP
TRRUST Analysis

Figure EV4.

Figure EV5. RIPK2 inhibition ameliorates shigellosis and DSS-induced gut inflammation in *Irgm1* knockout mice.

A Right panel, representative picture of fecal samples collected from *S. flexneri*-infected *Irgm1*$^{+/+}$ or *Irgm1*$^{-/-}$ or GSK583-treated *Irgm1*$^{-/-}$ mice. Left panel, the graph depicts fecal pathology scores based on stool consistency and color *of S. flexneri*-infected and GSK583-treated C57BL/6 mice. (n = 6 mice in each group, Mean ± SD. $^{**}P < 0.005$, Student's unpaired t-test).

B The graph depicts the total stool scores (stool consistency + blood) of DSS-treated *Irgm1*$^{+/+}$ or *Irgm1*$^{-/-}$ or GSK583-treated *Irgm1*$^{-/-}$ mice.

C Right panel, representative picture of colons of DSS-treated *Irgm1*$^{+/+}$ or *Irgm1*$^{-/-}$ or GSK583-treated *Irgm1*$^{-/-}$ mice; Left panel, the graph depicts the average colon length of same. (n = 3 mice each group, Mean ± SD. $^{*}P < 0.05$, $^{**}P < 0.005$, Student's unpaired t-test).

D Representative microscopic images of hematoxylin–eosin (H&E) staining of colon tissue of DSS-treated *Irgm1*$^{+/+}$ or *Irgm1*$^{-/-}$ or GSK583-treated *Irgm1*$^{-/-}$ mice.

E Representative microscopic images of hematoxylin–eosin (H&E) staining of colon tissue of *S. flexneri*-infected *Irgm1*$^{+/+}$ or *Irgm1*$^{-/-}$ or GSK583-treated *Irgm1*$^{-/-}$ mice.

F The graph depicts the histopathology score (average pathological scores from HE staining based on hyperplasia, inflammatory cells infiltration, epithelial cell death, and loss of goblet cells) of *S. flexneri*-infected *Irgm1*$^{+/+}$ or *Irgm1*$^{-/-}$ or GSK583-treated *Irgm1*$^{-/-}$ mice. (n = 3 mice in each group, Mean ± SD, $^{**}P < 0.005$, Student's unpaired t-test).

G The qRT–PCR analysis for indicated genes with the total RNA isolated from the colons of DSS-treated *Irgm1*$^{+/+}$ or *Irgm1*$^{-/-}$ or GSK583-treated *Irgm1*$^{-/-}$ mice. (n = 3 mice in each group, Mean ± SD. $^{*}P < 0.05$. $^{**}P < 0.005$, $^{***}P < 0.0005$, $^{****}P < 0.00005$, ordinary one-way ANOVA (Tukey's multiple comparisons test).

Source data are available online for this figure.

 © 2022 The Authors

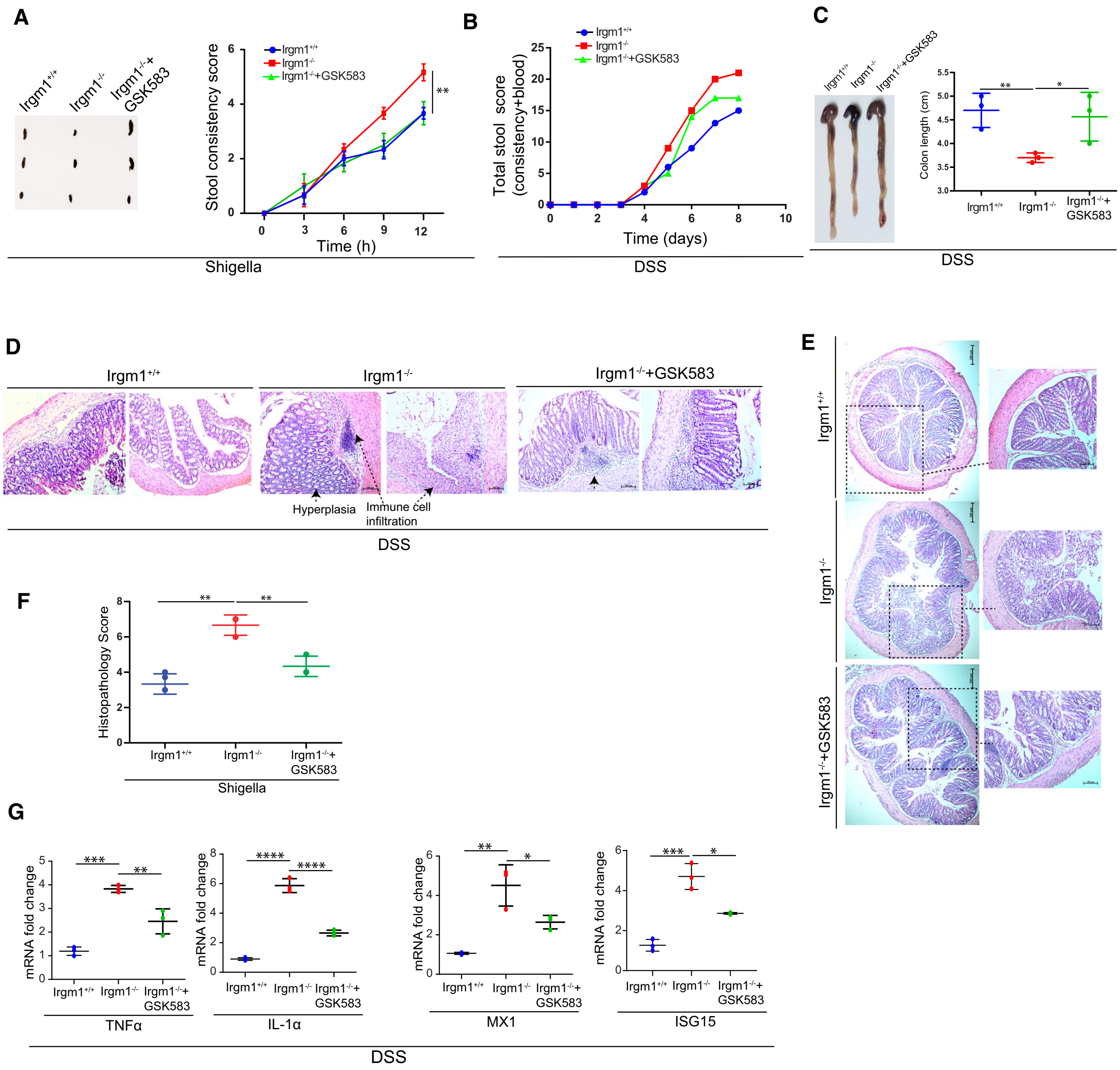
A
Irgm1+/+
Irgm1-/-
Irgm1-/-+ GSK583
Stool consistency score
Time (h)
Shigella
B
Total stool score (consistency+blood)
Time (days)
DSS
C
Colon length (cm)
DSS
D
Hyperplasia
Immune cell infiltration
DSS
E
F
Histopathology Score
Shigella
G
mRNA fold change
TNFα
IL-1α
MX1
ISG15
DSS

Figure EV5.

“Selective Autophagy of RIPosomes Maintains Innate Immune Homeostasis during Bacterial Infection”

Mehto et al

Table of content

Appendix Figure S1 to S5

Appendix Figure legends S1 to S5

Appendix Table S1

Appendix Figure S1. Bacterial infection induces RIPosome formation

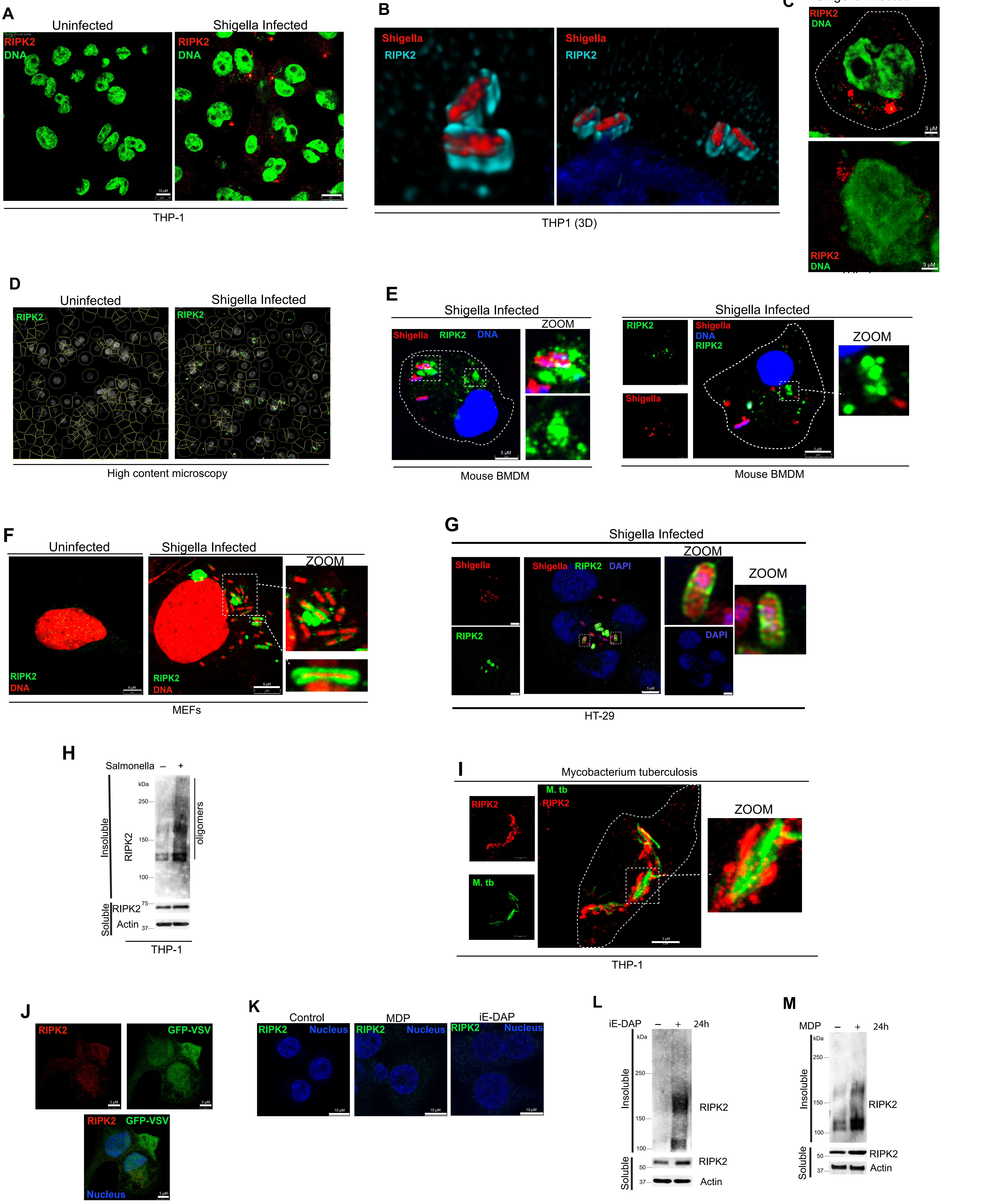

Appendix Fig S1. Bacterial infection induces RIPosomes formation

(A) Representative confocal images of uninfected and S. *flexneri* infected THP-1 cells (MOI 1:25, 8 hpi), Scale bar 10 µm.

(B) The digitally magnified three dimensional (3D) confocal images of RIPosomes recruited over RFP labelled S. *flexneri* in THP-1 cells (8hpi). DNA is stained with DAPI.

(C) Representative confocal images showing RIPosomes in S. *flexneri* infected THP-1 cells (MOI 1:25, 8 hpi).

(D) Representative high-content microscopy images of one full field (Yellow masks, software algorithms-defined cell boundaries) of RIPosomes in THP-1 cells infected with *S. flexneri* (MOI 1:25, 8 hpi).

(E-G) Representative confocal images of S. *flexneri* infected **(E)** mouse BMDM cells (Scale bar, 5 µm) or **(F)** MEFs cells (Scale bar, 3 µm) or **(G)** HT-29 cells (Scale bar, 8 µm). Zoom panels are digital magnification. DNA is stained with DAPI. DAPI is pseudocolored red in panel F for better contrast. In panel E and G, RFP-labelled S. *flexneri* was used for infection.

(H) Western blot analysis of soluble and insoluble fractions of THP-1 cell lysates, uninfected or infected with *Salmonella typhimurium* (4hpi).

(I) Representative confocal images of THP-1 cells infected with *M. tuberculosis* H37Rv (4 hpi). Zoom panels are digital magnification. Scale bar, 5 µm.

(J) Representative confocal images of RIPosomes in THP-1 cells infected with GFP VSV (MOI, 1:5, 8 hpi). DNA is stained with DAPI. Scale bar, 5 µm.

(K) Representative confocal images of RIPosomes in THP-1 cells treated with MDP (40 µg/ml) MDP or iE-DAP (40 µg/ml) for 24 h. DNA is stained with DAPI. Scale bar, 10 µm.

(L-M) Western blot analysis of soluble and insoluble fractions of THP-1 cells stimulated with **(L)** iE-DAP (40 µg/ml, 24 h) or**(M)** MDP (40 µg/ml, 24 h).

Appendix FigS2. NODs oligomerization is dependent on presence of RIPK2 oligomers

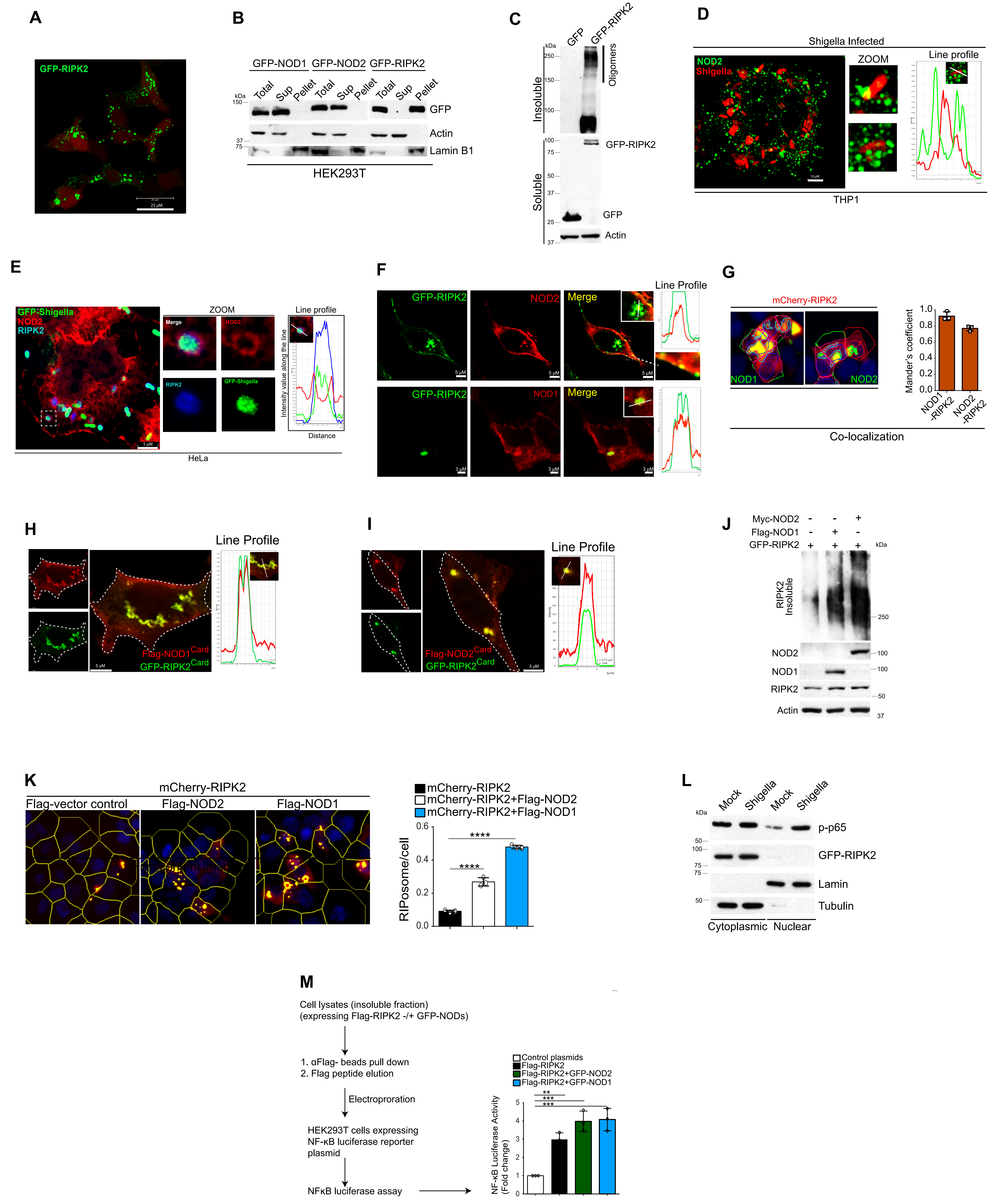

Appendix Fig S2. RIPK2 oligomers provide a platform for NODo-RIPosome formation.

(A) Representative confocal image of HEK293T transfected with GFP-RIPK2 plasmid (9 h). Scale bar, 25 μm.

(**B)** HEK293T cells transfected with GFP-NOD1 or GFP-NOD2 or GFP-RIPK2 were subjected to western blot analysis with total cellular extract (Total), soluble (Sup), and insoluble (pellet) fractions.

(C) Western blot analysis of soluble and insoluble fractions of HEK293T cells transfected with GFP-RIPK2 (9 h).

(D) Representative confocal images of THP-1 cells infected with RFP- labelled *S. flexneri* (8 hpi) and immuno-stained with NOD2 antibody. Zoom panels are digital magnification. Line profile: co-localization analysis using line intensity profile. Scale bar, 10 μm.

(E) Representative confocal images of HeLa cells transfected with myc-NOD2 for 4 h followed by infection with GFP-*S. flexneri* (MOI 1:25, 4 hpi). The cells were immuno-stained with c-Myc and RIPK2 antibodies. Zoom panels are digital magnification. Line profile: co-localization analysis using line intensity profile. Scale bar, 5 μm.

(F) Representative confocal images of HEK293T cells transfected with **(Upper panel)** GFP-RIPK2 and Flag-NOD2, Scale bar, 5 μm or **(Lower panel**) GFP-RIPK2 and Flag-NOD1. Line profile: co-localization analysis using line intensity profile. Scale bar, 3 μm.

(G) Right panel, representative high-content microscopy images (digitally zoomed; green masks for Flag-NOD1 or Flag-NOD2 and red masks for mCherry-RIPK2, blue mask for DAPI, cyan mask for co-localization, software algorithms-defined boundaries) of HEK293T cells transfected with mcherry-RIPK2 and GFP-NOD1 or GFP-NOD2 plasmids. About 17000 cells plated per well in a 96-well plate and NODo-RIPosomes were screened in 35 fields per well in 3 technical replicates. The graph depicts Mander's overlap (co-localization) coefficient. (n=3 biological replicates, Mean ± SD).

(H-I) Representative confocal images of HEK293T cells transfected with (**H)** GFP-RIPK2^{CARD} and Flag-NOD1^{CARD} or **(I)** GFP-RIPK2^{CARD} and Flag-NOD2^{CARD}. Line profile: co-localization analysis using line intensity profile. Scale bar, 5 μm.

(J) HEK293T cells transfected with GFP-RIPK2 and Flag-NOD1 or myc-NOD2 for 9 h. The triton X-100 soluble and insoluble fraction subjected to western blot analysis with indicated antibodies.

(K) Representative high-content microscopy image (yellow masks, software algorithms-defined cell boundaries) of RIPosomes in HEK293T cells transfected with mcherry-RIPK2 and Flag-NOD1 or Flag-NOD2 plasmid. About 17000 cells plated per well and were screened in 35 fields per well in 3 technical replicates. The graph depicts the average number of RIPosomes/cell. Mean ± SD. ****p < 0.00005, ordinary one-way ANOVA (Tukey's multiple comparisons test)**.**

(L) Cytoplasmic and nuclear fraction of *S. flexneri* infected (MOI 1:25, 4h) GFP-RIPK2 expressing HeLa cells subjected to western blot analysis with indicated antibodies

(M) Luciferase assay performed with the cell lysate of HEK293T cells transfected with NF-κB luciferase reporter vector and electroporated with purified RIPosomes (from insoluble fractions) with or without NOD1 or NOD2 and. Mean ± SD, n=3. **p < 0.005 and ***p < 0.0005, ordinary one-way ANOVA (Tukey's multiple comparisons test).

Appendix Fig S3. IRGM interacts and co-localizes with NODs, RIPK2, and RIPosomes.

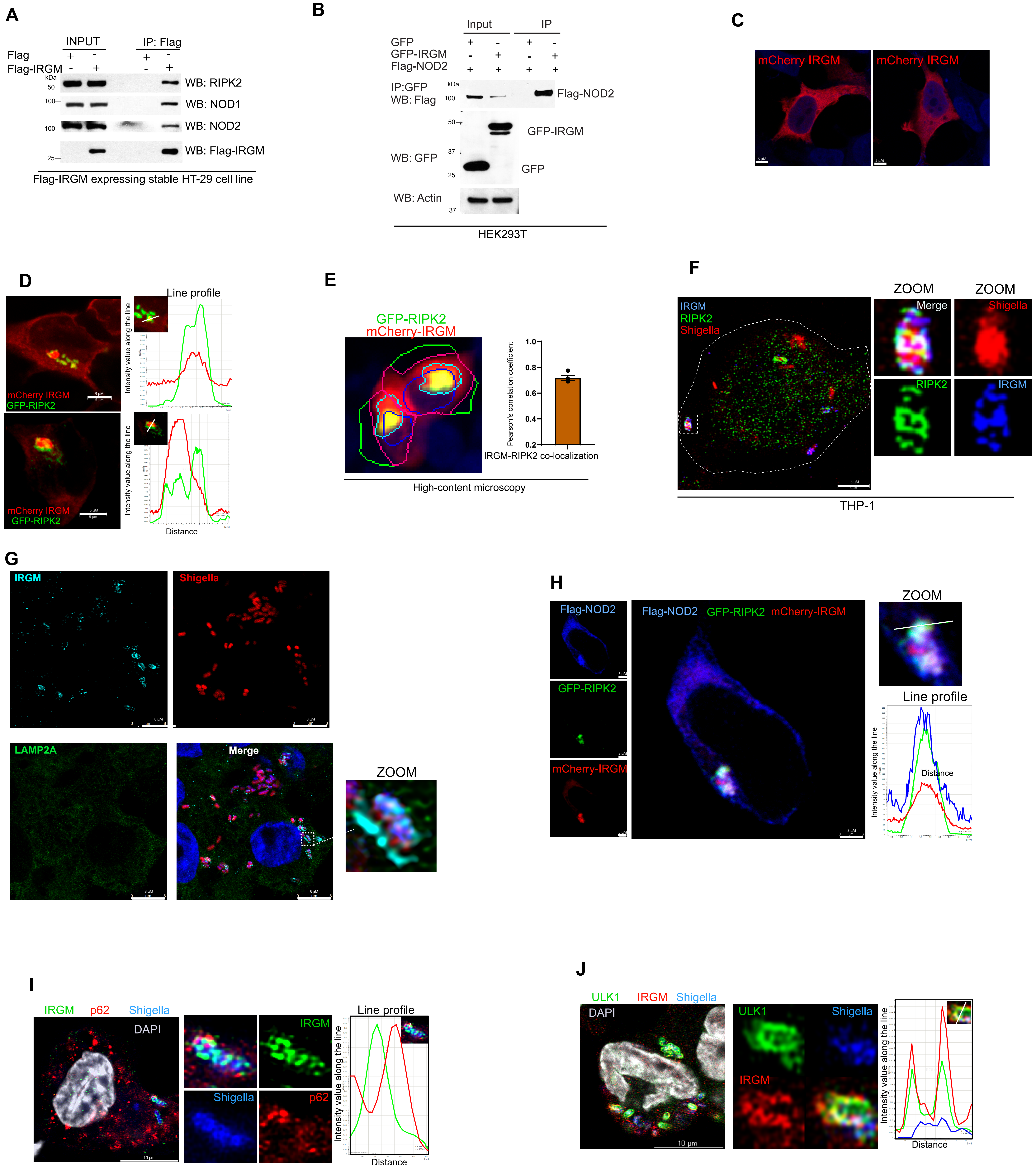

Appendix Fig S3. IRGM interacts and co-localizes with NODs, RIPK2, and RIPosomes.

(A) The lysates of HT-29 cells stably expressing control vector or Flag-IRGM were subjected to immunoprecipitation assay with Flag antibody and western blots are performed with indicated antibodies.

(B) Co-immunoprecipitation analysis using GFP antibody with lysates of HEK293T cell expressing Flag-NOD2 and GFP-IRGM.

(C-D) Representative confocal images of HEK293T cells transiently expressing **(C)** mCherry-IRGM or (**D)** mCherry-IRGM and GFP-RIPK2. Zoom panel is a digital magnification. Line profile: co-localization analysis using line intensity profile. Scale bar, 5 µm.

(E) Left panel, representative high-content microscopy image of HEK293T cells (green masks for GFP-RIPK2, red mask for mCherry-IRGM, and cyan mask for co-localization, software algorithms-defined cell boundaries) transfected with GFP-RIPK2 and mCherry-IRGM. About 17000 cells were plated per well in a 96-well plate and 35 fields per well were screened (n=4, biological replicates). The graph depicts Pearson's correlation coefficient.

(F) Representative confocal images of THP-1 cells infected with RFP expressing *S. flexneri* (MOI 1:25, 8 hpi) and immunostained with RIPK2 and IRGM antibodies. Zoom panels are digital magnification. Scale bar, 5 µm

(G) Representative confocal images of THP-1 cells infected with *S. flexneri* (MOI 1:25, 20 min) and immunostained with LAMP2A and IRGM antibodies. Zoom panels are digital magnification. Scale bar, 8µm

(H) Representative confocal images of HEK293T cells transfected with Flag-NOD2, GFP-RIPK2, and mCherry-IRGM. Zoom panels are digital magnification. Line profile: co-localization analysis using line intensity profile. Scale bar, 3 µm.

(I-J) Representative confocal images of THP-1 cells infected with *S. flexneri* (8 hpi) and immunostained with (**I)** p62 and IRGM antibodies or (**J)** ULK1 and IRGM antibodies**.** Zoom panels are digital magnification. Line profile: co-localization analysis using line intensity profile. Scale bar, 10 µm. (RFP-*Shigella* is pseudo colored to blue and p62 (panel **I**) or IRGM (panel J) are pseudo colored to red for better visualization of co-localization/juxtaposition, DAPI is pseudo colored to grey)

Appendix Fig S4. IRGM mediates degradation of NODs, RIPK2 and RIPosomes

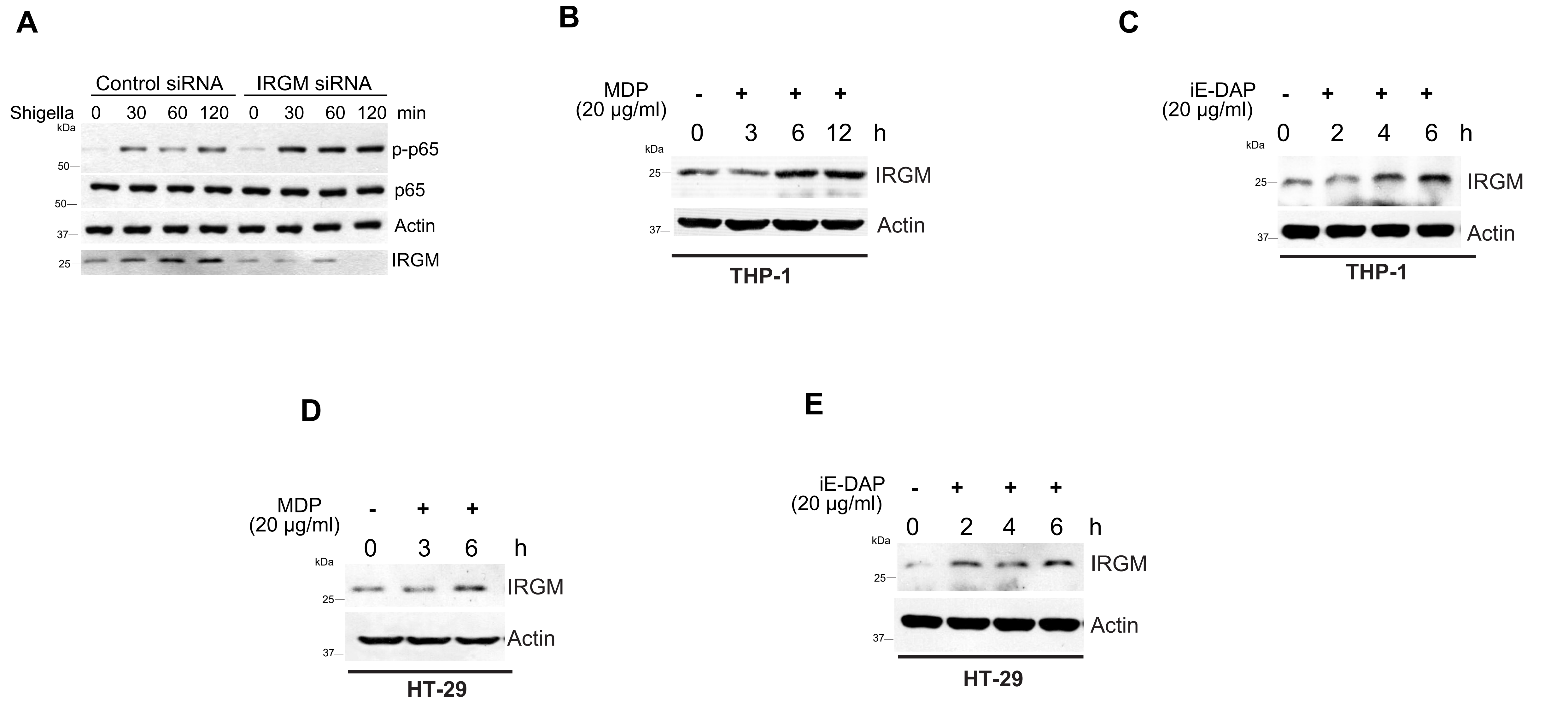

Appendix Fig S4. **IRGM mediates degradation of NODs, RIPK2 and RIPosomes.**

(**A**) Western blot analysis with the cell lysate of control and IRGM knockdown THP-1 cells infected with S. flexneri (MOI 1:25) for indicated time points and probe with indicated antibodies.
(**B-C**) Western blot analysis with the cell lysate of THP-1 cells treated with (B) MDP (20 μg/ml) or (C) iE-DAP (20 μg/ml) for indicated time points and probe with indicated antibodies.
(**D-E)** Western blot analysis with the cell lysate of HT-29 cells treated with (D) MDP (20 μg/ml) or (E) iE-DAP (20 μg/ml) for indicated time points and probe with indicated antibodies.

Appendix Figure S5. IRGM and p62 cooperate to mediate autophagic degradation of NODs, RIPK2, and RIPosomes

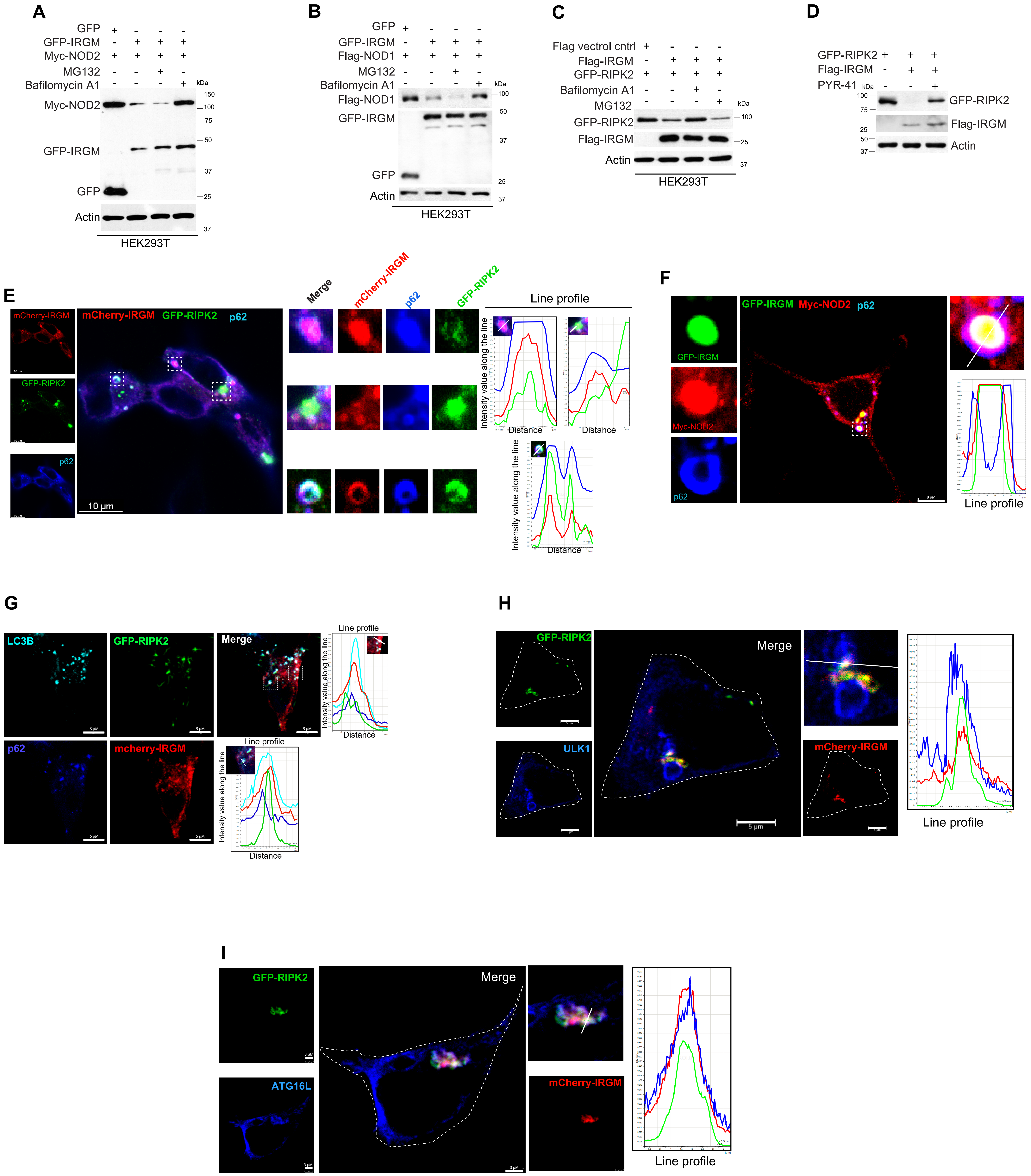

Appendix Fig S5. IRGM and p62 cooperates to mediate autophagic degradation of NODs, RIPK2 and RIPosomes.

(A-C) The cell lysate of HEK293T cells transfected with indicated plasmids and treated with MG132 (20 μM, 5 h) or Bafilomycin A1 (300 nM, 5 h) were subjected to immunoblot analysis.

(D) Western blot analysis with the lysate of HEK93T cells transiently transfected with GFP-RIPK2 in the presence and absence of Flag-IRGM for 4 h followed by treatment with PYR-41 (10 μM) for 5 h.

(E-F) Representative confocal images of HEK293T cells transfected with (**E)** GFP-RIPK2 and mCherry-IRGM, or (**F)** GFP-IRGM and myc-NOD2 immuno-stained with p62 antibody. Line profile: co-localization analysis using line intensity profile. Zoom panels are digital magnifications.

(G) Representative confocal images of HEK293T cells transfected with GFP-RIPK2 and mCherry-IRGM for 9h and immunostained with LC3b and p62.

(H-I) Representative confocal images of HEK293T cells transiently transfected with (**H**) GFP-RIPK2 and myc-ULK1 or (**I)** GFP-RIPK2 and Flag-ATG16L, Scale bar 5. Line profile: co-localization analysis using line intensity profile. Zoom panels are digital magnifications.

Appendix Table S1

Primers	Sequences (5’- 3’)
Human IL-1β-Forward	AAGCTTGGTGATGTCTGGTC
Human IL-1β-Reverse	ACAAAGGACATGGAGAACACC
Human TNF-α Forward	ACTTTGGAGTGATCGGCC
Human TNF-α Reverse	AACATGGGCTACAGGCTTG
Human IL-1A Forward	TGTATGTGACTGCCCAAGATG
Human IL-1A Reverse	TTAGTGCCGTGAGTTTCCC
Human IL17C Forward	GAGGTGTTGGAGGCAGAC
Human IL17C Reverse	CAGCTTCTGTGGATAGCGG
Human TNFSF10 Forward	GTCTCTCTGTGTGGCTGTAAC
Human TNFSF10 Reverse	GGGTCCCAATAACTGTCATCTTC
Human CXCL-2 Forward	AACCGAAGTCATAGCCACAC
Human CXCL-2 Reverse	CTTCTGGTCAGTTGGATTTGC
Human CXCL-6 Forward	GTTTGTCTGGACCCGGAAG
Human CXCL-6 Reverse	GTCCAGGGATCTCCAGAAAAC
Human CXCL-10 Forward	CTCCAGTCTCAGCACCATG
Human CXCL-10 Reverse	CAGGTACAGCGTACAGTTCTAG
Human IFIT1 Forward	TGAAGCCCTGGAGTACTATGAG
Human IFIT1 Reverse	AAAGTGGCTGATATCTGGGTG
Human MX1 Forward	CCAGTAATGTGGACATCGCC
Human MX1 Reverse	CTTGTCTTCAGTTCCTTTGTCC
Human OAS2 Forward	GAATACCTGAAGCCCTACGAAG
Human OAS2 Reverse	GACTGTTTTCCGTCCATAGGAG
Human TRIM22 Forward	GCACGCTCATCTCAGATCTC
Human TRIM22 Reverse	TCAATGTCCAGCTTTCACTCC
Human RSAD2/VIPERIN Forward	GAAGAGGACATGACGGAACAG
Human RSAD2/VIPERIN Reverse	CAATTAAGAGGCACTGGAACAC
Human β- Actin Forward	GAGCACAGAGCCTCGCCTTT
Human β- Actin Reverse	ACATGCCGGAGCCGTTGTC
Mouse TNF-α Forward	ACGGACCCCAAAAGATGAAG
Mouse TNF-α Reverse	TTCTCCACAGCCACAATGAG
Mouse IL-1β Forward	GGAACTGGCAGAAGAGGCACTC
Mouse IL-1β Reverse	GCAGGAATGAGAAGAGGCTGAGAC
Mouse IL-1A Forward	CCACTTGGTTAAATGACCTGC
Mouse IL-1A Reverse	GCTCACGAACAGTTGTGAATC
Mouse IFIT1 Forward	AGAGTCAAGGCAGGTTTCTG
Mouse IFIT1 Reverse	AAGCAGATTCTCCATGACCTG
Mouse MX1 Forward	AGGCAGTGGTATTGTCACCA
Mouse MX1 Reverse	AGACTTTGCCTCTCCACTCC
Mouse ISG15 Forward	GAGAGCAAGCAGCCAGAAG
Mouse ISG15 Reverse	CCCAGGCCATTGCTGCAGGC
