# Supplementary material for: Selective autophagy of RIPosomes maintains innate immune homeostasis during bacterial infection
Source: EMBO J. 2022 Oct 11;41(23):e111289. doi: 10.15252/embj.2022111289 (PMC9713718; doi:10.15252/embj.2022111289)
Supplement: Supplementary file 22 [file EMBJ-41-e111289-s022.pdf]

THE EMBO JOURNAL

Selective Autophagy of RIPosomes Maintains Innate Immune Homeostasis during Bacterial Infection

Santosh Chauhan, Subhash Mehto, Kautilya Jena, Rina Yadav, Swatismita Priyadarsini, Pallavi Samal, Sivaram Krishna, Kollori Dhar, Ashish Jain, Nishant Chauhan, Krushna Murmu, Ramyasingh Bal, Rinku Sahu, Pundrik Jaiswal, Bhabani Sahoo, Srinivas Patnaik, Thomas Kufer, Tor Erik Rusten, Swati Chauhan, and Punit Prasad
DOI: 10.15252/embj.2022111289

Corresponding author(s): Santosh Chauhan (schauhan@ils.res.in) , Subhash Mehto (subhash@ils.res.in)

Review Timeline:

Submission Date:	28th Mar 22
Editorial Decision:	9th May 22
Revision Received:	11th Aug 22
Editorial Decision:	8th Sep 22
Revision Received:	12th Sep 22
Accepted:	15th Sep 22

Editor: Karin Dumstrei

Transaction Report:

(Note: With the exception of the correction of typographical or spelling errors that could be a source of ambiguity, letters and reports are not edited. Depending on transfer agreements, referee reports obtained elsewhere may or may not be included in this compilation. Referee reports are anonymous unless the Referee chooses to sign their reports.)

1st Editorial Decision **9th May 2022**

Dear Santosh,

Thank you for submitting your manuscript to The EMBO Journal. Your study has now been seen by two referees and their comments are provided below.

As you can see from the comments, the referees find the analysis interesting but also that further data is needed to support for autophagy and lysosomal mediated degradation of RIPosome. Should you be able to extend the findings along those lines, then I would like to invite you to submit a revised version.

I am happy to discuss the raised points further and we can do so via email or a video call.

When preparing your letter of response to the referees' comments, please bear in mind that this will form part of the Review Process File, and will therefore be available online to the community. For more details on our Transparent Editorial Process, please visit our website: https://www.embopress.org/page/journal/14602075/authorguide#transparentprocess

I thank you for the opportunity to consider your work for publication. I look forward to discussing your revisions further with you.

with best wishes

Karin

Karin Dumstrei, PhD
Senior Editor
The EMBO Journal

Instructions for preparing your revised manuscript:

I have attached a PDF with helpful tips on how to prepare the revision.

Guide For Authors: https://www.embopress.org/page/journal/14602075/authorguide

We realise that it is difficult to revise to a specific deadline. In the interest of protecting the conceptual advance provided by the work, we recommend a revision within 3 months (7th Aug 2022).

As a matter of policy, competing manuscripts published during this period will not negatively impact on our assessment of the conceptual advance presented by your study. However, we request that you contact the editor as soon as possible upon publication of any related work, to discuss how to proceed.

Please discuss the revision progress ahead of this time with the editor if you require more time to complete the revisions.

Use the link below to submit your revision:

https://emboj.msubmit.net/cgi-bin/main.plex

Referee #1:

In this paper, the authors demonstrate that selective autophagy of detergent insoluble higher-order structures consisting of RIPK2 and NODs called RIPosome prevents persistent activation of NF-κB. They showed that RIPosome formation is induced upon infection of pathogenic bacteria, and RIPosomes localize around the bacteria. While the RIPosomes activate NF-κB, they are degraded in lysosomes. Mechanistically, the authors showed that RIPK2 interacts with multiple autophagy-related proteins such as ULK1, ATG16L and BECN1 and a selective autophagy receptor p62, which leads to autophagic degradation of RIPosomes . In addition, they found that IRGM, which has an ability to assemble not only autophagy-machineries but also pattern recognition receptors also associates with the RIPosome and participates to selective autophagy of RIPosomes. They proposed that p62 and IRGM cooperatively function to prevent persistent activation of NF-κB. Finally, in vivo analysis with Irgm1-deficient mice revealed that susceptibility to inflammation by bacterial infection and DSS-treatment in Irgm-knockout mice is suppressed by the inhibition of Ripk2. The presented study were well-conducted, and this reviewer had a few comments.

1. p62 is a selective autophagy receptor for ubiquitinated proteins. Are RIPosome components ubiquitinated? The E1-inhibitor is

available to examine whether ubiquitination is required for the selective autophagy of RIPosomes or not.
2. Since p62 and IRGM both have ability to bind to autophagy-machineries such as FIP200, ULK1, ATG16L and BECN1, a significance of the interaction of RIP2K with ULK1, ATG16L1 and BECN-1 is unclear. Whether the interaction defective mutation(s) of RIPK2 with ATG proteins has an effect on NF-κB activation remains unclear.
3. According to the author's model, THP-1 cells knocked out or knocked down ATG5 or p62 should show similar phenotypes observed in IRGM-knockdown THP-1 (IL-1β、TNFα induction and the suppression by RIPK2) . The authors should conduct such experiments.
4. Since cycloheximide is known to inhibit autophagy, autophagy-flux experiments using cycloheximide may be inappropriate.
5. Immunoblot data presented in this manuscript were obscure. The quantification for I.B. analyses are required.

Referee #2:

In the presented manuscript by Mehto et al, authors have shown that S.flexnerii and S.Typhimurium infections induce the formation of insoluble RIPK2 oligomers which are recruited to the bacteria (possibly the bacteria containing vacuoles) which subsequently activates NFkB. They further show that the RIPK2 oligomers and the RIPosomes consisting of RIPK2 and NOD1/2 are targeted into autophagosomes by p62 for degradation. Also, p62 mediated autophagic degradation of RIPosomes is mediated by IRGM. They also show that inhibition of RIPK2 using a small molecule ameliorates gut inflammation. The study has several layers and is interesting, however several data presented is not clear to the reviewer and are contradictory. IRGM-RIP2 and NOD regulating inflammation is understandable and obvious but the role of autophagy and lysosomal degradation of RIPosome is less convincing.

I will detail my concerns below.

Major concerns:
1. Authors claim that they have revealed that RIPosome formation upon infection, which is not entirely right. Ellwanger et al, 2019 Li Sci Alli - have already shown that RIPosomes are formed upon Shigella infection. Although the authors state that Ellwanger et al have shown RIPosome formation upon ectopically expressed RIPK2, they claim that the existence of RIPosome under physiological conditions such as infection is not established. I am surprised by this statement.
2. Mehto et al, claim that RIPosomes are targeted for degradation by autophagy but Ellwanger et al have shown that RIPosomes are regulated by XIAP and proteasomal degradation which is contradictory. However, authors have not addressed the role of XIAP, but they show that RIPK2 oligomers are not degraded via proteasomes. How do they reconcile their data with Ellwanger et al.
3. Riposome colocalizing with bacteria is shown as the novelty, which was not observed by Ellwanger et al. They also show that the NOD1/NOD2 ligands did not show visible RIposome formation but the oligomers were detected in the insoluble fraction which is intriguing. Fig 1A and several other figures where authors have used the DAPI to identify Shigella is unconvincing. Shigella appear as rods as they have shown in Fig 1G and 1H but the structures shown as bacteria appear to me as fragmented DNA. Also, many of the green spots which the authors claim as bacteria do not colocalize or juxtapose with the Riposome. RIP2 appears as big blob over the green spot - overexposed? In Supp Fig 2D authors have used either fluorescent bacteria or stained for Shigella specifically. Similar procedure has to be used throughout.
Fig1b and Fig1J - high content microscopy, I presume the grey color depicts DNA (please label in the image) but the bacterial DNA is not found to colocalize with RIP2.
4. In Fig2F the authors claim that NOD2 colocalize with RIPK2 and are juxtaposed to bacteria. The image shows some NOD2 dots colocalizing with RIP2K but the bacteria are not found to be covered with RIPosomes as the authors have stated earlier.
Fig 2H - The blot does not convincingly show that NOD enhances the oligomerization of RIPK2. GFP-RIPK2 lane is narrower compared to the other 2 lanes but the expression of RIPK2 appears the same.
Fig2L - p65 translocation into nucleus is doubtful. Red channel is saturated. Why was the nucleus not stained? It is not possible to decipher nuclear translocation of p65. Phospho-p65 immunoblot will be helpful.
5. Authors state inhibition of autophagy using bafilomycin A. Bafilomycin inhibits lysosomes not autophagy per se. As stated above degradation of RIP2K through autophagosomes and not through proteosomes contradicts the previous publication by Ellwanger et al. The role of XIAP in the context of autopagosomal degradation need to be addressed.
Fig 3A - ATG5 knock down in Shigella infected cells appear to be less efficient, despite that RIPK2 and NOD protein levels are higher. It is also not clear to me if the basal RIPK2 monomer is degraded by lysosomes as the HT-29 cells were not infected with Shigella or RIPK2 was ectopically expressed to form oligomers (Supp Fig 3).
Why was HEK293T cells treated with rapamycin which is not a physiologically relevant system (Supp Fig 3D)?
Fig 3E - RIPK2 monomer (soluble fraction) is increased in ATG5 depleted cells, however upon Shigella infection RIPK2 levels remain unaltered. This is not similar to what authors observed in Fig 3A.
6. When cargo is degraded via autophagy, the autophagy receptors also get degraded. There is no evidence of p62 degradation shown. Does Shigella induce autophagy or block autophagy? Accumulation of RIposomes upon infection (microscopy and insoluble fraction) suggests that autophagy is inhibited if RIPosomes are to be degraded via autophagy.
Fig 3F- Bacteria are not found to colocalize with RIPk2.
7. Suppl Fig 4H and 4I - How were nucleus and Shigella stained. Figure legends are less descriptive.
Fig 4L - It is intriguing that there is only one speck of IRGM-RIPK2 complex present in the entire cell.
Line 297-298: It is mentioned that IRGM is recruited to cytosolic Shigella. How did the authors conclude that the bacteria is

cytosolic and not present within a phagosome or bacteria containing vesicle?
LC3 staining is required to show if the bacteria and RIPosomes are targeted to autophagosomes.

8. Data presented (Supp Fig 5B) suggests that IRGM is induced upon Shigella infection. If IRGM mediates the degradation of RIPK2, how is RIPK2 expression and oligomerization increased upon Shigella infection compared to uninfected cells?
Fig 5G - Increased degradation of RIPk2 oligomers in cells overexpressing IRGM compared to cells expressing FLAG plasmid is not visible in the blot presented which makes IRGM-dependent degradation questionable.

9. Authors conclude that IRGM suppresses NFkB activation. Shigella enhances IRGM expression but NFkB activation must be enhanced as well. Did the authors test for NFkB activation at different time points post infection? This should be tested to establish the physiological relevance of IRGM-Riposome regulation in the context of infection.

10. IRGM, p62 and RIPK2 interact but this does not suggest that they are targeted to autophagosomes. If p62 specifically interacts with IRGM and RIPK2 individually, are these proteins separately targeted to autophagosomes or is the whole complex targeted? LC3 staining is needed.

11. RNA-seq data suggests that several inflammatory pathways are upregulated in IRGM-depleted cells upon infection which is conceivable. However, ER-phagy, endosomal, vacuolar pathways meaning lysosomal pathways are upregulated in IRGM-depleted cells. Previous data suggests that IRGM increased degradation through autophagy which is contradictory.

Minor comments:
The figure legends are less descriptive.
The scale bars are hardly visible
Many of the microscopy images are saturated making it hard to derive conclusions.
Manuscript needs to be edited for grammar.

Referee #1:

In this paper, the authors demonstrate that selective autophagy of detergent insoluble higher-order structures consisting of RIPK2 and NODs called RIPosome prevents persistent activation of NF-κB. They showed that RIPosome formation is induced upon infection of pathogenic bacteria, and RIPosomes localize around the bacteria. While the RIPosomes activate NF-κB, they are degraded in lysosomes. Mechanistically, the authors showed that RIPK2 interacts with multiple autophagy-related proteins such as ULK1, ATG16L and BECN1 and a selective autophagy receptor p62, which leads to autophagic degradation of RIPosomes . In addition, they found that IRGM, which has an ability to assemble not only autophagy-machineries but also pattern recognition receptors also associates with the RIPosome and participates to selective autophagy of RIPosomes. They proposed that p62 and IRGM cooperatively function to prevent persistent activation of NF-κB. Finally, in vivo analysis with Irgm1-deficient mice revealed that susceptibility to inflammation by bacterial infection and DSS-treatment in Irgm-knockout mice is suppressed by the inhibition of Ripk2. The presented study were well-conducted, and this reviewer had a few comments.

Response: We are very thankful to the reviewer for reading our manuscript thoroughly and for the appreciation. We have now further improved our manuscript by carefully addressing the constructive comments of the reviewers and performing a large number of experiments. Thanks a lot for your time and consideration!

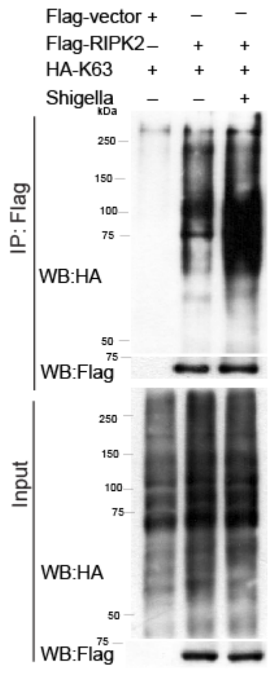

Figure 1

1. p62 is a selective autophagy receptor for ubiquitinated proteins. Are RIPosome components ubiquitinated? The E1-inhibitor is available to examine whether ubiquitination is required for the selective autophagy of RIPosomes or not.

Response: Thanks for the concern. The reviewer is correct. The literature suggests that p62 recognizes K63-linked ubiquitinated autophagy cargo.

*First, we tested whether Shigella infection induces RIPK2 (the major component of the RIPososmes) ubiquitination. Indeed, the Shigella infection increased the K63-linked ubiquitination of RIPK2 (**Figure 1 and Manuscript Fig EV1D**). Also, in immunofluorescence assays, we found that RIPosomes are ubiquitinated (**Figure 2 and Manuscript Fig 3F**). Next, we tested whether the endogenous RIPosomes that are recruited over the Shigella are ubiquitinated or not. Indeed, we found that ubiquitin puncta were juxtaposed or co-localized with RIPosomes coating the bacteria (**Figure 3 and Manuscript Fig EV1E**). The RIPosomes were formed not just around bacteria but throughout the infected cells. These cytosolic RIPosomes were also juxtaposed or co-localized with ubiquitin (**Figure 3 and Manuscript Fig EV1E**).*

*The E1 inhibitor PYR-41 is a selective and cell-permeable inhibitor of ubiquitin-activating enzyme E1 (PMID: 17909057). In high-content microscopy, we found that rapamycin-induced autophagic degradation of RIPosomes is blocked by PYR-41 in two different experimental setups ((Fig 4A) RIPK2 transfection then PYR-41 treatment (Fig 4B) PYR-41 treatment then RIPK2 transfection) (**Figure 4 and Manuscript Fig EV1F**) indicating that ubiquitination is required for autophagic degradation of RIPosomes. In addition, we found that IRGM-mediated degradation (autophagic) of RIPK2 protein is rescued upon treatment of cells with PYR-41 signifying that ubiquitination of RIPK2 and RIPosomes is important for their autophagic degradation (**Figure 5 and Manuscript Appendix Fig S5D**).*

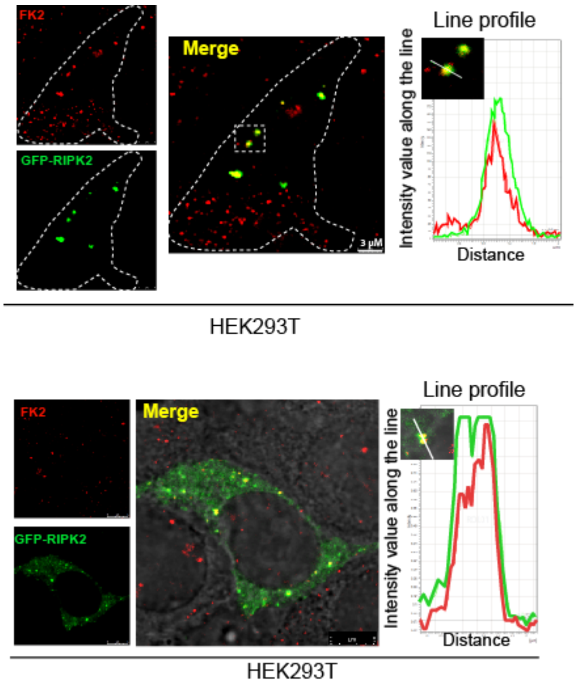

Figure 2

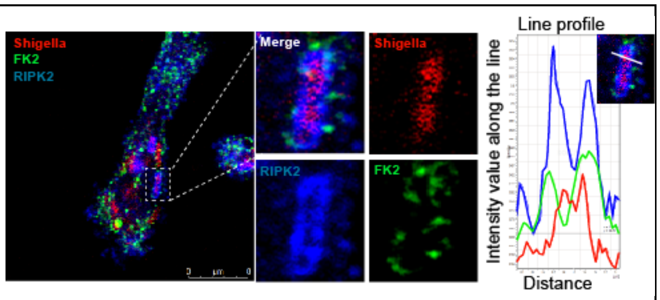

Figure 3

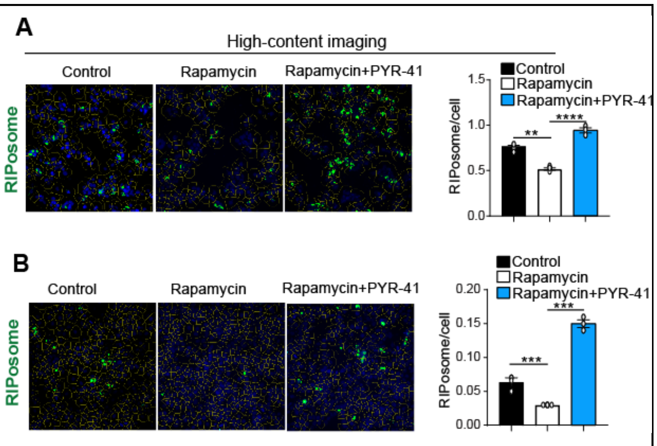

Figure 4

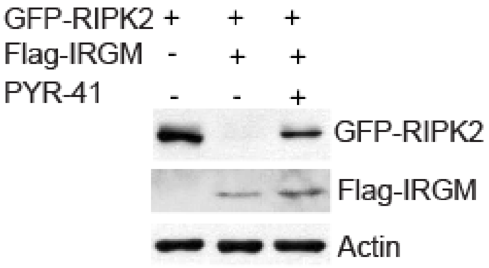

Figure 5

2. Since p62 and IRGM both have ability to bind to autophagy-machineries such as FIP200, ULK1, ATG16L and BECN1, a significance of the interaction of RIP2K with ULK1, ATG16L1 and BECN-1 is unclear. Whether the interaction defective mutation(s) of RIPK2 with ATG proteins has an effect on NF-κB activation remains unclear.

Response: We understand the concern. The cell usually has several fail-safe redundant mechanisms to resolve the inflammation. We showed that p62 and IRGM both cooperatively downregulate the RIPosomes numbers via autophagy. We found that there was a direct interaction of autophagy proteins and IRGM with RIPK2. It could be possible that autophagy proteins, IRGM and p62 are in the same complex that interacts with RIPosomes resulting in direct interactions. Also, it could be possible that where IRGM and p62 aid in the delivery of RIPosomes to preformed autophagosomal structures, the autophagy proteins (ULK1, Beclin1, and ATG16L1) could aid in de novo autophagosome formation juxtaposed to the RIPosomes.
I am sure that the reviewer will appreciate that a large amount of work is performed in this manuscript to show for the first time that endogenous RIPosomes are formed and are targeted by autophagy. In this process, we showed that indeed RIPK2 directly interacts with multiple autophagy proteins. It could be possible that RIPK2 makes contact with multiple residues of these different ATGs. At present, we even don't know which interfaces are primary. I am sure that the reviewer will understand that determining the interface between RIPK2 and ATG proteins (ULK1, ATG16L, BECLIN-1) and then determining the effect on NF-κB activation is an independent full study itself. I humbly request the reviewer to consider our point.

3. According to the author's model, THP-1 cells knocked out or knocked down ATG5 or p62 should show similar phenotypes observed in IRGM-knockdown THP-1 (IL-1β、TNFα induction and the suppression by RIPK2) . The authors should conduct such experiments.

*Response: Thanks for the concern. The proposed experiment with ATG5 knockdown was already present in the previous version of the manuscript. Indeed, the knockdown of ATG5 phenocopy the effects of IRGM knockdown in THP-1 cells (i.e. IL-1β, TNFα induction, and the rescue by RIPK2 knockdown) (**Manuscript Figure 3P**). Now, we have performed the suggested experiment with p62 knockdown cells (as well). Again, the p62 knockdown showed similar phenotypes as observed in IRGM or ATG5 knockdown cells (and rescue by RIPK2 knockdown) (**Figure 6 and Manuscript Fig EV1M**). Thanks for the important suggestion.*

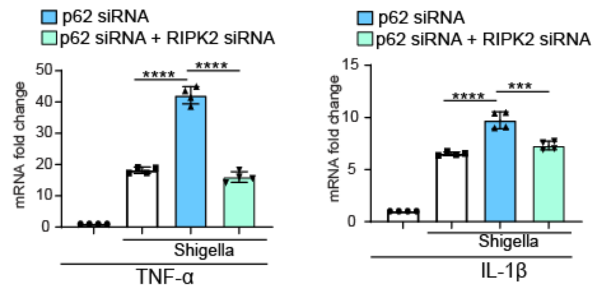

Figure 6

4. Since cycloheximide is known to inhibit autophagy, autophagy-flux experiments using cycloheximide may be inappropriate.

Response: We understand the reviewer's concern. However, we would like to refer to the publication from Dr. Noboru Mizushima's lab (Watanabe-Asano, PMID: 24525133, DOI: 10.1016/j.bbrc.2014.01.180) which shows that cycloheximide inhibits autophagy only in amino acid starvation conditions but not in basal conditions (Refer to Figure 1A and 1B). Our experiments were performed in basal conditions.

5. Immunoblot data presented in this manuscript were obscure. The quantification for I.B.

analyses are required.

Response: We have provided the quantifications of western blots where results appears less evident (Figure 3A, 3B, 5A, 5D, 5E, 5I, 5J, 5K , EV2A, EV2B, EV2C, EV2D, etc) . Thanks a lot for the suggestion!!

Referee #2:

In the presented manuscript by Mehto et al, authors have shown that S.flexnerii and S.Typhimurium infections induce the formation of insoluble RIPK2 oligomers which are recruited to the bacteria (possibly the bacteria containing vacuoles) which subsequently activates NFkB. They further show that the RIPK2 oligomers and the RIPosomes consisting of RIPK2 and NOD1/2 are targeted into autophagosomes by p62 for degradation. Also, p62 mediated autophagic degradation of RIPosomes is mediated by IRGM. They also show that inhibition of RIPK2 using a small molecule ameliorates gut inflammation. The study has several layers and is interesting, however several data presented is not clear to the reviewer and are contradictory. IRGM-RIP2 and NOD regulating inflammation is understandable and obvious but the role of autophagy and lysosomal degradation of RIPosome is less convincing.

Response: We are thankful to the reviewer for praising the work and providing us with critical suggestions. We have now performed several experiments to improve the manuscript by carefully addressing the comments. We are very thankful to the reviewer for the time and effort.

I will detail my concerns below.

Major concerns:

1. Authors claim that they have revealed that RIPosome formation upon infection, which is not entirely right. Ellwanger et al, 2019 Li Sci Alli - have already shown that RIPosomes are formed upon Shigella infection. Although the authors state that Ellwanger et al have shown RIPosome formation upon ectopically expressed RIPK2, they claim that the existence of RIPosome under physiological conditions such as infection is not established. I am surprised by this statement.

Response: The lead author of Ellwanger et al., 2019 (Life Science Alliance), Dr. Thomas Kufer is a collaborator and co-author in this manuscript. In this study, for the first time, we identified that ***endogenous*** *RIPosomes are formed in macrophages and they are recruited over the bacteria to induce NF-κB response. This information is novel and significantly important to the field.* ***We apologize for our confusing statement and we have modulated it.*** *Please see lines 60-63 in the manuscript.*

2. Mehto et al, claim that RIPosomes are targeted for degradation by autophagy but Ellwanger et al have shown that RIPosomes are regulated by XIAP and proteasomal degradation which is contradictory. However, authors have not addressed the role of XIAP, but they show that RIPK2 oligomers are not degraded via proteasomes. How do they reconcile their data with Ellwanger et al.

*Response: In this manuscript, we have presented multiple evidence (****genetic as well chemical inhibitors; overexpression and knockdowns/knockout****) in different cell types, in vitro, and in vivo that RIPosomes (endogenous and overexpressed) are targeted by autophagy.*
Whereas, in the case of Ellwanger et al., 2019 while addressing a reviewer comment, they indicated in Supplementary Figure 7D (only one experiment) that RIPosome is accumulated upon MG132 treatment. The role of the proteasome in RIPK2/RIPosome degradation was not extensively tested in Ellwanger et al. Also, the MG132 itself has several non-specific effects, this

includes autophagy inhibition (PMID: 27693582) or induction (PMID: 26648402).
Over and above, the predicted role of the proteasome in RIPK2 degradation is not entirely contradictory to our results. In our cycloheximide chase experiments (Old Manuscript Supplementary Figure 3A-3C and new manuscript figure Fig EV1A-EV1C), we found that where Bafilomycin A1 completely protect RIPK2 and NOD1 from degradation, MG132 partially protected them suggesting a milder role of the proteasome in this process. In addition, it is well-established fact in the field that large protein complexes/aggregates are usually targeted by autophagy but not by the proteasome (As big protein complexes or protein aggregates cannot enter proteasome barrels).
The main focus of our manuscript is to understand whether endogenous RIPosomes are formed (Figures 1 and 2) and if yes how they are degraded by autophagy (Figure 3-5) and what is the physiological relevance of this finding in vivo (Figure 6-7). To understand this, we have performed a very large number of experiments (>200 experiments) and utilized different approaches. Ellwanger et al., 2019 showed that XIAP counteracts RIPosomes formation by inducing ubiquitination of RIPK2 and they did not find any role of XIAP in the degradation of RIPosomes. Hence, further understanding the role of XIAP in this process, which appears nothing to do with autophagy is a completely independent study of an altogether different theme. I request the reviewer to consider our point.

3. Riposome colocalizing with bacteria is shown as the novelty, which was not observed by Ellwanger et al. They also show that the NOD1/NOD2 ligands did not show visible RIposome formation but the oligomers were detected in the insoluble fraction which is intriguing.

Response: Yes, the reviewer is correct.

Fig 1A and several other figures where authors have used the DAPI to identify Shigella is unconvincing. Shigella appear as rods as they have shown in Fig 1G and 1H but the structures shown as bacteria appear to me as fragmented DNA.. In Supp Fig 2D authors have used either fluorescent bacteria or stained for Shigella specifically. Similar procedure has to be used throughout.

*Response: Thanks for the concern. Shigella is rod-shaped bacteria. In confocal microscopy, we are taking images of a single plane (~20-30 nm of thickness) of a cell. So even the rod-shaped bacteria that are not aligned in the XY axis of the plane but moving in the Z direction will appear round or dot-like or fragmented. This is a very standard phenomenon in confocal microscopy (please check, Figure 4 in PMID: 24039575; Figure 5 in PMID: 17696608; Figure 1 in PMID: 24722587). Nevertheless, as suggested by the reviewer, we have performed high-resolution confocal microscopy with fluorescent protein-tagged bacteria (**Figure 7 and Manuscript Fig 1A**). Here also we observe all kinds of shapes of bacteria. For better visualization, now we have provided 3D images and a 3D movie of RIPosomes-trapped Shigella (**Figure 8 and Manuscript Fig 1A and Appendix Figure S1 and Movie EV1**). Almost all panels now use fluorescent protein-tagged bacteria (**Figure 1A, 1H, 1I, 1K, 1L, 1M, 2F, 4G, 4H, 4I, Appendix Figure S1B, S1E, S1G, S1I, S2D, S2E, S3F, S3H, S3I, S3J, and S5G).** Thanks for the suggestion!*

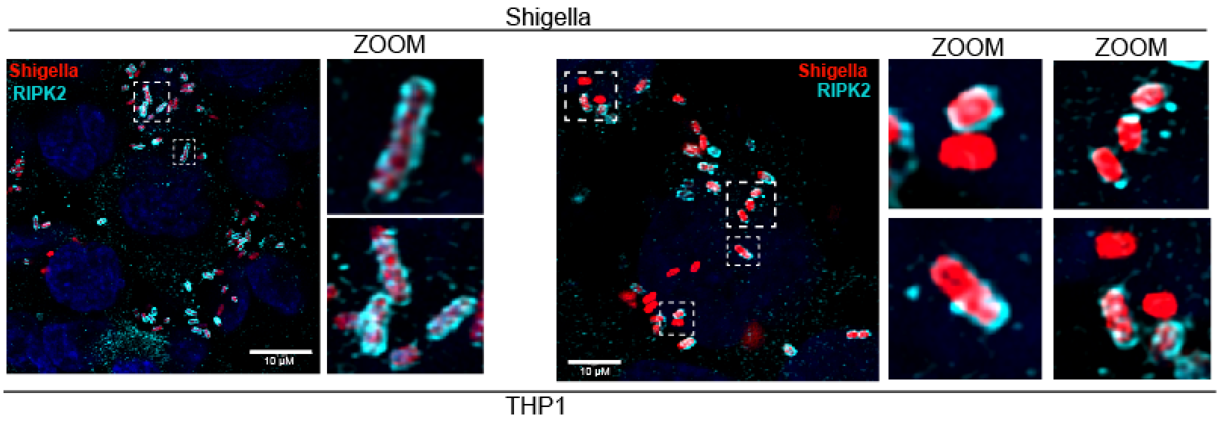

Figure 7

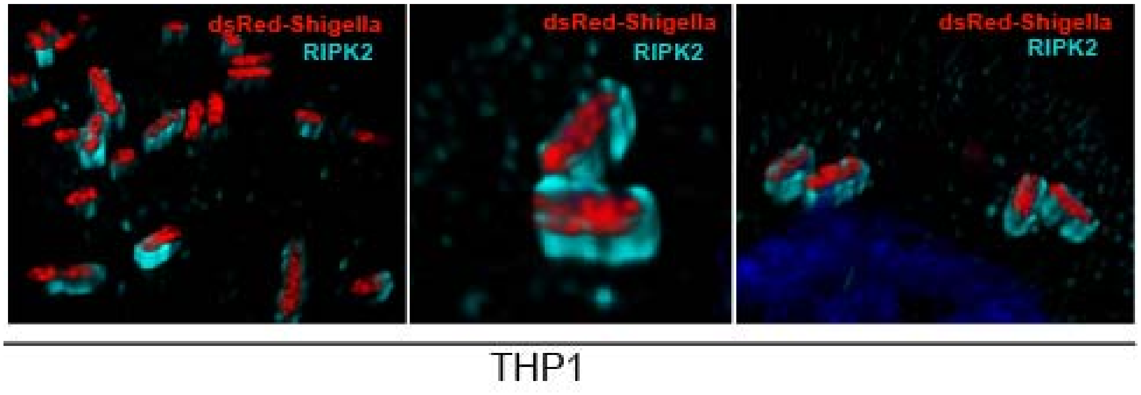

Figure 8

Also, many of the green spots which the authors claim as bacteria do not colocalize or juxtapose with the Riposome

*Response: Thanks for the concern. This is correct that not all the bacteria co-localize with RIPosomes. We have stated this at the starting of our manuscript results section very explicitly (**manuscript lines 109-113**). This is a very typical phenomenon that all the wild-type bacteria are not captured by innate immune systems or autophagy systems, usually only ~5-20% of wild-type bacteria are captured (Figure1 in PMID: 26015503; Figure 5 in PMID: 17696608; Figure 4A in PMID: 21646350; Figure 1 in PMID: 24722587).*
*In addition, we would also like to bring to the kind notice of the reviewer that in addition to recruitment or juxtaposition of RIPosomes (small punta of RIPK2) to the bacteria, the RIPosomes are formed throughout the infected cells (but not in uninfected cells) (**Figure 7 and Manuscript Appendix Fig S1A**). So it is not necessary that all RIPK2 puncta co-localize with bacteria and vice-versa.*

Fig1b and Fig1J - high content microscopy, I presume the grey color depicts DNA (please label in the image) but the bacterial DNA is not found to colocalize with RIP2.
Response: Sorry, we have labelled the images and yes, the grey color depicts DNA. Kindly

*consider that in high-content microscopy, the images are taken in 10X magnification and it is wide-field microscopy (not confocal microscopy). At this low magnification, we can count RIPosome puncta but cannot determine their co-localization with bacteria with a great degree of certainty. In our manuscript, we have first very nicely established that RIPosomes are formed in cells and also are recruited over the bacteria (in vitro and in vivo) using confocal microscopy. Only after establishing the phenomenon, we have used high-content microscopy <u>to quantitate the data</u>. Nonetheless, for the confidence in data, we have now taken a few images of both Shigella (**Figure 9**) and Salmonella (**Figure 10**) infected cells at higher magnification (and colored) using an HCS microscope. Here, we can observe that bacterial DNA does co-localize with RIPK2.*

Figure 9. Shigella infected THP1 cells

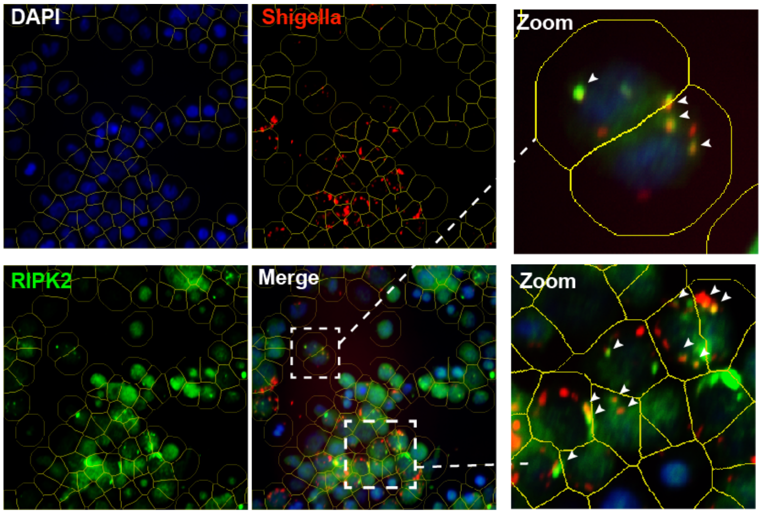

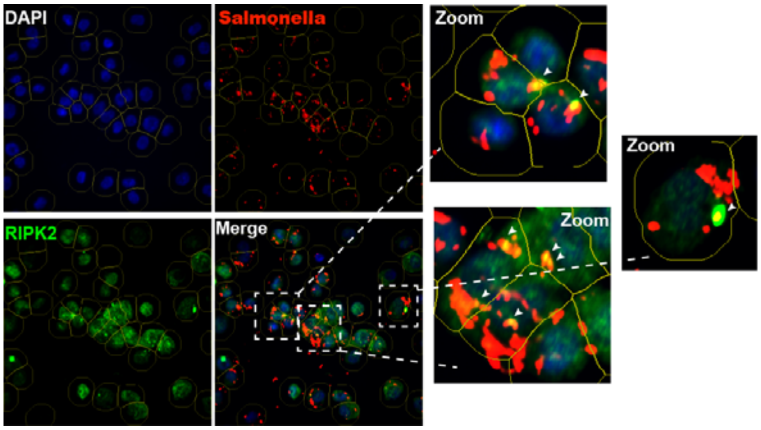

Figure 10. Salmonella infected THP1 cells

4. In Fig2F the authors claim that NOD2 colocalize with RIPK2 and are juxtaposed to bacteria. The image shows some NOD2 dots colocalizing with RIP2K but the bacteria are not found to be covered with RIPosomes as the authors have stated earlier.

Response: Thanks for the concern. As stated above, RIPosomes are not only recruited to the bacteria but also present throughout the infected cells. Also, all bacteria are not covered by RIPosomes. We have now provided another image in the main figure where we can see that NOD2 (pseudo-colored to red) co-localizes with the RIPosomes (Green)

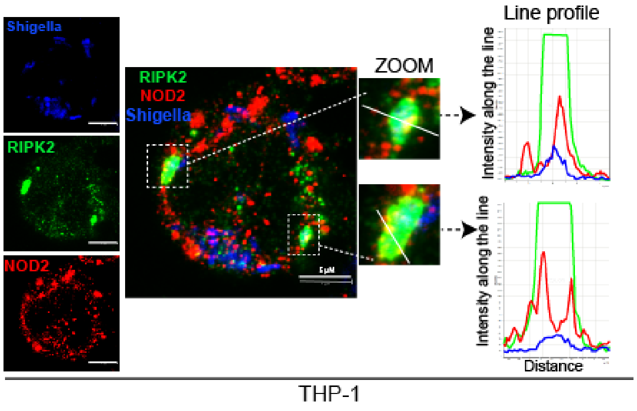

Figure 11

*that are recruited to bacteria (RFP expressing Shigella, pseudo-colored to blue) (**Figure 11 and Manuscript Fig 2F**).*

*NOD1/2 is reported to be recruited to the plasma membrane at the bacterial entry site of epithelial cells (PMID: 19898471). We performed immunofluorescence in HeLa cells (epithelial origin) and found that NOD2 is recruited to the plasma membrane as well as around the RIPosomes that coats Shigella (**Figure 12 and Manuscript Appendix Fig S2E**).*

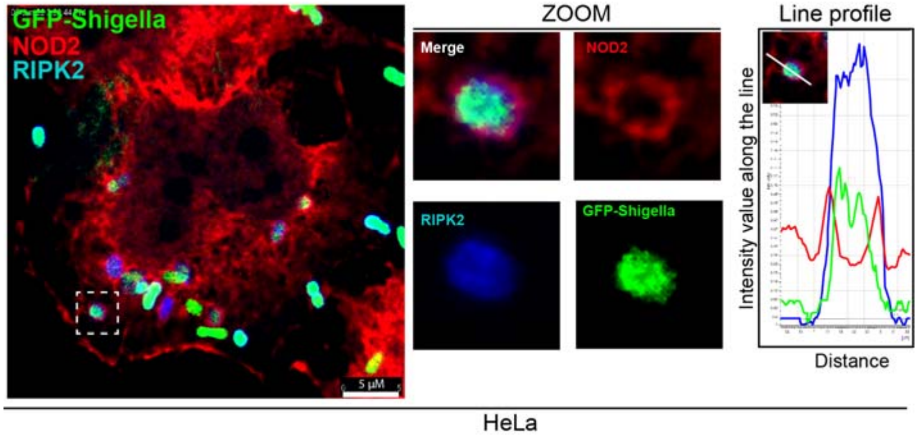

Figure 12

Fig 2H - The blot does not convincingly show that NOD enhances the oligomerization of RIPK2. GFP-RIPK2 lane is narrower compared to the other 2 lanes but the expression of RIPK2 appears the same.

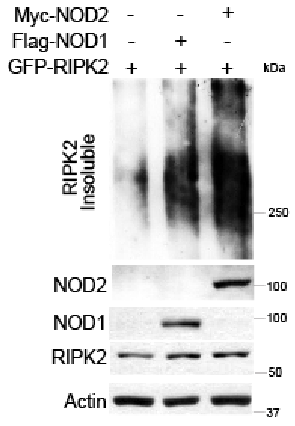

Figure 13

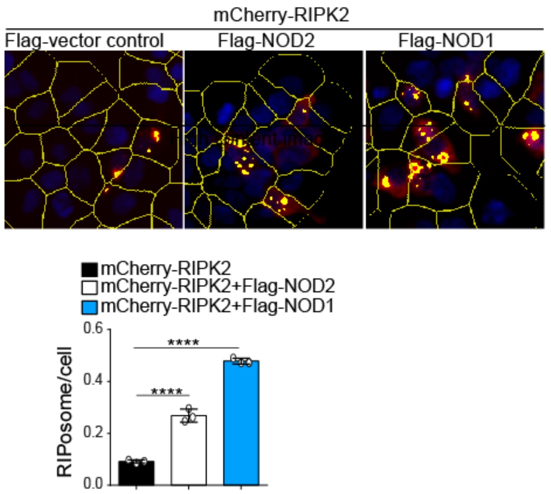

Figure 14

*Response: Thanks for the concern. We have repeated the western blot experiment to confirm whether NODs enhance RIPK2 oligomerization. Indeed, the presence of NODs significantly increases the oligomerization of RIPK2 (**Figure 13 and Manuscript Appendix Fig S2J**). Also, we performed high-content microscopy, which endorse the finding as the NOD1 and NOD2 could significantly enhance the RIPosome formation **(Figure 14 and Manuscript Appendix Fig S2K)**.*

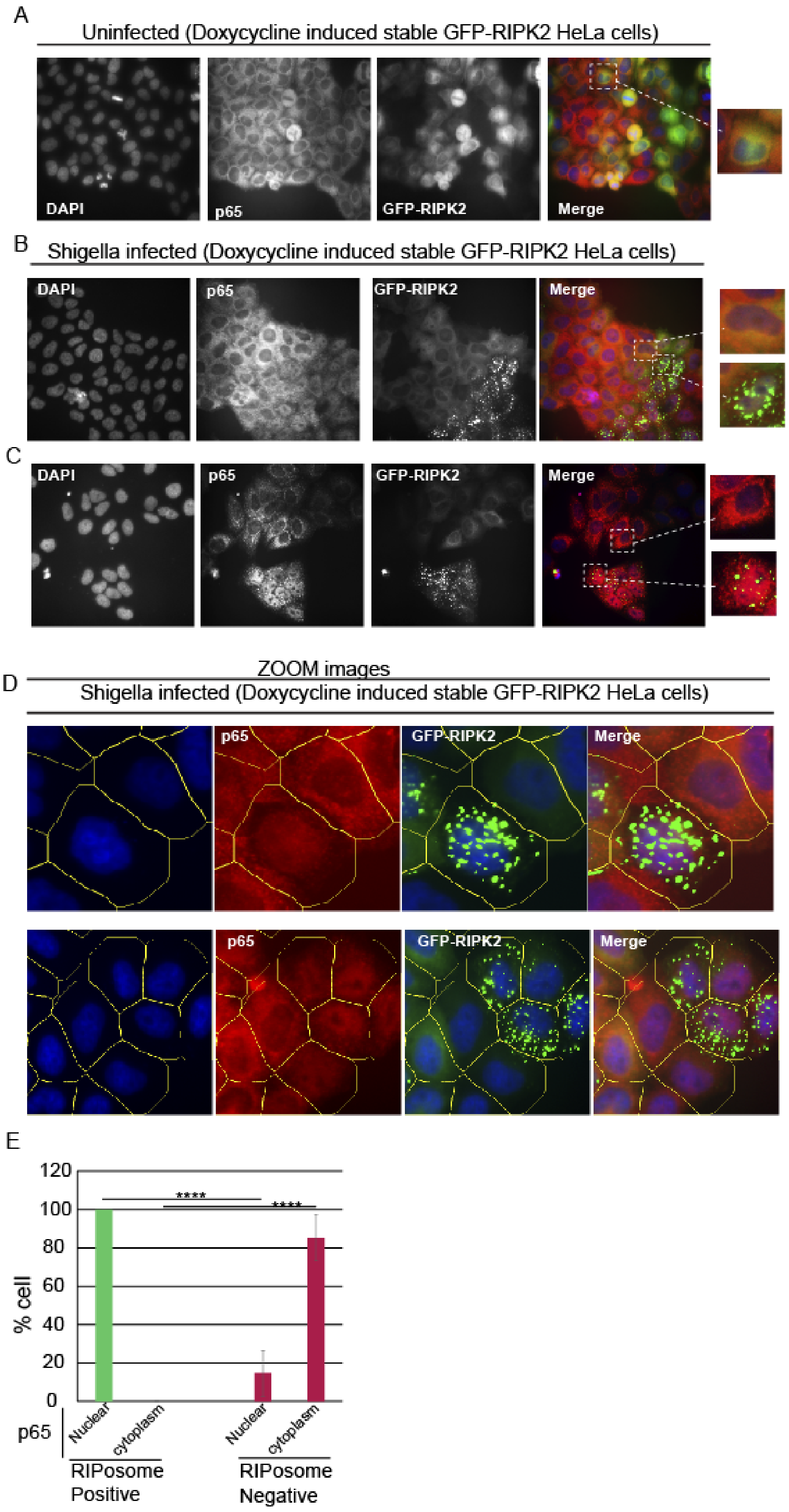
A
Uninfected (Doxycycline induced stable GFP-RIPK2 HeLa cells)
DAPI
p65
GFP-RIPK2
Merge
B
Shigella infected (Doxycycline induced stable GFP-RIPK2 HeLa cells)
DAPI
p65
GFP-RIPK2
Merge
C
DAPI
p65
GFP-RIPK2
Merge
D
ZOOM images
Shigella infected (Doxycycline induced stable GFP-RIPK2 HeLa cells)
p65
GFP-RIPK2
Merge
p65
GFP-RIPK2
Merge
E
% cell
120
100
80
60
40
20
0
****
****
p65
Nuclear
cytoplasm
Nuclear
cytoplasm
RIPosome Positive
RIPosome Negative

Figure 15

Fig2L - p65 translocation into nucleus is doubtful. Red channel is saturated. Why was the nucleus not stained? It is not possible to decipher nuclear translocation of p65. Phospho-p65 immunoblot will be helpful.

*Response: Thanks for the concern. We have now provided nuclear staining in the original images (**Figure 15C, Manuscript Figure 2L**) and also presented digitally zoomed images (**Figure 15B, 15C, and 15D**) (only for the review purpose). These are representative images from high-content screening assays where we have quantified multiple images for RIPosome-associated nuclear translocation of p65 (**Figure 15E, Manuscript Figure 2M).** As stated above, high content imaging is performed at 10X magnification and is wide field microscopy so pictures cannot be as clear as the confocal images are. Nevertheless, the new images show that the p65 is translocated to the nucleus of the cells that are positive for RIPosomes. Whereas the majority of RIPosome-negative cells have cytoplasmic p65. This data is quantitated from three different experiments and 100s of cells (**Figure 15E, Manuscript Figure 2M**)*

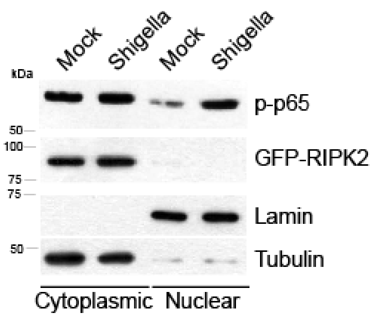

Figure 16

*In addition, as a control, now we have performed high content imaging with UNINFECTED cells that did not form RIPosomes and it is evident that p65 is not translocated to the nucleus in these cells (**Figure 15A**).*

*Further, as suggested by the reviewer, we performed immunoblotting with phospho-p65 (Ser535) from nuclear and cytoplasmic extracts of doxycycline-inducible stable GFP-RIPK2 HeLa cell line. Upon shigella infection, nuclear translocation of phospho-p65 is considerably enhanced (**Figure 16 and Manuscript Appendix Fig S2L**).*

5. Authors state inhibition of autophagy using bafilomycin A. Bafilomycin inhibits lysosomes not autophagy per se. As stated above degradation of RIP2K through autophagosomes and not through proteosomes contradicts the previous publication by Ellwanger et al. The role of XIAP in the context of autopagosomal degradation need to be addressed.

Response: Thanks for the concern. The reviewer is correct that Bafilomycin A1 inhibits lysosome functioning, but the lysosomes are an integral part of the autophagy process. Bafilomycin A1 treatment is the standard method of inhibiting autophagy flux throughout the field and almost all publications that are supposed to show autophagy flux modulation uses Bafilomycin A1. In our manuscript, we didn't just rely upon chemical methods (Bafilomycin or Rapamycin) of autophagy modulation, we have used genetic methods (ATG5 and p62 knockdown and knockout) to support our data.

*The point regarding contradiction with the publication by Ellwanger et al is well discussed above. We would like to bring to the kind notice of the reviewer that there is no link between XIAP and degradation of RIPosome in Ellwanger et al. As explained above the data shown by Ellwanger et al. are not contradictory to our results. They did not find any role of XIAP in RIPosome **degradation** but they discussed that XIAP might modulate signaling events leading to less RIPosomes formation in cells. Given that no such hypothesis exists, understanding the role of XIAP in RIPosomes autophagy is out of the context of the current manuscript theme.*

Fig 3A - ATG5 knockdown in Shigella infected cells appear to be less efficient, despite that

RIPK2 and NOD protein levels are higher.

*Response: We have changed the blot from the repeat experiment performed from the same lysates (**Figure 17 and Manuscript Fig 3A**). Thanks for the concern and for asking us to correct it.*

It is also not clear to me if the basal RIPK2 monomer is degraded by lysosomes as the HT-29 cells were not infected with Shigella or RIPK2 was ectopically expressed to form oligomers (Supp Fig 3).

Response: The reviewer is correct that autophagy degrades RIPK2 monomers (first two lanes Figure 3A and 3B) as well as oligomers (Figure 3C, 3D, 3E, 3H etc). In Supplementary Figure 3A-C, we chased endogenous RIPK2 monomers degradation in the absence and presence of cycloheximide.

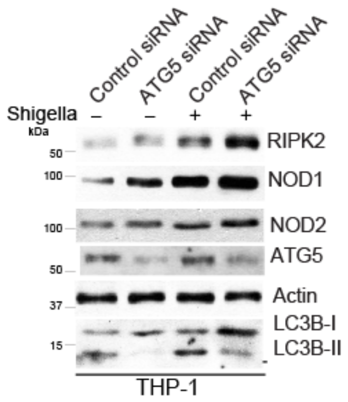

Figure 17

Why was HEK293T cells treated with rapamycin which is not a physiologically relevant system (Supp Fig 3D)?

*Response: Rapamycin is an inhibitor of mTOR activity and hence an inducer of autophagy and is very often used to induce autophagy flux and in assessing the degradation of autophagy cargoes. This is an additional data and since the reviewer pointed it out as unimportant to show, **we have removed the data**.*

Fig 3E - RIPK2 monomer (soluble fraction) is increased in ATG5 depleted cells, however upon Shigella infection RIPK2 levels remain unaltered. This is not similar to what authors observed in Fig 3A.

Response: We are very sorry for the confusion. However, it appears no problem with the data. Please note that the experimental conditions in Figures 3A and 3E are different. Where Fig 3A data is from total cell lysate, in Fig 3E western blot are run from soluble (lower panel) and an insoluble fraction (upper panel) of the cell lysate. Please note that RIPK2 monomers (soluble) are converted into oligomers (insoluble) upon Shigella infection. In total lysate (as in Figure 3A), the total amount (soluble and insoluble) of RIPK2 is increased upon Shigella infection. In Figure 3E, upon Shigella infection, the increase in RIPK2 oligomers is observed in the insoluble fraction (upper panel), and hence RIPK2 monomers (soluble, lower panel) remain unaltered, which is very much correct.

6. When cargo is degraded via autophagy, the autophagy receptors also get degraded. There is no evidence of p62 degradation shown.

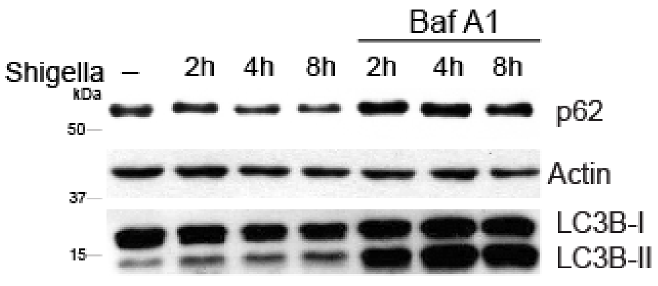

Figure 18

*Response: Thanks for the suggestion. We infected THP-1 cells with Shigella for different time points and observed that indeed p62 is degraded upon Shigella infection, which is rescued upon Bafilomycin A1 treatment indicating an autophagy-dependent degradation of p62 **(Figure 18 and Manuscript Fig EV1L)**.*

Does Shigella induce autophagy or block autophagy? Accumulation of RIposomes upon infection (microscopy and insoluble fraction) suggests that autophagy is inhibited if RIPosomes are to be degraded via autophagy.

Response: The central theme of the host-pathogen interaction is that the host cell mounts cell-autonomous defense and the pathogen blocks it (PMID: 28660242, PMID: 29233878, PMID: 22704617, PMID: 26904515). Here also, upon infection, the host cell induces autophagy and Shigella tries to block it. So both processes go hand-by-hand. This is well documented.
*Upon infection, RIPosomes accumulate because the total amount of RIPK2 and its oligomerization is increased (data in Manuscript Figure 1 and Figure 2) (not because autophagy is inhibited). These enhanced RIPosomes are **balanced** (**Homeostatic balance**) by their autophagic degradation (data in Manuscript Figure 3, Figure 5, and Figure 6). This is apparent from our data (Figure 3, Figure 5, Figure 6) that blocking autophagy by genetic or chemical means further induces the RIPosomes numbers and inflammation. Taken together our data show that upon Shigella infection RIPosomes numbers are increased to induce NF-κB response whereas autophagy suppresses this inflammatory response to maintain innate homeostasis.*

Fig 3F- Bacteria are not found to colocalize with RIPK2.
*Response: As discussed above, not all bacteria are co-localized with RIPosomes and vice versa. RIPosomes are present throughout the cells infected with bacteria (but not in uninfected cells). In the previous image, we were showing that RIPosomes were co-localizing with Ubiquitin. Now, we have presented another image where bacteria is juxtaposed to RIPK2 and ubiquitin **(Figure 19 and Manuscript Fig EV1E**). Thanks for the concern.*

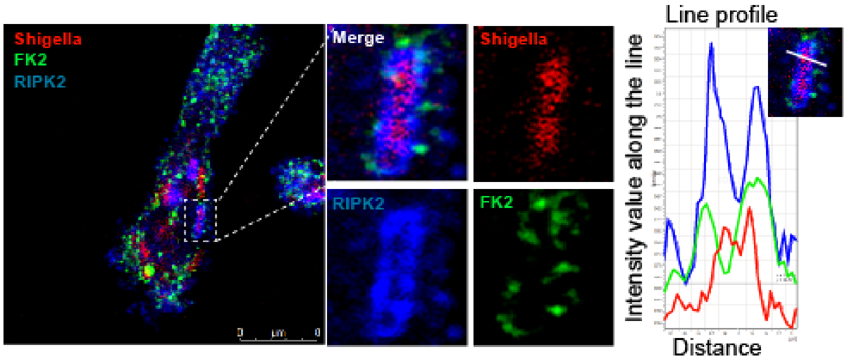

Figure 19

7. Suppl Fig 4H and 4I - How were nucleus and Shigella stained. Figure legends are less descriptive.
Response: We are sorry. We have described the legends properly now. Nucleus is stained with DAPI and Shigella was expressing RFP.

Fig 4L - It is intriguing that there is only one speck of IRGM-RIPK2 complex present in the entire cell.
*Response: Figure 4L in old manuscript figures was Western blot experiments, not immunofluorescence data. We guess the reviewer is talking about Figure 4I (?), where we have presented the colocalization of IRGM-RIPK2 and NOD2. We have presented more images here (only for the review purpose) **(Figure 20**). We found some cells form a single complex*

*(**Manuscript Fig 4J**) and some cells have several complexes (Figure 20).*

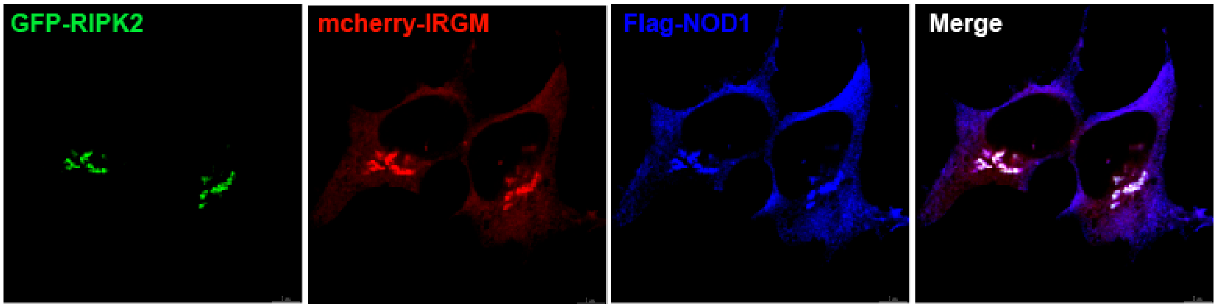

Figure 20

Line 297-298: It is mentioned that IRGM is recruited to cytosolic Shigella. How did the authors conclude that the bacteria is cytosolic and not present within a phagosome or bacteria containing vesicle?

*Response: Shigella is a predominantly cytosolic bacterium that ruptures its bacteria-containing vesicle very rapidly (within 10-15 minutes) after the invasion (PMID: 20070313; PMID: 27763815; PMID: 27092296). Also, Shigella tends to avoid the recruitment of Rab GTPase's and other maturation proteins (including LAMP proteins) using several methods (PMID: 27763815). We infected THP1 cells with Shigella for 20 minutes and performed immunofluorescence with EEA1 (early phagosome/endosome marker) or LAMP2A (late phagosome/endosome maturation marker) and IRGM to understand whether IRGM is recruited to phagosomal or cytosolic Shigella. We found that very few bacteria were positive for EEA1 protein (**Figure 21 and Manuscript Fig 4I**) and didn't observe LAMP2A recruitment over the Shigella (**Figure 22 and Manuscript Appendix Figure S3G**) confirming the previous publication's observations. EEA1-marked phagosomes were rarely localized with IRGM. (**Figure 21 and Manuscript Fig 4I**). Whereas IRGM was strongly recruited to several of the EEA1 negative bacteria (**Figure 21 and Manuscript Fig 4I**). The data suggest that IRGM is recruited to Shigella once they escaped or escaping the phagosomes. The IRGM is present on multiple bacteria that are LAMP2A negative (**Figure 22 and Manuscript Appendix Figure S3G**). Thanks for the question !!*

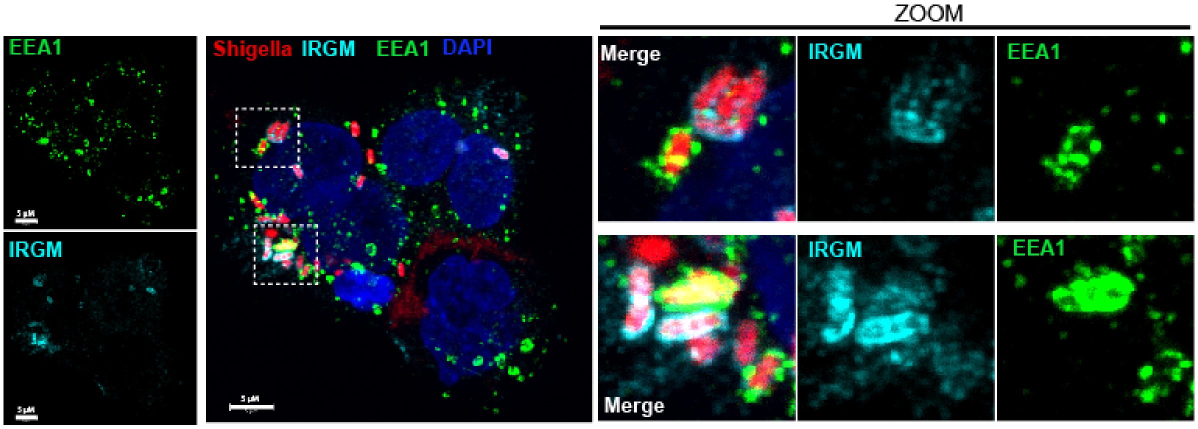

Figure 21

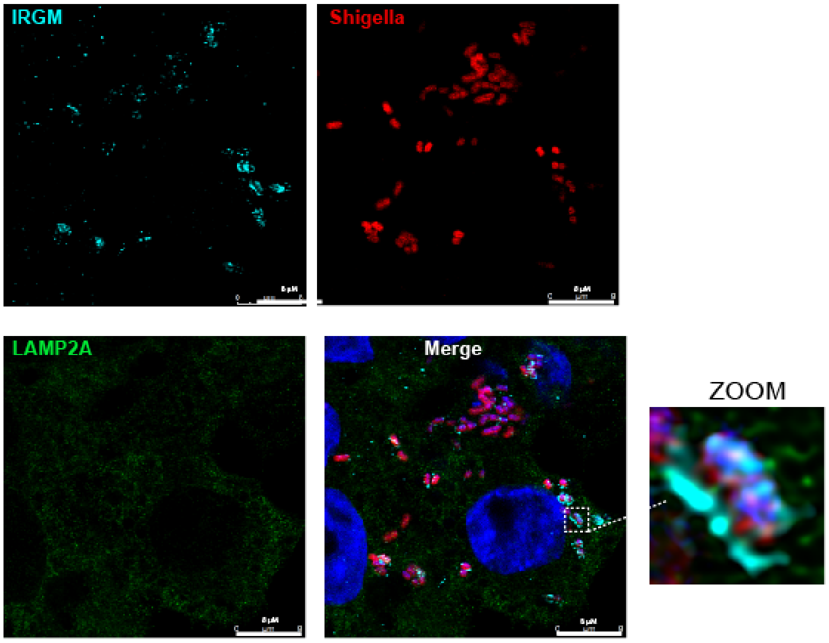

Figure 22

LC3 staining is required to show if the bacteria and RIPosomes are targeted to autophagosomes.

*Response: We performed the experiment as suggested by the reviewer. We found that LC3B perfectly co-localized over the RIPosomes that are covering the Shigella (**Figure 22B and manuscript Fig EV1I**) suggesting that RIPosomes and bacteria are targeted to autophagosomes. Thanks for the suggestions!*

Figure 22B

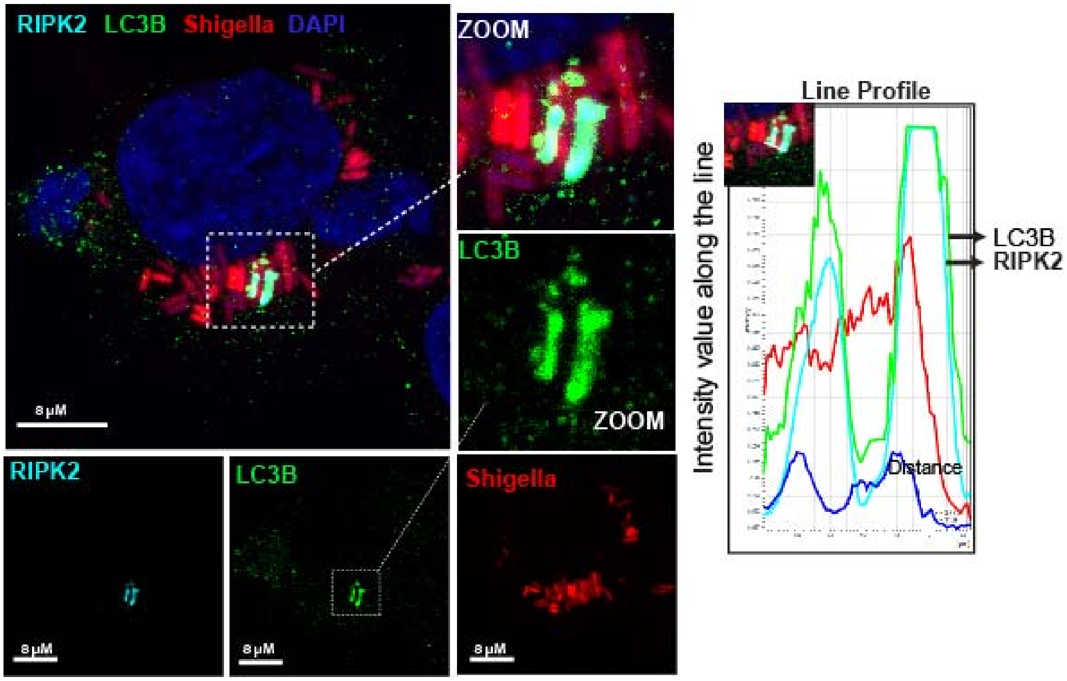

8. Data presented (Supp Fig 5B) suggests that IRGM is induced upon Shigella infection. If IRGM mediates the degradation of RIPK2, how is RIPK2 expression and oligomerization increased upon Shigella infection compared to uninfected cells?

Response: The induced IRGM expression suppresses the NOD2-RIPK2-NFκB response by degrading the RIPosomes and NODs/RIPK2. This is a negative feedback mechanism for maintaining inflammatory homeostasis in the cell. Please consider that NF-κB response is important for controlling Shigella infection, however same time several negative feedback mechanisms (not just IRGM) are switched on to cut down excess inflammation. These negative mechanisms do not completely shut down inflammatory mechanisms but suppress them so that it does not hurt the host but at the same time restrict the bacterial growth. This is one of the principles of maintaining immune homeostasis.
Upon Shigella infection, both IRGM expression and RIPosome number are increased. The increased IRGM mediates the autophagic degradation of RIPK2 and RIPosomes to reduce their amount (not completely abolish it). This is very clear from a large amount of data present in Figures, 5, 6, and 7 that if IRGM is not present NODs-RIPK2- NF-κB response is amplified in vitro and in vivo. IRGM-dependent autophagy maintains the homeostasis of RIPosomes numbers and thus NF-κB response. This negative feedback mechanism is true not just for IRGM (PMID: 30612879; PMID: 32715615) but for a large number of other proteins that are negative regulators of inflammation (PMID: 29357192; PMID: 34826185; PMID: 28356568: PMID: 21639951).

Fig 5G - Increased degradation of RIPk2 oligomers in cells overexpressing IRGM compared to cells expressing FLAG plasmid is not visible in the blot presented which makes IRGM-dependent degradation questionable.

Response: We are sorry for the confusion. However, it appears that there is no problem with the data. In Figure 5G, we showed that NOD2 induces RIPK2 oligomerization (lane 2 vs lane 1) and this enhanced Nodo-RIPosome complex is reduced by IRGM overexpression (lane 3 vs lane 2). NO lane in this Figure is just expressing RIPK2 and IRGM, which can be compared with lane 1 (Flag+RIPK2). The result, the reviewer is looking for is presented in Figure EV2G (old manuscript ***Supplementary Figure 5G)*** *which clearly shows the IRGM-dependent degradation of RIPK2 oligomers. Additionally, we would like to point out that the conclusion that IRGM degrades RIPK2 oligomers or RIPOsomes is not made from one panel but from* ***a large number of complementary assays*** *that include immunofluorescence, high-content microscopy, and western blotting. Please check Figures 5B, 5C, 5F, 5H, 5L, EV2F, EV2G, EV2H, EV2I, and EV2K.*

9. Authors conclude that IRGM suppresses NFkB activation. Shigella enhances IRGM expression but NFkB activation must be enhanced as well. Did the authors test for NFkB activation at different time points post infection? This should be tested to establish the physiological relevance of IRGM-Riposome regulation in the context of infection.

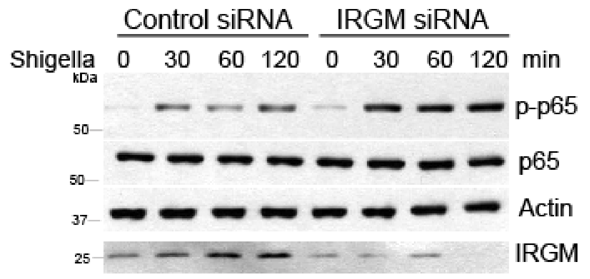

Figure 23

Response: We performed the experiment as suggested by the reviewer. To test the activation of NF-κB, we chased the phosphorylation (Ser536) of p65 in the presence and absence of IRGM ***(Figure 23 and Manuscript Appendix Fig S4A****). The phosphorylation of p65 is induced upon Shigella infection that is further enhanced upon IRGM knockdown. The data suggest that Shigella-induced IRGM expression suppresses the NF-κB activation.*

10. IRGM, p62 and RIPK2 interact but this does not suggest that they are targeted to autophagosomes. If p62 specifically interacts with IRGM and RIPK2 individually, are these proteins separately targeted to autophagosomes or is the whole complex targeted? LC3 staining is needed.

Response: Our data show that both RIPK2 monomers and oligomers are targeted by autophagy (Fig 3, Fig EV1). We have presented immunofluorescence, immunoprecipitation, and biochemical data to show that p62 acts as a receptor for autophagic degradation of RIPosomes. Additionally, in Figure 6 (now figure 5) and supplementary figure 6 (now figure appendix figure 5), we show that p62 and IRGM cooperatively mediate the degradation of RIPosomes.

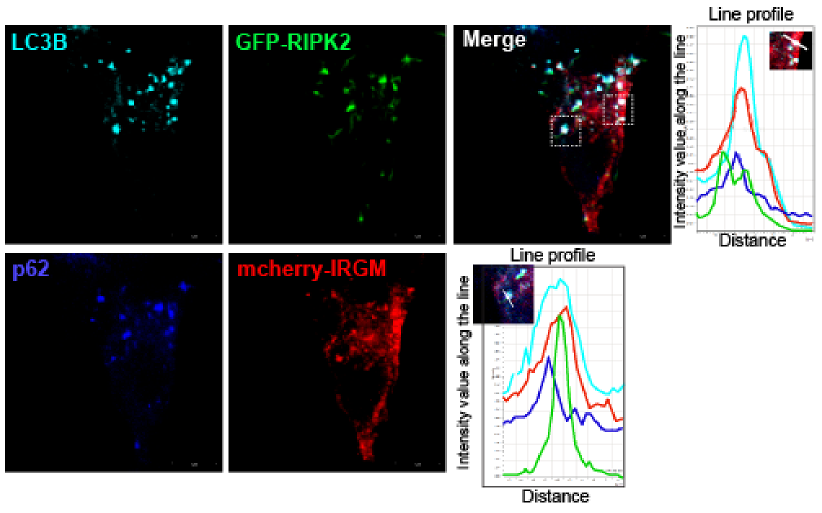

Figure 24

We already demonstrated that RIPK2, IRGM, and p62 are in the same complex. Now, as per the reviewer's suggestion, we have performed immunofluorescence assays and observed co-localization/juxtaposition of RIPosomes with IRGM, p62, and LC3B ***(5 color microscopy, Figure 24 and Manuscript Appendix Fig S5G****) supporting our data that RIPosomes are targeted to autophagosomes with the help of IRGM and p62. Thanks for the suggestion!*

11. RNA-seq data suggests that several inflammatory pathways are upregulated in IRGM-depleted cells upon infection which is conceivable. However, ER-phagy, endosomal, vacuolar pathways meaning lysosomal pathways are upregulated in IRGM-depleted cells. Previous data suggests that IRGM increased degradation through autophagy which is contradictory.

Response: We are sorry for the confusion. However, we feel that there is no contradiction in the data. Please allow us to explain the same:

In our RNA seq analysis, there is ***NO*** *gene ontology (GO) term named* ***ER-phagy*** *that is*

upregulated. Probably, the reviewer wants to point the ***ER-Phagosome*** *pathway. The term was only observed in Reactome analysis (Figure EV7A and EV7B) but not in Metascape analysis (Figure 7B, Figure EV7E and EV7F). Predominantly, upregulation of cytokines pathways (>300 genes upregulated) was observed upon IRGM knockdown (not autophagic/lysosomal pathways). We carefully examined the gene pool of the term mentioned by the reviewer and appeared in Reactome pathway analysis only (not in Metascape analysis).*

1. ***Endosomal/Vacuolar*** *– only 5 genes are upregulated under this term (HLA-H; HLA-B; HLA-C; HLA-F; B2M; CTSS) (Dataset EV2) and all the terms except one* ***are related to immunity****-related vacuolar pathway but not to the lysosomal pathway (HLA genes or B2M gene which is MHC class 1 pathway-related gene).*

2. ***ER-Phagy:*** *In our GO analysis data there is* ***NO term called ER-Phagy****. May be reviewer wants to point out the ER-Phagosome pathway. Again the genes represented under this term (HLA-H;SMARCD3;HLA-B;TAP2;HLA-C;TAP1;TAPBPL;HLA-F;PSMB10;PSMB8;PSMB9;PANX2;SLC7A8;SGSM2;LGALS9;TLR4;B2M) are mainly related HLA or MHC class 1 and 2 genes.* ***Hence are related to immunity****.*

Taken together there are ***hardly any lysosomal pathway genes that are represented in the terms indicated by the reviewer****. Such terms are not representative as they didn't appear in Metascape analysis whereas all the inflammation-related terms were common in Reactome and Metascape analysis. Moreover, upregulation of one or 2 gene expressions that too at the transcription level does not dictate biological processes. We and others never found that autophagy or lysosome biogenesis is induced upon IRGM knockdown (PMID: 32753672, PMID: 30612879, PMID: 33665357, PMID: 32715615, PMID: 25891078, PMID: 21102437, PMID: 29420192).*

Minor comments:
The figure legends are less descriptive.
Response: We are sorry for that. We have improved on them.

The scale bars are hardly visible
Response: We are sorry for that. We have increased the width of the scale bars in all figures.

Many of the microscopy images are saturated making it hard to derive conclusions.

Response: We have added several new figures. To see the bacteria and associated structures, its typical requirement is that the intensity of the image has to be kept high. Nevertheless, we have improved wherever possible in new images and also change old ones without compromising the clarity.

Manuscript needs to be edited for grammar.

Response: Thanks for the concern. We have carefully edited the manuscript for grammar.

1st Revision - Editorial Decision 8th Sep 2022

Dear Santosh,

Thank you for submitting your revised manuscript to The EMBO Journal. Your study has now been seen by the two referees. As you can see from the comments below, the referees appreciate the introduced changes and support publication here. I am therefore very pleased to let you know that we will accept your manuscript for publication here. Before doing so there are just a few points that need to be resolved:

Referee #2 raise one additional point that you can address with text changes either in the MS or the point-by-point response.

The following formatting points also need to be sorted out:

- please add 3-5 keywords

- Take a look at the funding information. I think it is incomplete in the journal submission system.

- "CRediT has replaced the traditional author contributions section because it offers a systematic machine readable author contributions format that allows for more effective research assessment. Please remove the Authors Contributions from the manuscript and use the free text boxes beneath each contributing author's name in our system to add specific details on the author's contribution. More information is available in our guide to authors."

- Conflict of Interest should be re-labeled Disclosure and competing interests statement

- please check the reference format. For citations with more than 10 authors truncate the author list at 10 authors followed by et. al

- For the 6 EV Datasets, the legends need to be added to the files as a separate tab

- For the Movie files, please remove the legends from the main MS and zip with the corresponding movie file.

- There is a callout for "Supplementary data 7", pls correct.

- Will you make sure that the deposited datasets are open and accessible

- Our publisher has also done their pre-publication check on your manuscript. When you log into the manuscript submission system you will see the file "Data Edited Manuscript file". Please look at the word file and the comments regarding the figure legends and respond to the issues.

- Please double check that the size of the synopsis image is correct => 550 wide by [200-400] high (pixels).

- We include a synopsis of the paper (see http://emboj.embopress.org/). Please provide me with a general summary statement and 3-5 bullet points that capture the key findings of the paper.

That should be all! Congratulations on a nice study

with best wishes

Karin

Karin Dumstrei, PhD
Senior Editor
The EMBO Journal

Use the link below to submit your revision:

https://emboj.msubmit.net/cgi-bin/main.plex

Referee #1:

The authors have addressed all my concerns and therefore I support the publication.

Referee #2:

The manuscript is substantially improved and the authors have addressed most of my concerns. However, the following points need to be addressed.

Authors now claim that "Ellwanger et al., 2019 showed that XIAP counteracts RIPosomes formation by inducing ubiquitination of RIPK2 and they did not find any role of XIAP in the degradation of RIPosomes. Hence, further understanding the role of XIAP in this process, which appears nothing to do with autophagy"

It is premature to think that XIAP does not have a role in the degradation of RIPosomes through autophagy without doing an experiment. Especially because XIAP has also been shown to regulate autophagy. It is important to discuss the role of XIAP in RIPosome formation in the context of autophagy.

It is important to specify that Bafilomycin A1 inhibits autophagy flux rather than autophagy as it does not prevent autophagosome formation.

Referee #2:

The manuscript is substantially improved and the authors have addressed most of my concerns. However, the following points need to be addressed.

Thanks to the reviewer for supporting.

Authors now claim that "Ellwanger et al., 2019 showed that XIAP counteracts RIPosomes formation by inducing ubiquitination of RIPK2 and they did not find any role of XIAP in the degradation of RIPosomes. Hence, further understanding the role of XIAP in this process, which appears nothing to do with autophagy"
It is premature to think that XIAP does not have a role in the degradation of RIPosomes through autophagy without doing an experiment. Especially because XIAP has also been shown to regulate autophagy. It is important to discuss the role of XIAP in RIPosome formation in the context of autophagy.

We have now discussed the probable role of XIAP in RIPosome turnover in the discussion part. Please see line number 518-521

It is important to specify that Bafilomycin A1 inhibits autophagy flux rather than autophagy as it does not prevent autophagosome formation.

We agree with reviewer and changed word "autophagy" to "autophagy flux". Please see line number 219. Thanks for the comment.

Dear Santosh,

Thank you for submitting your revised manuscript to The EMBO Journal. I have now looked at everything and all looks good.

I am therefore very pleased to accept the MS for publication here.

Congratulations on a nice study!

With best wishes

Karin

Karin Dumstrei, PhD
Senior Editor
The EMBO Journal

Please note that it is EMBO Journal policy for the transcript of the editorial process (containing referee reports and your response letter) to be published as an online supplement to each paper. If you do NOT want this, you will need to inform the Editorial Office via email immediately. More information is available here:
https://www.embopress.org/page/journal/14602075/authorguide#transparentprocess

Your manuscript will be processed for publication in the journal by EMBO Press. Manuscripts in the PDF and electronic editions of The EMBO Journal will be copy edited, and you will be provided with page proofs prior to publication. Please note that supplementary information is not included in the proofs.

You will be contacted by Wiley Author Services to complete licensing and payment information. The required 'Page Charges Authorization Form' is available here: https://www.embopress.org/pb-assets/embo-site/tej_apc.pdf - please download and complete the form and return to embopressproduction@wiley.com

EMBO Press participates in many Publish and Read agreements that allow authors to publish Open Access with reduced/no publication charges. Check your eligibility: https://authorservices.wiley.com/author-resources/Journal-Authors/open-access/affiliation-policies-payments/index.html

Should you be planning a Press Release on your article, please get in contact with embojournal@wiley.com as early as possible, in order to coordinate publication and release dates.

If you have any questions, please do not hesitate to call or email the Editorial Office. Thank you for your contribution to The EMBO Journal.

** Click here to be directed to your login page: https://emboj.msubmit.net

EMBO Press Author Checklist

Corresponding Author Name: Santosh Chauhan	
Journal Submitted to: The EMBO J	
Manuscript Number: EMBOJ-2022-111289R	

USEFUL LINKS FOR COMPLETING THIS FORM
The EMBO Journal - Author Guidelines
EMBO Reports - Author Guidelines
Molecular Systems Biology - Author Guidelines
EMBO Molecular Medicine - Author Guidelines

Reporting Checklist for Life Science Articles (updated January
This checklist is adapted from Materials Design Analysis Reporting (MDAR) Checklist for Authors. MDAR establishes a minimum set of requirements in transparent reporting in the life sciences (see Statement of Task: 10.31222/osf.io/9sm4x). Please follow the journal's guidelines in preparing your
Please note that a copy of this checklist will be published alongside your article.

Abridged guidelines for figures
1. Data
The data shown in figures should satisfy the following conditions:
- the data were obtained and processed according to the field's best practice and are presented to reflect the results of the experiments in an accurate and unbiased manner.
- ideally, figure panels should include only measurements that are directly comparable to each other and obtained with the same assay.
- plots include clearly labeled error bars for independent experiments and sample sizes. Unless justified, error bars should not be shown for technical
- if n<5, the individual data points from each experiment should be plotted. Any statistical test employed should be justified.
- Source Data should be included to report the data underlying figures according to the guidelines set out in the authorship guidelines on Data

2. Captions
Each figure caption should contain the following information, for each panel where they are relevant:
- a specification of the experimental system investigated (eg cell line, species name).
- the assay(s) and method(s) used to carry out the reported observations and measurements.
- an explicit mention of the biological and chemical entity(ies) that are being measured.
- an explicit mention of the biological and chemical entity(ies) that are altered/varied/perturbed in a controlled manner.
- the exact sample size (n) for each experimental group/condition, given as a number, not a range;
- a description of the sample collection allowing the reader to understand whether the samples represent technical or biological replicates (including how many animals, litters, cultures, etc.).
- a statement of how many times the experiment shown was independently replicated in the laboratory.
- definitions of statistical methods and measures:
  - common tests, such as t-test (please specify whether paired vs. unpaired), simple χ2 tests, Wilcoxon and Mann-Whitney tests, can be unambiguously identified by name only, but more complex techniques should be described in the methods section;
  - are tests one-sided or two-sided?
  - are there adjustments for multiple comparisons?
  - exact statistical test results, e.g., P values = x but not P values < x;
  - definition of 'center values' as median or average;
  - definition of error bars as s.d. or s.e.m.

Please complete ALL of the questions below.
Select "Not Applicable" only when the requested information is not relevant for your study.

Materials

Newly Created Materials	Information included in the manuscript?	In which section is the information available? (Reagents and Tools Table, Materials and Methods, Figures, Data Availability Section)
New materials and reagents need to be available; do any restrictions apply?	Yes	Material and Methods

Antibodies	Information included in the manuscript?	In which section is the information available? (Reagents and Tools Table, Materials and Methods, Figures, Data Availability Section)
For **antibodies** provide the following information:<br>- Commercial antibodies: RRID (if possible) or supplier name, catalogue number and or/clone number<br>- Non-commercial: RRID or citation	Yes	Material and Methods

DNA and RNA sequences	Information included in the manuscript?	In which section is the information available? (Reagents and Tools Table, Materials and Methods, Figures, Data Availability Section)
**Short novel DNA or RNA including primers, probes**: provide the sequences.	Yes	Appendix Table S1

Cell materials	Information included in the manuscript?	In which section is the information available? (Reagents and Tools Table, Materials and Methods, Figures, Data Availability Section)
**Cell lines:** Provide species information, strain. Provide accession number in repository **OR** supplier name, catalog number, clone number, and/**OR** RRID.	Yes	Material and Methods
**Primary cultures:** Provide species, strain, sex of origin, genetic modification status.	Yes	Material and Methods
Report if the cell lines were recently **authenticated** (e.g., by STR profiling) and tested for mycoplasma contamination.	Yes	Material and Methods

Experimental animals	Information included in the manuscript?	In which section is the information available? (Reagents and Tools Table, Materials and Methods, Figures, Data Availability Section)
**Laboratory animals or Model organisms:** Provide species, strain, sex, age, genetic modification status. Provide accession number in repository **OR** supplier name, catalog number, clone number, **OR** RRID.	Yes	Material and Methods
**Animal observed in or captured from the field:** Provide species, sex, and age where possible.	Not Applicable	
Please detail **housing and husbandry conditions**.	Yes	Material and Methods

Plants and microbes	Information included in the manuscript?	In which section is the information available? (Reagents and Tools Table, Materials and Methods, Figures, Data Availability Section)
**Plants:** provide species and strain, ecotype and cultivar where relevant, unique accession number if available, and source (including location for collected wild specimens).	Not Applicable	
**Microbes:** provide species and strain, unique accession number if available, and source.	Yes	Material and Methods

Human research participants	Information included in the manuscript?	In which section is the information available? (Reagents and Tools Table, Materials and Methods, Figures, Data Availability Section)
If collected and within the bounds of privacy constraints report on age, sex and gender or ethnicity for all study participants.	Not Applicable	

Core facilities	Information included in the manuscript?	In which section is the information available? (Reagents and Tools Table, Materials and Methods, Figures, Data Availability Section)
If your work benefited from core facilities, was their service mentioned in the acknowledgments section?	Yes	Acknowledgement Section

Design

Study protocol	Information included in the manuscript?	In which section is the information available? (Reagents and Tools Table, Materials and Methods, Figures, Data Availability Section)
If study protocol has been **pre-registered, provide DOI in the manuscript**. For clinical trials, provide the trial registration number **OR** cite DOI.	Not Applicable	
Report the **clinical trial registration number** (at ClinicalTrials.gov or equivalent), where applicable.	Not Applicable	

Laboratory protocol	Information included in the manuscript?	In which section is the information available? (Reagents and Tools Table, Materials and Methods, Figures, Data Availability Section)
Provide DOI OR other citation details if **external detailed step-by-step protocols** are available.	Not Applicable	

Experimental study design and statistics	Information included in the manuscript?	In which section is the information available? (Reagents and Tools Table, Materials and Methods, Figures, Data Availability Section)
Include a statement about **sample size** estimate even if no statistical methods were used.	Yes	Material and Methods
Were any steps taken to minimize the effects of subjective bias when allocating animals/samples to treatment (e.g. **randomization procedure**)? If yes, have they been described?	Not Applicable	In Shigella infection model male mice are used and in DSS-induced colitis model female mice are used. The use is based on availibility of mice.
Include a statement about **blinding** even if no blinding was done.	Not Applicable	
Describe **inclusion/exclusion criteria** if samples or animals were excluded from the analysis. Were the criteria pre-established?<br><br>If sample or data points were omitted from analysis, report if this was due to attrition or intentional exclusion and provide justification.	Not Applicable	
For every figure, are **statistical tests** justified as appropriate? Do the data meet the assumptions of the tests (e.g., normal distribution)? Describe any methods used to assess it. Is there an estimate of variation within each group of data? Is the variance similar between the groups that are being statistically compared?	Yes	In figure legends

Sample definition and in-laboratory replication	Information included in the manuscript?	In which section is the information available? (Reagents and Tools Table, Materials and Methods, Figures, Data Availability Section)
In the figure legends: state number of times the experiment was **replicated** in laboratory.	Yes	In figure legends
In the figure legends: define whether data describe **technical or biological replicates**.	Yes	In figure legends

Ethics

Ethics	Information included in the manuscript?	In which section is the information available? (Reagents and Tools Table, Materials and Methods, Figures, Data Availability Section)
Studies involving **human participants**: State details of **authority granting ethics approval** (IRB or equivalent committee(s), provide reference number for approval.	Not Applicable	
Studies involving **human participants**: Include a statement confirming that **informed consent** was obtained from all subjects and that the experiments conformed to the principles set out in the WMA Declaration of Helsinki and the Department of Health and Human Services Belmont Report.	Not Applicable	
Studies involving **human participants:** For publication of **patient photos**, include a statement confirming that consent to publish was obtained.	Not Applicable	
Studies involving experimental **animals**: State details of **authority granting ethics approva**l (IRB or equivalent committee(s), provide reference number for approval. Include a statement of compliance with ethical regulations.	Yes	Material and Methods
Studies involving **specimen and field samples:** State if relevant **permits** obtained, provide details of authority approving study; if none were required, explain why.	Not Applicable	

Dual Use Research of Concern (DURC)	Information included in the manuscript?	In which section is the information available? (Reagents and Tools Table, Materials and Methods, Figures, Data Availability Section)
Could your study fall under dual use research restrictions? Please check biosecurity documents and list of **select agents and toxins** (CDC): https://www.selectagents.gov/sat/list.htm	Not Applicable	
If you used a select agent, is the security level of the lab appropriate and reported in the manuscript?	Not Applicable	
If a study is subject to dual use research of concern regulations, is the name of the **authority granting approval and reference number** for the regulatory approval provided in the manuscript?	Not Applicable	

Reporting

The MDAR framework recommends adoption of discipline-specific guidelines, established and endorsed through community initiatives. Journals have their own policy about requiring specific guidelines and recommendations to complement MDAR.

Adherence to community standards	Information included in the manuscript?	In which section is the information available? (Reagents and Tools Table, Materials and Methods, Figures, Data Availability Section)
State if relevant guidelines or checklists (e.g., **ICMJE, MIBBI, ARRIVE, PRISMA**) have been followed or provided.	Not Applicable	
For **tumor marker prognostic studies,** we recommend that you follow the **REMARK** reporting guidelines (see link list at top right). See author guidelines, under 'Reporting Guidelines'. Please confirm you have followed these guidelines.	Not Applicable	
For **phase II and III randomized controlled trials**, please refer to the **CONSORT** flow diagram (see link list at top right) and submit the CONSORT checklist (see link list at top right) with your submission. See author guidelines, under 'Reporting Guidelines'. Please confirm you have submitted this list.	Not Applicable	

Data Availability

Data availability	Information included in the manuscript?	In which section is the information available? (Reagents and Tools Table, Materials and Methods, Figures, Data Availability Section)
Have **primary datasets** been deposited according to the journal's guidelines (see 'Data Deposition' section) and the respective accession numbers provided in the Data Availability Section?	Yes	Material and Methods
Were **human clinical and genomic datasets** deposited in a public access-controlled repository in accordance to ethical obligations to the patients and to the applicable consent agreement?	Not Applicable	
Are **computational models** that are central and integral to a study available without restrictions in a machine-readable form? Were the relevant accession numbers or links provided?	Not Applicable	
If publicly available data were reused, provide the respective **data citations in the reference list**.	Not Applicable	
