# Supplementary material for: Selective autophagy of RIPosomes maintains innate immune homeostasis during bacterial infection
Source: EMBO J. 2022 Oct 11;41(23):e111289. doi: 10.15252/embj.2022111289 (PMC9713718; doi:10.15252/embj.2022111289)
Supplement: Supplementary file 9 — Movie EV1 [file EMBJ-41-e111289-s009.zip › Movie EV1.doc]

**Movie EV1.** 3D rendering of confocal z-stack images (taken using SP8 STED Leica microscope) of THP-1 cells infected with fluorescently labeled *M. tuberculosis* (Green) and immunostained with RIPK2 antibody (Red). DNA is stained with DAPI (Blue).
