# Supplementary material for: Selective autophagy of RIPosomes maintains innate immune homeostasis during bacterial infection
Source: EMBO J. 2022 Oct 11;41(23):e111289. doi: 10.15252/embj.2022111289 (PMC9713718; doi:10.15252/embj.2022111289)
Supplement: Supplementary file 10 — Movie EV2 [file EMBJ-41-e111289-s013.zip › Movie EV2.doc]

**Movie EV2.** 3D rendering of confocal z-stack images (taken using SP8 STED Leica microscope) of HEK293T cells expressing RIPK2 CARD domain. DNA is stained with DAPI (pseudo-colored to red).
