# Supplementary material for: Selective autophagy of RIPosomes maintains innate immune homeostasis during bacterial infection
Source: EMBO J. 2022 Oct 11;41(23):e111289. doi: 10.15252/embj.2022111289 (PMC9713718; doi:10.15252/embj.2022111289)
Supplement: Supplementary file 11 — Movie EV3 [file EMBJ-41-e111289-s021.zip › Movie EV3.doc]

**Movie EV3.** 3D rendering of confocal z-stack images (taken using SP8 STED Leica microscope) of THP-1 cells infected with *S. flexneri*, (8 h) immunostained with IRGM antibody (Green) and DAPI (pseudo-colored to red)
