# Supplementary material for: Selective autophagy of RIPosomes maintains innate immune homeostasis during bacterial infection
Source: EMBO J. 2022 Oct 11;41(23):e111289. doi: 10.15252/embj.2022111289 (PMC9713718; doi:10.15252/embj.2022111289)
Supplement: Supplementary file 12 — Movie EV4 [file EMBJ-41-e111289-s004.zip › Movie EV4.doc]

**Movie EV4.** 3D rendering of confocal z-stack images (taken using SP8 STED Leica microscope) of THP-1 cells infected with *S. flexneri*, (8 h) immunostained with IRGM (Red) and RIPK2 (Green) antibody.
