# Supplementary material for: Selective autophagy of RIPosomes maintains innate immune homeostasis during bacterial infection
Source: EMBO J. 2022 Oct 11;41(23):e111289. doi: 10.15252/embj.2022111289 (PMC9713718; doi:10.15252/embj.2022111289)

Appendix Figure-S1

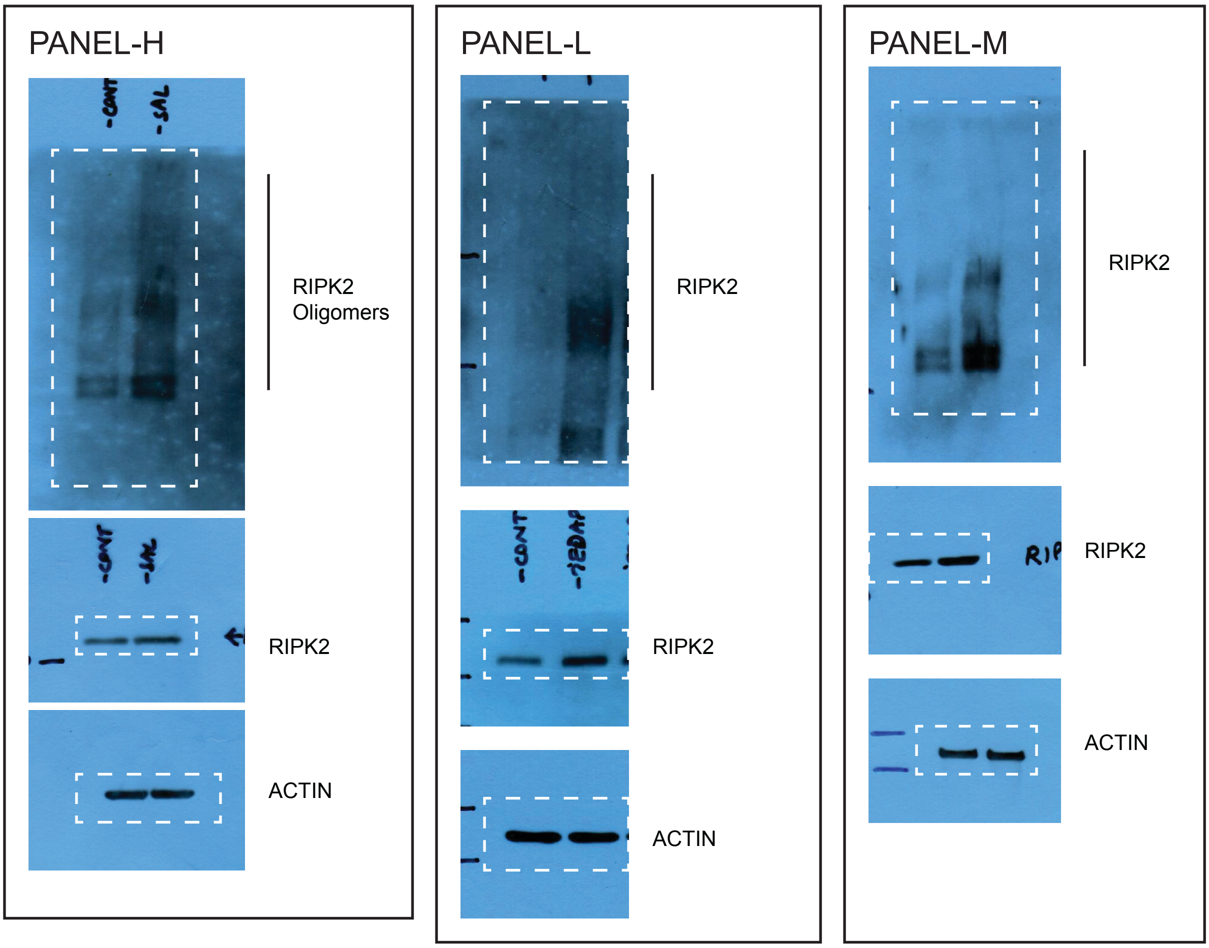

Supplement: Supplementary file 13 — Source Data for Expanded View and Appendix [file EMBJ-41-e111289-s007.zip › Appendix_SD.zip!/Appendix/Appendix Figure-S1/Western Blot/Western Blot Appendix Figure-S1.pdf]
