# Supplementary material for: Selective autophagy of RIPosomes maintains innate immune homeostasis during bacterial infection
Source: EMBO J. 2022 Oct 11;41(23):e111289. doi: 10.15252/embj.2022111289 (PMC9713718; doi:10.15252/embj.2022111289)
Supplement: Supplementary file 13 — Source Data for Expanded View and Appendix [file EMBJ-41-e111289-s007.zip › Expanded_view_SD.zip!/Expanded view /EV-3/Western Blot/Western Blot Figure-EV3_Part-I.pdf]

Figure-EV3

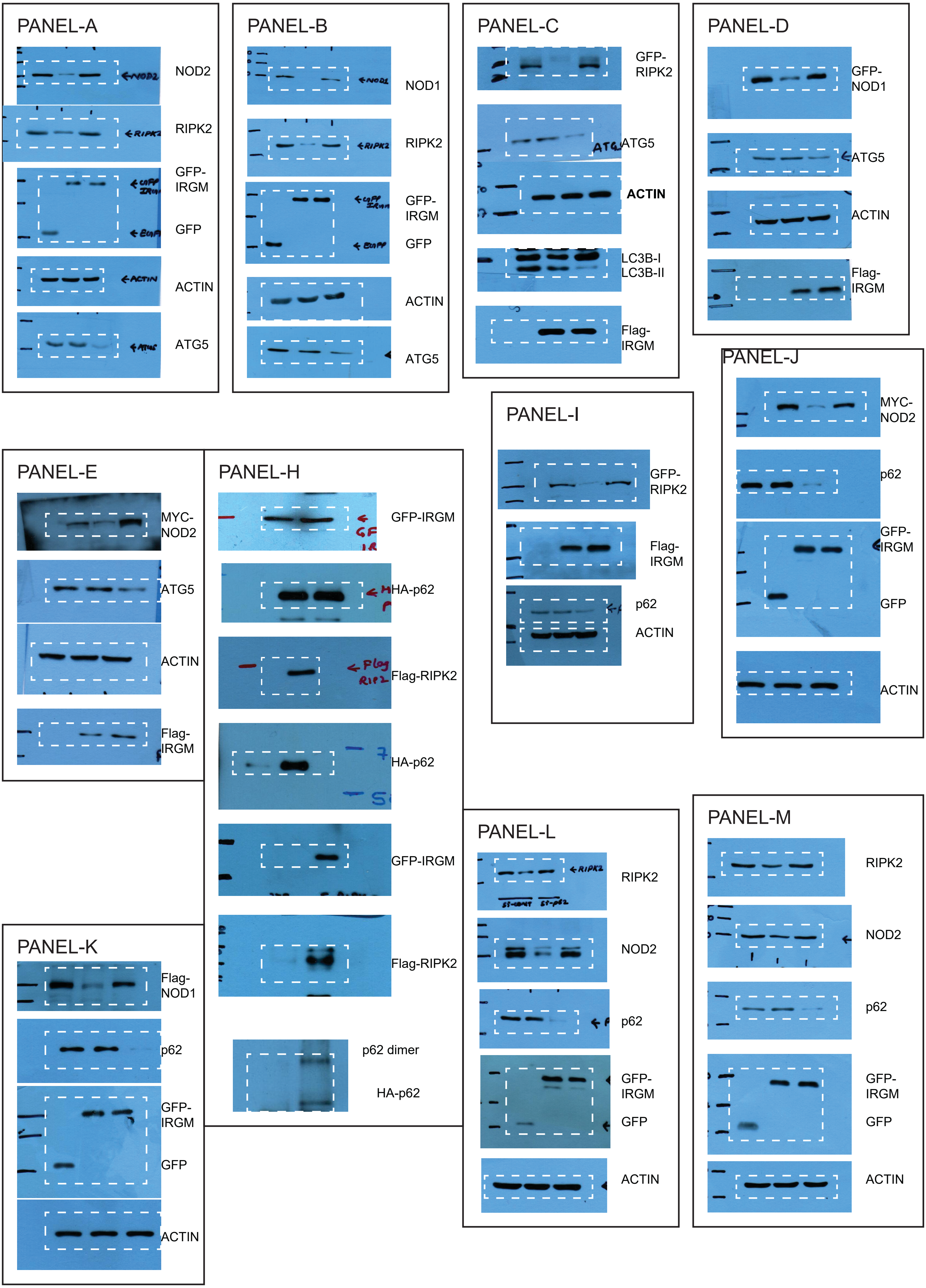
PANEL-A
NOD2
RIPK2
GFP-IRGM
GFP
ACTIN
ATG5
PANEL-B
NOD1
RIPK2
GFP-IRGM
GFP
ACTIN
ATG5
PANEL-C
GFP-RIPK2
ATG5
ACTIN
LC3B-I
LC3B-II
Flag-IRGM
PANEL-D
GFP-NOD1
ATG5
ACTIN
Flag-IRGM
PANEL-E
MYC-NOD2
ATG5
ACTIN
Flag-IRGM
PANEL-H
GFP-IRGM
HA-p62
Flag-RIPK2
HA-p62
GFP-IRGM
Flag-RIPK2
p62 dimer
HA-p62
PANEL-I
GFP-RIPK2
Flag-IRGM
p62
ACTIN
PANEL-J
MYC-NOD2
p62
GFP-IRGM
GFP
ACTIN
PANEL-K
Flag-NOD1
p62
GFP-IRGM
GFP
ACTIN
PANEL-L
RIPK2
NOD2
p62
GFP-IRGM
GFP
ACTIN
PANEL-M
RIPK2
NOD2
p62
GFP-IRGM
GFP
ACTIN
