# Supplementary material for: Selective autophagy of RIPosomes maintains innate immune homeostasis during bacterial infection
Source: EMBO J. 2022 Oct 11;41(23):e111289. doi: 10.15252/embj.2022111289 (PMC9713718; doi:10.15252/embj.2022111289)
Supplement: Supplementary file 13 — Source Data for Expanded View and Appendix [file EMBJ-41-e111289-s007.zip › Expanded_view_SD.zip!/Expanded view /EV-2/Western Blot/Western Blot Figure-EV2.pdf]

Figure-EV2

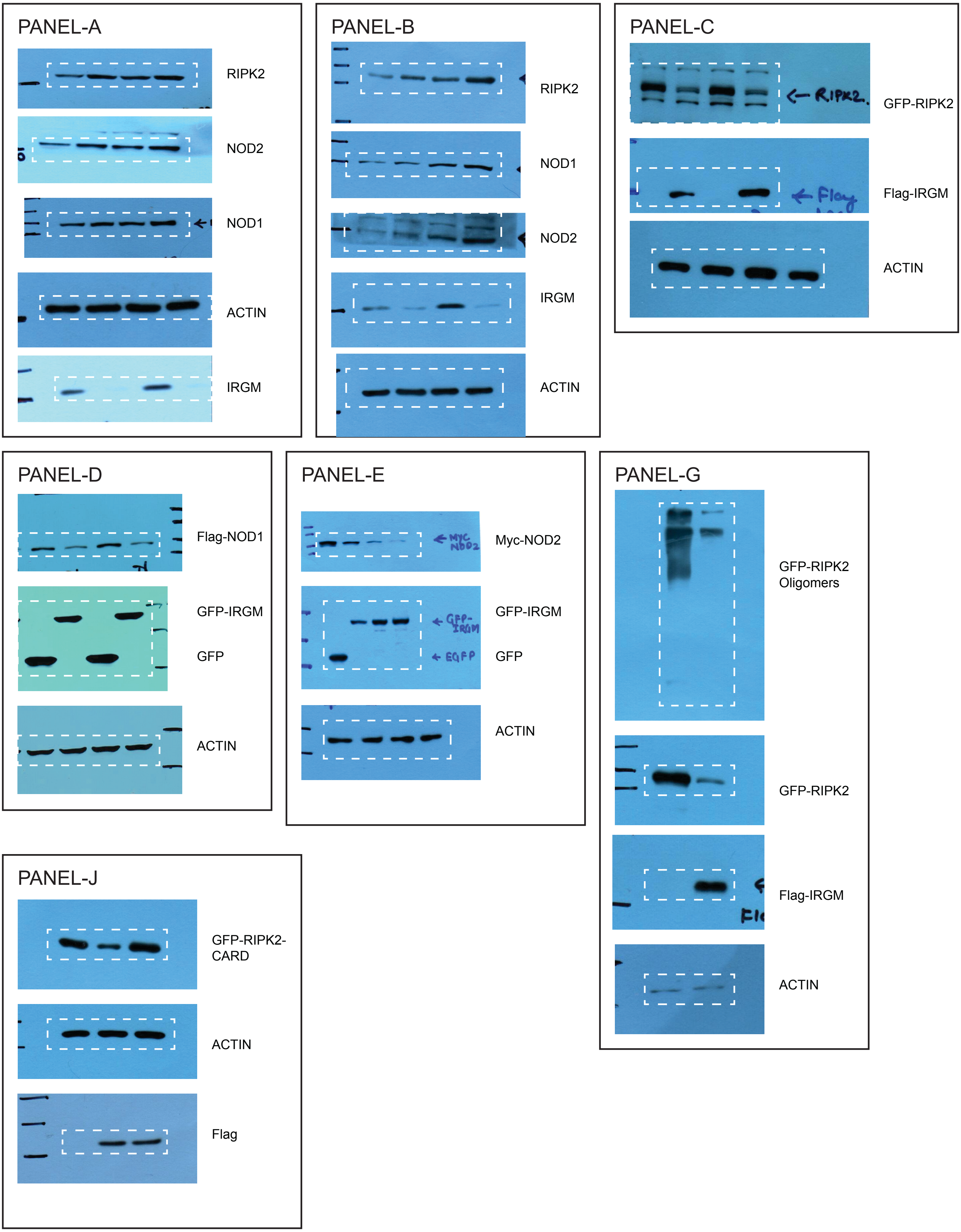
PANEL-A
RIPK2
NOD2
NOD1
ACTIN
IRGM
PANEL-B
RIPK2
NOD1
NOD2
IRGM
ACTIN
PANEL-C
GFP-RIPK2
Flag-IRGM
ACTIN
PANEL-D
Flag-NOD1
GFP-IRGM
GFP
ACTIN
PANEL-E
Myc-NOD2
GFP-IRGM
GFP
ACTIN
PANEL-G
GFP-RIPK2 Oligomers
GFP-RIPK2
Flag-IRGM
ACTIN
PANEL-J
GFP-RIPK2-CARD
ACTIN
Flag
