# Supplementary material for: Selective autophagy of RIPosomes maintains innate immune homeostasis during bacterial infection
Source: EMBO J. 2022 Oct 11;41(23):e111289. doi: 10.15252/embj.2022111289 (PMC9713718; doi:10.15252/embj.2022111289)
Supplement: Supplementary file 13 — Source Data for Expanded View and Appendix [file EMBJ-41-e111289-s007.zip › Expanded_view_SD.zip!/Expanded view /EV-1/Western Blot/Western Blot Figure EV1.pdf]

Figure EV1

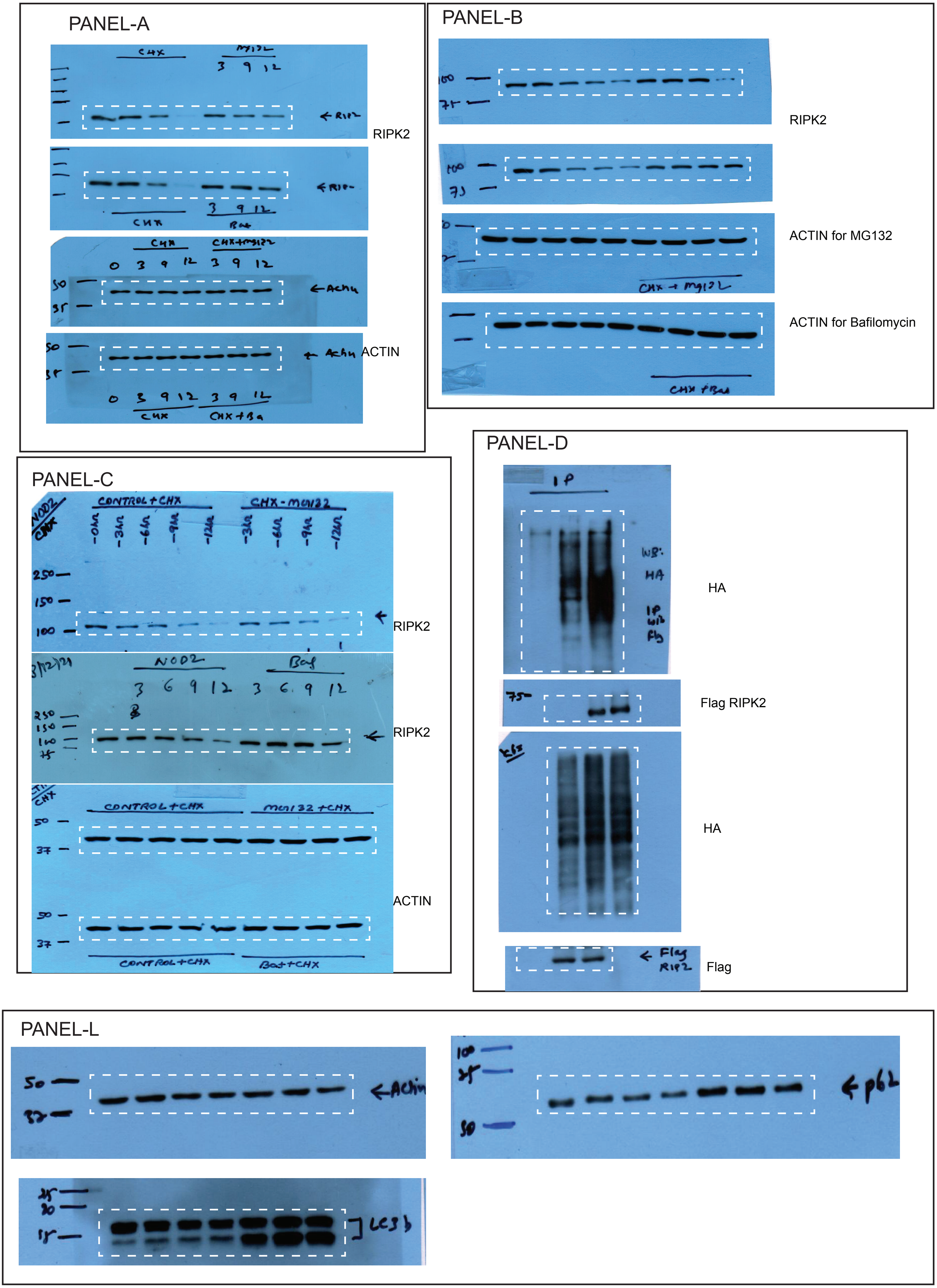

PANEL-A
RIPK2
ACTIN
PANEL-B
RIPK2
ACTIN for MG132
ACTIN for Bafilomycin
PANEL-C
RIPK2
RIPK2
ACTIN
PANEL-D
HA
Flag RIPK2
HA
Flag
PANEL-L
